# Discovery and Semisynthesis of Arthritacins A–J—Terpestacin-Derived Sesterterpenoids from the Plant Endophytic Fungus *Arthrinium* sp. HS66

**DOI:** 10.3390/jof12090693

**Published:** 2026-09-16

**Authors:** Xiao-Zheng Su, Kun Hu, Xing-Ren Li, Han-Dong Sun, Pema-Tenzin Puno

**Affiliations:** 1 State Key Laboratory of Phytochemistry and Natural Medicines, Kunming Institute of Botany, Chinese Academy of Sciences, Kunming 650201, China; suxiaozheng@sjtu.edu.cn (X.-Z.S.); hukun@mail.kib.ac.cn (K.H.); lixingren@mail.kib.ac.cn (X.-R.L.); hdsun@mail.kib.ac.cn (H.-D.S.); 2 Yunnan Key Laboratory of Natural Medicinal Chemistry, Kunming 650201, China

**Keywords:** terpestacin, sesterterpenoids, semisynthesis, endophytic fungus, *Arthrinium* sp. HS66

## Abstract

*Arthrinium* species are promising producers of terpenoids with diverse architectures and noteworthy pharmacological properties. In this work, ten undescribed terpestacin-type sesterterpenoids, arthritacins A–J (**1**–**10**), together with five known analogues (**11**–**15**), were obtained from the endophytic fungus *Arthrinium* sp. HS66 isolated from the stems of *Isodon xerophilus*. Their structures were established through comprehensive spectroscopic analysis, semisynthesis, and NMR calculations, further supported by DP4+ probability analysis. The semisynthetic studies further helped confirm the biosynthetically ambiguous configurations at C-11 and C-23 in terpestacin derivatives. All compounds were evaluated for cytotoxic activity against human neuroblastoma cells. Among them, compounds **1**, **2**, **4**, **6**, **7**, **9**, **12** and **13** showed moderate antiproliferative effects, with IC_50_ values ranging from 21.13 to 36.54 µM.

## 1. Introduction

Endophytic fungi are widely distributed in plant tissues and establish mutualistic interactions with their hosts, while also serving as prolific sources of structurally diverse and bioactive natural products [1,2]. Within this group, the genus *Arthrinium*, which includes about 88 species, is known to generate a wide array of secondary metabolites, particularly terpenoids, alkaloids, and polyketides. These compounds exhibit antitumor, anti-inflammatory, and antibacterial activities [3,4,5,6]. A distinctive subset of *Arthrinium* metabolites is the terpestacin-derived sesterterpenoids, characterized by a rare 5/15 dicyclic framework and an unusual octadecahydrocyclopenta[15]annulene scaffold. Since its initial report in 1993, terpestacin has attracted attention as a novel syncytium formation inhibitor with anti-HIV potential and as a selective inhibitor of tumor angiogenesis via targeting UQCRB of Complex III [7,8,9,10]. Additionally, novel terpestacin derivatives with l-amino acid residue showed promising activities against U87MG-derived glioblastoma stem cells [11]. These remarkable findings have spurred significant interest in its total synthesis and biosynthesis [12,13,14,15].

**Figure 1:**
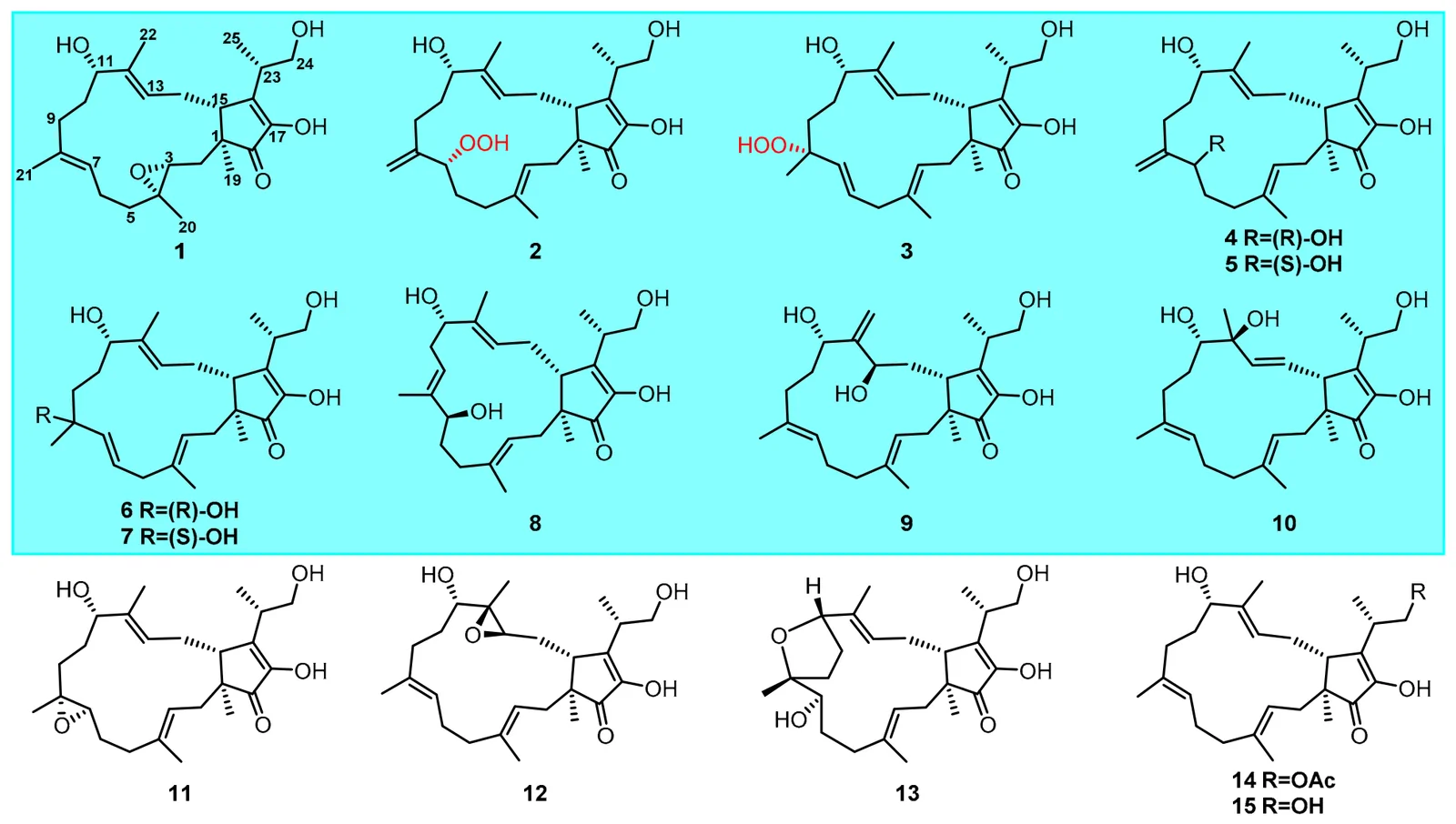
Structures of compounds **1**–**15** (blue highlight indicates new compounds).

However, only about 20 natural terpestacin analogues have been reported to date [16]. Notably, the absolute configurations of most members, especially the chiral centers (C-11 and C-23), were tentatively assigned via biogenetic pathway analysis, lacking robust experimental validation. Furthermore, ROESY correlations could be unreliable for configurational assignment of flexible macrocycles and side chains in previous cases. In our ongoing investigation of structurally diverse secondary metabolites from *Arthrinium* sp. HS66, an endophytic fungus from *Isodon xerophilus* [17], we also discovered ten undescribed terpestacin-type sesterterpenoids named arthritacins A–J (**1**–**10**), as well as five known congeners, including neofusistacin (**11**) [18], maydistacin D (**12**) [19], terpestacin B (**13**) [20], fusaproliferin (**14**) [21] and terpestacin (**15**) [7,8] (Figure 1). More importantly, compounds **2** and **3** represent the first terpestacin derivatives featuring a hydroperoxy group. To uncover the absolute configurations of these terpestacin-derived sesterterpenoids, we employed semisynthesis and quantum chemical calculations as a reliable strategy for structural assignment. This work details the isolation, structural elucidation, semisynthesis and biological evaluation of compounds **1**–**15**.

## 2. Materials and Methods

### 2.1. General Experimental Procedures

UV and ECD spectra were collected using an Applied Photophysics digital circular dichroism spectrometer (Applied Photophysics Ltd., Leatherhead, UK). Optical rotations were determined on a JASCO P-1020 polarimeter (JASCO Corporation, Tokyo, Japan). IR spectra were scanned on a Bruker Tensor 27 spectrophotometer with KBr pellets (Bruker, Billerica, MA, USA). Bruker Avance III 500 and AV 800 spectrometers were used to acquire 1D and 2D NMR spectra, with TMS as the internal standard. Unless otherwise stated, chemical shifts (*δ*) are expressed in ppm relative to solvent signals (methanol-*d*_4_: ^1^H NMR *δ* 3.31 and 4.87 ppm; ^13^C NMR *δ* 49.0 ppm). Data are reported as *δ* (ppm), multiplicity (s = singlet, d = doublet, t = triplet, q = quartet, m = multiplet, br = broad), coupling constants (Hz), and integration.

All transformations were conducted under an argon atmosphere in anhydrous solvents unless noted otherwise. MeOH, THF, MeCN, and all other reagents were obtained from commercial sources at the highest available grade and were used as received, without additional purification, except where stated.

Semipreparative HPLC was performed using Agilent 1100, 1200, and 1260 liquid chromatographs (Agilent, Santa Clara, CA, USA) equipped with an Agilent Zorbax SB-C18 column (9.4 mm × 250 mm). HRESIMS data were acquired on a SCIEX API QSTAR spectrometer (SCIEX, Framingham, MA, USA). Column chromatography was carried out with silica gel (100–200 mesh; Qingdao Marine Chemical, Inc., Qingdao, China). TLC monitoring of reactions and fractions was performed on glass plates precoated with silica gel 60 GF_254_ (0.25 mm; Qingdao Marine Chemical, Inc., Qingdao, China). Spots were visualized under UV light and by heating after spraying with 10% H_2_SO_4_ in EtOH or ammonium cerium nitrate/ammonium molybdate. HPLC solvents were supplied by Merck (Merck, Darmstadt, Germany), and column chromatography solvents were supplied by Liyan Company (Zhengzhou, China).

### 2.2. Fungal Material, Identification, and Fermentation

The fungal strain of *Arthrinium* sp. HS66 was obtained from fresh stems of *Isodon xerophilus* collected in August 2018 at Kunming Botanical Garden, Yunnan Province, China. Its identity was confirmed by ITS rDNA sequence analysis (GenBank Accession No. MT355097) [17]. The fungus was maintained on potato dextrose agar slants at 28 °C for 7 days. Under aseptic conditions, agar plugs were cut into approximately 0.5 × 0.5 × 0.5 cm^3^ pieces, and 15 pieces were introduced into three 500 mL Erlenmeyer flasks, each containing 200 mL medium composed of 0.4% glucose, 1% malt extract, and 0.4% yeast extract. The medium pH was adjusted to 7.0 before autoclaving. The flasks were then incubated at 28 °C on a rotary shaker (170 rpm) for 7 days to prepare the seed culture. Large-scale fermentation was conducted as follows: Solid rice medium was prepared in 125 Fernbach flasks (500 mL) by mixing 80 g rice with 90 mL distilled water, leaving the mixture overnight, and then autoclaving it. Each flask was inoculated with 5.0 mL of the seed culture and kept statically at 28 °C for 30 days [17].

### 2.3. Extraction and Purification

The culture medium was overlaid with MeOH and macerated, and the resulting mixture was filtered and concentrated. The extract was then partitioned with EtOAc, and the organic solvent was removed under reduced pressure to give a crude extract (130 g). This crude extract was fractionated by silica gel column chromatography using a CHCl_3_/Me_2_CO gradient (1:0, 9:1, 8:2, 7:3, 6:4, 1:1, 0:1), producing seven fractions (A–G). Fraction B (15 g, eluted with CHCl_3_/Me_2_CO 9:1) was separated on an RP-18 column with a MeOH/H_2_O gradient (30:70 to 100:0) to give fractions B1–B8. Fraction B5 (3.1 g, MeOH/H_2_O 70:30) was further divided on Sephadex LH-20 (CHCl_3_/MeOH 1:1) into seven subfractions, B5/1–7. Subfraction B5-2 (2.6 g) was subjected to RP-18 chromatography with a MeOH/H_2_O gradient (30:70 to 100:0), affording seventeen fractions (B5-2A–Q); compound **15** (1.5 g) was obtained as a pure substance by recrystallization. B5-2G (26 mg) was purified by semipreparative HPLC on a Zorbax SB-C18 column (3 mL/min, detector UV *λ*_max_ = 270 nm, MeCN/H_2_O 21:79) to yield **8** (1.5 mg, retention time = 54.5 min). B5-2H (72 mg) was purified by semipreparative HPLC on a Zorbax SB-C18 column (3 mL/min, detector UV *λ*_max_ = 270 nm, MeCN/H_2_O 32:68) to yield **4** (7.9 mg, retention time = 32.9 min), **6** (13.6 mg, retention time = 36.2 min), **7** (1.0 mg, retention time = 42.0 min), **2** (1.6 mg, retention time = 49.7 min), **5** (1.9 mg, retention time = 39.2 min), **11** (9.8 mg, retention time = 46.5 min) and **3** (9.3 mg, retention time = 41.5 min). B5-2K (43 mg) was purified by semipreparative HPLC on a Zorbax SB-C18 column (3 mL/min, detector UV *λ*_max_ = 270 nm, MeCN/H_2_O 52:48) to yield **1** (1.1 mg, retention time = 14.5 min) and **13** (1.0 mg, retention time = 18.3 min). B5-2M (29 mg) was purified by semipreparative HPLC on a Zorbax SB-C18 column (3 mL/min, detector UV *λ*_max_ = 270 nm, MeCN/H_2_O 35:65) to yield **9** (0.7 mg, retention time = 29.6 min) and **10** (1.7 mg, retention time = 32.0 min). B5-2N (21 mg) was purified by semipreparative HPLC on a Zorbax SB-C18 column (3 mL/min, detector UV *λ*_max_ = 270 nm, MeCN/H_2_O 40:60) to yield **12** (1.1 mg, retention time = 16.0 min). B5-2P (70 mg) was purified by semipreparative HPLC on a Zorbax SB-C18 column (3 mL/min, detector UV *λ*_max_ = 270 nm, MeCN/H_2_O 35:65) to yield **14** (11 mg, retention time = 31.5 min).

### 2.4. Synthesis of ***1***, ***9***, ***11***–***13***

Terpestacin (**15**) (50.0 mg, 0.12 mmol, 1.0 equiv.) was dissolved in MeOH (1.0 mL) with stirring. To this solution an aqueous solution of NaHCO_3_ (13.6 mg, 0.16 mmol, 1.3 equiv., in 0.1 mL H_2_O) and *m*-CPBA (85%, 33 mg, 1.3 equiv.) were added. The mixture was stirred at 0 °C for 0.5 h. Upon completion (TLC monitoring), saturated aq. Na_2_SO_3_ was added to quench the reaction, and the resulting solution was extracted with EtOAc (3×). The organic phases were combined, washed with brine, dried over anhydrous Na_2_SO_4_, filtered, and evaporated under reduced pressure. Then the crude product was purified by semipreparative HPLC with a Zorbax SB-C18 column (3 mL/min, detector UV *λ*_max_ = 270 nm, MeCN/H_2_O gradient system, from 10:90 to 100:0) to yield **13** (2.1 mg, retention time = 10.6 min, 4% yield), **11** (4.8 mg, retention time = 11.1 min, 9% yield), **1** (1.2 mg, retention time = 12.5 min, 2% yield) and **12** (9.6 mg, retention time = 14.2 min, 19% yield).

Compound **12** (5.0 mg, 0.012 mmol, 1.0 equiv.) was stirred in anhydrous THF (0.25 mL), and 3N HCl in THF (0.25 mL) was added. The reaction was kept at room temperature for 10.0 h. After completion (TLC monitoring), it was quenched with saturated aq. NaHCO_3_ and extracted with EtOAc (3×). The combined organic extracts were washed with brine, dried over Na_2_SO_4_, filtered, and concentrated in vacuo. Then the crude product was purified by semipreparative HPLC with a Zorbax SB-C18 column (1 mL/min, detector UV *λ*_max_ = 270 nm, MeCN/H_2_O, 54:46) to yield **9** (2.5 mg, retention time = 6.3 min, 50% yield).

Synthetic arthritacin A (**1**): White amorphous powder; R_f_ = 0.2 (silica, PE/Me_2_CO 2:1); [*α*${]}_{D}^{20.1}$: −27.0 (MeOH, *c* 0.06); ECD (MeOH) *λ*_max_ (Δ*ε*): 198 (2.39), 210 (0.45), 219 (1.41), 266 (−3.37) and 319 (1.42) nm; UV (MeOH) *λ*_max_ (log *ε*): 195 (2.90) and 265 (3.33) nm; HRESIMS at *m*/*z* 417.2649 ([M − H]^−^, calcd. for 417.2646). For ^1^H and ^13^C NMR data, see Tables S74 and S75.

Synthetic arthritacin I (**9**): White amorphous powder; R_f_ = 0.47 (silica, PE/Me_2_CO 1:1); [*α*${]}_{D}^{20.2}$: 47.60 (MeOH, *c* 0.05); ECD (MeOH) *λ*_max_ (Δ*ε*): 202 (1.98), 215 (−0.29), 256 (0.44), 279 (−0.16) and 318 (1.13) nm; UV (MeOH) *λ*_max_ (log *ε*): 195 (2.94) and 264 (3.38) nm; HRESIMS at *m*/*z* 441.2612 ([M + Na]^+^, calcd. for 441.2611). For ^1^H and ^13^C NMR data, see Tables S76 and S77.

Synthetic neofusistacin (**11**): White amorphous powder; R_f_ = 0.5 (silica, PE/Me_2_CO 1:1); [*α*${]}_{D}^{27.2}$: −3.19 (MeOH, *c* 0.126); ECD (MeOH) *λ*_max_ (Δ*ε*): 200 (−6.92), 225 (1.66), 267 (−4.83) and 320 (1.52) nm; UV (MeOH) *λ*_max_ (log *ε*): 195 (2.95) and 264 (3.30) nm; HRESIMS at *m*/*z* 419.2798 ([M + H]^+^, calcd. for 419.2792). For ^1^H and ^13^C NMR data, see Tables S78 and S79.

Isolated neofusistacin (**11**): White amorphous powder; [*α*${]}_{D}^{27.2}$: −3.62 (MeOH, *c* 0.146); ECD (MeOH) *λ*_max_ (Δ*ε*): 200 (−7.31), 225 (1.79), 266 (−5.16) and 322 (1.60) nm; UV (MeOH) *λ*_max_ (log *ε*): 195 (2.94) and 264 (3.27) nm; HRESIMS at *m*/*z* 419.2798 ([M + H]^+^, calcd. for 419.2792). For ^1^H and ^13^C NMR data, see Tables S78 and S79.

Synthetic maydistacin D (**12**): White amorphous powder; R_f_ = 0.42 (silica, PE/Me_2_CO 1:1); [*α*${]}_{D}^{27.1}$: 7.20 (MeOH, *c* 0.05); ECD (MeOH) *λ*_max_ (Δ*ε*): 198 (2.39), 210 (0.45), 220 (1.41), 266 (−3.37) and 319 (1.42) nm; UV (MeOH) *λ*_max_ (log *ε*): 195 (2.90) and 265 (3.33) nm; HRESIMS at *m*/*z* 441.2613 ([M + Na]^+^, calcd. for 441.2611). For ^1^H and ^13^C NMR data, see Tables S80 and S81.

Isolated maydistacin D (**12**): White amorphous powder; [*α*${]}_{D}^{27.1}$: 7.80 (MeOH, *c* 0.04); ECD (MeOH) *λ*_max_ (Δ*ε*): 199 (3.42), 210 (0.38), 220 (1.67), 266 (−4.96) and 319 (1.66) nm; UV (MeOH) *λ*_max_ (log *ε*): 195 (2.82) and 264 (3.17) nm; HRESIMS at *m*/*z* 441.2618 ([M + Na]^+^, calcd. for 441.2611). For ^1^H and ^13^C NMR data, see Tables S80 and S81.

Synthetic terpestacin B (**13**): White amorphous powder; R_f_ = 0.67 (silica, PE/Me_2_CO 1:1); [*α*${]}_{D}^{25.0}$: −0.53 (MeOH, *c* 0.076); ECD (MeOH) *λ*_max_ (Δ*ε*): 205 (−1.42), 228 (0.32), 259 (−0.42) and 321 (0.47) nm; UV (MeOH) *λ*_max_ (log *ε*): 195 (3.14) and 264 (3.71) nm; HRESIMS at *m*/*z* 419.2800 ([M + H]^+^, calcd. for 419.2792). For ^1^H and ^13^C NMR data, see Tables S82 and S83.

Isolated terpestacin B (**13**): White amorphous powder; [*α*${]}_{D}^{25.0}$: −6.03 (MeOH, *c* 0.063); ECD (MeOH) *λ*_max_ (Δ*ε*): 203 (−1.36), 228 (0.37), 260 (−0.38) and 321 (0.47) nm; UV (MeOH) *λ*_max_ (log *ε*): 195 (3.14) and 265 (3.72) nm; HRESIMS at *m*/*z* 441.2610 ([M + Na]^+^, calcd. for 441.2611). For ^1^H and ^13^C NMR data, see Tables S82 and S83.

### 2.5. Synthesis of ***2***, ***3***, ***16***

A solution of **15** (50.0 mg, 0.12 mmol, 1.0 equiv.) in anhydrous MeCN (0.5 mL) was treated with methylene blue (MB) (2.6 mg, 0.008 mmol) and stirred at room temperature under irradiation from a 1000 W sodium lamp for 10 min. The solvent was promptly removed under reduced pressure, and the resulting residue was purified by semipreparative HPLC on a Zorbax SB-C18 column (3 mL/min, detector UV *λ*_max_ = 270 nm, MeCN/H_2_O 27:73) to yield **2** (4.2 mg, retention time = 34.2 min, 8% yield), **3** (7.9 mg, retention time = 36.5 min, 15% yield) and **16** (1.7 mg, retention time = 39.6 min, 3% yield).

Synthetic arthritacin B (**2**): White amorphous powder; R_f_ = 0.07 (silica, PE/Me_2_CO 2:1); [*α*${]}_{D}^{20.2}$: 24.29 (MeOH, *c* 0.07); ECD (MeOH) *λ*_max_ (Δ*ε*): 204 (−3.99), 224 (0.36), 266 (−3.20) and 323 (1.22) nm; UV (MeOH) *λ*_max_ (log *ε*): 195 (2.76) and 264 (3.32) nm; HRESIMS at *m*/*z* 435.2743 ([M + H]^+^, calcd. for 435.2741). For ^1^H and ^13^C NMR data, see Tables S84 and S85.

Synthetic arthritacin C (**3**): White amorphous powder; R_f_ = 0.57 (silica, PE/Me_2_CO 1:1); [*α*${]}_{D}^{20.0}$: 1.40 (MeOH, *c* 0.1); ECD (MeOH) *λ*_max_ (Δ*ε*): 200 (−3.74), 223 (0.24), 270 (−0.69) and 320 (0.22) nm; UV (MeOH) *λ*_max_ (log *ε*): 195 (3.11) and 266 (3.91) nm; HRESIMS at *m*/*z* 435.2742 ([M + H]^+^, calcd. for 435.2741). For ^1^H and ^13^C NMR data, see Tables S86 and S87.

Synthetic **16**: White amorphous powder; R_f_ = 0.57 (silica, PE/Me_2_CO 1:1); HRESIMS at *m*/*z* 457.2566 ([M + Na]^+^, calcd. for 457.2561). ^1^H NMR (600 MHz, methanol-*d*_4_): *δ*_H_ 2.19 (overlapped, 1H, H-2a), 1.78 (overlapped, 1H, H-2b), 5.18 (t, *J* = 7.4 Hz 1H, H-3), 2.02 (m, 2H, H-5), 1.82 (overlapped, 1H, H-6a), 1.41 (overlapped, 1H, H-6b), 4.24 (dd, *J* = 9.0, 5.4 Hz, 1H, H-7), 2.23 (m, 1H, H-9a), 2.16 (overlapped, 1H, H-9b), 1.78 (overlapped, 1H, H-10a), 1.61 (overlapped, 1H, H-10b), 4.12 (dd, *J* = 7.8, 5.4 Hz, 1H, H-11), 5.37 (dd, *J* = 9.0, 2.4 Hz, 1H, H-13), 2.37 (m, 1H, H-14a), 1.95 (m, 1H, H-14b), 2.63 (overlapped, 1H, H-15), 0.91 (s, 3H, H-19), 1.62 (s, 3H, H-20), 5.13 (d, *J* = 1.0 Hz, 1H, H-21a), 5.10 (d, *J* = 1.0 Hz, 1H, H-21b), 1.60 (s, 3H, H-22), 2.60 (overlapped, 1H, H-23), 3.81 (dd, *J* = 10.4, 7.1 Hz, 1H, H-24a), 3.69 (dd, *J* = 10.4, 6.5 Hz, 1H, H-24b), 1.25 (d, *J* = 7.0 Hz, 3H, H-25). ^13^C NMR (150 MHz, methanol-*d*_4_): *δ*_C_ 50.0 (C, C-1), 39.4 (CH_2_, C-2), 121.8 (CH, C-3), 138.9 (C, C-4), 36.4 (CH_2_, C-5), 31.6 (CH_2_, C-6), 90.7 (CH, C-7), 148.3 (C, C-8), 26.8 (CH_2_, C-9), 34.2 (CH_2_, C-10), 76.5 (CH, C-11), 138.7 (C, C-12), 128.7 (CH, C-13), 29.9 (CH_2_, C-14), 51.2 (CH, C-15), 152.0 (C, C-16), 149.2 (C, C-17), 210.3 (C, C-18), 17.1 (CH_3_, C-19), 16.7 (CH_3_, C-20), 116.1 (CH_2_, C-21), 11.4 (CH_3_, C-22), 38.8 (CH, C-23), 65.9 (CH_2_, C-24), 14.8 (CH_3_, C-25).

### 2.6. Synthesis of ***4***–***6***

Compounds **2** (2.5 mg, 0.0058 mmol, 1.0 equiv.), **16** (1.7 mg, 0.0039 mmol, 1.0 equiv.) and **3** (2.5 mg, 0.0058 mmol, 1.0 equiv.) were each dissolved in anhydrous MeOH (0.5 mL), followed by the addition of triphenylphosphine (1.0 equiv.). The mixtures were stirred at room temperature for 3 h. After concentration, the products were purified by silica gel column chromatography using PE/Me_2_CO (5:1) as the eluent, yielding **4** (2.2 mg, 91% yield), **5** (1.5 mg, 91% yield) and **6** (2.2 mg, 91% yield).

Synthetic arthritacin D (**4**): White amorphous powder; R_f_ = 0.42 (silica, PE/Me_2_CO 1:1); [*α*${]}_{D}^{20.4}$: 6.50 (MeOH, *c* 0.08); ECD (MeOH) *λ*_max_ (Δ*ε*): 203 (−1.45), 221 (0.54), 267 (−0.62) and 318 (0.40) nm; UV (MeOH) *λ*_max_ (log *ε*): 195 (3.34) and 267 (3.88) nm; HRESIMS at *m*/*z* 441.2606 ([M + Na]^+^, calcd. for 441.2611). For ^1^H and ^13^C NMR data, see Tables S88 and S89.

Synthetic arthritacin E (**5**): White amorphous powder; R_f_ = 0.42 (silica, PE/Me_2_CO 1:1); [*α*${]}_{D}^{20.2}$: 18.18 (MeOH, *c* 0.11); ECD (MeOH) *λ*_max_ (Δ*ε*): 205 (−0.96), 223 (1.45), 266 (−2.16) and 323 (1.05) nm; UV (MeOH) *λ*_max_ (log *ε*): 195 (2.84) and 266 (3.31) nm; HRESIMS at *m*/*z* 441.2611 ([M + Na]^+^, calcd. for 441.2611). For ^1^H and ^13^C NMR data, see Tables S90 and S91.

Synthetic arthritacin F (**6**): White amorphous powder; R_f_ = 0.36 (silica, PE/Me_2_CO 1:1); [*α*${]}_{D}^{20.4}$: −28.73 (MeOH, *c* 0.11); ECD (MeOH) *λ*_max_ (Δ*ε*): 199 (−26.27), 227 (1.75), 266 (−1.80) and 324 (0.94) nm; UV (MeOH) *λ*_max_ (log *ε*): 195 (2.90) and 266 (3.38) nm; HRESIMS at *m*/*z* 441.2616 ([M + Na]^+^, calcd. for 441.2611). For ^1^H and ^13^C NMR data, see Tables S92 and S93.

### 2.7. Cytotoxicity Assay Against Human Neuroblastoma Cells

The human neuroblastoma SH-SY5Y cell line was purchased from the American Type Culture Collection (ATCC, Manassas, VA, USA). Cells were maintained in RPMI-1640 or DMEM (HyClone, Logan, UT, USA) supplemented with 10% fetal bovine serum (HyClone) at 37 °C under a humidified 5% CO_2_ atmosphere. Cytotoxic activity was evaluated by the MTS assay [22], using the reagent 3-(4,5-dimethylthiazol-2-yl)-5-(3-carboxymethoxyphenyl)-2-(4-sulfophenyl)-2*H*-tetrazolium (Sigma, St. Louis, MO, USA). In brief, cells were plated in 96-well plates and permitted to adhere for 12 h. Test compounds were then introduced at 40 μM. Following 48 h of incubation, viability was determined with the MTS assay. Compounds producing >50% growth inhibition were further tested at five concentrations (0.064, 0.32, 1.6, 8, and 40 μM) in triplicate, with doxorubicin and paclitaxel (Sigma, St. Louis, MO, USA) serving as positive controls. IC_50_ values were derived using the Reed–Muench method.

### 2.8. Methods for Quantum Chemical Calculations

Conformational space exploration for compounds **1**, **4**, **6**, **8**, and **10** was carried out using the Crest code (version 3.0.1) with the default iMTD-GC protocol [23]. Conformers with a distance matrix deviation below 0.25 Å and an energy difference below 0.25 kcal/mol were considered duplicates, and the higher-energy one was discarded. Following clustering, conformers lying within a 5 kcal/mol energy window were submitted to DFT geometry optimization at the B3LYP-D3BJ/6-31G(d) level. Harmonic frequency calculations were performed at the same level to verify that all optimized conformers corresponded to genuine local minima on the potential energy surface. Subsequently, single-point energies were computed at the M06-2X-D3/def2-TZVP level. The Gibbs free energy of each conformer was obtained by combining the thermal Gibbs free energy correction from frequency analysis with the electronic energy from the single-point calculation. The equilibrium population of each conformer at room temperature (298.15 K) was determined according to the Boltzmann distribution law:

$$p_{i}=\;\frac{n_{i}}{\sum_{j}n_{j}}=\;\frac{e^{-\Delta G_{i}/RT}}{\sum_{j}e^{-\Delta G_{j}/RT}}$$

where *P_i_* is the population of the *i*th conformer; *n_i_* is the number of molecules in the *i*th conformer; Δ*G* is the relative Gibbs free energy (kcal/mol); *T* is the room temperature (298.15 K); and *R* is the ideal gas constant (0.0019858995).

NMR shielding tensors for all dereplicated conformers were computed using the GIAO approach at the mPW1PW91-SCRF/6-31+G(d,p) level, with methanol described by the IEFPCM solvation model. For each candidate, linear regression of calculated against experimental chemical shifts (*δ*cal = a*δ*exp + b) provided the parameters a and b and the correlation coefficient R^2^; the mean absolute error (*MAE*), defined as Σn |*δ*cal − *δ*exp|/n, and the corrected mean absolute error, *CMAE*, defined as Σn |*δ*corr − *δ*exp|/n, where *δ*corr = (*δ*cal-b)/a, were calculated [24,25]. Then, the DP4+ probability analysis [26] was undertaken with the EXCEL spreadsheet provided by Sarotti, A.M. et al. The geometry optimization and single-point energy calculations were all completed in the Gaussian 16 program [27].

### 2.9. Physical Constants and Spectroscopic Data of Compounds

Arthritacin A (**1**): White amorphous powder; [*α*${]}_{D}^{25.4}$: −19.89 (MeOH, *c* 0.11); ECD (MeOH) *λ*_max_ (Δ*ε*): 199 (3.42), 210 (0.38), 220 (1.67), 266 (−4.96) and 319 (1.66) nm; UV (MeOH) *λ*_max_ (log *ε*): 195 (4.42) and 264 (4.07) nm; IR (*ν*_max_): 3424, 2934, 1701, 1652, 1385 and 1034 cm^–1^. HRESIMS at *m*/*z* 441.2609 ([M + Na]^+^, calcd. for 441.2611). For ^1^H and ^13^C NMR data, see Table 1 and Table 2.

Arthritacin B (**2**): White amorphous powder; [*α*${]}_{D}^{25.5}$: 12.63 (MeOH, *c* 0.08); ECD (MeOH) *λ*_max_ (Δ*ε*): 204 (−1.15), 222 (0.01), 261 (−0.88) and 321 (0.39) nm; UV (MeOH) *λ*_max_ (log *ε*): 195 (4.22) and 263 (3.62) nm; IR (*ν*_max_): 3422, 2937, 1699, 1649, 1384 and 1035 cm^–1^. HRESIMS at *m*/*z* 457.2555 ([M + Na]^+^, calcd. for 457.2561). For ^1^H and ^13^C NMR data, see Table 1 and Table 2.

Arthritacin C (**3**): White amorphous powder; [*α*${]}_{D}^{20.6}$: 2.25 (MeOH, *c* 0.16); ECD (MeOH) *λ*_max_ (Δ*ε*): 201 (−5.16), 218 (0.48), 267 (−1.19) and 321 (0.66) nm; UV (MeOH) *λ*_max_ (log *ε*): 195 (4.24) and 264 (3.73) nm; IR (*ν*_max_): 3426, 2936, 1710, 1630, 1384 and 1040 cm^–1^. HRESIMS at *m*/*z* 433.2601 ([M − H]^−^, calcd. for 433.2596). For ^1^H and ^13^C NMR data, see Table 1 and Table 2.

Arthritacin D (**4**): White amorphous powder; [*α*${]}_{D}^{24.5}$: 24.39 (MeOH, *c* 0.15); ECD (MeOH) *λ*_max_ (Δ*ε*): 203 (−6.90), 221 (0.67), 266 (−3.50) and 320 (0.96) nm; UV (MeOH) *λ*_max_ (log *ε*): 195 (2.86) and 266 (3.31) nm; IR (*ν*_max_): 3406, 2938, 1696, 1649, 1405 and 1040 cm^–1^. HRESIMS at *m*/*z* 441.2609 ([M + Na]^+^, calcd. for 441.2611). For ^1^H and ^13^C NMR data, see Table 1 and Table 2.

Arthritacin E (**5**): White amorphous powder; [*α*${]}_{D}^{25.1}$: 29.26 (MeOH, *c* 0.07); ECD (MeOH) *λ*_max_ (Δ*ε*): 204 (−1.86), 221 (0.75), 265 (−2.40) and 324 (0.69) nm; UV (MeOH) *λ*_max_ (log *ε*): 195 (2.85) and 266 (3.33) nm; IR (*ν*_max_): 3424, 2935, 1698, 1648, 1385 and 1033 cm^–1^. HRESIMS at *m*/*z* 441.2608 ([M + Na]^+^, calcd. for 441.2611). For ^1^H and ^13^C NMR data, see Table 1 and Table 2.

Arthritacin F (**6**): White amorphous powder; [*α*${]}_{D}^{25.7}$: −31.07 (MeOH, *c* 0.15); ECD (MeOH) *λ*_max_ (Δ*ε*): 199 (−38.98), 228 (1.87), 266 (−2.99) and 324 (0.97) nm; UV (MeOH) *λ*_max_ (log *ε*): 195 (2.82) and 264 (3.22) nm; IR (*ν*_max_): 3413, 2935, 1699, 1653, 1384 and 1037 cm^–1^. HRESIMS at *m*/*z* 441.2606 ([M + Na]^+^, calcd. for 441.2611). For ^1^H and ^13^C NMR data, see Table 3 and Table 4.

Arthritacin G (**7**): White amorphous powder; [*α*${]}_{D}^{26.8}$: −2.79 (MeOH, *c* 0.033); ECD (MeOH) *λ*_max_ (Δ*ε*): 201 (−4.29), 217 (0.53), 269 (−0.63) and 324 (0.44) nm; UV (MeOH) *λ*_max_ (log *ε*): 195 (3.00) and 263 (3.70) nm; IR (*ν*_max_): 3399, 2977, 1650, 1384 and 1047 cm^–1^. HRESIMS at *m*/*z* 417.2646 ([M − H]^−^, calcd. for 417.2651). For ^1^H and ^13^C NMR data, see Table 3 and Table 4.

Arthritacin H (**8**): White amorphous powder; [*α*${]}_{D}^{24.2}$: 57.27 (MeOH, *c* 0.06); ECD (MeOH) *λ*_max_ (Δ*ε*): 202 (−4.94), 217 (0.39), 266 (−1.30) and 320 (0.80) nm; UV (MeOH) *λ*_max_ (log *ε*): 195 (2.83) and 264 (3.42) nm; IR (*ν*_max_): 3434, 2933, 1696, 1649, 1384 and 1035 cm^–1^. HRESIMS at *m*/*z* 441.2612 ([M + Na]^+^, calcd. for 441.2611). For ^1^H and ^13^C NMR data, see Table 3 and Table 4.

Arthritacin I (**9**): White amorphous powder; [*α*${]}_{D}^{25.6}$: 90.74 (MeOH, *c* 0.07); ECD (MeOH) *λ*_max_ (Δ*ε*): 203 (2.44), 215 (−1.05), 259 (0.19), 275 (−0.59) and 320 (1.66) nm; UV (MeOH) *λ*_max_ (log *ε*): 195 (2.66) and 264 (3.13) nm; IR (*ν*_max_): 3401, 2928, 1694, 1647, 1384 and 1019 cm^–1^. HRESIMS at *m*/*z* 441.2614 ([M + Na]^+^, calcd. for 441.2611). For ^1^H and ^13^C NMR data, see Table 3 and Table 4.

Arthritacin J (**10**): White amorphous powder; [*α*${]}_{D}^{24.7}$: −6.36 (MeOH, *c* 0.1); ECD (MeOH) *λ*_max_ (Δ*ε*): 200 (9.67), 217 (−6.57), 243 (−2.44), 264 (−3.57) and 318 (2.32) nm; UV (MeOH) *λ*_max_ (log *ε*): 195 (2.79) and 266 (3.24) nm; IR (*ν*_max_): 3425, 2930, 1697, 1649, 1384 and 1033 cm^–1^. HRESIMS at *m*/*z* 441.2618 ([M + Na]^+^, calcd. for 441.2611). For ^1^H and ^13^C NMR data, see Table 3 and Table 4.

## 3. Results and Discussion

### 3.1. Structural Characterization

**Figure 2:**
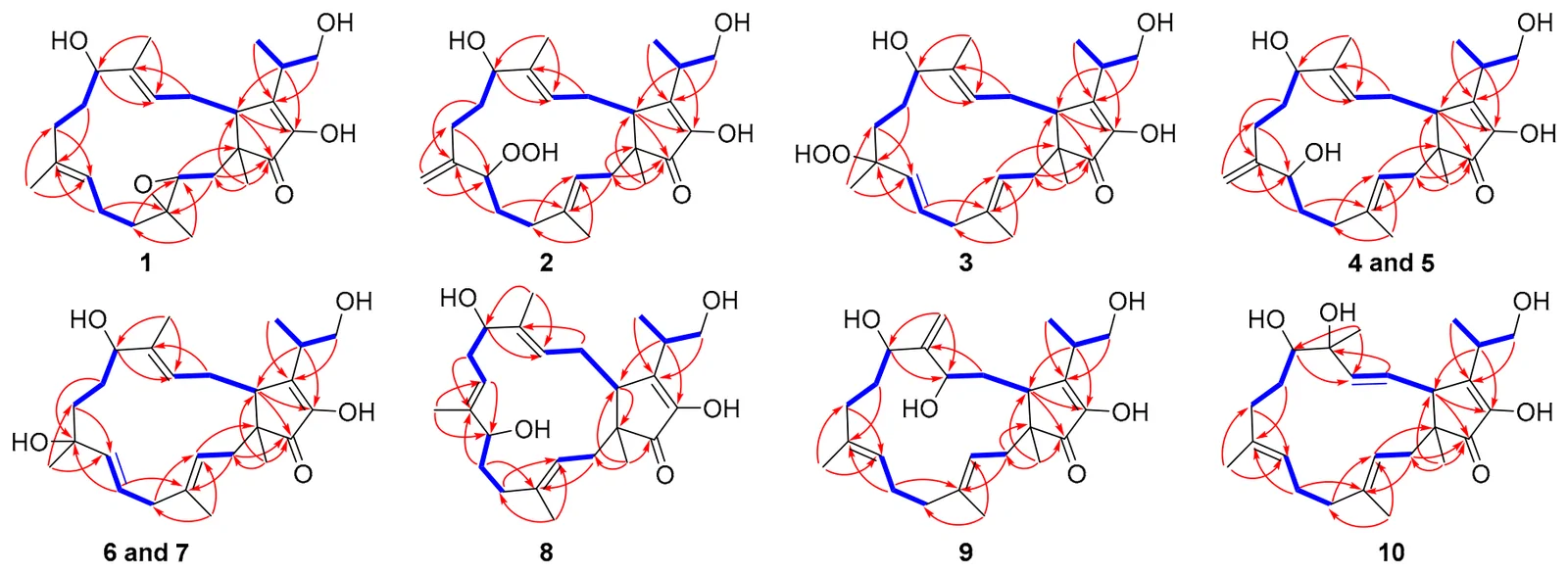
Key HMBC (red arrows) and ^1^H-^1^H COSY (blue bold lines) correlations of compounds **1**–**10**.

Compound **1** was obtained as a white amorphous powder. Its molecular formula, C_25_H_38_O_5_, was established by HRESIMS analysis (observed [M + Na]^+^ ion peak at *m*/*z* 441.2609, calcd. for 441.2611), indicating seven degrees of unsaturation. The ^1^H NMR spectrum of **1** displayed characteristic signals, including two olefinic protons at *δ*_H_ 5.26 (1H, dd, *J* = 8.0, 6.4 Hz) and 5.46 (1H, dd, *J* = 7.6, 1.7 Hz), an oxymethylene at *δ*_H_ 3.84 (1H, dd, *J* = 10.1, 7.2 Hz) and 3.72 (1H, dd, *J* = 10.1, 6.3 Hz), and five methyls at *δ*_H_ 1.12 (3H, s), 1.28 (3H, d, *J* = 7.1 Hz), 1.30 (3H, s), 1.62 (3H, s) and 1.68 (3H, s) (Table 1). The ^13^C NMR and HSQC data revealed 25 carbons, comprising six olefinic (two protonated), three non-protonated (one ketone, one oxygenated), four sp^3^ methines (two oxygenated), seven sp^3^ methylenes (one oxygenated) and five methyls (Table 2). Comparison with terpestacin (**15**) suggested structural similarity, with the key difference being the presence of two oxygenated carbons in **1** (*δ*_C_ 63.8, CH, C-3; *δ*_C_ 63.1, C, C-4) instead of two olefinic carbons (C-3 and C-4) in **15**. Subsequently, the ^1^H-^1^H COSY correlations of H_2_-2/H-3 and H_2_-5/H_2_-6/H-7, along with the HMBC correlations from H_2_-2/H_2_-6 to C-4, H-3 to C-1, H_2_-5/H_3_-20 to C-3, and H_3_-20 to C-5, supported that the planar structure of **1** was formed by epoxidation of the Δ^3,4^-double bond in **15** (Figure 2). Compound **1** was given a trivial name, arthritacin A.

Arthritacin B (**2**) possessed a molecular formula of C_25_H_38_O_6_, as determined by HRESIMS (observed *m*/*z* 457.2555 ([M + Na]^+^, calcd. for 457.2561)), corresponding to seven indices of hydrogen deficiency. Its 1D NMR data closely resembled those of terpestacin, except for a terminal double bond signal (*δ*_C_ 112.9; *δ*_H_ 5.18 and 5.14) and an oxygenated methine replacing a methyl and an olefinic carbon in **15** (Table 1 and Table 2). Then the key COSY cross peaks (H_2_-5/H_2_-6/H-7 and H_2_-9/H_2_-10/H-11) and HMBC correlations (H_2_-6/H_2_-10 to C-8, H_2_-9 to C-7, H_2_-21 to C-7/C-9) confirmed a Δ^8,21^-double bond in **2**, contrasting with the Δ^7,8^-double bond in **15** (Figure 2). Given the molecular formula, unsaturation of degrees, and the unusual oxygenated methine (C-7, *δ*_C_ 87.3), we proposed a hydroperoxy group at C-7.

Arthritacin C (**3**) shared the same molecular formula (C_25_H_38_O_6_, HRESIMS ion peak at *m*/*z* 433.2601, [M − H]^−^) and was identified as a terpestacin-type sesterterpenoid. Its 1D NMR data suggested structural similarity to **15**, but two coupled olefinic protons (*δ*_H_ 5.68 and 5.49) and an oxygenated non-protonated carbon (*δ*_C_ at 85.0) indicated key differences (Table 1 and Table 2). The ^1^H-^1^H COSY correlations of H_2_-5/H-6/H-7 and H_2_-9/H_2_-10/H-11, along with the HMBC correlations from H-6/H_2_-10 to C-8, H_2_-9 to C-7, and H_3_-21 to C-7 and C-9, constructed a Δ^6,7^-double bond and a hydroperoxy at C-8 in **3** (Figure 2). The large coupling constant (*J* = 16.0 Hz) between H-6 and H-7 confirmed the 6*E* configuration.

Compounds **4** and **5**, a pair of epimers, shared the same molecular formula, C_25_H_38_O_5_, as deduced by the HRESIMS data (*m*/*z* 441.2609 and 441.2608 [M + Na]^+^, calcd. for 441.2611), accounting for seven degrees of unsaturation. Their ^1^H NMR and ^13^C NMR data closely matched those of **2**, confirming a terpestacin-derived scaffold. However, the oxygenated methine signal (C-7, *δ*_C_ 87.3 in **2**) shifted to *δ*_C_ 73.9 (**4**) and 77.3 (**5**) (Table 1), indicating a hydroxyl group at C-7 in both compounds. The Δ^8,21^-double bond was corroborated by ^1^H-^1^H COSY and HMBC analyses, leading to the designation of **4** and **5** as arthritacins D and E, respectively (Figure 2).

Arthritacins F and G (**6** and **7**), another pair of epimers, also had the molecular formula C_25_H_38_O_5_, supported by their HRESIMS spectra (*m*/*z* 441.2606 [M + Na]^+^ for **6**, *m*/*z* 417.2646 [M − H]^−^ for **7**), showing seven indices of hydrogen deficiency. Comparison of 1D NMR data of **6** and **7** with **3** suggested that they shared a similar terpestacin-based structure. The main difference was the highfield shift of C-8 (*δ*_C_ 74.3 in **6**, *δ*_C_ 74.4 in **7**) compared to **3** (*δ*_C_ 85.0), indicating a hydroxyl instead of a hydroperoxy group at C-8 (Table 3). The ^1^H-^1^H COSY and HMBC correlations analogous to **3** established the Δ^6,7^-double bond (Figure 2), while the large coupling constant (*J* = 15.6 Hz) between H-6 and H-7 assigned the 6*E* configurations in both compounds.

Compound **8** was isolated as a white amorphous powder, and it was found to possess a molecular formula of C_25_H_38_O_5_ by HRESIMS ([M + Na]^+^ at *m*/*z* 441.2612, calcd. for 441.2611). Analysis of its NMR data revealed a close structural resemblance to **15** (Table 3 and Table 4), with key differences being the migration of the double bond from Δ^7,8^ to Δ^8,9^ and the replacement of a methylene with an oxygenated methine (C-7). These modifications were confirmed by the ^1^H-^1^H COSY cross peaks of H_2_-5/H_2_-6/H-7 and H-9/H_2_-10/H-11 along with the HMBC correlations from H_2_-6/H_2_-10 to C-8, H-9 to C-7 and H_3_-21 to C-7/C-9 (Figure 2).

Compound **9**, with the same molecular formula (C_25_H_38_O_5_, HRESIMS ion peak at *m*/*z* 441.2614 [M + Na]^+^, calcd. for 441.2611), exhibited 1D NMR features similar to **4**, including a terminal double bond and two oxygenated methines. However, the ^1^H-^1^H COSY correlations (H_2_-9/H_2_-10/H-11 and H-13/H_2_-14/H-15) and the HMBC correlations (H-11 to C-13, H_2_-14 to C-12 and H_2_-22 to C-11/C-13) showed the Δ^12,22^-double bond in **9** instead of a Δ^8,21^-double bond in **4** (Figure 2).

Compound **10** also shared the same molecular formula, C_25_H_38_O_5_, as established by HRESIMS analysis ([M + Na]^+^ at *m*/*z* 441.2618, calcd. for 441.2611). Its ^1^H NMR and ^13^C NMR data displayed two coupled olefinic protons (*δ*_H_ 5.63 and 5.28) and an oxygenated non-protonated carbon (*δ*_C_ 76.5), suggesting structural similarity to **6** (Table 3 and Table 4). The Δ^13,14^-double bond in **10** was confirmed by the ^1^H-^1^H COSY correlations of H_2_-9/H_2_-10/H-11 and H-13/H-14/H-15 and the HMBC correlations from H-11 to C-13, H-14 to C-12 and H_3_-22 to C-11/C-13 (Figure 2). The large coupling constant (*J* = 15.6 Hz) between H-13 and H-14 established the 13*E* configuration. Finally, compounds **8**–**10** were named as arthritacins H–J, respectively.

### 3.2. Semisynthetic Studies

**Scheme 1:**
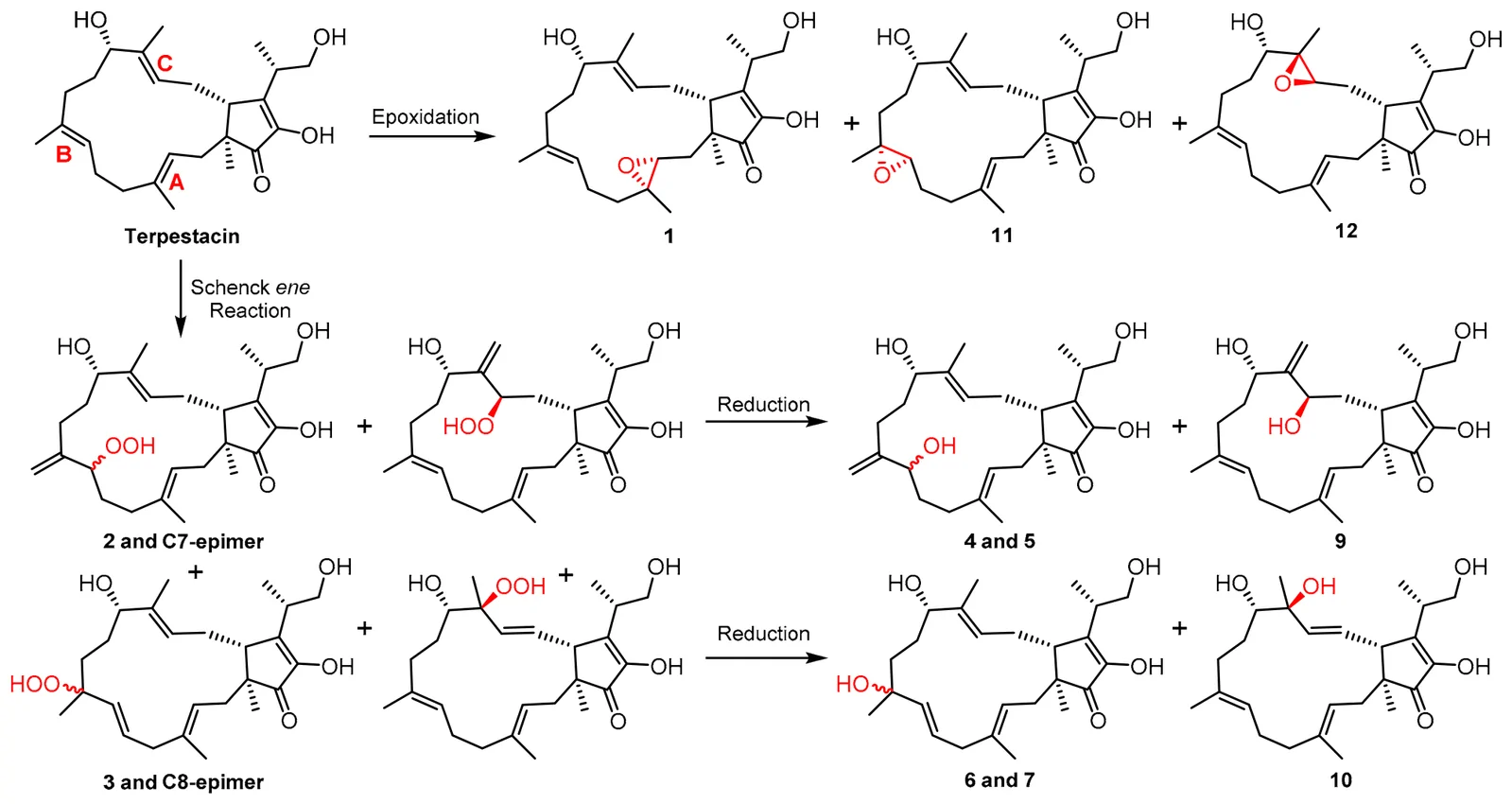
The biosynthetic pathways of compounds **1**–**10**.

Compounds **1**, **11** and **12** were biosynthetically derived from the selective epoxidation of three double bonds (A–C) in terpestacin, while the remaining compounds arose via spontaneous Schenck *ene* reactions and successive reductions at these double bonds (Scheme 1). Consequently, the configurations of biogenically conserved chiral centers (C-1, C-11, C-15 and C-23) in the derivatives were presumed to be inherited from terpestacin, though further experimental validation was required. Given an octadeca-hydrocyclopenta[15]annulene fragment and a flexible side chain coexisted in these molecules, ROESY correlations alone were deemed unreliable for configuration assignment. To resolve this, we pursued the semisynthesis of some isolated derivatives from terpestacin (the major constituent of our strain, yield: 0.15 g/Kg cultures). These efforts would unambiguously confirm the biogenically conserved configurations and facilitate the absolute configurational assignment of all compounds through quantum chemical calculations.

**Scheme 2:**
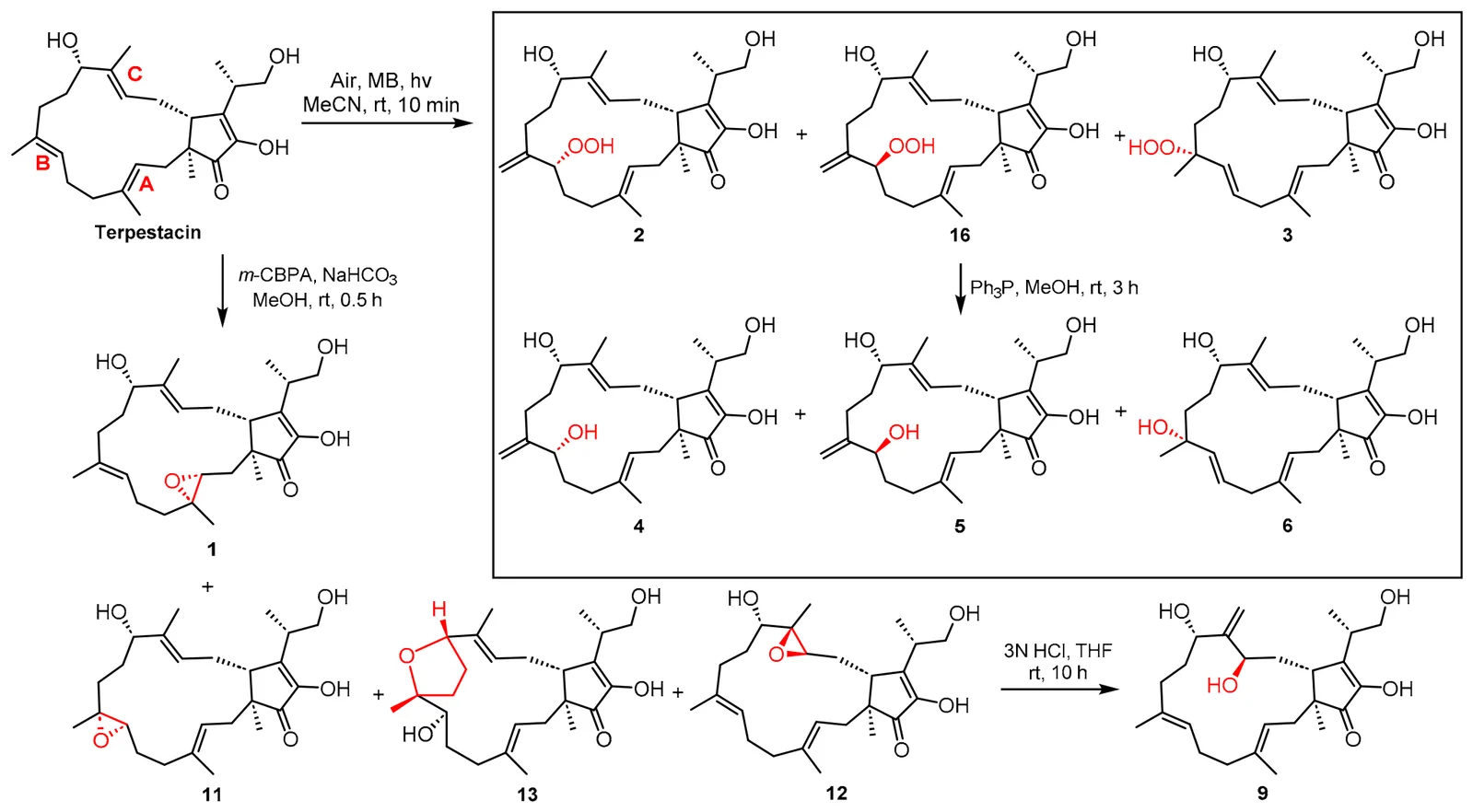
Semisynthesis of **1**–**6**, **9**, **11**–**13** and **16** from **15**.

To synthesize the target compounds **1**, **11** and **12**, we employed oxidant *m*-chloroperoxybenzoic acid (*m*-CPBA) for the epoxidation of terpestacin (Scheme 2). Surprisingly, this attempt not only afforded the desired products but also generated **13** (4% yield). The NMR data, specific rotation and ECD curves of the synthesized compounds were in good agreement with those of natural isolated counterparts. Subsequently, **9** was obtained from its precursor **12** via treatment with 3N HCl, following a reported procedure [28] (Scheme 2). The absolute configuration of **9** was determined as 1*S*, 11*S*, 13*R*, 15*R*, 23*S*, 3*E*, and 7*E*, based on the mechanism of acid-induced epoxy ring opening.

Since compounds **2** and **3** arose from a Schenck *ene* reaction of the Δ^7,8^-double bond of terpestacin, we explored the sensitized photooxygenation of **15** using single oxygen (1O_2_) [29,30,31]. The reaction mixture, containing **15** and methylene blue (MB) as a sensitizer, was irradiated under air with a 1000 W high-pressure sodium lamp, successfully yielding targeted peroxides **2** and **3** (Scheme 2). Their spectral properties (NMR data and specific rotation) aligned well with those of the authentic natural sample. Additionally, we obtained **16**, an epimer of **2** at C-7, while the epimer of **3** could not be purified due to its negligible yield. Next, we reduced these peroxides using triphenylphosphine (Ph_3_P) to afford desired **4**, **5** and **6** [32] (Scheme 2). Collectively, these semisynthetic efforts yielded ten isolated terpestacin derivatives, providing strong support for the proposed configurations of the biogenically conserved stereocenters (C-1, C-11, C-15 and C-23) in all new compounds.

Regarding the low yields of the natural arthritacins A–J, together with the semisynthetic results in which terpestacin was converted to arthritacins A–J (**1**–**10**), neofusistacin (**11**), maydistacin D (**12**), and terpestacin B (**13**) via epoxidation and the Schenck *ene* reaction, we preliminarily propose that these compounds are products of non-enzymatic spontaneous oxidation. This proposal is supported by the observation that when sufficient purified terpestacin was exposed to air without solvent, compounds **1**–**13** were also detected after several weeks. This finding indicates that the three double bonds in the macrocyclic scaffold of terpestacin are sensitive to oxygen and ambient light, suggesting that terpestacin should be stored under protection from light and oxygen. Furthermore, it points out that structural modifications should target these sensitive double bonds to generate more structurally diverse terpestacin congeners.

### 3.3. Configuration Determination

**Figure 3:**
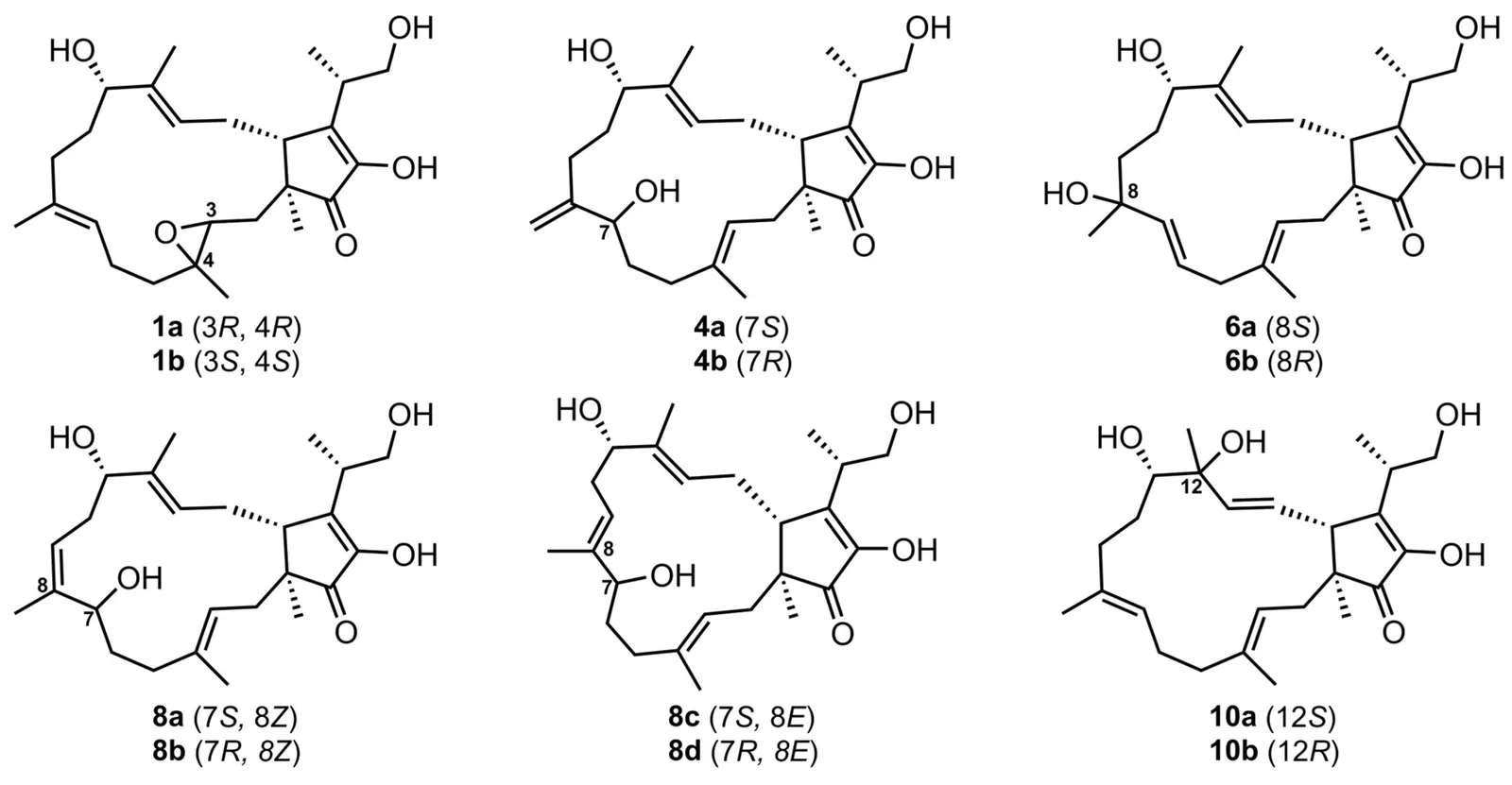
The candidate structures of compounds **1**, **4**, **6**, **8** and **10**.

Based on biosynthetic pathway analysis and semisynthetic results, the absolute configurations of these new terpestacin derivatives were narrowed down to five key compounds (**1**, **4**, **6**, **8** and **10**) with their candidate structures proposed in Figure 3. Due to ambiguous ROESY correlations, the Δ^8,9^-double bond of compound **8** remained uncertain, resulting in four possible candidates. To determine the absolute configurations, we performed NMR chemical shift calculations followed by DP4+ analyses. Statistical evaluation, including correlation coefficient (R^2^), mean absolute errors (MAEs), and corrected mean absolute errors (CMAEs), supported the correct candidates (**1a**, **4b**, **6b**, **8c**, **10a**), which exhibited the highest DP4+ (all data) probabilities (for details, see the Supporting Information and Table 5). Therefore, the absolute configurations of the new compounds were assigned as follows: (1*S*, 3*R*, 4*R*, 11*S*, 15*R*, 23*S*, 7*E*, 12*E*)-**1**, (1*S*, 7*R*, 11*S*, 15*R*, 23*S*, 3*E*, 12*E*)-**2**, (1*S*, 8*R*, 11*S*, 15*R*, 23*S*, 3*E*, 6*E*, 12*E*)-**3**, (1*S*, 7*R*, 11*S*, 15*R*, 23*S*, 3*E*, 12*E*)-**4**, (1*S*, 7*S*, 11*S*, 15*R*, 23*S*, 3*E*, 12*E*)-**5**, (1*S*, 8*R*, 11*S*, 15*R*, 23*S*, 3*E*, 6*E*, 12*E*)-**6**, (1*S*, 8*S*, 11*S*, 15*R*, 23*S*, 3*E*, 6*E*, 12*E*)-**7**, (1*S*, 7*S*, 11*S*, 15*R*, 23*S*, 3*E*, 8*E*, 12*E*)-**8**, (1*S*, 11*S*, 12*S*, 15*R*, 23*S*, 3*E*, 7*E*, 13*E*)-**10**.

### 3.4. Cytotoxic Activities

All isolates (**1**–**15**) were evaluated for cytotoxicity against the human neuroblastoma cell line SH-SY5Y, using doxorubicin and paclitaxel as positive controls. Among them, eight compounds, including **1**, **2**, **4**, **6**, **7**, **9**, **12** and **13,** exhibited moderate cytotoxic activity, with IC_50_ values ranging from 21.13 to 36.54 µM (Table 6). Preliminary structure–activity relationships can be drawn from the cytotoxic results (Table 6). The parent terpestacin (**15**) and its 24-O-acetyl congener fusaproliferin (**14**) were both inactive (IC_50_ > 40 µM), indicating that the intact macrocyclic triene is insufficient for activity and that the free C-24 hydroxyl is not essential. In contrast, all compounds bearing an additional oxygen functionality on the macrocycle, except **3**, **5**, **8**, **10** and **11**, showed moderate cytotoxicity, revealing that oxidative modification of the macrocyclic double bonds is required for activity against SH-SY5Y cells. This result suggests a possible orientation for structural optimization.

This study represents the first report of terpestacin-type sesterterpenoids demonstrating inhibitory effects against neuroblastoma, a common malignant tumor frequently occurring in children. These findings suggest that this class of compounds may serve as a promising chemical scaffold for the development of novel anti-neuroblastoma agents.

## 4. Conclusions

Fifteen terpestacin-type sesterterpenoids, including ten new ones designated as arthritacins A–J (**1**–**10**), were isolated from the plant endophytic fungus *Arthrinium* sp. HS66. Notably, compounds **2** and **3** represent the first terpestacin derivatives characterized by a unique hydroperoxy group. Through semisynthesis starting from terpestacin, ten congeners were obtained, enabling the unambiguous assignment of configurations at the biogenically conserved stereocenters (C-1, C-11, C-15 and C-23) in terpestacin analogues. Based on these results, the NMR calculations coupled with DP4+ analyses further confirmed the absolute configurations of all new compounds. These findings establish a robust structural framework for future investigation into the biological functions of terpestacin derivatives.

## Figures and Tables

**Table 1:** ^1^H NMR data of compounds **1**–**5**.

No.	1 ^a,b^	2 ^a,b^	3 ^a,c^	4 ^a,c^	5 ^a,b^
	*δ*_H_ (*J* in Hz)	*δ*_H_ (*J* in Hz)	*δ*_H_ (*J* in Hz)	*δ*_H_ (*J* in Hz)	*δ*_H_ (*J* in Hz)
2	1.64 (m)	2.23 (dd, 14.3, 9.1)	2.15 (dd, 14.1, 7.4)	2.23 (overlapped)	2.18 (overlapped)
	1.54 (dd, 14.3, 1.4)	1.78 (overlapped)	1.90 (overlapped)	1.80 (overlapped)	1.79 (overlapped)
3	2.98 (dd, 8.6, 2.3)	5.16 (m)	5.24 (m)	5.16 (overlapped)	5.20 (t, 7.0)
5	2.11 (overlapped)	2.11 (m)	2.77 (d, 6.5)	2.11 (t, 7.2)	2.03 (overlapped)
	1.21 (td, 13.5, 2.6)				1.99 (overlapped)
6	2.33 (m)	1.95 (overlapped)	5.68 (dt, 16.0, 6.6)	1.86 (overlapped)	1.76 (overlapped)
	2.05 (overlapped)	1.80 (overlapped)		1.70 (m)	1.56 (m)
7	5.26 (dd, 8.0, 6.4)	4.13 (dd, 7.9, 4.7)	5.49 (dt, 16.0, 1.1)	3.96 (t, 5.9)	4.03 (dd, 8.0, 6.0)
9	2.11 (overlapped)	2.28 (m)	1.99 (m)	2.25 (overlapped)	2.22 (overlapped)
	1.82 (overlapped)	2.05 (m)	1.53 (m)	2.02 (overlapped)	2.17 (overlapped)
10	1.83 (overlapped)	1.84 (m)	1.66 (overlapped)	1.84 (overlapped)	1.78 (overlapped)
	1.68 (m)	1.64 (overlapped)	1.59 (overlapped)	1.64 (overlapped)	1.65 (m)
11	3.93 (dd, 10.9, 3.3)	4.07 (t, 6.5)	3.92 (dd, 9.1, 3.7)	4.09 (t, 6.6)	4.09 (dd, 7.5, 5.5)
13	5.46 (dd, 7.6, 1.7)	5.36 (dd, 9.0, 2.3)	5.28 (dd, 8.7, 2.1)	5.37 (dd, 9.1, 2.7)	5.37 (dd, 9.1, 2.7)
14	2.56 (m)	2.39 (m)	2.38 (m)	2.38 (m)	2.38 (m)
	2.01 (overlapped)	1.95 (overlapped)	1.87 (overlapped)	2.00 (overlapped)	1.95 (m)
15	2.85 (dd, 11.6, 1.8)	2.63 (overlapped)	2.55 (dd, 11.2, 1.8)	2.62 (overlapped)	2.63 (overlapped)
19	1.12 (s)	0.94 (s)	0.98 (s)	0.95 (s)	0.93 (s)
20	1.30 (s)	1.67 (s)	1.67 (s)	1.65 (s)	1.63 (s)
21	1.68 (s)	5.18 (d, 1.1)	1.36 (s)	5.19 (d, 1.1)	5.07 (d, 1.6)
		5.14 (d, 1.1)		5.04 (d, 1.1)	5.02 (d, 1.6)
22	1.62 (s)	1.63 (s)	1.59 (s)	1.61 (s)	1.61 (s)
23	2.64 (m)	2.61 (overlapped)	2.61 (m)	2.62 (overlapped)	2.63 (overlapped)
24	3.84 (dd, 10.1, 7.2)	3.82 (dd, 10.4, 7.1)	3.83 (dd, 10.4, 7.1)	3.83 (dd, 10.4, 7.0)	3.80 (dd, 9.5, 7.2)
	3.72 (dd, 10.1, 6.3)	3.69 (dd, 10.4, 6.5)	3.70 (dd, 10.4, 6.4)	3.70 (dd, 10.4, 6.4)	3.72 (dd, 9.5, 5.9)
25	1.28 (d, 7.1)	1.28 (d, 7.1)	1.26 (d, 7.1)	1.27 (d, 7.1)	1.27 (d, 7.1)

^a^ In methanol-*d*_4_. ^b^ Recorded at 800 MHz. ^c^ Recorded at 500 MHz.

**Table 2:** ^13^C NMR data of compounds **1**–**5**.

No.	1 ^a,b^	2 ^a,b^	3 ^a,c^	4 ^a,c^	5 ^a,b^
	*δ*_C_, Type	*δ*_C_, Type	*δ*_C_, Type	*δ*_C_, Type	*δ*_C_, Type
1	47.9 C	49.8 C	49.9 C	49.8 C	50.1 C
2	40.9 CH_2_	40.2 CH_2_	40.2 CH_2_	40.0 CH_2_	39.5 CH_2_
3	63.8 CH	122.1 CH	121.3 CH	121.7 CH	121.5 CH
4	63.1 C	138.7 C	138.5 C	139.0 C	139.1 C
5	39.9 CH_2_	36.2 CH_2_	43.2 CH_2_	35.9 CH_2_	36.5 CH_2_
6	24.2 CH_2_	30.0 CH_2_	128.2 CH	34.5 CH_2_	35.6 CH_2_
7	125.7 CH	87.3 CH	135.0 CH	73.9 CH	77.3 CH
8	135.3 C	147.1 C	85.0 C	151.3 C	151.3 C
9	35.8 CH_2_	31.2 CH_2_	34.9 CH_2_	30.7 CH_2_	26.8 CH_2_
10	31.2 CH_2_	32.7 CH_2_	30.8 CH_2_	33.0 CH_2_	34.2 CH_2_
11	77.0 CH	77.3 CH	79.8 CH	77.3 CH	76.7 CH
12	137.9 C	138.5 C	139.2 C	138.6 C	138.6 C
13	130.2 CH	128.7 CH	127.1 CH	128.6 CH	128.6 CH
14	30.6 CH_2_	30.1 CH_2_	30.1 CH_2_	30.0 CH_2_	30.1 CH_2_
15	51.0 CH	51.4 CH	51.0 CH	51.3 CH	51.1 CH
16	151.9 C	152.2 C	152.1 C	152.2 C	151.8 C
17	149.5 C	149.0 C	149.0 C	149.1 C	149.6 C
18	209.2 C	210.1 C	210.3 C	210.2 C	210.6 C
19	18.0 CH_3_	17.1 CH_3_	16.5 CH_3_	17.2 CH_3_	17.1 CH_3_
20	17.1 CH_3_	16.4 CH_3_	17.9 CH_3_	16.7 CH_3_	16.9 CH_3_
21	14.9 CH_3_	112.9 CH_2_	24.5 CH_3_	110.6 CH_2_	113.7 CH_2_
22	10.8 CH_3_	11.3 CH_3_	11.9 CH_3_	11.4 CH_3_	11.5 CH_3_
23	38.9 CH	38.9 CH	38.8 CH	38.8 CH	38.8 CH
24	66.0 CH_2_	65.8 CH_2_	65.9 CH_2_	65.9 CH_2_	66.1 CH_2_
25	14.7 CH_3_	14.7 CH_3_	14.7 CH_3_	14.8 CH_3_	14.8 CH_3_

^a^ In methanol-*d*_4_. ^b^ Recorded at 200 MHz. ^c^ Recorded at 125 MHz.

**Table 3:** ^1^H NMR data of compounds **6**–**10**.

No.	6 ^a,b^	7 ^a,c^	8 ^a,c^	9 ^a,c^	10 ^a,c^
	*δ*_H_ (*J* in Hz)	*δ*_H_ (*J* in Hz)	*δ*_H_ (*J* in Hz)	*δ*_H_ (*J* in Hz)	*δ*_H_ (*J* in Hz)
2	2.08 (dd, 14.1, 6.4)	2.15 (m)	2.31 (m)	2.43 (t, 12.2)	2.29 (dd, 13.3, 9.7)
	1.95 (dd, 14.1, 8.6)	1.90 (overlapped)	1.72 (d, 15.6)	1.66 (overlapped)	1.87 (dd, 13.3, 6.6)
3	5.27 (m)	5.20 (t, 6.7)	5.11 (overlapped)	5.37 (dd, 10.9, 3.3)	5.32 (t, 8.0)
5	2.79 (m)	2.77 (m)	2.09 (overlapped)	2.28 (d, 13.6)	2.17 (overlapped)
			1.87 (m)	2.15 (m)	2.12 (overlapped)
6	5.76 (m)	5.71 (dt, 15.6, 6.8)	1.75 (m)	2.24 (m)	2.24 (m)
			1.66 (overlapped)		
7	5.49 (d, 15.5)	5.49 (d, 15.6)	3.84 (dd, 11.4, 3.4)	5.15 (t, 6.2)	5.09 (t, 5.7)
9	1.75 (m)	1.67 (overlapped)	5.11 (overlapped)	2.08 (m)	2.18 (overlapped)
	1.65 (overlapped)			1.97 (m)	2.11 (overlapped)
10	1.63 (overlapped)	1.67 (overlapped)	2.59 (overlapped)	1.72 (m)	1.78 (m)
	1.52 (m)	1.36 (m)	2.24 (m)	1.66 (overlapped)	1.31 (m)
11	3.89 (dd, 9.8, 2.6)	4.08 (m)	3.90 (dd, 11.4, 4.0)	4.24 (t, 6.4)	3.48 (d, 10.7)
13	5.21 (dd, 9.5, 1.6)	5.31 (dd, 8.5, 2.6)	5.46 (d, 10.0)	3.95 (d, 10.8)	5.63 (d, 15.6)
14	2.35 (m)	2.37 (m)	2.44 (m)	1.83 (dd, 12.0, 3.2)	5.28 (dd, 15.6, 10.3)
	1.88 (m)	1.88 (overlapped)	2.07 (overlapped)	1.63 (overlapped)	
15	2.51 (dd, 11.4, 2.0)	2.55 (d, 10.0)	2.65 (d, 11.2)	3.01 (d, 10.0)	3.33 (overlapped)
19	1.00 (s)	0.96 (s)	0.98 (s)	0.94 (s)	0.94 (s)
20	1.68 (s)	1.66 (s)	1.62 (s)	1.664 (s)	1.65 (overlapped)
21	1.27 (s)	1.26 (overlapped)	1.67 (overlapped)	1.660 (s)	1.65 (overlapped)
22	1.57 (s)	1.58 (s)	1.67 (overlapped)	5.25 (d, 3.5)	1.21 (s)
23	2.60 (m)	2.60 (m)	2.61 (overlapped)	2.67 (m)	2.71 (m)
24	3.83 (dd, 10.4, 7.2)	3.82 (m)	3.81 (m)	3.83 (dd, 10.1, 6.6)	3.77 (dd, 10.0, 7.3)
	3.69 (dd, 10.4, 6.4)	3.71 (m)	3.70 (m)	3.75 (dd, 10.1, 6.4)	3.66 (dd, 10.0, 6.3)
25	1.25 (d, 7.1)	1.26 (overlapped)	1.27 (d, 6.3)	1.29 (d, 7.0)	1.20 (d, 7.4)

^a^ In methanol-*d*_4_. ^b^ Recorded at 500 MHz. ^c^ Recorded at 800 MHz.

**Table 4:** ^13^C NMR data of compounds **6**–**10**.

No.	6 ^a,b^	7 ^a,c^	8 ^a,c^	9 ^a,c^	10 ^a,c^
	*δ*_C_, Type	*δ*_C_, Type	*δ*_C_, Type	*δ*_C_, Type	*δ*_C_, Type
1	49.8 C	49.5 C	49.0 C	49.8 C	50.1 C
2	39.9 CH_2_	40.4 CH_2_	40.3 CH_2_	40.2 CH_2_	39.3 CH_2_
3	120.9 CH	121.0 CH	123.7 CH	122.7 CH	121.8 CH
4	138.4 C	138.7 C	137.1 C	139.6 C	138.5 C
5	42.9 CH_2_	43.0 CH_2_	36.3 CH_2_	41.2 CH_2_	40.7 CH_2_
6	126.1 CH	127.1 CH	30.4 CH_2_	25.2 CH_2_	24.8 CH_2_
7	138.0 CH	138.3 CH	77.5 CH	124.1 CH	122.0 CH
8	74.3 C	74.4 C	138.6 C	135.7 C	135.2 C
9	39.0 CH_2_	39.4 CH_2_	125.3 CH	34.5 CH_2_	33.2 CH_2_
10	30.6 CH_2_	32.5 CH_2_	33.5 CH_2_	35.1 CH_2_	29.6 CH_2_
11	80.4 CH	79.3 CH	79.7 CH	72.5 CH	75.6 CH
12	139.1 C	139.5 C	137.3 C	157.2 C	76.5 C
13	127.4 CH	127.1 CH	130.8 CH	70.1 CH	140.5 CH
14	30.0 CH_2_	30.2 CH_2_	30.3 CH_2_	36.3 CH_2_	131.6 CH
15	50.9 CH	51.3 CH	51.7 CH	46.9 CH	54.6 CH
16	152.1 C	152.1 C	151.9 C	153.0 C	149.9 C
17	148.8 C	148.8 C	149.7 C	149.4 C	149.7 C
18	210.1 C	210.1 C	210.3 C	210.8 C	209.7 C
19	16.5 CH_3_	16.4 CH_3_	17.1 CH_3_	17.9 CH_3_	18.7 CH_3_
20	18.0 CH_3_	18.0 CH_3_	15.4 CH_3_	15.4 CH_3_	16.1 CH_3_
21	30.4 CH_3_	27.5 CH_3_	9.9 CH_3_	17.4 CH_3_	19.2 CH_3_
22	11.4 CH_3_	11.8 CH_3_	10.6 CH_3_	110.4 CH_2_	17.7 CH_2_
23	38.8 CH	38.9 CH	38.8 CH	38.6 CH	38.0 CH
24	65.9 CH_2_	65.9 CH_2_	65.9 CH_2_	65.9 CH_2_	66.1 CH_2_
25	14.6 CH_3_	14.7 CH_3_	14.7 CH_3_	14.9 CH_3_	14.7 CH_3_

^a^ In methanol-*d*_4_. ^b^ Recorded at 125 MHz. ^c^ Recorded at 200 MHz.

**Table 5:** NMR calculated results of compounds **1**, **4**, **6**, **8** and **10**.

Candidates	R^2^		MAE		CMAE		DP4+
	^13^C	^1^H	^13^C	^1^H	^13^C	^1^H	All Data
**1a**	0.9976	0.98	2.5	0.17	2.3	0.13	100%
**1b**	0.9956	0.9737	3.1	0.18	2.8	0.15	0
**4a**	0.9973	0.9686	2.8	0.19	2.4	0.18	0
**4b**	0.9972	0.9705	2.7	0.17	2.2	0.16	100%
**6a**	0.9974	0.9816	2.3	0.21	2.1	0.16	0
**6b**	0.998	0.9878	2.3	0.19	2.1	0.11	100%
**8a**	0.993	0.9696	4.2	0.21	3.8	0.17	0
**8b**	0.9945	0.9718	3.7	0.22	3.2	0.16	0
**8c**	0.9976	0.9911	2.5	0.13	2	0.09	100%
**8d**	0.9962	0.9842	3.1	0.19	2.8	0.13	0
**10a**	0.9987	0.9873	1.7	0.17	1.7	0.09	100%
**10b**	0.9983	0.9879	2	0.18	1.8	0.12	0

**Table 6:** Cytotoxicity (IC_50_ in µM) of terpestacin derivatives toward the SH-SY5Y cell line.

Compound	IC_50_ ± SD (µM)
**1**	31.42 ± 0.47
**2**	36.54 ± 0.89
**4**	35.74 ± 2.34
**6**	21.13 ± 1.06
**7**	30.84 ± 0.79
**9**	33.91 ± 2.07
**12**	32.41 ± 1.15
**13**	28.79 ± 1.35
**Doxorubicin**	0.553 ± 0.015
**Paclitaxel**	<0.016

## Data Availability

The datasets used and/or analyzed during the current study are available from the corresponding author on reasonable request.

## Supplementary Materials

The following supporting information can be downloaded at: https://www.mdpi.com/article/10.3390/jof12090693/s1, Section S1: Computational data of compound **1**; Section S2: Computational data of compound **4**; Section S3: Computational data of compound **6**; Section S4: Computational data of compound **8**; Section S5: Computational data of compound **10**; Section S6: NMR data of synthetic and natural **1**, **9**, **11**, **12**, **13**; Section S7: NMR data comparison of synthetic and natural **2**, **3**; Section S8: NMR data comparison of synthetic and natural **4**, **5**, **6**; Section S9: NMR, HRESIMS, CD, UV, OR, and IR spectra.

## Abbreviations

The following abbreviations are used in this manuscript.

HPLC	High-performance liquid chromatography
HRESIMS	High-resolution electrospray ionization mass spectrometry
NMR	Nuclear magnetic resonance
COSY	Correlation spectroscopy
HSQC	Heteronuclear single quantum coherence spectroscopy
HMBC	Heteronuclear multiple bond correlation spectroscopy
ROESY	Rotating frame Overhauser effect spectroscopy
UV	Ultraviolet spectroscopy
ECD	Electronic circular dichroism
IR	Infrared spectroscopy
TLC	Thin-layer chromatography
ITS	Internal transcribed spacer
MeOH	Methanol
THF	Tetrahydrofuran
MeCN	Acetonitrile
EtOAc	Ethyl acetate
CHCl_3_	Chloroform
PE	Petroleum ether
Me_2_CO	Acetone
*m*-CPBA	*m*-Chloroperbenzoic Acid
SD	Standard deviation
