# Supplementary material for: Discovery and Semisynthesis of Arthritacins A–J—Terpestacin-Derived Sesterterpenoids from the Plant Endophytic Fungus Arthrinium sp. HS66
Source: J Fungi (Basel). 2026 Sep 16;12(9):693. doi: 10.3390/jof12090693 (PMC13608384; doi:10.3390/jof12090693)
Supplement: Supplementary file 1 [file jof-12-00693-s001.zip › jof-4542356-supplementary.pdf]

Supplementary Materials for

Discovery and Semisynthesis of Arthritacins A–J — Terpestacin-derived Sesterterpenoids from the Plant Endophytic Fungus *Arthrinium* sp. HS66

Xiao-Zheng Su [1,2], Kun Hu [1,2], Xing-Ren Li [1,2], Han-Dong Sun [1,2] and Pema-Tenzin Puno [1,2,*]

[1]State Key Laboratory of Phytochemistry and Natural Medicines, Kunming Institute of Botany, Chinese Academy of Sciences, Kunming 650201, China; suxiaozheng@sjtu.edu.cn (X.-Z.S.); hukun@mail.kib.ac.cn (K.H.); lixingren@mail.kib.ac.cn (X.-R.L.); hdsun@mail.kib.ac.cn (H.-D.S.)

[2]Yunnan Key Laboratory of Natural Medicinal Chemistry, Kunming 650201, China

[*]Corresponding author Tel.: (86) 871-65223616
E-mail: punopematenzin@mail.kib.ac.cn.

Contents of Supporting Information

Contents	Pages
S1. Computational data of compound **1**	3–13
S2. Computational data of compound **4**	13–23
S3. Computational data of compound **6**	24–36
S4. Computational data of compound **8**	37–57
S5. Computational data of compound **10**	58–69
S6. NMR data of synthetic and natural **1**, **9**, **11**, **12**, **13**	70–74
S7. NMR data comparison of synthetic and natural **2**, **3**	75–76
S8. NMR data comparison of synthetic and natural **4**, **5**, **6**	77–79
S9. NMR, HRESIMS, CD, UV, OR, and IR spectra	80–237

Experimental details

S1. *Computational data of compound 1*

Table S1. Experimental and calculated NMR data of **1a** and **1b**.

No.	**1a**		**1b**		**1**	
	$\delta_{calcd.\ H}$	$\delta_{calcd.\ C}$	$\delta_{calcd.\ H}$	$\delta_{calcd.\ C}$	$\delta_{exptl.\ H}$	$\delta_{exptl.\ C}$
1		52.8		50.9		47.9
2	1.55	38.1	1.73	38.8	1.64	40.9
	1.27		1.16		1.54	
3	2.5	62.7	2.83	61.5	2.98	63.8
4		61.6		64.4		63.1
5	1.68	40.1	1.48	40.1	2.11	39.9
	1.05		1.26		1.21	
6	2.31	27.1	2.22	27.0	2.33	24.2
	2.21		2.09		2.05	
7	5.7	124.3	5.51	122.8	5.26	125.7
8		137.2		139.6		135.3
9	2.01	34.4	2.09	36.4	2.11	35.8
	1.93		2.04		1.82	
10	1.92	38.0	1.72	39.3	1.83	31.2
	1.58		1.55		1.68	
11	3.79	77.7	3.95	77.2	3.93	77.0
12		139.3		142.9		137.9
13	5.71	126.1	6.26	120.3	5.46	130.2
14	2.35	30.4	2.65	28.6	2.56	30.6
	2.18		1.80		2.01	
15	2.73	54.0	2.76	54.8	2.85	51.0
16		152.9		153.2		151.9
17		142.8		143.4		149.4
18		203.8		203.8		209.2
19	1.00	20.6	1.11	19.7	1.12	18.0
20	1.12	16.6	1.04	17.3	1.30	17.1
21	1.73	20.1	1.63	18.3	1.68	14.9
22	1.51	13.1	1.75	17.7	1.62	10.8
23	2.72	41.5	2.77	41.5	2.64	38.9
24	3.93	67.0	4.01	66.8	3.84	66.0
	3.66		3.54		3.72	
25	1.12	14.8	1.15	14.7	1.28	14.7

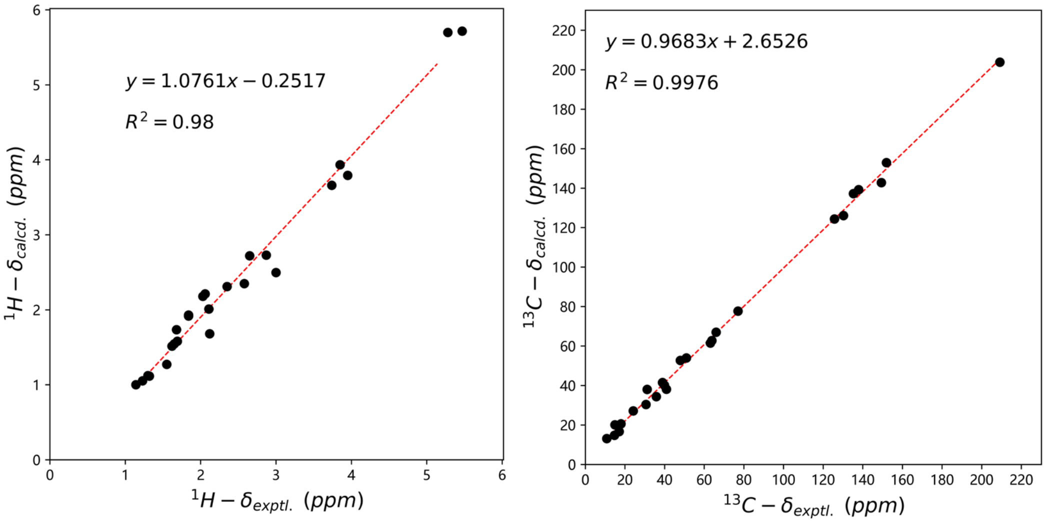

Figure S1. Linear regression analyses between experimental and calculated NMR data of **1a**.

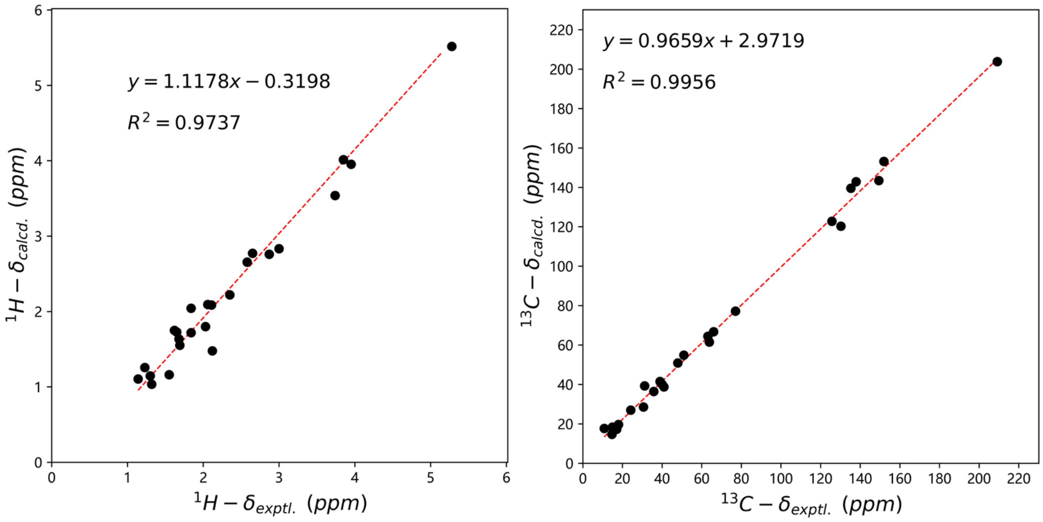

Figure S2. Linear regression analyses between experimental and calculated NMR data of **1b**.

Functional	Solvent?		Basis Set		Type of Data	
mPW1PW91	PCM		6-31+G(d, p)		Unscaled Shifts	
	Isomer 1	Isomer 2	Isomer 3	Isomer 4	Isomer 5	Isomer 6
sDP4+ (H data)	99.33%	0.67%	–	–	–	–
sDP4+ (C data)	100.00%	0.00%	–	–	–	–
sDP4+ (all data)	100.00%	0.00%	–	–	–	–
uDP4+ (H data)	90.19%	9.81%	–	–	–	–
uDP4+ (C data)	99.98%	0.02%	–	–	–	–
uDP4+ (all data)	100.00%	0.00%	–	–	–	–
DP4+ (H data)	99.93%	0.07%	–	–	–	–
DP4+ (C data)	100.00%	0.00%	–	–	–	–
DP4+ (all data)	100.00%	0.00%	–	–	–	–

Figure S3. DP4+ analysis results of **1a** (left) and **1b** (right).

Table S2. Conformational analysis of the B3LYP-D3BJ/6-31G(d) optimized conformers of **1a** in the gas phase (T=298.15 K).

Conformer	E (Hartree)[a]	C (Hartree)[b]	G (kcal/mol)[c]	ΔG (kcal/mol)[d]	Population[e]
**1a-1**	-1351.445161	0.523083	-847703.603945	0.0	55.90%
**1a-2**	-1351.447259	0.526351	-847702.869952	0.733993	16.18%
**1a-3**	-1351.448997	0.52862	-847702.536536	1.067409	9.21%
**1a-4**	-1351.445313	0.525111	-847702.426956	1.176989	7.66%
**1a-5**	-1351.445677	0.525703	-847702.283528	1.320417	6.01%
**1a-6**	-1351.447182	0.527375	-847702.178773	1.425172	5.04%

[a]Electronic energy obtained at the M06-2X-D3/def2-TZVP level of theory; [b]Thermal correction to Gibbs free energy obtained at the B3LYP-D3BJ/6-31G(d) level of theory; [c]Gibbs free energy (E + C); [d]The relative Gibbs free energy; [e]The Boltzmann distribution of each conformer.

Table S3. Atomic coordinates (Å) of **1a-1** obtained at the B3LYP-D3BJ/6-31G(d) level of theory in the gas phase.

C	1.554277	-0.737842	-0.884993	H	-0.399433	-1.058457	1.276121
C	0.821172	-1.970887	-0.290920	H	-4.249717	-1.371855	-0.969977
C	-0.499091	-1.655095	0.367457	H	-5.424035	0.767888	-1.595476
C	-1.744180	-2.452157	0.278431	H	-5.424994	2.028792	-0.364983
C	-2.726549	-2.357884	1.437384	H	-2.950877	0.666838	-1.560009
C	-4.215972	-2.303388	1.020999	H	-3.529497	2.288492	-1.956024
C	-4.490924	-1.167132	0.073165	H	-2.615219	1.374645	0.810235
C	-4.869982	0.080600	0.377960	H	-0.507264	1.144067	1.168690
C	-4.887978	1.139516	-0.713500	H	1.766428	2.381433	0.815733
C	-3.463891	1.542480	-1.149434	H	1.548752	2.151167	-0.908393
C	-2.606459	2.099739	-0.006963	H	0.705189	-2.706293	-1.090999
C	-1.158295	2.321656	-0.410078	H	1.466658	-2.430227	0.471947
C	-0.193902	1.726556	0.302768	H	1.611284	-0.051209	1.199172
C	1.291019	1.731269	0.067465	H	5.232995	-1.425372	-1.653993
C	1.925209	0.325016	0.213515	H	3.505835	1.718727	1.876833
C	3.445115	0.383826	0.229024	H	5.936556	-0.074254	1.471688
C	3.964814	-0.468058	-0.680096	H	5.570504	0.958628	2.867902
C	2.919953	-1.214520	-1.375873	H	4.123240	-1.312499	2.061323
C	0.802225	-0.190191	-2.113225	H	0.724994	-0.984809	-2.861273
C	-1.838502	-3.697840	-0.573474	H	-0.198969	0.144947	-1.850340
C	-5.215927	0.565491	1.762259	H	1.349691	0.641730	-2.568165
C	-0.913413	3.196254	-1.612413	H	5.753594	1.857689	-0.215653
C	4.219360	1.232915	1.200846	H	5.607688	2.914520	1.205450
C	5.123093	0.344733	2.079807	H	4.395211	2.988077	-0.086660
C	5.044144	2.314193	0.481728	H	0.138246	3.466224	-1.728691
O	3.159858	-2.088241	-2.201454	H	-1.238424	2.706492	-2.539442
O	-3.198646	3.285081	0.554030	H	-1.480966	4.132340	-1.528773
O	5.263577	-0.730207	-0.964065	H	-3.445871	3.864010	-0.184218

O	-1.585721	-1.220052	-0.463293	H	-1.651435	-4.593545	0.030164
O	4.394211	-0.679306	2.744548	H	-2.841295	-3.787586	-1.004552
H	-2.497968	-1.451772	2.009661	H	-1.124368	-3.675399	-1.398498
H	-2.562991	-3.215456	2.105016	H	-6.269798	0.875028	1.806867
H	-4.817281	-2.217868	1.932392	H	-5.055494	-0.188862	2.536963
H	-4.502673	-3.255258	0.555864	H	-4.626435	1.455285	2.014948

Table S4. Atomic coordinates (Å) of **1a-2** obtained at the B3LYP-D3BJ/6-31G(d) level of theory in the gas phase.

C	-2.410649	0.021626	-1.209166	H	-0.462816	1.728244	-1.819049
C	-2.342123	1.473759	-0.656948	H	1.171555	1.089609	0.339278
C	-1.108519	2.246350	-1.105994	H	3.349260	-1.043692	2.042008
C	-0.471345	3.288898	-0.271918	H	2.012453	-0.931676	0.904789
C	0.998651	3.632241	-0.459913	H	4.240716	-0.393129	-0.737535
C	1.934364	3.130602	0.654699	H	4.858389	-1.627178	0.345712
C	2.042429	1.627055	0.711066	H	2.954280	-3.105827	-0.207374
C	3.081587	0.896199	1.150372	H	0.771921	-3.165890	-0.539739
C	3.044346	-0.614455	1.078112	H	-1.285691	-2.640126	-1.766231
C	3.936488	-1.190622	-0.051445	H	-0.490705	-1.331764	-2.634107
C	3.219189	-2.268955	-0.874634	H	-3.239227	2.016134	-0.973584
C	1.948451	-1.746654	-1.517122	H	-2.358206	1.422895	0.436458
C	0.768022	-2.310272	-1.215058	H	-0.364031	-0.072859	-0.421902
C	-0.579233	-1.809140	-1.654888	H	-4.763106	-1.649196	1.266709
C	-1.174151	-0.769023	-0.659421	H	0.265802	-1.897126	1.334215
C	-1.672277	-1.406936	0.626239	H	-1.795301	-0.773062	3.276805
C	-3.021554	-1.391191	0.655566	H	-0.115499	-1.177988	3.680675
C	-3.573227	-0.638314	-0.473272	H	0.512470	0.518847	2.273946
C	-2.644371	0.026012	-2.720446	H	-2.709827	-0.987290	-3.127108
C	-1.072695	3.649349	1.070972	H	-3.593537	0.528008	-2.930275
C	4.357843	1.480728	1.696034	H	-1.850991	0.558929	-3.254497
C	2.120392	-0.558006	-2.429221	H	-0.407973	-3.571004	3.044026
C	-0.756936	-1.872374	1.724689	H	-1.034643	-4.015338	1.445615
C	-0.781108	-0.838507	2.872112	H	-2.120661	-3.306472	2.647013
C	-1.102673	-3.274794	2.249543	H	2.202998	0.371926	-1.851555
O	-4.774155	-0.510746	-0.676933	H	3.044342	-0.663587	-3.006685
O	4.168728	-2.704500	-1.853348	H	1.288093	-0.440209	-3.127794
O	-3.869468	-1.890740	1.586941	H	3.720166	-3.359953	-2.410732
O	-1.279060	3.636257	-1.419842	H	-0.803990	2.912281	1.835225
O	-0.442174	0.477028	2.448263	H	-0.722818	4.636393	1.395433
H	1.071085	4.726803	-0.511787	H	-2.161814	3.686380	0.999769
H	1.333742	3.249995	-1.431727	H	5.225489	1.104420	1.138346

H	1.577020	3.519575	1.618867	H	4.379620	2.571963	1.663361
H	2.922618	3.578382	0.502548	H	4.502429	1.172728	2.740491

Table S5. Atomic coordinates (Å) of **1a-3** obtained at the B3LYP-D3BJ/6-31G(d) level of theory in the gas phase.

C	1.587537	-1.195674	-1.232116	H	0.080592	-2.022182	1.005843
C	0.235681	-1.939515	-1.192299	H	-3.233191	0.132899	2.396246
C	-0.521336	-1.851863	0.109829	H	-5.004405	2.370519	0.060712
C	-1.977333	-2.085527	0.243749	H	-4.714525	2.341869	1.789927
C	-2.553949	-2.481133	1.591713	H	-2.796449	3.387484	0.952702
C	-3.948127	-1.861313	1.861241	H	-2.249982	1.738742	1.231379
C	-3.939762	-0.362132	1.728462	H	-2.956204	3.049010	-1.431363
C	-4.609358	0.407962	0.859369	H	-0.649485	0.758009	-2.046817
C	-4.374827	1.916695	0.835674	H	1.756714	2.333219	-0.918738
C	-2.914500	2.352157	0.611848	H	1.835741	1.370186	-2.379036
C	-2.442069	2.262242	-0.859594	H	-0.398049	-1.564397	-1.998388
C	-0.930611	2.478001	-0.928190	H	0.448407	-2.994882	-1.407910
C	-0.150046	1.572341	-1.531201	H	0.922519	0.225158	0.274487
C	1.341851	1.447428	-1.404023	H	4.619768	-2.491248	0.659337
C	1.668839	0.207123	-0.531245	H	2.748704	2.141687	0.975080
C	3.031752	0.218639	0.167937	H	4.384783	2.247856	2.623386
C	3.526969	-1.036362	0.205287	H	3.647795	0.646204	2.793230
C	2.665682	-1.981624	-0.492142	H	5.539138	-0.170387	1.770031
C	2.057119	-1.142953	-2.704102	H	1.329609	-0.610757	-3.324434
C	-2.821435	-2.443495	-0.960961	H	3.025503	-0.641665	-2.796174
C	-5.624981	-0.086838	-0.135746	H	2.161429	-2.161285	-3.091763
C	-0.412730	3.705569	-0.217296	H	4.876561	3.139960	0.212739
C	3.598617	1.471960	0.779820	H	4.073848	2.363274	-1.169142
C	4.293034	1.264650	2.148690	H	5.443167	1.566512	-0.362501
C	4.553082	2.179255	-0.203177	H	-0.404052	3.578618	0.873017
O	2.852719	-3.194124	-0.488551	H	0.600772	3.968755	-0.529884
O	-2.845363	1.043117	-1.462130	H	-1.054383	4.570985	-0.427091
O	4.638890	-1.525131	0.821822	H	-2.418406	0.338611	-0.935173
O	-1.430215	-0.738249	0.252619	H	-2.359408	-2.131094	-1.898241
O	5.605810	0.751163	2.075473	H	-2.975620	-3.528160	-0.996737
H	-1.854860	-2.156072	2.371308	H	-3.800071	-1.963404	-0.900110
H	-2.621562	-3.575512	1.653484	H	-6.583390	0.430703	0.006974
H	-4.241380	-2.147698	2.881399	H	-5.280666	0.146818	-1.150244
H	-4.688149	-2.315911	1.196058	H	-5.820145	-1.160192	-0.066609

Table S6. Atomic coordinates (Å) of **1a-4** obtained at the B3LYP-D3BJ/6-31G(d) level of theory in the gas phase.

C	-1.471401	0.889790	-0.737482	H	0.540097	1.029967	1.400531

C	-0.683017	2.060190	-0.090083	H	4.354000	1.343786	-0.917121
C	0.639466	1.667672	0.520035	H	5.446525	-0.797074	-1.678538
C	1.908312	2.427911	0.442016	H	5.430692	-2.119466	-0.514624
C	2.911511	2.244225	1.572094	H	2.980535	-0.620009	-1.588282
C	4.389593	2.168873	1.120202	H	3.495399	-2.239526	-2.071213
C	4.609895	1.076904	0.108297	H	2.664928	-1.424268	0.751728
C	4.955383	-0.196115	0.339397	H	0.570739	-1.147246	1.163854
C	4.916408	-1.196630	-0.805252	H	-1.740430	-2.310905	0.799484
C	3.471152	-1.530625	-1.229718	H	-1.551242	-2.001947	-0.915075
C	2.617117	-2.111287	-0.096378	H	-0.558438	2.834641	-0.851008
C	1.155347	-2.269216	-0.479997	H	-1.297568	2.497215	0.709955
C	0.223334	-1.679148	0.278688	H	-1.531184	0.090326	1.300700
C	-1.265457	-1.635385	0.074238	H	-5.109125	1.783322	-1.402846
C	-1.865880	-0.224958	0.299976	H	-3.444441	-1.561946	1.975450
C	-3.391752	-0.245769	0.329490	H	-5.548558	-1.143047	2.873893
C	-3.882075	0.684064	-0.514060	H	-4.881497	0.449705	2.478521
C	-2.825581	1.443738	-1.168750	H	-6.279178	0.355023	0.601038
C	-0.765736	0.386510	-2.011614	H	0.227109	0.000247	-1.790304
C	2.025645	3.711259	-0.348837	H	-1.353802	-0.397035	-2.500535
C	5.313643	-0.763691	1.688623	H	-0.672940	1.219498	-2.714764
C	0.860651	-3.079845	-1.715545	H	-3.935596	-2.914241	-0.090038
C	-4.156936	-1.180096	1.230698	H	-5.468347	-2.034169	-0.277480
C	-5.278740	-0.481990	2.042800	H	-5.218782	-3.076901	1.128646
C	-4.724758	-2.377744	0.445051	H	1.180705	-2.556788	-2.625921
O	-3.052310	2.380694	-1.926940	H	1.402576	-4.034186	-1.687274
O	3.179885	-3.339126	0.399812	H	-0.200384	-3.314976	-1.821326
O	-5.171726	1.025789	-0.782902	H	3.392837	-3.892962	-0.367771
O	1.692978	1.240223	-0.355940	H	1.293938	3.753325	-1.157478
O	-6.476002	-0.258999	1.330213	H	1.880986	4.580859	0.302681
H	2.667781	1.316084	2.101279	H	3.021466	3.790600	-0.797621
H	2.787808	3.069882	2.286934	H	5.196200	-0.045818	2.504596
H	5.007051	2.017765	2.012062	H	4.699042	-1.645191	1.908392
H	4.694962	3.135381	0.699352	H	6.356788	-1.110393	1.692848

Table S7. Atomic coordinates (Å) of **1a-5** obtained at the B3LYP-D3BJ/6-31G(d) level of theory in the gas phase.

C	1.869759	-1.268534	-1.207454	H	0.370648	-1.832683	1.087997
C	0.556991	-2.074874	-1.095753	H	-3.027927	0.247612	2.273585
C	-0.226932	-1.846113	0.173874	H	-4.852381	2.183220	-0.273852
C	-1.667021	-2.149280	0.336179	H	-4.567819	2.323243	1.451382
C	-2.224057	-2.389427	1.728548	H	-2.675029	3.345112	0.537139

C	-3.648568	-1.815867	1.925805	H	-2.083056	1.736394	0.941256
C	-3.703716	-0.338275	1.648597	H	-2.828826	2.822344	-1.809567
C	-4.391139	0.316409	0.702808	H	-0.487115	0.565686	-2.314214
C	-4.211282	1.823959	0.540238	H	1.925776	2.260248	-1.387822
C	-2.764182	2.283131	0.278506	H	1.974422	1.091576	-2.685421
C	-2.293714	2.092662	-1.183536	H	-0.077308	-1.838897	-1.953693
C	-0.787242	2.334187	-1.280602	H	0.823667	-3.137564	-1.166061
C	0.003730	1.417114	-1.851804	H	1.014629	0.273491	0.061112
C	1.497430	1.313453	-1.725719	H	4.832682	-2.095608	1.050158
C	1.817393	0.207564	-0.681458	H	2.919237	2.498265	0.315895
C	3.096799	0.386023	0.126632	H	3.381588	0.820033	2.817283
C	3.689639	-0.810140	0.331426	H	3.130602	2.577408	2.771132
C	2.961609	-1.884698	-0.333318	H	1.026113	2.056460	1.869503
C	2.384115	-1.383116	-2.658248	H	1.647292	-0.994558	-3.368225
C	-2.477728	-2.725467	-0.804914	H	3.320796	-0.833001	-2.793089
C	-5.368836	-0.313495	-0.252868	H	2.570050	-2.435250	-2.896722
C	-0.280028	3.599553	-0.630932	H	5.509319	1.204798	1.327084
C	3.438129	1.673624	0.822708	H	5.133250	2.942204	1.330196
C	2.890396	1.627540	2.266973	H	5.317778	2.070828	-0.203009
C	4.941808	1.990391	0.821762	H	-0.287714	3.535259	0.466720
O	3.230264	-3.073457	-0.192652	H	0.736761	3.848472	-0.944344
O	-2.670558	0.823760	-1.691875	H	-0.920149	4.453009	-0.888987
O	4.752646	-1.121800	1.114412	H	-2.220768	0.166852	-1.122555
O	-1.208126	-0.784544	0.145183	H	-2.588717	-3.808263	-0.675691
O	1.497712	1.347069	2.334319	H	-3.476261	-2.282450	-0.825521
H	-1.544387	-1.927101	2.453881	H	-2.013666	-2.540932	-1.774765
H	-2.231610	-3.468383	1.933639	H	-5.021007	-0.158589	-1.281034
H	-3.939521	-2.013484	2.967523	H	-5.514617	-1.383909	-0.085780
H	-4.360105	-2.367020	1.303634	H	-6.352256	0.169011	-0.168548

Table S8. Atomic coordinates (Å) of **1a-6** obtained at the B3LYP-D3BJ/6-31G(d) level of theory in the gas phase.

C	-1.706762	-1.271813	0.959118	H	0.003989	-2.089575	-1.130186
C	-0.345513	-1.993241	1.044756	H	3.384512	0.134805	-2.232977
C	0.521502	-1.901111	-0.186377	H	4.899049	2.411124	0.242890
C	1.988217	-2.104613	-0.190232	H	4.759554	2.373498	-1.504646
C	2.690355	-2.494454	-1.478867	H	2.760263	3.387505	-0.835421
C	4.088929	-1.843986	-1.628813	H	2.267172	1.730250	-1.160508
C	4.037985	-0.344851	-1.502833	H	2.717200	3.044008	1.555610
C	4.611220	0.439885	-0.579523	H	0.389808	0.703778	1.918119
C	4.346890	1.943867	-0.581610	H	-1.913999	2.247409	0.563063

C	2.865242	2.353679	-0.485577	H	-2.133329	1.299901	2.013734
C	2.266050	2.252869	0.938444	H	0.209802	-1.604025	1.900541
C	0.752217	2.450251	0.868831	H	-0.560362	-3.050526	1.249041
C	-0.070346	1.521677	1.372729	H	-0.937320	0.136024	-0.519097
C	-1.539681	1.373941	1.096809	H	-4.678268	-2.609027	-1.061219
C	-1.755931	0.114777	0.211847	H	-4.384064	0.716472	-2.058057
C	-3.057039	0.061210	-0.581441	H	-4.026284	2.626550	0.293706
C	-3.552788	-1.191923	-0.589713	H	-5.212108	2.714765	-1.017448
C	-2.722283	-2.106946	0.179389	H	-5.856141	0.534806	-0.138912
C	-2.293519	-1.180610	2.387597	H	-1.625796	-0.618807	3.047831
C	2.729201	-2.438375	1.087117	H	-3.272584	-0.691628	2.381156
C	5.542046	-0.033141	0.505130	H	-2.414094	-2.188702	2.796969
C	0.289723	3.690307	0.141293	H	-2.166272	1.479500	-2.834470
C	-3.712125	1.189712	-1.327480	H	-2.026362	2.596595	-1.472253
C	-4.622650	2.021332	-0.394960	H	-3.293184	2.836619	-2.682060
C	-2.739092	2.077609	-2.118557	H	0.898333	4.556133	0.432609
O	-2.882312	-3.322299	0.211398	H	0.385166	3.592739	-0.947898
O	2.629674	1.036658	1.570343	H	-0.751563	3.935894	0.364857
O	-4.658679	-1.646807	-1.234516	H	2.264766	0.328091	1.003315
O	1.416945	-0.769258	-0.259240	H	2.897262	-3.520004	1.145957
O	-5.455791	1.217562	0.423655	H	3.701304	-1.942112	1.108962
H	2.055803	-2.189672	-2.319479	H	2.180821	-2.127141	1.976935
H	2.788212	-3.587252	-1.526767	H	6.498327	0.504659	0.449585
H	4.475970	-2.125820	-2.618556	H	5.101335	0.193231	1.483339
H	4.778064	-2.281133	-0.900258	H	5.765221	-1.102094	0.456414

Table S9. Conformational analysis of the B3LYP-D3BJ/6-31G(d) optimized conformers of **1b** in the gas phase (T=298.15 K).

Conformer	E (Hartree)[a]	C (Hartree)[b]	G (kcal/mol)[c]	ΔG (kcal/mol)[d]	Population[e]
**1b-1**	-1351.449532	0.525835	-847704.619585	0.0	43.65%
**1b-2**	-1351.450661	0.527027	-847704.58014	0.039445	40.84%
**1b-3**	-1351.448288	0.525568	-847704.007089	0.612496	15.51%

[a]Electronic energy obtained at the M06-2X-D3/def2-TZVP level of theory; [b]Thermal correction to Gibbs free energy obtained at the M06-2X-D3/def2-SVP level of theory; [c]Gibbs free energy (E + C); [d]The relative Gibbs free energy; [e]The Boltzmann distribution of each conformer.

Table S10. Atomic coordinates (Å) of **1b-1** obtained at the B3LYP-D3BJ/6-31G(d) level of theory in the gas phase.

C	1.711168	-1.177670	-1.099233	H	-1.034979	-2.070437	-1.472769
C	0.806438	-2.141508	-0.284149	H	-3.521734	-0.192417	2.012016
C	-0.680092	-1.944919	-0.448593	H	-5.086179	2.420681	-0.077683
C	-1.693484	-2.175870	0.605506	H	-4.896406	2.142163	1.644145

C	-3.093757	-2.581684	0.172300	H	-2.919967	3.264040	0.961071
C	-4.232052	-2.054326	1.077924	H	-2.433147	1.594058	1.219919
C	-4.191854	-0.556414	1.232997	H	-2.935609	2.977124	-1.453540
C	-4.786614	0.353794	0.450317	H	-0.625246	0.749429	-1.992568
C	-4.516532	1.840220	0.658758	H	1.771615	2.367224	-0.906799
C	-3.032867	2.245078	0.572280	H	1.824232	1.353638	-2.334489
C	-2.451071	2.189049	-0.857480	H	1.043807	-3.165941	-0.600118
C	-0.941276	2.425868	-0.836846	H	1.083767	-2.061285	0.771317
C	-0.135824	1.549865	-1.448953	H	1.090971	0.281659	0.412391
C	1.356902	1.456616	-1.348082	H	5.401691	-1.830050	-0.318075
C	1.770616	0.254539	-0.449342	H	2.758435	2.236422	1.040312
C	3.189730	0.402474	0.070546	H	5.049057	0.578825	2.181074
C	3.935414	-0.679488	-0.241225	H	4.293839	2.017448	2.895573
C	3.143710	-1.691509	-0.930716	H	3.045677	-0.468881	2.487791
C	1.383638	-1.241879	-2.600741	H	2.093779	-0.651014	-3.186280
C	-1.302801	-2.557015	2.015827	H	1.464984	-2.279834	-2.938708
C	-5.715692	0.020946	-0.685727	H	0.379554	-0.877580	-2.825239
C	-0.469624	3.635184	-0.068773	H	5.606129	1.769505	-0.023428
C	3.630633	1.591085	0.880784	H	5.038440	3.252528	0.774074
C	4.106193	1.135430	2.275130	H	4.371090	2.775354	-0.798496
C	4.728443	2.396000	0.164150	H	-0.529287	3.479403	1.016090
O	3.590866	-2.781975	-1.268647	H	0.563060	3.901310	-0.308138
O	-2.793424	0.973074	-1.504687	H	-1.095160	4.508290	-0.296454
O	5.236169	-0.934639	0.042768	H	-2.452446	0.261716	-0.924228
O	-1.308436	-0.827007	0.219127	H	-1.994491	-2.107734	2.734811
O	3.122558	0.373475	2.962925	H	-1.345160	-3.644913	2.142621
H	-3.135973	-3.679389	0.132752	H	-0.298439	-2.213858	2.267420
H	-3.258231	-2.215310	-0.847612	H	-5.922976	-1.048414	-0.777451
H	-4.166036	-2.533553	2.062743	H	-6.675726	0.541298	-0.566494
H	-5.181719	-2.385771	0.645183	H	-5.269141	0.367680	-1.624571

Table S11. Atomic coordinates (Å) of **1b-2** obtained at the B3LYP-D3BJ/6-31G(d) level of theory in the gas phase.

C	1.074995	-0.344092	1.622123	H	-0.652195	0.775853	-0.551769
C	-0.129202	0.629289	1.566662	H	-2.570376	1.197807	-2.166419
C	-0.234424	1.369101	0.256799	H	-5.033297	-0.686865	-0.998756
C	-0.344643	2.823711	0.064529	H	-3.430850	-0.767894	-1.699816
C	-1.039892	3.309480	-1.197295	H	-2.831090	-0.989302	1.030545
C	-2.575736	3.134491	-1.158678	H	-4.419871	-1.736184	0.976290
C	-2.981425	1.690386	-1.282517	H	-3.465634	-3.159869	-0.992777
C	-3.737490	0.943693	-0.464324	H	-0.844727	-3.214684	1.432709

C	-3.968419	-0.520162	-0.780151	H	1.330701	-3.061668	-0.653765
C	-3.550285	-1.476634	0.364007	H	1.540402	-3.143049	1.084770
C	-2.917931	-2.800994	-0.103552	H	-1.057175	0.061625	1.700363
C	-1.443938	-2.639063	-0.469975	H	-0.038901	1.318034	2.411271
C	-0.502802	-2.883020	0.455638	H	0.622425	-0.717330	-0.482492
C	0.977907	-2.637288	0.294355	H	4.467694	1.374354	1.330021
C	1.290873	-1.121426	0.279011	H	2.522457	-1.886493	-1.977231
C	2.683913	-0.746549	-0.192633	H	3.594250	0.957768	-2.180175
C	3.262393	0.121153	0.664793	H	3.153111	-0.119848	-3.519901
C	2.363464	0.469412	1.758491	H	1.453201	1.226803	-1.736305
C	0.958261	-1.210725	2.887733	H	0.030474	-1.788596	2.892614
C	-0.310315	3.783462	1.229162	H	1.801222	-1.902875	2.979089
C	-4.406690	1.453807	0.785957	H	0.967308	-0.559715	3.767564
C	-1.134072	-2.165773	-1.868812	H	4.909635	-1.737708	-2.682317
C	3.151617	-1.080520	-1.579849	H	4.782107	-2.428649	-1.054181
C	2.908637	0.155411	-2.486694	H	5.282280	-0.743845	-1.257156
C	4.620562	-1.522354	-1.646995	H	-0.120616	-2.435257	-2.178132
O	2.629518	1.302567	2.617792	H	-1.835831	-2.597335	-2.592665
O	-3.109832	-3.712578	0.976895	H	-1.205818	-1.075190	-1.967131
O	4.453239	0.760436	0.567047	H	-2.593900	-4.507999	0.768429
O	0.921285	2.135897	-0.172218	H	0.119952	4.739888	0.911798
O	1.561578	0.601064	-2.480039	H	-1.323677	3.976560	1.600350
H	-0.633664	2.749528	-2.048130	H	0.298966	3.393376	2.046595
H	-0.792335	4.365227	-1.362684	H	-4.075676	0.893882	1.669455
H	-2.996770	3.699581	-2.002457	H	-4.215804	2.513044	0.970663
H	-2.981827	3.593858	-0.251536	H	-5.494611	1.315312	0.720735

Table S12. Atomic coordinates (Å) of **1b-3** obtained at the B3LYP-D3BJ/6-31G(d) level of theory in the gas phase.

C	1.707367	-1.290471	-0.994023	H	-0.967772	-2.220724	-1.602095
C	0.706316	-2.165933	-0.191408	H	-4.699174	-0.387901	-1.005790
C	-0.748353	-2.006480	-0.555188	H	-4.893985	1.903870	-0.686888
C	-1.889283	-2.176094	0.373547	H	-4.970532	2.555507	0.950879
C	-3.205987	-2.673099	-0.207004	H	-2.893635	3.442989	0.446693
C	-4.481891	-2.047520	0.403701	H	-2.310708	1.841161	0.870312
C	-4.594971	-0.589185	0.059579	H	-2.802371	2.996403	-1.901047
C	-4.467403	0.471692	0.866806	H	-0.539307	0.617711	-2.192174
C	-4.410955	1.876124	0.294398	H	1.858444	2.250123	-1.130809
C	-2.951855	2.385501	0.165503	H	1.950399	1.100329	-2.448207
C	-2.358294	2.222222	-1.257832	H	0.975212	-3.216159	-0.366863
C	-0.843619	2.407735	-1.210395	H	0.855284	-1.972042	0.875211

C	-0.047337	1.449821	-1.700013	H	0.996716	0.308491	0.323704
C	1.433245	1.315350	-1.506113	H	5.297963	-1.930625	0.170027
C	1.744851	0.193322	-0.471812	H	2.653722	2.293738	0.901475
C	3.118252	0.366479	0.152923	H	4.777131	0.703620	2.404207
C	3.860431	-0.752663	0.009504	H	4.004172	2.220441	2.906564
C	3.104929	-1.812027	-0.648638	H	2.724100	-0.266317	2.617633
C	1.519955	-1.475190	-2.509829	H	0.563547	-1.083137	-2.861422
C	-1.677867	-2.436210	1.848081	H	2.314426	-0.973342	-3.069590
C	-4.290223	0.398632	2.361846	H	1.574188	-2.541646	-2.750656
C	-0.357463	3.667086	-0.536323	H	4.445229	2.620822	-0.817680
C	3.517329	1.618065	0.886309	H	5.572685	1.668259	0.161635
C	3.847394	1.287515	2.355947	H	4.977572	3.232070	0.759942
C	4.700331	2.327799	0.205735	H	0.690623	3.880560	-0.760970
O	3.550314	-2.938112	-0.840419	H	-0.946949	4.533070	-0.864704
O	-2.737510	0.993056	-1.850505	H	-0.458972	3.613259	0.555279
O	5.125013	-1.002024	0.429141	H	-2.428028	0.288339	-1.244798
O	-1.470540	-0.854711	-0.065374	H	-0.724781	-2.035971	2.196679
O	2.780955	0.616895	3.015018	H	-2.468104	-1.960319	2.434867
H	-3.236206	-3.763484	-0.074700	H	-1.704002	-3.512494	2.053573
H	-3.204334	-2.486187	-1.287753	H	-5.070724	0.982182	2.868484
H	-4.504272	-2.219067	1.484070	H	-4.332377	-0.620212	2.753380
H	-5.341180	-2.593997	-0.009275	H	-3.329253	0.835020	2.666117

S2. *Computational data of compound 4*

Table S13. Experimental and calculated NMR data of **4a** and **4b**.

No.	**4a**		**4b**		**4**	
	$\delta_{calcd. H}$	$\delta_{calcd. C}$	$\delta_{calcd. H}$	$\delta_{calcd. C}$	$\delta_{exptl. H}$	$\delta_{exptl. C}$
1		54.7		52.3		49.8
2	2.41	36.6	2.18	39.6	2.23	40.0
	2.34		2.04		1.80	
3	5.02	124.4	5.21	122.1	5.16	121.7
4		136.0		140.3		139.0
5	2.20	37.9	2.20	36.9	2.11	35.9
	1.92		1.83			
6	1.52	33.7	2.06	41.0	1.86	34.5
	1.32		0.83		1.70	
7	3.65	72.5	3.79	72.6	3.96	73.9
8		155.2		156.6		151.3
9	2.34	33.3	2.1	32.3	2.25	30.7

	1.99		1.44		2.02	
10	1.64	36.5	1.67	37.3	1.84	33.0
	1.33		1.58		1.64	
11	3.97	78.0	3.91	79.7	4.09	77.3
12		139.9		140.2		138.6
13	6.13	127.4	5.92	129.6	5.37	128.6
14	2.30	28.4	2.41	30.2	2.38	30.0
	2.21		1.86		2.00	
15	2.65	53.1	2.53	54.3	2.62	51.3
16		155.2		156.1		152.2
17		143.7		142.7		149.1
18		204.5		204.1		210.2
19	0.92	24.3	0.96	20.1	0.95	17.2
20	1.63	17.4	1.73	19.1	1.65	16.7
21	5.26	107.7	5.16	104.2	5.19	110.6
	5.16		5.14		5.04	
22	1.64	14.2	1.64	12.9	1.61	11.4
23	2.87	42.2	2.70	41.2	2.62	38.8
24	3.94	68.0	3.79	66.3	3.83	65.9
	3.47		3.68		3.70	
25	1.18	15.8	1.02	16.8	1.27	14.8

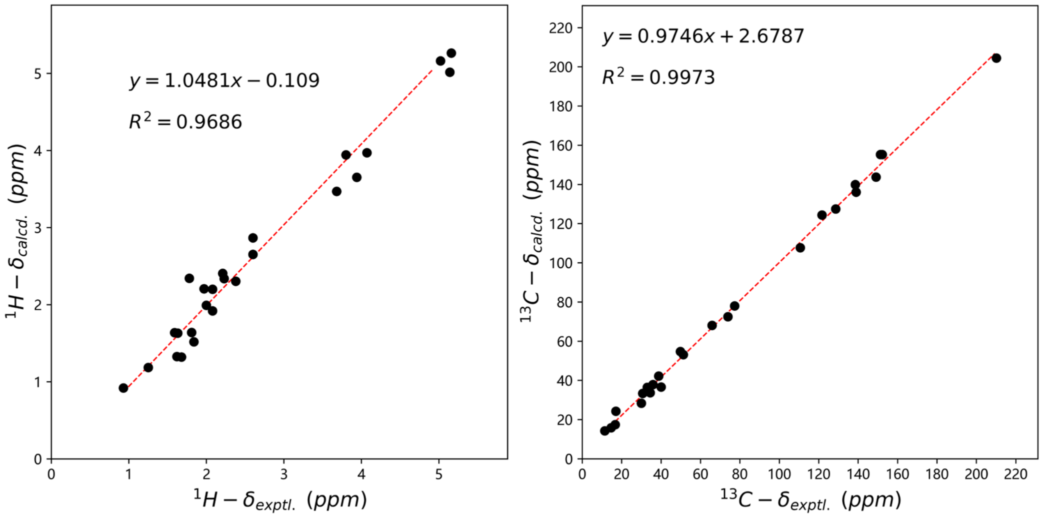

Figure S4. Linear regression analyses between experimental and calculated NMR data of **4a**.

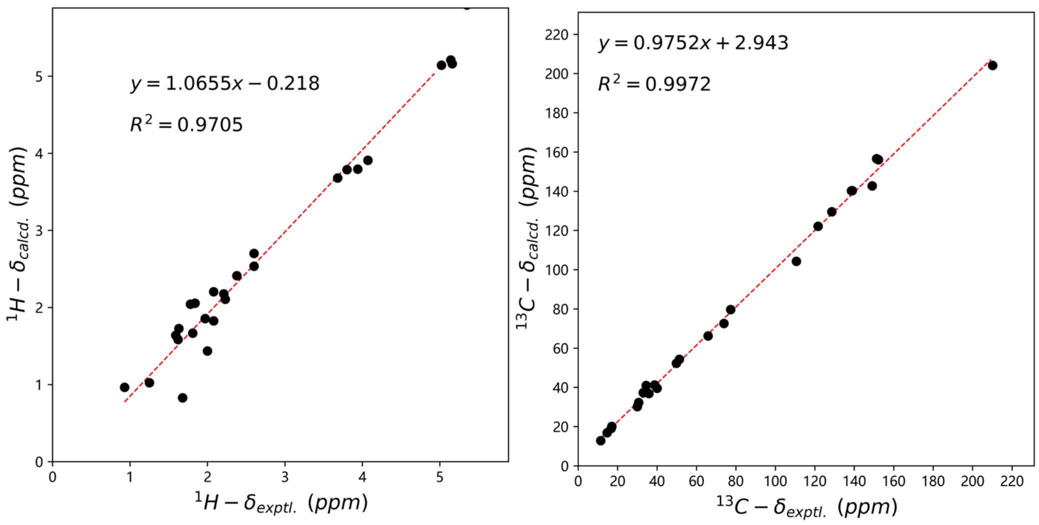

Figure S5. Linear regression analyses between experimental and calculated NMR data of **4b**.

Functional	Solvent?	Basis Set	Type of Data
mPW1PW91	PCM	6-31+G(d,p)	Unscaled Shifts

	Isomer 1	Isomer 2	Isomer 3	Isomer 4	Isomer 5	Isomer 6
sDP4+ (H data)	1.26%	98.74%	–	–	–	–
sDP4+ (C data)	2.97%	97.03%	–	–	–	–
sDP4+ (all data)	0.04%	99.96%	–	–	–	–
uDP4+ (H data)	0.04%	99.96%	–	–	–	–
uDP4+ (C data)	2.53%	97.47%	–	–	–	–
uDP4+ (all data)	0.00%	100.00%	–	–	–	–
DP4+ (H data)	0.00%	100.00%	–	–	–	–
DP4+ (C data)	0.08%	99.92%	–	–	–	–
DP4+ (all data)	0.00%	100.00%	–	–	–	–

Figure S6. DP4+ analysis results of **4a** (left) and **4b** (right).

Table S14. Conformational analysis of the B3LYP-D3BJ/6-31G(d) optimized conformers of **4a** in the gas phase (T=298.15 K).

Conformer	E (Hartree)[a]	C (Hartree)[b]	G (kcal/mol)[c]	ΔG (kcal/mol)[d]	Population[e]
**4a-1**	-1351.462534	0.526933	-847712.089408	0.0	55.76%
**4a-2**	-1351.464271	0.529394	-847711.635499	0.453908	25.91%
**4a-3**	-1351.460326	0.526349	-847711.070467	1.018941	9.98%
**4a-4**	-1351.455661	0.521851	-847710.965662	1.123746	8.36%

[a]Electronic energy obtained at the M06-2X-D3/def2-TZVP level of theory; [b]Thermal correction to Gibbs free energy obtained at the M06-2X-D3/def2-SVP level of theory; [c]Gibbs free energy (E + C); [d]The relative Gibbs free energy; [e]The Boltzmann distribution of each conformer.

Table S15. Atomic coordinates (Å) of **4a-1** obtained at the B3LYP-D3BJ/6-31G(d) level of theory in the gas phase.

C	2.219997	1.051664	1.187751	H	-3.294893	2.324123	-0.955048
C	1.357634	2.327112	1.278271	H	-2.873276	2.354317	-2.659357
C	0.530757	2.568871	0.044414	H	1.004923	2.271443	-0.890522
C	-0.692892	3.110303	-0.047325	H	-1.568089	0.630758	-0.502767
C	-1.356375	3.239782	-1.403008	H	-1.836779	-1.785359	-0.723474
C	-2.460821	2.192196	-1.655778	H	-3.361390	-2.401241	-1.333469
C	-1.953044	0.757500	-1.516827	H	-4.603600	-1.580914	0.479980
C	-2.970476	-0.338196	-1.794487	H	-3.606427	-0.158062	0.684484
C	-2.891621	-1.527387	-0.869775	H	-2.605134	-2.951858	1.378070
C	-3.564084	-1.236866	0.491709	H	-0.352312	-2.792864	1.458930
C	-2.847460	-1.915954	1.664764	H	0.857569	-0.427121	2.942871
C	-1.566526	-1.194945	2.034360	H	1.723691	-1.827931	2.341901
C	-0.372405	-1.777434	1.851510	H	2.049296	3.168403	1.428871
C	0.951473	-1.095156	2.081217	H	0.732042	2.270277	2.176118
C	1.411982	-0.250567	0.860600	H	0.496734	0.067286	0.344662
C	2.266754	-0.994607	-0.159077	H	4.594360	0.469465	-1.730590
C	3.200942	-0.157368	-0.664514	H	1.336223	-2.891512	0.097626
C	3.197746	1.131743	0.017536	H	2.063309	-1.965244	-2.704478
C	3.086263	0.918638	2.456564	H	1.416420	-3.563660	-2.294086
C	-1.495392	3.621113	1.122222	H	0.048392	-1.091486	-2.039131
C	-3.837176	-0.260834	-2.809195	H	2.470095	0.932342	3.361087
C	-1.731708	0.212471	2.560580	H	3.664331	-0.011106	2.446343
C	2.057134	-2.423834	-0.579486	H	3.787785	1.757061	2.508849
C	1.430635	-2.510601	-1.988999	H	4.113674	-2.813668	-1.211769
C	3.371948	-3.223161	-0.520446	H	3.191912	-4.271606	-0.783935
O	4.092844	-0.369227	-1.663488	H	3.800206	-3.193706	0.486971
O	0.089372	-2.068871	-2.026693	H	-1.553893	0.958979	1.778215
O	-3.777544	-1.916027	2.755281	H	-2.744638	0.356323	2.945330
O	3.926121	2.067313	-0.296272	H	-1.027650	0.431164	3.370399
O	-0.777990	0.549855	-2.347014	H	-3.312793	-2.284383	3.523491
H	-4.513103	-1.081444	-3.034707	H	-1.645125	4.705902	1.033624
H	-3.912319	0.611901	-3.452521	H	-2.496465	3.171248	1.149152
H	-1.800387	4.239357	-1.507716	H	-1.017281	3.430528	2.085644
H	-0.602148	3.137505	-2.188276	H	-1.105371	0.353524	-3.240769

Table S16. Atomic coordinates (Å) of **4a-2** obtained at the B3LYP-D3BJ/6-31G(d) level of theory in the gas phase.

C	1.984268	1.315425	1.062083	H	-3.251115	1.815353	-1.896983
C	1.108229	2.538820	0.703039	H	-2.635040	1.091517	-3.371066
C	0.515237	2.464696	-0.674113	H	1.166595	2.052682	-1.445536
C	-0.734128	2.798279	-1.024861	H	-1.484820	0.492709	-0.609678
C	-1.245982	2.476904	-2.409589	H	-5.094945	-0.384472	-0.966983
C	-2.349638	1.396892	-2.354356	H	-4.033765	0.814434	-0.256237
C	-1.900601	0.159926	-1.562883	H	-4.364681	-2.005331	0.861947
C	-3.014352	-0.813024	-1.251735	H	-5.305168	-0.605768	1.364872
C	-4.155648	-0.259657	-0.414003	H	-3.659616	-0.973525	2.974783
C	-4.332599	-0.916524	0.965423	H	-0.915798	0.732160	1.802241
C	-3.284477	-0.541441	2.028447	H	1.112992	-0.871648	2.634180
C	-1.893022	-1.121981	1.830846	H	0.561304	-1.912018	1.333549
C	-0.797895	-0.349281	1.782599	H	1.761816	3.418322	0.796477
C	0.609373	-0.873802	1.657810	H	0.321303	2.649344	1.455361
C	1.443761	-0.092848	0.625755	H	0.782576	0.068555	-0.234429
C	2.673508	-0.836478	0.086470	H	5.284310	0.785349	-0.693514
C	3.667067	0.052762	-0.148800	H	2.291350	-2.831983	0.678223
C	3.313906	1.388330	0.320979	H	1.903661	-2.062356	-2.232165
C	2.329937	1.380800	2.564131	H	1.924416	-3.732466	-1.640971
C	-1.744413	3.383289	-0.063213	H	-0.053240	-1.812772	-1.324843
C	-2.990831	-2.084916	-1.658614	H	1.422173	1.352811	3.175458
C	-1.814792	-2.634869	1.808724	H	2.973913	0.547448	2.862812
C	2.675032	-2.312816	-0.210867	H	2.860689	2.314440	2.775843
C	1.700865	-2.696149	-1.356364	H	4.787433	-2.614576	0.267540
C	4.071161	-2.878401	-0.514824	H	4.466031	-2.493824	-1.459297
O	4.882961	-0.108315	-0.727214	H	4.019372	-3.970822	-0.581043
O	0.335054	-2.646320	-0.989830	H	-1.156960	-3.011648	2.600369
O	-3.304555	0.881727	2.126642	H	-2.797197	-3.092227	1.959127
O	4.039814	2.366342	0.169848	H	-1.416695	-2.991103	0.852975
O	-0.779332	-0.445829	-2.229418	H	-2.683466	1.130101	2.828806
H	-3.803257	-2.766854	-1.420793	H	-1.269043	3.965425	0.730578
H	-2.147467	-2.514422	-2.188566	H	-2.436680	4.050643	-0.590576
H	-1.661036	3.372614	-2.892402	H	-2.352487	2.605412	0.420037
H	-0.421705	2.118511	-3.033698	H	-1.091095	-0.774471	-3.089125

Table S17. Atomic coordinates (Å) of **4a-3** obtained at the B3LYP-D3BJ/6-31G(d) level of theory in the gas phase.

C	2.204892	1.067717	1.185966	H	-3.275163	2.289423	-1.012728
C	1.351574	2.350798	1.255266	H	-2.839478	2.306195	-2.713961
C	0.533616	2.582142	0.013733	H	1.015276	2.277315	-0.914915
C	-0.691187	3.118483	-0.091928	H	-1.527331	0.630141	-0.524852
C	-1.346355	3.228706	-1.453574	H	-1.771216	-1.778583	-0.677615
C	-2.433196	2.162091	-1.704999	H	-3.264674	-2.438340	-1.316535
C	-1.904006	0.737388	-1.544188	H	-4.585421	-1.568392	0.403441
C	-2.900968	-0.378463	-1.816649	H	-3.593232	-0.148117	0.643400
C	-2.825170	-1.544068	-0.862180	H	-2.635246	-2.932030	1.453685
C	-3.541664	-1.230019	0.471014	H	-0.379496	-2.770585	1.521022
C	-2.872635	-1.883816	1.695758	H	0.821557	-0.374892	2.958707
C	-1.595363	-1.162816	2.064962	H	1.690867	-1.785207	2.386766
C	-0.401983	-1.745896	1.887270	H	2.048617	3.188933	1.398473
C	0.920151	-1.057210	2.108953	H	0.720690	2.309953	2.150193
C	1.388202	-0.233874	0.877281	H	0.476159	0.083007	0.355186
C	2.239976	-0.999119	-0.129041	H	4.588364	0.419455	-1.710889
C	3.183454	-0.177714	-0.642832	H	1.276009	-2.879193	0.135250
C	3.186894	1.122423	0.017962	H	2.127179	-2.032977	-2.657723
C	3.066483	0.947481	2.459377	H	1.439952	-3.610898	-2.233785
C	-1.504731	3.637529	1.066200	H	0.099854	-1.114481	-2.054911
C	-3.749980	-0.335701	-2.848179	H	2.447329	0.977482	3.361353
C	-1.758854	0.252852	2.568631	H	3.638546	0.014016	2.464292
C	2.027863	-2.435745	-0.523866	H	3.773173	1.782124	2.501912
C	1.458519	-2.550874	-1.954716	H	4.105095	-2.854841	-1.061111
C	3.332012	-3.244541	-0.392812	H	3.154592	-4.296964	-0.641996
O	4.080292	-0.414271	-1.632080	H	3.717867	-3.197244	0.630895
O	0.125892	-2.092243	-2.059523	H	-1.558001	0.988731	1.781266
O	-3.737786	-1.826234	2.835030	H	-2.774635	0.410586	2.937616
O	3.923253	2.047477	-0.308551	H	-1.067793	0.470683	3.389483
O	-0.718664	0.538023	-2.359924	H	-4.606600	-2.152999	2.554274
H	-4.408894	-1.171113	-3.069972	H	-1.032422	3.460367	2.034953
H	-3.824395	0.521877	-3.511713	H	-1.660375	4.720347	0.964362
H	-1.804415	4.220553	-1.570050	H	-2.503243	3.181745	1.091556
H	-0.585161	3.130912	-2.232657	H	-1.033110	0.334454	-3.256723

Table S18. Atomic coordinates (Å) of **4a-4** obtained at the B3LYP-D3BJ/6-31G(d) level of theory in the gas phase.

C	-1.422759	0.777013	-0.896563	H	3.740156	2.858506	-0.261884
C	-1.292603	2.184984	-0.224194	H	4.277684	2.664912	1.397892
C	-0.043667	2.377045	0.584518	H	-0.003876	1.823178	1.521966
C	1.014708	3.146395	0.286605	H	2.301732	0.794878	0.020488
C	2.191267	3.216501	1.236601	H	4.819459	-0.718561	-1.922935
C	3.436923	2.473012	0.718796	H	3.096960	-0.498082	-1.639847
C	3.207180	0.961597	0.606903	H	4.889902	-2.491314	-0.148511
C	4.363171	0.221769	-0.054189	H	3.889751	-2.901784	-1.534548
C	4.041063	-0.774150	-1.153298	H	2.865130	-1.840496	1.140545
C	3.960873	-2.233125	-0.669133	H	0.753762	-1.191581	1.224404
C	2.805209	-2.524737	0.285692	H	-1.204881	-2.026261	-1.032340
C	1.419899	-2.414016	-0.328892	H	-1.509668	-2.396970	0.648150
C	0.465548	-1.726709	0.318261	H	-2.164494	2.318625	0.432941
C	-1.001971	-1.679869	-0.015100	H	-1.381142	2.935952	-1.013930
C	-1.692017	-0.311834	0.196911	H	-1.387131	0.056462	1.185492
C	-3.201251	-0.504966	0.191813	H	-5.038343	0.648192	-2.113799
C	-3.749382	0.088035	-0.889396	H	-3.180166	-1.602110	2.004417
C	-2.744539	0.821640	-1.659867	H	-5.715429	-0.041583	1.344983
C	-0.260506	0.540386	-1.862397	H	-5.316689	-0.849085	2.874429
C	1.151947	3.963084	-0.973315	H	-3.989866	1.376685	1.782123
C	5.624312	0.418319	0.345856	H	0.697943	0.585324	-1.343710
C	1.204065	-3.204778	-1.594862	H	-0.335534	-0.430468	-2.359696
C	-3.922887	-1.265802	1.269967	H	-0.280157	1.313330	-2.637194
C	-4.889870	-0.322896	2.014594	H	-3.979033	-3.191661	0.242212
C	-4.672511	-2.489384	0.715828	H	-5.409204	-2.185086	-0.033843
O	-5.041100	0.107544	-1.296875	H	-5.195321	-3.018199	1.521291
O	-4.235805	0.824085	2.540653	H	1.686402	-4.183960	-1.505542
O	3.020320	-3.879520	0.727527	H	0.148480	-3.364192	-1.824158
O	-3.001186	1.423362	-2.696033	H	1.657633	-2.699271	-2.458156
O	2.879792	0.402625	1.894788	H	2.283556	-4.093783	1.321521
H	6.453151	-0.125929	-0.100461	H	1.354458	5.014813	-0.731220
H	5.886102	1.122737	1.131632	H	2.000790	3.613752	-1.575542
H	2.459440	4.268197	1.409204	H	0.265822	3.925870	-1.609422
H	1.914950	2.787097	2.204291	H	3.718296	0.292010	2.371443

Table S19. Conformational analysis of the B3LYP-D3BJ/6-31G(d) optimized conformers of **4b** in the gas phase (T=298.15 K).

Conformer	E (Hartree)[a]	C (Hartree)[b]	G (kcal/mol)[c]	ΔG (kcal/mol)[d]	Population[e]
**4b-1**	-1351.457662	0.524333	-847710.663753	0.0	57.61%
**4b-2**	-1351.457955	0.52519	-847710.309918	0.353835	31.69%
**4b-3**	-1351.455818	0.524676	-847709.29163	1.372123	5.68%
**4b-4**	-1351.455874	0.524848	-847709.218721	1.445032	5.02%

[a]Electronic energy obtained at the M06-2X-D3/def2-TZVP level of theory; [b]Thermal correction to Gibbs free energy obtained at the M06-2X-D3/def2-SVP level of theory; [c]Gibbs free energy (E + C); [d]The relative Gibbs free energy; [e]The Boltzmann distribution of each conformer.

Table S20. Atomic coordinates (Å) of **4b-1** obtained at the B3LYP-D3BJ/6-31G(d) level of theory in the gas phase.

C	-2.548384	0.028172	-1.466492	H	1.875237	2.941143	1.794098
C	-1.886119	1.300781	-2.074843	H	0.838088	1.624929	1.291067
C	-1.099145	2.096211	-1.069867	H	-1.581483	2.218023	-0.099359
C	0.115825	2.647541	-1.208173	H	2.445475	1.206163	-0.645313
C	0.752281	3.346928	-0.020209	H	1.685141	-0.579079	0.860958
C	1.525470	2.387380	0.909465	H	2.815457	-1.204826	2.055598
C	2.757308	1.731053	0.264607	H	4.415299	-1.856061	0.354608
C	3.369095	0.700805	1.200065	H	3.655315	-0.866047	-0.872414
C	2.751917	-0.674751	1.098099	H	2.392026	-3.342505	0.395167
C	3.443718	-1.500571	-0.004301	H	0.172553	-3.353037	0.234492
C	2.629531	-2.709118	-0.475698	H	-1.191063	-2.219594	-2.220210
C	1.332474	-2.299194	-1.142627	H	-1.981687	-2.923442	-0.821208
C	0.156649	-2.699637	-0.636296	H	-2.697258	1.911846	-2.495641
C	-1.189692	-2.230985	-1.125919	H	-1.253438	0.991823	-2.914213
C	-1.509560	-0.793133	-0.626590	H	-0.558423	-0.247808	-0.660644
C	-1.998287	-0.722996	0.813174	H	-4.594626	0.896529	1.619213
C	-3.144688	-0.016824	0.880888	H	-0.183232	-1.385385	1.652503
C	-3.566240	0.473995	-0.423482	H	-2.202010	-0.491586	3.745917
C	-3.284088	-0.741458	-2.572115	H	-0.445551	-0.672146	3.886273
C	0.913716	2.652154	-2.489677	H	-1.884487	1.354226	2.612463
C	4.388301	0.980252	2.012642	H	-2.612165	-0.990667	-3.399768
C	1.463544	-1.397728	-2.345764	H	-3.724563	-1.670078	-2.195202
C	-1.225952	-1.290198	1.972600	H	-4.094080	-0.118863	-2.964208
C	-1.251962	-0.366150	3.211451	H	-1.674998	-3.399360	1.548632
C	-1.744454	-2.685221	2.373614	H	-2.793993	-2.627550	2.681276
O	-3.855026	0.362430	1.979529	H	-1.162721	-3.082329	3.213970
O	-1.028810	1.002562	2.906004	H	2.387727	-1.624975	-2.885094
O	3.480379	-3.422186	-1.382066	H	0.623638	-1.507396	-3.038138
O	-4.561671	1.171928	-0.585919	H	1.502183	-0.339912	-2.054790

O	3.676882	2.719476	-0.196551	H	2.962313	-4.165438	-1.729613
H	4.796295	0.236633	2.692181	H	1.984919	2.546088	-2.293291
H	4.873984	1.952059	2.011493	H	0.605219	1.872021	-3.191536
H	1.431171	4.133742	-0.369195	H	0.790722	3.617402	-3.001171
H	-0.025022	3.834197	0.579583	H	3.806464	3.353748	0.526226

Table S21. Atomic coordinates (Å) of **4b-2** obtained at the B3LYP-D3BJ/6-31G(d) level of theory in the gas phase.

C	1.797606	-1.701494	-0.712071	H	-4.289777	-2.616951	0.782412
C	1.486948	-2.863079	0.281102	H	-3.384775	-1.645699	1.930407
C	0.028259	-3.213366	0.358747	H	-0.358801	-3.738430	-0.512065
C	-0.853192	-2.946174	1.335236	H	-1.893478	-0.851960	0.134273
C	-2.296108	-3.382247	1.177589	H	-3.268883	0.591277	1.636653
C	-3.289865	-2.214077	0.998133	H	-4.984937	0.650269	1.310998
C	-2.867928	-1.263945	-0.134582	H	-4.421880	2.923668	1.297647
C	-3.825059	-0.101111	-0.326030	H	-4.359503	2.573801	-0.421834
C	-4.002708	0.838431	0.858646	H	-2.013572	2.661040	1.522579
C	-3.887602	2.341722	0.538021	H	-0.548002	1.146463	0.995052
C	-2.441523	2.856515	0.524979	H	-0.458598	-0.364541	-1.384385
C	-1.561046	2.174980	-0.502564	H	0.636793	0.930836	-1.787662
C	-0.641170	1.297146	-0.081478	H	1.887473	-2.588752	1.263455
C	0.201010	0.388908	-0.939666	H	2.057147	-3.738093	-0.056091
C	1.304653	-0.330530	-0.134023	H	0.869889	-0.527340	0.855435
C	2.559857	0.512488	0.081121	H	5.437739	-0.645235	-0.595741
C	3.647116	-0.185963	-0.305528	H	1.574788	1.948763	1.265639
C	3.307791	-1.525114	-0.771179	H	3.340084	3.055215	2.288374
C	1.302975	-2.041413	-2.128264	H	3.770453	1.338527	2.365111
C	-0.533121	-2.208812	2.610962	H	5.219312	1.786878	0.623019
C	-4.491179	0.066576	-1.471236	H	1.817891	-2.941241	-2.479479
C	-1.828013	2.476188	-1.953981	H	0.225619	-2.221770	-2.149395
C	2.503577	1.895025	0.683036	H	1.528489	-1.231441	-2.829621
C	3.648647	2.195596	1.682783	H	3.345063	2.971190	-1.012178
C	2.441337	2.990781	-0.399057	H	2.382377	3.976877	0.075407
O	4.964834	0.153056	-0.276930	H	1.566963	2.867308	-1.042712
O	4.873008	2.568803	1.087724	H	-2.565693	1.774673	-2.362309
O	-2.532887	4.267335	0.307236	H	-2.229285	3.487693	-2.064266
O	4.155543	-2.340551	-1.119460	H	-0.920488	2.390576	-2.560020
O	-2.617912	-1.989227	-1.336968	H	-1.625144	4.608526	0.270811
H	-5.192529	0.883869	-1.612829	H	0.534914	-2.017122	2.737031
H	-4.335912	-0.589379	-2.320968	H	-1.048346	-1.238688	2.647109
H	-2.375639	-4.044596	0.310973	H	-0.879630	-2.776306	3.484741

H	-2.607039	-3.964252	2.056964	H	-3.441354	-2.443189	-1.579542

Table S22. Atomic coordinates (Å) of **4b-3** obtained at the B3LYP-D3BJ/6-31G(d) level of theory in the gas phase.

C	-1.367871	-0.768225	0.795517	H	4.033740	-1.877538	-2.367168
C	-1.055334	-2.172208	0.173965	H	2.575105	-0.927021	-2.095282
C	0.187359	-2.266847	-0.668513	H	0.096991	-1.871671	-1.681768
C	1.351849	-2.846797	-0.337125	H	3.015029	-0.842798	0.310544
C	2.470023	-2.968143	-1.363568	H	5.396538	1.993813	0.388325
C	3.243849	-1.664976	-1.634400	H	5.213984	0.580030	1.438355
C	3.843563	-1.038729	-0.375855	H	3.546185	2.372593	1.950049
C	4.614268	0.254427	-0.590450	H	2.778125	0.853663	1.509291
C	4.744819	1.146935	0.623851	H	2.681636	1.818986	-0.905930
C	3.384375	1.677853	1.118589	H	0.624501	1.263361	-1.238059
C	2.589739	2.397297	0.024125	H	-1.551684	2.087076	0.800085
C	1.117638	2.505822	0.360754	H	-1.762475	2.309936	-0.926053
C	0.237550	1.823491	-0.384147	H	-1.916099	-2.455957	-0.447528
C	-1.243946	1.696678	-0.174634	H	-1.022075	-2.888268	0.999918
C	-1.769466	0.246844	-0.330503	H	-1.410517	-0.115713	-1.303910
C	-3.293880	0.253466	-0.338122	H	-4.968550	-1.097440	1.992007
C	-3.758472	-0.376217	0.758362	H	-3.375652	1.187569	-2.227674
C	-2.679879	-0.959231	1.550584	H	-5.432835	0.404714	-3.037706
C	-0.253642	-0.369421	1.764741	H	-4.627997	-0.995077	-2.307189
C	1.658336	-3.445993	1.011847	H	-6.069070	-0.556115	-0.459972
C	5.172413	0.581095	-1.760854	H	-0.416315	0.622887	2.192871
C	0.775694	3.329495	1.574321	H	-0.235071	-1.088869	2.589456
C	-4.085775	0.921151	-1.431864	H	0.717968	-0.375959	1.268194
C	-5.119587	-0.033524	-2.083424	H	-4.044050	2.912601	-0.523655
C	-4.772148	2.208338	-0.937695	H	-5.507792	1.972704	-0.165644
O	-5.041430	-0.559682	1.175331	H	-5.297838	2.697652	-1.764995
O	-6.305684	-0.211848	-1.339164	H	1.368116	4.250223	1.574107
O	3.215159	3.676887	-0.149097	H	-0.282041	3.598110	1.621099
O	-2.874077	-1.564987	2.598339	H	1.026846	2.789954	2.497884
O	4.667542	-1.967714	0.352484	H	2.775090	4.108164	-0.898275
H	5.746069	1.497933	-1.868736	H	2.620522	-3.070803	1.379222
H	5.079477	-0.034147	-2.650495	H	0.893908	-3.238295	1.762762
H	3.182301	-3.728349	-1.026493	H	1.766829	-4.536426	0.927520
H	2.057734	-3.319414	-2.317099	H	5.482426	-2.078837	-0.164217

Table S23. Atomic coordinates (Å) of **4b-4** obtained at the B3LYP-D3BJ/6-31G(d) level of theory in the gas phase.

C	1.805103	-1.688906	-0.724243	H	-4.257101	-2.663303	0.756822
C	1.512657	-2.858916	0.264689	H	-3.357094	-1.702591	1.917686
C	0.059840	-3.231672	0.340096	H	-0.321652	-3.750198	-0.537107
C	-0.823546	-2.987547	1.320780	H	-1.849243	-0.907108	0.129321
C	-2.263285	-3.430771	1.155007	H	-3.159060	0.544884	1.640074
C	-3.258129	-2.262468	0.980771	H	-4.888460	0.554421	1.386772
C	-2.829856	-1.303005	-0.141398	H	-4.385725	2.844898	1.410036
C	-3.770030	-0.123869	-0.311571	H	-4.415638	2.523366	-0.316508
C	-3.932203	0.784287	0.898657	H	-1.976721	2.744253	1.505052
C	-3.874181	2.296477	0.610828	H	-0.582717	1.123658	0.972239
C	-2.446436	2.872802	0.523459	H	-0.462239	-0.376248	-1.407969
C	-1.573391	2.185566	-0.510713	H	0.630838	0.928859	-1.798436
C	-0.668144	1.288104	-0.102838	H	1.908325	-2.581689	1.248106
C	0.185632	0.383981	-0.958469	H	2.097158	-3.723225	-0.075711
C	1.289737	-0.326699	-0.143704	H	0.848779	-0.532634	0.841317
C	2.532584	0.531722	0.082422	H	5.429741	-0.579470	-0.589973
C	3.630995	-0.149748	-0.303690	H	1.510500	1.969166	1.236283
C	3.312912	-1.489914	-0.778635	H	3.241650	3.073595	2.313267
C	1.319311	-2.033092	-2.142351	H	3.669997	1.356834	2.403305
C	-0.510387	-2.263540	2.605829	H	5.166818	1.813518	0.692908
C	-4.429611	0.077130	-1.454940	H	1.847963	-2.924857	-2.493746
C	-1.821109	2.538806	-1.951281	H	0.244590	-2.228233	-2.167538
C	2.456162	1.913734	0.682801	H	1.535917	-1.218756	-2.841445
C	3.569744	2.214456	1.717669	H	1.572716	2.878342	-1.073716
C	2.425791	3.007019	-0.403098	H	3.349205	2.989194	-0.986502
O	4.943371	0.209935	-0.269746	H	2.343578	3.993138	0.066592
O	4.812966	2.588492	1.163292	H	-1.678719	3.614750	-2.112452
O	-2.502338	4.290039	0.338500	H	-1.149608	2.005529	-2.627192
O	4.172315	-2.291803	-1.130519	H	-2.847549	2.287311	-2.246458
O	-2.593832	-2.014776	-1.354133	H	-3.059494	4.459603	-0.437329
H	-5.123845	0.902695	-1.582866	H	-0.849360	-2.846613	3.472275
H	-4.278355	-0.563832	-2.316511	H	0.555445	-2.061293	2.733261
H	-2.337214	-4.085967	0.282429	H	-1.036236	-1.299723	2.655889
H	-2.576108	-4.020486	2.028430	H	-3.421895	-2.460513	-1.596199

S3. *Computational data of compound 6*

Table S24. Experimental and calculated NMR data of **6a** and **6b**.

No.	**6a**		**6b**		**6**	
	$\delta_{calcd.\ H}$	$\delta_{calcd.\ C}$	$\delta_{calcd.\ H}$	$\delta_{calcd.\ C}$	$\delta_{exptl.\ H}$	$\delta_{exptl.\ C}$
1		52.2		54.2		49.8
2	2.16	39.9	2.06	41.5	2.08	39.9
	2.00		1.62		1.95	
3	5.49	123.7	5.65	121.7	5.27	120.9
4		138.2		141.4		138.4
5	2.71	43.8	2.78	46.0	2.79	42.9
	2.70					
6	6.03	128.9	6.19	124.4	5.76	126.1
7	5.78	136.8	6.03	139.6	5.49	138.0
8		75.4		73.2		74.3
9	1.54	38.5	1.71	41.4	1.75	39.0
	1.20		1.56		1.65	
10	1.52	31.2	2.03	30.2	1.63	30.6
	1.12		1.31		1.52	
11	3.73	78.6	3.65	77.5	3.89	80.4
12		140.4		138.9		139.1
13	6.06	128.6	5.78	124.0	5.21	127.4
14	2.56	32.4	2.20	32.7	2.35	30.0
	1.60		1.98		1.88	
15	2.82	53.9	2.77	53.9	2.51	50.9
16		155.1		152.7		152.1
17		142.5		142.9		148.8
18		203.7		204.8		210.1
19	1.02	19.8	0.82	17.9	1.00	16.5
20	1.57	18.6	1.63	17.7	1.68	18.0
21	1.09	23.6	1.06	30.7	1.27	30.4
22	1.68	14.2	1.63	18.8	1.57	11.4
23	2.93	42.4	2.71	41.2	2.60	38.8
24	3.87	66.8	3.97	67.2	3.83	65.9
	3.63		3.65		3.69	
25	1.15	15.0	1.15	14.8	1.25	14.6

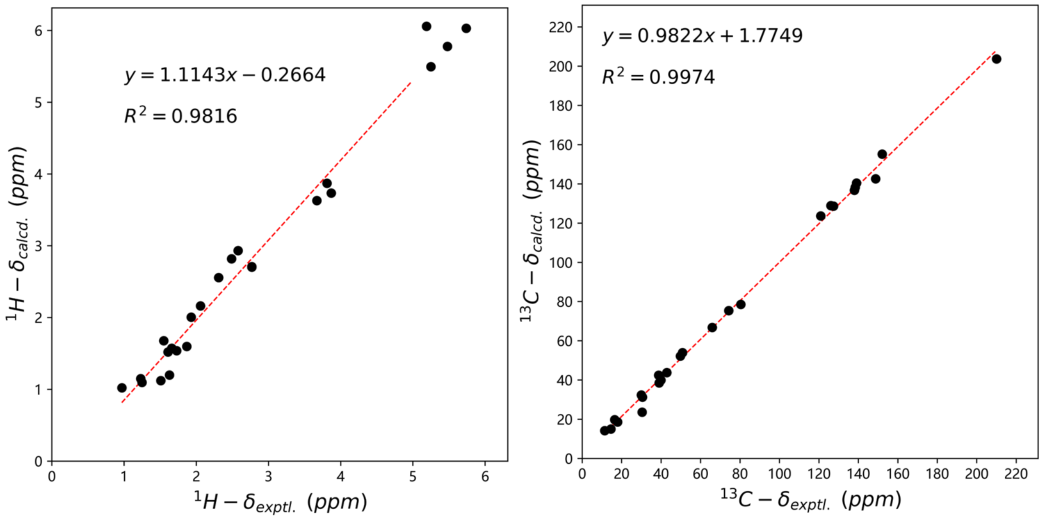

Figure S7. Linear regression analyses between experimental and calculated NMR data of **6a**.

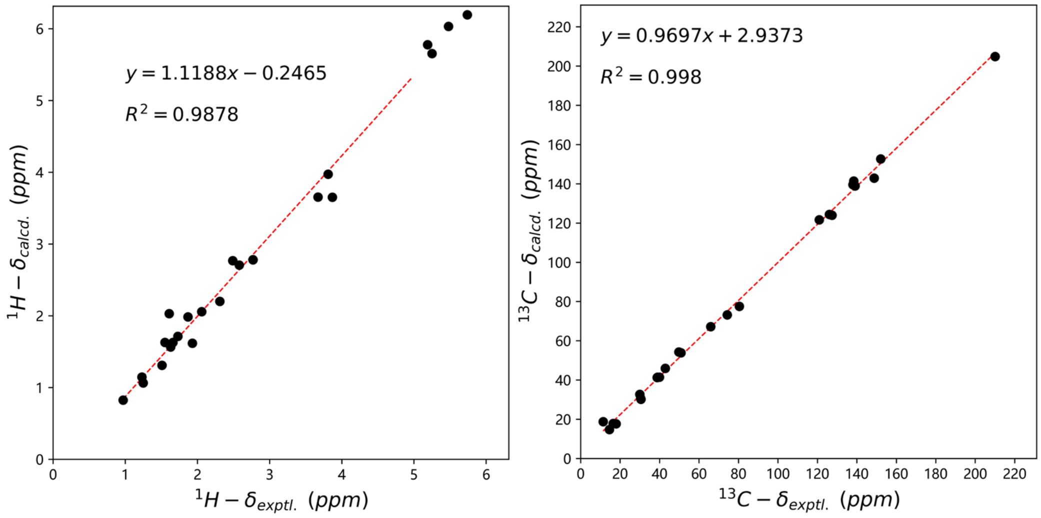

Figure S8. Linear regression analyses between experimental and calculated NMR data of **6b**.

Functional	Solvent?	Basis Set	Type of Data
mPW1PW91	PCM	6-31+G(d,p)	Unscaled Shifts

	Isomer 1	Isomer 2	Isomer 3	Isomer 4	Isomer 5	Isomer 6
sDP4+ (H data)	0.00%	100.00%	–	–	–	–
sDP4+ (C data)	33.62%	66.38%	–	–	–	–
sDP4+ (all data)	0.00%	100.00%	–	–	–	–
uDP4+ (H data)	0.13%	99.87%	–	–	–	–
uDP4+ (C data)	1.09%	98.91%	–	–	–	–
uDP4+ (all data)	0.00%	100.00%	–	–	–	–
DP4+ (H data)	0.00%	100.00%	–	–	–	–
DP4+ (C data)	0.55%	99.45%	–	–	–	–
DP4+ (all data)	0.00%	100.00%	–	–	–	–

Figure S9. DP4+ analysis results of **6a** (left) and **6b** (right).

Table S25. Conformational analysis of the B3LYP-D3BJ/6-31G(d) optimized conformers of **6a** in the gas phase (T=298.15 K).

Conformer	E (Hartree)[a]	C (Hartree)[b]	G (kcal/mol)[c]	ΔG (kcal/mol)[d]	Population[e]
**6a-1**	-1351.470102	0.528558	-847715.818891	0.0	46.25%
**6a-2**	-1351.467183	0.526647	-847715.186403	0.632489	15.89%
**6a-3**	-1351.467463	0.526956	-847715.168105	0.650787	15.41%
**6a-4**	-1351.465041	0.524983	-847714.886558	0.932333	9.58%
**6a-5**	-1351.465979	0.526274	-847714.664643	1.154249	6.58%
**6a-6**	-1351.46774	0.52808	-847714.636637	1.182254	6.28%

[a]Electronic energy obtained at the M06-2X-D3/def2-TZVP level of theory; [b]Thermal correction to Gibbs free energy obtained at the M06-2X-D3/def2-SVP level of theory; [c]Gibbs free energy (E + C); [d]The relative Gibbs free energy; [e]The Boltzmann distribution of each conformer.

Table S26. Atomic coordinates (Å) of **6a-1** obtained at the B3LYP-D3BJ/6-31G(d) level of theory in the gas phase.

C	-2.573097	0.310011	-1.333809	H	1.092465	1.157150	0.445872
C	-2.232796	1.830239	-1.164528	H	1.873036	-1.068524	1.052192
C	-0.848689	2.186648	-1.628220	H	3.384644	-1.326741	1.911170
C	0.024873	2.926502	-0.930510	H	3.667101	-0.448792	-0.967662
C	1.447715	3.110728	-1.406574	H	4.601696	-1.647053	-0.087505
C	2.459977	2.396570	-0.529928	H	2.579347	-3.188487	-0.188440
C	2.136686	1.443856	0.347607	H	0.336167	-3.118100	-0.234745
C	3.035890	0.700278	1.303792	H	-1.022097	-1.601028	-2.489248
C	2.938099	-0.812773	1.052302	H	-1.813828	-2.610825	-1.289522
C	3.584531	-1.280588	-0.259960	H	-2.335272	2.093864	-0.107654
C	2.788739	-2.404716	-0.934920	H	-3.002236	2.397744	-1.705948
C	1.474071	-1.904630	-1.500196	H	-0.555206	0.047063	-0.535894
C	0.307509	-2.356655	-1.013671	H	-5.057731	-0.386310	1.464591
C	-1.045613	-1.840485	-1.423292	H	-0.177780	-1.630839	1.474612
C	-1.462142	-0.554354	-0.643017	H	-2.020600	-0.061801	3.320085
C	-2.017272	-0.875948	0.732467	H	-0.483182	-0.796785	3.811585
C	-3.332237	-0.575543	0.785360	H	0.587853	0.692524	2.576488
C	-3.789773	0.062825	-0.447965	H	-3.153116	-1.050378	-2.947378
C	-2.897503	0.002661	-2.797361	H	-3.763558	0.598391	-3.101194
C	-0.321128	3.568284	0.399918	H	-2.060892	0.247634	-3.460409
C	4.482447	1.187706	1.334520	H	-1.129541	-3.108938	3.239378
C	1.585139	-0.854919	-2.580138	H	-1.829553	-3.497228	1.656308
C	-1.182552	-1.422904	1.857830	H	-2.770709	-2.564048	2.827483
C	-1.028112	-0.355880	2.963252	H	1.617760	0.152460	-2.149099
C	-1.764060	-2.726192	2.431524	H	2.506903	-1.003201	-3.150360
O	-4.952748	0.396353	-0.646175	H	0.741278	-0.885668	-3.275354
O	3.638973	-2.925900	-1.963216	H	3.122894	-3.590854	-2.446054

O	-4.212547	-0.746871	1.803295	H	0.515697	4.168007	0.771075
O	-0.379922	0.818395	2.500574	H	-1.188948	4.231285	0.307882
O	2.460054	0.865133	2.637666	H	-0.554551	2.819355	1.165922
H	1.705123	4.180770	-1.423285	H	4.977849	1.031338	0.371870
H	1.556365	2.759965	-2.443012	H	5.037211	0.641520	2.102998
H	3.498237	2.702947	-0.652914	H	4.530133	2.257810	1.566922
H	-0.527294	1.765435	-2.580883	H	2.459653	1.819130	2.826073

Table S27. Atomic coordinates (Å) of **6a-2** obtained at the B3LYP-D3BJ/6-31G(d) level of theory in the gas phase.

C	-2.701759	-1.260608	0.230815	H	0.909919	0.257252	-0.995495
C	-2.268715	-2.127288	-0.989447	H	2.011516	1.083902	0.905381
C	-1.400506	-1.399162	-1.977824	H	3.674684	1.641994	0.932909
C	-0.228731	-1.798282	-2.495031	H	3.201468	-1.275662	0.220023
C	0.541808	-0.902250	-3.444079	H	4.545026	-0.561251	1.099376
C	1.775338	-0.259469	-2.825328	H	2.863324	-0.040360	2.988139
C	1.809163	0.290325	-1.608232	H	0.617520	0.371851	3.144957
C	2.951604	1.082263	-1.003220	H	-1.312859	-1.837938	2.497575
C	3.012591	0.875083	0.515045	H	-1.731028	-0.208491	2.992498
C	3.458525	-0.519372	0.970830	H	-3.192083	-2.464226	-1.480949
C	2.810028	-0.906946	2.309249	H	-1.764281	-3.022210	-0.611671
C	1.357478	-1.300152	2.124553	H	-0.609125	-0.706289	0.229971
C	0.361922	-0.523992	2.580453	H	-4.539637	1.948700	-0.717285
C	-1.100214	-0.781653	2.305364	H	0.087702	1.679093	1.213046
C	-1.492496	-0.463511	0.829386	H	-0.366644	2.325597	-1.183069
C	-1.822731	1.003381	0.604830	H	-1.510658	3.538413	-0.590110
C	-3.044310	1.135179	0.041891	H	1.272168	3.459677	-0.248182
C	-3.643817	-0.159292	-0.249789	H	-3.774575	-1.591213	2.111451
C	-3.478994	-2.146775	1.215802	H	-4.389309	-2.509790	0.728959
C	0.436406	-3.125069	-2.231871	H	-2.887169	-3.013593	1.528211
C	4.307983	0.841798	-1.665895	H	-1.436217	2.426679	3.016276
C	1.123860	-2.561437	1.329030	H	-2.356754	3.407103	1.858694
C	-0.871574	2.119696	0.922515	H	-0.669370	3.828442	2.228225
C	-0.602062	2.985772	-0.333903	H	0.204823	-3.074262	1.629065
C	-1.366307	2.997957	2.083832	H	1.035252	-2.343635	0.259376
O	-4.722987	-0.294435	-0.817767	H	1.964111	-3.249121	1.458475
O	3.589155	-1.986090	2.836015	H	3.159124	-2.272605	3.657462
O	-3.709544	2.263930	-0.304379	H	-0.049328	-3.708453	-1.447574
O	0.425163	3.929807	-0.116504	H	0.435725	-3.733253	-3.146992
O	2.618378	2.496180	-1.136423	H	1.487017	-2.981918	-1.951123
H	-0.116943	-0.109575	-3.823282	H	5.062909	1.468873	-1.182832

H	0.854809	-1.483439	-4.321492	H	4.284628	1.104463	-2.729100
H	2.663331	-0.228692	-3.454472	H	4.613345	-0.205449	-1.581151
H	-1.776437	-0.424316	-2.290259	H	2.385753	2.651407	-2.066427

Table S28. Atomic coordinates (Å) of **6a-3** obtained at the B3LYP-D3BJ/6-31G(d) level of theory in the gas phase.

C	-2.596888	0.296725	-1.325956	H	1.095974	1.132610	0.424752
C	-2.280437	1.828562	-1.175264	H	1.898257	-1.076575	1.075511
C	-0.882684	2.192478	-1.587427	H	3.426767	-1.307021	1.907490
C	-0.008749	2.950185	-0.906548	H	3.676173	-0.437642	-0.971495
C	1.414271	3.114872	-1.399452	H	4.603864	-1.648980	-0.106413
C	2.438664	2.402943	-0.532412	H	2.579986	-3.179822	-0.209334
C	2.137415	1.440966	0.340611	H	0.336414	-3.138212	-0.273166
C	3.036384	0.719202	1.320314	H	-1.042348	-1.614794	-2.506626
C	2.960091	-0.802465	1.054009	H	-1.822364	-2.616880	-1.292717
C	3.588382	-1.273141	-0.268605	H	-2.449865	2.122483	-0.134758
C	2.786279	-2.388907	-0.949085	H	-3.030146	2.370566	-1.766693
C	1.467867	-1.886073	-1.505950	H	-0.549659	0.047320	-0.597419
C	0.303733	-2.360669	-1.035613	H	-4.993258	-0.405051	1.554865
C	-1.054496	-1.848383	-1.439032	H	-0.102927	-1.607581	1.416831
C	-1.459476	-0.556240	-0.664060	H	-1.943819	-0.271762	3.445814
C	-1.969715	-0.868914	0.733702	H	-0.301344	-0.879650	3.749602
C	-3.282294	-0.565011	0.829352	H	-0.922813	1.188983	1.925241
C	-3.786176	0.044972	-0.406183	H	-3.186579	-1.078667	-2.922603
C	-2.949489	-0.020555	-2.780693	H	-3.833051	0.557552	-3.067299
C	-0.314061	3.674258	0.382012	H	-2.131337	0.239860	-3.460617
C	4.478119	1.217821	1.322591	H	-1.000643	-3.214849	3.103510
C	1.575608	-0.817894	-2.566535	H	-1.693670	-3.529168	1.501841
C	-1.106868	-1.454599	1.821803	H	-2.659206	-2.694791	2.728211
C	-0.964826	-0.462102	2.987277	H	1.667177	0.175577	-2.112646
C	-1.649666	-2.804619	2.321474	H	2.471796	-0.986725	-3.170960
O	-4.960135	0.347910	-0.575320	H	0.707311	-0.800795	-3.231409
O	3.623908	-2.904473	-1.991848	H	3.108405	-3.579746	-2.460881
O	-4.128220	-0.737711	1.872207	H	-0.232298	4.758820	0.229620
O	-0.353252	0.767216	2.588080	H	-1.319751	3.480356	0.763399
O	2.538315	0.976805	2.641836	H	0.411303	3.411876	1.159923
H	1.675036	4.183455	-1.428812	H	5.040047	0.689870	2.099160
H	1.497187	2.758505	-2.436177	H	4.504649	2.287070	1.550667
H	3.469181	2.734565	-0.647569	H	4.969440	1.046585	0.360207
H	-0.539060	1.748424	-2.522078	H	1.564319	0.895789	2.621422

Table S29. Atomic coordinates (Å) of **6a-4** obtained at the B3LYP-D3BJ/6-31G(d) level of theory in the gas phase.

C	-2.754956	0.484212	-1.105434	H	0.707809	1.257737	1.018925
C	-2.515969	1.939946	-1.578811	H	1.908811	-0.895060	0.913761
C	-1.564416	2.715016	-0.708610	H	3.552501	-1.072088	1.509231
C	-0.299024	3.057256	-0.998140	H	3.017720	0.452370	-1.090665
C	0.560787	3.799001	0.004782	H	4.514341	-0.287714	-0.560624
C	1.753214	2.996755	0.510810	H	3.522811	-2.542699	-0.725899
C	1.678645	1.755297	0.994344	H	1.111761	0.011030	-1.465771
C	2.794457	0.927235	1.609270	H	-0.954600	-0.530099	-2.824758
C	2.904266	-0.438242	0.891639	H	-1.114822	-2.063193	-1.983503
C	3.426597	-0.412327	-0.551407	H	-3.493882	2.437811	-1.588991
C	3.122458	-1.707505	-1.315086	H	-2.166918	1.912306	-2.615524
C	1.649435	-1.993835	-1.590109	H	-0.735329	0.495508	-0.427672
C	0.756352	-0.998812	-1.656250	H	-4.314582	-0.350639	2.276877
C	-0.731110	-1.044143	-1.879335	H	0.066683	-2.098088	0.572405
C	-1.445415	-0.278351	-0.741382	H	-1.491298	-2.086064	3.196123
C	-1.780082	-1.069645	0.516226	H	0.202932	-2.542359	2.923248
C	-2.929771	-0.600027	1.049105	H	-0.883212	0.053657	2.725688
C	-3.531516	0.433782	0.207129	H	-3.779867	-1.307019	-1.840260
C	-3.615937	-0.266907	-2.141301	H	-4.591516	0.220368	-2.235400
C	0.393412	2.759832	-2.304386	H	-3.135019	-0.264431	-3.124389
C	4.139133	1.647411	1.645363	H	-2.476044	-3.597927	1.410252
C	1.336249	-3.448426	-1.818788	H	-0.844806	-4.300751	1.340788
C	-0.888156	-2.131949	1.101876	H	-1.642043	-3.760381	-0.145564
C	-0.600437	-1.878196	2.592733	H	0.321967	-3.617184	-2.188284
C	-1.501392	-3.532618	0.915373	H	2.041879	-3.867090	-2.545750
O	-4.517434	1.083032	0.535691	H	1.458676	-4.022069	-0.889265
O	3.859962	-1.778681	-2.547220	H	3.456521	-1.123994	-3.140229
O	-3.521225	-0.923081	2.228537	H	0.588822	3.690536	-2.854570
O	-0.135425	-0.551632	2.848805	H	1.368915	2.296711	-2.116438
O	2.457468	0.683246	2.981803	H	-0.175466	2.097075	-2.959415
H	-0.057087	4.115741	0.855114	H	4.059563	2.561785	2.239864
H	0.935063	4.721403	-0.460240	H	4.488527	1.905534	0.641344
H	2.714423	3.505820	0.494064	H	4.882625	0.998384	2.117934
H	-1.953583	2.989587	0.272599	H	1.600013	0.213854	2.996397

Table S30. Atomic coordinates (Å) of **6a-5** obtained at the B3LYP-D3BJ/6-31G(d) level of theory in the gas phase.

C	-2.823936	0.291438	-1.132346	H	2.836213	2.421284	-0.619238
C	-2.656157	1.707269	-1.745371	H	1.885194	-0.450417	0.815480
C	-1.742387	2.609410	-0.961232	H	3.463633	-0.852137	1.482316
C	-0.462834	2.893491	-1.250270	H	3.095624	0.193553	-1.385828
C	0.404351	3.704042	-0.297720	H	4.556235	-0.480354	-0.688644
C	1.189562	2.792098	0.625126	H	3.458366	-2.657411	-0.352712
C	2.353390	2.206994	0.335076	H	1.098977	-0.214795	-1.459837
C	3.100874	1.248341	1.252538	H	-0.988156	-0.896955	-2.701326
C	2.950582	-0.211106	0.753117	H	-1.165728	-2.302917	-1.664447
C	3.463105	-0.537934	-0.654166	H	-3.658595	2.149247	-1.799457
C	3.082359	-1.953710	-1.106871	H	-2.306217	1.595982	-2.776056
C	1.594999	-2.229756	-1.302000	H	-0.787977	0.451896	-0.471922
C	0.720844	-1.233410	-1.493917	H	-4.421972	-0.251367	2.292051
C	-0.769297	-1.283814	-1.696231	H	0.071904	-1.981602	0.864542
C	-1.485835	-0.370621	-0.671911	H	-1.323404	-1.194883	3.462327
C	-1.799219	-1.000137	0.680016	H	0.355698	-1.740727	3.253328
C	-2.991889	-0.549468	1.130783	H	-0.687436	0.646413	2.155595
C	-3.631917	0.338371	0.160768	H	-3.757300	-1.608317	-1.702090
C	-3.633470	-0.595868	-2.100725	H	-4.627659	-0.163771	-2.251491
C	0.264907	2.405630	-2.475575	H	-3.139169	-0.667025	-3.074388
C	4.571884	1.667602	1.337005	H	-2.371042	-3.203433	2.269291
C	1.241920	-3.693853	-1.295511	H	-0.730530	-3.887075	2.282451
C	-0.843722	-1.875401	1.449798	H	-1.638263	-3.787092	0.764160
C	-0.465237	-1.196141	2.779257	H	0.217119	-3.890670	-1.618812
C	-1.433818	-3.272559	1.708305	H	1.924139	-4.241594	-1.955255
O	-4.663879	0.957339	0.390843	H	1.365344	-4.115765	-0.288013
O	3.793674	-2.325168	-2.300304	H	3.409332	-1.796663	-3.018749
O	-3.595954	-0.776757	2.322739	H	0.540851	3.253691	-3.116898
O	0.014777	0.136815	2.590933	H	1.201988	1.914793	-2.188513
O	2.607383	1.306981	2.585575	H	-0.311225	1.700782	-3.077739
H	-0.222570	4.375906	0.298434	H	5.124542	0.961029	1.964515
H	1.092641	4.332894	-0.877206	H	4.644661	2.661640	1.788278
H	0.738388	2.589693	1.594088	H	5.037696	1.699284	0.346543
H	-2.163047	3.019887	-0.042103	H	1.739395	0.857868	2.611386

Table S31. Atomic coordinates (Å) of **6a-6** obtained at the B3LYP-D3BJ/6-31G(d) level of theory in the gas phase.

C	2.578862	-0.119796	-1.360979	H	-1.025998	-1.232496	0.394780
C	2.269337	-1.657542	-1.370149	H	-1.898501	0.986717	1.135314
C	0.884017	-1.976100	-1.856870	H	-3.430519	1.138575	1.986364
C	0.022725	-2.795506	-1.236885	H	-3.646358	0.443162	-0.941534
C	-1.407093	-2.922152	-1.710653	H	-4.638160	1.537223	0.011098
C	-2.406080	-2.308395	-0.747155	H	-2.667945	3.152578	0.061982
C	-2.072262	-1.488681	0.250710	H	-0.417618	3.117339	0.070898
C	-2.979595	-0.847049	1.269526	H	1.012298	1.890407	-2.307312
C	-2.950651	0.686562	1.105664	H	1.761739	2.761639	-0.978806
C	-3.605701	1.224100	-0.174981	H	2.389524	-2.045343	-0.354330
C	-2.843338	2.426037	-0.748678	H	3.041000	-2.141200	-1.984564
C	-1.506607	2.015810	-1.334150	H	0.542825	0.010899	-0.577282
C	-0.359283	2.440265	-0.781137	H	5.009284	0.308260	1.539762
C	1.011770	1.999575	-1.219870	H	0.082239	1.348679	1.621075
C	1.439705	0.635656	-0.592880	H	2.033995	-0.218078	3.355404
C	1.966114	0.796885	0.820969	H	0.423225	0.321103	3.861175
C	3.288677	0.531475	0.857875	H	-0.466805	-1.175537	2.509763
C	3.778605	0.054607	-0.435346	H	3.143717	1.425781	-2.804630
C	2.911208	0.357169	-2.776283	H	3.794020	-0.181448	-3.133749
C	0.388692	-3.593042	0.000230	H	2.087256	0.168881	-3.473013
C	-4.403117	-1.398696	1.287469	H	2.611629	2.330934	3.056230
C	-1.572548	1.073049	-2.511910	H	0.941755	2.718722	3.525154
C	1.099154	1.176409	1.989424	H	1.594908	3.302627	1.982655
C	1.024850	0.005947	2.994245	H	-0.723942	1.199178	-3.190486
C	1.591886	2.458979	2.681215	H	-1.572296	0.029100	-2.178279
O	4.952787	-0.221374	-0.654052	H	-2.493984	1.243285	-3.076254
O	-3.701359	3.001351	-1.740054	H	-3.202586	3.717188	-2.164918
O	4.150527	0.607919	1.902638	H	-0.459523	-4.193640	0.341191
O	0.507209	-1.181419	2.419897	H	1.222036	-4.276158	-0.202274
O	-2.339053	-1.164625	2.540848	H	0.683435	-2.945345	0.833685
H	-1.670701	-3.982936	-1.840299	H	-4.979990	-0.926349	2.091825
H	-1.528418	-2.459951	-2.701041	H	-4.389371	-2.478341	1.458230
H	-3.448133	-2.582114	-0.907770	H	-4.923039	-1.196018	0.346338
H	0.546502	-1.448529	-2.749318	H	-2.794357	-0.657001	3.232326

Table S32. Conformational analysis of the B3LYP-D3BJ/6-31G(d) optimized conformers of **6b** in the gas phase (T=298.15 K).

Conformer	E (Hartree)[a]	C (Hartree)[b]	G (kcal/mol)[c]	ΔG (kcal/mol)[d]	Population[e]
**6b-1**	-1351.463395	0.525114	-847713.77134	0.0	42.89%
**6b-2**	-1351.462898	0.525222	-847713.391477	0.379863	22.58%
**6b-3**	-1351.461775	0.524647	-847713.047519	0.723821	12.63%
**6b-4**	-1351.461823	0.524814	-847712.973191	0.798149	11.14%
**6b-5**	-1351.460694	0.523717	-847712.953042	0.818298	10.77%

[a]Electronic energy obtained at the M06-2X-D3/def2-TZVP level of theory; [b]Thermal correction to Gibbs free energy obtained at the M06-2X-D3/def2-SVP level of theory; [c]Gibbs free energy (E + C); [d]The relative Gibbs free energy; [e]The Boltzmann distribution of each conformer.

Table S33. Atomic coordinates (Å) of **6b-1** obtained at the B3LYP-D3BJ/6-31G(d) level of theory in the gas phase.

C	-1.716927	0.807169	-0.809720	H	3.660629	0.438464	2.279061
C	-1.286068	2.078205	-0.007679	H	4.689628	-2.096837	1.894439
C	0.061752	2.023969	0.659313	H	5.109813	-2.411402	0.215499
C	1.186248	2.653430	0.281755	H	2.747032	-3.035913	0.947688
C	2.426238	2.604880	1.165067	H	2.417228	-1.341022	1.235785
C	3.506457	1.707568	0.617371	H	3.084527	-2.625282	-1.452540
C	4.009403	0.656239	1.267390	H	0.477001	-1.464002	0.829780
C	5.027944	-0.318957	0.704527	H	-1.717062	-2.014675	-1.274055
C	4.493344	-1.775875	0.863379	H	-1.809931	-2.532528	0.397854
C	2.996100	-2.023638	0.605000	H	-2.041576	2.249373	0.773253
C	2.511867	-1.917520	-0.842270	H	-1.354789	2.929571	-0.690848
C	1.023917	-2.158439	-1.063181	H	-1.533961	-0.173234	1.151602
C	0.119958	-1.810817	-0.138452	H	-5.460908	1.195310	-1.472219
C	-1.375298	-1.770450	-0.264832	H	-3.334053	-1.889882	1.906173
C	-1.960737	-0.400068	0.164531	H	-5.748234	-0.029154	1.883777
C	-3.470100	-0.494449	0.316309	H	-5.240169	-1.119358	3.189223
C	-4.088271	0.323154	-0.561305	H	-3.848146	1.147847	2.280474
C	-3.122890	1.110694	-1.327098	H	-1.034743	-0.293112	-2.574025
C	-0.777335	0.601388	-2.001426	H	-0.876450	1.460251	-2.672593
C	1.333020	3.463591	-0.980151	H	0.263244	0.515841	-1.688865
C	6.351223	-0.183049	1.470201	H	-5.854773	-1.921898	0.144222
C	0.699397	-2.634058	-2.453967	H	-5.533140	-3.025881	1.499083
C	-4.123908	-1.369866	1.349892	H	-4.515987	-3.076817	0.047174
C	-4.877018	-0.491174	2.369618	H	1.132848	-3.627720	-2.629976
C	-5.064638	-2.411453	0.721523	H	-0.372265	-2.700551	-2.650567
O	-3.451735	1.922577	-2.183778	H	1.144946	-1.955750	-3.191975
O	2.817046	-0.633355	-1.443961	H	2.416980	0.042845	-0.867486
O	-5.412540	0.513815	-0.770011	H	0.431298	3.466671	-1.594697

O	-4.036969	0.488567	2.966619	H	1.593130	4.503911	-0.743484
O	5.348929	-0.032232	-0.648771	H	2.150717	3.071543	-1.598677
H	2.151654	2.270457	2.171713	H	6.782564	0.802747	1.275541
H	2.815103	3.630522	1.264003	H	7.058848	-0.946256	1.128933
H	3.887100	1.925245	-0.379476	H	6.202284	-0.296757	2.549193
H	0.097643	1.459622	1.591529	H	4.542050	-0.230019	-1.170059

Table S34. Atomic coordinates (Å) of **6b-2** obtained at the B3LYP-D3BJ/6-31G(d) level of theory in the gas phase.

C	-1.732889	0.749434	-0.864896	H	3.603828	0.494501	2.263809
C	-1.349387	2.038922	-0.064923	H	4.662651	-2.035278	1.930270
C	-0.003069	2.021186	0.607728	H	5.102527	-2.374364	0.261140
C	1.117681	2.650680	0.219362	H	2.739237	-3.013301	0.986347
C	2.355799	2.630550	1.106626	H	2.388109	-1.315886	1.234064
C	3.447091	1.733698	0.580138	H	3.096585	-2.648111	-1.420178
C	3.956942	0.698709	1.250744	H	0.457391	-1.500543	0.824215
C	4.990569	-0.274653	0.712348	H	-1.719333	-2.068310	-1.292314
C	4.472114	-1.735121	0.891804	H	-1.823355	-2.572360	0.383872
C	2.980453	-2.005863	0.624221	H	-2.112849	2.176626	0.712428
C	2.509485	-1.935340	-0.829570	H	-1.436979	2.886124	-0.751645
C	1.026519	-2.195471	-1.061113	H	-1.523298	-0.194042	1.109693
C	0.111099	-1.854133	-0.145417	H	-5.471306	1.107660	-1.516685
C	-1.383433	-1.816184	-0.282897	H	-3.323203	-1.898531	1.918979
C	-1.961548	-0.438864	0.134084	H	-5.380117	0.336581	2.044583
C	-3.466409	-0.518251	0.311346	H	-5.061422	-0.863075	3.324575
C	-4.096010	0.254110	-0.595989	H	-3.824400	1.169518	3.513298
C	-3.140243	1.013077	-1.398651	H	-1.000309	-0.358585	-2.602605
C	-0.772319	0.549373	-2.038617	H	-0.881032	1.396239	-2.723575
C	1.260812	3.433763	-1.060102	H	0.265668	0.492454	-1.711114
C	6.305997	-0.110600	1.486215	H	-4.847715	-2.953594	0.242505
C	0.718149	-2.680585	-2.452372	H	-6.024438	-1.653540	0.471227
C	-4.099259	-1.293072	1.433357	H	-5.659683	-2.768596	1.808126
C	-4.606486	-0.304254	2.491314	H	1.166273	-3.668991	-2.621738
C	-5.229069	-2.221751	0.961538	H	-0.351487	-2.762557	-2.654156
O	-3.477953	1.791032	-2.284298	H	1.158357	-1.999145	-3.190597
O	2.807851	-0.659213	-1.452131	H	2.385561	0.022027	-0.897954
O	-5.423387	0.453202	-0.789122	H	2.071090	3.024618	-1.677609
O	-3.491187	0.464429	2.939144	H	0.353744	3.428776	-1.666712
O	5.319840	-0.007342	-0.642881	H	1.528416	4.477506	-0.847429
H	2.081163	2.312026	2.118511	H	6.727631	0.876706	1.278254
H	2.735805	3.661232	1.187977	H	7.025181	-0.871263	1.163784

H	3.830854	1.937869	-0.418397	H	6.149516	-0.207652	2.565813
H	0.034950	1.478771	1.552787	H	4.519542	-0.224533	-1.166911

Table S35. Atomic coordinates (Å) of **6b-3** obtained at the B3LYP-D3BJ/6-31G(d) level of theory in the gas phase.

C	-2.591672	0.075365	-1.380900	H	1.089910	1.279162	0.536755
C	-2.296618	1.618219	-1.434655	H	1.988936	-0.942284	1.259771
C	-0.888222	1.932286	-1.851355	H	3.555178	-1.080340	2.050519
C	-0.028482	2.771031	-1.252752	H	3.719617	-0.497200	-0.881176
C	1.406316	2.855918	-1.729976	H	4.668067	-1.611137	0.086820
C	2.417734	2.225549	-0.791966	H	2.672543	-3.185416	0.139533
C	2.126438	1.475542	0.272012	H	0.421954	-3.097363	0.192841
C	3.125617	0.871241	1.235833	H	-1.020601	-1.981933	-2.225642
C	3.045787	-0.666673	1.172602	H	-1.754680	-2.797311	-0.858728
C	3.645853	-1.270535	-0.107627	H	-2.500766	2.051766	-0.450532
C	2.849702	-2.461339	-0.664684	H	-3.030593	2.063657	-2.119054
C	1.509064	-2.039621	-1.242746	H	-0.565134	-0.027313	-0.562282
C	0.362053	-2.442534	-0.675808	H	-5.072561	-0.280827	1.492796
C	-1.015373	-2.036459	-1.134690	H	-0.132899	-1.277855	1.671632
C	-1.462715	-0.652104	-0.571845	H	-2.123462	0.192644	3.453333
C	-2.012203	-0.770163	0.838608	H	-0.426574	-0.176475	3.827339
C	-3.335373	-0.498577	0.849563	H	-1.286806	1.491762	1.722436
C	-3.807491	-0.073576	-0.472343	H	-3.116520	-1.516223	-2.786912
C	-2.893482	-0.445649	-2.787457	H	-3.772368	0.074941	-3.179524
C	-0.369355	3.671599	-0.091167	H	-2.056087	-0.272657	-3.471867
C	2.798733	1.361763	2.655571	H	-1.595680	-3.267625	2.025071
C	1.577176	-1.143279	-2.453378	H	-2.668355	-2.315734	3.063673
C	-1.158402	-1.131504	2.024797	H	-1.000178	-2.652681	3.577669
C	-1.126258	0.045948	3.015841	H	1.842869	-0.118877	-2.167688
C	-1.636142	-2.419204	2.715884	H	2.341845	-1.505666	-3.149517
O	-4.979515	0.182438	-0.717777	H	0.631172	-1.099075	-2.997633
O	3.627666	-3.169479	-1.640220	H	4.154012	-2.512723	-2.121530
O	-4.212733	-0.544661	1.880807	H	-0.212227	4.720731	-0.375507
O	-0.652507	1.249358	2.415848	H	-1.407506	3.580187	0.236960
O	4.476561	1.212763	0.904125	H	0.281371	3.476597	0.767859
H	1.687994	3.909938	-1.879665	H	3.487865	0.909296	3.376235
H	1.498063	2.383285	-2.718360	H	1.767583	1.121825	2.928874
H	3.464154	2.404666	-1.037701	H	2.901969	2.452925	2.711181
H	-0.518978	1.365233	-2.706323	H	4.554997	2.175292	1.001916

Table S36. Atomic coordinates (Å) of **6b-4** obtained at the B3LYP-D3BJ/6-31G(d) level of theory in the gas phase.

C	-1.606443	1.103416	-0.643091	H	3.699406	-0.087310	2.374223
C	-1.133940	2.177086	0.389813	H	4.622765	-2.546847	1.525327
C	0.198966	1.935566	1.045601	H	5.058578	-2.558480	-0.178863
C	1.356042	2.574504	0.808276	H	2.657960	-3.199679	0.400970
C	2.575966	2.303194	1.679364	H	2.397292	-1.575249	0.999292
C	3.627853	1.478964	0.982275	H	3.057545	-2.361968	-1.875708
C	4.074664	0.302330	1.425533	H	0.460863	-1.526370	0.557100
C	5.059325	-0.594175	0.696303	H	-1.716350	-1.583725	-1.633870
C	4.459025	-2.028556	0.571756	H	-1.858769	-2.398245	-0.088900
C	2.957210	-2.153762	0.257134	H	-1.895798	2.231980	1.180209
C	2.504369	-1.756107	-1.148924	H	-1.151446	3.143929	-0.121262
C	1.011883	-1.884237	-1.425814	H	-1.504738	-0.238831	1.094231
C	0.106606	-1.672430	-0.462063	H	-5.309345	1.775738	-1.290527
C	-1.383235	-1.545270	-0.593538	H	-3.419376	-1.976629	1.502673
C	-1.924106	-0.255559	0.077647	H	-4.428814	-0.005855	2.633490
C	-3.440037	-0.309751	0.181266	H	-5.843527	-0.143806	1.564928
C	-4.001081	0.688835	-0.530425	H	-6.216119	-1.364664	3.461854
C	-2.986623	1.566732	-1.108478	H	-0.942893	0.324153	-2.578109
C	-0.656521	1.081974	-1.844522	H	-0.707178	2.055547	-2.342104
C	1.560245	3.604325	-0.272531	H	0.374123	0.891931	-1.545404
C	6.374396	-0.666951	1.484415	H	-5.726271	-1.707174	-0.503953
C	0.692843	-2.079534	-2.883826	H	-5.524107	-3.027367	0.670843
C	-4.161017	-1.366661	0.970361	H	-4.352430	-2.817591	-0.652063
C	-5.037519	-0.713469	2.047987	H	-0.377046	-2.063132	-3.099416
C	-4.993244	-2.290647	0.062516	H	1.177543	-1.295397	-3.478436
O	-3.259301	2.543138	-1.797351	H	1.089509	-3.040650	-3.237578
O	2.875354	-0.397086	-1.495070	H	2.487677	0.176934	-0.809732
O	-5.310354	0.968879	-0.734864	H	0.672395	3.761372	-0.887175
O	-5.551047	-1.752726	2.874557	H	1.854022	4.568893	0.162673
O	5.414713	-0.071670	-0.575391	H	2.375678	3.304645	-0.943494
H	2.268416	1.795624	2.600519	H	6.851951	0.316765	1.483948
H	3.005426	3.272780	1.976911	H	7.053392	-1.383775	1.010253
H	4.034378	1.863766	0.048138	H	6.202672	-0.975817	2.521003
H	0.195477	1.201124	1.851846	H	4.608475	-0.129801	-1.131075

Table S37. Atomic coordinates (Å) of **6b-5** obtained at the B3LYP-D3BJ/6-31G(d) level of theory in the gas phase.

C	-2.683494	0.634096	-1.159724	H	1.176351	1.402472	0.622412
C	-2.410159	2.152217	-0.896945	H	2.068111	-0.833491	1.067899
C	-1.044326	2.569808	-1.364754	H	3.726556	-1.101161	1.575850
C	-0.114605	3.250389	-0.677094	H	3.204018	-0.264768	-1.307096
C	1.267881	3.455036	-1.259790	H	4.577410	-1.120160	-0.653002
C	2.366203	2.667235	-0.572171	H	3.140121	-3.101977	-0.157270
C	2.179171	1.697432	0.323680	H	1.196302	-0.351268	-1.344837
C	3.262839	0.953659	1.064824	H	-0.874684	-0.679673	-2.666283
C	3.106450	-0.568182	0.840260	H	-1.311535	-2.109737	-1.749030
C	3.490390	-1.027145	-0.573004	H	-2.519150	2.350584	0.174711
C	2.893210	-2.384166	-0.951199	H	-3.205248	2.724364	-1.392870
C	1.385218	-2.423587	-1.170148	H	-0.700760	0.458786	-0.294633
C	0.677793	-1.306788	-1.389319	H	-5.020725	-0.733354	1.522755
C	-0.788334	-1.151429	-1.680625	H	-0.158579	-1.927532	0.960303
C	-1.481804	-0.227820	-0.638001	H	-1.741434	-0.895018	3.351636
C	-2.008686	-0.974832	0.575529	H	-0.087907	-1.543674	3.363463
C	-3.320460	-0.710328	0.761181	H	-0.919098	0.834916	2.101470
C	-3.833269	0.213975	-0.250437	H	-2.363766	0.830704	-3.319597
C	-3.099075	0.419234	-2.620831	H	-3.231953	-0.642082	-2.852619
C	-0.328227	3.862014	0.685518	H	-4.054960	0.923480	-2.792426
C	3.137619	1.277739	2.563247	H	-1.851660	-3.755462	0.891472
C	0.812326	-3.817226	-1.168489	H	-2.666337	-3.082511	2.311589
C	-1.114937	-1.790547	1.471245	H	-1.016786	-3.729964	2.453813
C	-0.820480	-0.998430	2.760590	H	-0.227985	-3.856912	-1.499074
C	-1.701306	-3.171380	1.804799	H	1.409456	-4.464159	-1.820478
O	-4.998858	0.591210	-0.282992	H	0.860453	-4.249795	-0.159166
O	3.547808	-2.929801	-2.111516	H	3.295419	-2.357586	-2.854741
O	-4.157213	-1.145614	1.733633	H	-1.270804	3.564670	1.151929
O	-0.235748	0.278274	2.506346	H	0.491410	3.602575	1.363324
O	4.528073	1.408011	0.568348	H	-0.337634	4.957720	0.605957
H	1.536830	4.521891	-1.209116	H	3.899450	0.733222	3.137776
H	1.262406	3.204155	-2.330171	H	2.151608	0.991211	2.941971
H	3.385243	2.940406	-0.836335	H	3.281748	2.350853	2.722199
H	-0.766808	2.216693	-2.358882	H	5.212793	0.969087	1.096495

S4. *Computational data of compound 8*

Table S38. Experimental and calculated NMR data of **8a** and **8b**.

No.	**8a**		**8b**		**8**	
	$\delta_{calcd.\ H}$	$\delta_{calcd.\ C}$	$\delta_{calcd.\ H}$	$\delta_{calcd.\ C}$	$\delta_{exptl.\ H}$	$\delta_{exptl.\ C}$
1		51.2		54.4		49.0
2	2.40	39.5	2.21	42.8	2.31	40.3
	2.29		1.42		1.72	
3	4.94	123.1	5.55	121.3	5.11	123.7
4		137.3		143.9		137.1
5	2.04	37.9	2.08	35.2	2.09	36.3
	1.92		2.00		1.87	
6	1.56	35.6	1.63	41.9	1.75	30.4
	1.44		1.37		1.66	
7	4.36	69.0	4.52	69.1	3.84	77.5
8		142.8		140.9		138.6
9	5.44	119.1	5.81	122.1	5.11	125.3
10	2.22	40.9	1.95	35.2	2.59	33.5
	2.12		1.94		2.24	
11	4.04	77.4	3.77	83.4	3.90	79.7
12		145.1		139.7		137.3
13	6.13	120.7	5.58	125.6	5.46	130.8
14	2.52	28.6	2.46	32.7	2.44	30.3
	1.83		1.81		2.07	
15	2.52	54.6	2.75	53.5	2.65	51.7
16		154.4		151.8		151.9
17		144.4		143.4		149.7
18		204.1		205.2		210.3
19	1.07	22.0	0.81	17.4	0.98	17.1
20	1.57	17.0	1.83	19.7	1.62	15.4
21	1.69	19.9	1.68	19.5	1.67	9.9
22	1.73	20.7	1.54	12.1	1.67	10.6
23	3.00	39.9	2.66	41.9	2.61	38.8
24	4.11	66.7	3.98	67.3	3.81	65.9
	3.71		3.63		3.70	
25	1.12	15.6	1.11	14.3	1.27	14.7

Table S39. Experimental and calculated NMR data of **8c** and **8d**.

No.	**8a**		**8b**		**8**	
	$\delta_{calcd. H}$	$\delta_{calcd. C}$	$\delta_{calcd. H}$	$\delta_{calcd. C}$	$\delta_{exptl. H}$	$\delta_{exptl. C}$
1		52.6		53.5		49.0
2	2.21	42.5	2.10	38.7	2.31	40.3
	1.55		1.85		1.72	
3	5.41	122.8	5.64	123.8	5.11	123.7
4		142.6		144.4		137.1
5	2.04	37.0	2.08	35.9	2.09	36.3
	1.97		1.88		1.87	
6	2.03	29.2	1.93	29.1	1.75	30.4
	1.62		1.61		1.66	
7	4.07	78.1	4.16	80.2	3.84	77.5
8		141.0		139.4		138.6
9	5.60	117.6	5.63	119.1	5.11	125.3
10	2.47	35.7	2.46	34.3	2.59	33.5
	2.24		2.44		2.24	
11	3.80	80.7	4.21	76.1	3.90	79.7
12		138.6		138.7		137.3
13	5.77	129.9	6.14	124.2	5.46	130.8
14	2.35	32.6	2.52	30.9	2.44	30.3
	1.99		1.83		2.07	
15	2.64	54.4	2.66	53.4	2.65	51.7
16		153.1		153.8		151.9
17		144.0		141.8		149.7
18		204.4		204.3		210.3
19	0.83	17.5	1.10	18.7	0.98	17.1
20	1.74	17.1	1.65	17.9	1.62	15.4
21	1.57	15.7	1.50	16.2	1.67	9.9
22	1.71	12.5	1.74	15.1	1.67	10.6
23	2.62	41.4	2.87	40.3	2.61	38.8
24	3.93	68.6	3.66	69.2	3.81	65.9
	3.68		3.59		3.70	
25	1.17	15.0	1.23	16.2	1.27	14.7

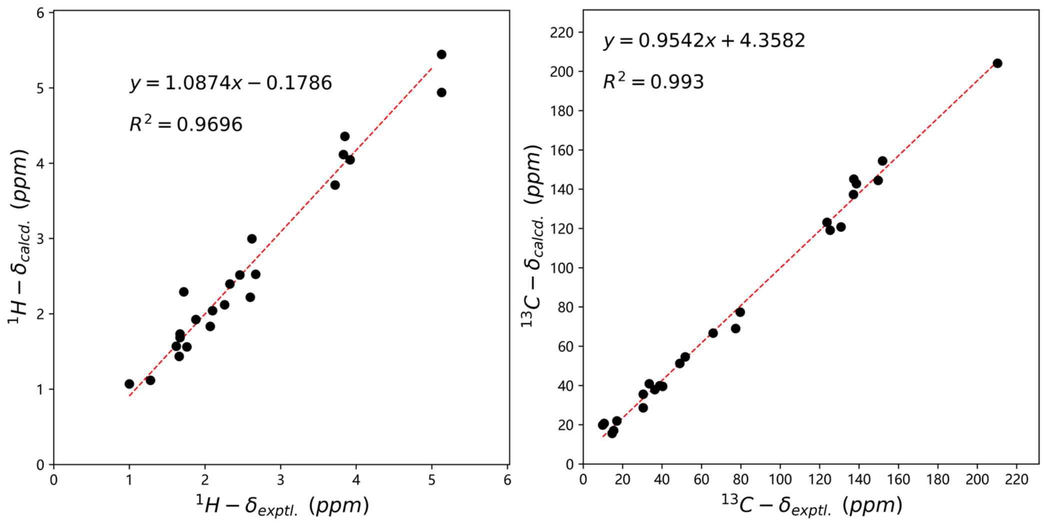

Figure S10. Linear regression analyses between experimental and calculated NMR data of **8a**.

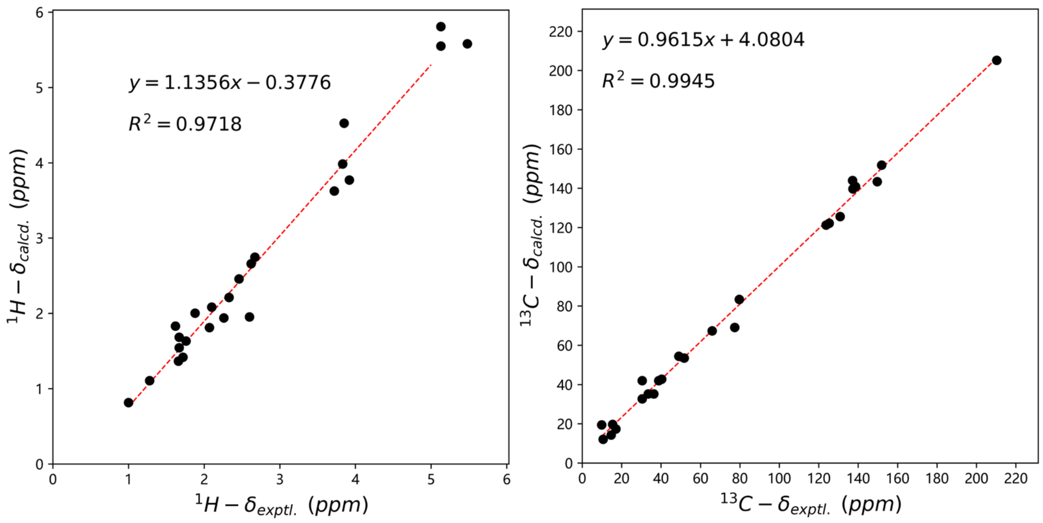

Figure S11. Linear regression analyses between experimental and calculated NMR data of **8b**.

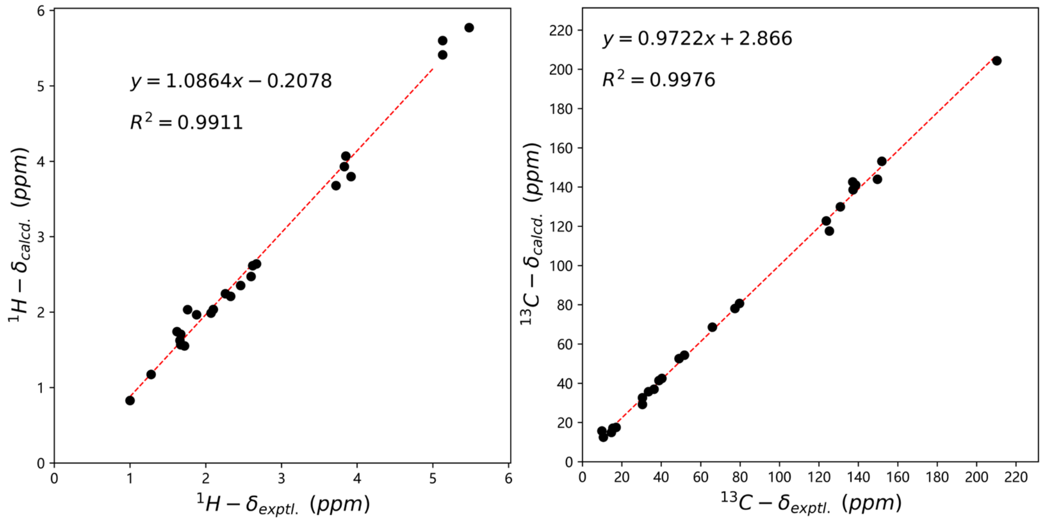

Figure S12. Linear regression analyses between experimental and calculated NMR data of **8c**.

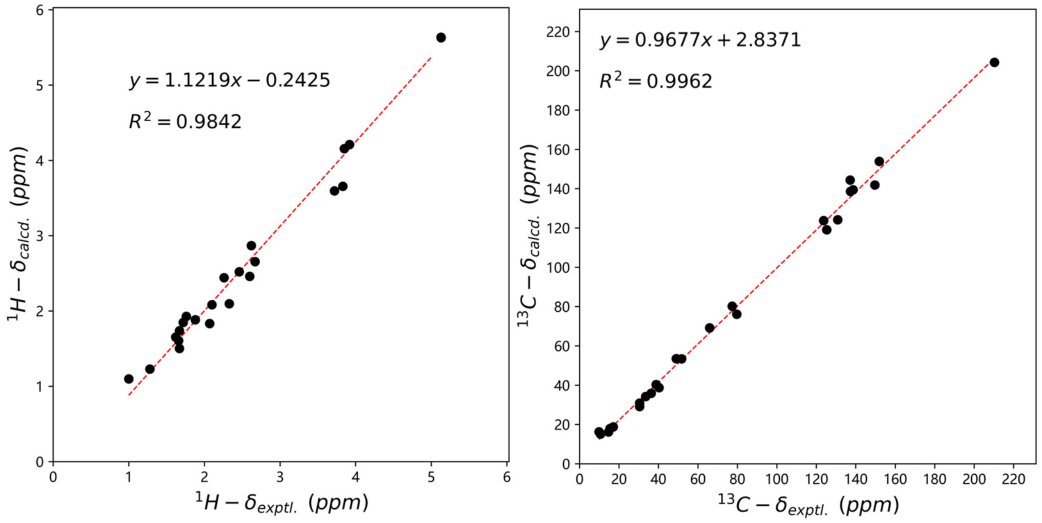

Figure S13. Linear regression analyses between experimental and calculated NMR data of **8d**.

Functional	Solvent?	Basis Set	Type of Data		
mPW1PW91	PCM	6-31+G(d,p)	Unscaled Shifts		

	Isomer 1	Isomer 2	Isomer 3	Isomer 4	Isomer 5	Isomer 6
sDP4+ (H data)	0.00%	0.00%	97.28%	2.71%	–	–
sDP4+ (C data)	0.00%	0.00%	100.00%	0.00%	–	–
sDP4+ (all data)	0.00%	0.00%	100.00%	0.00%	–	–
uDP4+ (H data)	0.00%	0.00%	99.58%	0.42%	–	–
uDP4+ (C data)	0.00%	0.00%	100.00%	0.00%	–	–
uDP4+ (all data)	0.00%	0.00%	100.00%	0.00%	–	–
DP4+ (H data)	0.00%	0.00%	99.99%	0.01%	–	–
DP4+ (C data)	0.00%	0.00%	100.00%	0.00%	–	–
DP4+ (all data)	0.00%	0.00%	100.00%	0.00%	–	–

Figure S14. DP4+ analysis results of **8a** (1st column), **8b** (2nd column), **8c** (3rd column) and **8d** (4th column).

Table S40 Conformational analysis of the B3LYP-D3BJ/6-31G(d) optimized conformers of **8a** in the gas phase (T=298.15 K).

Conformer	E (Hartree)[a]	C (Hartree)[b]	G (kcal/mol)[c]	ΔG (kcal/mol)[d]	Population[e]
**8a-1**	-1351.468003	0.527825	-847714.961864	0.0	50.13%
**8a-2**	-1351.466952	0.527422	-847714.555326	0.406538	25.23%
**8a-3**	-1351.466957	0.528317	-847713.996619	0.965246	9.82%
**8a-4**	-1351.466046	0.527545	-847713.909177	1.052688	8.47%
**8a-5**	-1351.465477	0.527248	-847713.738428	1.223437	6.35%

[a]Electronic energy obtained at the M06-2X-D3/def2-TZVP level of theory; [b]Thermal correction to Gibbs free energy obtained at the M06-2X-D3/def2-SVP level of theory; [c]Gibbs free energy (E + C); [d]The relative Gibbs free energy; [e]The Boltzmann distribution of each conformer.

Table S41. Atomic coordinates (Å) of **8a-1** obtained at the B3LYP-D3BJ/6-31G(d) level of theory in the gas phase.

C	-1.974956	1.638177	0.658615	H	-1.469837	-0.711085	1.907921
C	-1.287251	1.471604	2.048417	H	1.616053	-0.365434	0.624974
C	-0.810345	0.066773	2.291152	H	5.024180	-0.345889	-0.799226
C	0.350671	-0.334838	2.826440	H	2.892057	1.206918	0.812551
C	0.750159	-1.793763	2.771359	H	4.557847	1.667631	0.488072
C	2.029938	-2.020011	1.930984	H	3.980011	1.969542	-1.925972
C	1.977553	-1.393854	0.524140	H	0.804394	2.914076	-0.560220
C	3.345509	-1.371978	-0.129515	H	-1.292953	2.440350	-1.938626
C	4.054064	-0.248071	-0.309479	H	-0.568342	0.952892	-2.514738
C	3.682547	1.163298	0.059198	H	-2.020246	1.785658	2.804604
C	3.216520	2.029463	-1.131244	H	-0.449942	2.174677	2.101178
C	1.890039	1.546517	-1.700174	H	-0.414252	0.270222	-0.023265
C	0.743446	2.108416	-1.289858	H	-5.157860	-0.456362	0.312462
C	-0.630595	1.617530	-1.648960	H	-0.787161	-1.257303	-2.042387
C	-1.251006	0.819552	-0.468579	H	-3.191088	-2.931255	-1.201016
C	-2.251317	-0.227867	-0.919477	H	-1.609715	-3.482551	-1.798100
C	-3.423360	-0.087280	-0.265388	H	-2.149751	-2.263603	0.681088
C	-3.358263	0.989577	0.719218	H	-1.210556	3.655132	0.310390
C	-2.171460	3.136908	0.383185	H	-2.729576	3.312178	-0.541734
C	1.354289	0.599263	3.453329	H	-2.744209	3.579720	1.203788
C	3.872475	-2.708693	-0.584739	H	-2.320019	-2.062164	-3.869164
C	1.952440	0.398463	-2.683315	H	-2.380591	-0.297337	-3.716274
C	-1.865151	-1.332517	-1.864337	H	-3.693339	-1.284177	-3.051631
C	-2.117633	-2.709248	-1.216227	H	1.305301	0.576814	-3.549960
C	-2.610483	-1.238258	-3.206832	H	2.971373	0.254115	-3.054441
O	-4.282847	1.257743	1.478065	H	1.643707	-0.544055	-2.218112
O	3.152617	3.359580	-0.621837	H	2.739917	3.903202	-1.311942
O	-4.546780	-0.844062	-0.348335	H	2.300918	0.610948	2.899898
O	-1.586443	-2.811159	0.111983	H	0.992673	1.627238	3.519743
O	1.107185	-2.108110	-0.358731	H	1.594047	0.263641	4.471199
H	0.937610	-2.180786	3.783080	H	4.844720	-2.606890	-1.076329
H	-0.072768	-2.387796	2.357011	H	3.989744	-3.401963	0.258481
H	2.214803	-3.096202	1.839080	H	3.167867	-3.177727	-1.278994
H	2.890865	-1.594400	2.459876	H	0.237275	-2.264878	0.060115

Table S42. Atomic coordinates (Å) of **8a-2** obtained at the B3LYP-D3BJ/6-31G(d) level of theory in the gas phase.

C	-1.984147	1.592330	0.719417	H	-1.423950	-0.827827	1.849675
C	-1.298298	1.358189	2.100931	H	1.643711	-0.382630	0.571465
C	-0.791852	-0.048084	2.273535	H	5.064556	-0.206843	-0.822037
C	0.380938	-0.428289	2.801131	H	2.872218	1.234039	0.811826
C	0.829470	-1.870425	2.699305	H	4.518890	1.764482	0.494930
C	2.123984	-2.028779	1.867341	H	3.923457	2.067247	-1.921315
C	2.053294	-1.392187	0.465736	H	0.744624	2.922696	-0.514807
C	3.422472	-1.306883	-0.179488	H	-1.354085	2.437928	-1.882608
C	4.089292	-0.154558	-0.335474	H	-0.613428	0.968385	-2.485601
C	3.663241	1.234376	0.057618	H	-2.039993	1.617917	2.869460
C	3.165751	2.101135	-1.119412	H	-0.476761	2.075251	2.196311
C	1.845483	1.594111	-1.682886	H	-0.440951	0.232567	-0.019308
C	0.691234	2.126606	-1.255039	H	-5.217567	-0.402136	0.307317
C	-0.678090	1.620132	-1.609536	H	-0.866980	-1.269385	-2.060000
C	-1.281597	0.796749	-0.437664	H	-3.266197	-2.831487	-1.020252
C	-2.301477	-0.224050	-0.899773	H	-1.837069	-3.487284	-1.869646
C	-3.475890	-0.063714	-0.258595	H	-1.620512	-3.610288	0.448774
C	-3.384015	0.978430	0.757169	H	-1.175043	3.605575	0.450545
C	-2.146395	3.104990	0.502927	H	-2.701491	3.330139	-0.413090
C	1.354871	0.511837	3.466813	H	-2.709346	3.526552	1.341407
C	4.002366	-2.614310	-0.655431	H	-2.440203	-2.046676	-3.855870
C	1.924768	0.460750	-2.681635	H	-2.497700	-0.283483	-3.680499
C	-1.941851	-1.330400	-1.852734	H	-3.795816	-1.276116	-2.999305
C	-2.193525	-2.693254	-1.199013	H	2.940710	0.353126	-3.073258
C	-2.716918	-1.230582	-3.178346	H	1.654175	-0.497411	-2.224159
O	-4.302376	1.249398	1.523628	H	1.256250	0.629760	-3.533784
O	3.075798	3.424596	-0.596437	H	2.633789	3.962120	-1.273046
O	-4.623006	-0.777638	-0.375115	H	2.287024	0.606684	2.896010
O	-1.484015	-2.738068	0.049956	H	0.947101	1.514710	3.607333
O	1.222764	-2.143155	-0.426442	H	1.635687	0.125506	4.455930
H	1.017816	-2.287749	3.699236	H	3.322623	-3.094994	-1.366440
H	0.026816	-2.469148	2.254097	H	4.974684	-2.466920	-1.135272
H	2.360850	-3.094070	1.763725	H	4.137592	-3.319408	0.175260
H	2.960199	-1.570441	2.408559	H	0.322356	-2.236178	-0.057666

Table S43. Atomic coordinates (Å) of **8a-3** obtained at the B3LYP-D3BJ/6-31G(d) level of theory in the gas phase.

C	1.943993	1.545930	0.731437	H	0.921106	1.619922	-1.805552
C	0.972847	2.608352	0.171040	H	-1.539706	0.182064	-0.650045
C	0.314781	2.209168	-1.117566	H	-4.529834	-1.852702	0.235351
C	-0.946038	2.492184	-1.470936	H	-3.258518	0.966917	0.341265
C	-1.531824	1.916212	-2.739321	H	-4.820303	0.503564	0.986895
C	-2.558793	0.803977	-2.430765	H	-3.786716	-0.664188	2.834625
C	-1.975078	-0.289972	-1.529166	H	-0.765100	1.167230	2.163257
C	-3.001585	-1.319371	-1.082393	H	1.348289	-0.205856	2.872176
C	-3.805842	-1.084304	-0.036158	H	0.698604	-1.541556	1.940029
C	-3.794293	0.148905	0.825381	H	1.565989	3.523645	0.027303
C	-3.140080	-0.018053	2.216579	H	0.216477	2.843401	0.927246
C	-1.765590	-0.664511	2.144824	H	0.677491	-0.009834	-0.081294
C	-0.653558	0.085107	2.124655	H	5.083641	0.671202	-1.181384
C	0.744405	-0.454185	1.990172	H	2.277786	-2.590078	1.241300
C	1.433903	0.061904	0.709420	H	1.971186	-2.512676	-1.779260
C	2.634689	-0.763248	0.230558	H	1.915610	-3.983304	-0.791680
C	3.560412	0.063846	-0.309126	H	-0.012228	-2.010359	-1.062538
C	3.195777	1.466324	-0.134167	H	1.573598	2.104283	2.814364
C	2.419664	1.995589	2.128331	H	3.126035	1.279205	2.559930
C	-1.872976	3.313797	-0.595992	H	2.923973	2.963590	2.046584
C	-3.060823	-2.607932	-1.860462	H	4.774176	-2.409908	0.825455
C	-1.751220	-2.178357	2.117003	H	4.481493	-2.679938	-0.890415
C	2.667037	-2.267761	0.266915	H	4.049840	-3.937548	0.287073
C	1.719927	-2.902951	-0.782401	H	-2.760424	-2.580341	1.993445
C	4.077999	-2.856599	0.110014	H	-1.134337	-2.566612	1.301594
O	3.860929	2.393123	-0.586502	H	-1.354620	-2.581530	3.058742
O	-3.119804	1.302321	2.757021	H	-2.604033	1.260634	3.578136
O	4.716866	-0.210853	-0.961370	H	-2.031123	2.861353	0.391420
O	0.346321	-2.720084	-0.490845	H	-1.471129	4.319410	-0.424228
O	-0.826134	-0.890144	-2.168852	H	-2.857451	3.432197	-1.059696
H	-2.031478	2.697985	-3.327774	H	-2.128493	-3.174700	-1.742766
H	-0.731629	1.501669	-3.360023	H	-3.885362	-3.237674	-1.515381
H	-2.925882	0.358034	-3.366569	H	-3.208848	-2.434187	-2.937077
H	-3.437825	1.224825	-1.930194	H	-1.122091	-1.327351	-2.983179

Table S44. Atomic coordinates (Å) of **8a-4** obtained at the B3LYP-D3BJ/6-31G(d) level of theory in the gas phase.

C	-2.021350	1.536874	0.734503	H	-1.291415	-0.864960	1.793725
C	-1.255601	1.326572	2.074403	H	1.672852	-0.437150	0.472891
C	-0.694041	-0.062555	2.225804	H	5.087270	0.002875	-0.904828
C	0.486775	-0.399009	2.766005	H	2.798741	1.277131	0.740038
C	0.981898	-1.827544	2.680288	H	4.404686	1.921018	0.427676
C	2.296592	-1.942565	1.876135	H	3.786931	2.147332	-2.007181
C	2.198772	-1.390628	0.447034	H	0.610231	2.925802	-0.571409
C	3.549208	-1.216907	-0.220730	H	-1.489523	2.400753	-1.890813
C	4.117602	-0.017595	-0.405160	H	-0.742998	0.943349	-2.517007
C	3.588784	1.334466	-0.013039	H	-1.963209	1.561713	2.882120
C	3.041017	2.166175	-1.193509	H	-0.454521	2.071268	2.129014
C	1.729040	1.608702	-1.730484	H	-0.521574	0.176342	-0.074514
C	0.565471	2.118001	-1.299260	H	-5.249339	-0.498431	0.478752
C	-0.799796	1.588951	-1.635488	H	-1.060231	-1.226025	-2.208454
C	-1.371521	0.752360	-0.458904	H	-3.163439	-2.940316	-0.813429
C	-2.413033	-0.258214	-0.895139	H	-1.797533	-3.510333	-1.793637
C	-3.551191	-0.117769	-0.185714	H	-0.455358	-2.463885	-0.113923
C	-3.407541	0.899172	0.847350	H	-1.279202	3.572508	0.421649
C	-2.232416	3.044223	0.518972	H	-2.834466	3.248442	-0.372130
C	1.414565	0.577150	3.447538	H	-2.768862	3.454450	1.380183
C	4.231370	-2.481649	-0.680715	H	-4.069469	-1.502455	-2.752757
C	1.830817	0.464485	-2.715049	H	-2.780048	-2.233191	-3.733167
C	-2.090524	-1.367585	-1.853685	H	-2.945183	-0.468991	-3.650015
C	-2.131898	-2.718544	-1.101534	H	1.615508	-0.501008	-2.243411
C	-3.028197	-1.394922	-3.071460	H	1.130937	0.588978	-3.548679
O	-4.275299	1.144219	1.678902	H	2.839119	0.396296	-3.135273
O	2.916155	3.490646	-0.684279	H	2.440746	4.005383	-1.356015
O	-4.695725	-0.840057	-0.253328	H	1.693974	0.202305	4.441576
O	-1.375788	-2.722532	0.094756	H	2.353160	0.712989	2.894343
O	1.338053	-2.220557	-0.374093	H	0.965453	1.563088	3.581423
H	1.171093	-2.228908	3.686472	H	5.219892	-2.269639	-1.097028
H	0.213000	-2.457105	2.221632	H	4.363247	-3.196923	0.142547
H	2.615543	-2.994165	1.841295	H	3.646410	-2.988238	-1.459854
H	3.099372	-1.402274	2.390679	H	1.670212	-3.131632	-0.327118

Table S45. Atomic coordinates (Å) of **8a-5** obtained at the B3LYP-D3BJ/6-31G(d) level of theory in the gas phase.

C	-1.902943	-0.304260	-1.601318	H	0.383167	0.954910	-2.559195
C	-1.674953	1.228588	-1.824841	H	2.124622	0.540114	-0.636853
C	-0.233562	1.624269	-1.960465	H	4.993840	-0.844973	1.186963
C	0.371233	2.712873	-1.451113	H	3.371222	-0.742983	-1.442588
C	1.856441	2.913338	-1.673930	H	4.870560	-1.599550	-1.129685
C	2.723435	2.598484	-0.431886	H	3.690678	-3.205001	0.312480
C	2.397297	1.220932	0.173746	H	0.801257	-2.541548	-1.700905
C	3.538599	0.624433	0.960063	H	-1.450766	-2.854538	-0.693747
C	4.184624	-0.477611	0.554262	H	-0.819988	-2.510155	0.898731
C	3.916910	-1.300843	-0.677105	H	-2.136858	1.770094	-0.994175
C	3.125760	-2.601206	-0.419800	H	-2.232158	1.505972	-2.730920
C	1.736114	-2.367353	0.157635	H	-0.448054	-0.088386	0.035250
C	0.652205	-2.389923	-0.634416	H	-5.428055	-0.292074	-0.157770
C	-0.758986	-2.200476	-0.149961	H	-1.113814	-0.802410	2.424082
C	-1.284798	-0.735360	-0.232906	H	-3.215750	1.405364	2.481596
C	-2.410472	-0.551279	0.768107	H	-2.057122	1.065311	3.781117
C	-3.593705	-0.460571	0.126740	H	-0.401193	1.627898	2.273009
C	-3.402781	-0.421155	-1.325645	H	-1.698619	-2.167870	-2.724541
C	-1.501998	-1.098970	-2.844895	H	-2.092076	-0.753095	-3.699231
C	-0.317375	3.783996	-0.640564	H	-0.441499	-0.978586	-3.082214
C	3.906902	1.341601	2.232255	H	-2.852936	-1.247010	4.149339
C	1.660631	-2.134996	1.650424	H	-3.013233	-2.382981	2.796390
C	-2.143699	-0.463208	2.243356	H	-4.135204	-1.024898	2.937727
C	-2.216531	1.012675	2.692781	H	0.902109	-2.773427	2.119563
C	-3.095259	-1.329789	3.083613	H	2.618180	-2.346869	2.134579
O	-4.326998	-0.377967	-2.128608	H	1.408571	-1.092691	1.872968
O	3.101291	-3.267498	-1.678396	H	2.518298	-4.036813	-1.576712
O	-4.848381	-0.353143	0.629088	H	0.290592	4.068277	0.225995
O	-1.306450	1.860968	2.005338	H	-0.448929	4.689479	-1.248394
O	1.257067	1.321926	1.055049	H	-1.294402	3.478486	-0.263248
H	2.186976	2.265533	-2.494785	H	4.719984	0.831503	2.757271
H	2.052907	3.947651	-1.987745	H	4.225279	2.373186	2.032339
H	2.586204	3.358049	0.345995	H	3.041855	1.405205	2.902036
H	3.778579	2.630851	-0.729793	H	0.504993	1.548569	0.480490

Table S46. Conformational analysis of the B3LYP-D3BJ/6-31G(d) optimized conformers of **8b** in the gas phase (T=298.15 K).

Conformer	E (Hartree)[a]	C (Hartree)[b]	G (kcal/mol)[c]	ΔG (kcal/mol)[d]	Population[e]
**8b-1**	-1351.45852	0.522386	-847712.423947	0.0	77.21%
**8b-2**	-1351.45834	0.524153	-847711.202317	1.22163	9.81%
**8b-3**	-1351.455288	0.52144	-847710.989714	1.434233	6.85%
**8b-4**	-1351.45748	0.523737	-847710.923996	1.499951	6.13%

[a]Electronic energy obtained at the M06-2X-D3/def2-TZVP level of theory; [b]Thermal correction to Gibbs free energy obtained at the M06-2X-D3/def2-SVP level of theory; [c]Gibbs free energy (E + C); [d]The relative Gibbs free energy; [e]The Boltzmann distribution of each conformer.

Table S47. Atomic coordinates (Å) of **8b-1** obtained at the B3LYP-D3BJ/6-31G(d) level of theory in the gas phase.

C	-1.508096	-0.669394	0.838967	H	0.021931	-1.488231	-1.602688
C	-1.104748	-2.041250	0.205087	H	3.331875	-0.929244	0.780133
C	0.129657	-2.009104	-0.650158	H	5.250944	2.234363	0.131675
C	1.315583	-2.583849	-0.395967	H	2.649798	0.906287	1.136769
C	2.425620	-2.506009	-1.430541	H	3.308738	2.462318	1.640481
C	3.291443	-1.233810	-1.347312	H	2.385201	1.911466	-1.201882
C	4.058468	-1.046563	-0.027068	H	0.281244	1.359965	-1.399851
C	4.961846	0.177065	-0.094212	H	-1.737406	2.183855	0.796311
C	4.558814	1.404418	0.263051	H	-2.085191	2.364464	-0.913441
C	3.202965	1.782384	0.786259	H	-1.949254	-2.383324	-0.412322
C	2.338377	2.496056	-0.270681	H	-1.005699	-2.764303	1.019123
C	0.891168	2.595052	0.164733	H	-1.708752	-0.061746	-1.266595
C	-0.040369	1.914661	-0.516176	H	-5.049161	-1.141499	2.186132
C	-1.499993	1.776696	-0.190744	H	-3.760249	1.342868	-2.012501
C	-2.005052	0.314893	-0.277889	H	-5.961432	-0.620165	-1.242209
C	-3.522507	0.279499	-0.194111	H	-5.767376	0.225967	-2.790486
C	-3.920982	-0.385438	0.910322	H	-4.037041	-1.726223	-1.740355
C	-2.780407	-0.942117	1.638093	H	-0.615343	0.786731	2.210552
C	-0.398620	-0.194973	1.781386	H	-0.316207	-0.908432	2.607549
C	1.638656	-3.368512	0.851847	H	0.559369	-0.143439	1.262219
C	6.347858	-0.062712	-0.637812	H	-5.963715	2.404347	-1.454166
C	0.632537	3.411993	1.404398	H	-4.743645	2.796254	-0.228008
C	-4.404507	0.895141	-1.245501	H	-5.971653	1.567067	0.113314
C	-5.223511	-0.208436	-1.945587	H	1.248285	4.316817	1.386175
C	-5.327399	1.981345	-0.668097	H	-0.414195	3.708116	1.503376
O	-2.898333	-1.579695	2.677810	H	0.909653	2.854033	2.309107
O	2.937031	3.780863	-0.476194	H	2.423000	4.226850	-1.167576
O	-5.174014	-0.612530	1.370869	H	2.647527	-3.134032	1.205068
O	-4.400953	-1.231504	-2.491444	H	0.931443	-3.192908	1.665329

O	4.780506	-2.229723	0.337132	H	1.635924	-4.445488	0.630459
H	3.074424	-3.383974	-1.331732	H	6.916422	-0.733841	0.017178
H	1.991247	-2.544367	-2.436481	H	6.320226	-0.530986	-1.633088
H	2.656541	-0.353046	-1.497061	H	6.902521	0.874798	-0.731766
H	4.011788	-1.239969	-2.179056	H	5.333505	-2.482842	-0.418811

Table S48. Atomic coordinates (Å) of **8b-2** obtained at the B3LYP-D3BJ/6-31G(d) level of theory in the gas phase.

C	-1.426594	-0.806844	0.702200	H	0.174032	-1.427313	-1.753690
C	-0.973298	-2.124231	-0.008317	H	3.418975	-0.947131	0.719610
C	0.275947	-2.006570	-0.834431	H	5.279727	2.290022	0.305174
C	1.470025	-2.571105	-0.595558	H	2.692148	0.848765	1.178891
C	2.596596	-2.405019	-1.601629	H	3.299812	2.393139	1.774071
C	3.432792	-1.123137	-1.422517	H	2.444977	1.963836	-1.110588
C	4.165923	-1.000073	-0.075768	H	0.359859	1.367265	-1.373526
C	5.042813	0.244171	-0.048551	H	-1.720188	2.051055	0.813899
C	4.604054	1.439058	0.371218	H	-2.033120	2.311141	-0.891813
C	3.228648	1.755411	0.884509	H	-1.797562	-2.449727	-0.659564
C	2.366372	2.499931	-0.152774	H	-0.866677	-2.891873	0.762738
C	0.909124	2.543406	0.257583	H	-1.625580	-0.080918	-1.361479
C	0.007621	1.873969	-0.472850	H	-4.952037	-1.480731	1.973942
C	-1.455937	1.696052	-0.186300	H	-3.679706	1.227525	-2.098025
C	-1.938786	0.233992	-0.355737	H	-5.747637	0.434583	-2.855314
C	-3.460046	0.167505	-0.287789	H	-4.867075	-0.987084	-2.269018
C	-3.840851	-0.580940	0.765530	H	-6.222565	-0.743112	-0.334274
C	-2.700029	-1.167104	1.460417	H	0.616885	-0.248618	1.196539
C	-0.348129	-0.358563	1.692954	H	-0.601133	0.588739	2.176446
C	1.788024	-3.427460	0.605661	H	-0.259318	-1.118656	2.475714
C	6.445603	0.066724	-0.572582	H	-5.716696	1.786264	0.126496
C	0.606446	3.294494	1.528587	H	-5.616555	2.633093	-1.422717
C	-4.334679	0.879579	-1.286532	H	-4.304023	2.800276	-0.235828
C	-5.373515	-0.060187	-1.951984	H	-0.447423	3.567954	1.616213
C	-5.031675	2.103312	-0.662816	H	0.871850	2.697276	2.411499
O	-2.818881	-1.884171	2.447628	H	1.206030	4.209285	1.569546
O	2.936600	3.807142	-0.282860	H	2.423423	4.275750	-0.959763
O	-5.091424	-0.876492	1.214165	H	1.065260	-3.315455	1.416661
O	-6.514172	-0.328368	-1.165242	H	1.807716	-4.488295	0.317283
O	4.905921	-2.187645	0.233559	H	2.786912	-3.198467	0.989415
H	3.262564	-3.273830	-1.545192	H	6.450672	-0.343108	-1.593674
H	2.181790	-2.389401	-2.616497	H	6.980602	1.019860	-0.599081
H	2.782668	-0.247953	-1.534415	H	7.014813	-0.629104	0.055519

H	4.171409	-1.064648	-2.236027	H	5.482138	-2.382346	-0.522323

Table S49. Atomic coordinates (Å) of **8b-3** obtained at the B3LYP-D3BJ/6-31G(d) level of theory in the gas phase.

C	-1.501627	-0.663119	0.852839	H	0.012695	-1.515984	-1.577282
C	-1.106077	-2.047461	0.241017	H	3.332232	-0.969500	0.789625
C	0.123008	-2.031116	-0.621996	H	5.207888	2.214872	0.107251
C	1.307349	-2.610927	-0.372438	H	2.580301	0.861592	1.027382
C	2.411127	-2.539644	-1.414128	H	3.248474	2.355232	1.661047
C	3.276266	-1.266517	-1.339366	H	2.398164	2.026606	-1.249229
C	4.052081	-1.078973	-0.024606	H	0.317687	1.299887	-1.354313
C	4.944493	0.152771	-0.096672	H	-1.748641	2.188878	0.766928
C	4.524631	1.378370	0.245835	H	-2.066156	2.343736	-0.948992
C	3.158981	1.746138	0.748117	H	-1.954892	-2.397344	-0.365898
C	2.341377	2.551726	-0.290808	H	-1.004094	-2.757017	1.066521
C	0.883170	2.656041	0.119756	H	-1.692282	-0.084757	-1.262422
C	-0.026827	1.907269	-0.515341	H	-5.046519	-1.101645	2.201879
C	-1.491259	1.767144	-0.209376	H	-3.741211	1.292918	-2.044001
C	-1.993463	0.304543	-0.280304	H	-5.959317	-0.633250	-1.229329
C	-3.511127	0.269717	-0.202074	H	-5.756424	0.174240	-2.797014
C	-3.913425	-0.372938	0.914238	H	-4.043561	-1.767254	-1.699422
C	-2.775449	-0.915835	1.656197	H	-0.312175	-0.875896	2.626384
C	-0.388172	-0.179101	1.785385	H	0.569981	-0.148302	1.265005
C	1.634831	-3.391998	0.876438	H	-0.593679	0.814429	2.192604
C	6.335013	-0.076149	-0.632141	H	-5.936626	2.383324	-1.509390
C	0.588220	3.571272	1.278574	H	-4.712927	2.794069	-0.293363
C	-4.388813	0.867490	-1.267261	H	-5.949968	1.582008	0.076782
C	-5.217188	-0.245062	-1.941621	H	0.900043	4.596419	1.041556
C	-5.303062	1.973612	-0.714143	H	-0.473131	3.604913	1.530736
O	-2.895773	-1.530481	2.709453	H	1.135329	3.266806	2.181097
O	2.947151	3.822346	-0.542820	H	3.082964	4.255409	0.314682
O	-5.168190	-0.588787	1.375999	H	2.647245	-3.161801	1.222462
O	-4.403853	-1.288317	-2.462378	H	0.933769	-3.209297	1.693758
O	4.786127	-2.256899	0.330147	H	1.624697	-4.469842	0.659668
H	3.060687	-3.417037	-1.315409	H	6.905123	-0.741501	0.027140
H	1.971207	-2.581731	-2.417401	H	6.315157	-0.547198	-1.626158
H	2.639527	-0.386260	-1.485642	H	6.882100	0.865706	-0.726919
H	3.991274	-1.271838	-2.175683	H	5.327010	-2.510004	-0.434561

Table S50. Atomic coordinates (Å) of **8b-4** obtained at the B3LYP-D3BJ/6-31G(d) level of theory in the gas phase.

C	1.460209	-1.074334	-0.568943	H	-0.252490	-1.516677	1.847135
C	0.895226	-2.338327	0.157198	H	-3.360221	-0.834307	-0.774898
C	-0.368169	-2.107616	0.936940	H	-4.967355	2.552402	-0.527399
C	-1.596671	-2.573577	0.662908	H	-2.474087	0.883510	-1.260289
C	-2.742021	-2.283086	1.618039	H	-2.941576	2.443869	-1.935304
C	-3.459417	-0.941542	1.369738	H	-2.222017	2.071204	0.993210
C	-4.134777	-0.801409	-0.005403	H	-0.211475	1.315121	1.355657
C	-4.905850	0.507961	-0.100387	H	2.015934	1.735406	-0.757527
C	-4.360026	1.649040	-0.543783	H	2.264509	2.037975	0.946395
C	-2.948715	1.839205	-1.020196	H	1.673970	-2.702815	0.842594
C	-2.066618	2.558261	0.018527	H	0.757218	-3.116986	-0.597853
C	-0.596828	2.469064	-0.335642	H	1.623190	-0.296517	1.480498
C	0.217514	1.755927	0.453286	H	5.011105	-1.942840	-1.745950
C	1.670726	1.444717	0.238211	H	5.349131	-0.378631	1.483549
C	2.016132	-0.046633	0.486225	H	4.421233	2.477433	0.970492
C	3.522022	-0.255027	0.470479	H	6.008708	1.903142	1.498572
C	3.878587	-1.022627	-0.575327	H	5.870194	0.725716	-0.682608
C	2.721993	-1.535841	-1.296874	H	0.318539	-1.344222	-2.363902
C	0.440942	-0.573394	-1.596252	H	-0.526128	-0.383820	-1.129123
C	-1.941704	-3.429715	-0.530980	H	0.773716	0.341075	-2.093754
C	-6.333977	0.461524	0.381959	H	3.148165	1.261261	2.865398
C	-0.185608	3.142542	-1.619177	H	4.766696	0.938497	3.500185
C	4.519989	0.342008	1.425132	H	3.624682	-0.395021	3.272988
C	5.135574	1.652813	0.872754	H	0.892385	3.306063	-1.682545
C	3.979564	0.548461	2.845817	H	-0.485240	2.544210	-2.490363
O	2.805468	-2.263989	-2.279291	H	-0.689932	4.109971	-1.708717
O	-2.535723	3.910384	0.074622	H	-2.006321	4.367251	0.747040
O	5.134619	-1.360292	-0.968802	H	-2.067454	-4.476703	-0.219477
O	5.475684	1.594512	-0.498594	H	-2.900034	-3.124473	-0.962040
O	-4.959657	-1.934129	-0.306780	H	-1.179703	-3.403194	-1.312772
H	-3.476908	-3.094418	1.560071	H	-6.938410	-0.207321	-0.242583
H	-2.365451	-2.269680	2.647795	H	-6.404026	0.088645	1.414778
H	-2.740763	-0.121040	1.477878	H	-6.790189	1.454909	0.360329
H	-4.216845	-0.794082	2.154231	H	-5.574460	-2.057173	0.433596

Table S51. Conformational analysis of the B3LYP-D3BJ/6-31G(d) optimized conformers of **8c** in the gas phase (T=298.15 K).

Conformer	E (Hartree)[a]	C (Hartree)[b]	G (kcal/mol)[c]	ΔG (kcal/mol)[d]	Population[e]
**8c-1**	-1351.462461	0.523593	-847714.139526	0.0	42.90%
**8c-2**	-1351.463119	0.524603	-847713.918671	0.220855	29.54%
**8c-3**	-1351.462615	0.524557	-847713.631414	0.508112	18.19%
**8c-4**	-1351.46279	0.525358	-847713.238643	0.900883	9.37%

[a]Electronic energy obtained at the M06-2X-D3/def2-TZVP level of theory; [b]Thermal correction to Gibbs free energy obtained at the M06-2X-D3/def2-SVP level of theory; [c]Gibbs free energy (E + C); [d]The relative Gibbs free energy; [e]The Boltzmann distribution of each conformer.

Table S52. Atomic coordinates (Å) of **8c-1** obtained at the B3LYP-D3BJ/6-31G(d) level of theory in the gas phase.

C	-1.321425	-0.721965	0.769447	H	0.648989	-0.753282	-1.152886
C	-0.817846	-1.958313	-0.027908	H	5.176359	-1.688092	0.933816
C	0.506847	-1.736584	-0.704751	H	2.880758	0.614947	1.376301
C	1.510143	-2.622482	-0.819237	H	4.462350	2.715712	-0.238336
C	2.780817	-2.254467	-1.560382	H	3.600702	2.975415	1.270185
C	4.076410	-2.428748	-0.745216	H	2.327251	2.401884	-1.442411
C	4.223288	-1.450654	0.438841	H	0.231213	1.754051	-1.459997
C	4.307263	0.008984	-0.000289	H	-1.522695	2.084432	1.075184
C	3.556950	0.947177	0.592872	H	-2.041490	2.486235	-0.547898
C	3.565447	2.423736	0.322525	H	-1.584526	-2.207164	-0.779375
C	2.342355	2.925918	-0.474108	H	-0.771979	-2.805290	0.662117
C	1.017993	2.693340	0.223088	H	-1.575713	0.112588	-1.248046
C	0.028937	2.078435	-0.437629	H	-4.838521	-1.513108	2.033083
C	-1.377673	1.822005	0.024392	H	-3.666287	1.577241	-1.776516
C	-1.860597	0.372197	-0.217897	H	-5.821709	-0.511005	-1.240129
C	-3.375176	0.287238	-0.119877	H	-5.661673	0.531712	-2.667738
C	-3.743267	-0.542571	0.879006	H	-3.887089	-1.509678	-1.899100
C	-2.581905	-1.170815	1.505860	H	0.647296	0.051586	1.297412
C	-0.264718	-0.285867	1.791600	H	-0.626142	0.528932	2.425585
C	1.467872	-4.024662	-0.259813	H	-0.027421	-1.131425	2.444592
C	5.333875	0.286135	-1.071228	H	-4.642569	2.760942	0.199971
C	0.912819	3.239482	1.623015	H	-5.839792	1.470790	0.396948
C	-4.287718	1.018216	-1.065955	H	-5.878993	2.507621	-1.045815
C	-5.099995	0.000480	-1.892825	H	1.335818	4.248699	1.657888
C	-5.219233	1.998621	-0.333602	H	-0.118173	3.289831	1.978082
O	-2.670588	-1.971387	2.429279	H	1.477952	2.622207	2.334251
O	2.442261	4.341451	-0.680525	H	3.344941	4.527033	-0.984540
O	-4.985125	-0.871652	1.307116	H	0.460085	-4.343551	0.011917
O	-4.270290	-0.930160	-2.576299	H	1.860286	-4.740671	-0.992662

O	3.236544	-1.689855	1.434272	H	2.099575	-4.099734	0.633317
H	2.856913	-2.894790	-2.451526	H	4.989657	-0.049368	-2.058037
H	2.709935	-1.220496	-1.919055	H	5.577551	1.347537	-1.154399
H	4.933589	-2.311542	-1.417491	H	6.267552	-0.253033	-0.864415
H	4.136085	-3.446798	-0.346070	H	2.368705	-1.586570	1.005485

Table S53. Atomic coordinates (Å) of **8c-2** obtained at the B3LYP-D3BJ/6-31G(d) level of theory in the gas phase.

C	1.332267	-0.658002	-0.831525	H	-0.594132	-0.754706	1.128751
C	0.880372	-1.915220	-0.030678	H	-5.144817	-1.720024	-0.904491
C	-0.441533	-1.728392	0.662271	H	-2.893501	0.610751	-1.378021
C	-1.433449	-2.627331	0.774244	H	-4.456763	2.701395	0.286433
C	-2.697712	-2.286159	1.539709	H	-3.641858	2.960621	-1.243825
C	-4.005443	-2.465384	0.745184	H	-2.310508	2.384096	1.434624
C	-4.185185	-1.476606	-0.425236	H	-0.201875	1.798478	1.432755
C	-4.276745	-0.021598	0.028259	H	1.524778	2.143169	-1.118875
C	-3.553796	0.930843	-0.575865	H	2.064066	2.540336	0.499245
C	-3.581067	2.406891	-0.298681	H	1.665600	-2.135056	0.708219
C	-2.349758	2.909692	0.467505	H	0.846295	-2.760755	-0.723190
C	-1.030192	2.699541	-0.250693	H	1.568154	0.154528	1.191958
C	-0.012990	2.126227	0.407781	H	4.845186	-1.435992	-2.092309
C	1.391042	1.878712	-0.066982	H	3.679752	1.626320	1.739612
C	1.866546	0.424913	0.169187	H	5.394522	-0.882224	1.458713
C	3.378770	0.322469	0.089355	H	5.476343	0.272371	2.804744
C	3.751470	-0.470243	-0.934978	H	3.301364	-0.341620	3.268478
C	2.591417	-1.069193	-1.590773	H	-0.669133	0.062290	-1.311099
C	0.245408	-0.236279	-1.825192	H	0.569612	0.599679	-2.451992
C	-1.381770	-4.019978	0.192453	H	0.023175	-1.077603	-2.488935
C	-5.280286	0.237091	1.125133	H	6.058952	1.108616	-0.131138
C	-0.959800	3.223326	-1.661402	H	6.070115	2.176547	1.290088
C	4.279676	0.944366	1.117890	H	5.056616	2.565800	-0.112812
C	4.810541	-0.168253	2.045944	H	-1.447618	4.202167	-1.718072
C	5.438473	1.746357	0.504337	H	0.065412	3.329078	-2.022050
O	2.681138	-1.836104	-2.542454	H	-1.488645	2.556534	-2.354900
O	-2.577653	4.311526	0.678721	H	-1.784731	4.660179	1.116245
O	4.994018	-0.812544	-1.351249	H	-2.032283	-4.092241	-0.687310
O	3.768676	-0.926317	2.651979	H	-0.375082	-4.317212	-0.106388
O	-3.214826	-1.696868	-1.441500	H	-1.745714	-4.753548	0.922871
H	-2.751654	-2.939213	2.423440	H	-4.914603	-0.114243	2.098725
H	-2.634467	-1.256086	1.911206	H	-5.520238	1.297108	1.229647
H	-4.851744	-2.365261	1.433912	H	-6.217920	-0.299971	0.930106

H	-4.060660	-3.479679	0.335787	H	-2.340358	-1.588893	-1.027600

Table S54. Atomic coordinates (Å) of **8c-3** obtained at the B3LYP-D3BJ/6-31G(d) level of theory in the gas phase.

C	-1.290776	-0.669004	0.739528	H	0.685655	-0.733331	-1.172673
C	-0.809396	-1.915057	-0.057300	H	5.196780	-1.768061	0.907241
C	0.521672	-1.716047	-0.730123	H	2.944060	0.571735	1.367824
C	1.508706	-2.619697	-0.847857	H	4.546504	2.674799	-0.243844
C	2.786822	-2.272476	-1.586631	H	3.704690	2.921995	1.273798
C	4.079878	-2.477115	-0.774248	H	2.419352	2.370813	-1.432749
C	4.247599	-1.509435	0.415522	H	0.305406	1.792988	-1.470210
C	4.356842	-0.048869	-0.014625	H	-1.456674	2.133417	1.057552
C	3.623284	0.898655	0.584311	H	-1.964682	2.555893	-0.564070
C	3.659798	2.377152	0.322642	H	-1.581571	-2.152119	-0.805291
C	2.443669	2.889273	-0.461359	H	-0.777239	-2.760305	0.635643
C	1.111541	2.678263	0.233273	H	-1.501754	0.175445	-1.276533
C	0.102965	2.113544	-0.445713	H	-4.826744	-1.440497	1.954959
C	-1.311044	1.878383	0.005036	H	-3.636928	1.784262	-1.720214
C	-1.807647	0.434510	-0.251887	H	-5.534835	0.607917	-2.796856
C	-3.324157	0.359340	-0.172397	H	-3.995723	-0.268805	-2.837707
C	-3.706269	-0.457552	0.827954	H	-5.766824	-0.908214	-0.766983
C	-2.562265	-1.095252	1.469769	H	0.695635	0.054065	1.276780
C	-0.229489	-0.254467	1.765378	H	-0.572266	0.571753	2.394945
C	1.438382	-4.023467	-0.295497	H	-0.019886	-1.103732	2.422944
C	5.385645	0.219288	-1.085573	H	-5.962028	2.388144	-1.159854
C	1.020085	3.193674	1.645908	H	-4.915560	2.595881	0.259475
C	-4.232233	1.067260	-1.139046	H	-5.994366	1.206113	0.165058
C	-4.802554	0.065605	-2.174829	H	-0.010171	3.302204	1.990948
C	-5.345087	1.858133	-0.425931	H	1.534957	2.520556	2.343834
O	-2.670028	-1.890341	2.395940	H	1.511187	4.169929	1.716178
O	2.678819	4.291718	-0.657978	H	1.898149	4.644935	-1.113524
O	-4.958771	-0.775217	1.247489	H	1.815556	-4.743775	-1.032163
O	-5.364868	-1.110504	-1.626586	H	2.069389	-4.115620	0.596539
O	3.257871	-1.738418	1.411170	H	0.424397	-4.322803	-0.024600
H	2.849831	-2.908710	-2.481827	H	5.037553	-0.111494	-2.072714
H	2.736769	-1.235024	-1.938860	H	5.637476	1.278587	-1.166424
H	4.938039	-2.372085	-1.447258	H	6.313955	-0.329850	-0.880181
H	4.120049	-3.498743	-0.381759	H	2.391862	-1.614022	0.984464

Table S55. Atomic coordinates (Å) of **8c-4** obtained at the B3LYP-D3BJ/6-31G(d) level of theory in the gas phase.

C	1.230657	-1.051355	-0.545845	H	-0.802005	-0.747371	1.278648
C	0.571414	-2.174351	0.305636	H	-5.316586	-1.318840	-0.976967
C	-0.750329	-1.767936	0.898802	H	-2.772733	0.668470	-1.496616
C	-1.853741	-2.525575	1.012018	H	-4.143835	3.073233	-0.112012
C	-3.104782	-1.967681	1.662962	H	-3.220009	3.092468	-1.601466
C	-4.377741	-2.051791	0.799187	H	-2.121727	2.606726	1.198155
C	-4.362556	-1.154127	-0.455183	H	-0.109175	1.764605	1.383951
C	-4.291464	0.335371	-0.127354	H	1.779540	1.645296	-1.071979
C	-3.425802	1.135819	-0.763740	H	2.276203	2.154863	0.522744
C	-3.281474	2.623289	-0.611733	H	1.277789	-2.447376	1.104690
C	-2.040458	3.041126	0.189317	H	0.463026	-3.056960	-0.330737
C	-0.721333	2.604918	-0.419898	H	1.430251	-0.079229	1.412134
C	0.174552	1.970448	0.349256	H	4.713793	-2.254295	-1.588381
C	1.561066	1.514777	-0.009326	H	5.050446	-0.396890	1.657092
C	1.840456	0.046096	0.401633	H	4.509851	2.161115	0.125842
C	3.335448	-0.248740	0.435680	H	5.699080	2.062553	1.433521
C	3.649199	-1.140311	-0.524777	H	5.810588	0.212431	-0.689221
C	2.466749	-1.664144	-1.197625	H	0.723668	0.140555	-2.312841
C	0.261202	-0.585982	-1.638948	H	-0.034276	-1.450002	-2.242038
C	-1.948769	-3.956711	0.539785	H	-0.631115	-0.128746	-1.210066
C	-5.307169	0.810633	0.882323	H	4.474299	1.234332	3.369300
C	-0.504995	2.987316	-1.860779	H	3.098457	0.140216	3.170796
C	4.328599	0.384083	1.381073	H	3.037603	1.774419	2.495325
C	5.175905	1.472472	0.672240	H	-0.858416	4.009987	-2.029205
C	3.691930	0.914612	2.673794	H	0.543872	2.933417	-2.159565
O	2.516729	-2.502791	-2.089529	H	-1.076251	2.332190	-2.531496
O	-2.099020	4.473212	0.264077	H	-1.297891	4.762554	0.729077
O	4.878336	-1.599840	-0.877932	H	-0.973644	-4.401783	0.334034
O	6.180889	0.944862	-0.168715	H	-2.448241	-4.576440	1.294953
O	-3.372625	-1.581132	-1.383507	H	-2.549792	-4.017842	-0.375202
H	-3.291233	-2.531864	2.588754	H	-5.411670	1.897378	0.888809
H	-2.931065	-0.925514	1.957214	H	-6.295734	0.381214	0.672689
H	-5.241010	-1.788050	1.420147	H	-5.040384	0.500579	1.900981
H	-4.540519	-3.083164	0.468784	H	-2.515003	-1.532256	-0.926002

Table S56. Conformational analysis of the B3LYP-D3BJ/6-31G(d) optimized conformers of **8d** in the gas phase (T=298.15 K).

Conformer	E (Hartree)[a]	C (Hartree)[b]	G (kcal/mol)[c]	ΔG (kcal/mol)[d]	Population[e]
**8d-1**	-1351.467917	0.527971	-847714.815971	0.0	36.41%
**8d-2**	-1351.467348	0.527681	-847714.640973	0.174997	27.10%
**8d-3**	-1351.467123	0.527623	-847714.536376	0.279595	22.71%
**8d-4**	-1351.466956	0.527927	-847714.24061	0.575361	13.78%

[a]Electronic energy obtained at the M06-2X-D3/def2-TZVP level of theory; [b]Thermal correction to Gibbs free energy obtained at the M06-2X-D3/def2-SVP level of theory; [c]Gibbs free energy (E + C); [d]The relative Gibbs free energy; [e]The Boltzmann distribution of each conformer.

Table S57. Atomic coordinates (Å) of **8d-1** obtained at the B3LYP-D3BJ/6-31G(d) level of theory in the gas phase.

C	-1.995109	0.614050	-1.460457	H	0.647352	1.167017	-1.638725
C	-1.344573	1.963291	-1.044937	H	3.441859	1.355077	2.536545
C	0.165098	1.959173	-1.067555	H	1.891419	-1.013046	0.813507
C	0.945950	2.858083	-0.444609	H	3.401002	-3.047311	0.507914
C	2.458297	2.783528	-0.510644	H	4.455364	-2.101058	-0.543375
C	3.144549	2.667063	0.866018	H	2.920814	-3.456671	-1.879030
C	2.954877	1.300192	1.553174	H	0.401768	-1.301270	-2.335244
C	3.610251	0.154467	0.787670	H	-2.022877	-2.110439	-1.834438
C	2.930969	-0.965920	0.507092	H	-1.458883	-2.647488	-0.264947
C	3.425356	-2.202153	-0.192138	H	-1.695235	2.216081	-0.036918
C	2.540884	-2.533329	-1.422045	H	-1.739695	2.744660	-1.709415
C	1.091476	-2.732924	-1.003266	H	-0.666369	-0.206433	0.085894
C	0.121436	-1.981261	-1.536010	H	-5.497386	0.839243	-0.057196
C	-1.293033	-1.900165	-1.042831	H	-2.236067	-2.291124	1.700971
C	-1.580266	-0.496155	-0.437554	H	-1.440392	0.460298	2.661714
C	-2.718951	-0.553223	0.573942	H	-1.495217	-0.933328	3.757161
C	-3.794443	0.112530	0.094396	H	0.550478	-0.420366	2.359059
C	-3.484155	0.778931	-1.171839	H	-2.170073	-0.598947	-3.278811
C	-1.769422	0.364863	-2.955645	H	-2.291465	1.143878	-3.519952
C	0.380996	3.998814	0.370390	H	-0.710750	0.397062	-3.226020
C	5.069237	0.372752	0.465462	H	-3.648477	-1.883393	3.653632
C	0.867981	-3.755544	0.087285	H	-4.656930	-1.723003	2.200939
C	-2.545147	-1.253907	1.896826	H	-4.122745	-0.280352	3.056413
C	-1.377805	-0.637685	2.703054	H	-0.112993	-4.230379	0.002574
C	-3.820140	-1.287004	2.751089	H	1.620284	-4.551788	0.039381
O	-4.308604	1.429784	-1.804396	H	0.922656	-3.298700	1.083373
O	2.715045	-1.528395	-2.413527	H	2.611110	-0.678907	-1.950345
O	-5.038536	0.280554	0.604763	H	0.990436	4.901850	0.247537
O	-0.132901	-1.101602	2.201688	H	-0.644623	4.244681	0.086995

O	1.569934	1.082142	1.850916	H	0.384438	3.753137	1.440264
H	2.764342	1.942762	-1.145699	H	5.202349	1.083581	-0.360119
H	2.829771	3.696837	-0.996508	H	5.600193	0.791691	1.330393
H	2.765283	3.441667	1.540823	H	5.572729	-0.554012	0.185668
H	4.216209	2.857697	0.748706	H	1.074929	1.220135	1.020521

Table S58. Atomic coordinates (Å) of **8d-2** obtained at the B3LYP-D3BJ/6-31G(d) level of theory in the gas phase.

C	1.995220	0.112046	1.613475	H	-0.960650	1.125889	1.606602
C	1.110374	1.138781	2.358664	H	-1.774564	2.071223	-2.873380
C	-0.049527	1.722876	1.590688	H	-1.586006	-0.430859	-0.621662
C	-0.115262	2.908290	0.955657	H	-4.495334	-1.084827	-0.692864
C	-1.431008	3.383593	0.360529	H	-4.054268	-0.468049	0.885937
C	-1.522686	3.366301	-1.179427	H	-2.767620	-2.811759	-0.573795
C	-1.615712	1.954423	-1.792852	H	-0.625895	-2.893152	-0.240505
C	-2.781692	1.151351	-1.229465	H	0.550367	-1.980157	2.463614
C	-2.594129	-0.038948	-0.649941	H	1.380827	-3.002065	1.300398
C	-3.640666	-0.925294	-0.023471	H	1.774528	1.929599	2.722869
C	-3.079422	-2.307690	0.353171	H	0.699681	0.644124	3.248547
C	-1.872731	-2.195352	1.266188	H	0.338574	-0.364864	0.345540
C	-0.664760	-2.516543	0.779887	H	4.338757	1.052202	-1.235588
C	0.665978	-2.175127	1.393983	H	1.389016	-3.009430	-1.062214
C	1.223030	-0.897961	0.708233	H	1.713409	-0.886364	-3.219913
C	2.126248	-1.098073	-0.506127	H	1.164958	-2.553104	-3.477105
C	3.001440	-0.067082	-0.576402	H	-0.211289	-0.445891	-2.244056
C	2.927284	0.781253	0.606758	H	2.327054	-1.098686	3.405244
C	2.914859	-0.599180	2.628861	H	3.542877	-1.347143	2.134731
C	1.033091	3.876393	0.833815	H	3.569380	0.133141	3.112275
C	-4.135205	1.798970	-1.381209	H	3.188965	-3.626653	-2.624771
C	-2.133436	-1.603015	2.628111	H	3.893660	-3.153039	-1.067677
C	1.978485	-2.203745	-1.512679	H	3.954887	-2.034805	-2.435907
C	1.182702	-1.728384	-2.753057	H	-3.073767	-1.993741	3.029548
C	3.337838	-2.786592	-1.937075	H	-1.334589	-1.820761	3.341487
O	3.588327	1.806716	0.749280	H	-2.240809	-0.510746	2.575094
O	-4.165938	-3.015616	0.960398	H	-3.834342	-3.898678	1.186852
O	3.889287	0.242441	-1.552674	H	1.176415	4.172594	-0.213013
O	-0.157679	-1.401104	-2.453699	H	0.808792	4.797282	1.389506
O	-0.348513	1.290243	-1.689386	H	1.979612	3.469100	1.189310
H	-2.251864	2.784362	0.768618	H	-4.944444	1.078412	-1.238278
H	-1.596621	4.416663	0.695947	H	-4.284942	2.610024	-0.655463
H	-0.658425	3.870789	-1.625998	H	-4.251325	2.243213	-2.378546

H	-2.408194	3.936962	-1.481612	H	-0.042549	1.390003	-0.769011

Table S59. Atomic coordinates (Å) of **8d-3** obtained at the B3LYP-D3BJ/6-31G(d) level of theory in the gas phase.

C	-1.991165	0.498697	-1.484503	H	0.658534	1.055489	-1.669906
C	-1.354621	1.883484	-1.175978	H	3.324438	1.548950	2.526824
C	0.156210	1.888588	-1.180178	H	1.830924	-0.836482	0.605627
C	0.919087	2.842345	-0.618338	H	3.550513	-2.843625	0.554604
C	2.432960	2.764781	-0.639317	H	4.454539	-1.895252	-0.623615
C	3.077192	2.751972	0.763743	H	2.976510	-3.597495	-1.612908
C	2.879587	1.427638	1.529704	H	0.410996	-1.346380	-2.177478
C	3.580424	0.260795	0.847779	H	-1.998994	-2.256492	-1.627917
C	2.901351	-0.814034	0.435244	H	-1.381291	-2.651531	-0.034851
C	3.456979	-2.054879	-0.205685	H	-1.718792	2.216174	-0.196292
C	2.568690	-2.608457	-1.338371	H	-1.749870	2.603357	-1.906647
C	1.134089	-2.791750	-0.873996	H	-0.656178	-0.182585	0.119978
C	0.149057	-2.050051	-1.392856	H	-5.497558	0.784245	-0.106121
C	-1.255842	-1.965623	-0.874104	H	-2.266136	-2.193414	1.874536
C	-1.564348	-0.525243	-0.379467	H	-1.345359	0.585623	2.630389
C	-2.704323	-0.523601	0.632190	H	-1.419941	-0.735378	3.809763
C	-3.786477	0.092140	0.102957	H	0.589410	-0.349356	2.296796
C	-3.482183	0.665695	-1.209243	H	-2.117480	-0.862591	-3.201421
C	-1.762086	0.141686	-2.957424	H	-2.323770	0.849148	-3.575390
C	0.330596	4.041998	0.088475	H	-0.707307	0.207543	-3.237275
C	5.074163	0.431603	0.703869	H	-3.610836	-1.575255	3.824225
C	0.947033	-3.769253	0.262968	H	-4.647334	-1.496830	2.384518
C	-2.527074	-1.131952	1.999831	H	-4.041641	-0.010043	3.106917
C	-1.318026	-0.509209	2.736920	H	1.753239	-4.511472	0.284371
C	-3.782737	-1.049151	2.879047	H	0.938934	-3.245971	1.227330
O	-4.314711	1.254273	-1.890685	H	0.002230	-4.314108	0.180828
O	2.708779	-1.713474	-2.437399	H	2.170456	-2.068522	-3.162484
O	-5.034940	0.280000	0.595727	H	0.944514	4.933492	-0.085438
O	-0.100283	-1.042195	2.239990	H	-0.686170	4.264106	-0.243192
O	1.485882	1.202623	1.784116	H	0.301365	3.880720	1.174054
H	2.753839	1.876570	-1.195988	H	5.328506	1.093578	-0.134553
H	2.817984	3.641261	-1.179663	H	5.505897	0.883555	1.606506
H	2.674246	3.567607	1.373970	H	5.583326	-0.519562	0.536533
H	4.151434	2.940617	0.664579	H	1.017785	1.321184	0.935196

Table S60. Atomic coordinates (Å) of **8d-4** obtained at the B3LYP-D3BJ/6-31G(d) level of theory in the gas phase.

C	1.870344	0.085734	-1.650521	H	-0.706697	-0.804861	-2.036616
C	1.410673	-1.403785	-1.683420	H	-3.066532	-1.928192	2.294429
C	-0.082584	-1.589528	-1.608729	H	-2.070656	0.853433	1.219032
C	-0.682762	-2.657768	-1.056660	H	-3.812843	2.703964	1.252267
C	-2.191528	-2.780538	-0.996080	H	-4.747272	1.866107	0.012991
C	-2.753028	-2.936475	0.432969	H	-3.375376	3.652389	-0.984044
C	-2.688062	-1.663864	1.297127	H	-0.686856	1.815640	-1.803837
C	-3.550256	-0.512952	0.773805	H	1.679184	2.760456	-1.050507
C	-3.078412	0.740285	0.830944	H	1.066659	2.678241	0.589584
C	-3.735407	2.020090	0.397029	H	1.872443	-1.925275	-0.839646
C	-2.903275	2.695033	-0.727808	H	1.816211	-1.857874	-2.599014
C	-1.474955	2.932998	-0.254250	H	0.487580	0.199472	0.051566
C	-0.451594	2.352739	-0.890200	H	5.422512	-0.223214	-0.292271
C	0.963928	2.234024	-0.405378	H	1.252729	0.984288	2.312223
C	1.366829	0.734864	-0.319532	H	3.258280	-1.300624	2.566397
C	2.499559	0.528565	0.663130	H	2.035410	-0.850891	3.770501
C	3.637925	0.225646	0.007030	H	0.448723	-1.540140	2.236290
C	3.383949	0.032719	-1.422727	H	2.166506	0.316060	-3.761705
C	1.545671	0.767935	-2.981745	H	0.497078	0.643565	-3.266075
C	0.112121	-3.805328	-0.466883	H	1.764956	1.838649	-2.965980
C	-4.941887	-0.886565	0.328077	H	3.066772	1.421293	3.980526
C	-1.341456	3.765175	0.998130	H	3.242754	2.469680	2.560673
C	2.257509	0.570146	2.143815	H	4.294503	1.060027	2.746679
C	2.256472	-0.877727	2.691852	H	-2.041317	4.610200	0.977156
C	3.277701	1.430944	2.904832	H	-1.571533	3.187861	1.904167
O	4.259998	-0.237512	-2.235288	H	-0.335783	4.175595	1.113770
O	-2.986134	1.907536	-1.907726	H	-2.805516	0.992488	-1.628294
O	4.885382	0.010549	0.492204	H	0.554491	-3.526748	0.497261
O	1.372744	-1.749467	2.006149	H	-0.519956	-4.684625	-0.308269
O	-1.329340	-1.267985	1.517479	H	0.933116	-4.102682	-1.126829
H	-2.657357	-1.916144	-1.486284	H	-4.931851	-1.473190	-0.598759
H	-2.492545	-3.667572	-1.571501	H	-5.438615	-1.506775	1.086332
H	-2.204427	-3.723373	0.961572	H	-5.567686	-0.008703	0.157495
H	-3.793962	-3.271922	0.375677	H	-0.899165	-1.235776	0.641664

S5. *Computational data of compound 10*

Table S61. Experimental and calculated NMR data of **10a** and **10b**.

No.	**10a**		**10b**		**10**	
	$\delta_{\text{calcd. H}}$	$\delta_{\text{calcd. C}}$	$\delta_{\text{calcd. H}}$	$\delta_{\text{calcd. C}}$	$\delta_{\text{exptl. H}}$	$\delta_{\text{exptl. C}}$
1		55.4		54.7		50.1
2	2.11	39.7	2.26	39.0	2.29	39.3
	1.78		1.67		1.87	
3	5.65	121.3	5.63	120.6	5.32	121.8
4		140.7		141.0		138.5
5	2.13	42.8	2.27	41.4	2.17	40.7
	2.07		2.03		2.12	
6	2.36	27.2	2.37	26.7	2.24	24.8
	2.18		2.15			
7	5.16	119.6	5.10	119.8	5.09	122.0
8		135.4		136.8		135.2
9	2.20	33.9	2.20	33.8	2.18	33.2
	1.98		1.93		2.11	
10	1.69	29.7	2.11	30.4	1.78	29.6
	1.13		1.11		1.31	
11	3.59	73.3	3.29	74.5	3.48	75.6
12		77.9		78.4		76.5
13	5.95	136.9	6.14	138.2	5.63	140.5
14	5.67	132.3	5.91	130.6	5.28	131.6
15	3.57	53.4	3.52	54.9	3.33	54.6
16		149.6		149.7		149.9
17		145.5		144.7		149.7
18		203.6		204.3		209.7
19	0.83	19.2	0.84	19.8	0.94	18.7
20	1.63	16.9	1.56	16.8	1.65	16.1
21	1.60	20.1	1.58	20.2	1.65	19.2
22	1.12	18.2	1.25	27.2	1.21	17.7
23	3.32	38.7	2.92	40.6	2.71	38.0
24	3.87	69.0	3.98	66.4	3.77	66.1
	3.31		3.53		3.66	
25	1.00	13.2	1.01	15.8	1.20	14.7

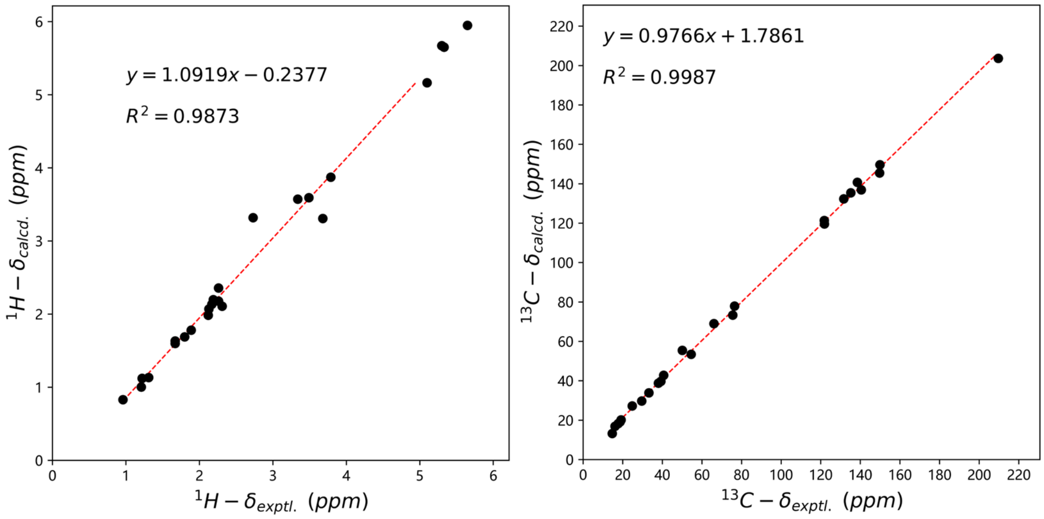

Figure S15. Linear regression analyses between experimental and calculated NMR data of **10a**.

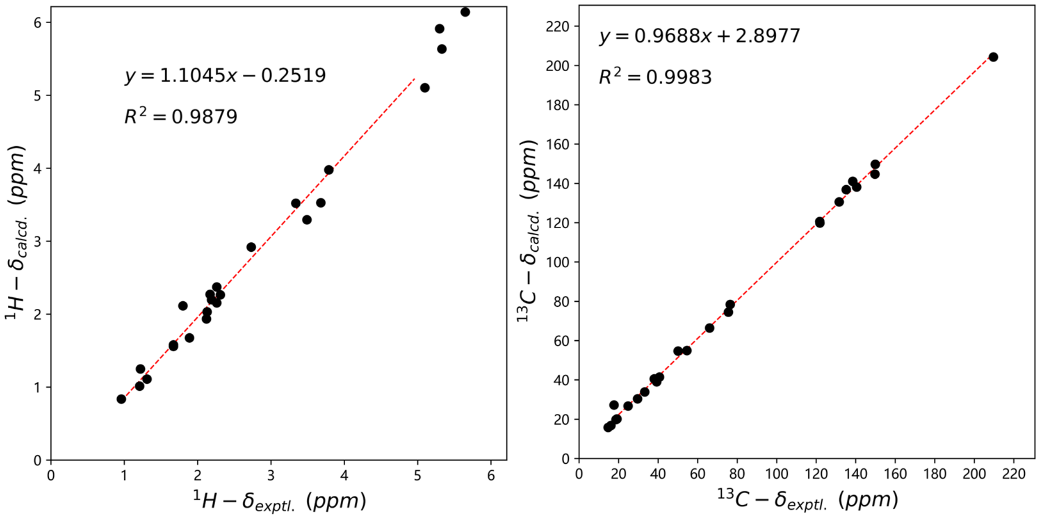

Figure S16. Linear regression analyses between experimental and calculated NMR data of **10b**.

Functional	Solvent?	Basis Set	Type of Data
mPW1PW91	PCM	6-31+G(d,p)	Unscaled Shifts

	Isomer 1	Isomer 2	Isomer 3	Isomer 4	Isomer 5	Isomer 6
sDP4+ (H data)	99.90%	0.10%	–	–	–	–
sDP4+ (C data)	83.00%	17.00%	–	–	–	–
sDP4+ (all data)	99.98%	0.02%	–	–	–	–
uDP4+ (H data)	99.20%	0.80%	–	–	–	–
uDP4+ (C data)	22.78%	77.22%	–	–	–	–
uDP4+ (all data)	97.34%	2.66%	–	–	–	–
DP4+ (H data)	100.00%	0.00%	–	–	–	–
DP4+ (C data)	59.03%	40.97%	–	–	–	–
DP4+ (all data)	100.00%	0.00%	–	–	–	–

Figure S17. DP4+ analysis results of **10a** (left) and **10b** (right).

Table S62. Conformational analysis of the B3LYP-D3BJ/6-31G(d) optimized conformers of **10a** in the gas phase (T=298.15 K).

Conformer	E (Hartree)	C (Hartree)	G (kcal/mol)	ΔG (kcal/mol)	Population
**10a-1**	-1351.469288	0.522868	-847718.878261	0.0	52.13%
**10a-2**	-1351.469157	0.523173	-847718.604954	0.273308	32.85%
**10a-3**	-1351.468211	0.523508	-847717.801409	1.076853	8.46%
**10a-4**	-1351.467257	0.522793	-847717.651405	1.226857	6.56%

Electronic energy obtained at @@@ level of theory; Thermal correction to Gibbs free energy obtained at @@@ level of theory; Gibbs free energy (E + C); The relative Gibbs free energy; The Boltzmann distribution of each conformer.

Table S63. Atomic coordinates (Å) of **10a-1** obtained at the B3LYP-D3BJ/6-31G(d) level of theory in the gas phase.

C	1.341118	-1.667543	-0.365669	H	-0.330434	-1.808137	2.123786
C	0.877664	-2.811454	0.585646	H	-2.500063	-0.464406	0.261056
C	-0.413197	-2.508835	1.292781	H	-4.035502	0.855956	-2.533505
C	-1.640702	-2.971245	1.011736	H	-4.556019	1.991753	-1.314114
C	-2.825171	-2.528104	1.843139	H	-2.201039	2.285938	-2.149910
C	-3.978695	-1.916644	1.023948	H	-1.741726	0.787415	-1.349573
C	-3.551137	-0.732652	0.183919	H	-2.593568	1.839299	0.835888
C	-4.348826	-0.021516	-0.625115	H	-0.324341	1.071642	1.615323
C	-3.883396	1.139127	-1.480576	H	1.331165	1.284277	-0.961993
C	-2.442125	1.627180	-1.308459	H	0.815713	-3.728209	-0.008048
C	-2.224900	2.419203	-0.023319	H	1.669240	-2.971935	1.330808
C	-0.734381	2.743106	0.266863	H	1.393794	-0.522123	1.508150
C	0.007140	1.492577	0.665145	H	4.997606	-2.054689	-1.463514
C	0.981929	0.882899	-0.013411	H	5.025061	0.480876	1.104702
C	1.680481	-0.367270	0.461411	H	3.103434	2.661290	0.190846
C	3.190553	-0.219324	0.362275	H	4.834745	2.796062	0.547084
C	3.702796	-1.136520	-0.478252	H	4.962904	1.174571	-1.304074
C	2.686239	-2.063173	-0.966125	H	0.260239	-2.403270	-2.078376
C	0.325090	-1.477186	-1.498779	H	-0.663883	-1.242932	-1.099188
C	-1.953815	-3.928421	-0.109299	H	0.624146	-0.675812	-2.180707
C	-5.819215	-0.324022	-0.781684	H	3.523017	0.344338	3.092973
C	-0.087222	3.523319	-0.876020	H	2.474850	1.597920	2.403599
C	3.996275	0.860282	1.025237	H	4.137052	1.991032	2.877987
C	4.058134	2.128562	0.138855	H	-2.697140	4.158270	0.638358
C	3.501148	1.217648	2.433176	H	-1.058840	-4.360489	-0.561333
O	-2.964482	3.629696	-0.133396	H	-2.587679	-4.750726	0.247477
O	4.992222	-1.268812	-0.879531	H	-2.512943	-3.419631	-0.905020
O	2.930877	-3.000626	-1.716439	H	0.018563	2.914505	-1.777707
O	-0.828786	3.595421	1.437698	H	0.913892	3.868724	-0.591643
O	4.274263	1.856097	-1.234268	H	-0.709489	4.388391	-1.115371

H	-2.490010	-1.801957	2.593802	H	0.060131	3.937790	1.620072
H	-3.222809	-3.392827	2.395085	H	-6.425885	0.522380	-0.431527
H	-4.418033	-2.697189	0.388013	H	-6.075936	-0.472394	-1.839506
H	-4.774158	-1.626665	1.723801	H	-6.133519	-1.213215	-0.230760

Table S64. Atomic coordinates (Å) of **10a-2** obtained at the B3LYP-D3BJ/6-31G(d) level of theory in the gas phase.

C	-1.584501	1.407029	-1.076954	H	0.791558	3.267904	-1.184803
C	-1.257601	2.831693	-0.530806	H	2.267717	0.707951	0.084151
C	0.205136	3.075955	-0.287199	H	4.454294	-0.166095	-2.304928
C	0.864612	3.094555	0.881720	H	4.995615	-1.304864	-1.096209
C	2.347272	3.398456	0.916237	H	2.888049	-1.978974	-2.265960
C	3.245373	2.207100	1.333458	H	2.045458	-0.618775	-1.535184
C	3.255471	1.096686	0.319640	H	2.897905	-1.627314	0.743840
C	4.316674	0.559597	-0.301862	H	0.714681	-0.790266	1.202836
C	4.206615	-0.570901	-1.311693	H	-1.176756	-1.477578	-1.112581
C	2.873635	-1.319905	-1.390857	H	-1.623555	3.551090	-1.274424
C	2.617146	-2.187289	-0.160453	H	-1.848530	2.991325	0.377688
C	1.133404	-2.589412	0.028804	H	-0.955085	0.795838	0.935841
C	0.329409	-1.358448	0.355364	H	-5.390633	0.957453	-1.290540
C	-0.752888	-0.913453	-0.285307	H	-4.381986	-0.560635	1.842117
C	-1.482724	0.351337	0.086546	H	-2.380536	-2.721425	1.066766
C	-2.924806	0.037284	0.455213	H	-3.919350	-2.907074	1.930228
C	-3.767412	0.566658	-0.450851	H	-4.782890	-1.970602	-0.183883
C	-3.068541	1.389952	-1.433147	H	-0.893325	1.886535	-3.066544
C	-0.743588	1.098649	-2.321615	H	0.319283	1.043942	-2.071856
C	0.227118	2.849521	2.227373	H	-1.035037	0.149858	-2.780873
C	5.743183	1.003323	-0.086025	H	-1.449522	-0.993077	2.652719
C	0.603513	-3.398297	-1.152483	H	-2.870155	-1.322382	3.657370
C	-3.351007	-0.847937	1.589958	H	-2.479899	0.342849	3.198894
C	-3.399880	-2.331028	1.146742	H	3.170867	-3.908730	0.483086
C	-2.484369	-0.694027	2.847177	H	0.488910	3.651679	2.929690
O	3.430664	-3.349405	-0.269227	H	-0.862465	2.786897	2.179563
O	-5.114076	0.415934	-0.523154	H	0.590843	1.914937	2.674864
O	-3.633637	1.979777	-2.346360	H	-0.405222	-3.777006	-0.948848
O	1.189688	-3.429585	1.210624	H	1.271423	-4.242952	-1.334152
O	-3.990646	-2.529588	-0.125031	H	0.554931	-2.794407	-2.063133
H	2.520964	4.219062	1.627889	H	0.307192	-3.812587	1.334724
H	2.675420	3.753003	-0.067313	H	6.230574	1.204796	-1.049479
H	4.254243	2.586722	1.517643	H	5.834792	1.901096	0.528357
H	2.891288	1.816863	2.299289	H	6.325533	0.205308	0.394458

Table S65. Atomic coordinates (Å) of **10a-3** obtained at the B3LYP-D3BJ/6-31G(d) level of theory in the gas phase.

C	-1.386970	1.630702	-0.286888	H	0.607261	1.530506	1.837874
C	-0.824325	2.719515	0.670752	H	3.052334	1.560477	-1.229126
C	0.551744	2.399543	1.181803	H	3.714048	-0.356660	-2.667471
C	1.703863	3.032579	0.914683	H	4.464518	-1.741353	-1.878451
C	2.995488	2.556849	1.545049	H	2.093101	-2.176295	-2.277416
C	4.140948	2.263456	0.545761	H	1.629171	-0.692268	-1.436501
C	3.787988	1.234508	-0.491249	H	2.590008	-1.604441	0.678444
C	4.260582	-0.014860	-0.608514	H	0.328036	-1.116654	1.614505
C	3.760500	-0.916602	-1.725672	H	-1.307729	-1.302585	-0.977008
C	2.361992	-1.504287	-1.454084	H	-0.851168	3.673373	0.136913
C	2.266224	-2.265954	-0.140252	H	-1.518796	2.813851	1.518401
C	0.824206	-2.718668	0.220405	H	-1.402741	0.430074	1.547959
C	0.013812	-1.523852	0.652623	H	-5.083806	2.081920	-1.205337
C	-0.974392	-0.923581	-0.013611	H	-5.019869	-0.650455	1.129290
C	-1.696980	0.297435	0.498972	H	-3.048262	-2.734017	0.100969
C	-3.206367	0.131252	0.418126	H	-4.773270	-2.931297	0.458009
C	-3.752120	1.101624	-0.337528	H	-4.963866	-1.232829	-1.307221
C	-2.759247	2.074018	-0.782778	H	-0.401028	2.424997	-2.031676
C	-0.459694	1.470110	-1.499729	H	0.544340	1.185503	-1.178156
C	1.823921	4.242797	0.023317	H	-0.833186	0.717132	-2.200194
C	5.274439	-0.646549	0.312825	H	-2.438011	-1.765023	2.365559
C	0.184874	-3.556420	-0.884779	H	-4.088139	-2.223154	2.823805
C	-3.982543	-1.000054	1.028066	H	-3.515411	-0.573551	3.117423
C	-4.016300	-2.224117	0.080937	H	2.899769	-3.936190	0.561614
C	-3.473425	-1.413023	2.416032	H	0.856560	4.656067	-0.268076
O	3.126174	-3.396517	-0.216317	H	2.394932	5.035972	0.523807
O	-5.053991	1.251587	-0.687366	H	2.369092	4.000231	-0.898023
O	-3.037262	3.071033	-1.438925	H	-0.762384	-3.989534	-0.541612
O	1.047140	-3.552088	1.386911	H	0.866375	-4.363800	-1.163517
O	-4.247303	-1.887739	-1.275915	H	-0.029461	-2.961966	-1.776473
H	2.802502	1.652920	2.134278	H	0.201933	-3.970052	1.613507
H	3.353015	3.327511	2.245787	H	6.232187	-0.781587	-0.209618
H	4.420867	3.199618	0.042829	H	5.464941	-0.062229	1.216348
H	5.021125	1.956481	1.118849	H	4.943625	-1.649420	0.606990

Table S66. Atomic coordinates (Å) of **10a-4** obtained at the B3LYP-D3BJ/6-31G(d) level of theory in the gas phase.

C	-1.598911	1.468380	-1.011512	H	0.787777	3.317339	-1.119436
C	-1.246322	2.870778	-0.428661	H	2.274680	0.672124	0.108895
C	0.223992	3.096418	-0.213849	H	4.350859	-0.179266	-2.388972
C	0.914984	3.068669	0.936352	H	4.953973	-1.316997	-1.221208
C	2.399378	3.366451	0.939844	H	2.790167	-2.005592	-2.275591
C	3.303102	2.167486	1.320252	H	1.984535	-0.622715	-1.549436
C	3.271059	1.063165	0.300600	H	2.862836	-1.601375	0.733395
C	4.304208	0.536765	-0.375225	H	0.651035	-0.816202	1.276370
C	4.145312	-0.586306	-1.387490	H	-1.141090	-1.411875	-1.139890
C	2.807339	-1.331622	-1.412363	H	-1.623036	3.616902	-1.139866
C	2.563841	-2.170506	-0.155424	H	-1.813697	3.006417	0.498646
C	1.090329	-2.588392	0.068894	H	-0.995055	0.793149	0.988363
C	0.299155	-1.347706	0.391480	H	-5.401915	1.026547	-1.253064
C	-0.746191	-0.871788	-0.283498	H	-4.407763	-0.695621	1.762543
C	-1.497829	0.371788	0.112093	H	-2.321286	-2.765087	0.951037
C	-2.939356	0.017864	0.447792	H	-3.888239	-3.025327	1.737895
C	-3.781780	0.593441	-0.430678	H	-4.714158	-2.035749	-0.341358
C	-3.085612	1.477731	-1.358287	H	-0.928475	1.997422	-2.996628
C	-0.778810	1.186152	-2.277086	H	0.286792	1.110374	-2.044189
C	0.313406	2.777013	2.289076	H	-1.089843	0.254938	-2.758887
C	5.736787	0.990415	-0.231388	H	-1.488138	-1.095547	2.632885
C	0.537679	-3.393760	-1.110729	H	-2.926909	-1.496718	3.579282
C	-3.362191	-0.938678	1.524336	H	-2.562129	0.196628	3.209703
C	-3.349993	-2.399940	1.007035	H	4.225485	-3.196905	0.083717
C	-2.532705	-0.822956	2.810814	H	0.663230	1.812596	2.681058
O	3.319865	-3.397281	-0.192350	H	0.617665	3.538133	3.019428
O	-5.127213	0.439609	-0.519328	H	-0.778289	2.744744	2.272038
O	-3.650992	2.125126	-2.232272	H	-0.473994	-3.738663	-0.880703
O	1.057152	-3.390056	1.258399	H	1.172955	-4.266469	-1.285676
O	-3.887824	-2.544078	-0.297478	H	0.505442	-2.806261	-2.033674
H	2.594132	4.175819	1.658942	H	1.820583	-3.988683	1.163947
H	2.705600	3.734116	-0.046007	H	6.167018	1.217366	-1.216181
H	4.320087	2.538687	1.474900	H	5.852935	1.876663	0.394956
H	2.976264	1.771401	2.292938	H	6.356833	0.191944	0.199789

Table S67. Conformational analysis of the B3LYP-D3BJ/6-31G(d) optimized conformers of **10b** in the gas phase (T=298.15 K).

Conformer	E (Hartree)[a]	C (Hartree)[b]	G (kcal/mol)[c]	ΔG (kcal/mol)[d]	Population[e]
**10b-1**	-1351.467369	0.521353	-847718.624933	0.0	43.36%
**10b-2**	-1351.470183	0.524648	-847718.323143	0.30179	26.04%
**10b-3**	-1351.469786	0.525027	-847717.836411	0.788523	11.45%
**10b-4**	-1351.465771	0.52144	-847717.567746	1.057187	7.27%
**10b-5**	-1351.466871	0.522707	-847717.462778	1.162155	6.09%
**10b-6**	-1351.465892	0.521775	-847717.433148	1.191786	5.79%

[a]Electronic energy obtained at the M06-2X-D3/def2-TZVP level of theory; [b]Thermal correction to Gibbs free energy obtained at the B3LYP-D3BJ/6-31G(d) level of theory; [c]Gibbs free energy (E + C); [d]The relative Gibbs free energy; [e]The Boltzmann distribution of each conformer.

Table S68. Atomic coordinates (Å) of **10b-1** obtained at the B3LYP-D3BJ/6-31G(d) level of theory in the gas phase.

C	1.325629	-1.458293	-0.581507	H	-0.119060	-1.633081	2.040475
C	1.026185	-2.600761	0.434963	H	-2.499024	-0.461318	0.445426
C	-0.219285	-2.368945	1.242618	H	-4.096154	0.524100	-2.456379
C	-1.425566	-2.934842	1.086546	H	-4.731695	1.668377	-1.298984
C	-2.566362	-2.550451	2.003847	H	-2.352310	2.095124	-2.113370
C	-3.815132	-2.026679	1.266164	H	-1.835089	0.694899	-1.205289
C	-3.510192	-0.852618	0.362897	H	-2.907293	1.859358	0.871878
C	-4.356424	-0.285539	-0.508708	H	-0.295053	1.174611	1.643104
C	-3.985250	0.869836	-1.417164	H	0.827040	1.470426	-1.186277
C	-2.593745	1.479345	-1.239550	H	0.985571	-3.539076	-0.125812
C	-2.494438	2.377245	0.001026	H	1.885550	-2.681096	1.116350
C	-1.035681	2.793873	0.333732	H	1.518412	-0.266061	1.252505
C	-0.168778	1.595495	0.645214	H	4.893563	-1.628150	-1.932578
C	0.719021	1.050382	-0.188038	H	3.000939	1.949060	1.087230
C	1.613774	-0.107331	0.170228	H	5.680020	0.496964	0.896519
C	3.072760	0.218245	-0.119621	H	5.245644	1.809436	2.009780
C	3.618963	-0.717904	-0.923209	H	4.156488	-0.782963	1.997605
C	2.655078	-1.767414	-1.260362	H	0.205134	-2.336460	-2.201037
C	0.221463	-1.394126	-1.644511	H	-0.755168	-1.244709	-1.179037
C	-1.758756	-3.952430	0.026270	H	0.394032	-0.584505	-2.359817
C	-5.780256	-0.753982	-0.681181	H	4.815564	3.218807	-0.098990
C	-1.013715	3.758586	1.527862	H	3.524298	2.742760	-1.218876
C	3.750362	1.411060	0.493981	H	5.050837	1.846284	-1.202998
C	4.852573	0.944618	1.465774	H	-2.854793	4.064696	-0.849945
C	4.321037	2.361868	-0.571266	H	-2.288140	-4.809433	0.462731
O	-3.303817	3.540167	-0.164292	H	-2.427188	-3.516640	-0.727345

O	4.883070	-0.805949	-1.399469	H	-0.875989	-4.327756	-0.495182
O	2.938443	-2.733106	-1.959238	H	0.009591	4.105948	1.717465
O	-0.593726	3.477412	-0.852008	H	-1.370216	3.261929	2.436840
O	4.360804	0.048311	2.454219	H	-1.659595	4.615415	1.328621
H	-2.219810	-1.788180	2.712432	H	0.275634	3.861939	-0.657001
H	-2.863637	-3.425191	2.600900	H	-6.013630	-1.639312	-0.085364
H	-4.263428	-2.847081	0.690947	H	-6.483183	0.039692	-0.393136
H	-4.565517	-1.752104	2.020936	H	-5.990119	-0.986812	-1.734056

Table S69. Atomic coordinates (Å) of **10b-2** obtained at the B3LYP-D3BJ/6-31G(d) level of theory in the gas phase.

C	1.375830	-1.716499	-0.431606	H	-0.151837	-1.742335	2.143864
C	0.978555	-2.815776	0.595377	H	-2.414033	-0.407412	0.422331
C	-0.276244	-2.482754	1.353578	H	-3.882054	0.610032	-2.546701
C	-1.516955	-2.952760	1.155498	H	-4.469774	1.812739	-1.424507
C	-2.658756	-2.464567	2.021186	H	-2.042697	2.067743	-2.193446
C	-3.845687	-1.877832	1.230714	H	-1.645422	0.663465	-1.232718
C	-3.442762	-0.743073	0.314751	H	-2.752155	1.986954	0.761228
C	-4.230307	-0.146070	-0.591902	H	-0.194582	1.064511	1.723226
C	-3.770452	0.968315	-1.511629	H	0.956761	1.172105	-1.105523
C	-2.348870	1.494757	-1.311422	H	0.894488	-3.761733	0.052345
C	-2.228320	2.423764	-0.094974	H	1.809104	-2.934505	1.305244
C	-0.758674	2.692929	0.320032	H	1.594444	-0.501649	1.382560
C	-0.036165	1.420003	0.704580	H	4.938462	-2.010856	-1.771932
C	0.819258	0.790539	-0.099305	H	4.869071	1.177608	-0.078658
C	1.701335	-0.358060	0.298917	H	2.467102	2.293855	1.452752
C	3.169436	-0.073333	-0.015241	H	4.033700	3.084931	1.223241
C	3.679323	-1.045097	-0.798300	H	3.483040	3.024928	-1.079840
C	2.699232	-2.093131	-1.081644	H	0.245663	-2.574090	-2.056222
C	0.308625	-1.615151	-1.532459	H	-0.668839	-1.381948	-1.105190
C	-1.887117	-3.960136	0.097779	H	0.558581	-0.845936	-2.268985
C	-5.673652	-0.536737	-0.795778	H	3.742011	0.350433	2.652460
C	-0.706284	3.670947	1.505041	H	5.202782	1.324248	2.398934
C	4.006350	1.028446	0.586907	H	5.145173	-0.356004	1.829807
C	3.314185	2.386495	0.771094	H	-2.326075	4.117450	-0.999133
C	4.558680	0.558398	1.951629	H	-2.500184	-4.764681	0.524698
O	-2.894078	3.660037	-0.354616	H	-2.487636	-3.487134	-0.689748
O	4.941438	-1.170922	-1.268101	H	-1.016092	-4.413198	-0.379932
O	2.964289	-3.098870	-1.729822	H	-1.251960	4.582876	1.256562
O	-0.167242	3.302920	-0.833524	H	0.334361	3.926568	1.736909
O	2.772229	2.945699	-0.425457	H	-1.158213	3.230042	2.401286

H	-2.283354	-1.708984	2.722302	H	0.796154	3.362888	-0.680882
H	-3.035625	-3.300311	2.629516	H	-5.866690	-0.782414	-1.849001
H	-4.326474	-2.681213	0.657590	H	-5.975445	-1.392472	-0.187528
H	-4.600992	-1.541944	1.955366	H	-6.337235	0.302814	-0.547045

Table S70. Atomic coordinates (Å) of **10b-3** obtained at the B3LYP-D3BJ/6-31G(d) level of theory in the gas phase.

C	1.513552	-1.558581	-0.895702	H	-1.008568	-2.958671	-0.305654
C	1.160910	-2.828646	-0.070062	H	-2.188665	-0.791502	0.058245
C	-0.248045	-2.799249	0.453365	H	-4.147783	0.080521	-2.605058
C	-0.661320	-2.546725	1.703975	H	-4.862269	1.060245	-1.347506
C	-2.129414	-2.484940	2.084369	H	-2.642323	1.902030	-2.263680
C	-3.164070	-2.545627	0.951899	H	-1.852199	0.541612	-1.504774
C	-3.137044	-1.320289	0.065781	H	-3.117959	1.436691	0.688878
C	-4.141065	-0.844747	-0.685912	H	-0.514697	0.709696	1.518449
C	-4.013944	0.391813	-1.557548	H	0.688373	1.288444	-1.230318
C	-2.720869	1.199007	-1.427367	H	1.301937	-3.695717	-0.727549
C	-2.675518	2.017304	-0.126727	H	1.892060	-2.928041	0.739214
C	-1.240435	2.416838	0.298177	H	1.400665	-0.608885	1.070508
C	-0.376132	1.202872	0.556017	H	5.194304	-1.214997	-1.849388
C	0.569384	0.778028	-0.280544	H	4.532691	1.656579	0.203031
C	1.575050	-0.291004	0.035800	H	1.866851	2.334530	1.537829
C	3.007504	0.228637	-0.086810	H	3.334227	3.323309	1.517768
C	3.723190	-0.556566	-0.917090	H	2.986741	3.396266	-0.827254
C	2.935991	-1.679774	-1.424161	H	0.640436	-2.312998	-2.724168
C	0.572673	-1.413149	-2.104556	H	-0.463763	-1.276553	-1.788298
C	0.260684	-2.301139	2.873686	H	0.852443	-0.556937	-2.725974
C	-5.496126	-1.502538	-0.771395	H	4.761744	-0.032882	1.979734
C	-1.281129	3.276986	1.572432	H	3.183348	0.406321	2.658789
C	3.619306	1.341411	0.727716	H	4.523949	1.566715	2.710961
C	2.748279	2.587484	0.947200	H	-3.009215	3.765388	-0.855131
C	4.049131	0.786715	2.104575	H	0.004183	-2.963095	3.711538
O	-3.495442	3.179681	-0.248908	H	1.315133	-2.457729	2.636511
O	5.028885	-0.453530	-1.256107	H	0.148738	-1.273529	3.247686
O	3.403167	-2.553393	-2.145469	H	-0.274609	3.634283	1.819353
O	-0.738949	3.196264	-0.793521	H	-1.659075	2.701491	2.425480
O	2.240299	3.181439	-0.247192	H	-1.937333	4.135381	1.418186
H	-2.292681	-1.557127	2.655169	H	0.204793	3.377742	-0.617218
H	-2.330476	-3.297230	2.799595	H	-6.287029	-0.802831	-0.468946
H	-2.999689	-3.460209	0.362666	H	-5.719008	-1.792158	-1.807632
H	-4.153028	-2.665956	1.408188	H	-5.579198	-2.398607	-0.152361

Table S71. Atomic coordinates (Å) of **10b-4** obtained at the B3LYP-D3BJ/6-31G(d) level of theory in the gas phase.

C	-1.407596	1.443720	-0.529251	H	0.436714	1.404479	1.724451
C	-0.982863	2.536862	0.490980	H	3.016536	1.665346	-1.026195
C	0.370011	2.293646	1.097474	H	3.913338	-0.139574	-2.517942
C	1.486262	3.024555	0.957960	H	4.697425	-1.498975	-1.716394
C	2.752247	2.624765	1.686709	H	2.286462	-1.947685	-2.209213
C	3.992771	2.429933	0.780529	H	1.829212	-0.553172	-1.243184
C	3.778560	1.400090	-0.290868	H	2.872115	-1.658362	0.774762
C	4.379162	0.210696	-0.439939	H	0.301546	-1.210313	1.589424
C	3.955675	-0.705003	-1.578995	H	-0.833328	-1.448216	-1.240030
C	2.576397	-1.343903	-1.340636	H	-1.038072	3.503173	-0.017546
C	2.530166	-2.230322	-0.093819	H	-1.738323	2.561921	1.290909
C	1.102641	-2.750150	0.227639	H	-1.520177	0.194117	1.267321
C	0.182759	-1.604266	0.579600	H	-5.059920	1.655793	-1.627279
C	-0.728197	-1.060835	-0.228440	H	-2.943742	-2.088864	1.014509
C	-1.643581	0.061923	0.183204	H	-5.668419	-0.710765	1.014195
C	-3.105716	-0.283186	-0.067442	H	-5.158113	-2.084335	2.015761
C	-3.712484	0.698625	-0.766431	H	-4.149539	0.536584	2.155666
C	-2.788957	1.792488	-1.071930	H	-0.425651	2.375144	-2.209804
C	-0.417847	1.405246	-1.702358	H	0.593285	1.206801	-1.340695
C	1.586878	4.275903	0.122761	H	-0.685488	0.638114	-2.435329
C	5.456163	-0.337237	0.461563	H	-5.074060	-1.890177	-1.194480
C	1.143468	-3.743613	1.397574	H	-4.762431	-3.328089	-0.198067
C	-3.728185	-1.534311	0.485445	H	-3.524508	-2.737264	-1.323417
C	-4.809298	-1.171286	1.522713	H	3.094156	-3.873434	-0.932404
C	-4.308430	-2.426093	-0.624993	H	0.620108	4.619770	-0.249534
O	3.443191	-3.320387	-0.212513	H	2.037143	5.092343	0.702680
O	-5.003258	0.794346	-1.163665	H	2.236843	4.116510	-0.747368
O	-3.131952	2.809778	-1.662737	H	0.138948	-4.138296	1.594676
O	0.687839	-3.426393	-0.970220	H	1.494917	-3.255217	2.312955
O	-4.309824	-0.332227	2.556430	H	1.820703	-4.567649	1.167454
H	2.574664	1.697290	2.243340	H	-0.161668	-3.854934	-0.779299
H	2.995509	3.402669	2.426944	H	5.624415	0.267955	1.355724
H	4.246883	3.392784	0.315058	H	5.211782	-1.359648	0.774698
H	4.843764	2.169262	1.417050	H	6.408964	-0.405499	-0.082056

Table S72. Atomic coordinates (Å) of **10b-5** obtained at the B3LYP-D3BJ/6-31G(d) level of theory in the gas phase.

C	1.136143	-1.860187	-0.381499	H	-0.443553	-1.697676	2.166034
C	0.603808	-2.897237	0.651710	H	-2.517872	-0.210301	0.404710
C	-0.624466	-2.434696	1.383315	H	-3.789022	0.910563	-2.607191
C	-1.899172	-2.798471	1.176840	H	-4.282470	2.186940	-1.520720
C	-3.004292	-2.199065	2.019472	H	-1.828716	2.189785	-2.215977
C	-4.112375	-1.506200	1.200724	H	-1.594576	0.757617	-1.242484
C	-3.574726	-0.433655	0.279904	H	-2.564617	2.159218	0.738750
C	-4.272911	0.229894	-0.652710	H	-0.136588	1.079671	1.677943
C	-3.673064	1.273363	-1.574375	H	1.137400	1.079188	-1.100404
C	-2.210493	1.653865	-1.339513	H	0.434466	-3.836308	0.116877
C	-2.025622	2.568445	-0.121005	H	1.404767	-3.090658	1.378882
C	-0.537171	2.747793	0.282892	H	1.434701	-0.651760	1.419882
C	0.100388	1.433560	0.673977	H	4.681282	-2.657035	-1.567635
C	0.924139	0.720686	-0.095839	H	5.041686	0.177966	0.649983
C	1.614715	-0.544836	0.343106	H	4.251552	1.492197	-1.310379
C	3.120874	-0.482685	0.108321	H	3.068157	2.308870	-0.274123
C	3.525338	-1.517017	-0.655997	H	5.859999	2.429456	0.070017
C	2.418760	-2.411864	-0.991549	H	0.443522	-0.913059	-2.231314
C	0.104952	-1.651806	-1.498744	H	-0.047697	-2.599727	-2.024208
C	-2.344455	-3.784274	0.127690	H	-0.851256	-1.321463	-1.087355
C	-5.744648	-0.006005	-0.886968	H	2.653017	1.410437	2.142240
C	-0.414594	3.735601	1.451299	H	4.359264	1.774804	2.433266
C	4.020982	0.588830	0.656382	H	3.728577	0.147548	2.775473
C	4.046362	1.820273	-0.280661	H	-2.045432	4.259486	-1.034577
C	3.667163	1.000840	2.091788	H	-2.891041	-3.271026	-0.673868
O	-2.616802	3.843144	-0.365711	H	-1.511808	-4.319619	-0.332872
O	4.774023	-1.811326	-1.082775	H	-3.031710	-4.525043	0.556913
O	2.560907	-3.441219	-1.641163	H	-0.894478	3.335441	2.350999
O	0.075446	3.306366	-0.893255	H	-0.898318	4.680131	1.196134
O	4.971152	2.811460	0.143468	H	0.641492	3.915797	1.688679
H	-2.571743	-1.477797	2.723827	H	0.953382	3.633005	-0.639677
H	-3.470870	-2.990581	2.624404	H	-6.319873	0.902433	-0.661427
H	-4.662436	-2.263586	0.627439	H	-5.939377	-0.242177	-1.942005
H	-4.842140	-1.083570	1.905744	H	-6.149295	-0.817488	-0.277843

Table S73. Atomic coordinates (Å) of **10b-6** obtained at the B3LYP-D3BJ/6-31G(d) level of theory in the gas phase.

C	-1.420679	1.653545	-0.312455	H	0.573313	1.510730	1.810130
C	-0.841591	2.727062	0.651907	H	2.967474	1.539880	-1.126674
C	0.528990	2.389969	1.167174	H	3.638766	-0.333891	-2.662502
C	1.685234	3.028392	0.931795	H	4.368028	-1.742418	-1.895141
C	2.966008	2.544573	1.579445	H	1.899879	-2.004726	-2.223439
C	4.123641	2.247368	0.594903	H	1.618996	-0.575583	-1.241667
C	3.760861	1.227031	-0.445197	H	2.721398	-1.754073	0.707697
C	4.266183	-0.001883	-0.624204	H	0.297913	-1.052729	1.687661
C	3.699752	-0.892490	-1.720515	H	-1.152597	-1.296405	-0.991805
C	2.295306	-1.421686	-1.382868	H	-0.857509	3.685192	0.125557
C	2.267342	-2.296990	-0.127690	H	-1.534855	2.825211	1.500308
C	0.827847	-2.687515	0.302084	H	-1.487000	0.454157	1.512144
C	0.041985	-1.462848	0.710041	H	-5.111676	2.149531	-1.160867
C	-0.916660	-0.883916	-0.012697	H	-5.038006	-0.825604	0.867287
C	-1.718087	0.305615	0.449237	H	-2.741152	-2.725442	0.241563
C	-3.223651	0.088516	0.315540	H	-4.449391	-3.135925	0.470886
C	-3.787757	1.097440	-0.382560	H	-3.694923	-1.357456	-1.563102
C	-2.798966	2.106522	-0.773681	H	-0.481220	2.471741	-2.073562
C	-0.517136	1.513271	-1.546149	H	0.496496	1.239274	-1.247011
C	1.820775	4.252923	0.062527	H	-0.892518	0.757810	-2.243303
C	5.365003	-0.617943	0.203814	H	-4.179484	-2.127236	2.808224
C	0.865725	-3.675541	1.476926	H	-3.736902	-0.421419	2.989093
C	-3.970679	-1.071798	0.916931	H	-2.522373	-1.603691	2.464574
C	-3.770692	-2.363974	0.092897	H	2.622284	-3.984855	-0.990164
C	-3.576555	-1.318031	2.381866	H	2.372161	5.042180	0.590390
O	3.070952	-3.462383	-0.303290	H	2.392865	4.028090	-0.846883
O	-5.086436	1.290116	-0.691965	H	0.858838	4.664010	-0.249120
O	-3.094926	3.137341	-1.364485	H	1.453444	-4.555123	1.209061
O	0.267847	-3.326488	-0.854789	H	-0.152495	-3.983397	1.745863
O	-4.067398	-2.209418	-1.282085	H	1.320131	-3.214944	2.360927
H	2.759849	1.639772	2.162993	H	-0.591332	-3.700274	-0.601348
H	3.316040	3.311302	2.287896	H	5.640615	-0.019216	1.075405
H	4.416982	3.183894	0.099670	H	5.072205	-1.617692	0.547512
H	4.994064	1.929107	1.176601	H	6.268181	-0.758798	-0.406365

S6. *NMR data of synthetic and natural 1, 9, 11, 12, 13*

Table S74. ^{1}H NMR data of synthetic **1** and natural **1**.

No.	Synthetic **1**[a]	Natural **1**[a]
	δ_H, mult (J)	δ_H, mult (J)
2	1.63 (m), 1.54 (dd, 14.3, 1.4)	1.64 (m), 1.54 (dd, 14.3, 1.4)
3	2.98 (dd, 8.5, 2.2)	2.98 (dd, 8.6, 2.3)
5	2.12 (overlapped), 1.22 (td, 13.4, 2.5)	2.11 (overlapped), 1.21 (td, 13.5, 2.6)
6	2.34 (m), 2.06 (overlapped)	2.33 (m), 2.05 (overlapped)
7	5.26 (dd, 8.0, 6.4)	5.26 (dd, 8.0, 6.4)
9	2.12 (overlapped), 1.82 (overlapped)	2.11 (overlapped), 1.82 (overlapped)
10	1.83 (overlapped), 1.68 (m)	1.83 (overlapped), 1.68 (m)
11	3.93 (dd, 10.8, 3.2)	3.93 (dd, 10.9, 3.3)
13	5.46 (dd, 7.6, 1.7)	5.46 (dd, 7.6, 1.7)
14	2.56 (m), 2.02 (overlapped)	2.56 (m), 2.01 (overlapped)
15	2.86 (dd, 11.5, 1.6)	2.85 (dd, 11.6, 1.8)
19	1.12 (s)	1.12 (s)
20	1.30 (s)	1.30 (s)
21	1.66 (s)	1.68 (s)
22	1.61 (s)	1.62 (s)
23	2.64 (m)	2.64 (m)
24	3.85 (dd, 10.3, 7.3), 3.71 (dd, 10.3, 6.5)	3.84 (dd, 10.1, 7.2), 3.72 (dd, 10.1, 6.3)
25	1.29 (d, 7.1)	1.28 (d, 7.1)

[a] ^{1}H NMR (800 MHz) in Methanol-d_4 (δ in ppm, J in Hz).

Table S75. ^{13}C NMR data of synthetic **1** and natural **1**.

No.	Synthetic **1**[a]	Natural **1**[a]	$\Delta\delta$	No.	Synthetic **1**[a]	Natural **1**[a]	$\Delta\delta$
	δ_C, type				δ_C, type		
1	47.9 C	47.9 C	0.0	14	30.5 CH_2	30.6 CH_2	−0.1
2	40.8 CH_2	40.9 CH_2	−0.1	15	51.0 CH	51.0 CH	0.0
3	63.8 CH	63.8 CH	0.0	16	152.1 C	151.9 C	+0.2
4	63.1 C	63.1 C	0.0	17	148.9 C	149.4 C	−0.5
5	39.9 CH_2	39.9 CH_2	0.0	18	208.8 C	209.2 C	−0.4
6	24.2 CH_2	24.2 CH_2	0.0	19	18.0 CH_3	18.0 CH_3	0.0
7	125.7 CH	125.7 CH	0.0	20	17.1 CH_3	17.1 CH_3	0.0
8	135.3 C	135.3 C	0.0	21	14.9 CH_3	14.9 CH_3	0.0
9	35.8 CH_2	35.8 CH_2	0.0	22	10.8 CH_3	10.8 CH_3	0.0
10	31.2 CH_2	31.2 CH_2	0.0	23	38.9 CH	38.9 CH	0.0
11	77.0 CH	77.0 CH	0.0	24	65.8 CH_2	66.0 CH_2	−0.2
12	138.0 C	137.9 C	+0.1	25	14.7 CH_3	14.7 CH_3	0.0
13	130.1 CH	130.2 CH	−0.1				

[a] ^{13}C NMR (200 MHz) in Methanol-d_4 (δ in ppm).

Table S76. ^{1}H NMR data of synthetic **9** and natural **9**.

No.	Synthetic **9**[a]	Natural **9**[b]
	δ_H, mult (*J*)	δ_H, mult (*J*)
2	2.42 (t, 11.6, 1.66 (overlapped)	2.43 (t, 12.2), 1.66 (overlapped)
3	5.37 (dd, 10.6, 3.7)	5.37 (dd, 10.9, 3.3)
5	2.28 (overlapped), 2.15 (m)	2.28 (d, 13.6), 2.15 (m)
6	2.24 (overlapped)	2.24 (m)
7	5.15 (t, 5.4)	5.15 (t, 6.2)
9	2.08 (m), 1.96 (m)	2.08 (m), 1.97 (m)
10	1.72 (m), 1.66 (overlapped)	1.72 (m), 1.66 (overlapped)
11	4.24 (t, 6.4)	4.24 (t, 6.4)
13	3.94 (d, 10.6)	3.95 (d, 10.8)
14	1.82 (dd, 14.0, 11.0), 1.63 (overlapped)	1.83 (dd, 14.0, 11.2), 1.63 (overlapped)
15	3.00 (d, 9.9)	3.01 (d, 10.0)
19	0.94 (s)	0.94 (s)
20	1.664 (s)	1.664 (s)
21	1.660 (s)	1.660 (s)
22	5.25 (brs)	5.25 (d, 3.5)
23	2.67 (m)	2.67 (m)
24	3.84 (dd, 10.4, 6.7), 3.74 (dd, 10.4, 6.5)	3.83 (dd, 10.1, 6.6), 3.75 (dd, 10.1, 6.4)
25	1.28 (d, 7.0)	1.29 (d, 7.0)

[a] ^{1}H NMR (600 MHz) in Methanol-d_4, [b] ^{1}H NMR (800 MHz) in Methanol-d_4 (δ in ppm, *J* in Hz).

Table S77. ^{13}C NMR data of synthetic **9** and natural **9**.

No.	Synthetic **9**[a]	Natural **9**[b]	$\Delta\delta$	No.	Synthetic **9**[a]	Natural **9**[b]	$\Delta\delta$
	δ_C, type				δ_C, type		
1	50.0 C	49.8 C	+0.2	14	36.3 CH_2	36.3 CH_2	0.0
2	40.1 CH_2	40.2 CH_2	−0.1	15	46.9 CH	46.9 CH	0.0
3	122.7 CH	122.7 CH	0.0	16	153.2 C	153.0 C	+0.2
4	139.7 C	139.6 C	+0.1	17	149.0 C	149.4 C	−0.4
5	41.2 CH_2	41.2 CH_2	0.0	18	210.4 C	210.8 C	−0.4
6	25.2 CH_2	25.2 CH_2	0.0	19	17.9 CH_3	17.9 CH_3	0.0
7	124.1 CH	124.1 CH	0.0	20	15.4 CH_3	15.4 CH_3	0.0
8	135.7 C	135.7 C	0.0	21	17.4 CH_3	17.4 CH_3	0.0
9	34.5 CH_2	34.5 CH_2	0.0	22	110.5 CH_2	110.4 CH_2	+0.1
10	35.1 CH_2	35.1 CH_2	0.0	23	38.6 CH	38.6 CH	0.0
11	72.5 CH	72.5 CH	0.0	24	65.8 CH_2	65.9 CH_2	−0.1
12	157.1 C	157.2 C	−0.1	25	14.8 CH_3	14.9 CH_3	−0.1
13	70.1 CH	70.1 CH	0.0				

[a] ^{13}C NMR (150 MHz) in Methanol-d_4, [b] ^{13}C NMR (125 MHz) in Methanol-d_4 (δ in ppm).

Table S78. ^{1}H NMR data of synthetic **11** and natural **11**.

No.	Synthetic **11**[a]	Natural **11**[a]
	δ_H, mult (*J*)	δ_H, mult (*J*)
2	2.23 (overlapped), 1.95 (overlapped)	2.22 (overlapped), 1.95 (overlapped)
3	5.41 (overlapped)	5.41 (overlapped)
5	2.30 (overlapped), 2.25 (overlapped)	2.29 (overlapped), 2.24 (overlapped)
6	1.88 (m), 1.62 (m)	1.88 (m), 1.62 (m)
7	2.95 (dd, 7.3, 4.4)	2.95 (dd, 7.4, 4.4)
9	1.98 (overlapped), 1.02 (m)	1.98 (overlapped), 1.02 (m)
10	1.69 (m)	1.68 (m)
11	3.97 (t, 7.2)	3.97 (t, 7.6)
13	5.37 (overlapped)	5.37 (overlapped)
14	2.42 (m), 1.96 (overlapped)	2.42 (m), 1.96 (overlapped)
15	2.69 (dd, 10.5, 1.8)	2.69 (dd, 10.5, 2.0)
19	0.95 (s)	0.95 (s)
20	1.74 (s)	1.74 (s)
21	1.34 (s)	1.34 (s)
22	1.56 (s)	1.56 (s)
23	2.62 (m)	2.62 (m)
24	3.85 (dd, 10.3, 7.2), 3.70 (dd, 10.3, 6.4)	3.85 (dd, 10.4, 7.2), 3.70 (dd, 10.4, 6.4)
25	1.28 (d, 7.1)	1.28 (d, 7.1)

[a] ^{1}H NMR (500 MHz) in Methanol-d_4 (δ in ppm, *J* in Hz).

Table S79. ^{13}C NMR data of synthetic **11** and natural **11**.

No.	Synthetic **11**[a]	Natural **11**[a]	$\Delta\delta$	No.	Synthetic **11**[a]	Natural **11**[a]	$\Delta\delta$
	δ_C, type				δ_C, type		
1	50.7 C	50.7 C	0.0	14	30.1 CH_2	30.1 CH_2	0.0
2	40.3 CH_2	40.3 CH_2	0.0	15	51.3 CH	51.3 CH	0.0
3	123.3 CH	123.3 CH	0.0	16	152.0 C	152.0 C	0.0
4	137.4 C	137.4 C	0.0	17	149.1 C	149.1 C	0.0
5	36.6 CH_2	36.6 CH_2	0.0	18	209.9 C	209.9 C	0.0
6	25.3 CH_2	25.3 CH_2	0.0	19	16.8 CH_3	16.8 CH_3	0.0
7	64.0 CH	64.0 CH	0.0	20	17.1 CH_3	17.0 CH_3	+0.1
8	62.2 C	62.2 C	0.0	21	17.0 CH_3	17.0 CH_3	0.0
9	35.1 CH_2	35.1 CH_2	0.0	22	10.4 CH_3	10.4 CH_3	0.0
10	30.5 CH_2	30.5 CH_2	0.0	23	38.9 CH	38.9 CH	0.0
11	77.7 CH	77.7 CH	0.0	24	65.9 CH_2	65.9 CH_2	0.0
12	137.6 C	137.6 C	0.0	25	14.7 CH_3	14.7 CH_3	0.0
13	130.9 CH	130.9 CH	0.0				

[a] ^{13}C NMR (125 MHz) in Methanol-d_4 (δ in ppm).

Table S80. ^{1}H NMR data of synthetic **12** and natural **12**.

No.	Synthetic **12**[a]	Natural **12**[a]
	δ_H, mult (J)	δ_H, mult (J)
2	2.39 (dd, 13.4, 9.7), 1.83 (overlapped)	2.38 (dd, 12.9, 10.1), 1.82 (overlapped)
3	5.37 (t, 7.9)	5.36 (t, 8.0)
5	2.27 (overlapped), 2.06 (m)	2.26 (overlapped), 2.05 (m)
6	2.29 (overlapped), 2.22 (overlapped)	2.28 (overlapped), 2.21 (overlapped)
7	5.13 (t, 6.2)	5.12 (t, 6.0)
9	2.20 (overlapped), 2.01 (m)	2.19 (overlapped), 2.00 (m)
10	1.77 (m), 1.67 (overlapped)	1.75 (m), 1.66 (overlapped)
11	3.07 (t, 6.9)	3.06 (t, 6.9)
13	2.90 (dd, 7.3, 2.0)	2.89 (dd, 7.2, 1.6)
14	1.84 (overlapped), 1.57 (m)	1.83 (overlapped), 1.58 (m)
15	2.83 (dd, 8.5, 3.8)	2.82 (dd, 8.5, 3.6)
19	1.04 (s)	1.03 (s)
20	1.69 (s)	1.68 (s)
21	1.65 (s)	1.64 (s)
22	1.26 (s)	1.25 (s)
23	2.71 (m)	2.69 (m)
24	3.85 (dd, 10.5, 7.1), 3.71 (dd, 10.5, 6.2)	3.83 (dd, 9.8, 7.1), 3.71 (dd, 9.8, 6.0)
25	1.25 (d, 7.6)	1.24 (d, 8.5)

[a] ^{1}H NMR (500 MHz) in Methanol-d_4, [b] ^{1}H NMR (800 MHz) in Methanol-d_4 (δ in ppm, J in Hz).

Table S81. ^{13}C NMR data of synthetic **12** and natural **12**.

No.	Synthetic **12**[a]	Natural **12**[a]	$\Delta\delta$	No.	Synthetic **12**[a]	Natural **12**[a]	$\Delta\delta$
	δ_C, type				δ_C, type		
1	50.2 C	50.2 C	0.0	14	29.6 CH_2	29.6 CH_2	0.0
2	39.2 CH_2	39.3 CH_2	+0.1	15	49.4 CH	49.4 CH	0.0
3	122.5 CH	122.5 CH	0.0	16	151.7 C	151.5 C	+0.2
4	139.7 C	139.7 C	0.0	17	149.2 C	149.8 C	−0.6
5	41.1 CH_2	41.1 CH_2	0.0	18	209.4 C	209.7 C	−0.3
6	24.8 CH_2	24.8 CH_2	0.0	19	18.7 CH_3	18.7 CH_3	0.0
7	124.7 CH	124.7 CH	0.0	20	15.7 CH_3	15.7 CH_3	+0.1
8	134.8 C	134.8 C	0.0	21	16.5 CH_3	16.5 CH_3	0.0
9	34.7 CH_2	34.7 CH_2	0.0	22	10.9 CH_2	10.9 CH_2	0.0
10	31.6 CH_2	31.6 CH_2	0.0	23	38.4 CH	38.4 CH	0.0
11	76.6 CH	76.7 CH	−0.1	24	65.8 CH_2	65.9 CH_2	−0.1
12	65.2 C	65.2 C	0.0	25	14.7 CH_3	14.8 CH_3	−0.1
13	63.4 CH	63.4 CH	0.0				

[a] ^{13}C NMR (150 MHz) in Methanol-d_4, [b] ^{13}C NMR (125 MHz) in Methanol-d_4 (δ in ppm).

Table S82. ^{1}H NMR data of synthetic **13** and natural **13**.

No.	Synthetic **13**[a]	Natural **13**[a]
	δ_H, mult (J)	δ_H, mult (J)
2	2.03 (m)	2.03 (m)
3	5.47 (t, 7.5)	5.47 (t, 6.9)
5	2.24 (overlapped)	2.24 (overlapped)
6	2.09 (m), 1.59 (m)	2.09 (m), 1.59 (m)
7	3.57 (dd, 10.3, 1.3)	3.57 (dd, 10.5, 1.3)
9	1.85 (m), 1.75 (overlapped)	1.85 (m), 1.75 (overlapped)
10	2.23 (overlapped), 1.74 (overlapped)	2.23 (overlapped), 1.74 (overlapped)
11	4.44 (d, 7.2)	4.43 (d, 7.8)
13	5.59 (t, 4.5)	5.58 (t, 4.9)
14	2.47 (dd, 17.5, 2.5), 1.75 (overlapped)	2.47 (dd, 16.8, 1.7), 1.75 (overlapped)
15	2.54 (dd, 12.1, 1.5)	2.54 (dd, 11.9, 1.9)
19	1.04 (s)	1.04 (s)
20	1.69 (s)	1.69 (s)
21	1.13 (s)	1.13 (s)
22	1.53 (s)	1.53 (s)
23	2.59 (m)	2.59 (m)
24	3.83 (dd, 10.0, 7.5), 3.69 (dd, 10.0, 6.5)	3.82 (dd, 9.7, 7.2), 3.69 (dd, 9.7, 6.5)
25	1.26 (d, 7.0)	1.26 (d, 7.1)

[a] ^{1}H NMR (800 MHz) in Methanol-d_4 (δ in ppm, J in Hz).

Table S83. ^{13}C NMR data of synthetic **13** and natural **13**.

No.	Synthetic **13**[a]	Natural **13**[a]	$\Delta\delta$	No.	Synthetic **13**[a]	Natural **13**[a]	$\Delta\delta$
	δ_C, type				δ_C, type		
1	50.0 C	50.0 C	0.0	14	29.4 CH_2	29.4 CH_2	0.0
2	39.9 CH_2	39.9 CH_2	0.0	15	50.8 CH	50.7 CH	+0.1
3	120.5 CH	120.6 CH	−0.1	16	152.0 C	151.9 C	+0.1
4	139.3 C	139.2 C	+0.1	17	149.2 C	149.2 C	0.0
5	34.0 CH_2	34.0 CH_2	0.0	18	210.0 C	210.3 C	−0.3
6	30.4 CH_2	30.4 CH_2	0.0	19	16.7 CH_3	16.8 CH_3	−0.1
7	77.4 CH	77.4 CH	0.0	20	19.4 CH_3	19.4 CH_3	0.0
8	87.3 C	87.3 C	0.0	21	19.7 CH_3	19.7 CH_3	0.0
9	36.4 CH_2	36.4 CH_2	0.0	22	14.5 CH_2	14.5 CH_2	0.0
10	30.9CH_2	30.9CH_2	0.0	23	38.9 CH	38.9 CH	0.0
11	83.6 CH	83.6 CH	0.0	24	65.9 CH_2	66.0 CH_2	−0.1
12	137.4 C	137.3 C	+0.1	25	14.6 CH_3	14.7 CH_3	−0.1
13	123.5 CH	123.5 CH	0.0				

[a] ^{13}C NMR (200 MHz) in Methanol-d_4 (δ in ppm).

S7. *NMR data comparison of synthetic and natural 2, 3*

Table S84. ^{1}H NMR data of synthetic **2** and natural **2**.

No.	Synthetic **2**[a]	Natural **2**[b]
	δ_H, mult (J)	δ_H, mult (J)
2	2.22 (dd, 14.2, 9.0), 1.77 (overlapped)	2.23 (dd, 14.3, 9.1), 1.78 (overlapped)
3	5.17 (m)	5.16 (m)
5	2.12 (m)	2.11 (m)
6	1.95 (overlapped), 1.79 (overlapped)	1.95 (overlapped), 1.80 (overlapped)
7	4.14 (dd, 7.9, 4.7)	4.13 (dd, 7.9, 4.7)
9	2.27 (m), 2.05 (m)	2.28 (m), 2.05 (m)
10	1.83 (m), 1.63 (overlapped)	1.84 (m), 1.64 (overlapped)
11	4.08 (t, 6.6)	4.07 (t, 6.5)
13	5.36 (dd, 8.9, 2.0)	5.36 (dd, 9.0, 2.3)
14	2.40 (m), 1.95 (overlapped)	2.39 (m), 1.95 (overlapped)
15	2.62 (overlapped)	2.63 (overlapped)
19	0.95 (s)	0.96 (s)
20	1.66 (s)	1.67 (s)
21	5.19 (d, 1.0), 5.15 (d, 1.0)	5.18 (d, 1.1), 5.14 (d, 1.1)
22	1.62 (s)	1.63 (s)
23	2.61 (overlapped)	2.61 (overlapped)
24	3.82 (dd, 10.2, 7.0), 3.70 (dd, 10.3, 6.3)	3.82 (dd, 10.4, 7.1), 3.69 (dd, 10.4, 6.5)
25	1.27 (d, 7.1)	1.28 (d, 7.1)

[a] ^{1}H NMR (500 MHz) in Methanol-d_4, [b] ^{1}H NMR (800 MHz) in Methanol-d_4, (δ in ppm, J in Hz).

Table S85. ^{13}C NMR data of synthetic **2** and natural **2**.

No.	Synthetic **2**[a]	Natural **2**[b]	$\Delta\delta$	No.	Synthetic **2**[a]	Natural **2**[b]	$\Delta\delta$
	δ_C, type				δ_C, type		
1	49.8 C	49.8 C	0.0	14	30.1 CH_2	30.1 CH_2	0.0
2	40.2 CH_2	40.2 CH_2	0.0	15	51.4 CH	51.4 CH	0.0
3	122.1 CH	122.1 CH	0.0	16	152.1 C	152.2 C	−0.1
4	138.7 C	138.7 C	0.0	17	149.1 C	149.0 C	+0.1
5	36.2 CH_2	36.2 CH_2	0.0	18	210.3 C	210.1 C	+0.2
6	30.0 CH_2	30.0 CH_2	0.0	19	17.1 CH_3	17.1 CH_3	0.0
7	87.2 CH	87.3 CH	−0.1	20	16.4 CH_3	16.4 CH_3	0.0
8	147.1 C	147.1 C	0.0	21	112.9 CH_2	112.9 CH_2	0.0
9	31.2 CH_2	31.2 CH_2	0.0	22	11.3 CH_3	11.3 CH_3	0.0
10	32.7 CH_2	32.7 CH_2	0.0	23	38.8 CH	38.9 CH	−0.1
11	77.3 CH	77.3 CH	0.0	24	65.9 CH_2	65.8 CH_2	+0.1
12	138.5 C	138.5 C	0.0	25	14.7 CH_3	14.7 CH_3	0.0
13	128.7 CH	128.7 CH	0.0				

[a] ^{13}C NMR (125 MHz) in Methanol-d_4, [b] ^{13}C NMR (200 MHz) in Methanol-d_4 (δ in ppm).

Table S86. ^{1}H NMR data of synthetic **3** and natural **3**.

No.	Synthetic **3**[a]	Natural **3**[a]
	δ_H, mult (J)	δ_H, mult (J)
2	2.14 (dd, 14.1, 7.4), 1.90 (overlapped)	2.15 (dd, 14.1, 7.4), 1.90 (overlapped)
3	5.24 (m)	5.24 (m)
5	2.77 (d, 6.5)	2.77 (d, 6.5)
6	5.68 (dt,15.8, 6.5)	5.68 (dt,16.0, 6.6)
7	5.48 (d,15.8, 1.0)	5.49 (dt,16.0, 1.1)
9	1.98 (m), 1.52 (m)	1.99 (m), 1.53 (m)
10	1.65 (overlapped), 1.59 (overlapped)	1.66 (overlapped), 1.59 (overlapped)
11	3.92 (dd, 8.8, 3.5)	3.92 (dd, 9.1, 3.7)
13	5.28 (dd, 9.0, 2.4)	5.28 (dd, 8.7, 2.1)
14	2.38 (m), 1.88 (overlapped)	2.38 (m), 1.87 (overlapped)
15	2.56 (dd, 11.0, 1.5)	2.55 (dd, 11.2, 1.8)
19	0.98 (s)	0.98 (s)
20	1.66 (s)	1.67 (s)
21	1.35 (s)	1.36 (s)
22	1.59 (s)	1.59 (s)
23	2.60 (m)	2.61 (m)
24	3.83 (dd, 10.3, 7.2), 3.69 (dd, 10.3, 6.4)	3.83 (dd, 10.4, 7.1), 3.70 (dd, 10.4, 6.4)
25	1.26 (d, 7.1)	1.26 (d, 7.1)

[a] ^{1}H NMR (500 MHz) in Methanol-d_4 (δ in ppm, J in Hz).

Table S87. ^{13}C NMR data of synthetic **3** and natural **3**.

No.	Synthetic **3**[a]	Natural **3**[a]	$\Delta\delta$	No.	Synthetic **3**[a]	Natural **3**[a]	$\Delta\delta$
	δ_C, type				δ_C, type		
1	50.0 C	49.9 C	+0.1	14	30.1 CH_2	30.1 CH_2	0.0
2	40.2 CH_2	40.2 CH_2	0.0	15	51.0 CH	51.0 CH	0.0
3	121.3 CH	121.3 CH	0.0	16	152.1 C	152.1 C	0.0
4	138.5 C	138.5 C	0.0	17	148.9 C	149.0 C	−0.1
5	43.2 CH_2	43.2 CH_2	0.0	18	210.1 C	210.3 C	−0.2
6	128.2 CH	128.2 CH	0.0	19	16.5 CH_3	16.5 CH_3	0.0
7	135.0 CH	135.0 CH	0.0	20	17.9 CH_3	17.9 CH_3	0.0
8	85.0 C	85.0 C	0.0	21	24.5 CH_3	24.5 CH_3	0.0
9	35.0 CH_2	34.9 CH_2	0.0	22	11.9 CH_3	11.9 CH_3	0.0
10	30.8 CH_2	30.8 CH_2	0.0	23	38.9 CH	38.8 CH	+0.1
11	79.8 CH	79.8 CH	0.0	24	65.9 CH_2	65.9 CH_2	0.0
12	139.2 C	139.2 C	0.0	25	14.7 CH_3	14.7 CH_3	0.0
13	127.1 CH	127.1 CH	0.0				

[a] ^{13}C NMR (125 MHz) in Methanol-d_4 (δ in ppm).

S8. *NMR data comparison of synthetic and natural 4, 5, 6*

Table S88. ^{1}H NMR data of synthetic **4** and natural **4**.

No.	Synthetic **4**[a]	Natural **4**[b]
	δ_H, mult (J)	δ_H, mult (J)
2	2.22 (overlapped), 1.79 (overlapped)	2.23 (overlapped), 1.80 (overlapped)
3	5.15 (overlapped)	5.16 (overlapped)
5	2.10 (t, 7.3)	2.11 (t, 7.2)
6	1.85 (overlapped), 1.69 (m)	1.86 (overlapped), 1.70 (m)
7	3.95 (t, 5.9)	3.96 (t, 5.9)
9	2.24 (overlapped), 2.01 (overlapped)	2.25 (overlapped), 2.02 (overlapped)
10	1.83 (overlapped), 1.64 (overlapped)	1.84 (overlapped), 1.64 (overlapped)
11	4.08 (t, 6.6)	4.09 (t, 6.6)
13	5.36 (dd, 9.1, 2.7)	5.37 (dd, 9.1, 2.7)
14	2.37 (m), 1.99 (overlapped)	2.38 (m), 2.00 (overlapped)
15	2.61 (overlapped)	2.62 (overlapped)
19	0.94 (s)	0.95 (s)
20	1.64 (s)	1.65 (s)
21	5.18 (d, 0.8), 5.03 (d, 0.8)	5.19 (d, 1.1), 5.04 (d, 1.1)
22	1.60 (s)	1.61 (s)
23	2.61 (overlapped)	2.62 (overlapped)
24	3.82 (dd, 10.4, 7.1), 3.68 (dd, 10.4, 6.5)	3.83 (dd, 10.4, 7.0), 3.70 (dd, 10.4, 6.4)
25	1.26 (d, 7.1)	1.27 (d, 7.1)

[a] ^{1}H NMR (600 MHz) in Methanol-d_4, [b] ^{1}H NMR (500 MHz) in Methanol-d_4, (δ in ppm, J in Hz).

Table S89. ^{13}C NMR data of synthetic **4** and natural **4**.

No.	Synthetic **4**[a]	Natural **4**[b]	$\Delta\delta$	No.	Synthetic **4**[a]	Natural **4**[b]	$\Delta\delta$
	δ_C, type				δ_C, type		
1	49.9 C	49.8 C	−0.1	14	30.0 CH_2	30.0 CH_2	0.0
2	40.0 CH_2	40.0 CH_2	0.0	15	51.4 CH	51.3 CH	+0.1
3	121.7 CH	121.7 CH	0.0	16	152.2 C	152.2 C	0.0
4	139.0 C	139.0 C	0.0	17	148.9 C	149.1 C	−0.2
5	35.9 CH_2	35.9 CH_2	0.0	18	210.1 C	210.2 C	−0.1
6	34.5 CH_2	34.5 CH_2	0.0	19	17.1 CH_3	17.2 CH_3	−0.1
7	73.9 CH	73.9 CH	0.0	20	16.7 CH_3	16.7 CH_3	0.0
8	151.3 C	151.3 C	0.0	21	110.6 CH_2	110.6 CH_2	0.0
9	30.7 CH_2	30.7 CH_2	0.0	22	11.4 CH_3	11.4 CH_3	0.0
10	33.0 CH_2	33.0 CH_2	0.0	23	38.8 CH	38.8 CH	0.0
11	77.3 CH	77.3 CH	0.0	24	65.8 CH_2	65.9 CH_2	−0.1
12	138.6 C	138.6 C	0.0	25	14.7 CH_3	14.8 CH_3	−0.1
13	128.6 CH	128.6 CH	0.0				

[a] ^{13}C NMR (150 MHz) in Methanol-d_4, [b] ^{13}C NMR (125 MHz) in Methanol-d_4 (δ in ppm).

Table S90. ^{1}H NMR data of synthetic **5** and natural **5**.

No.	Synthetic **5**[a]	Natural **5**[b]
	δ_H, mult (J)	δ_H, mult (J)
2	2.18 (overlapped), 1.79 (overlapped)	2.18 (overlapped), 1.79 (overlapped)
3	5.19 (t, 6.8)	5.20 (t, 7.0)
5	2.03 (overlapped), 1.98 (overlapped)	2.03 (overlapped), 1.99 (overlapped)
6	1.76 (overlapped), 1.56 (m)	1.76 (overlapped), 1.56 (m)
7	4.03 (dd, 8.0, 6.1)	4.03 (dd, 8.0, 6.0)
9	2.22 (overlapped), 2.17 (overlapped)	2.22 (overlapped), 2.17 (overlapped)
10	1.78 (overlapped), 1.66 (m)	1.78 (overlapped), 1.65 (m)
11	4.08 (dd, 7.5, 5.6)	4.09 (dd, 7.5, 5.5)
13	5.37 (dd, 9.2, 2.5)	5.37 (dd, 9.1, 2.7)
14	2.38 (m), 1.95 (m)	2.38 (m), 1.95 (m)
15	2.61 (overlapped)	2.63 (overlapped)
19	0.94 (s)	0.93 (s)
20	1.63 (s)	1.63 (s)
21	5.07 (d, 1.4), 5.02 (d, 1.4)	5.07 (d, 1.6), 5.02 (d, 1.6)
22	1.61 (s)	1.61 (s)
23	2.61 (overlapped)	2.63 (overlapped)
24	3.83 (dd, 10.5, 7.1), 3.72 (dd, 10.5, 6.5)	3.80 (dd, 9.5, 7.2), 3.72 (dd, 9.5, 5.9)
25	1.26 (d, 7.1)	1.27 (d, 7.1)

[a] ^{1}H NMR (600 MHz) in Methanol-d_4, [b] ^{1}H NMR (800 MHz) in Methanol-d_4, (δ in ppm, J in Hz).

Table S91. ^{13}C NMR data of synthetic **5** and natural **5**.

No.	Synthetic **5**[a]	Natural **5**[b]	$\Delta\delta$	No.	Synthetic **5**[a]	Natural **5**[b]	$\Delta\delta$
	δ_C, type				δ_C, type		
1	50.2 C	50.1 C	+0.1	14	30.0 CH_2	30.1 CH_2	−0.1
2	39.5 CH_2	39.5 CH_2	0.0	15	51.2 CH	51.1 CH	+0.1
3	121.4 CH	121.5 CH	−0.1	16	152.1 C	151.8 C	+0.3
4	139.1 C	139.1 C	0.0	17	148.9 C	149.6 C	−0.7
5	36.5 CH_2	36.5 CH_2	0.0	18	210.0 C	210.6 C	−0.6
6	35.6 CH_2	35.6 CH_2	0.0	19	17.0 CH_3	17.1 CH_3	−0.1
7	77.3 CH	77.3 CH	0.0	20	16.9 CH_3	16.9 CH_3	0.0
8	151.3 C	151.3 C	0.0	21	113.7 CH_2	113.7 CH_2	0.0
9	26.8 CH_2	26.8 CH_2	0.0	22	11.5 CH_3	11.5 CH_3	0.0
10	34.2 CH_2	34.2 CH_2	0.0	23	38.8 CH	38.8 CH	0.0
11	76.7 CH	76.7 CH	0.0	24	65.8 CH_2	66.1 CH_2	−0.3
12	138.7 C	138.6 C	0.0	25	14.7 CH_3	14.8 CH_3	−0.1
13	128.5 CH	128.6 CH	−0.1				

[a] ^{13}C NMR (150 MHz) in Methanol-d_4, [b] ^{13}C NMR (200 MHz) in Methanol-d_4 (δ in ppm).

Table S92. ^{1}H NMR data of synthetic **6** and natural **6**.

No.	Synthetic **6**[a]	Natural **6**[b]
	δ_H, mult (*J*)	δ_H, mult (*J*)
2	2.07 (dd, 14.1, 6.3), 1.93 (dd, 14.1, 8.6)	2.08 (dd, 14.1, 6.4), 1.95 (dd, 14.1, 8.6)
3	5.26 (m)	5.27 (m)
5	2.79 (m)	2.79 (m)
6	5.75 (m)	5.76 (m)
7	5.48 (d, 15.4)	5.49 (d, 15.5)
9	1.74 (m), 1.64 (overlapped)	1.75 (m), 1.65 (overlapped)
10	1.62 (overlapped), 1.51 (m)	1.63 (overlapped), 1.52 (m)
11	3.88 (dd, 9.9, 2.5)	3.89 (dd, 9.8, 2.6)
13	5.20 (dd, 9.5, 1.5)	5.21 (dd, 9.5, 1.6)
14	2.33 (m), 1.87 (m)	2.35 (m), 1.88 (m)
15	2.50 (dd, 11.5, 1.9)	2.51 (dd, 11.4, 2.0)
19	0.99 (s)	1.00 (s)
20	1.66 (s)	1.68 (s)
21	1.26 (s)	1.27 (s)
22	1.56 (s)	1.57 (s)
23	2.59 (m)	2.60 (m)
24	3.82 (dd, 10.4, 7.2), 3.68 (dd, 10.4, 6.5)	3.83 (dd, 10.4, 7.2), 3.69 (dd, 10.4, 6.4)
25	1.24 (d, 7.1)	1.25 (d, 7.1)

[a] ^{1}H NMR (600 MHz) in Methanol-d_4, [b] ^{1}H NMR (500 MHz) in Methanol-d_4, (δ in ppm, *J* in Hz).

Table S93. ^{13}C NMR data of synthetic **6** and natural **6**.

No.	Synthetic **6**[a]	Natural **6**[b]	$\Delta\delta$	No.	Synthetic **6**[a]	Natural **6**[b]	$\Delta\delta$
	δ_C, type				δ_C, type		
1	49.8 C	49.8 C	0.0	14	30.0 CH_2	30.0 CH_2	0.0
2	39.9 CH_2	39.9 CH_2	0.0	15	50.9 CH	50.9 CH	0.0
3	120.9 CH	120.9 CH	0.0	16	152.1 C	152.1 C	0.0
4	138.4 C	138.4 C	0.0	17	148.8 C	148.8 C	0.0
5	42.9 CH_2	42.9 CH_2	0.0	18	210.1 C	210.1 C	0.0
6	126.1 CH	126.1 CH	0.0	19	16.5 CH_3	16.5 CH_3	0.0
7	138.0 CH	138.0 CH	0.0	20	18.0 CH_3	18.0 CH_3	0.0
8	74.3 C	74.3 C	0.0	21	30.4 CH_3	30.4 CH_3	0.0
9	39.0 CH_2	39.0 CH_2	0.0	22	11.4 CH_3	11.4 CH_3	0.0
10	30.6 CH_2	30.6 CH_2	0.0	23	38.8 CH	38.8 CH	0.0
11	80.5 CH	80.4 CH	+0.1	24	65.8 CH_2	65.9 CH_2	−0.1
12	139.1 C	139.1 C	0.0	25	14.6 CH_3	14.6 CH_3	0.0
13	127.4 CH	127.4 CH	0.0				

[a] ^{13}C NMR (150 MHz) in Methanol-d_4, [b] ^{13}C NMR (125 MHz) in Methanol-d_4 (δ in ppm).

S9. ***NMR, HRESIMS, ECD, UV, ORD, and IR spectra (next page)***

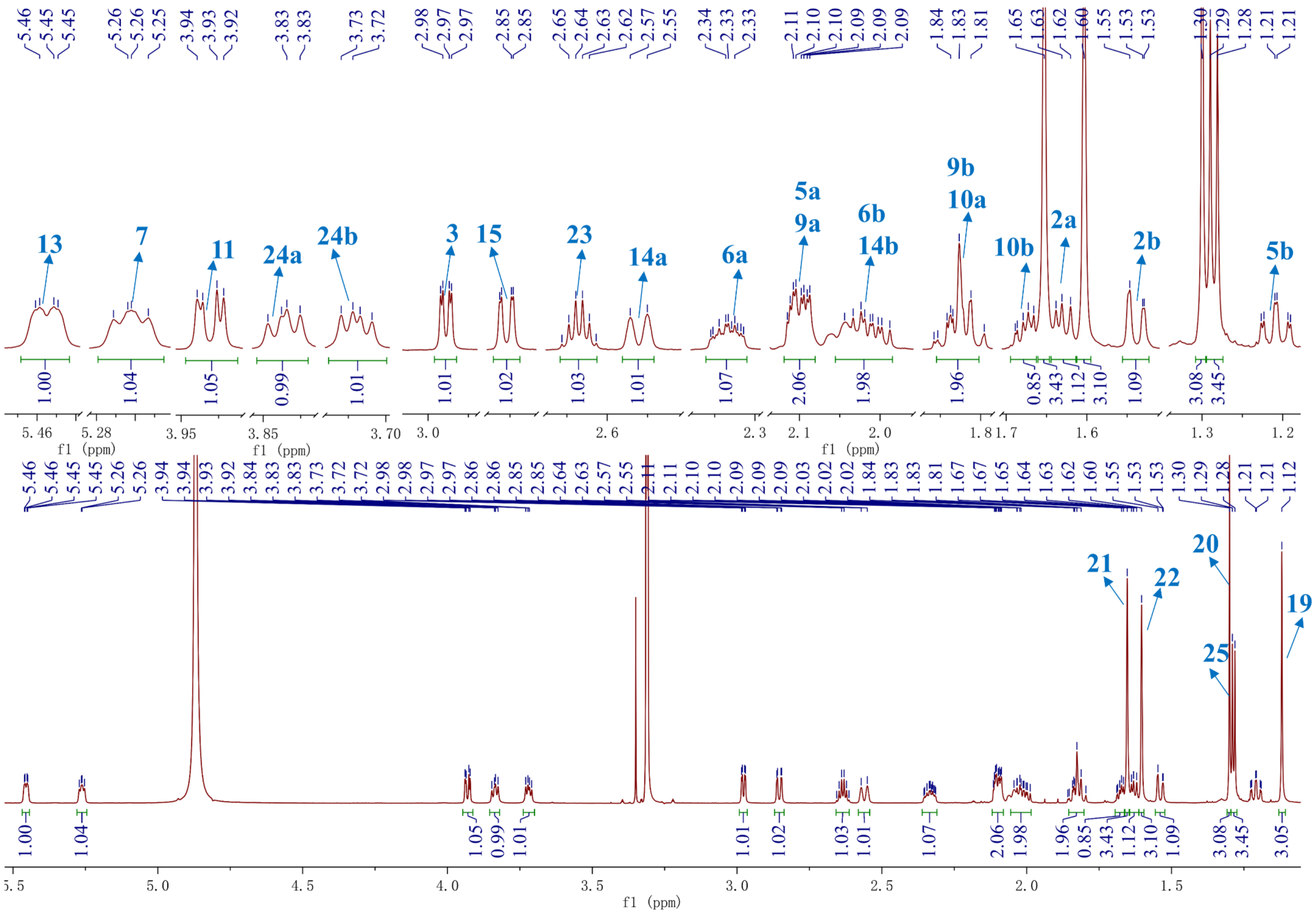

Figure S18. ^{1}H NMR spectrum of arthritacin A (**1**) (Methanol-d_4).

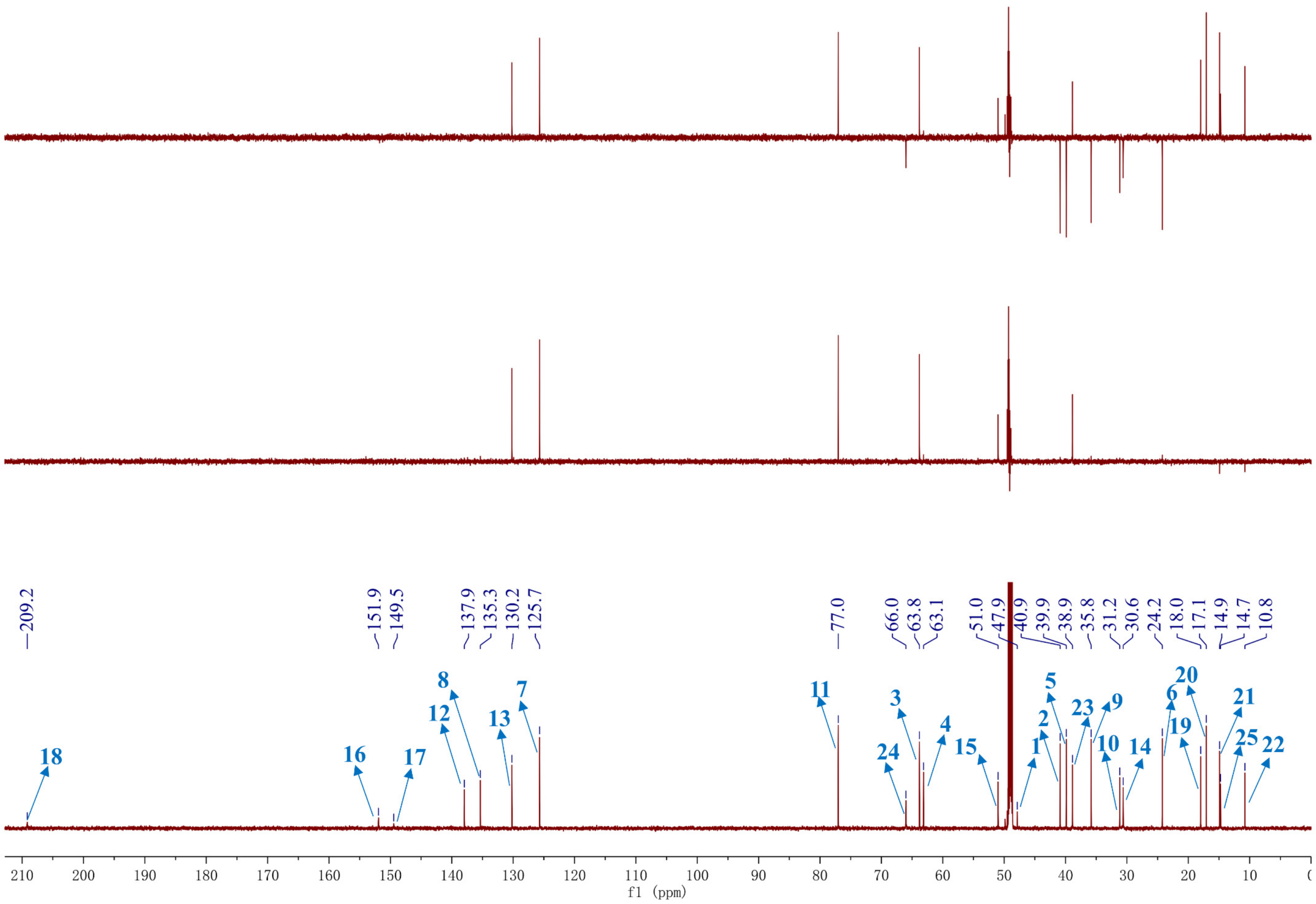

Figure S19. ^{13}C NMR and DEPT spectrum of arthritacin A (**1**) (Methanol-d_4).

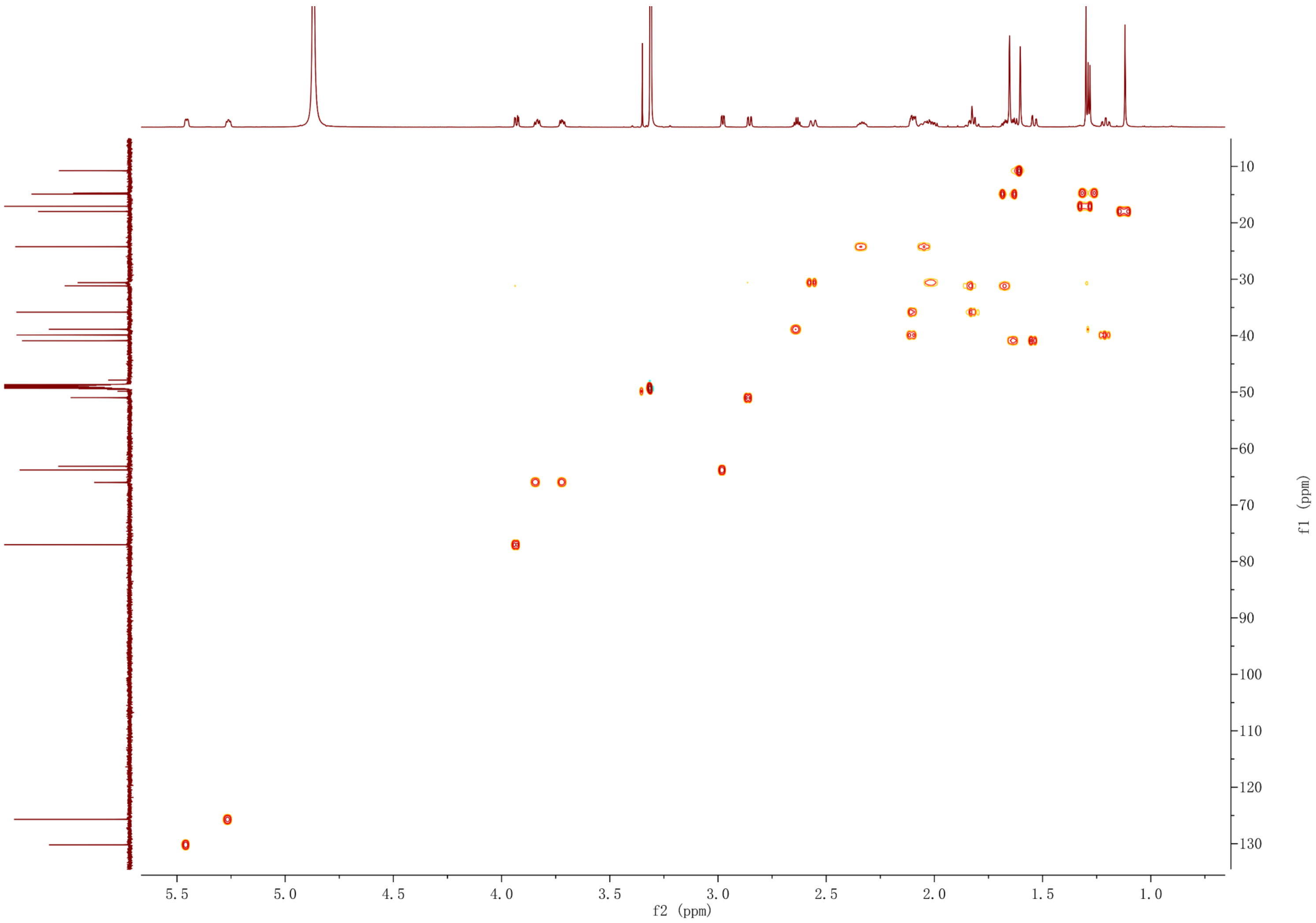

Figure S20. HSQC spectrum of arthritacin A (**1**) (Methanol-d_4).

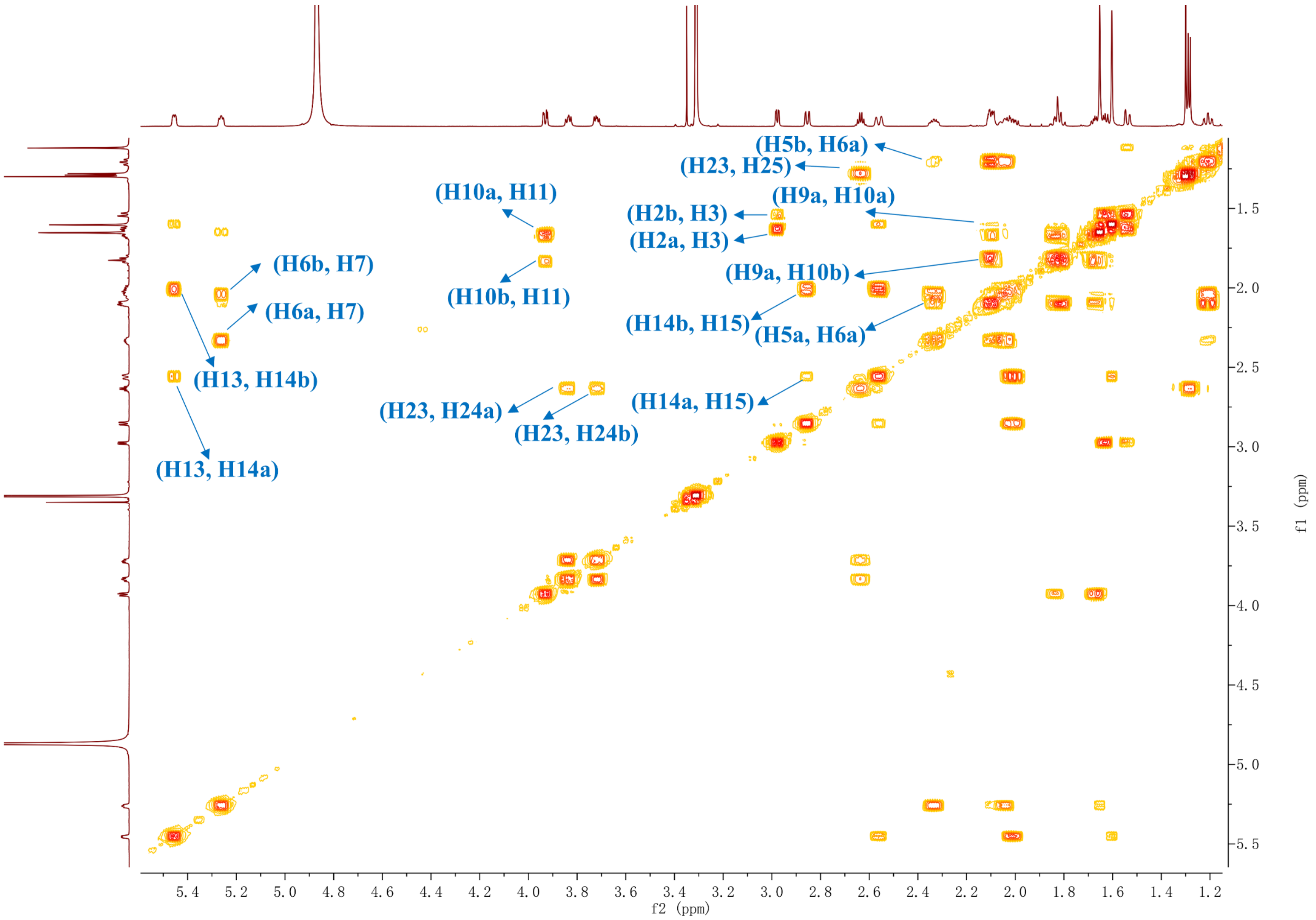

Figure S21. ^{1}H-^{1}H COSY spectrum of arthritacin A (**1**) (Methanol-d_4) and the selected cross-peaks (blue arrows and texts).

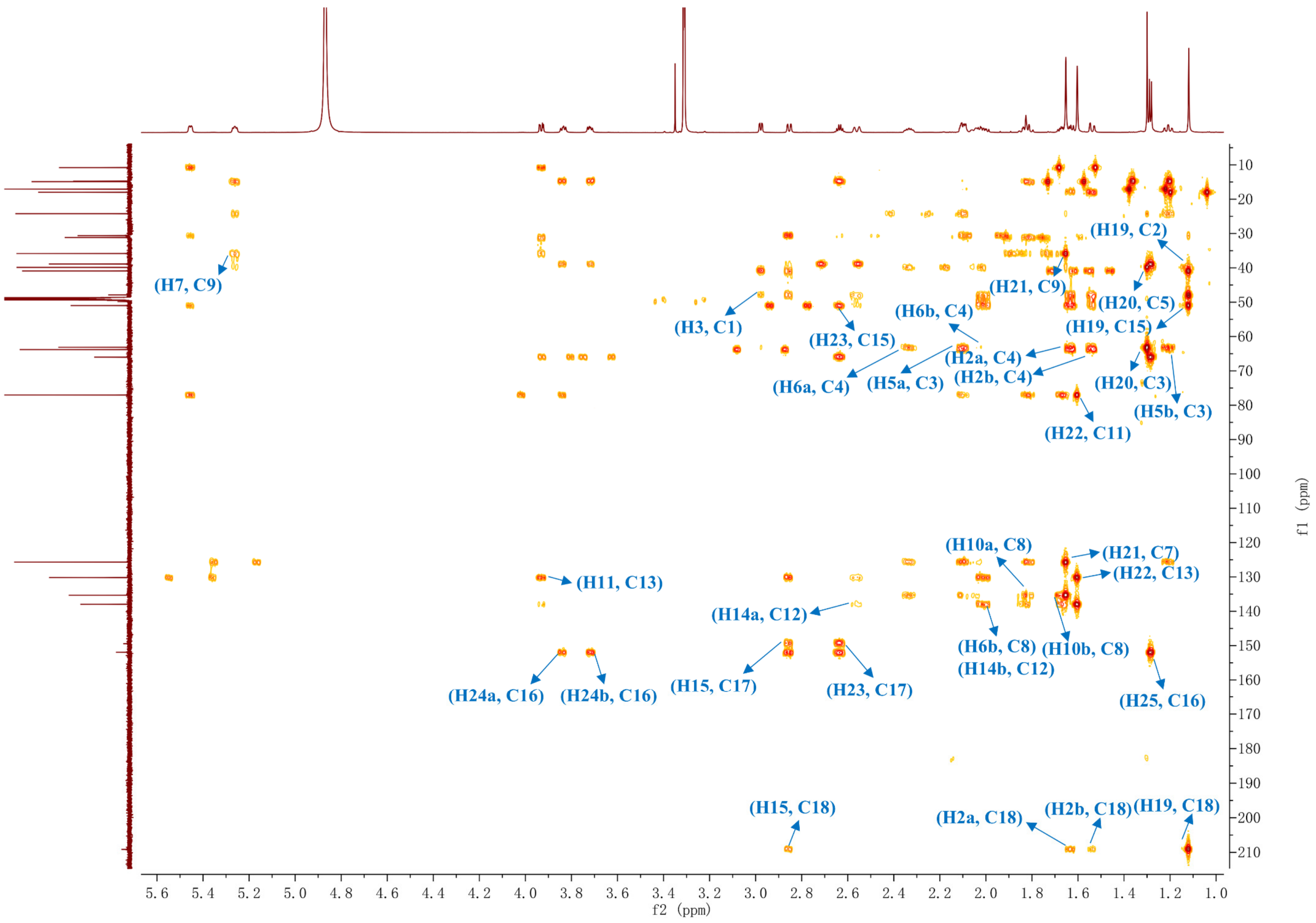

Figure S22. HMBC spectrum of arthritacin A (**1**) (Methanol-d_4) and the selected correlations (blue arrows and texts).

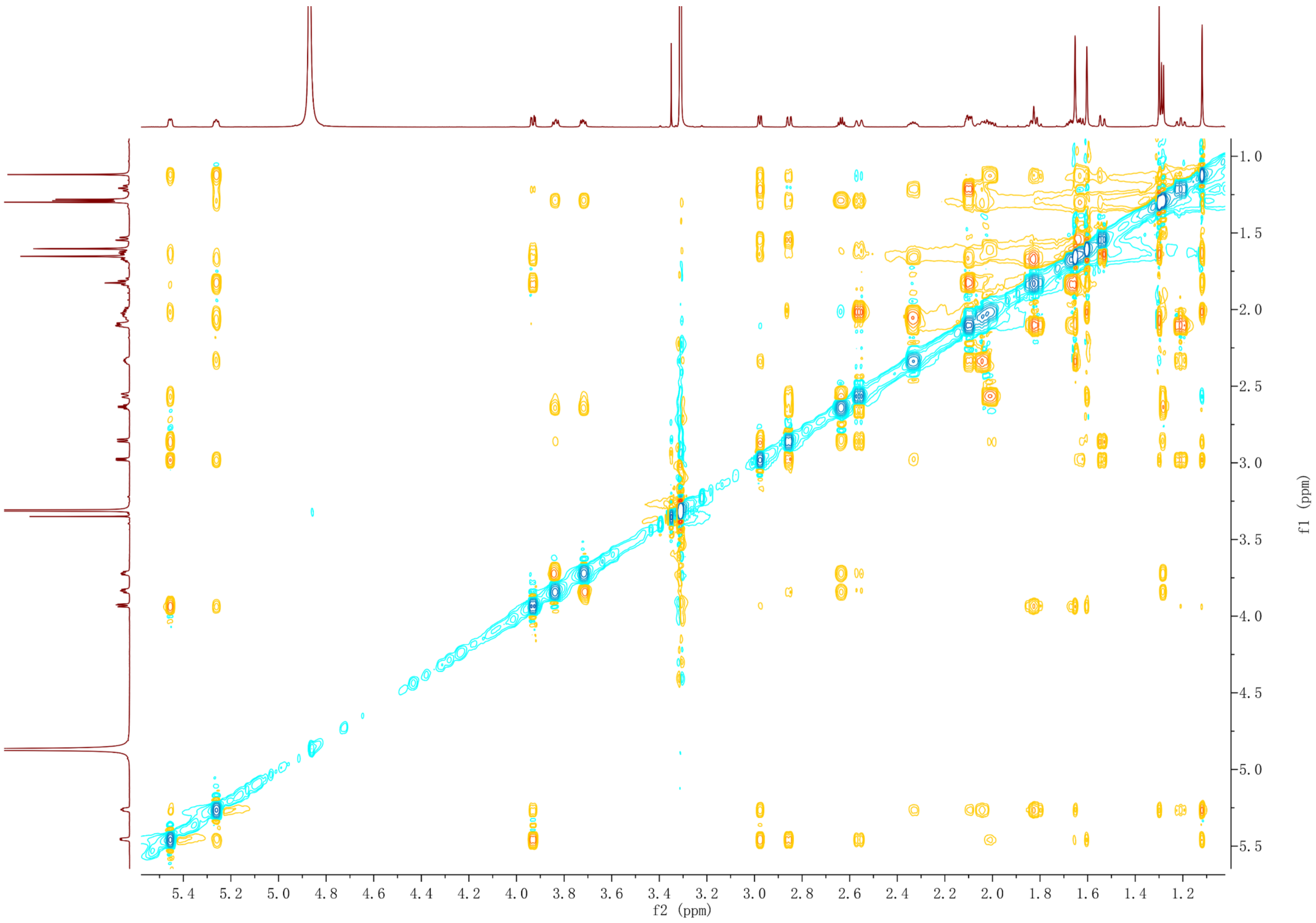

Figure S23. ROESY spectrum of arthritacin A (**1**) (Methanol-d_4) and the selected correlations (blue arrows and texts).

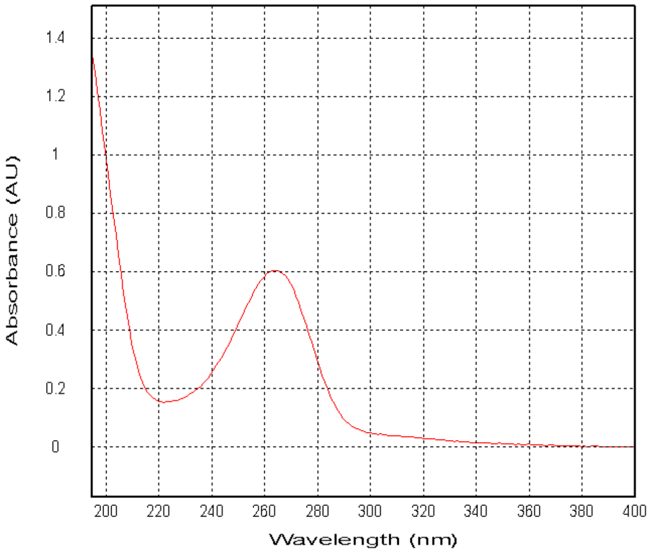

Figure S24. UV spectrum of arthritacin A (**1**).

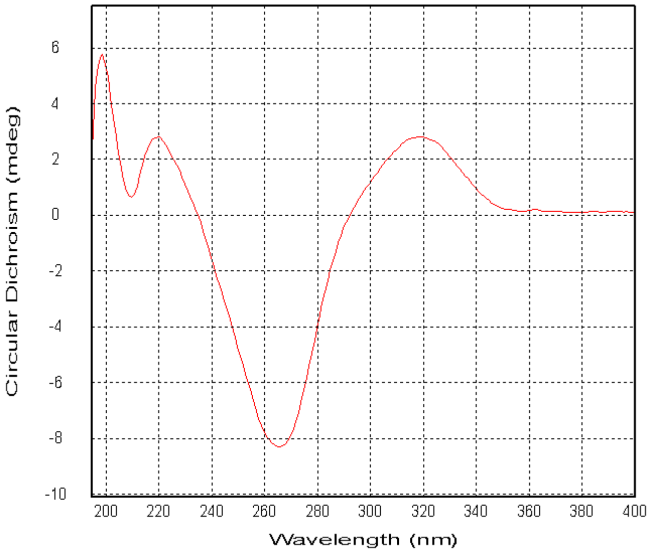

Figure S25. ECD spectrum of arthritacin A (**1**).

Rudolph Research Analytical

This sample was measured on an Autopol VI, Serial #91058
Manufactured by Rudolph Research Analytical, Hackettstown, NJ, USA.

Measurement Date : Friday, 28-MAY-2021

Set Temperature : OFF

Time Delay : Disabled

Delay between Measurement : Disabled

n	Average	Std.Dev.	% RSD	Maximum	Minimum
5	-19.89	0.28	-1.40	-19.64	-20.36

S.No	Sample ID	Time	Result	Scale	OR °Arc	WLG.nm	Lg.mm	Conc.g/100ml	Temp.
1	SHSJ29	10:49:14 AM	-19.82	SR	-0.0218	589	100.00	0.110	25.4
2	SHSJ29	10:49:23 AM	-19.64	SR	-0.0216	589	100.00	0.110	25.4
3	SHSJ29	10:49:31 AM	-19.73	SR	-0.0217	589	100.00	0.110	25.4
4	SHSJ29	10:49:39 AM	-19.91	SR	-0.0219	589	100.00	0.110	25.4
5	SHSJ29	10:49:47 AM	-20.36	SR	-0.0224	589	100.00	0.110	25.4

Figure S26. ORD report of arthritacin A (**1**).

User Spectra

Fragmentor Voltage	Collision Energy	Ionization Mode
85	0	ESI

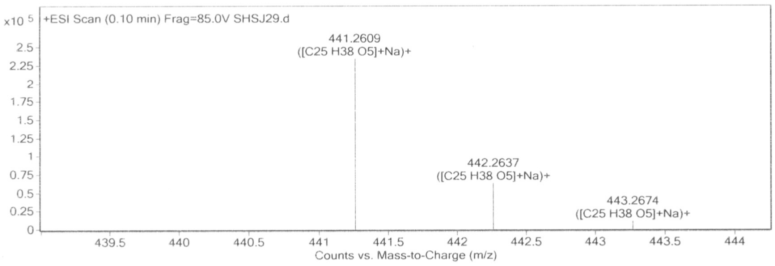

Peak List

m/z	z	Abund	Formula	Ion
64.0163	1	50962.85		
101.0036	1	42315.71		
203.147	1	22077.94		
401.2684	1	48142.01		
441.2609	1	234358.31	C25 H38 O5	(M+Na)+
442.2637	1	62559.24	C25 H38 O5	(M+Na)+
457.2363	1	29682.72		
477.3308	1	20745.66		
859.5311	1	74616.79		
860.5338	1	40277.12		

Formula Calculator Element Limits

Element	Min	Max
C	3	60
H	0	120
O	0	30

Formula Calculator Results

Formula	CalculatedMass	CalculatedMz	Mz	Diff. (mDa)	Diff. (ppm)	DBE
C25 H38 O5	418.2719	441.2611	441.2609	0.20	0.45	7.0000

--- End Of Report ---

Figure S27. HRESIMS spectrum of arthritacin A (**1**).

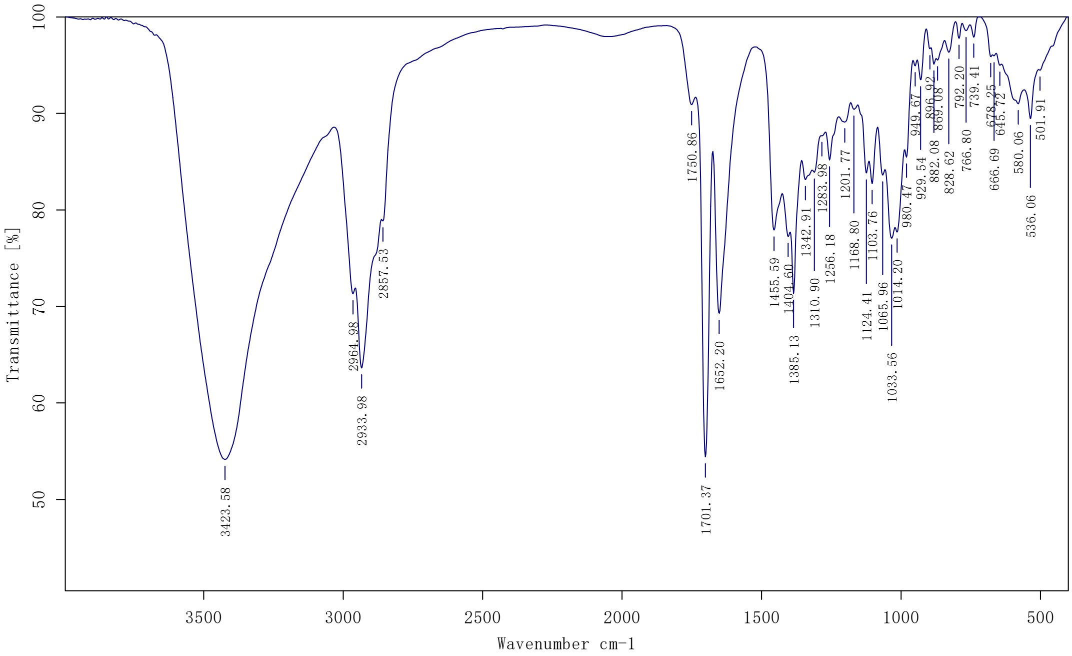

Sample Name: SHSJ29
Sample Form: KBr
Path of File: E:\data
Date of Measurement: 2025/7/3

Resolution: 4
Aperture Setting: 6 mm
Number of Background Scans: 16
Number of Sample Scans: 16

Beamsplitter Setting: KBr
Source Setting: MIR
Instrument Type: BRUKER VERTEX 70
Soft Version: OPUS8.1

Figure S28. IR spectrum of arthritacin A (**1**).

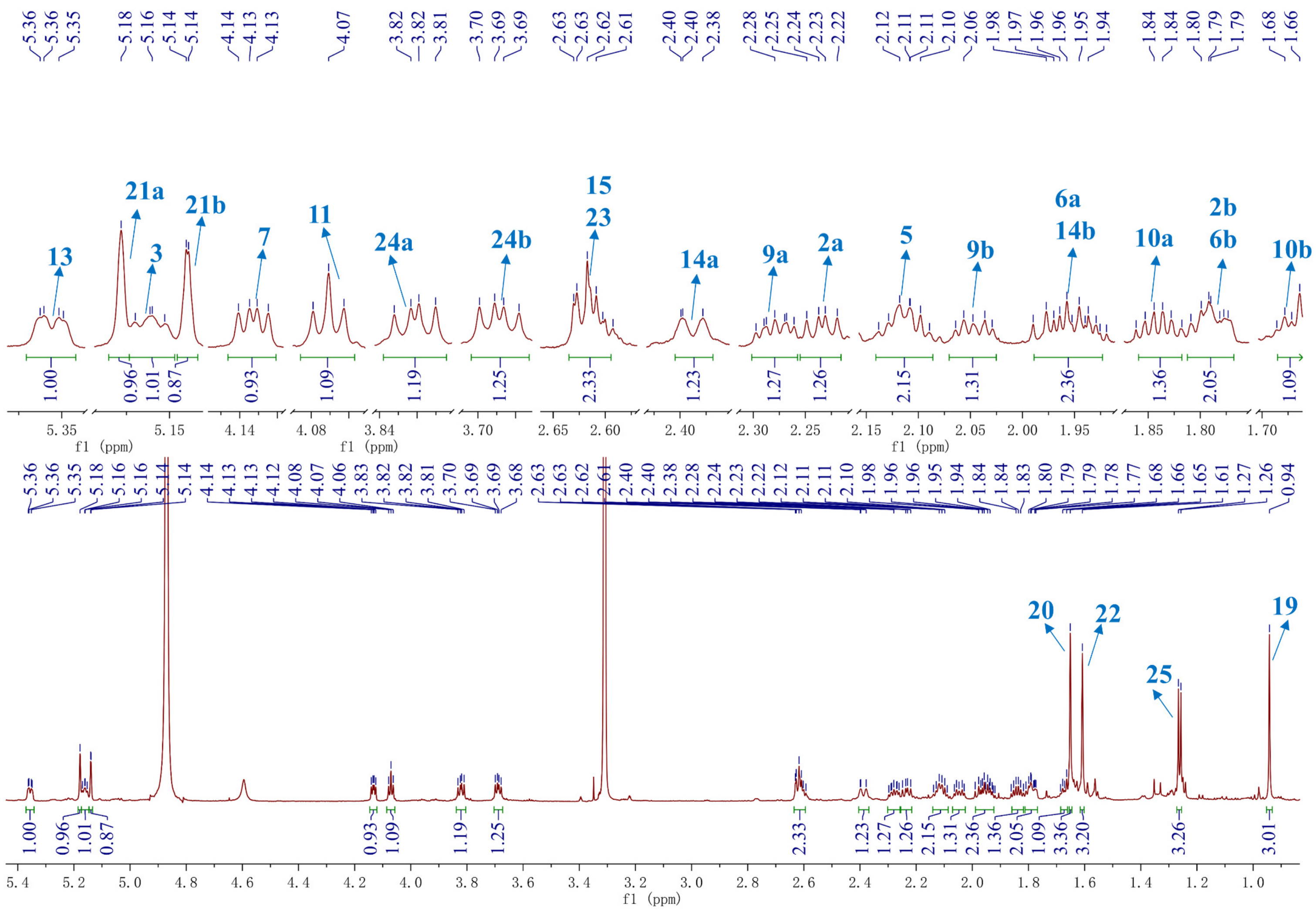

Figure S29. ^{1}H NMR spectrum of arthritacin B (**2**) (Methanol-d_4).

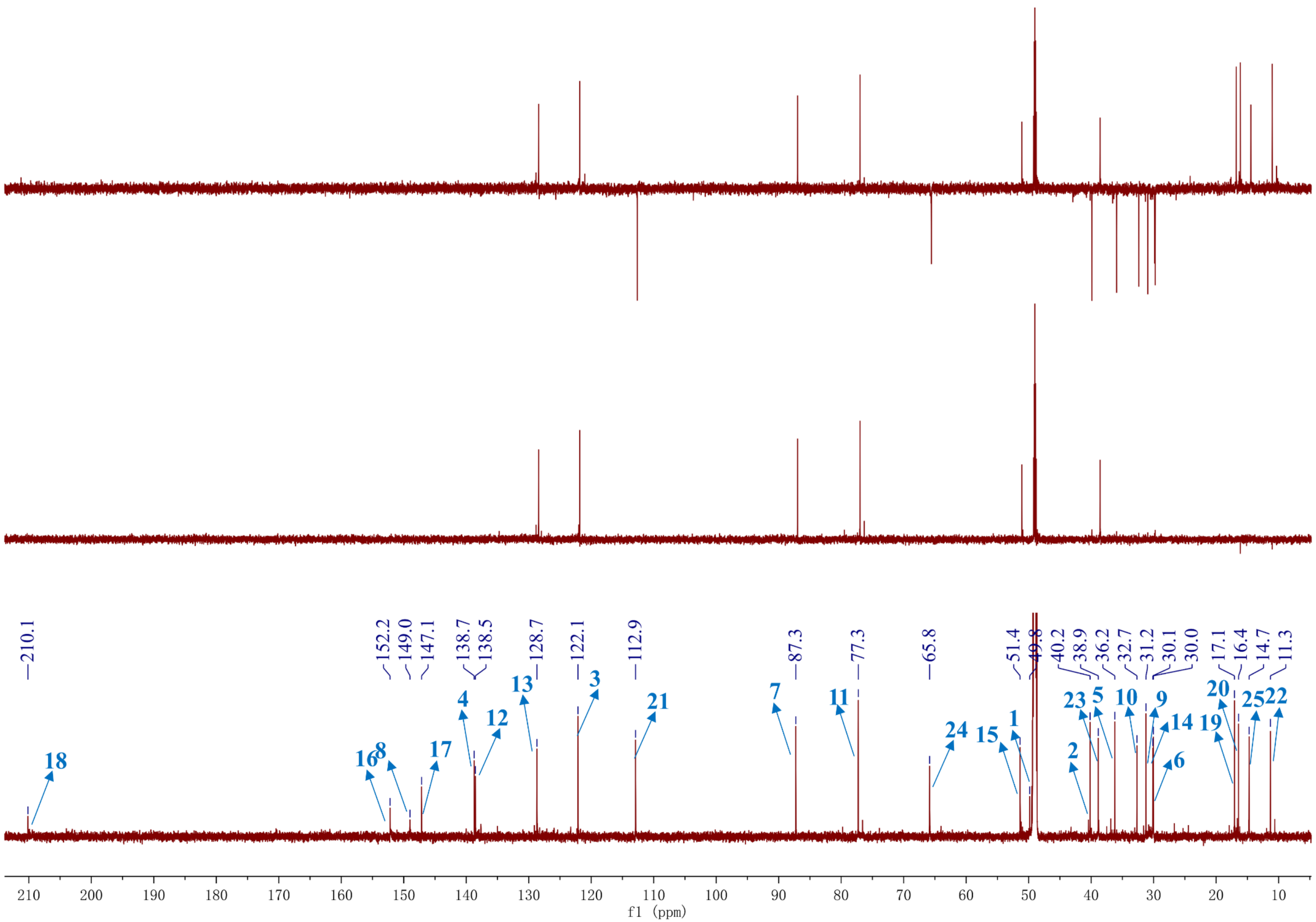

Figure S30. ^{13}C NMR and DEPT spectrum of arthritacin B (**2**) (Methanol-*d*$_4$).

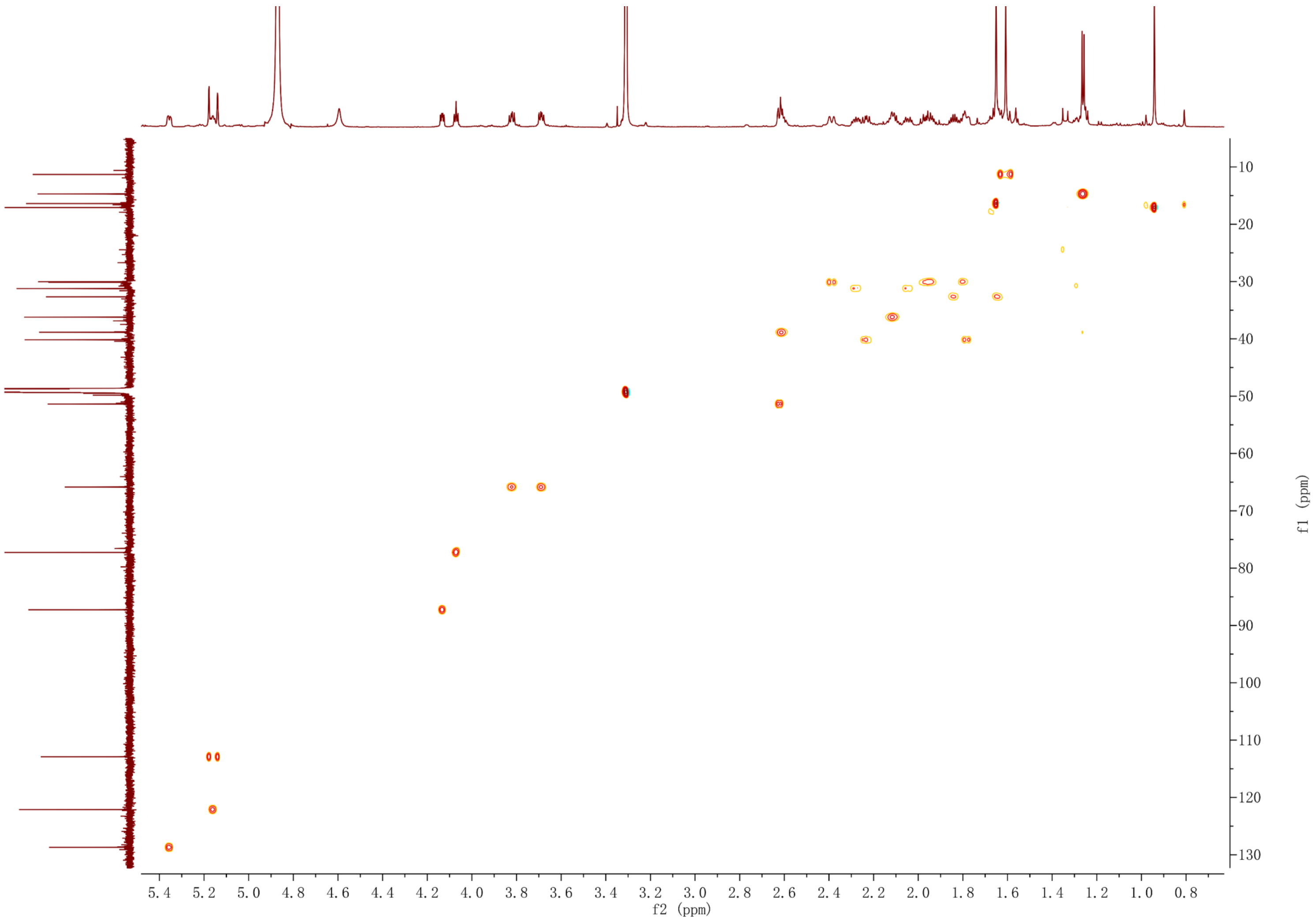

Figure S31. HSQC spectrum of arthritacin B (**2**) (Methanol-d_4).

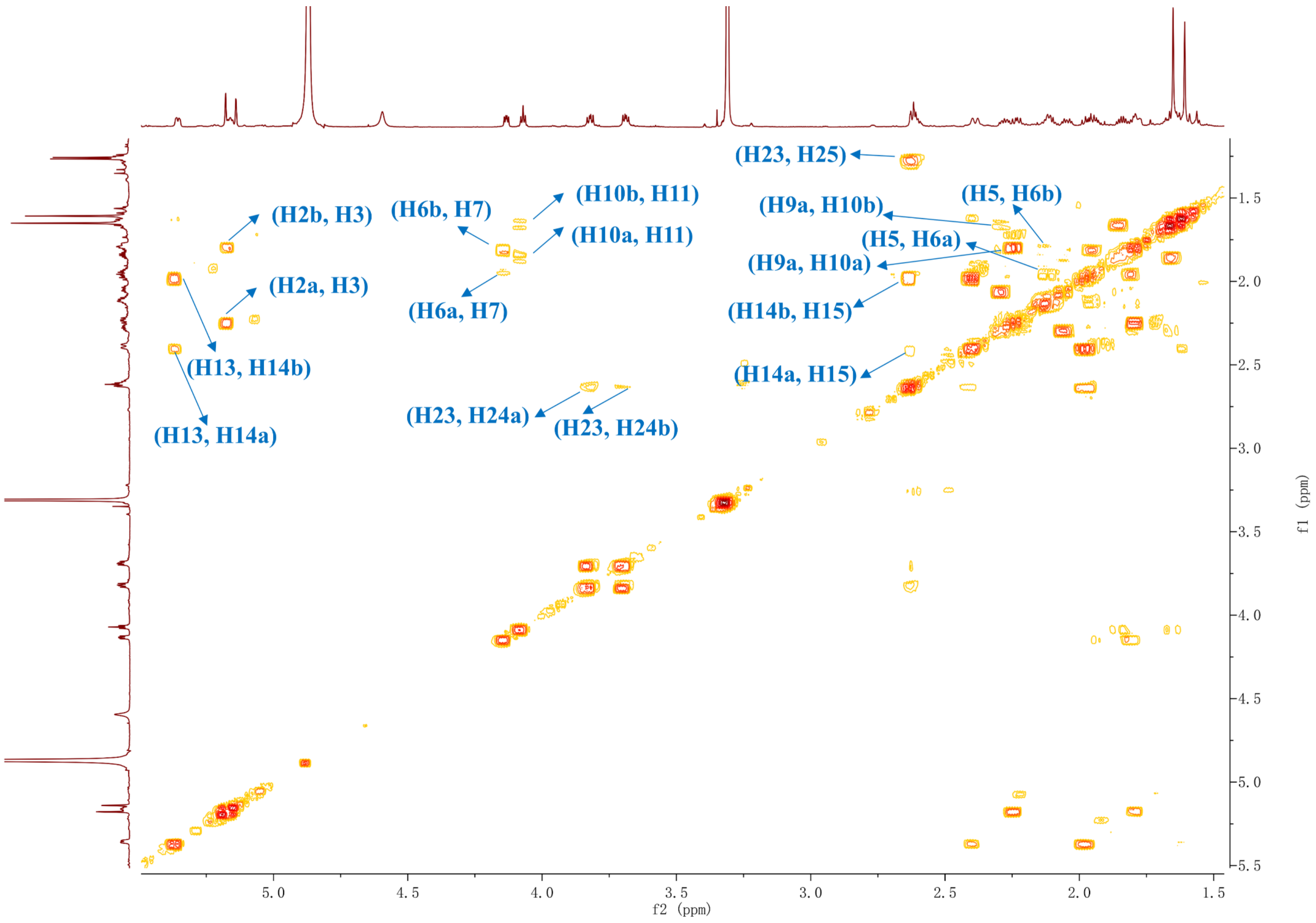

Figure S32. ^{1}H-^{1}H COSY spectrum of arthritacin B (**2**) (Methanol-d_4) and the selected cross-peaks (blue arrows and texts).

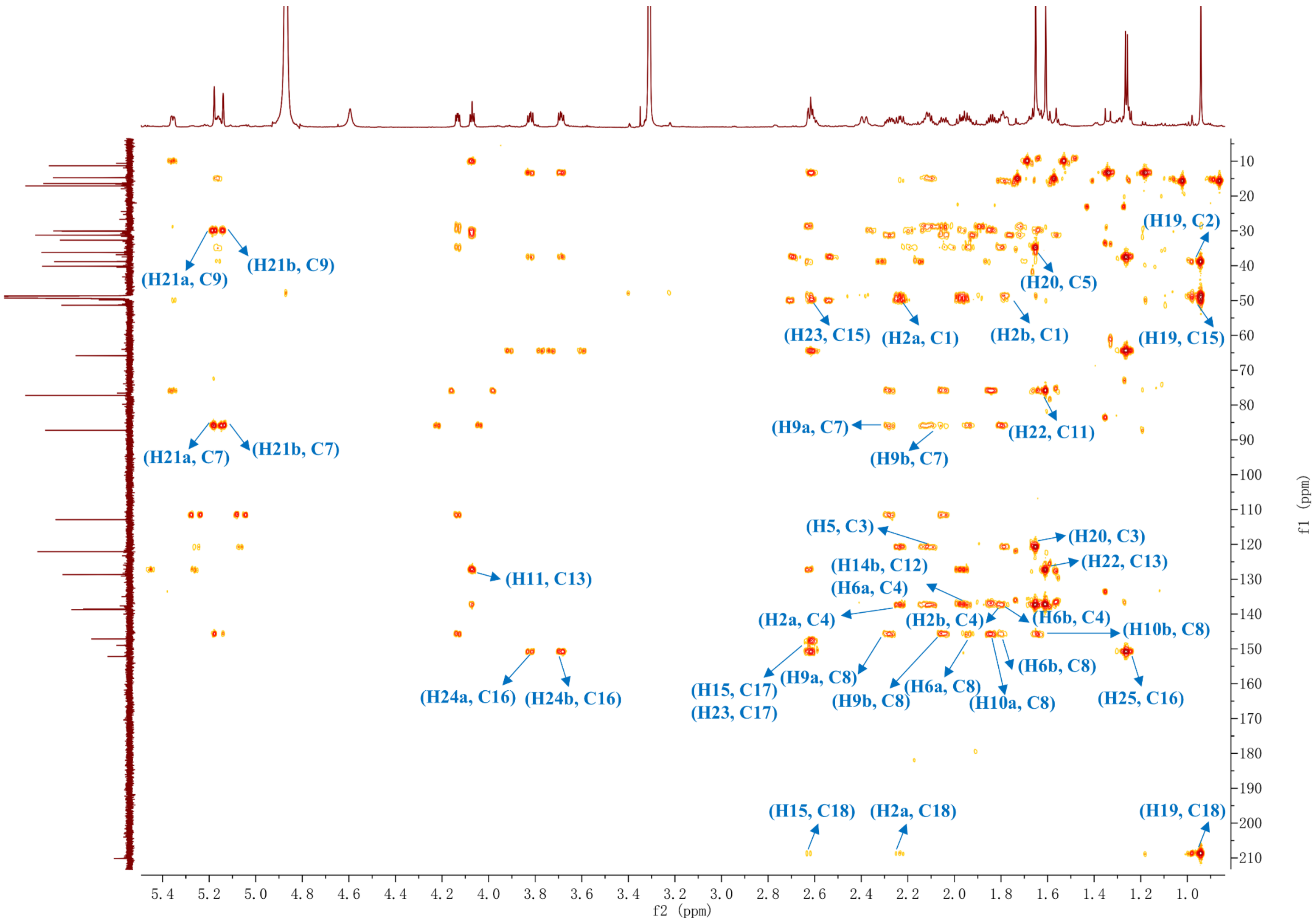

Figure S33. HMBC spectrum of arthritacin B (**2**) (Methanol-d_4) and the selected correlations (blue arrows and texts).

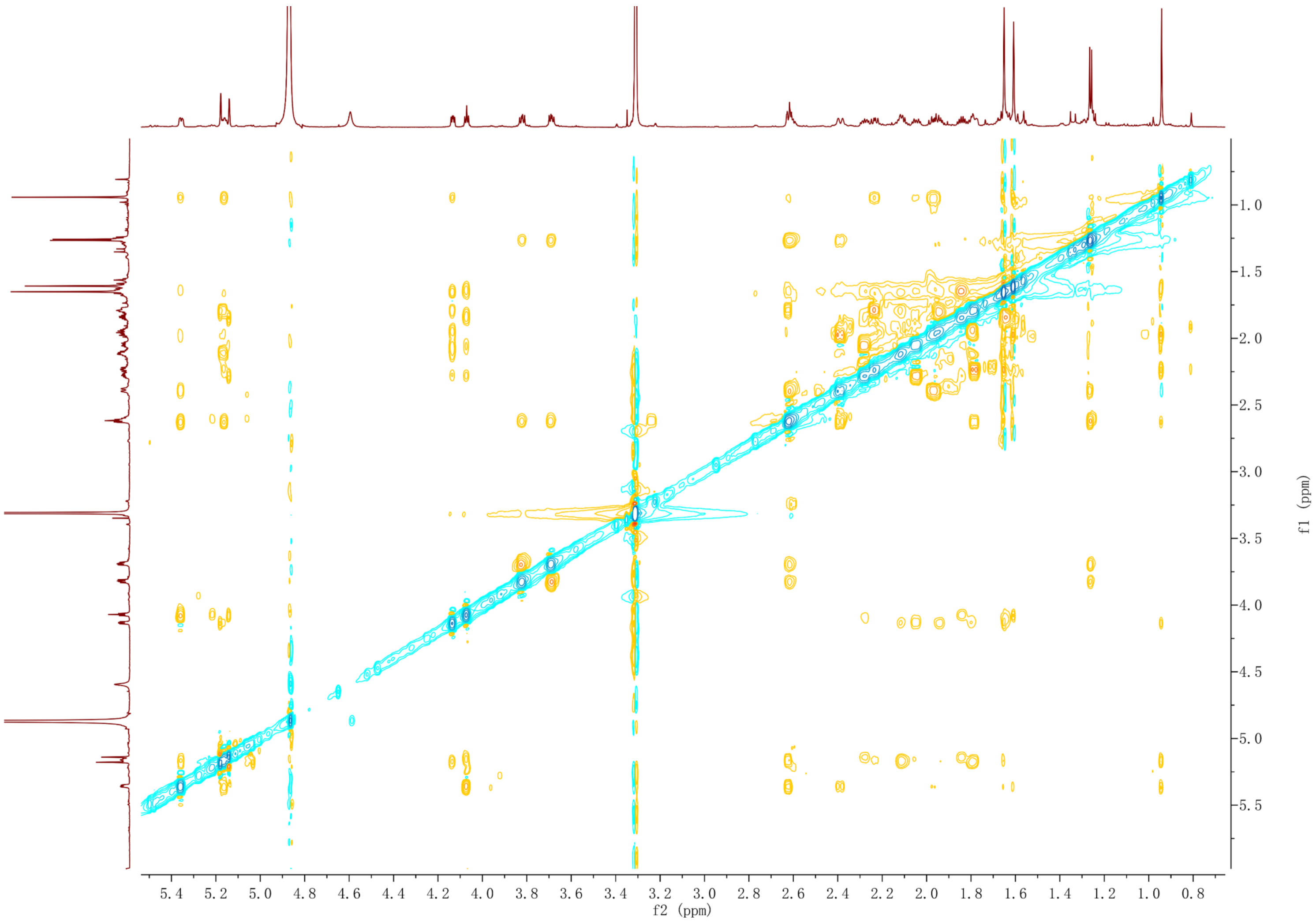

Figure S34. ROESY spectrum of arthritacin B (**2**) (Methanol-d_4) and the selected correlations (blue arrows and texts).

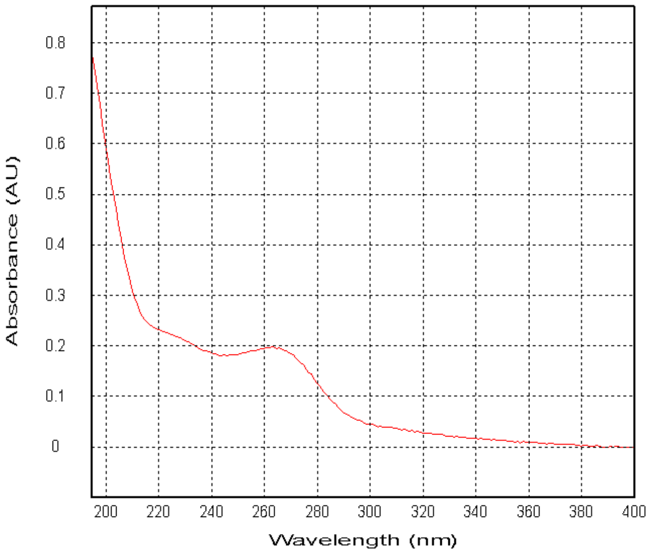

Figure S35. UV spectrum of arthritacin B (**2**).

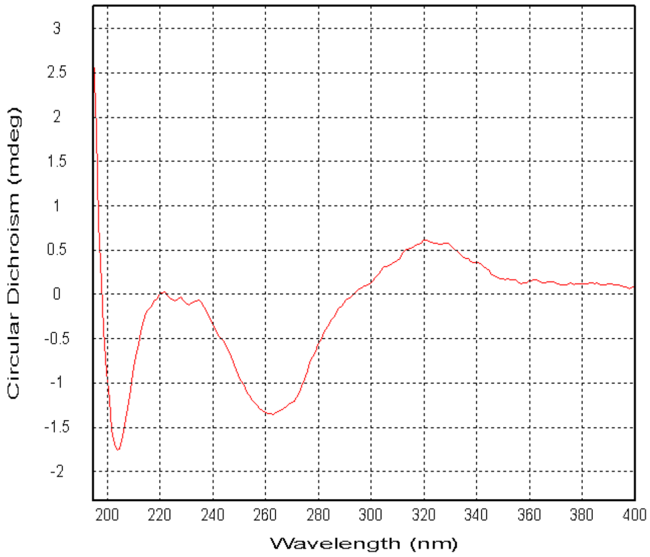

Figure S36. ECD spectrum of arthritacin B (**2**).

Rudolph Research Analytical

This sample was measured on an Autopol VI, Serial #91058
Manufactured by Rudolph Research Analytical, Hackettstown, NJ, USA.

Measurement Date : Friday, 28-MAY-2021

Set Temperature : OFF

Time Delay : Disabled

Delay between Measurement : Disabled

n	Average	Std.Dev.	% RSD	Maximum	Minimum
5	12.63	0.22	1.74	12.75	12.25

S.No	Sample ID	Time	Result	Scale	OR °Arc	WLG.nm	Lg.mm	Conc.g/100ml	Temp.
1	SHSJ26	10:56:19 AM	12.63	SR	0.0101	589	100.00	0.080	25.5
2	SHSJ26	10:56:27 AM	12.75	SR	0.0102	589	100.00	0.080	25.5
3	SHSJ26	10:56:35 AM	12.75	SR	0.0102	589	100.00	0.080	25.5
4	SHSJ26	10:56:44 AM	12.25	SR	0.0098	589	100.00	0.080	25.5
5	SHSJ26	10:56:52 AM	12.75	SR	0.0102	589	100.00	0.080	25.5

Figure S37. ORD report of arthritacin B (**2**).

User Spectra

Fragmentor Voltage	Collision Energy	Ionization Mode
135	0	ESI

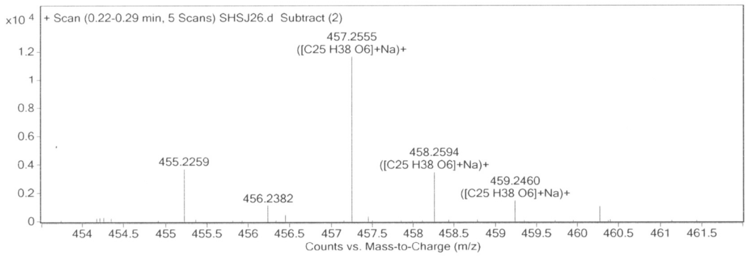

Peak List

m/z	z	Abund	Formula	Ion
274.2743	1	19733.72		
302.306	1	7544.31		
318.301	1	10747.7		
359.3175		6756.35		
381.298	1	7139.05		
399.2541	1	8276.84		
401.2686	1	9210.06		
417.2639	1	11656.33		
435.2749	1	12266.15		
457.2555	1	11678.55	C25 H38 O6	(M+Na)+

Formula Calculator Element Limits

Element	Min	Max
C	3	60
H	0	120
O	0	30

Formula Calculator Results

Formula	CalculatedMass	CalculatedMz	Mz	Diff. (mDa)	Diff. (ppm)	DBE
C25 H38 O6	434.2668	457.2561	457.2555	0.60	1.31	7.0000

--- End Of Report ---

Figure S38. HRESIMS spectrum of arthritacin B (**2**).

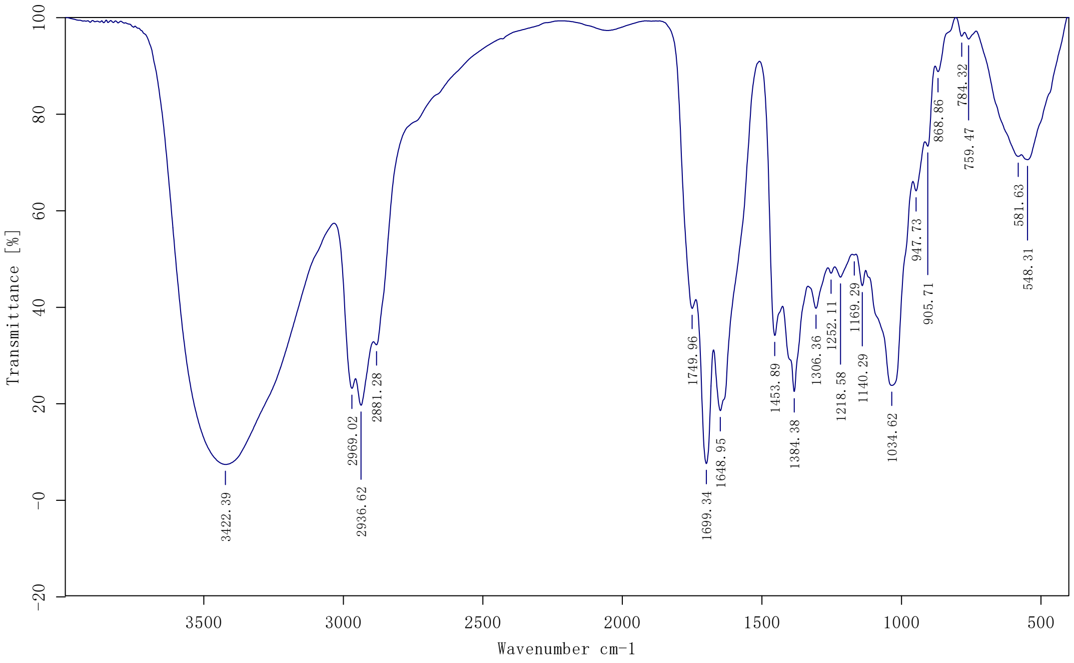

Sample Name: SHSJ26
Sample Form: KBr
Path of File: E:\data
Date of Measurement: 2025/7/3

Resolution: 4
Aperture Setting: 6 mm
Number of Background Scans: 16
Number of Sample Scans: 16

Beamsplitter Setting: KBr
Source Setting: MIR
Instrument Type: BRUKER VERTEX 70
Soft Version: OPUS8.1

Figure S39. IR spectrum of arthritacin B (**2**).

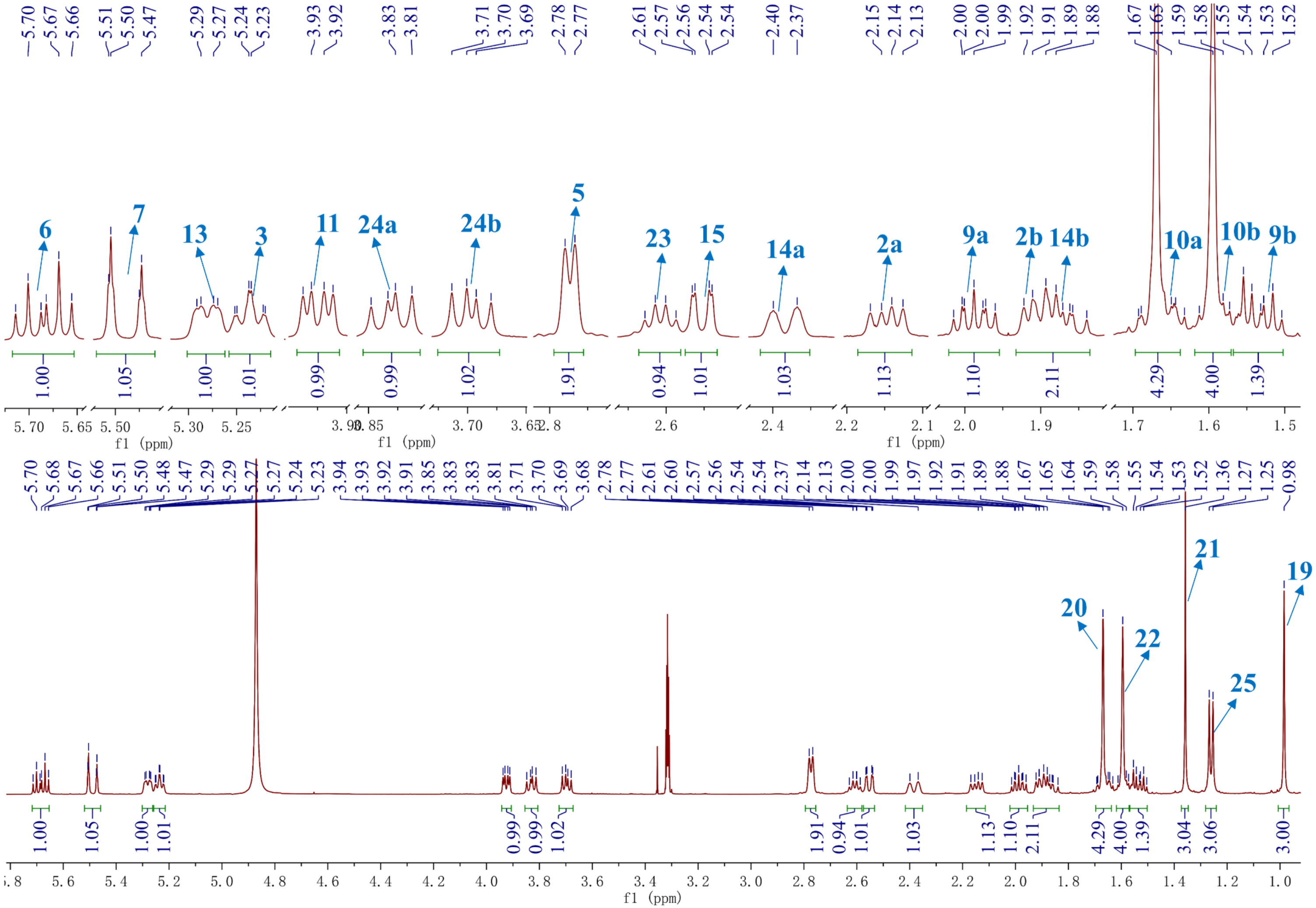

Figure S40. ^{1}H NMR spectrum of arthritacin C (**3**) (Methanol-d_4).

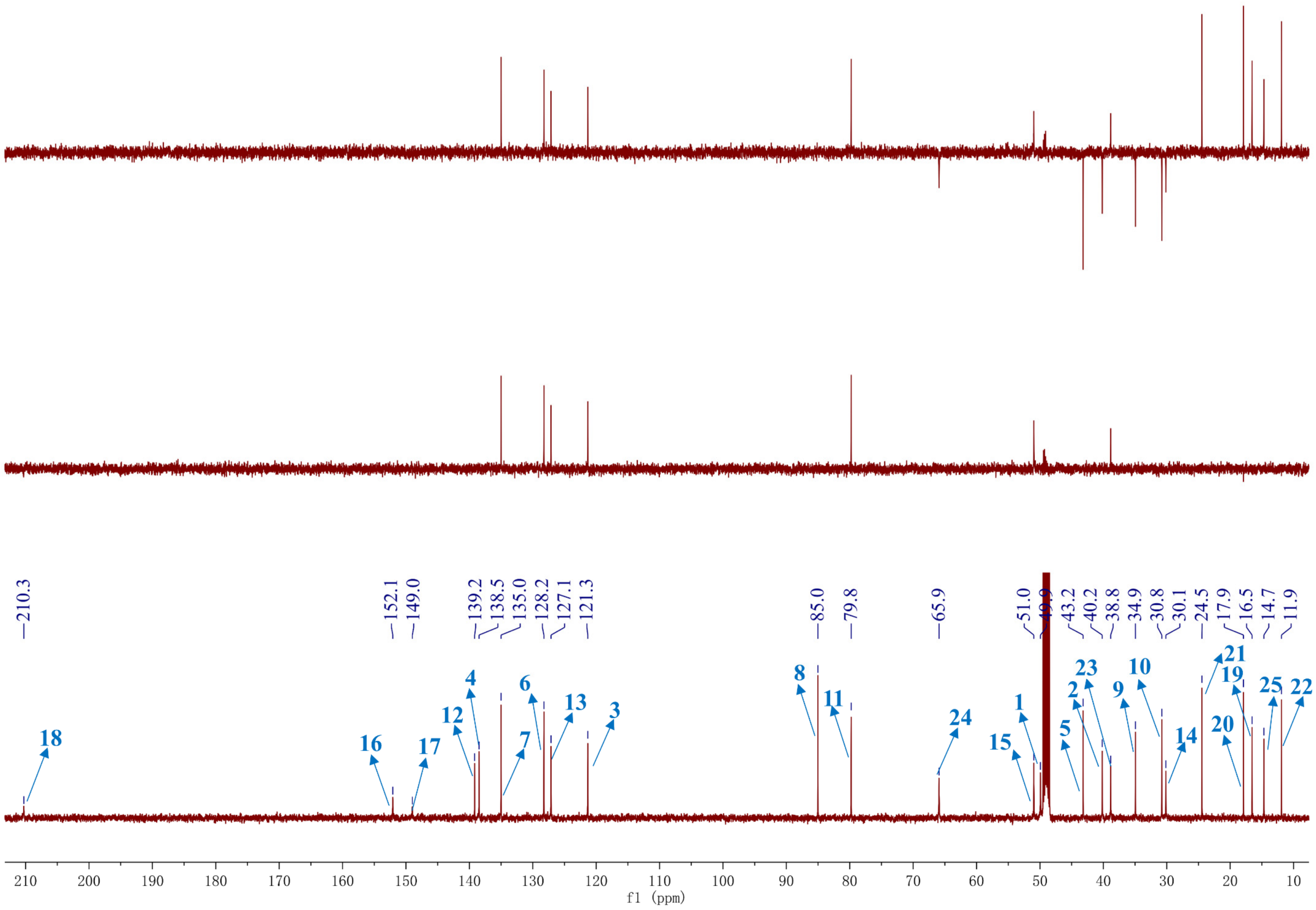

Figure S41. ^{13}C NMR and DEPT spectrum of arthritacin C (**3**) (Methanol-d_4).

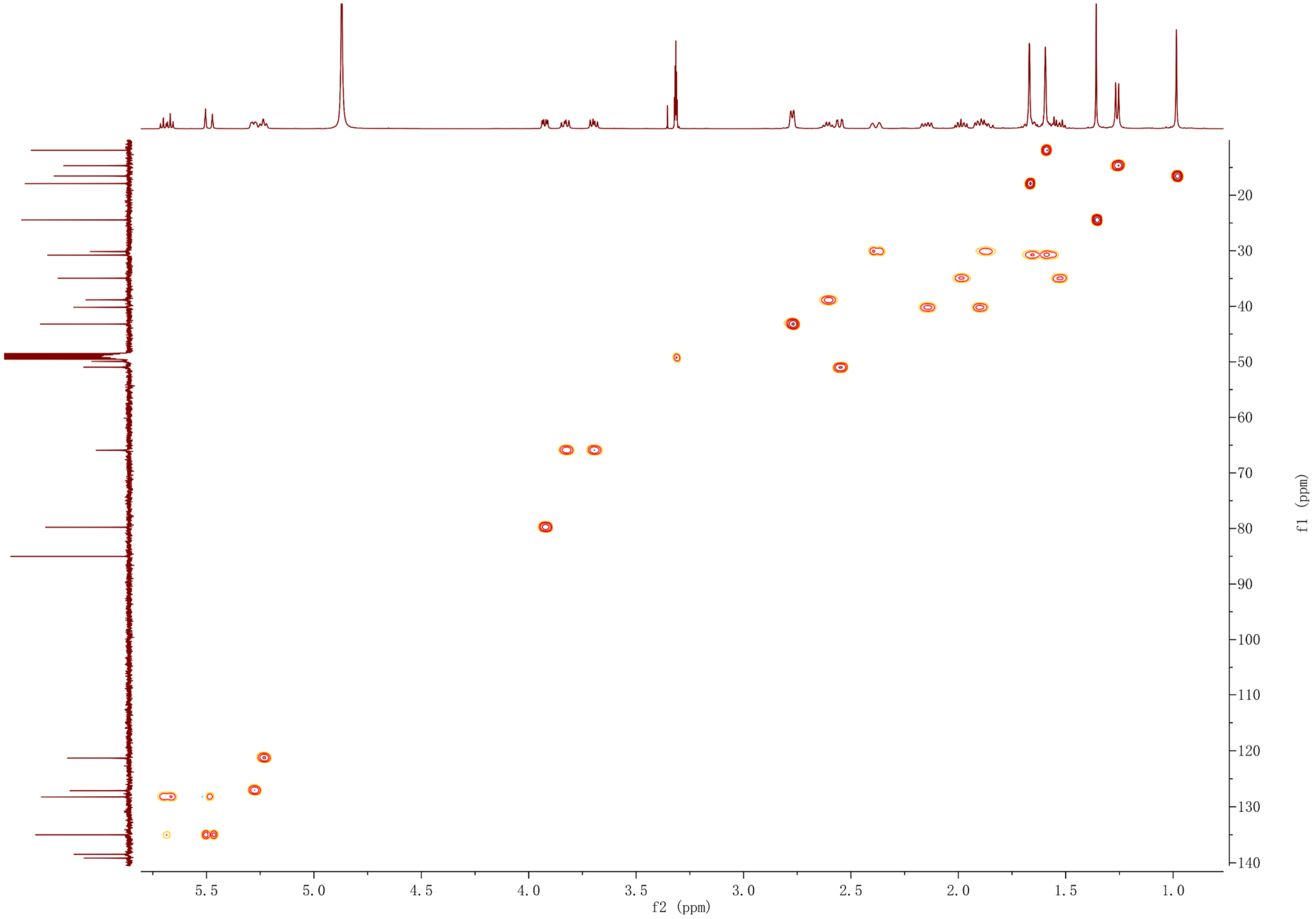

Figure S42. HSQC spectrum of arthritacin C (**3**) (Methanol-d_4).

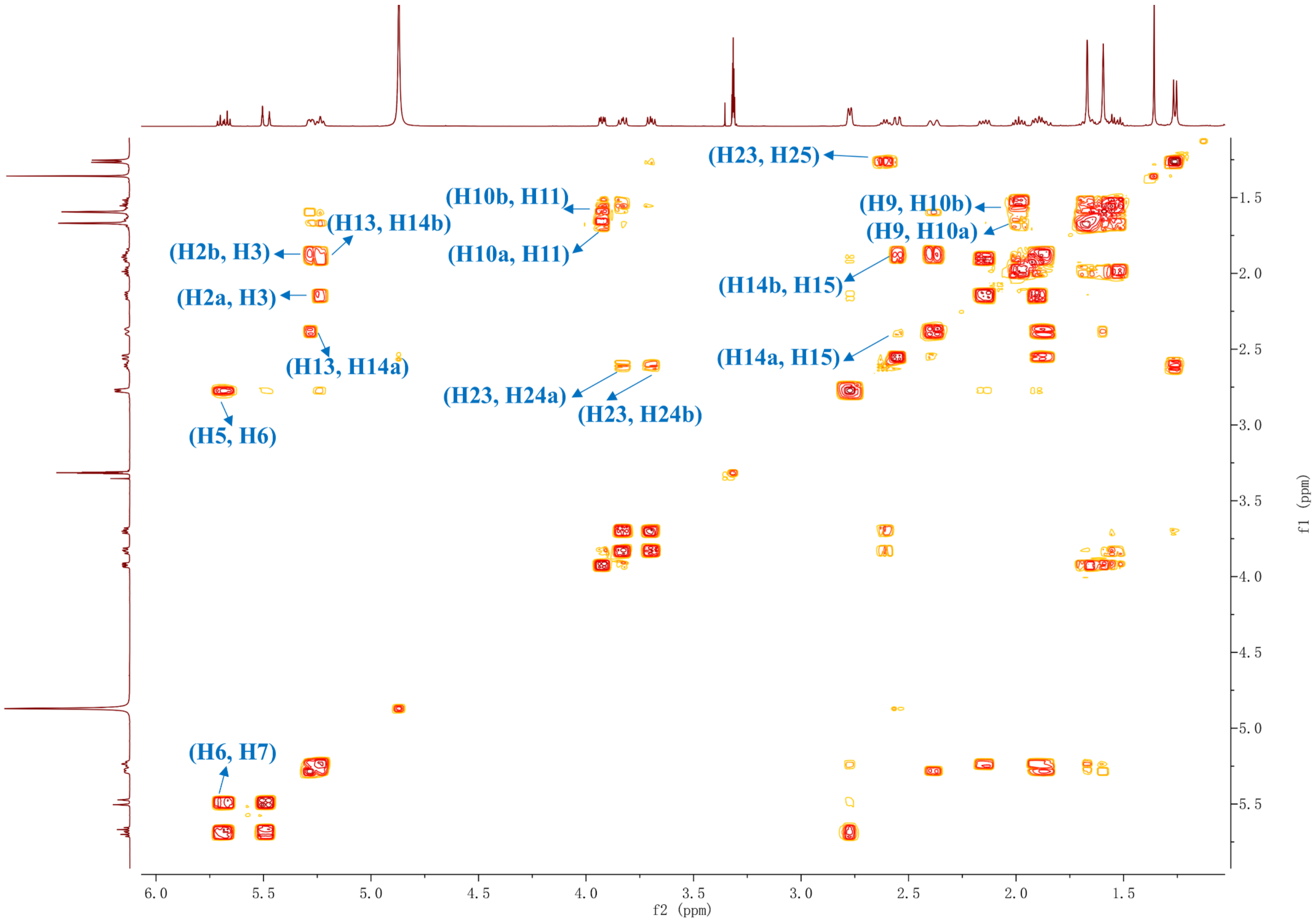

Figure S43. ^{1}H-^{1}H COSY spectrum of arthritacin C (**3**) (Methanol-d_4) and the selected cross-peaks (blue arrows and texts).

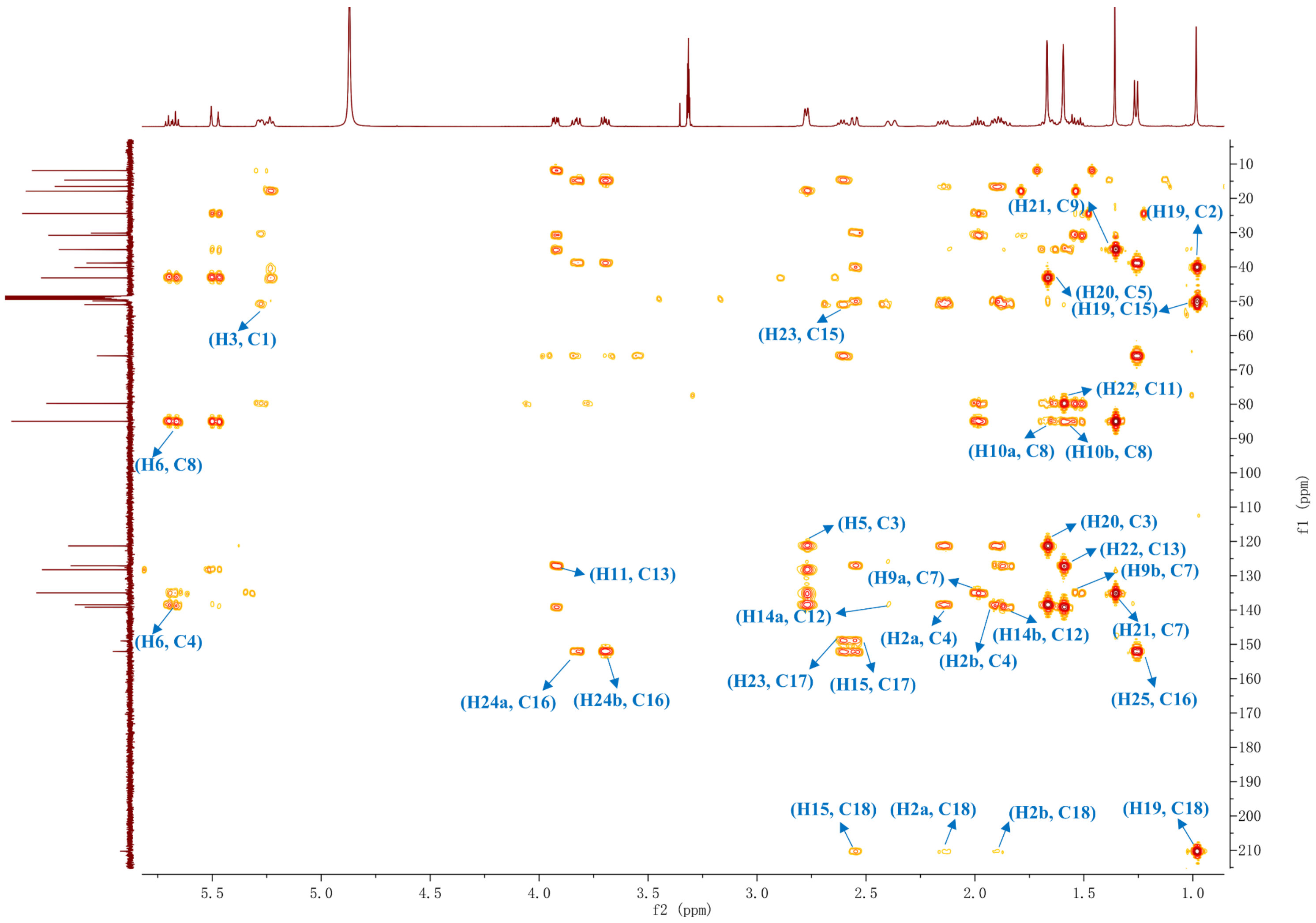

Figure S44. HMBC spectrum of arthritacin C (**3**) (Methanol-d_4) and the selected correlations (blue arrows and texts).

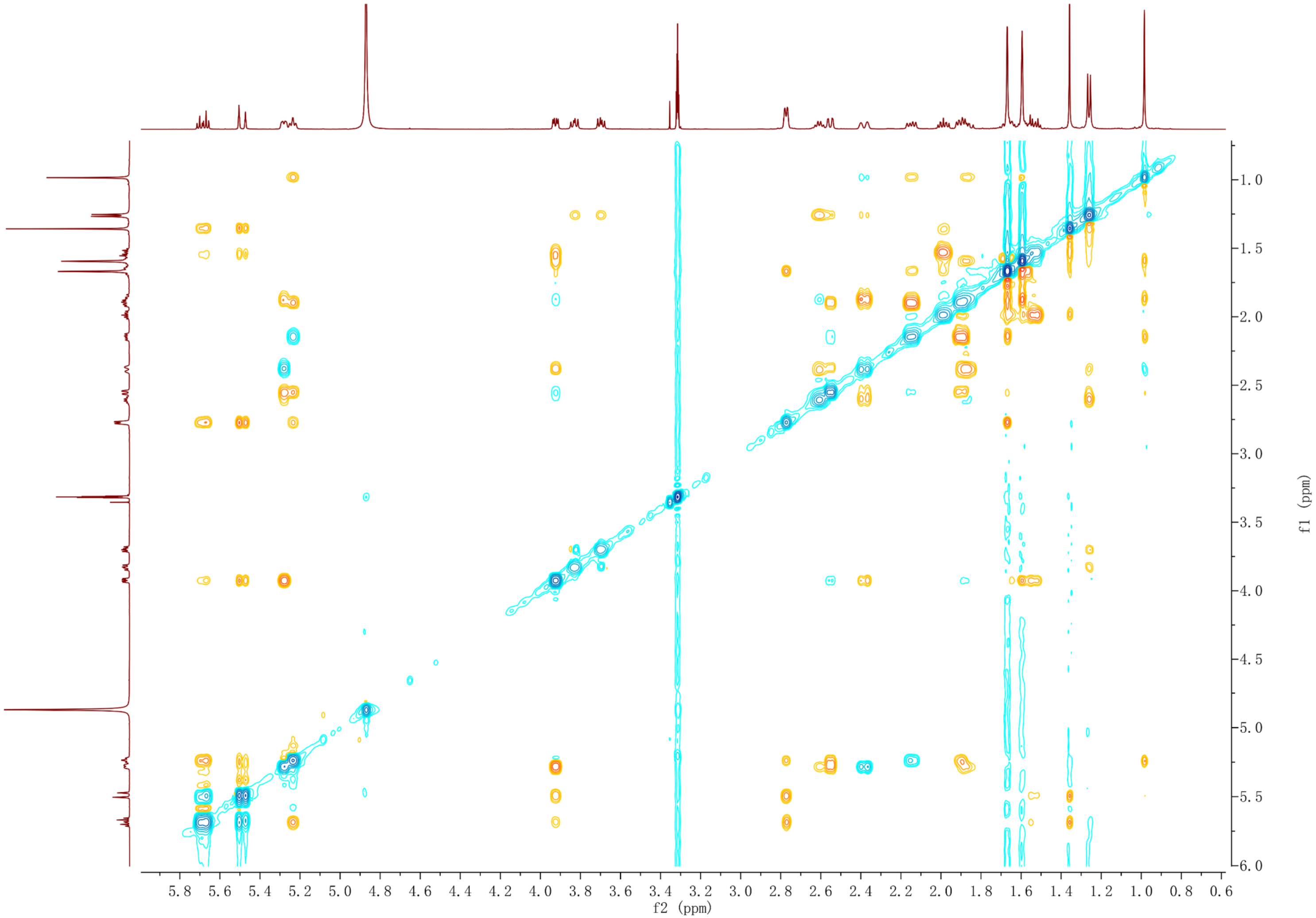

Figure S45. ROESY spectrum of arthritacin C (**3**) (Methanol-d_4) and the selected correlations (blue arrows and texts).

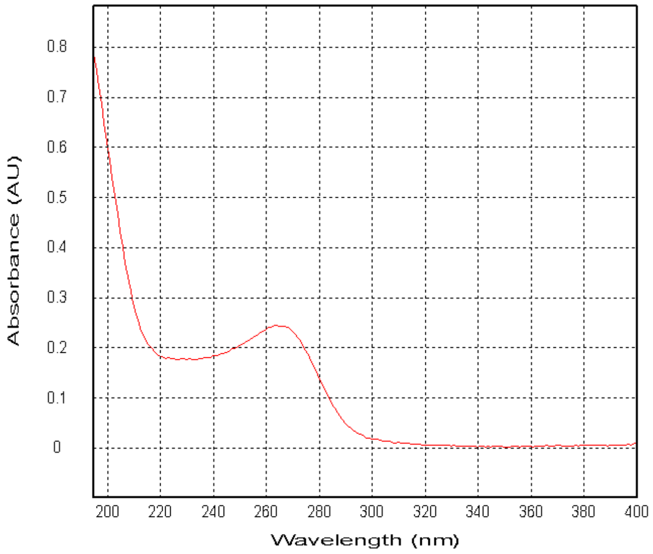

Figure S46. UV spectrum of arthritacin C (**3**).

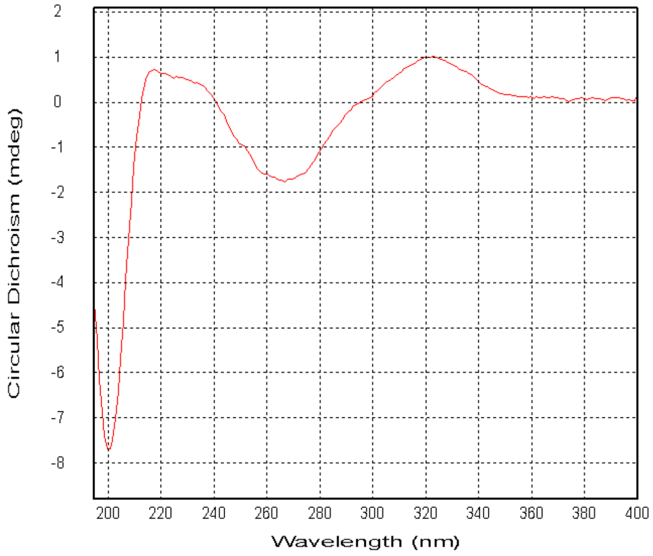

Figure S47. ECD spectrum of arthritacin C (**3**).

Rudolph Research Analytical

This sample was measured on an Autopol VI, Serial #91058
Manufactured by Rudolph Research Analytical, Hackettstown, NJ, USA.

Measurement Date : Tuesday, 02-JAN-2024

Set Temperature : 20.0

Time Delay : Disabled

Delay between Measurement : Disabled

n	Average	Std.Dev.	% RSD	Maximum	Minimum
5	2.25	0.84	37.33	3.75	1.88

S.No	Sample ID	Time	Result	Scale	OR °Arc	WLG.nm	Lg.mm	Conc.g/100ml	Temp.
1	SHSJR20	09:39:27 AM	3.75	SR	0.006	589	100.00	0.160	20.7
2	SHSJR20	09:39:33 AM	1.88	SR	0.003	589	100.00	0.160	20.7
3	SHSJR20	09:39:40 AM	1.88	SR	0.003	589	100.00	0.160	20.6
4	SHSJR20	09:39:46 AM	1.88	SR	0.003	589	100.00	0.160	20.5
5	SHSJR20	09:39:52 AM	1.88	SR	0.003	589	100.00	0.160	20.4

Figure S48. ORD report of arthritacin C (**3**).

User Spectra

Fragmentor Voltage	Collision Energy	Ionization Mode
135	0	ESI

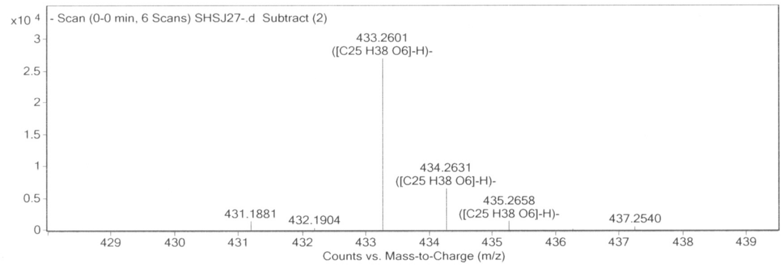

Peak List

m/z	z	Abund	Formula	Ion
89.0244	1	7591.31		
255.2326	1	3640.46		
283.2644	1	4161.78		
433.2601	1	26927.04	C25 H38 O6	(M-H)-
434.2631	1	6487.27	C25 H38 O6	(M-H)-
469.237	1	2407.77		
499.1738	1	1806.8		
501.2452	1	1461.75		
523.2908	1	2321.51		
547.2511		1343.94		

Formula Calculator Element Limits

Element	Min	Max
C	3	60
H	0	120
O	0	30
N	0	5

Formula Calculator Results

Formula	CalculatedMass	CalculatedMz	Mz	Diff. (mDa)	Diff. (ppm)	DBE
C25 H38 O6	434.2668	433.2596	433.2601	-0.50	-1.15	7.0000

--- End Of Report ---

Figure S49. HRESIMS spectrum of arthritacin C (**3**).

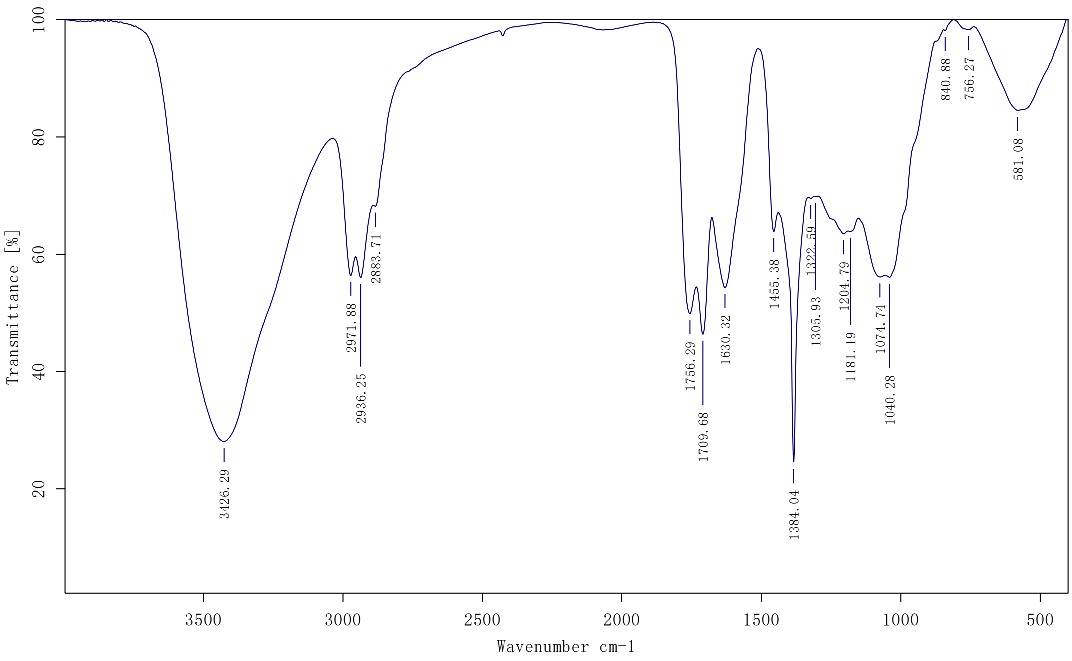

Sample Name: SHSJ27
Sample Form: KBr
Path of File: E:\data
Date of Measurement: 2025/7/3

Resolution: 4
Aperture Setting: 6 mm
Number of Background Scans: 16
Number of Sample Scans: 16

Beamsplitter Setting: KBr
Source Setting: MIR
Instrument Type: BRUKER VERTEX 70
Soft Version: OPUS8.1

Figure S50. IR spectrum of arthritacin C (**3**).

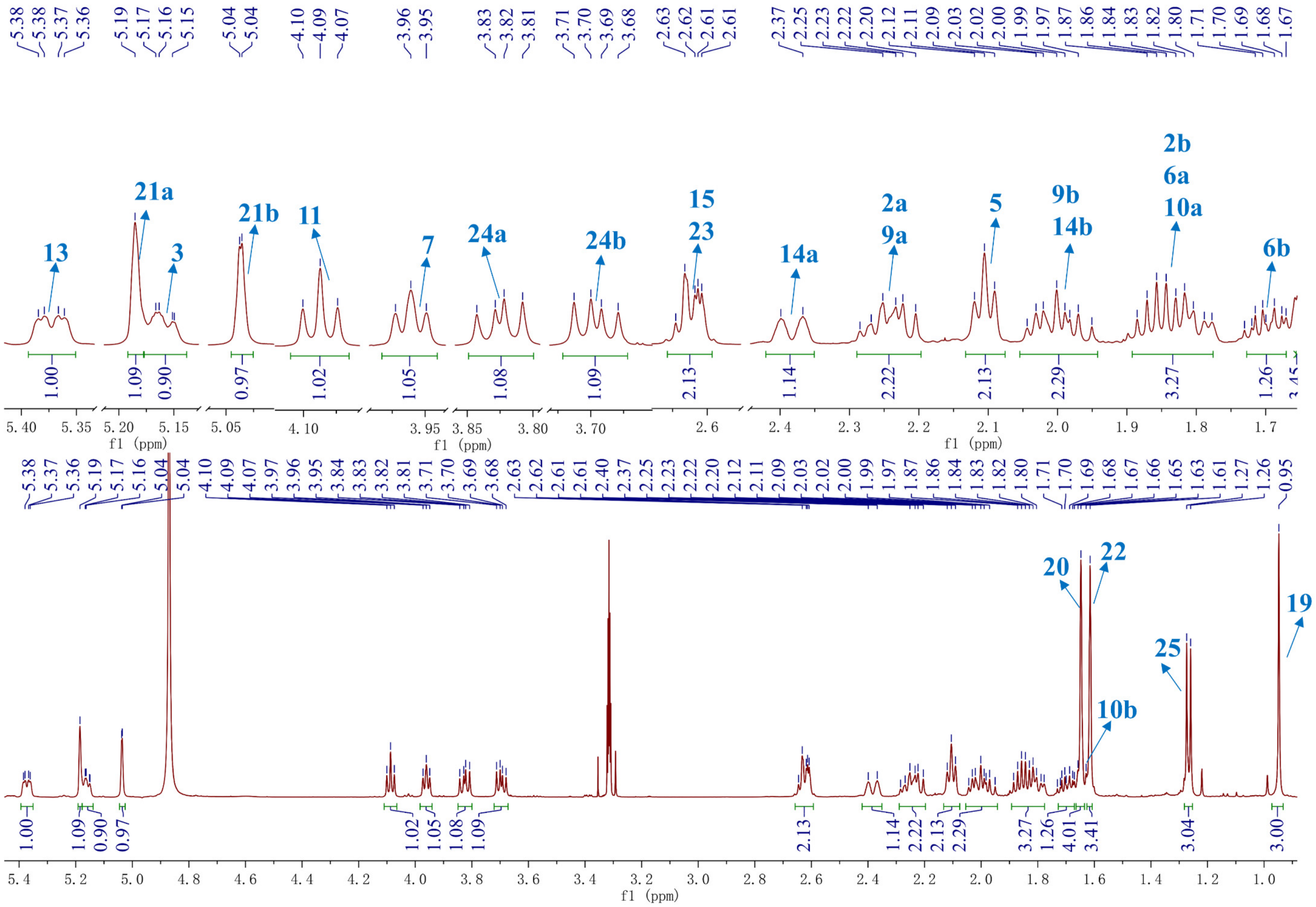

Figure S51. ^{1}H NMR spectrum of arthritacin D (**4**) (Methanol-d_4).

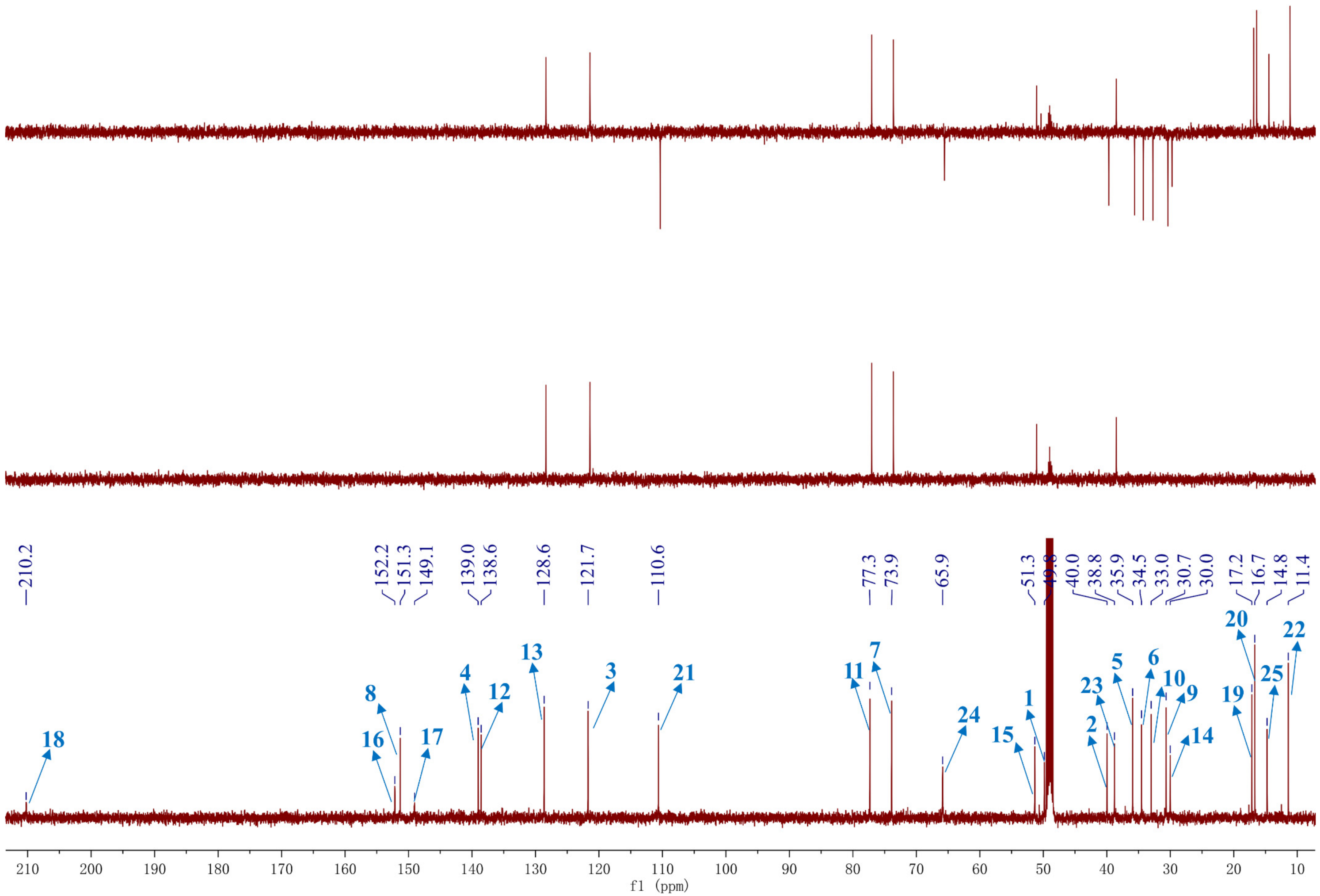

Figure S52. ^{13}C NMR and DEPT spectrum of arthritacin D (**4**) (Methanol-d_4).

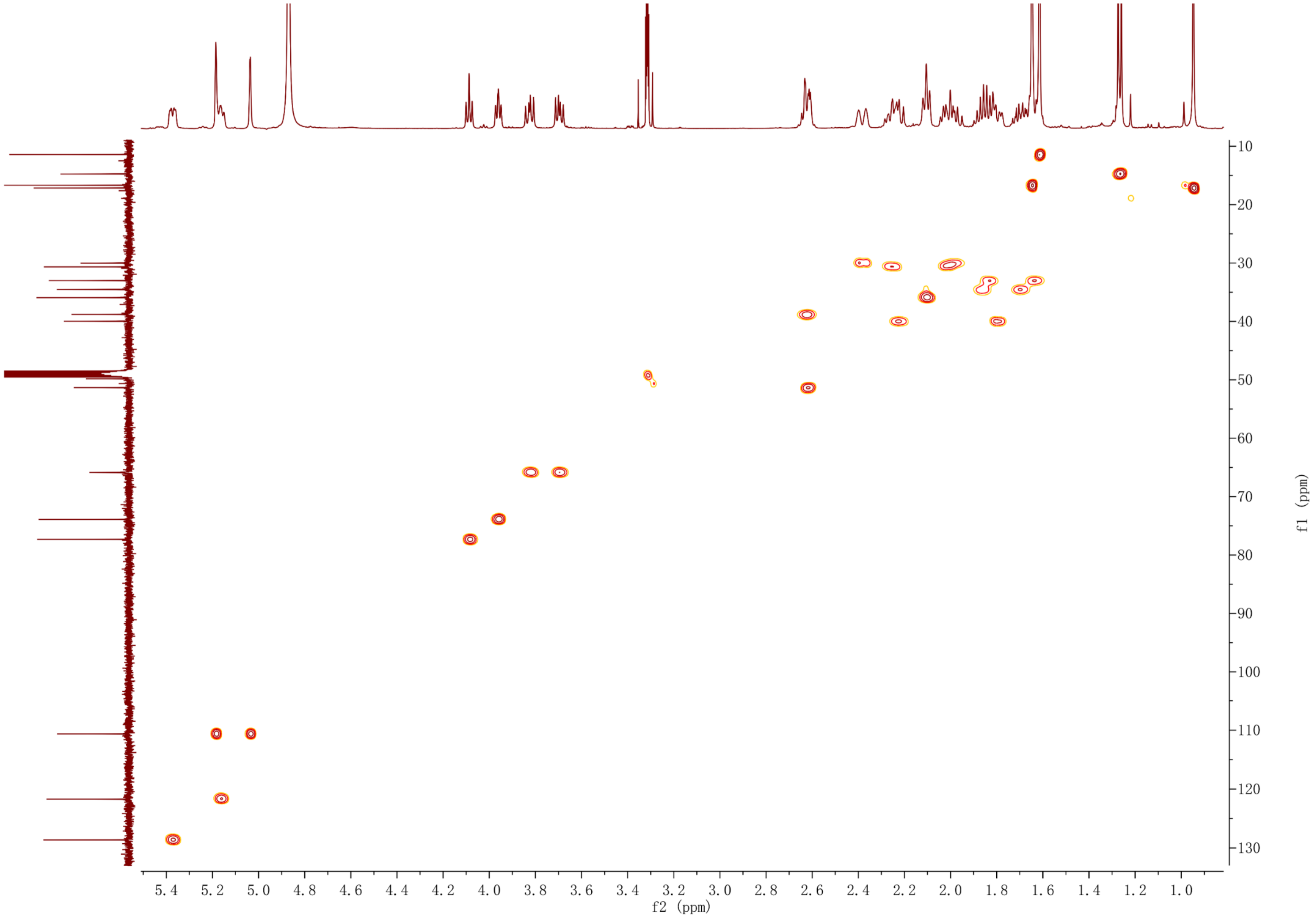

Figure S53. HSQC spectrum of arthritacin D (**4**) (Methanol-d_4).

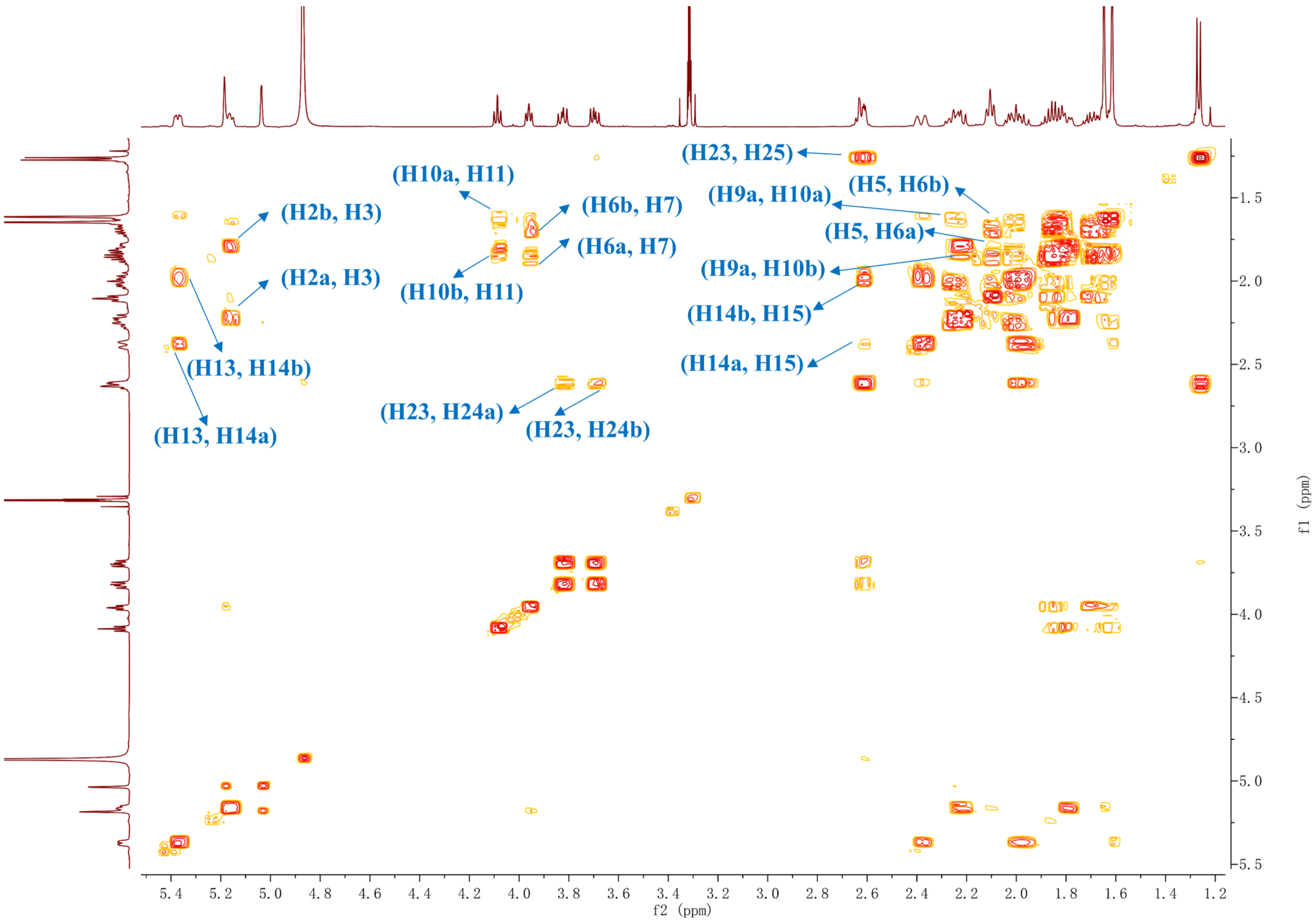

Figure S54. ^{1}H-^{1}H COSY spectrum of arthritacin D (**4**) (Methanol-d_4) and the selected cross-peaks (blue arrows and texts).

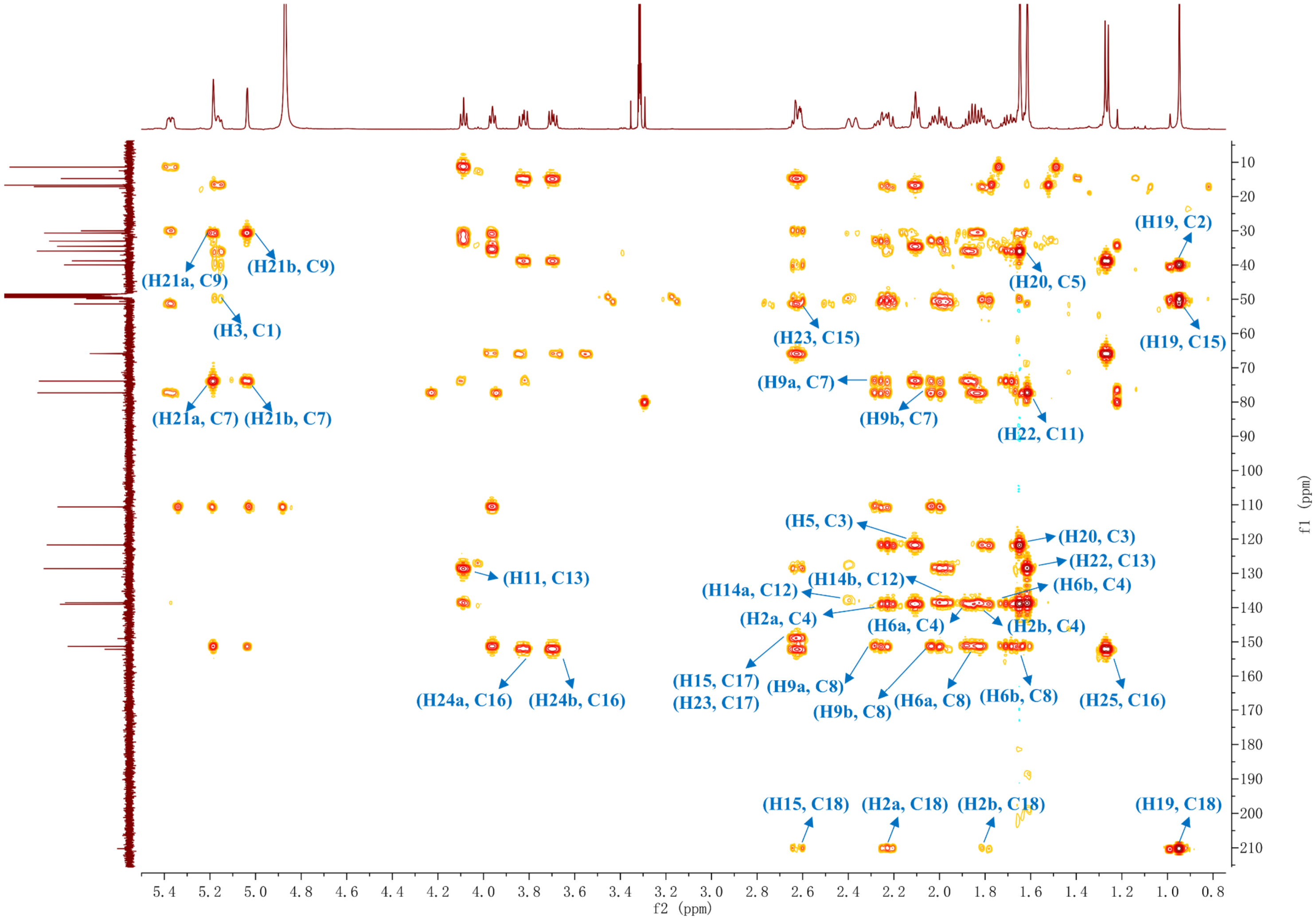

Figure S55. HMBC spectrum of arthritacin D (**4**) (Methanol-d_4) and the selected correlations (blue arrows and texts).

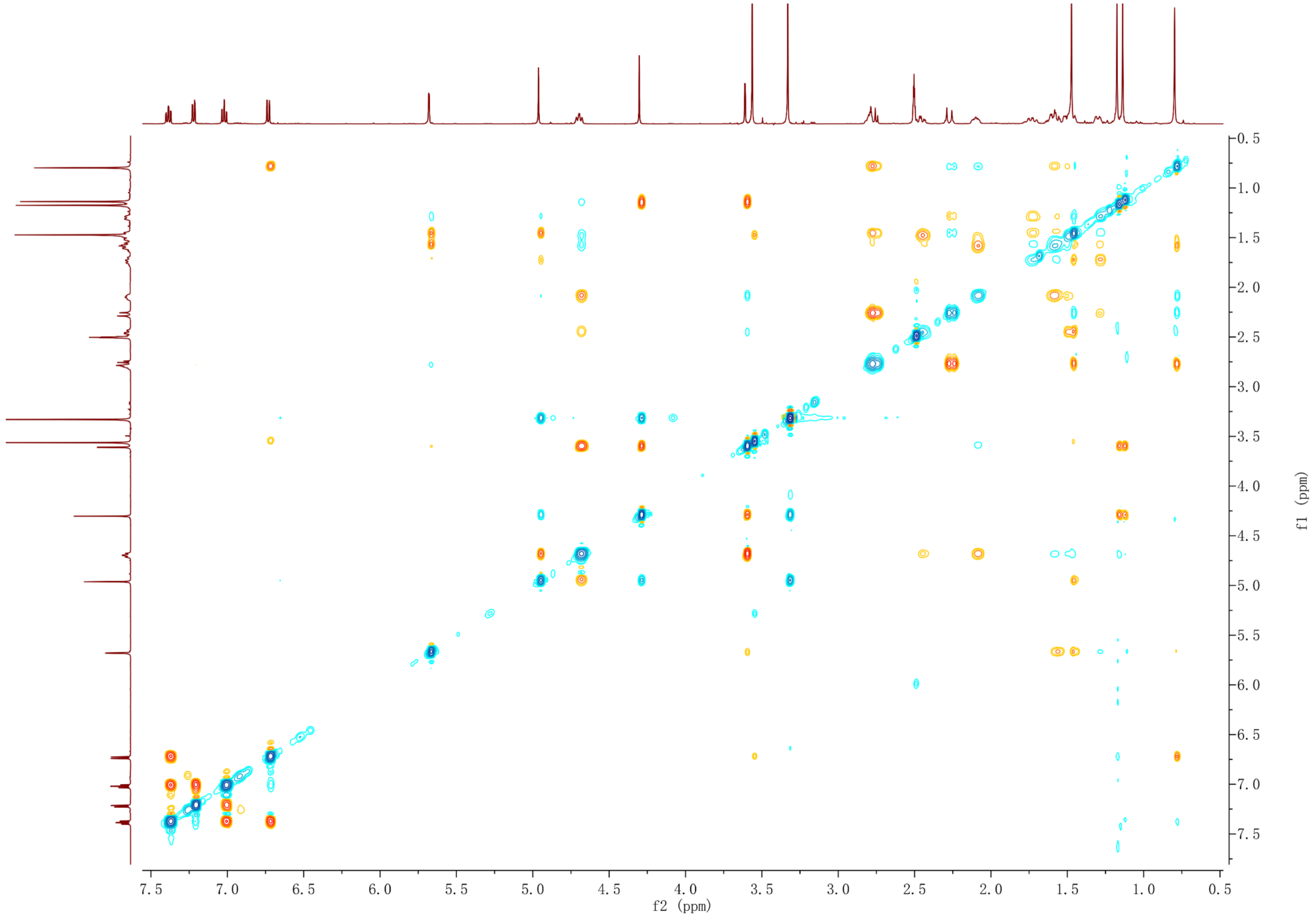

Figure S56. ROESY spectrum of arthritacin D (**4**) (Methanol-d_4) and the selected correlations (blue arrows and texts).

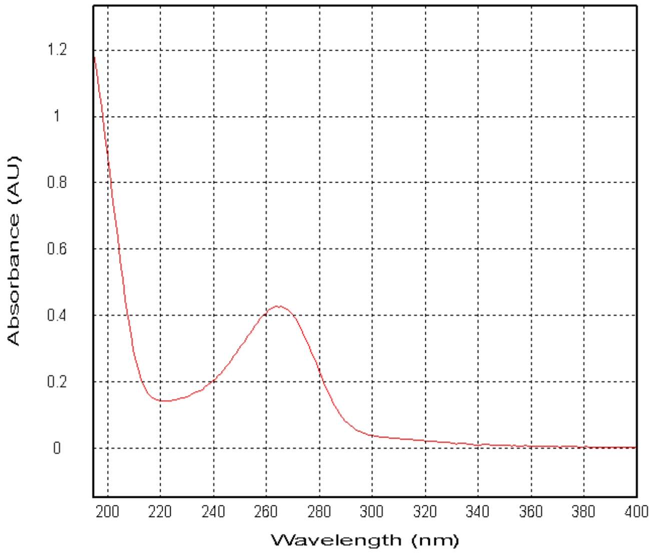

Figure S57. UV spectrum of arthritacin D (**4**).

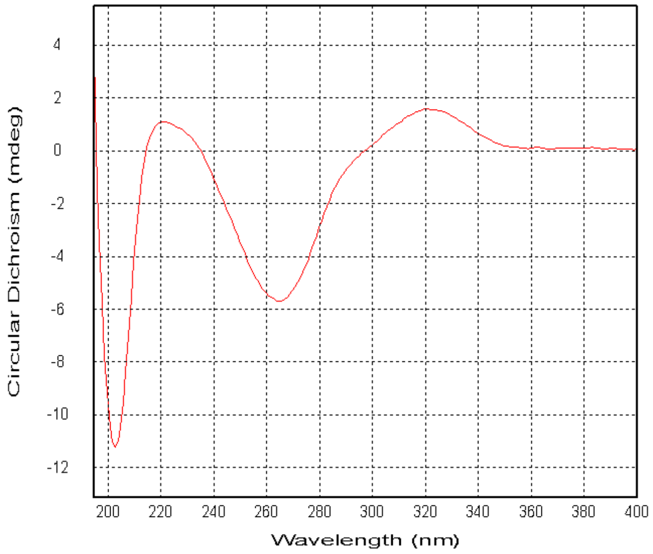

Figure S58. ECD spectrum of arthritacin D (**4**).

Rudolph Research Analytical

This sample was measured on an Autopol VI, Serial #91058
Manufactured by Rudolph Research Analytical, Hackettstown, NJ, USA.

Measurement Date : Friday, 28-MAY-2021

Set Temperature : OFF

Time Delay : Disabled

Delay between Measurement : Disabled

n	Average	Std.Dev.	% RSD	Maximum	Minimum
5	24.39	0.14	0.57	24.53	24.20

S.No	Sample ID	Time	Result	Scale	OR °Arc	WLG.nm	Lg.mm	Conc.g/100ml	Temp.
1	SHSJ44	10:01:08 AM	24.20	SR	0.0363	589	100.00	0.150	24.5
2	SHSJ44	10:01:16 AM	24.53	SR	0.0368	589	100.00	0.150	24.5
3	SHSJ44	10:01:24 AM	24.27	SR	0.0364	589	100.00	0.150	24.5
4	SHSJ44	10:01:33 AM	24.47	SR	0.0367	589	100.00	0.150	24.5
5	SHSJ44	10:01:41 AM	24.47	SR	0.0367	589	100.00	0.150	24.5

Figure S59. ORD report of arthritacin D (**4**).

User Spectra

Fragmentor Voltage	Collision Energy	Ionization Mode
135	0	ESI

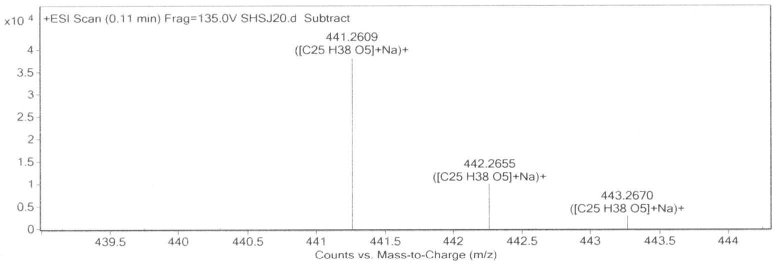

Peak List

m/z	z	Abund	Formula	Ion
102.1278	1	18761.85		
118.1229		4974.24		
251.1261	1	5738.05		
401.2684	1	21141.05		
402.2726	1	5871.94		
441.2609	1	37975.18	C25 H38 O5	(M+Na)+
442.2655	1	9870.73	C25 H38 O5	(M+Na)+
453.1681	1	5327.82		
654.3311	1	10755.1		
659.2876	1	7428.39		

Formula Calculator Element Limits

Element	Min	Max
C	3	60
H	0	120
O	0	30

Formula Calculator Results

Formula	CalculatedMass	CalculatedMz	Mz	Diff. (mDa)	Diff. (ppm)	DBE
C25 H38 O5	418.2719	441.2611	441.2609	0.20	0.45	7.0000

--- End Of Report ---

Figure S60. HRESIMS spectrum of arthritacin D (**4**).

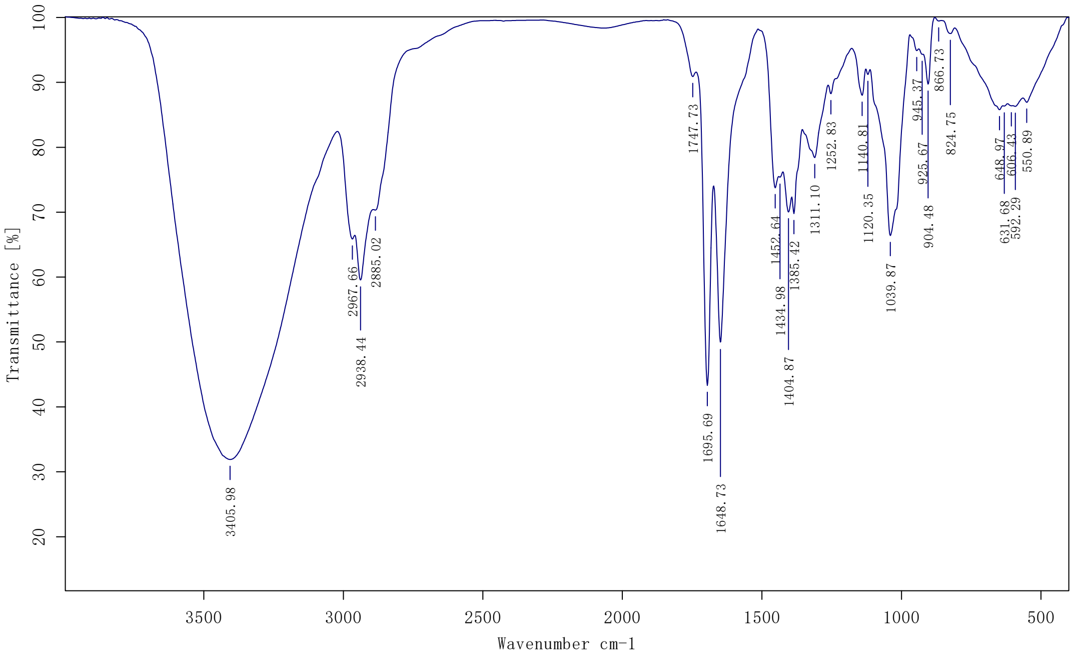

Sample Name: SHSJ20
Sample Form: KBr
Path of File: E:\data
Date of Measurement: 2025/7/3
Resolution: 4
Aperture Setting: 6 mm
Number of Background Scans: 16
Number of Sample Scans: 16
Beamsplitter Setting: KBr
Source Setting: MIR
Instrument Type: BRUKER VERTEX 70
Soft Version: OPUS8.1

Figure S61. IR spectrum of arthritacin D (**4**).

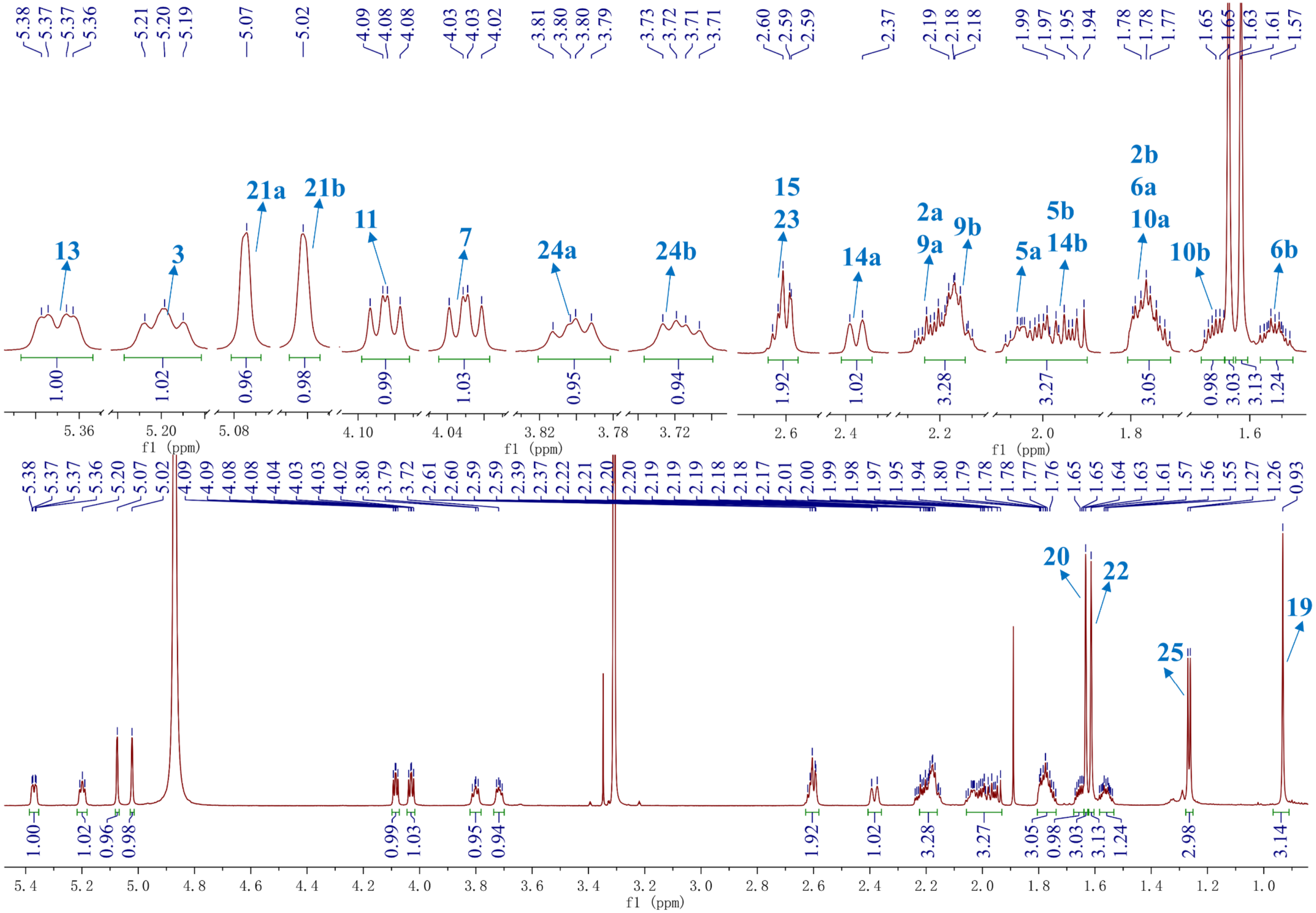

Figure S62. ^{1}H NMR spectrum of arthritacin E (**5**) (Methanol-d_4).

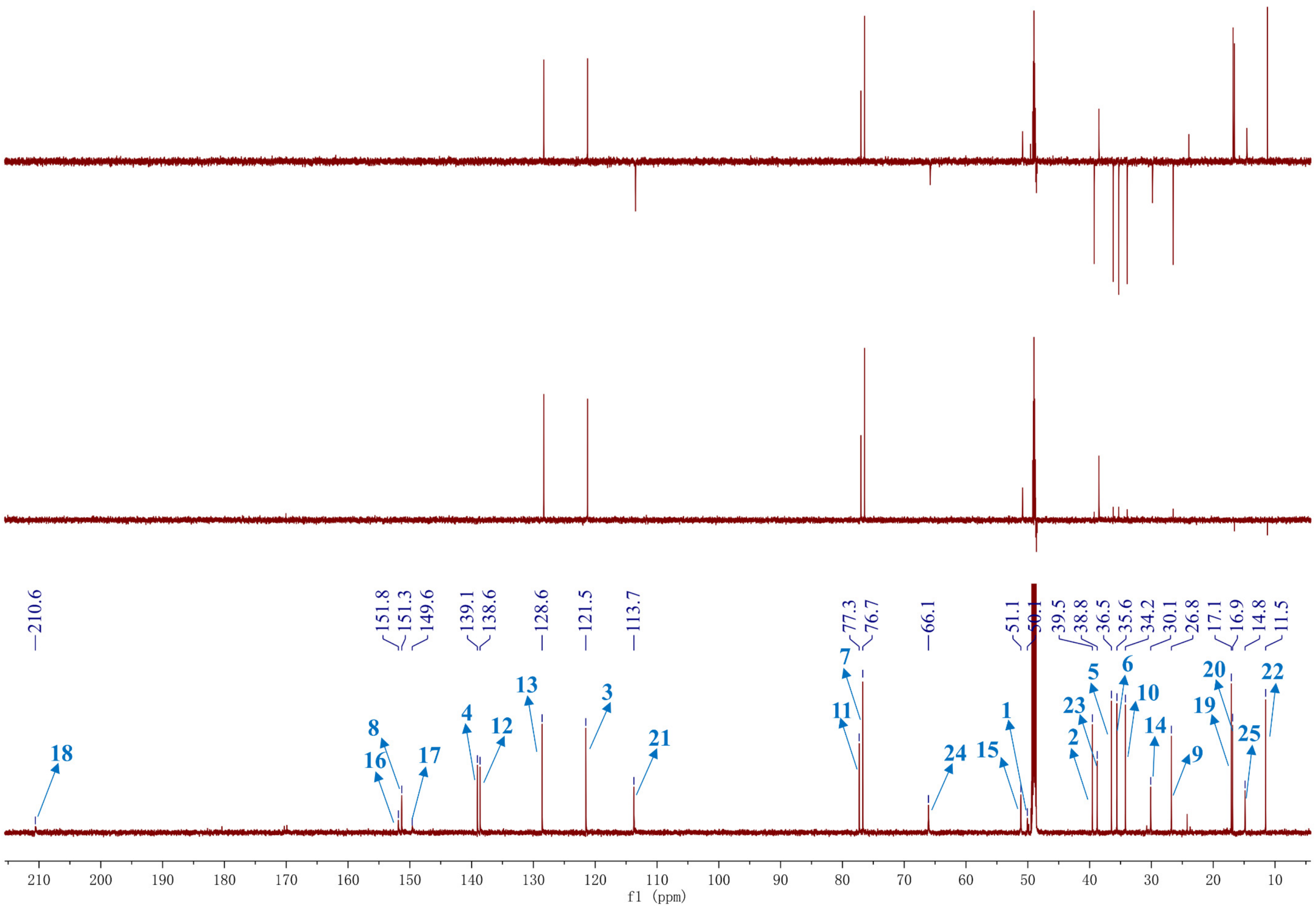

Figure S63. ^{13}C NMR and DEPT spectrum of arthritacin E (**5**) (Methanol-d_4).

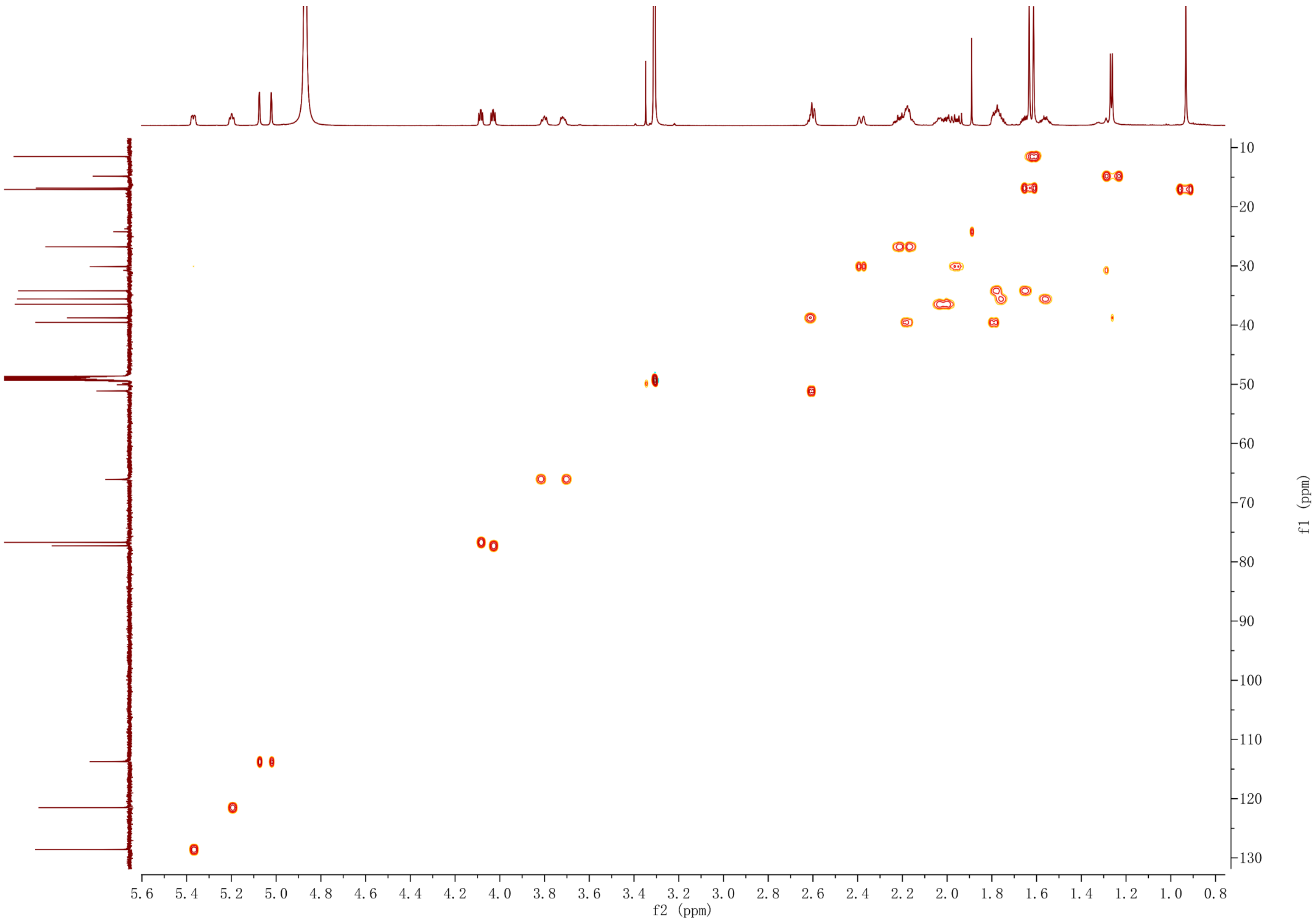

Figure S64. HSQC spectrum of arthritacin E (**5**) (Methanol-d_4).

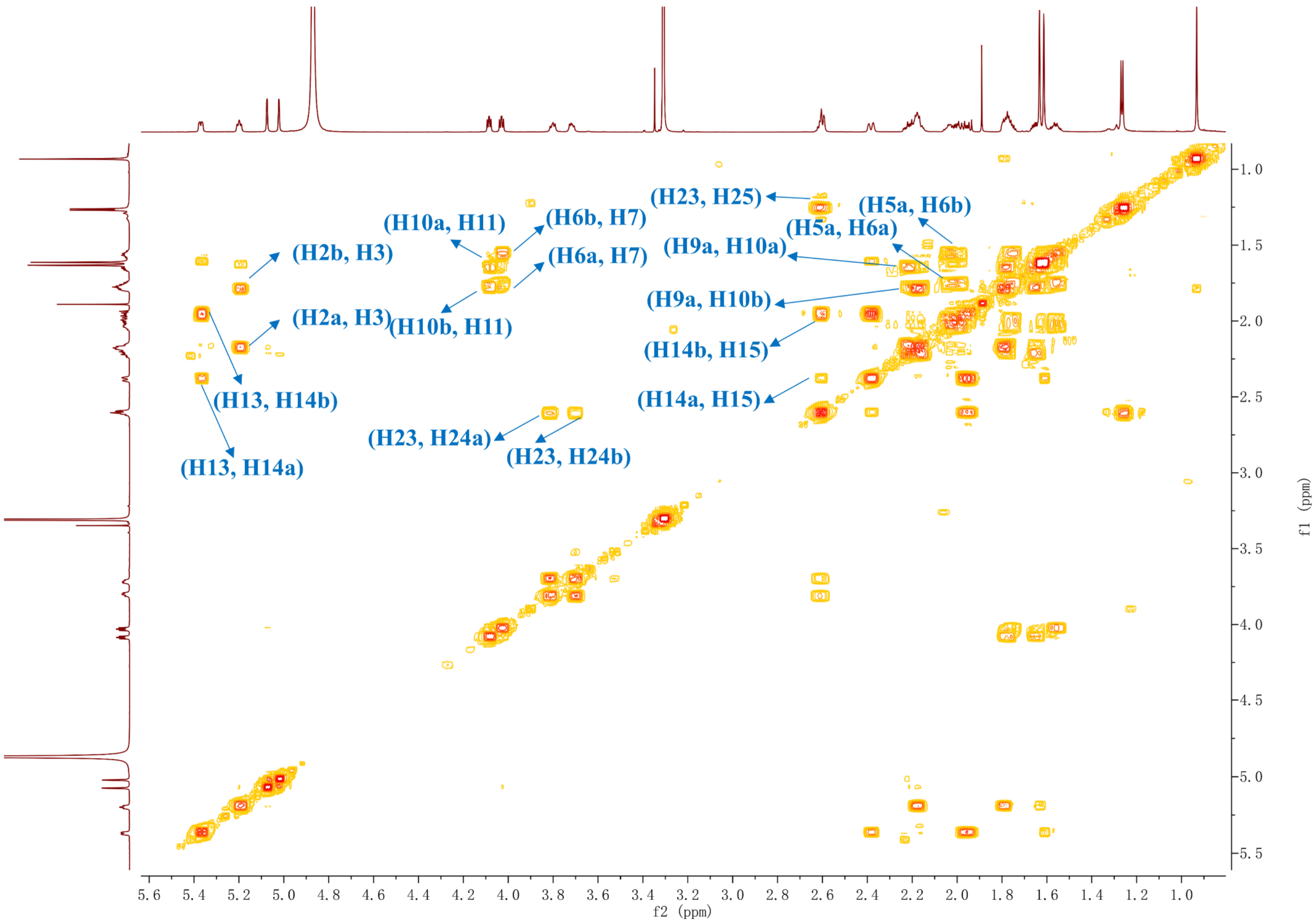

Figure S65. ${}^{1}H$-${}^{1}H$ COSY spectrum of arthritacin E (**5**) (Methanol-d_4) and the selected cross-peaks (blue arrows and texts).

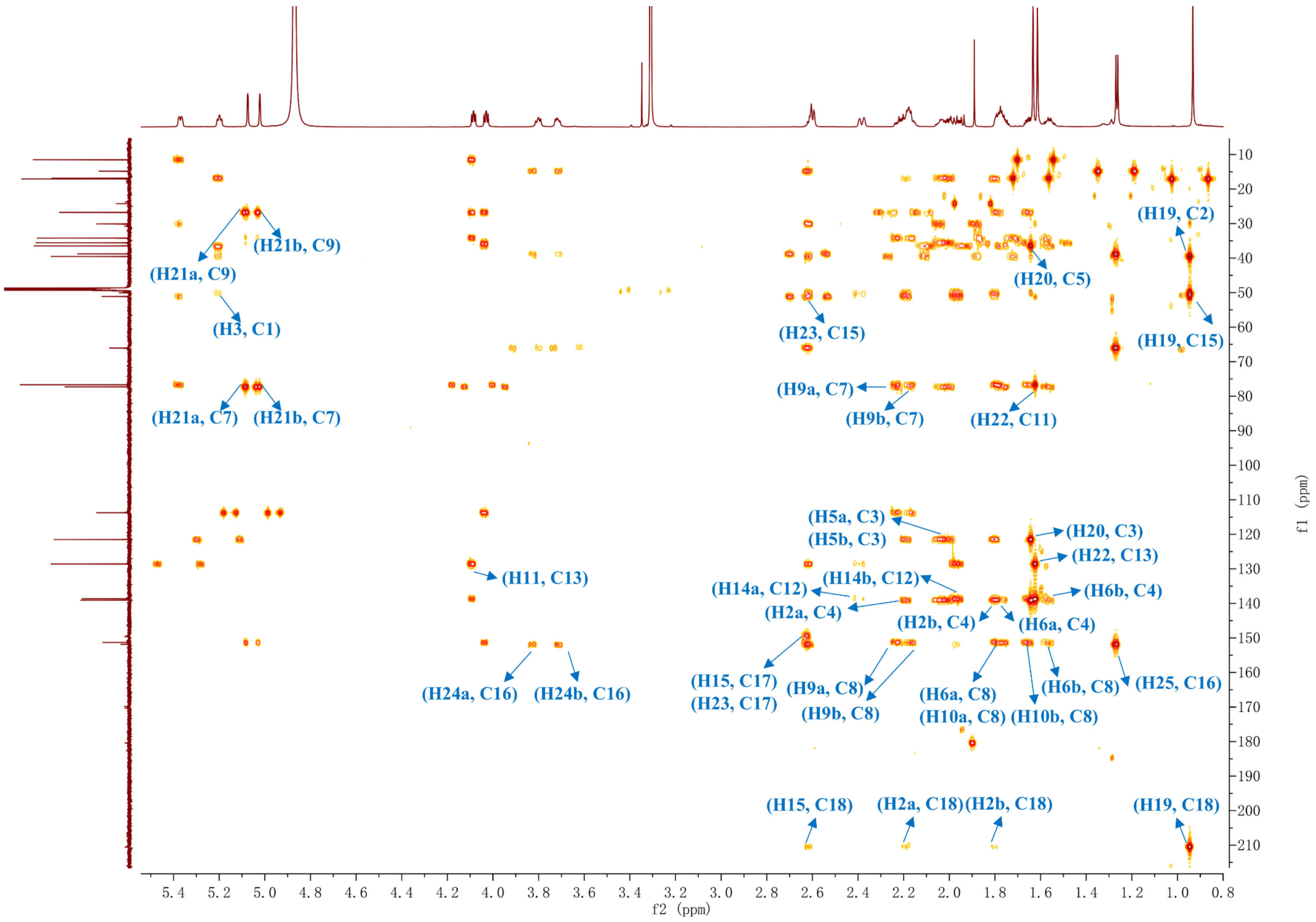

Figure S66. HMBC spectrum of arthritacin E (**5**) (Methanol-d_4) and the selected correlations (blue arrows and texts).

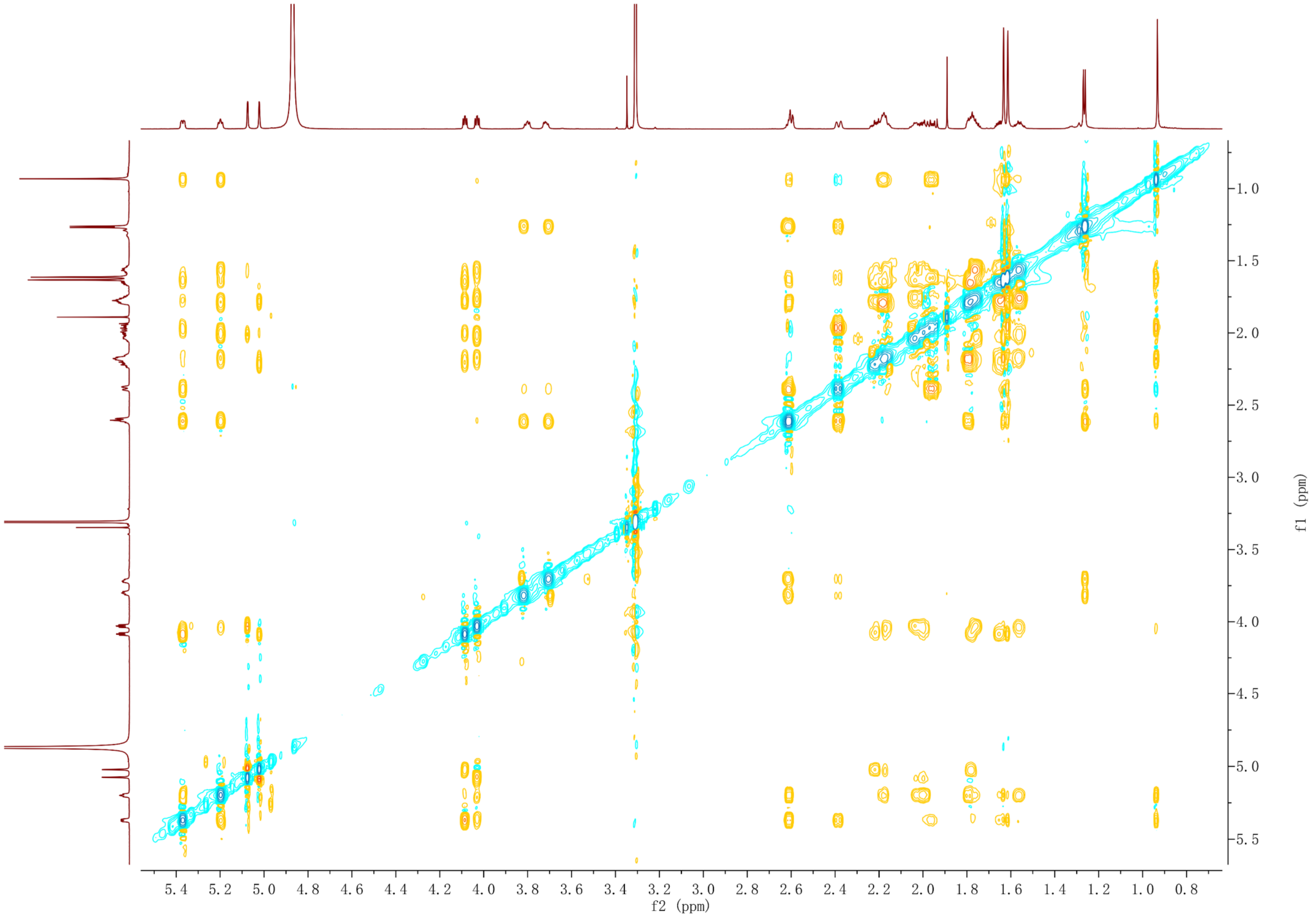

Figure S67. ROESY spectrum of arthritacin E (**5**) (Methanol-d_4) and the selected correlations (blue arrows and texts).

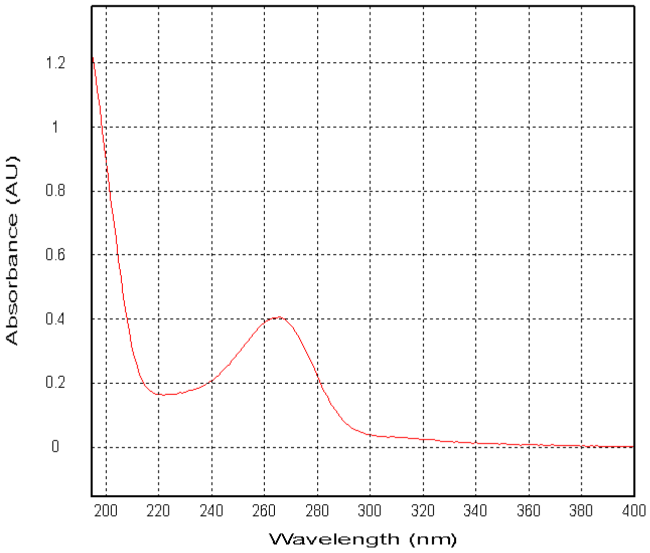

Figure S68. UV spectrum of arthritacin E (**5**).

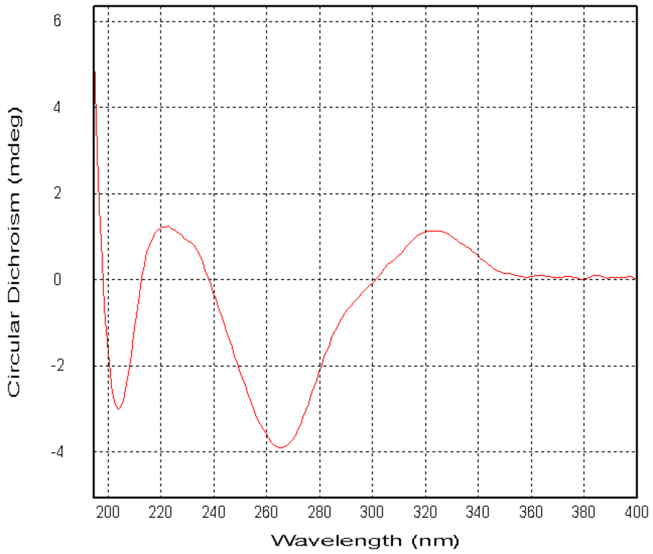

Figure S69. ECD spectrum of arthritacin E (**5**).

Rudolph Research Analytical

This sample was measured on an Autopol VI, Serial #91058
Manufactured by Rudolph Research Analytical, Hackettstown, NJ, USA.

Measurement Date : Friday, 28-MAY-2021

Set Temperature : OFF

Time Delay : Disabled

Delay between Measurement : Disabled

n	Average	Std.Dev.	% RSD	Maximum	Minimum
5	29.26	0.87	2.97	30.29	28.29

S.No	Sample ID	Time	Result	Scale	OR °Arc	WLG.nm	Lg.mm	Conc.g/100ml	Temp.
1	SHSJ46	10:27:07 AM	30.29	SR	0.0212	589	100.00	0.070	25.1
2	SHSJ46	10:27:16 AM	30.00	SR	0.0210	589	100.00	0.070	25.1
3	SHSJ46	10:27:24 AM	29.14	SR	0.0204	589	100.00	0.070	25.1
4	SHSJ46	10:27:32 AM	28.57	SR	0.0200	589	100.00	0.070	25.1
5	SHSJ46	10:27:40 AM	28.29	SR	0.0198	589	100.00	0.070	25.1

Figure S70. ORD report of arthritacin E (**5**).

User Spectra

Fragmentor Voltage	Collision Energy	Ionization Mode
85	0	ESI

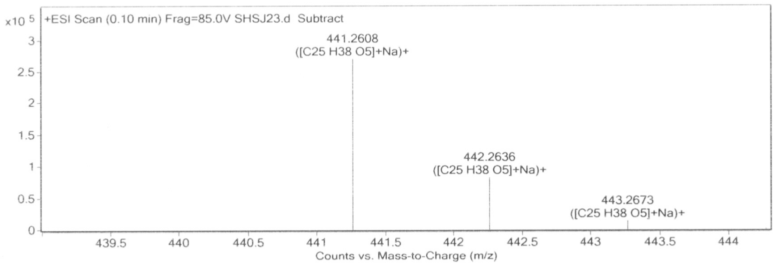

Peak List

m/z	z	Abund	Formula	Ion
62.0796		26226.71		
401.2682	1	80358.44		
402.2721	1	21978.86		
441.2608	1	270078.75	C25 H38 O5	(M+Na)+
442.2636	1	81982.8	C25 H38 O5	(M+Na)+
457.235	1	23741.88		
480.3497	1	38417.58		
859.5308	1	134767.95		
860.5338	1	70701.45		
861.5363	1	21257.1		

Formula Calculator Element Limits

Element	Min	Max
C	3	60
H	0	120
O	0	30

Formula Calculator Results

Formula	CalculatedMass	CalculatedMz	Mz	Diff. (mDa)	Diff. (ppm)	DBE
C25 H38 O5	418.2719	441.2611	441.2608	0.30	0.68	7.0000

--- End Of Report ---

Figure S71. HRESIMS spectrum of arthritacin E (**5**).

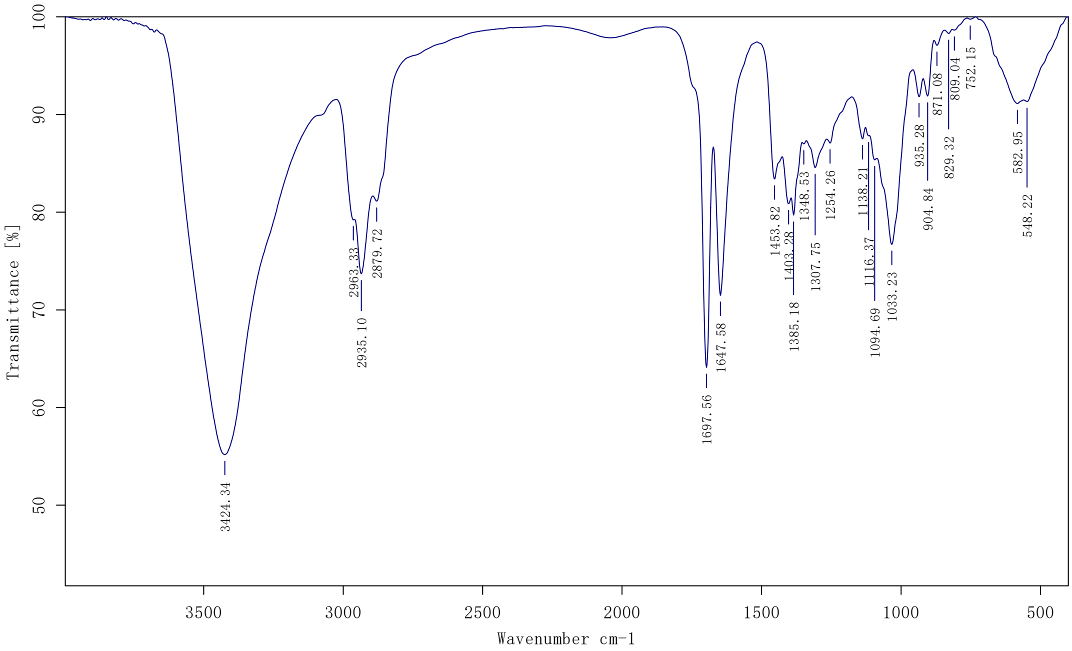

Sample Name: SHSJ23	Resolution: 4	Beamsplitter Setting: KBr
Sample Form: KBr	Aperture Setting: 6 mm	Source Setting: MIR
Path of File: E:\data	Number of Background Scans: 16	Instrument Type: BRUKER VERTEX 70
Date of Measurement: 2025/7/3	Number of Sample Scans: 16	Soft Version: OPUS8.1

Figure S72. IR spectrum of arthritacin E (**5**).

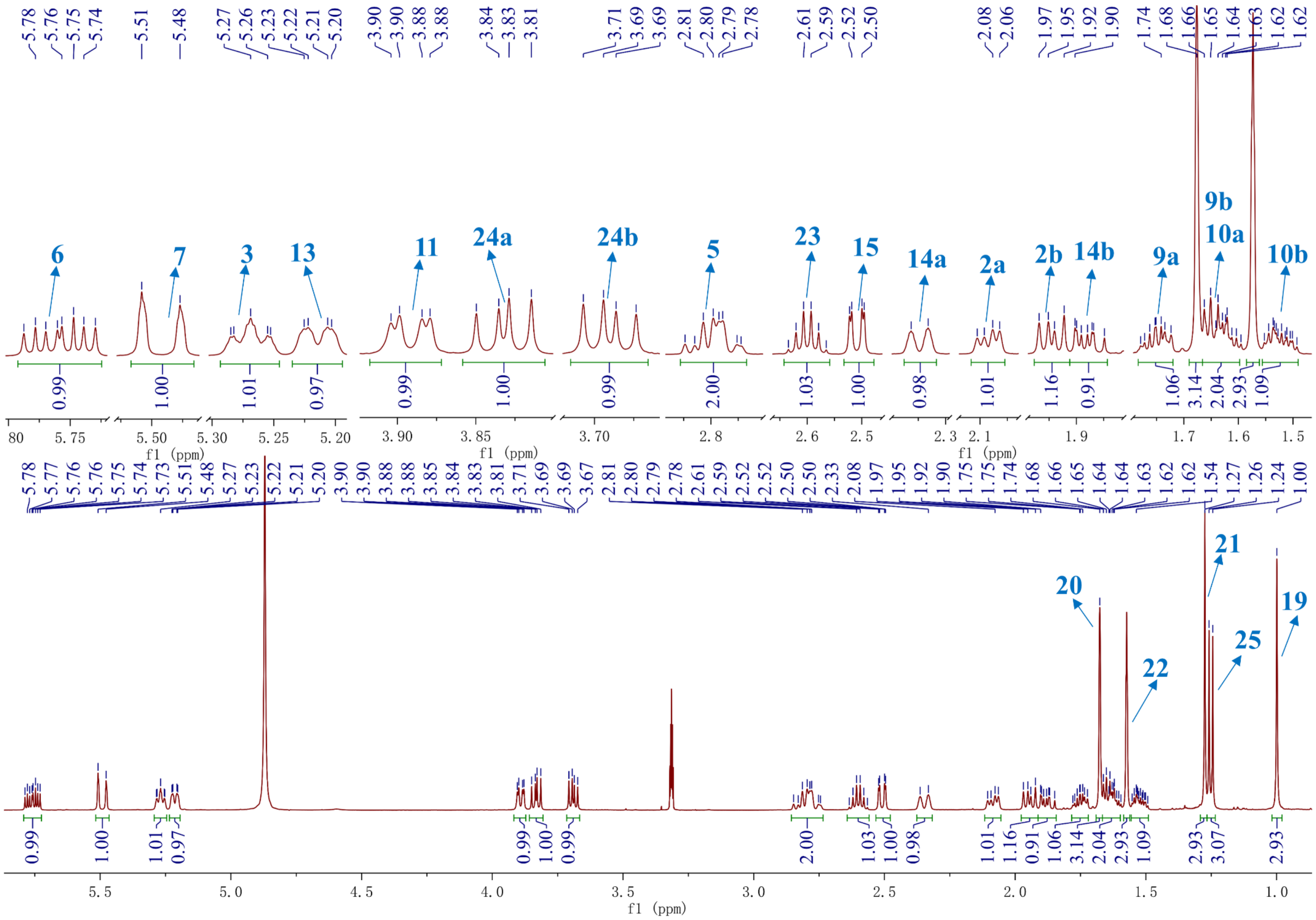

Figure S73. ^{1}H NMR spectrum of arthritacin F (**6**) (Methanol-d_4).

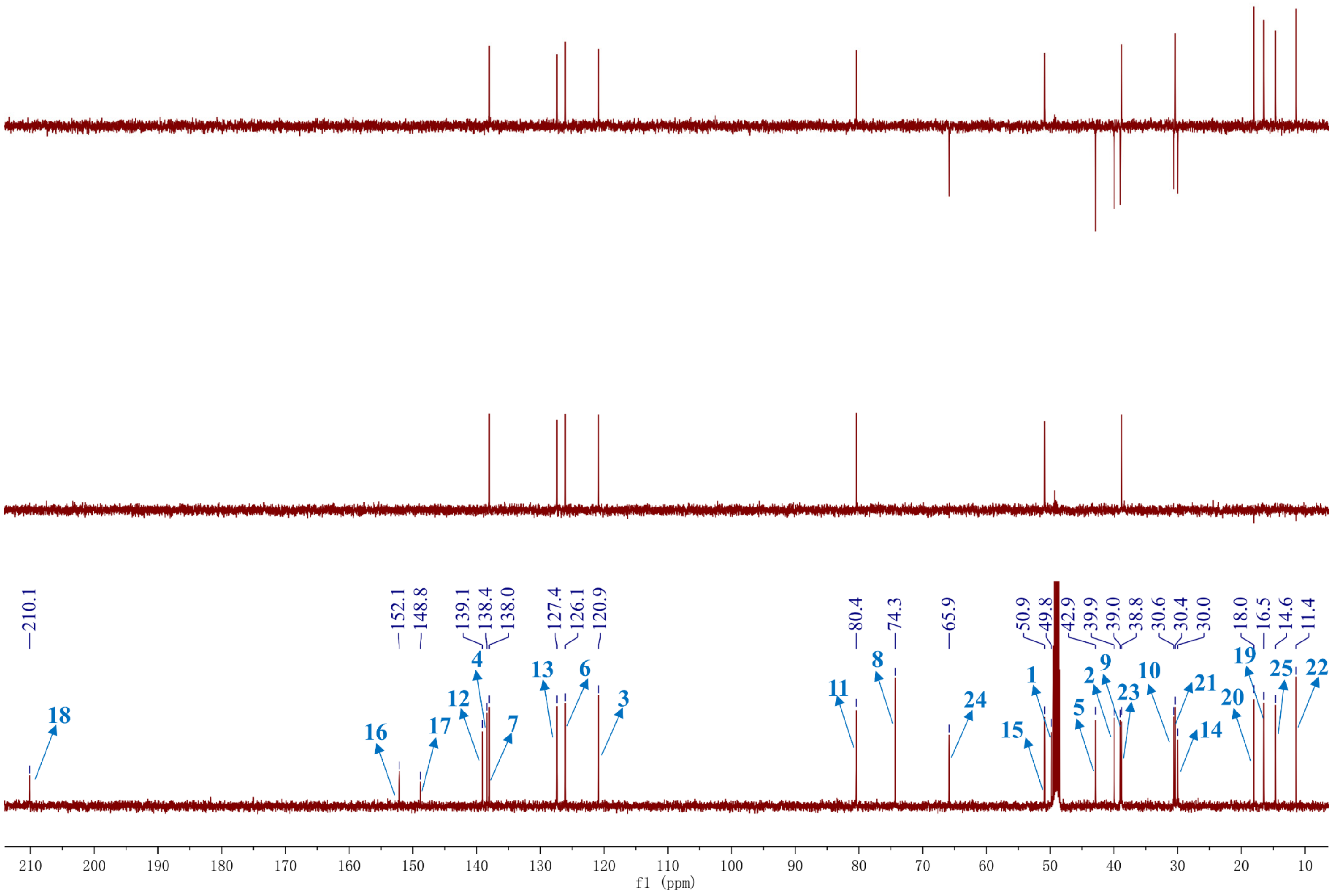

Figure S74. ^{13}C NMR and DEPT spectrum of arthritacin F (**6**) (Methanol-d_4).

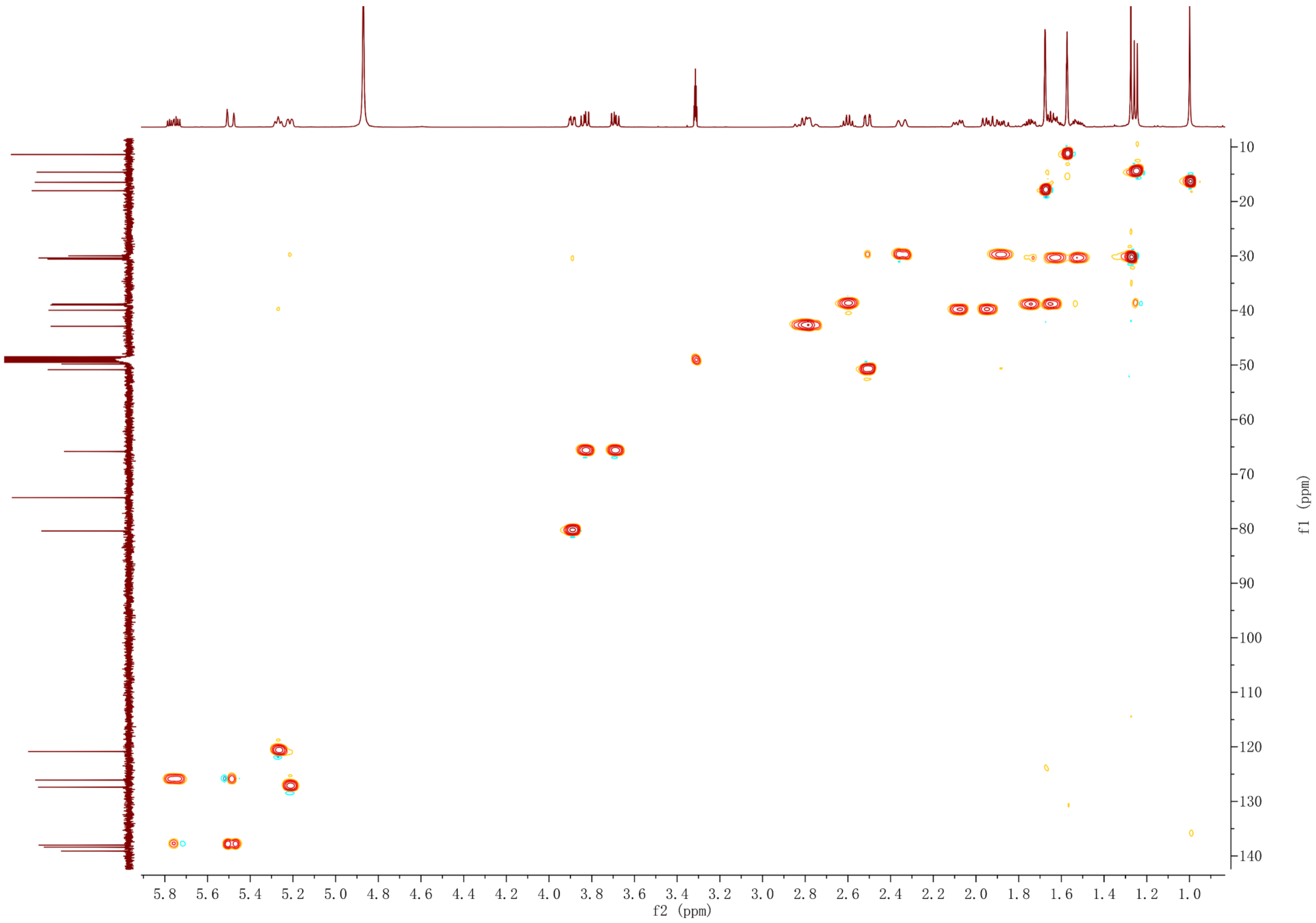

Figure S75. HSQC spectrum of arthritacin F (**6**) (Methanol-d_4).

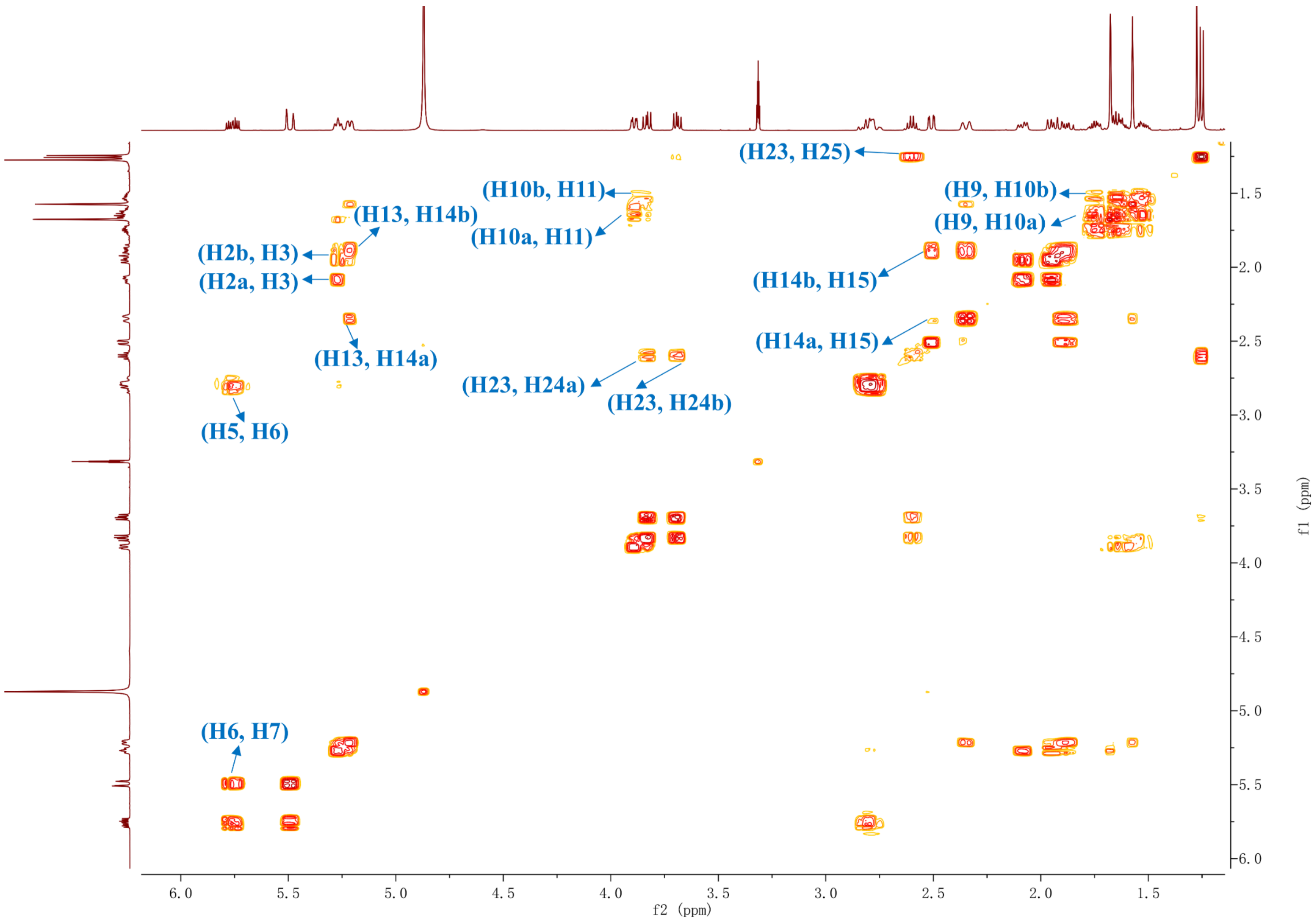

Figure S76. ^{1}H-^{1}H COSY spectrum of arthritacin F (**6**) (Methanol-d_4) and the selected cross-peaks (blue arrows and texts).

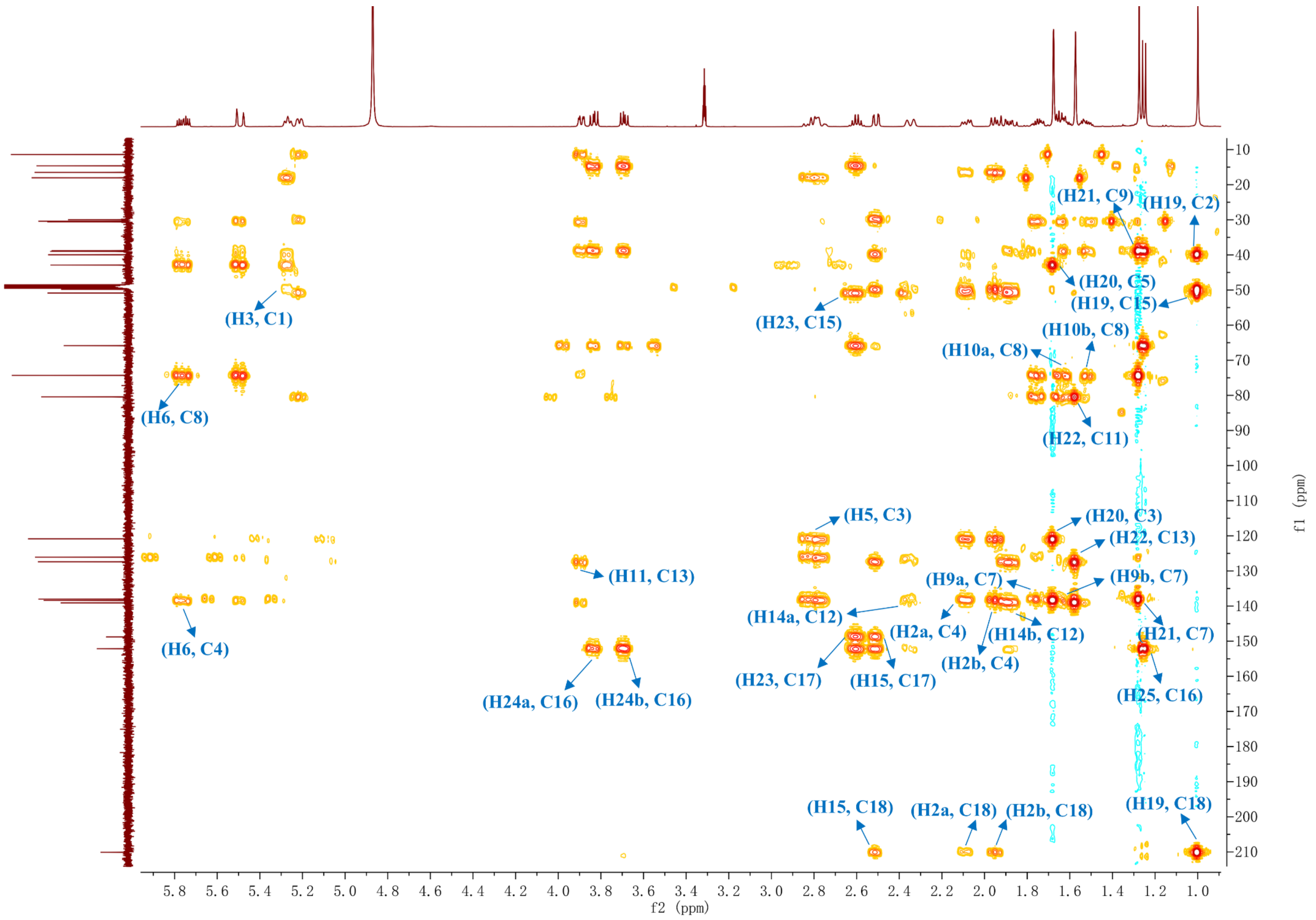

Figure S77. HMBC spectrum of arthritacin F (**6**) (Methanol-d_4) and the selected correlations (blue arrows and texts).

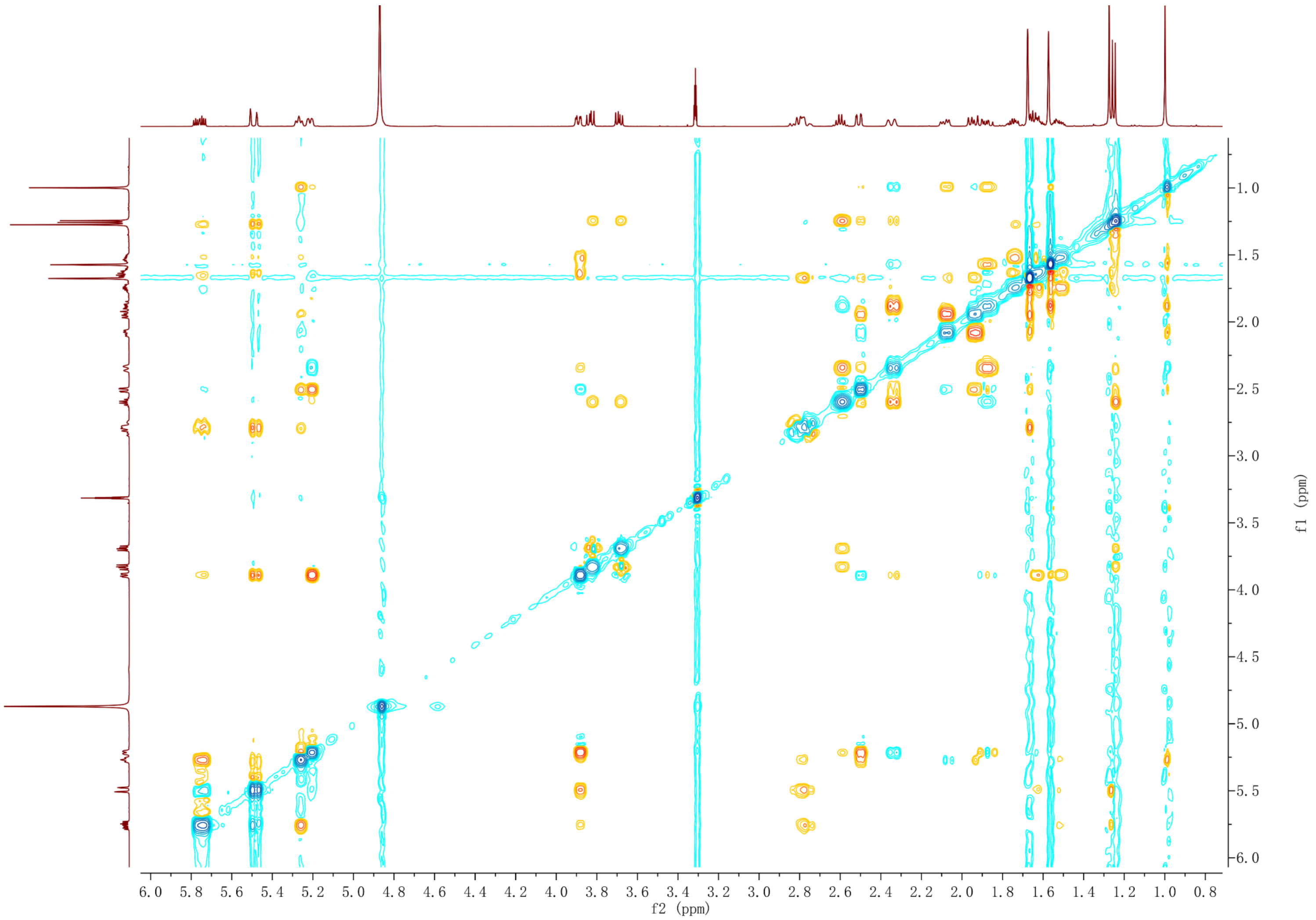

Figure S78. ROESY spectrum of arthritacin F (**6**) (Methanol-d_4) and the selected correlations (blue arrows and texts).

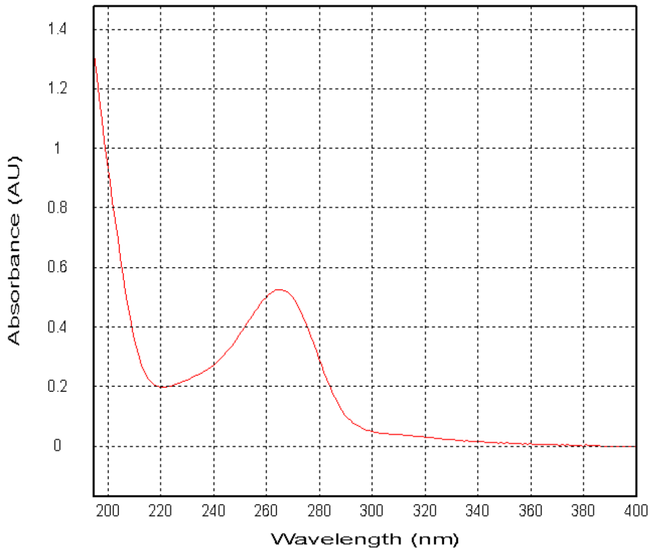

Figure S79. UV spectrum of arthritacin F (**6**).

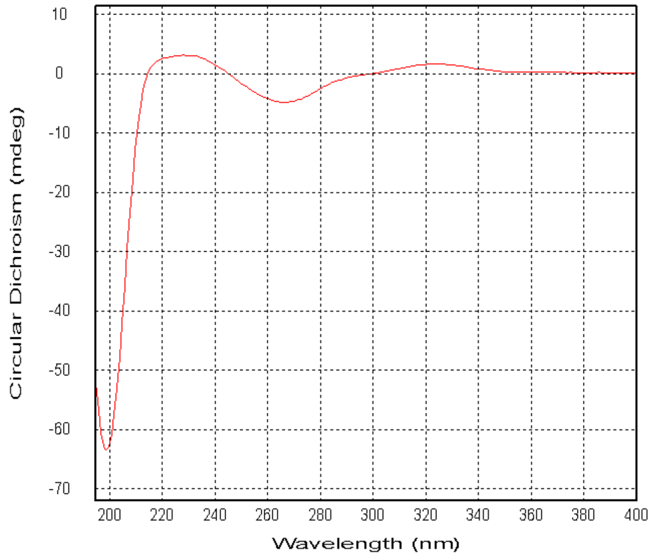

Figure S80. ECD spectrum of arthritacin F (**6**).

Rudolph Research Analytical

This sample was measured on an Autopol VI, Serial #91058
Manufactured by Rudolph Research Analytical, Hackettstown, NJ, USA.

Measurement Date : Friday, 28-MAY-2021

Set Temperature : OFF

Time Delay : Disabled

Delay between Measurement : Disabled

n	Average	Std.Dev.	% RSD	Maximum	Minimum
5	-31.07	0.34	-1.09	-30.87	-31.67

S.No	Sample ID	Time	Result	Scale	OR °Arc	WLG.nm	Lg.mm	Conc.g/100ml	Temp.
1	SHSJ22	11:10:50 AM	-30.87	SR	-0.0463	589	100.00	0.150	25.7
2	SHSJ22	11:10:58 AM	-30.87	SR	-0.0463	589	100.00	0.150	25.7
3	SHSJ22	11:11:06 AM	-31.00	SR	-0.0465	589	100.00	0.150	25.7
4	SHSJ22	11:11:14 AM	-30.93	SR	-0.0464	589	100.00	0.150	25.7
5	SHSJ22	11:11:23 AM	-31.67	SR	-0.0475	589	100.00	0.150	25.7

Figure S81. ORD report of arthritacin F (**6**).

User Spectra

Fragmentor Voltage	Collision Energy	Ionization Mode
135	0	ESI

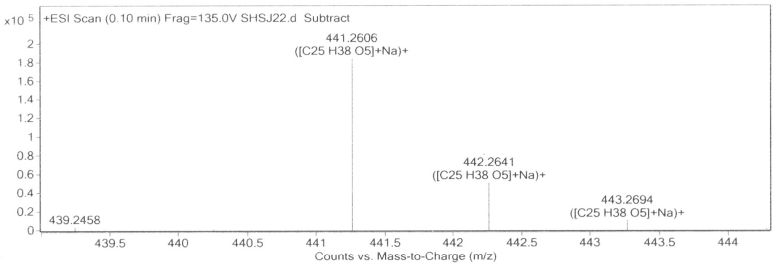

Peak List

m/z	z	Abund	Formula	Ion
383.2573	1	21521.98		
401.268	1	58534.31		
419.2788	1	149177.63		
420.2821	1	37957.27		
441.2606	1	184038.89	C25 H38 O5	(M+Na)+
442.2641	1	50619.09	C25 H38 O5	(M+Na)+
457.2354	1	21393.8		
859.5326	1	129515.99		
860.5353	1	70916.5		
861.5391	1	20052.4		

Formula Calculator Element Limits

Element	Min	Max
C	3	60
H	0	120
O	0	30

Formula Calculator Results

Formula	CalculatedMass	CalculatedMz	Mz	Diff. (mDa)	Diff. (ppm)	DBE
C25 H38 O5	418.2719	441.2611	441.2606	0.50	1.13	7.0000

--- End Of Report ---

Figure S82. HRESIMS spectrum of arthritacin F (**6**).

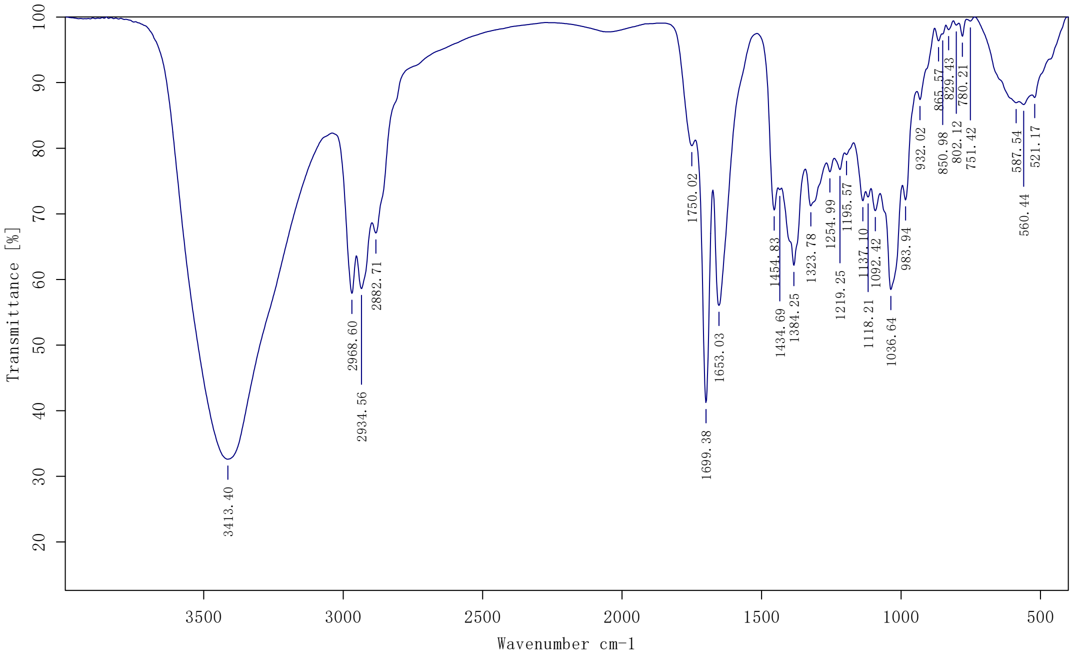

Sample Name: SHSJ22	Resolution: 4	Beamsplitter Setting: KBr
Sample Form: KBr	Aperture Setting: 6 mm	Source Setting: MIR
Path of File: E:\data	Number of Background Scans: 16	Instrument Type: BRUKER VERTEX 70
Date of Measurement: 2025/7/3	Number of Sample Scans: 16	Soft Version: OPUS8.1

Figure S83. IR spectrum of arthritacin F (**6**).

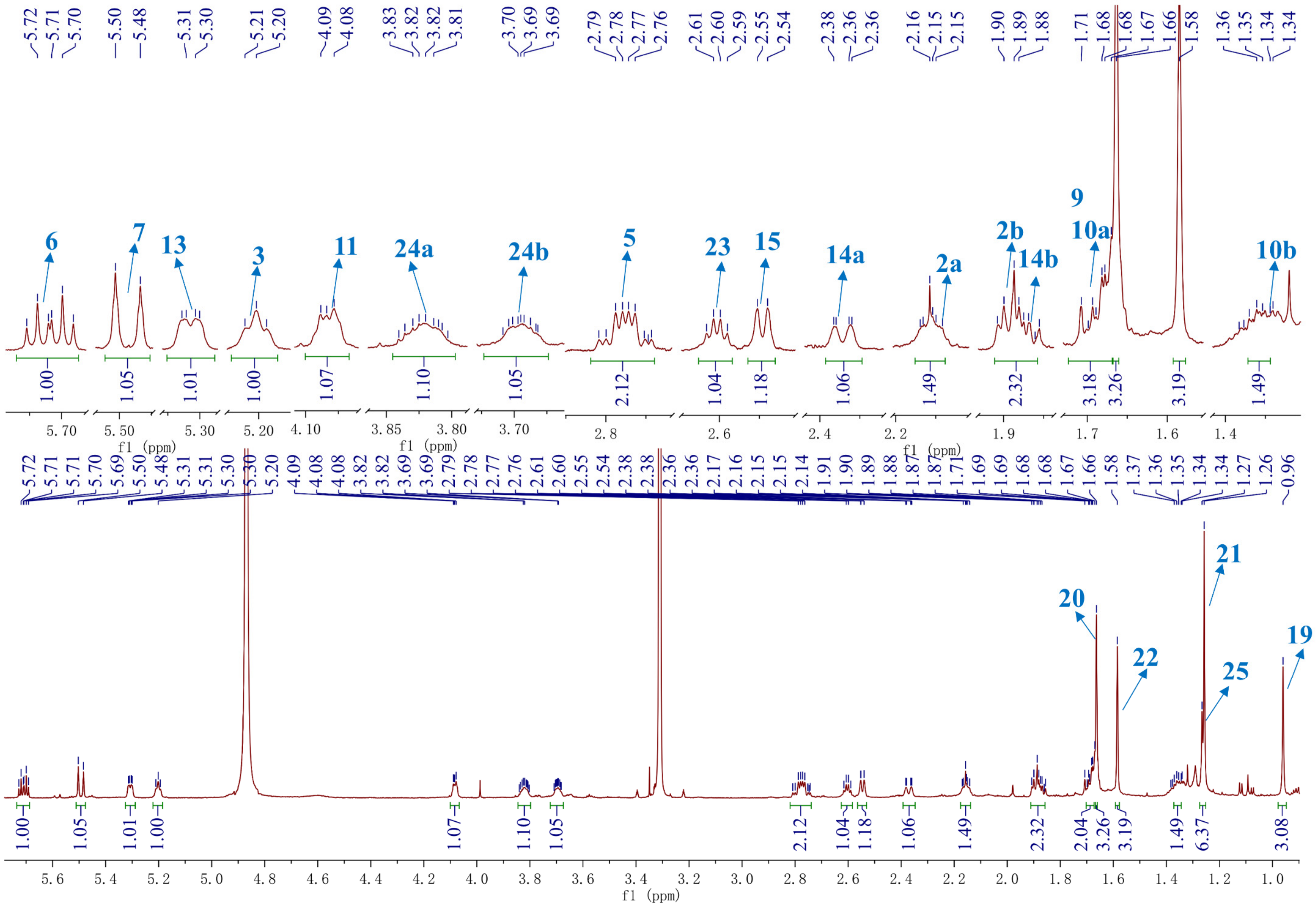

Figure S84. ^{1}H NMR spectrum of arthritacin G (**7**) (Methanol-d_4).

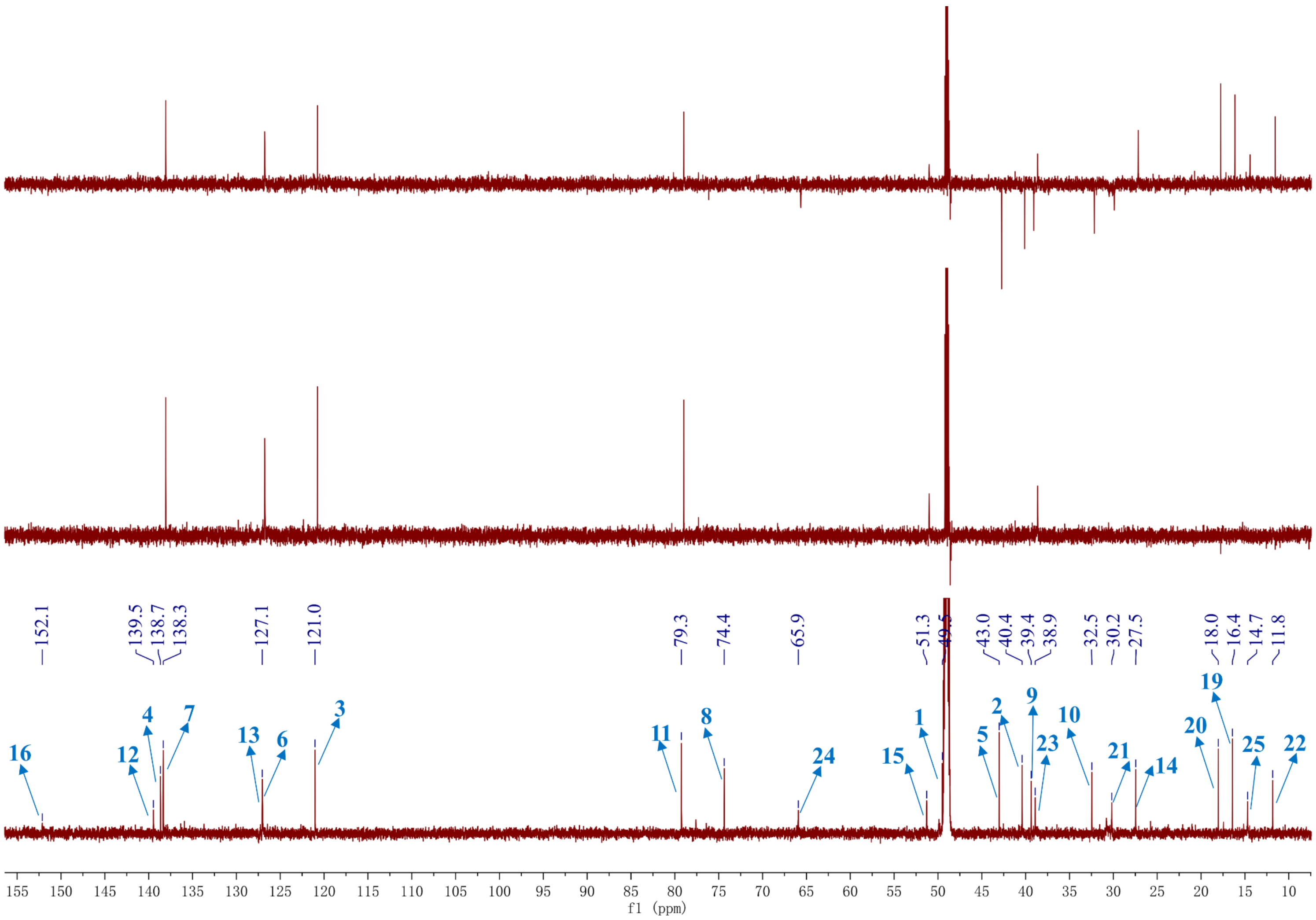

Figure S85. ^{13}C NMR and DEPT spectrum of arthritacin G (**7**) (Methanol-d_4).

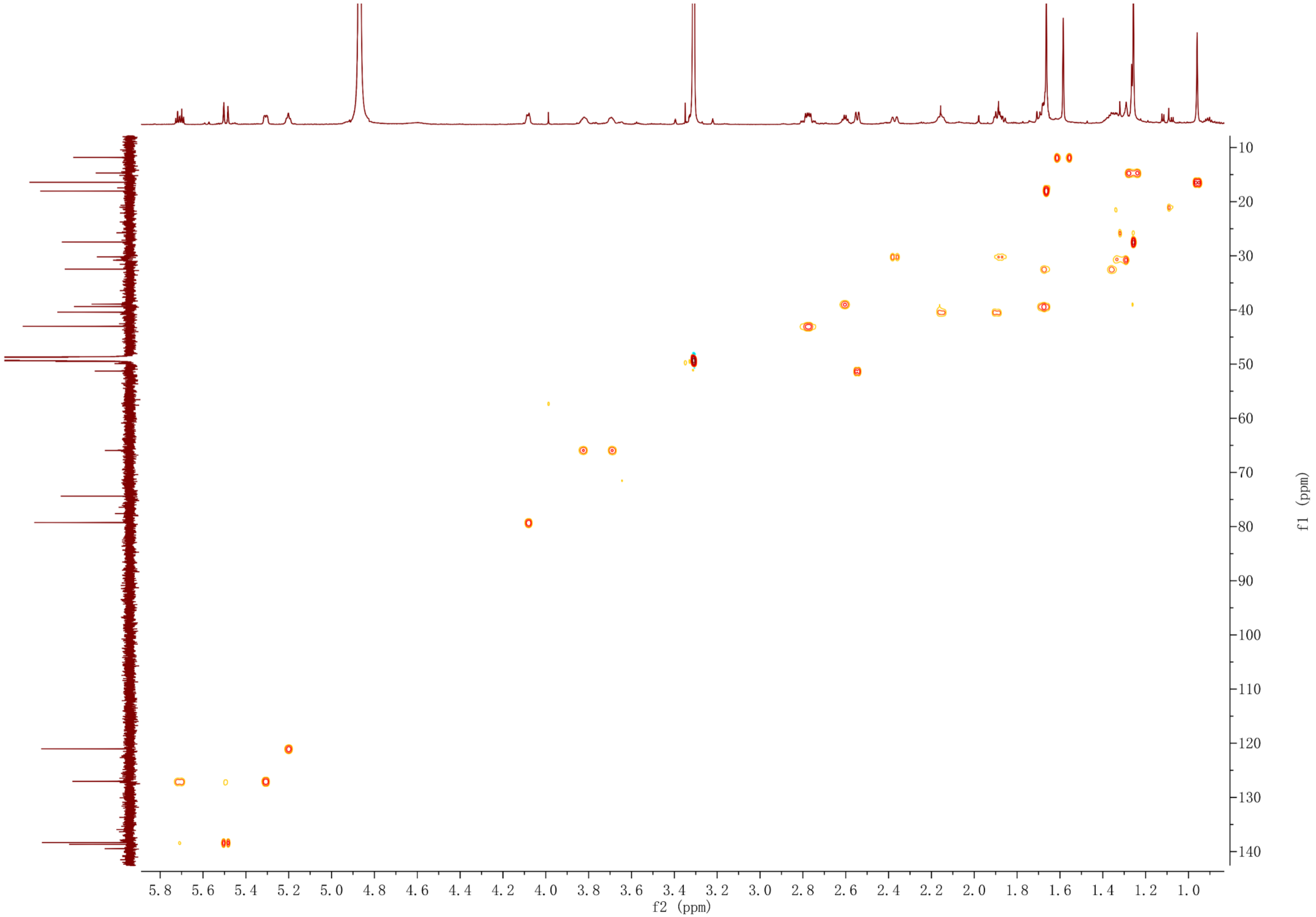

Figure S86. HSQC spectrum of arthritacin G (**7**) (Methanol-d_4).

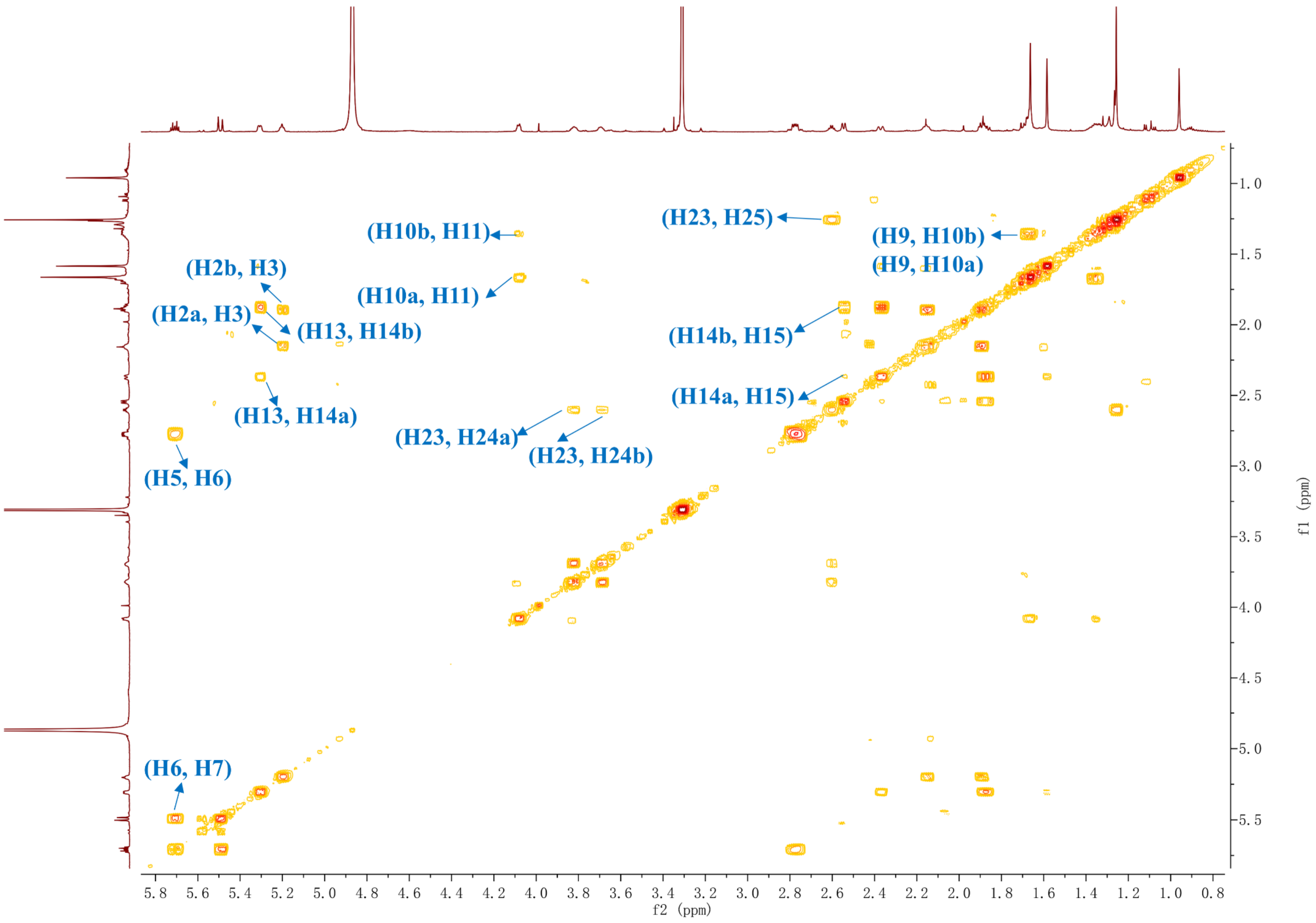

Figure S87. ^{1}H-^{1}H COSY spectrum of arthritacin G (**7**) (Methanol-d_4) and the selected cross-peaks (blue arrows and texts).

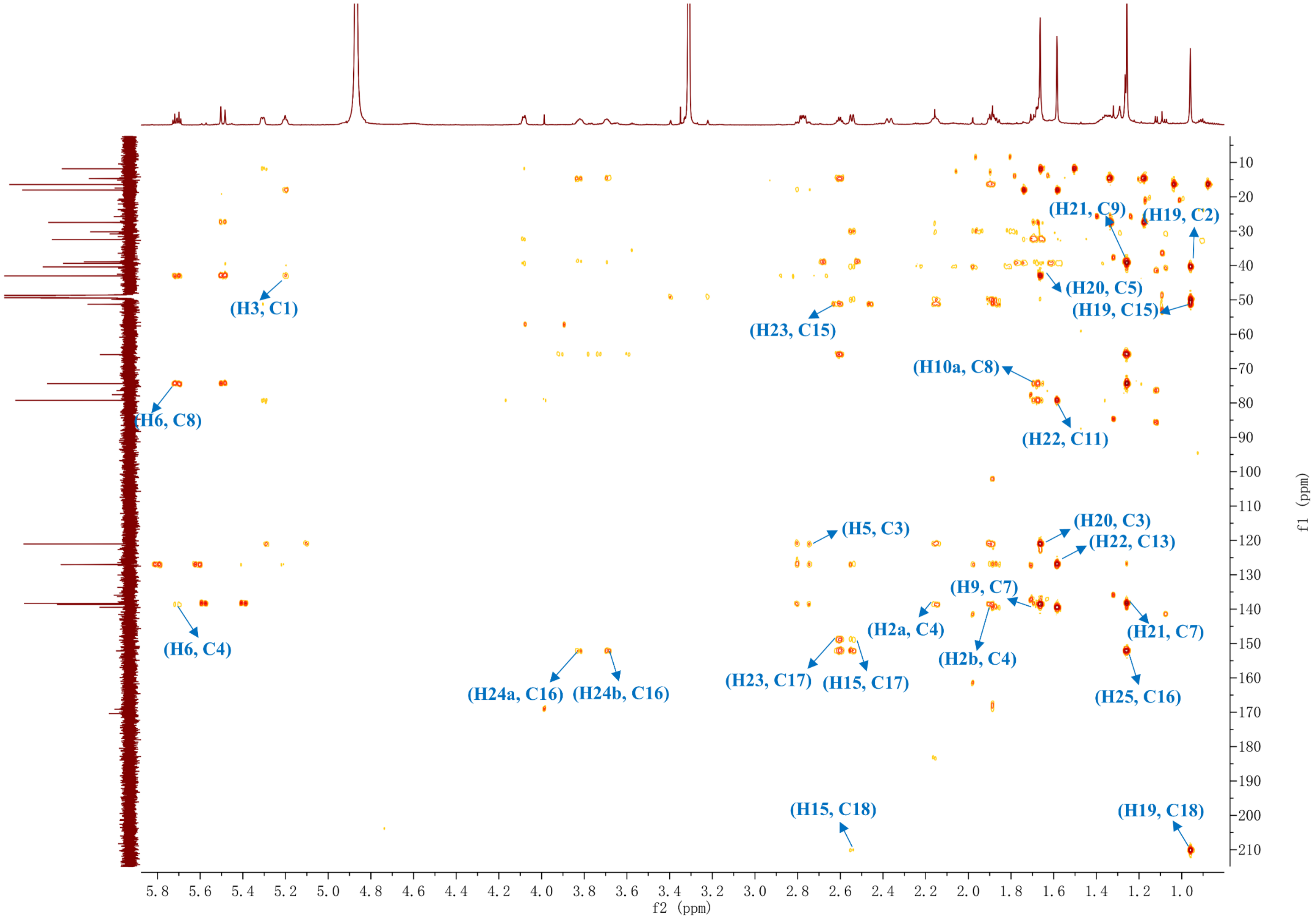

Figure S88. HMBC spectrum of arthritacin G (**7**) (Methanol-d_4) and the selected correlations (blue arrows and texts).

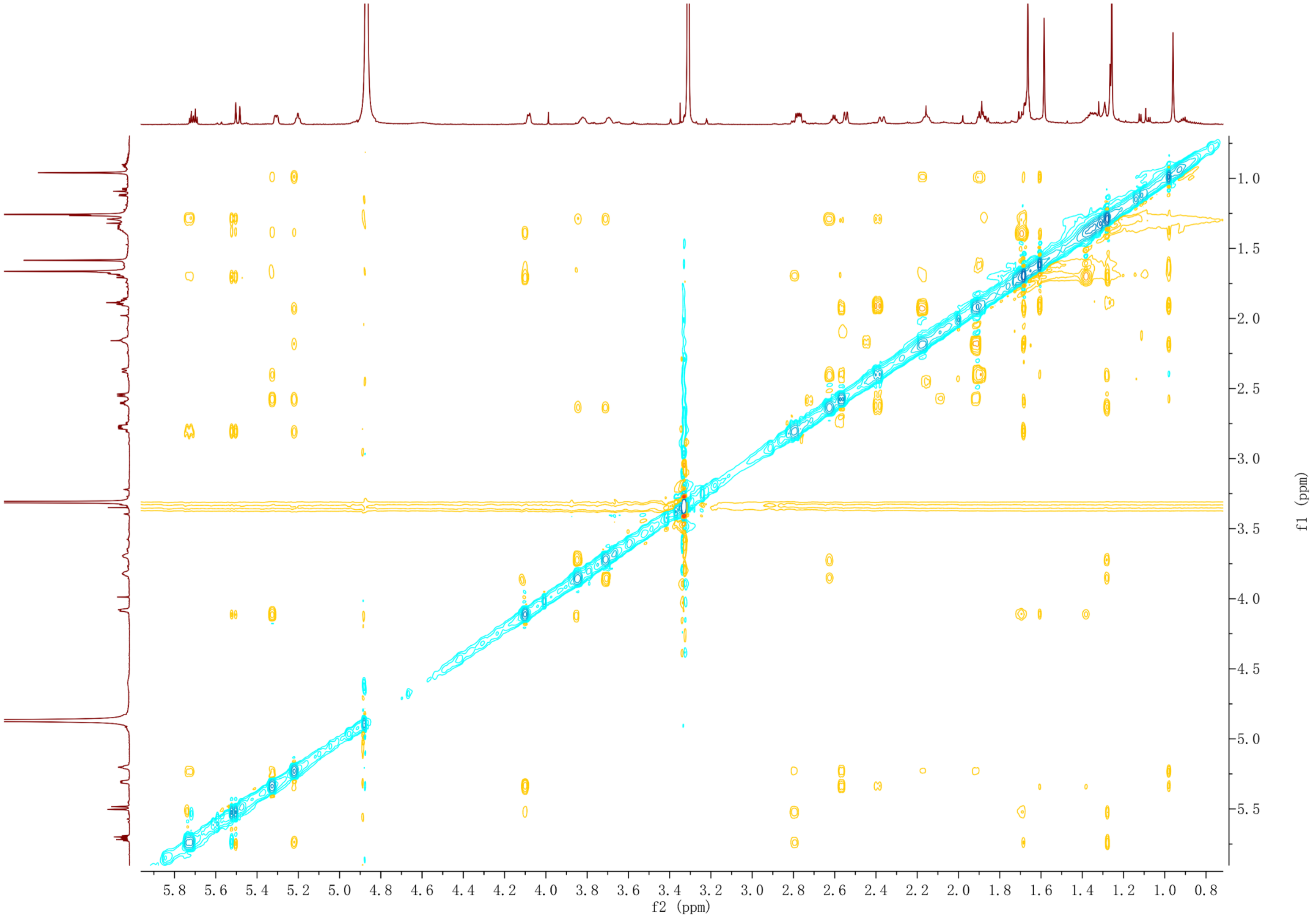

Figure S89. ROESY spectrum of arthritacin G (**7**) (Methanol-d_4) and the selected correlations (blue arrows and texts).

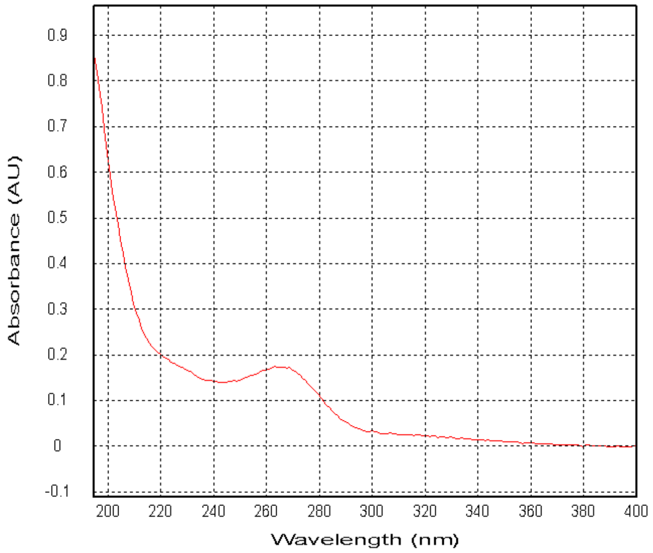

Figure S90. UV spectrum of arthritacin G (**7**).

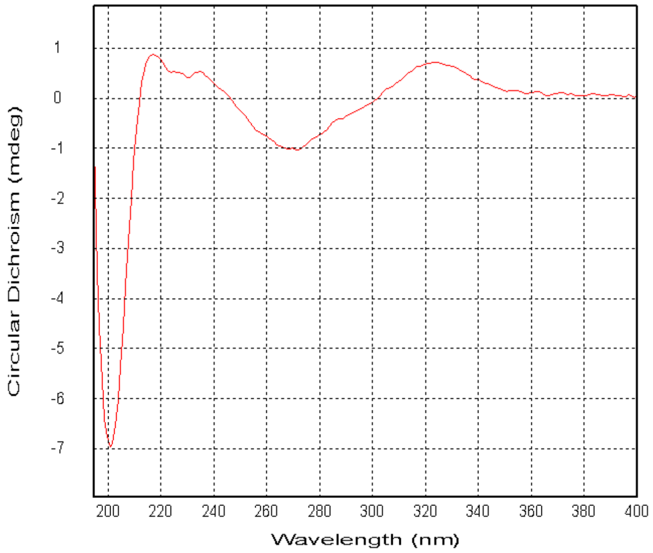

Figure S91. ECD spectrum of arthritacin G (**7**).

Rudolph Research Analytical

This sample was measured on an Autopol VI, Serial #91058
Manufactured by Rudolph Research Analytical, Hackettstown, NJ, USA.

Measurement Date : Monday, 21-APR-2025

Set Temperature : OFF

Time Delay : Disabled

Delay between Measurement : Disabled

n	Average	Std.Dev.	% RSD	Maximum	Minimum
5	-2.79	0.69	-24.73	-2.12	-3.64

S.No	Sample ID	Time	Result	Scale	OR °Arc	WLG.nm	Lg.mm	Conc.g/100ml	Temp.
1	SHSH24	02:21:44 PM	-3.33	SR	-0.0011	589	100.00	0.033	26.8
2	SHSH24	02:21:52 PM	-3.64	SR	-0.0012	589	100.00	0.033	26.8
3	SHSH24	02:22:01 PM	-2.73	SR	-0.0009	589	100.00	0.033	26.9
4	SHSH24	02:22:09 PM	-2.12	SR	-0.0007	589	100.00	0.033	26.9
5	SHSH24	02:22:17 PM	-2.12	SR	-0.0007	589	100.00	0.033	26.9

Figure S92. ORD report of arthritacin G (**7**).

User Spectra

Fragmentor Voltage	Collision Energy	Ionization Mode
135	0	ESI

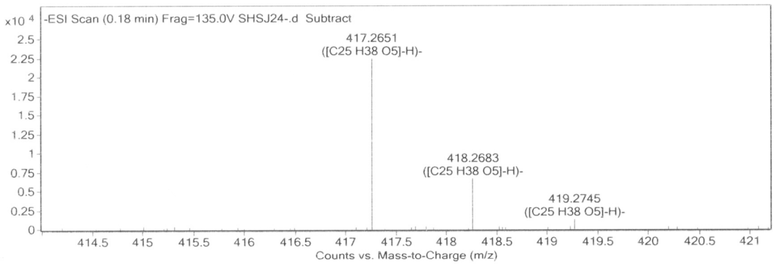

Peak List

m/z	z	Abund	Formula	Ion
89.0245	1	26933.06		
112.9855		23202.64		
119.0362		14402.92		
255.2333	1	19698.17		
283.2647	1	16313.44		
417.2651	1	22458.1	C25 H38 O5	(M-H)-
955.9713	1	7141.02		
1010.0247	1	11033.77		
1033.9881	1	52888.38		
1034.9913	1	10721.09		

Formula Calculator Element Limits

Element	Min	Max
C	3	60
H	0	120
O	0	30

Formula Calculator Results

Formula	CalculatedMass	CalculatedMz	Mz	Diff. (mDa)	Diff. (ppm)	DBE
C25 H38 O5	418.2719	417.2646	417.2651	-0.50	-1.20	7.0000

--- End Of Report ---

Figure S93. HRESIMS spectrum of arthritacin G (**7**).

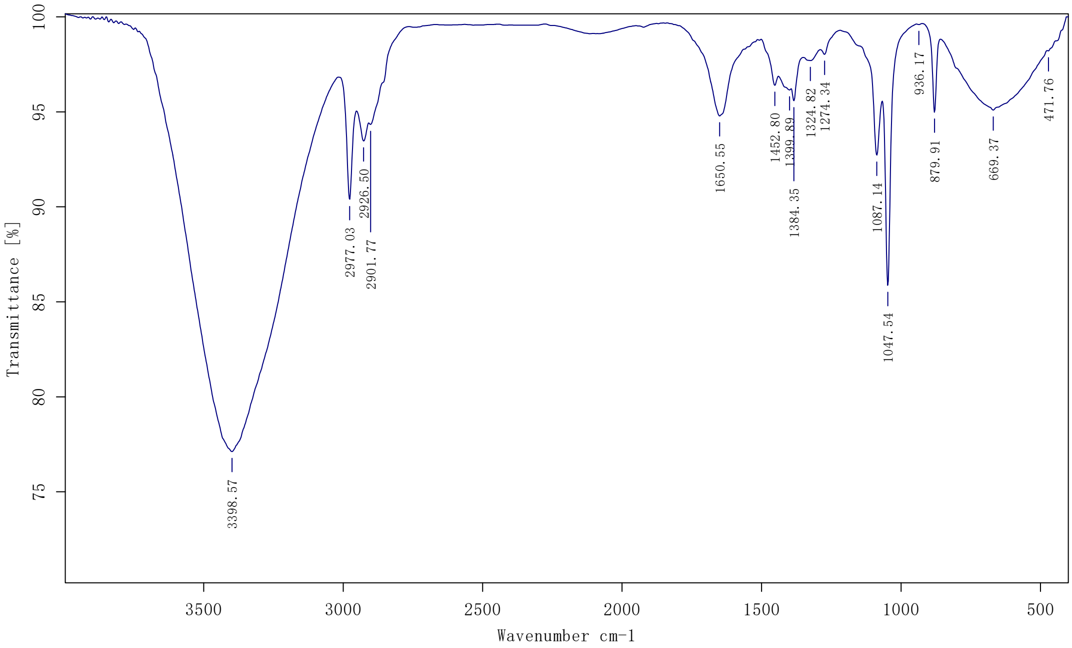

Sample Name: SHSJ24
Sample Form: KBr
Path of File: E:\data
Date of Measurement: 2025/7/3

Resolution: 4
Aperture Setting: 6 mm
Number of Background Scans: 16
Number of Sample Scans: 16

Beamsplitter Setting: KBr
Source Setting: MIR
Instrument Type: BRUKER VERTEX 70
Soft Version: OPUS8.1

Figure S94. IR spectrum of arthritacin G (**7**).

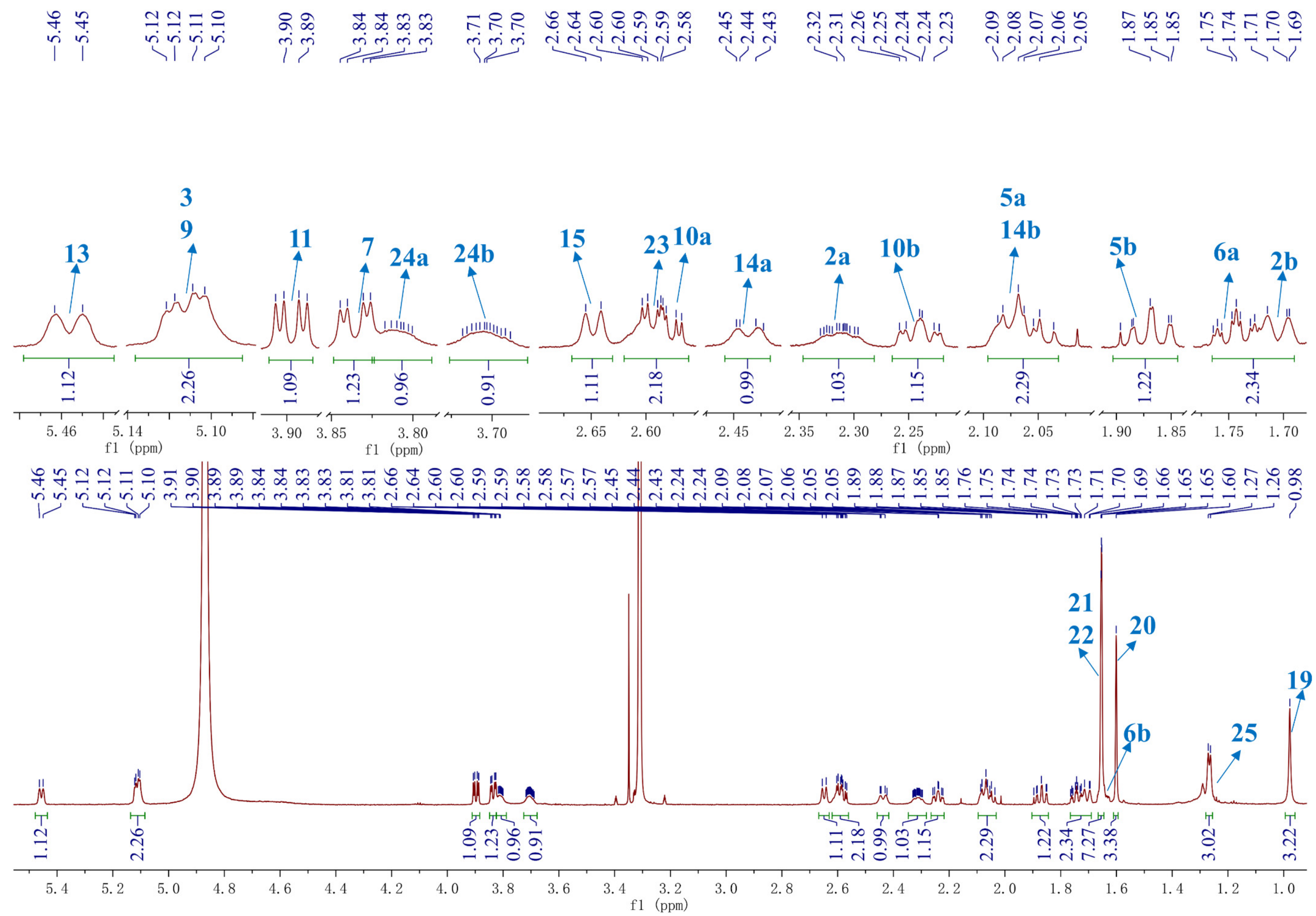

Figure S95. ^{1}H NMR spectrum of arthritacin H (**8**) (Methanol-d_4).

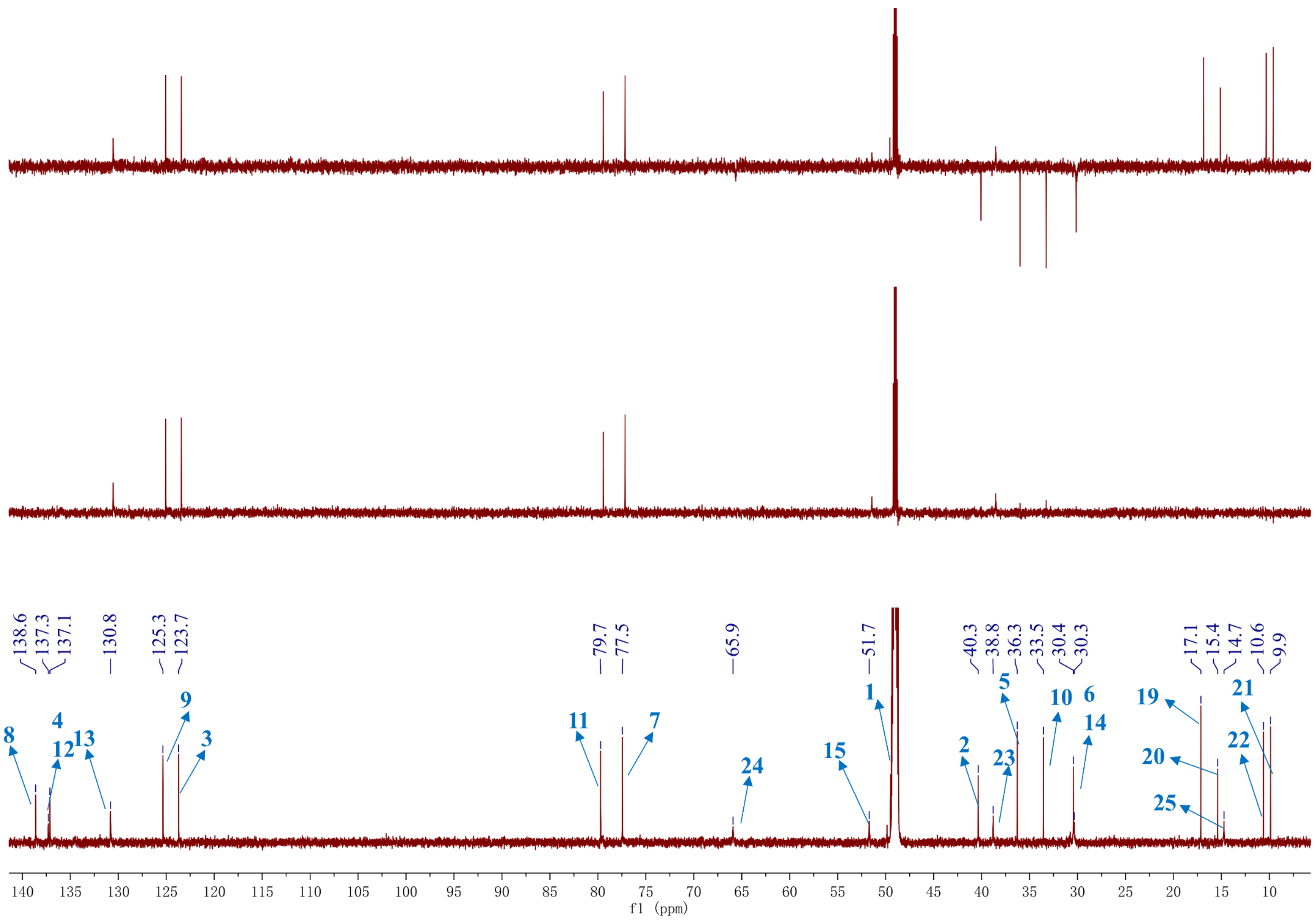

Figure S96. ^{13}C NMR and DEPT spectrum of arthritacin H (**8**) (Methanol-d_4).

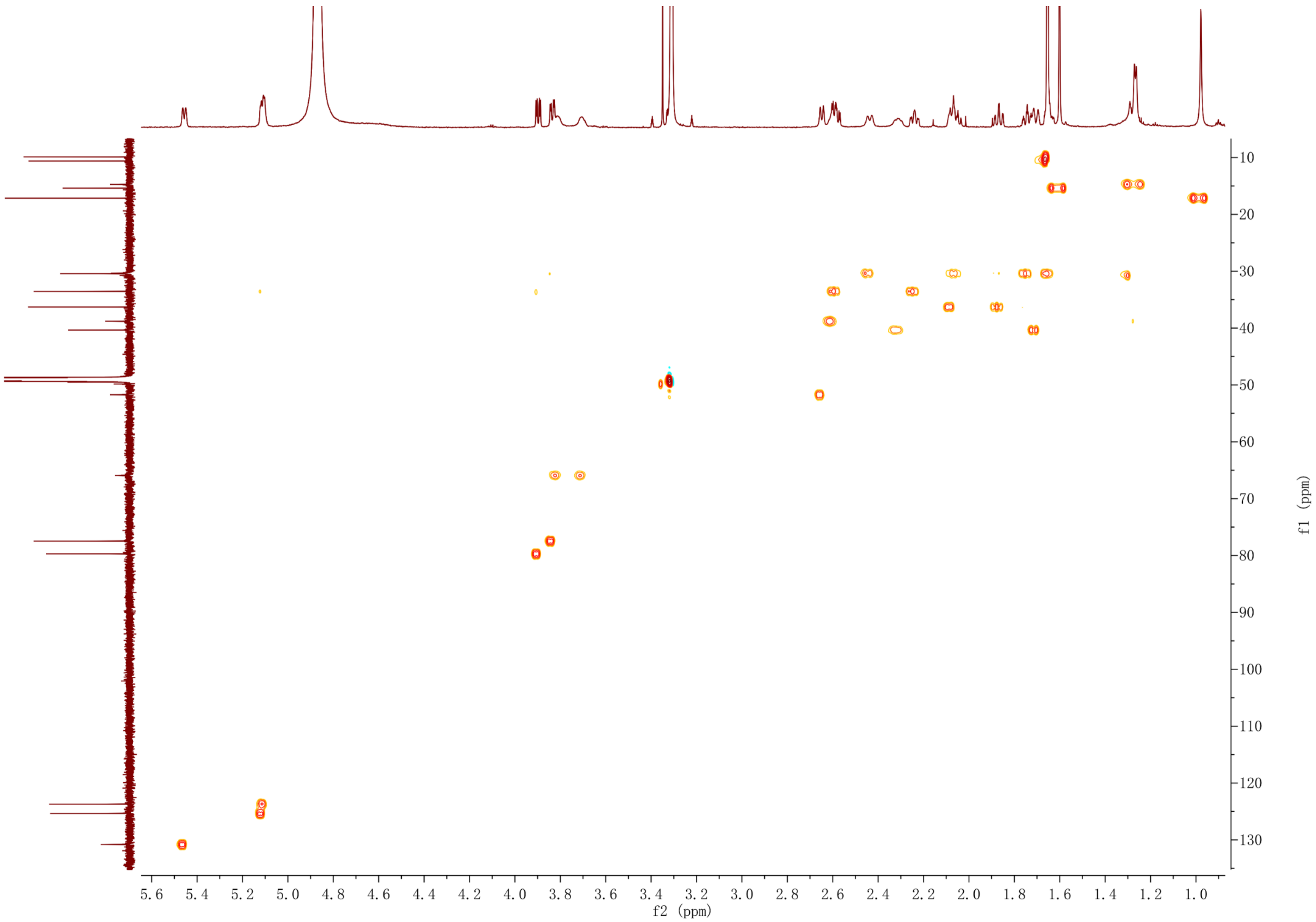

Figure S97. HSQC spectrum of arthritacin H (**8**) (Methanol-d_4).

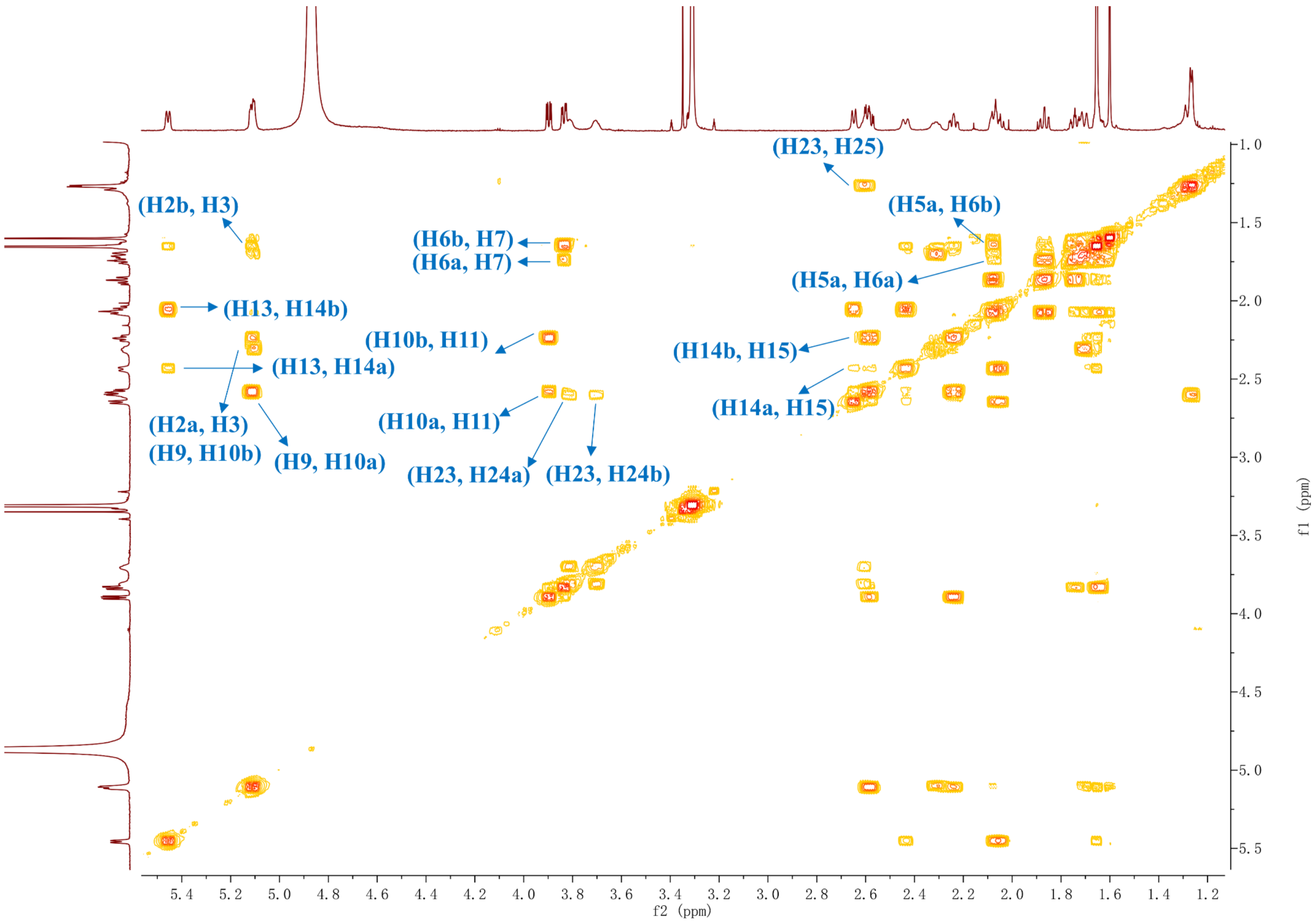

Figure S98. ^{1}H-^{1}H COSY spectrum of arthritacin H (**8**) (Methanol-d_4) and the selected cross-peaks (blue arrows and texts).

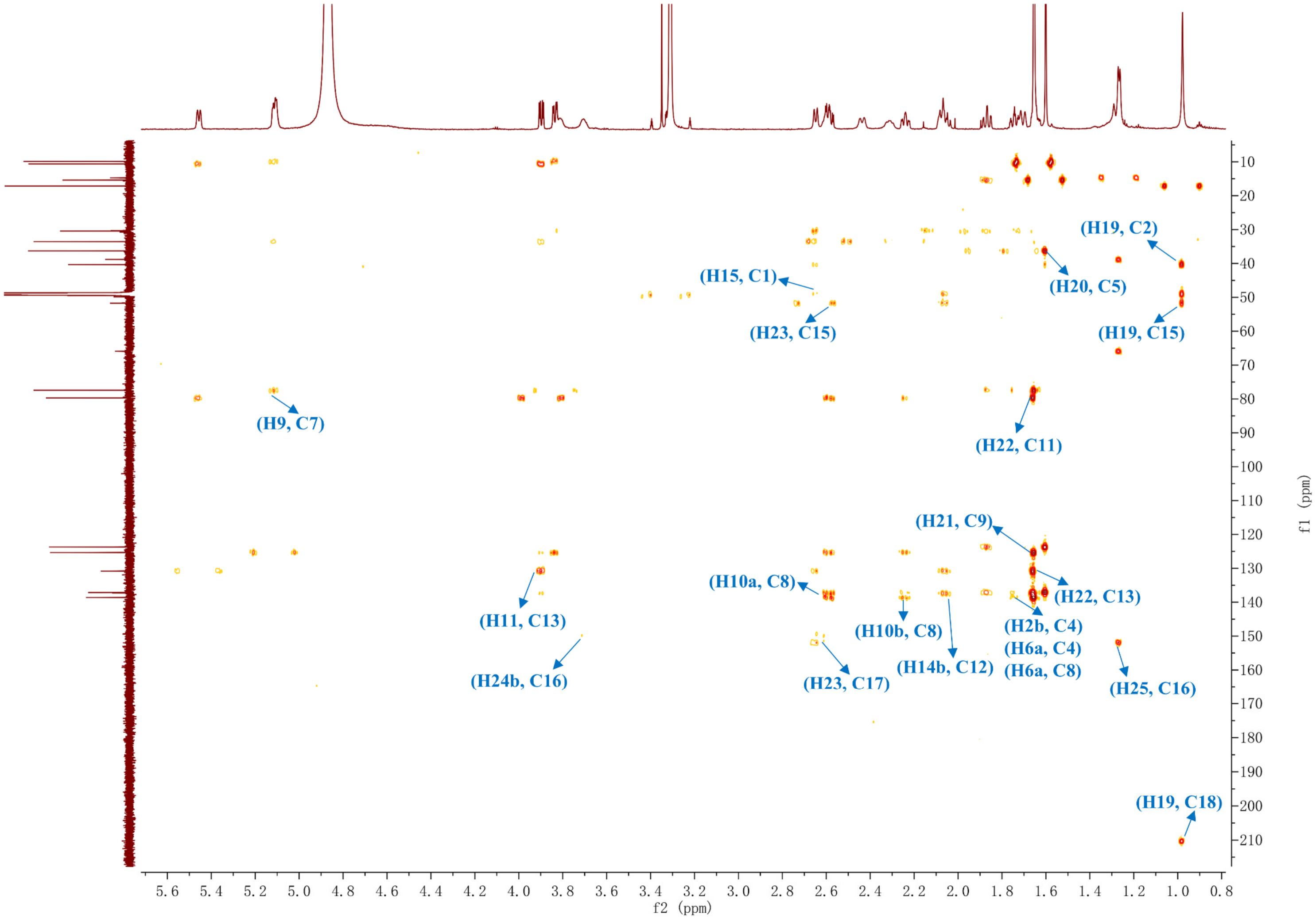

Figure S99. HMBC spectrum of arthritacin H (**8**) (Methanol-d_4) and the selected correlations (blue arrows and texts).

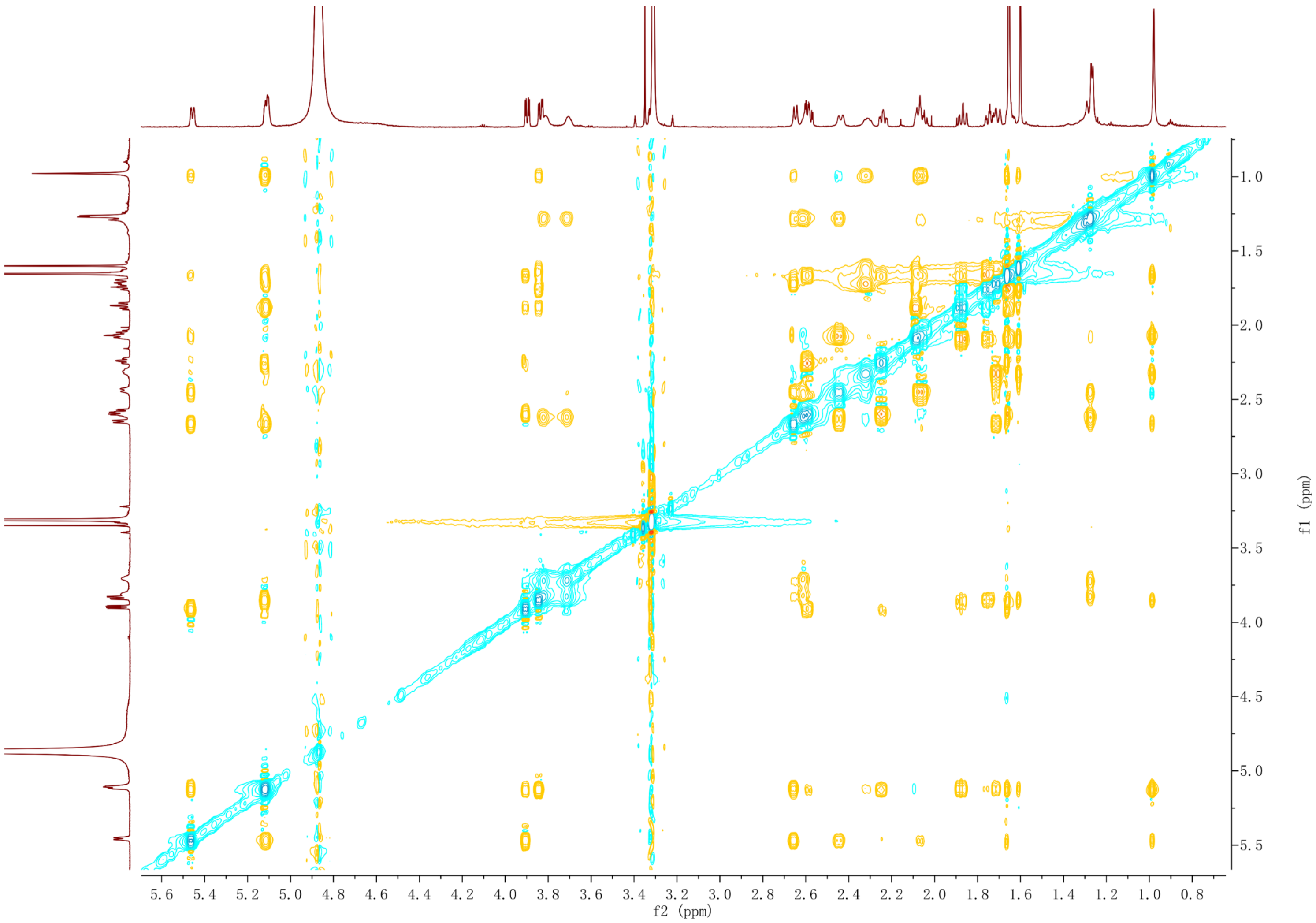

Figure S100. ROESY spectrum of arthritacin H (**8**) (Methanol-d_4) and the selected correlations (blue arrows and texts).

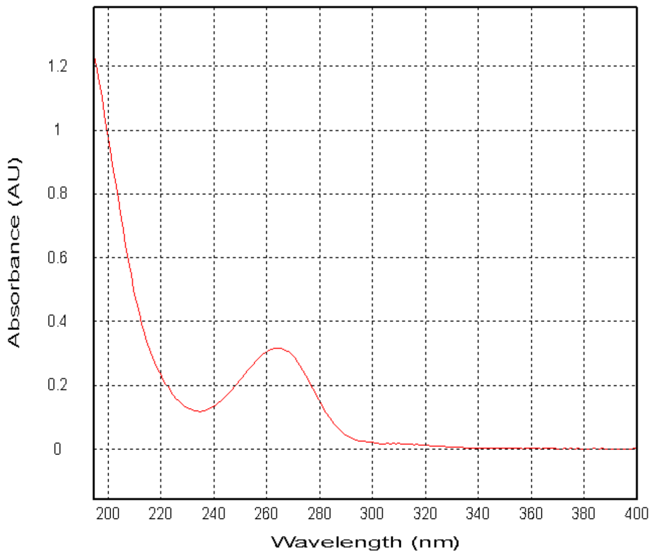

Figure S101. UV spectrum of arthritacin H (**8**).

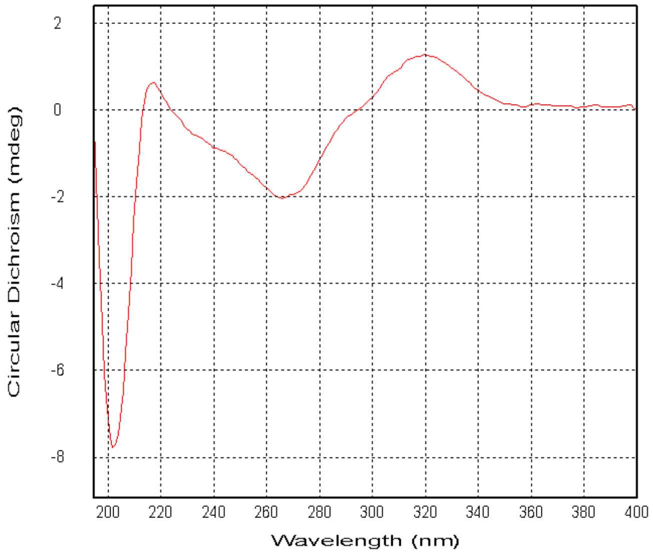

Figure S102. ECD spectrum of arthritacin H (**8**).

Rudolph Research Analytical

This sample was measured on an Autopol VI, Serial #91058
Manufactured by Rudolph Research Analytical, Hackettstown, NJ, USA.

Measurement Date : Friday, 28-MAY-2021

Set Temperature : OFF

Time Delay : Disabled

Delay between Measurement : Disabled

n	Average	Std.Dev.	% RSD	Maximum	Minimum
5	57.27	0.48	0.83	57.67	56.67

S.No	Sample ID	Time	Result	Scale	OR °Arc	WLG.nm	Lg.mm	Conc.g/100ml	Temp.
1	SHSJ37A	09:50:30 AM	57.67	SR	0.0346	589	100.00	0.060	24.2
2	SHSJ37A	09:50:38 AM	56.67	SR	0.0340	589	100.00	0.060	24.2
3	SHSJ37A	09:50:46 AM	56.83	SR	0.0341	589	100.00	0.060	24.2
4	SHSJ37A	09:50:54 AM	57.50	SR	0.0345	589	100.00	0.060	24.2
5	SHSJ37A	09:51:03 AM	57.67	SR	0.0346	589	100.00	0.060	24.2

Figure S103. ORD report of arthritacin H (**8**).

User Spectra

Fragmentor Voltage	Collision Energy	Ionization Mode
85	0	ESI

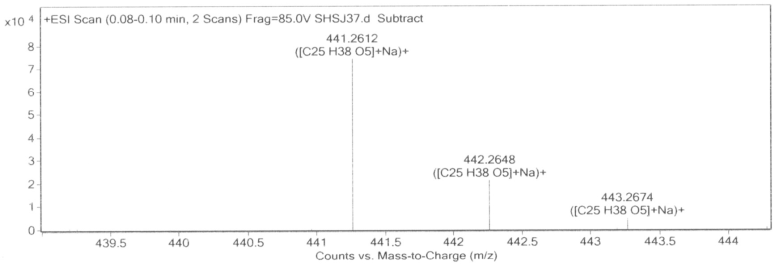

Peak List

m/z	z	Abund	Formula	Ion
141.9585		15874.63		
274.2741	1	16706.26		
307.1176	1	18819.95		
318.3001	1	14648.1		
329.0993	1	21700.68		
381.2972	1	18555.71		
401.2685	1	20959.73		
419.2786	1	25109.04		
441.2612	1	74286.7	C25 H38 O5	(M+Na)+
442.2648	1	21153.03	C25 H38 O5	(M+Na)+

Formula Calculator Element Limits

Element	Min	Max
C	3	60
H	0	120
O	0	30

Formula Calculator Results

Formula	CalculatedMass	CalculatedMz	Mz	Diff. (mDa)	Diff. (ppm)	DBE
C25 H38 O5	418.2719	441.2611	441.2612	-0.10	-0.23	7.0000

--- End Of Report ---

Figure S104. HRESIMS spectrum of arthritacin H (**8**).

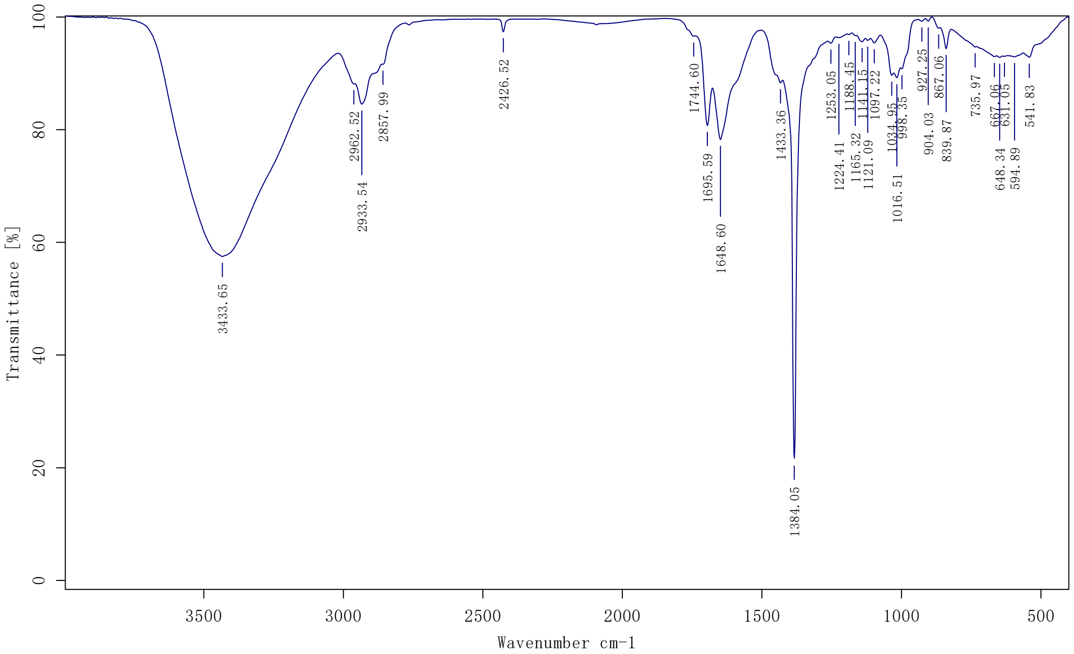

Sample Name: SHSJ37	Resolution: 4	Beamsplitter Setting: KBr
Sample Form: KBr	Aperture Setting: 6 mm	Source Setting: MIR
Path of File: E:\data	Number of Background Scans: 16	Instrument Type: BRUKER VERTEX 70
Date of Measurement: 2025/7/3	Number of Sample Scans: 16	Soft Version: OPUS8.1

Figure S105. IR spectrum of arthritacin H (**8**).

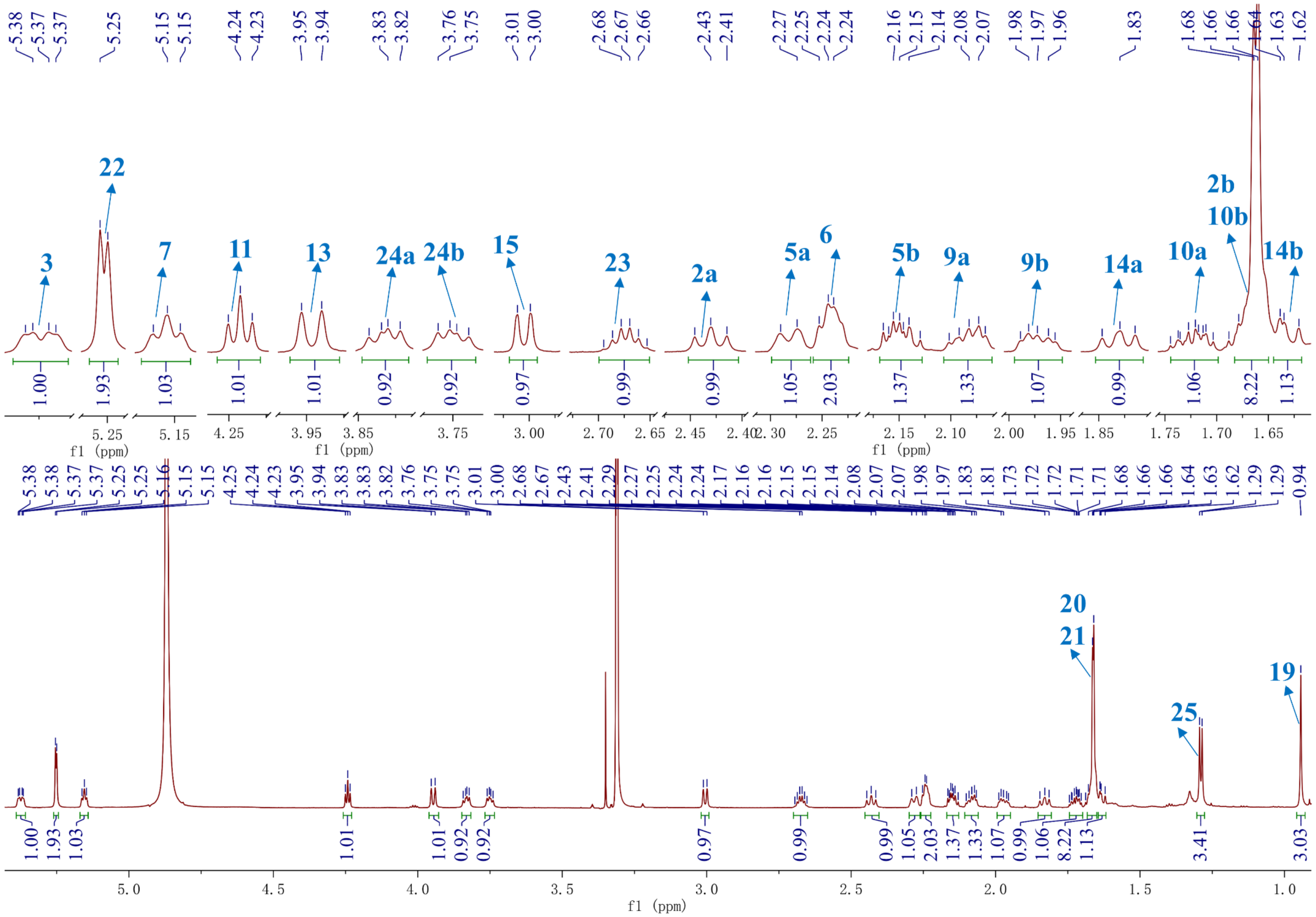

Figure S106. ^{1}H NMR spectrum of arthritacin I (**9**) (Methanol-d_4).

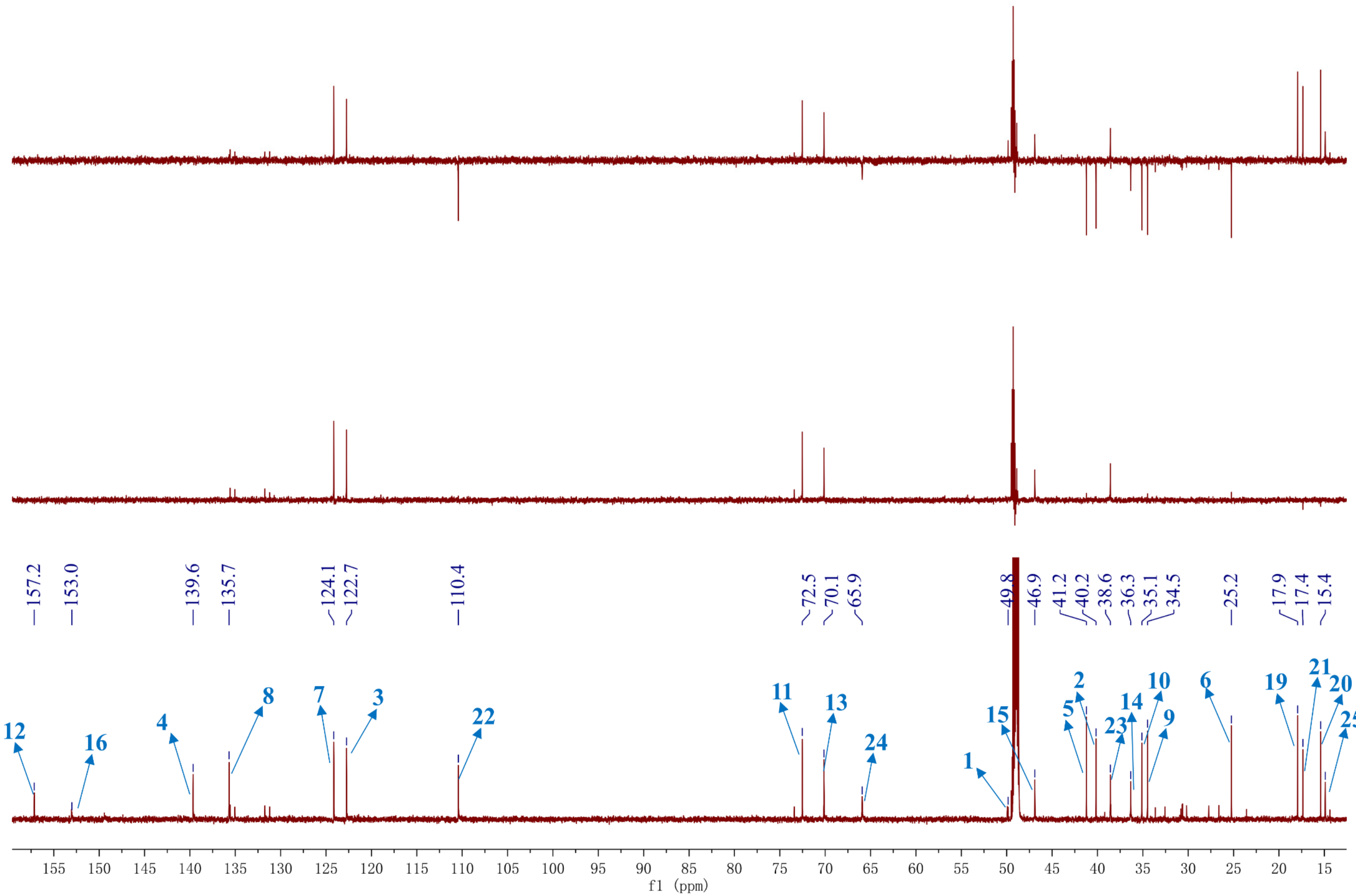

Figure S107. ^{13}C NMR and DEPT spectrum of arthritacin I (**9**) (Methanol-d_4).

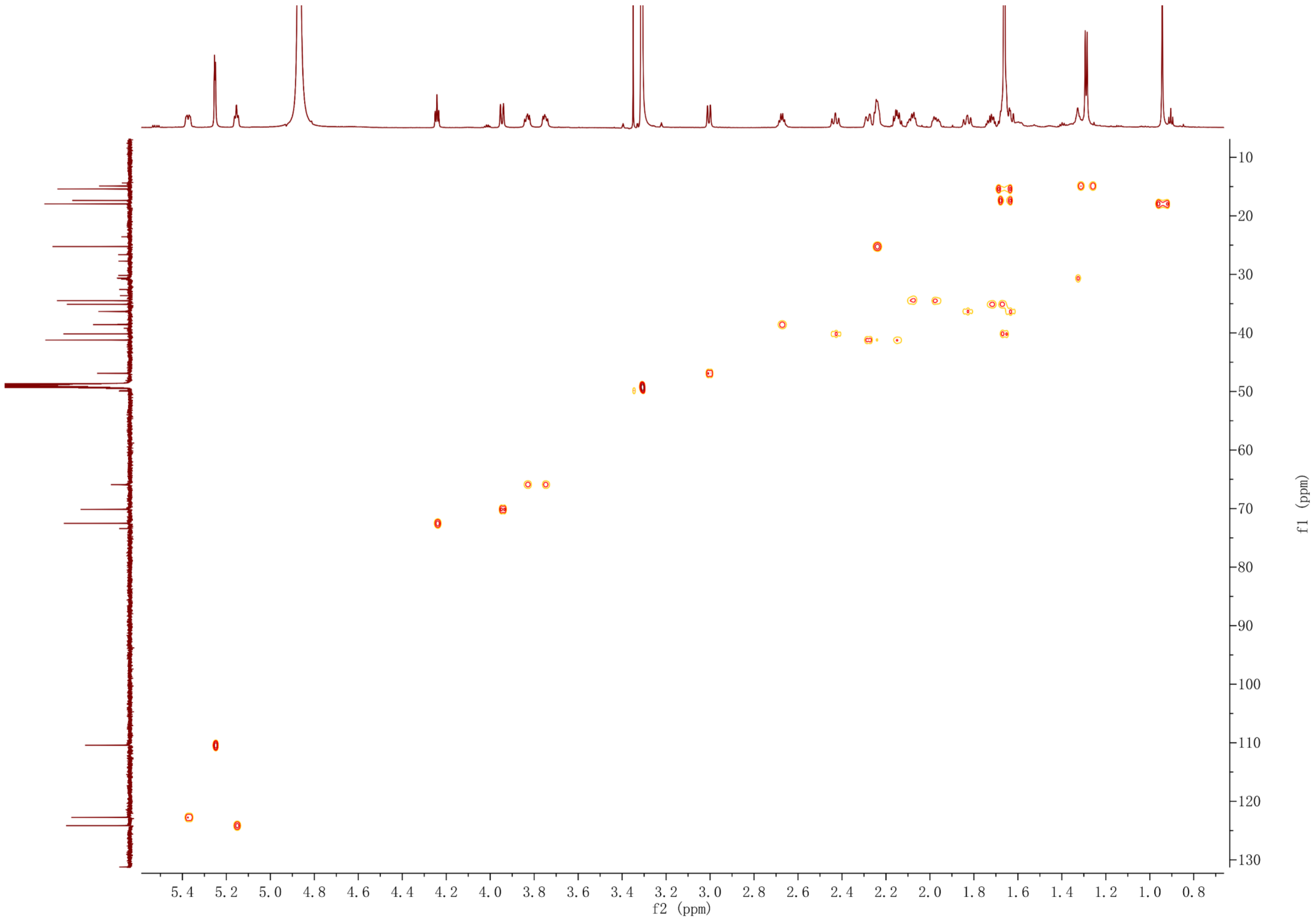

Figure S108. HSQC spectrum of arthritacin I (**9**) (Methanol-d_4).

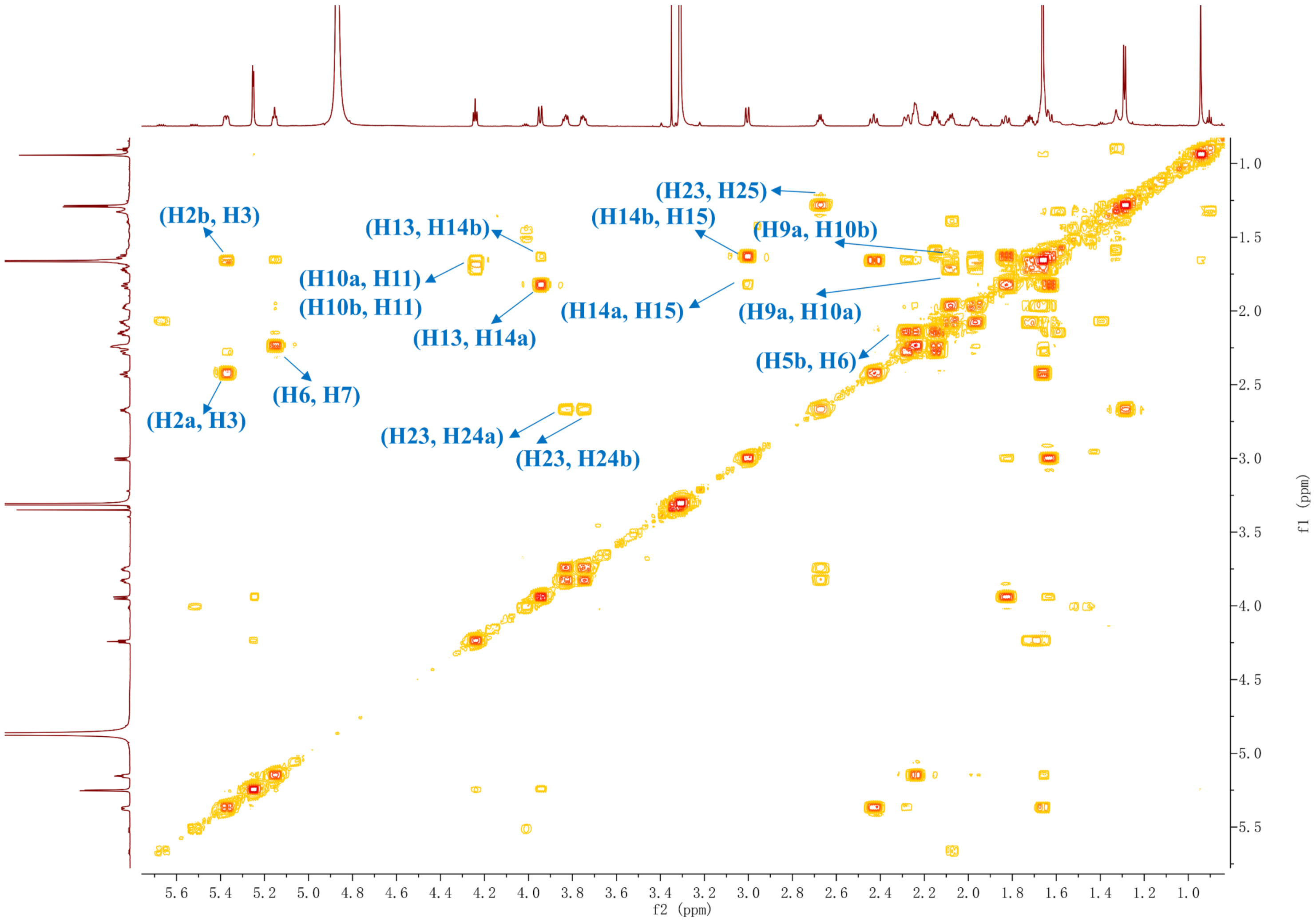

Figure S109. ^{1}H-^{1}H COSY spectrum of arthritacin I (**9**) (Methanol-d_4) and the selected cross-peaks (blue arrows and texts).

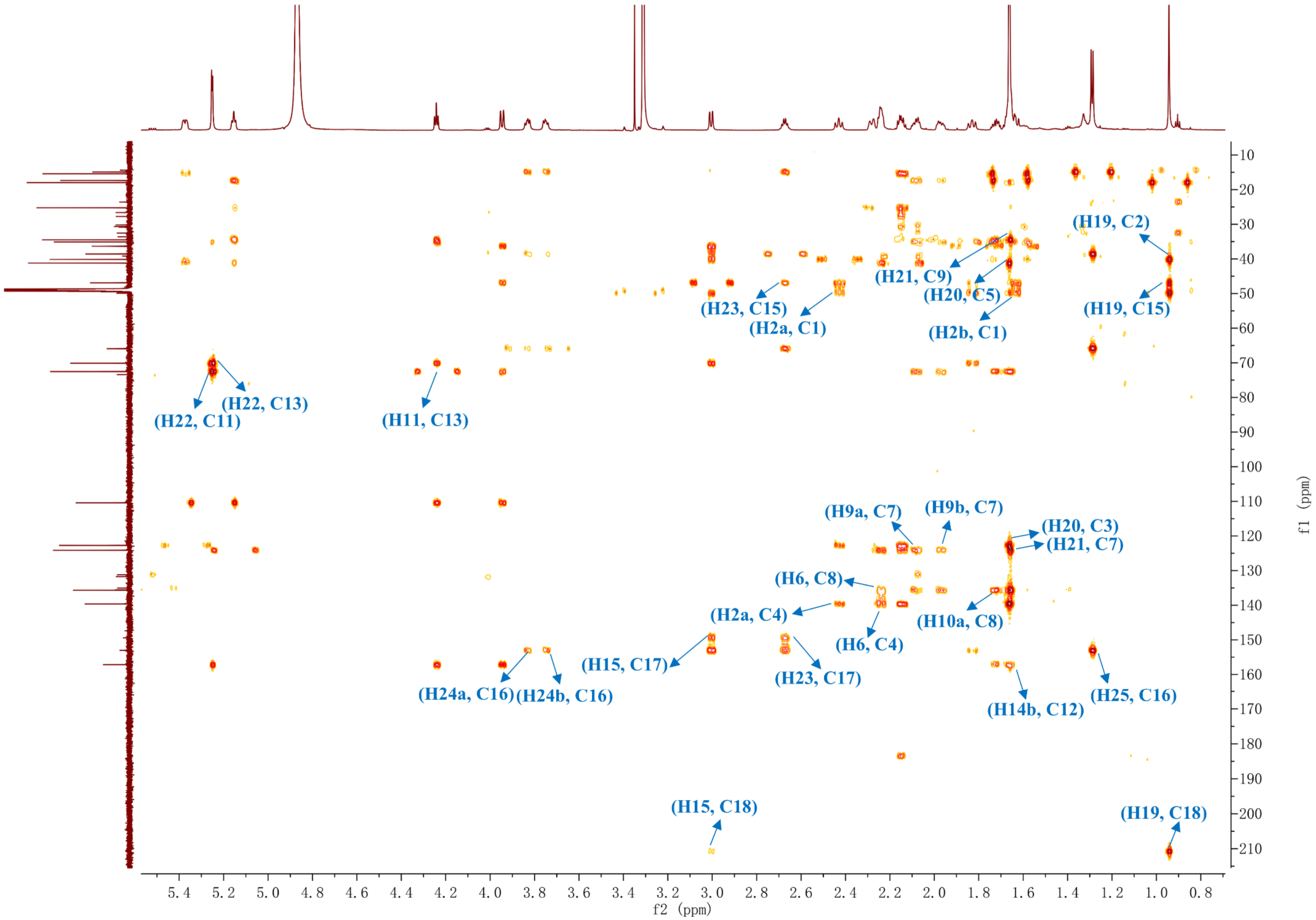

Figure S110. HMBC spectrum of arthritacin I (**9**) (Methanol-d_4) and the selected correlations (blue arrows and texts).

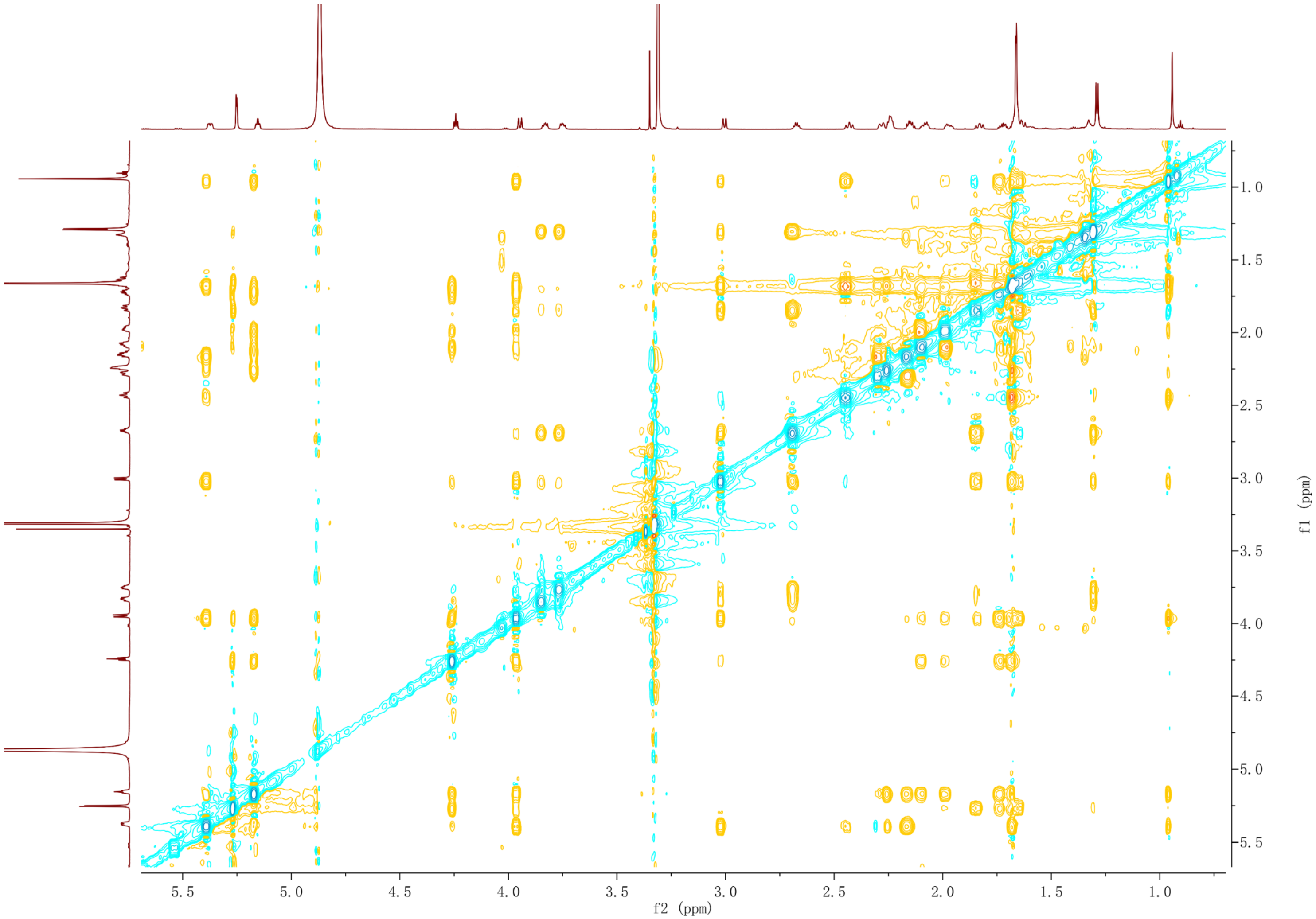

Figure S111. ROESY spectrum of arthritacin I (**9**) (Methanol-d_4) and the selected correlations (blue arrows and texts).

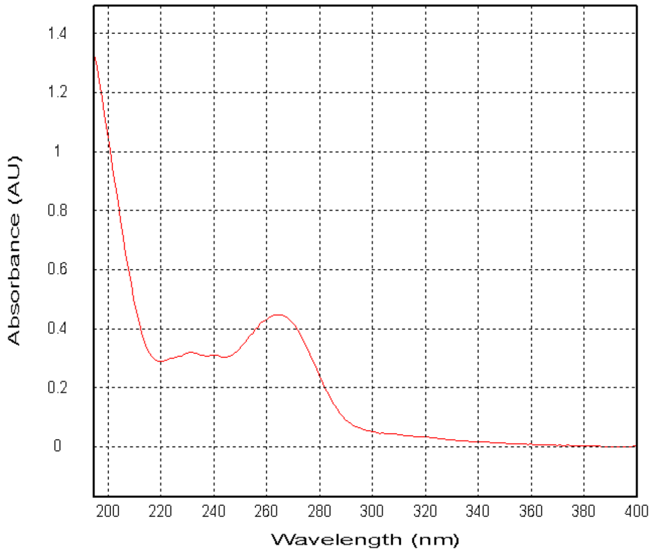

Figure S112. UV spectrum of arthritacin I (**9**).

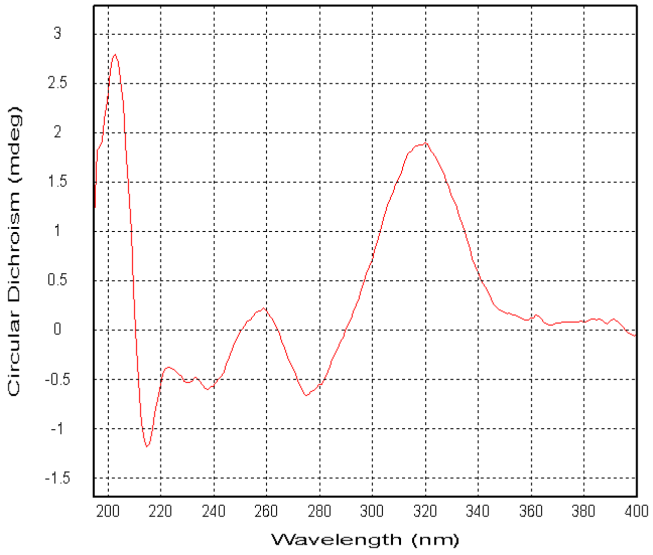

Figure S113. ECD spectrum of arthritacin I (**9**).

Rudolph Research Analytical

This sample was measured on an Autopol VI, Serial #91058
Manufactured by Rudolph Research Analytical, Hackettstown, NJ, USA.

Measurement Date : Friday, 28-MAY-2021

Set Temperature : OFF

Time Delay : Disabled

Delay between Measurement : Disabled

n	Average	Std.Dev.	% RSD	Maximum	Minimum
5	90.74	0.52	0.57	91.43	90.29

S.No	Sample ID	Time	Result	Scale	OR °Arc	WLG.nm	Lg.mm	Conc.g/100ml	Temp.
1	SHSJ32	11:03:24 AM	90.29	SR	0.0632	589	100.00	0.070	25.6
2	SHSJ32	11:03:33 AM	90.29	SR	0.0632	589	100.00	0.070	25.6
3	SHSJ32	11:03:41 AM	91.14	SR	0.0638	589	100.00	0.070	25.6
4	SHSJ32	11:03:49 AM	90.57	SR	0.0634	589	100.00	0.070	25.6
5	SHSJ32	11:03:57 AM	91.43	SR	0.0640	589	100.00	0.070	25.6

Figure S114. ORD report of arthritacin I (**9**).

User Spectra

Fragmentor Voltage	Collision Energy	Ionization Mode
85	0	ESI

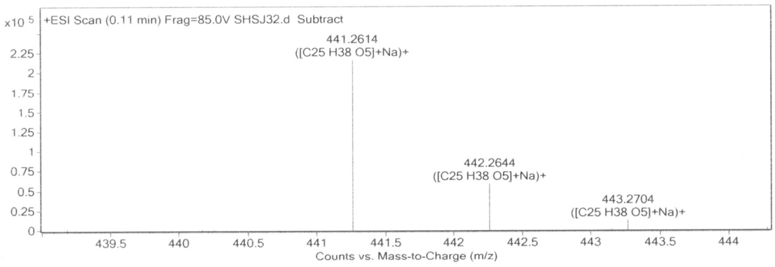

Peak List

m/z	z	Abund	Formula	Ion
102.1278	1	19902.18		
419.2791	1	66860.66		
420.2826	1	15081.68		
441.2614	1	216372.63	C25 H38 O5	(M+Na)+
442.2644	1	58581.28	C25 H38 O5	(M+Na)+
443.2704	1	12002.03	C25 H38 O5	(M+Na)+
457.237	1	21124.11		
859.5322	1	77402.36		
860.5356	1	41553.68		
861.5401	1	17929.89		

Formula Calculator Element Limits

Element	Min	Max
C	3	60
H	0	120
O	0	30

Formula Calculator Results

Formula	CalculatedMass	CalculatedMz	Mz	Diff. (mDa)	Diff. (ppm)	DBE
C25 H38 O5	418.2719	441.2611	441.2614	-0.30	-0.68	7.0000

--- End Of Report ---

Figure S115. HRESIMS spectrum of arthritacin I (**9**).

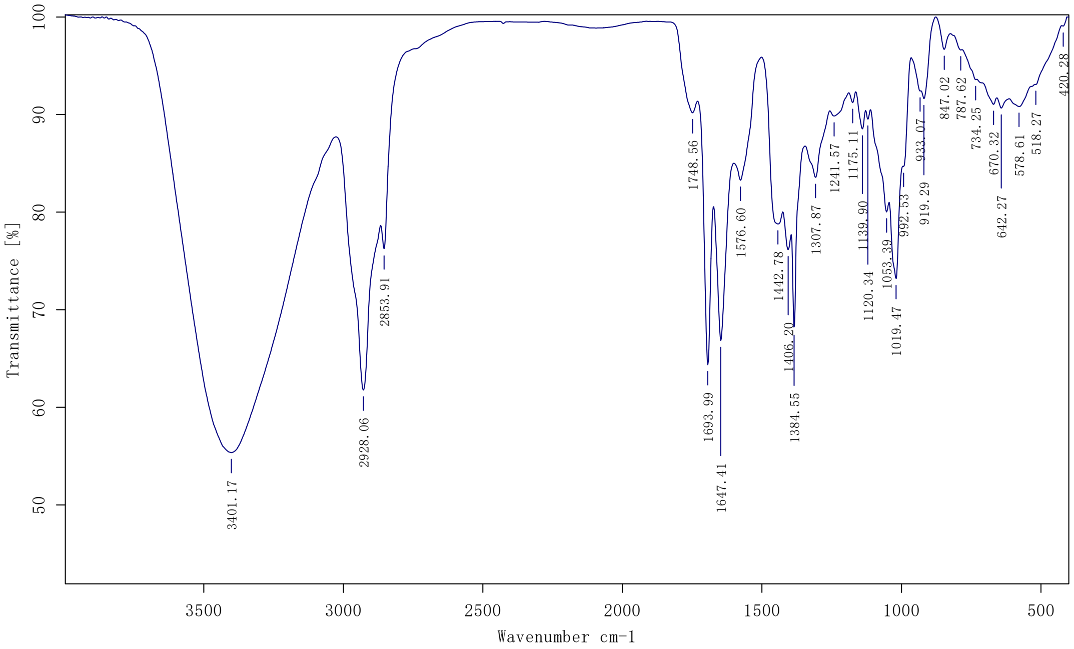

Sample Name: SHSJ32
Sample Form: KBr
Path of File: E:\data
Date of Measurement: 2025/7/3

Resolution: 4
Aperture Setting: 6 mm
Number of Background Scans: 16
Number of Sample Scans: 16

Beamsplitter Setting: KBr
Source Setting: MIR
Instrument Type: BRUKER VERTEX 70
Soft Version: OPUS8.1

Figure S116. IR spectrum of arthritacin I (**9**).

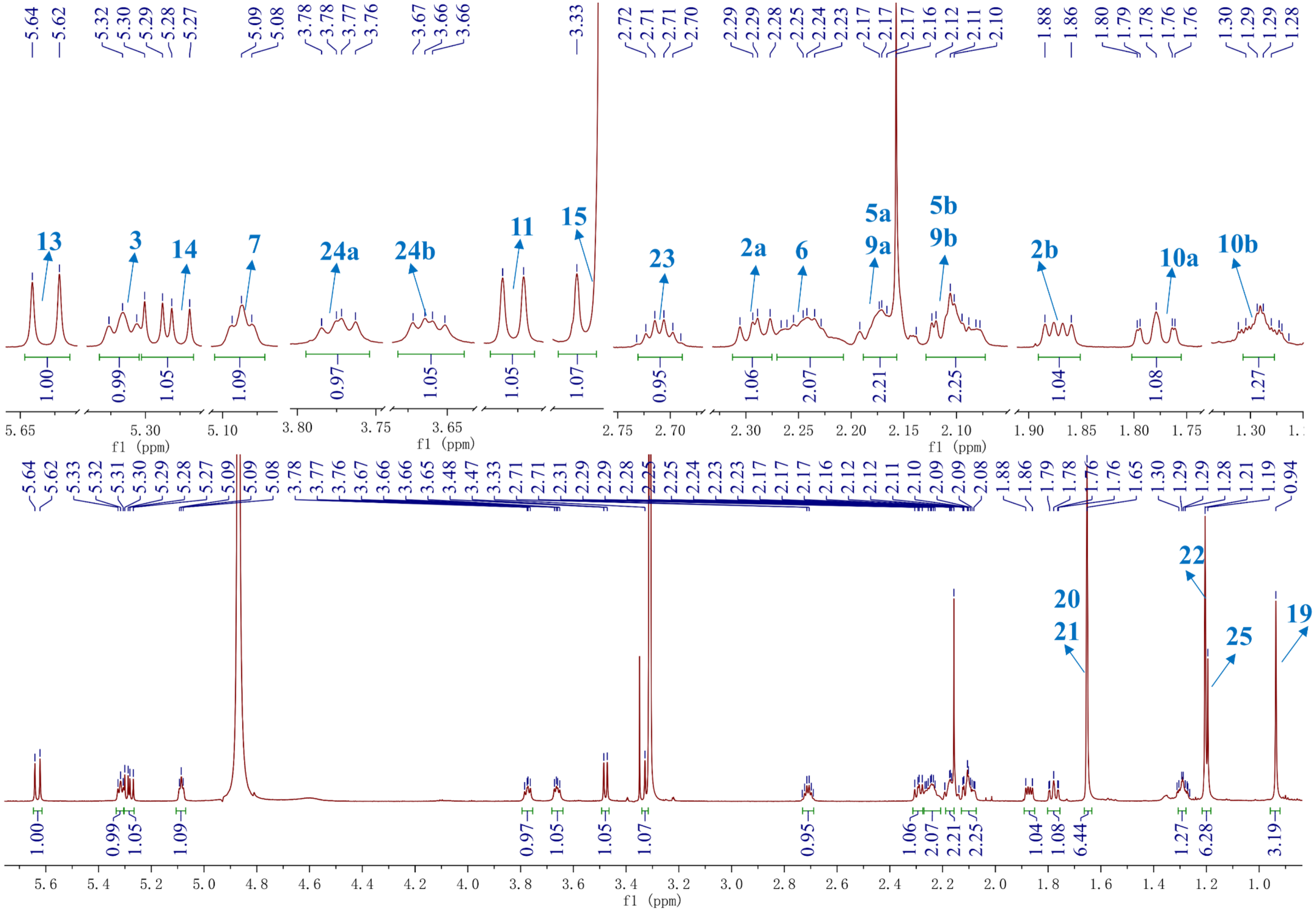

Figure S117. ^{1}H NMR spectrum of arthritacin J (**10**) (Methanol-d_4).

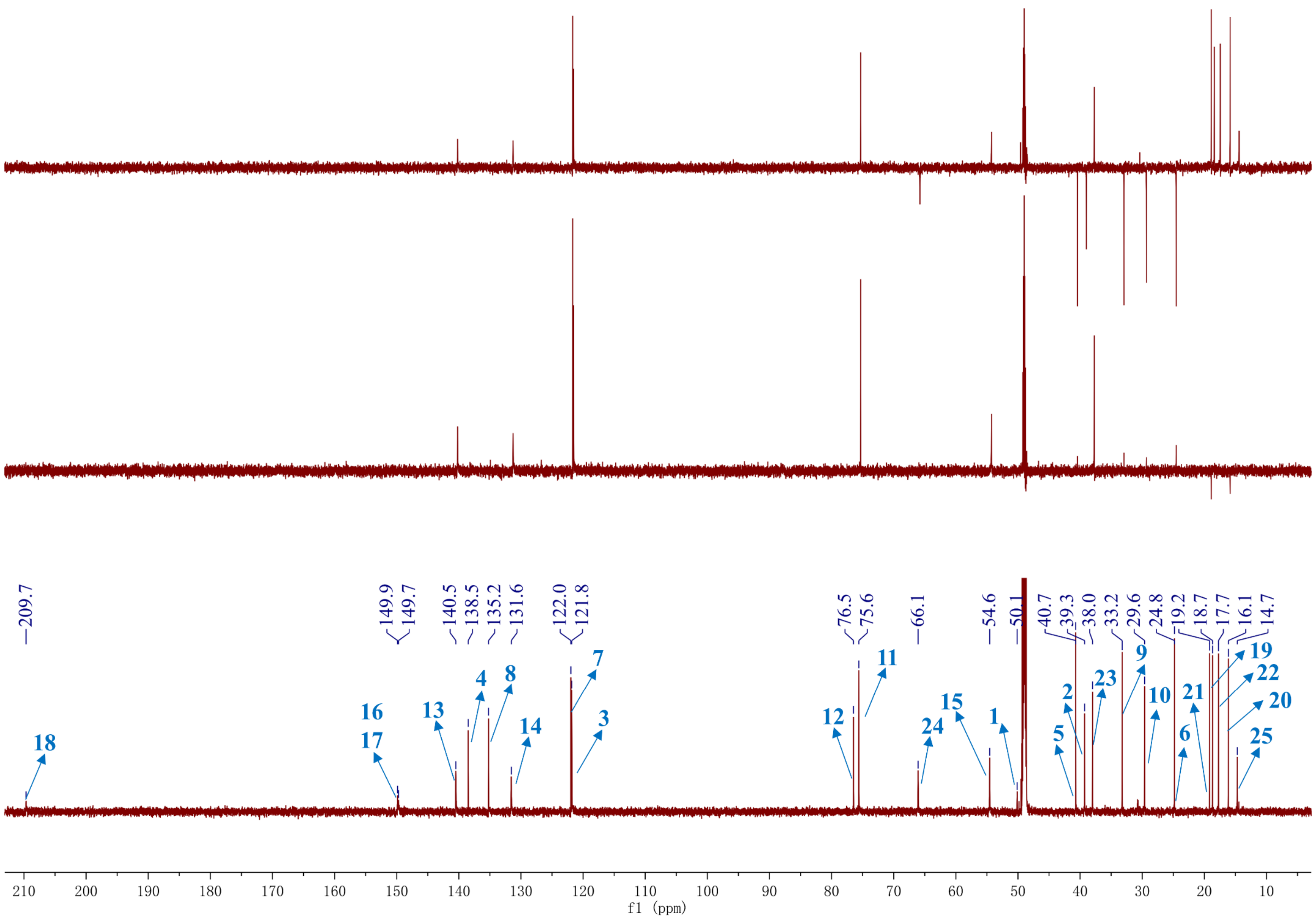

Figure S118. ^{13}C NMR and DEPT spectrum of arthritacin J (**10**) (Methanol-d_4).

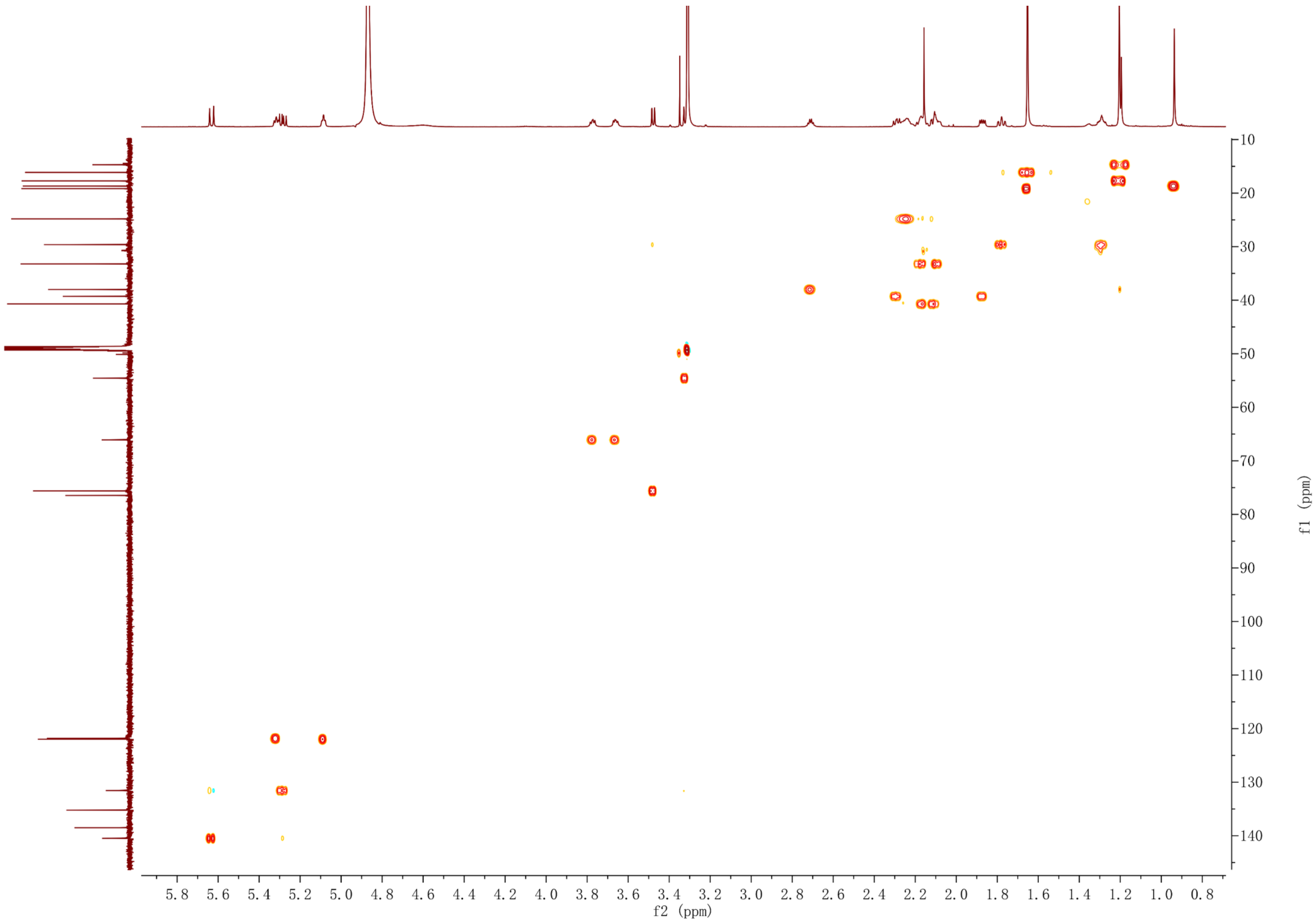

Figure S119. HSQC spectrum of arthritacin J (**10**) (Methanol-d_4).

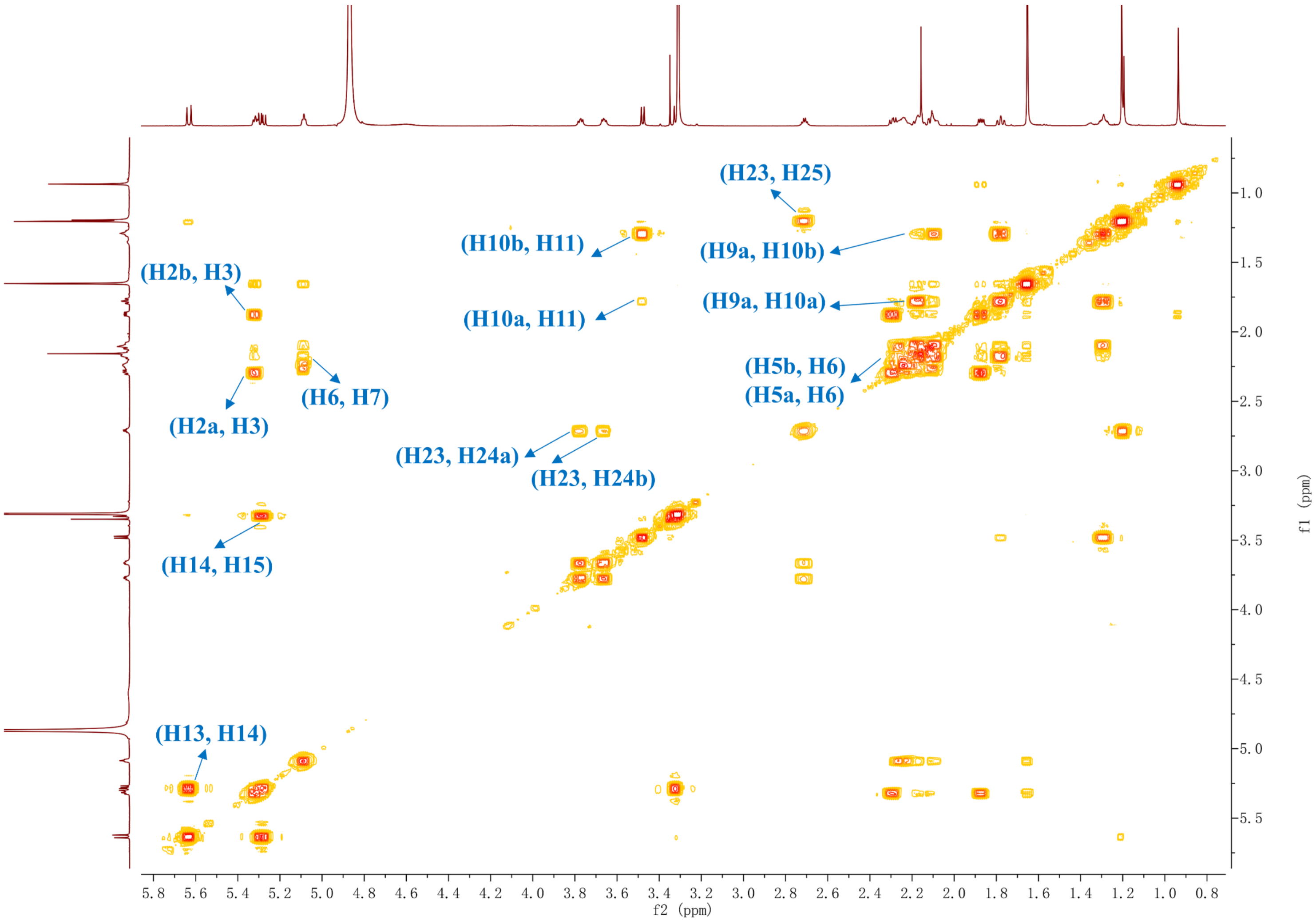

Figure S120. ^{1}H-^{1}H COSY spectrum of arthritacin J (**10**) (Methanol-d_4) and the selected cross-peaks (blue arrows and texts).

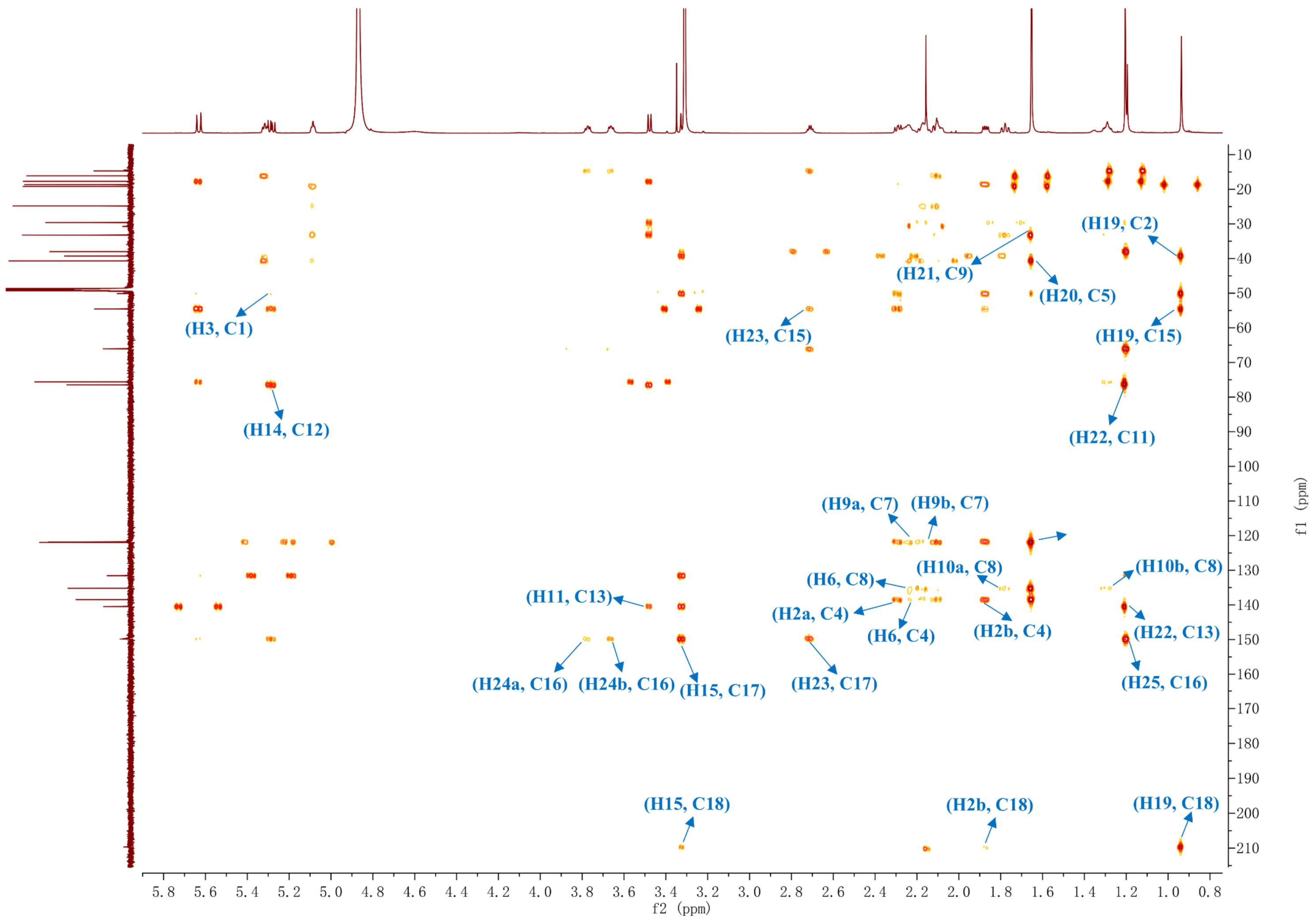

Figure S121. HMBC spectrum of arthritacin J (**10**) (Methanol-d_4) and the selected correlations (blue arrows and texts).

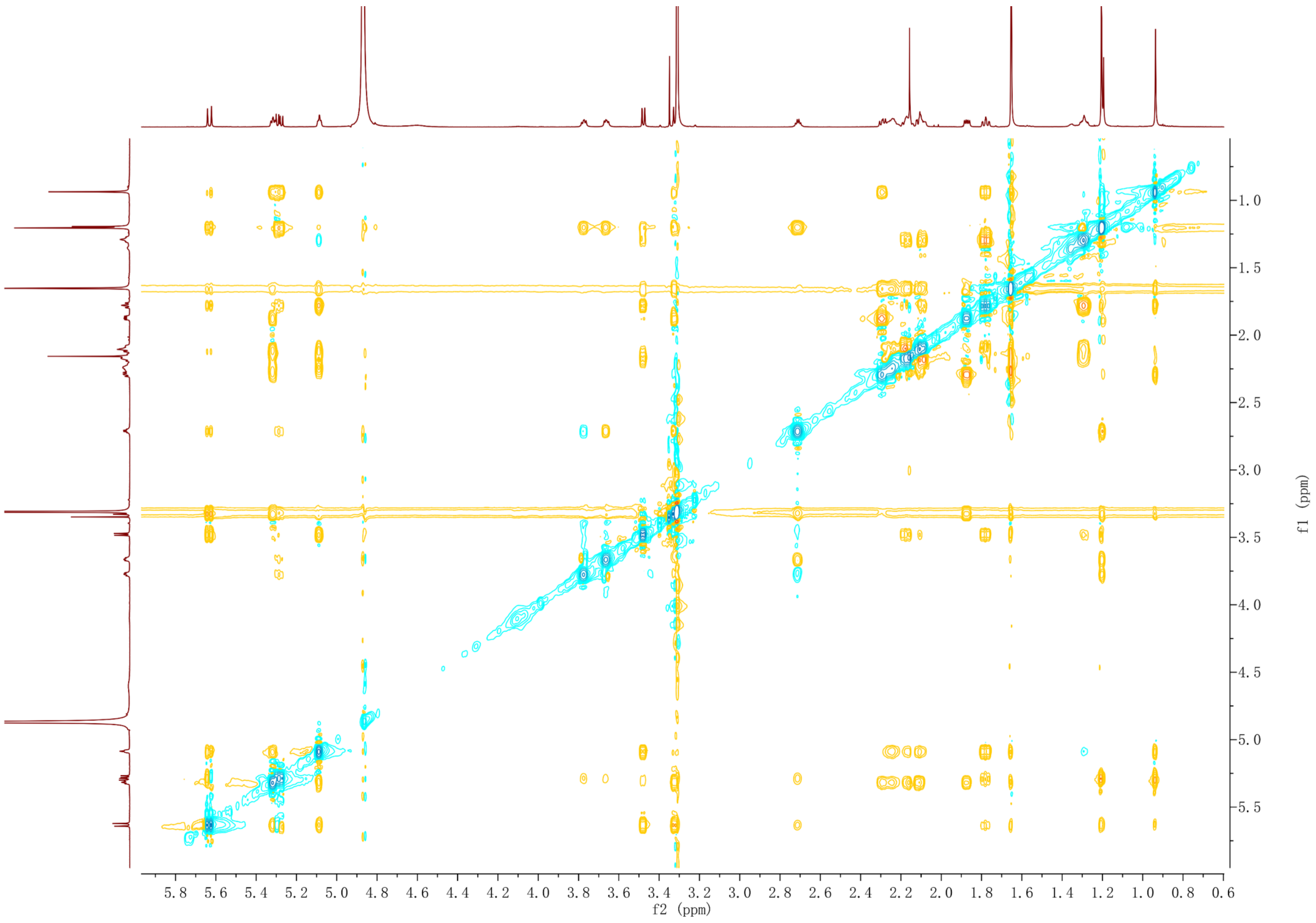

Figure S122. ROESY spectrum of arthritacin J (**10**) (Methanol-d_4) and the selected correlations (blue arrows and texts).

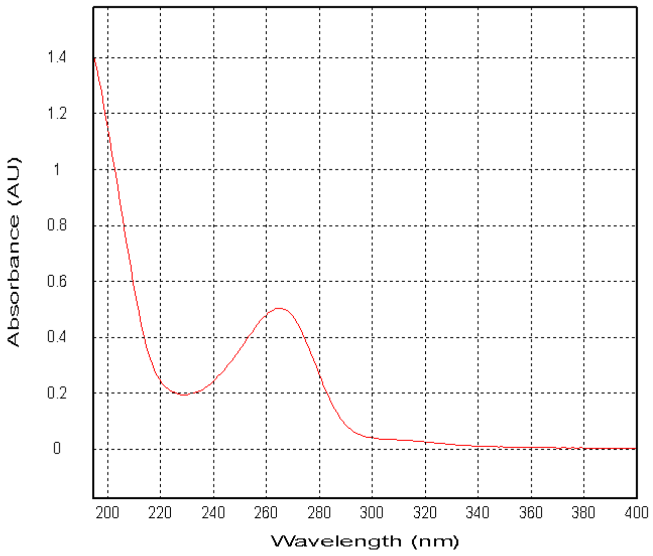

Figure S123. UV spectrum of arthritacin J (**10**).

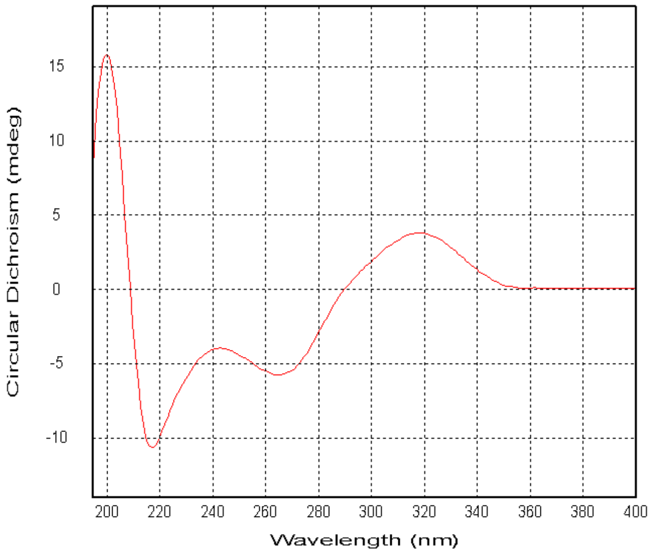

Figure S124. ECD spectrum of arthritacin J (**10**).

Rudolph Research Analytical

This sample was measured on an Autopol VI, Serial #91058
Manufactured by Rudolph Research Analytical, Hackettstown, NJ, USA.

Measurement Date : Friday, 28-MAY-2021

Set Temperature : OFF

Time Delay : Disabled

Delay between Measurement : Disabled

n	Average	Std.Dev.	% RSD	Maximum	Minimum
5	-6.36	0.35	-5.50	-5.90	-6.80

S.No	Sample ID	Time	Result	Scale	OR °Arc	WLG.nm	Lg.mm	Conc.g/100ml	Temp.
1	SHSJ33A	10:09:39 AM	-5.90	SR	-0.0059	589	100.00	0.100	24.7
2	SHSJ33A	10:09:48 AM	-6.20	SR	-0.0062	589	100.00	0.100	24.7
3	SHSJ33A	10:09:56 AM	-6.80	SR	-0.0068	589	100.00	0.100	24.7
4	SHSJ33A	10:10:04 AM	-6.30	SR	-0.0063	589	100.00	0.100	24.7
5	SHSJ33A	10:10:12 AM	-6.60	SR	-0.0066	589	100.00	0.100	24.7

Figure S125. ORD report of arthritacin J (**10**).

User Spectra

Fragmentor Voltage	Collision Energy	Ionization Mode
135	0	ESI

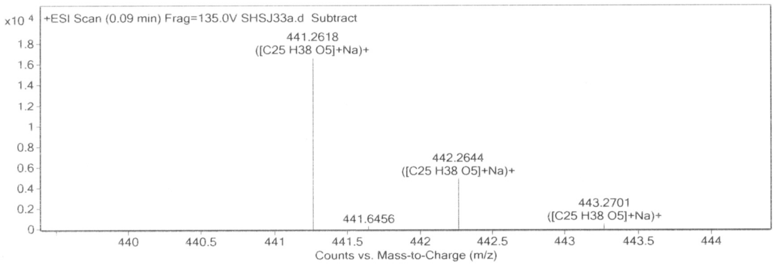

Peak List

m/z	z	Abund	Formula	Ion
59.0606		3452.65		
69.0448		2772.45		
274.2753	1	1789.01		
318.3009	1	1971.22		
383.2593	1	3898.78		
419.2796	1	6400.04		
441.2618	1	16628.61	C25 H38 O5	(M+Na)+
442.2644	1	4909.39	C25 H38 O5	(M+Na)+
457.2354	1	2585.36		
477.3335	1	1426.04		
654.3347	1	1323.92		
859.5359	1	1442.85		

Formula Calculator Element Limits

Element	Min	Max
C	3	60
H	0	120
O	0	30
N	0	5

Formula Calculator Results

Formula	CalculatedMass	CalculatedMz	Mz	Diff. (mDa)	Diff. (ppm)	DBE
C25 H38 O5	418.2719	441.2611	441.2618	-0.70	-1.59	7.0000

--- End Of Report ---

Figure S126. HRESIMS spectrum of arthritacin J (**10**).

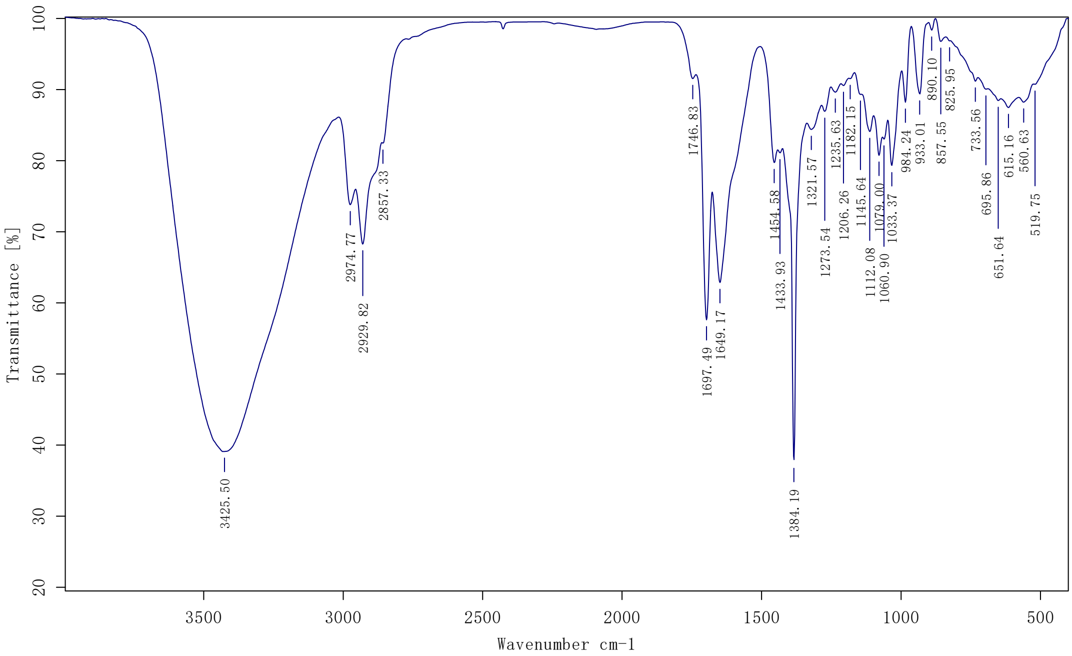

Sample Name: SHSJ33	Resolution: 4	Beamsplitter Setting: KBr
Sample Form: KBr	Aperture Setting: 6 mm	Source Setting: MIR
Path of File: E:\data	Number of Background Scans: 16	Instrument Type: BRUKER VERTEX 70
Date of Measurement: 2025/7/3	Number of Sample Scans: 16	Soft Version: OPUS8.1

Figure S127. IR spectrum of arthritacin J (**10**).

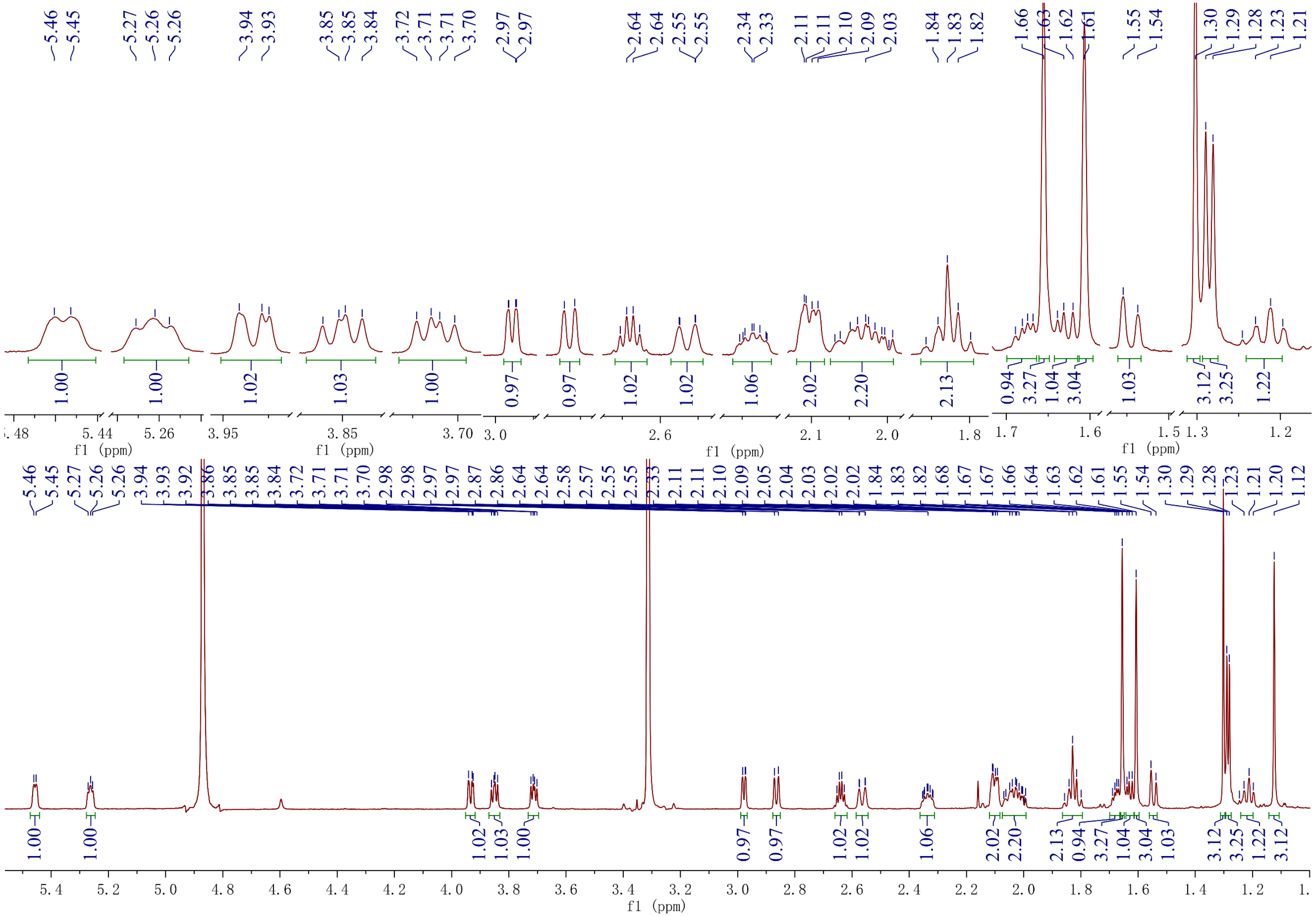

Figure S128. ^{1}H NMR spectrum of synthetic **1** (Methanol-d_4).

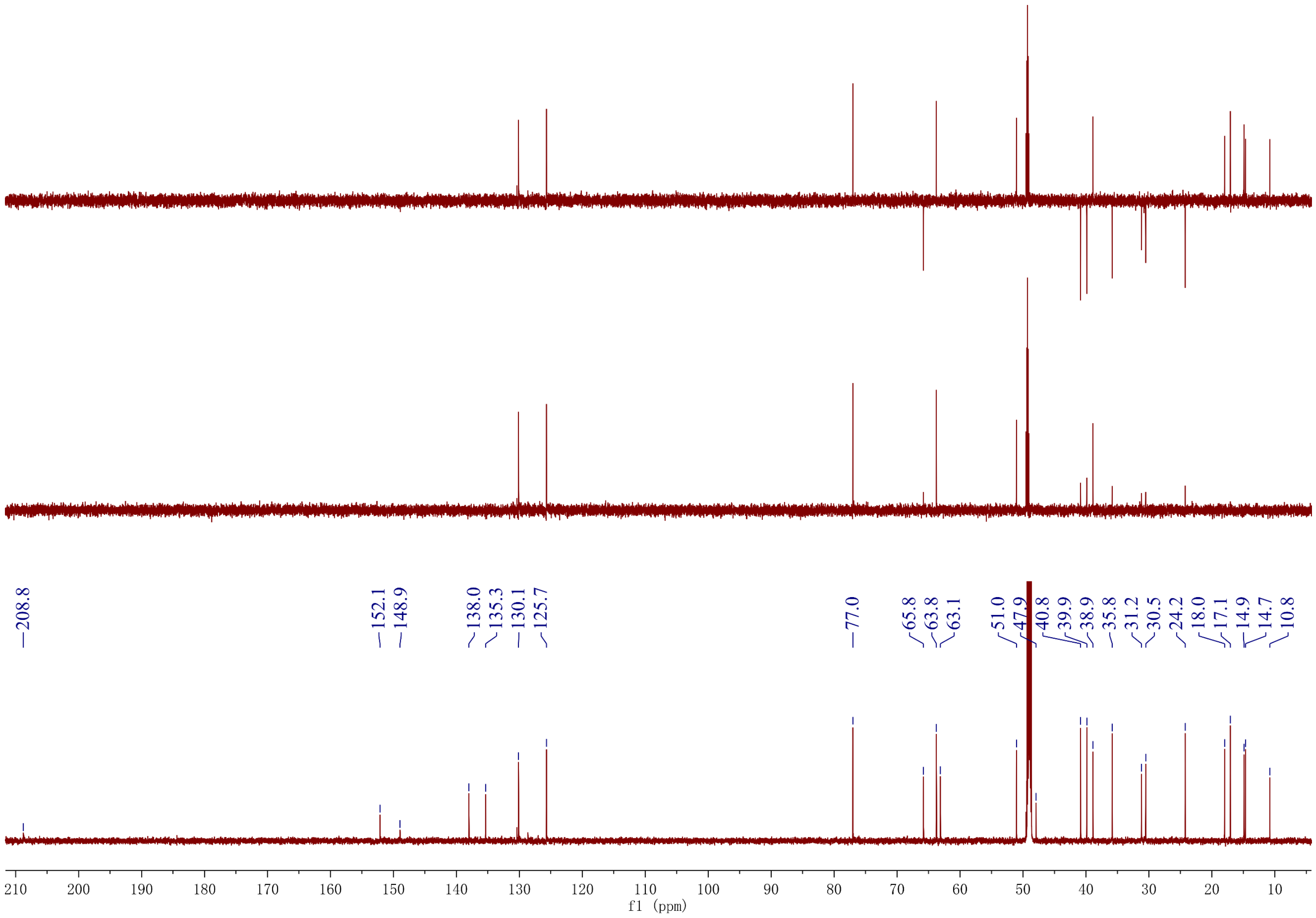

Figure S129. ^{13}C NMR and DEPT spectrum of synthetic **1** (Methanol-d_4).

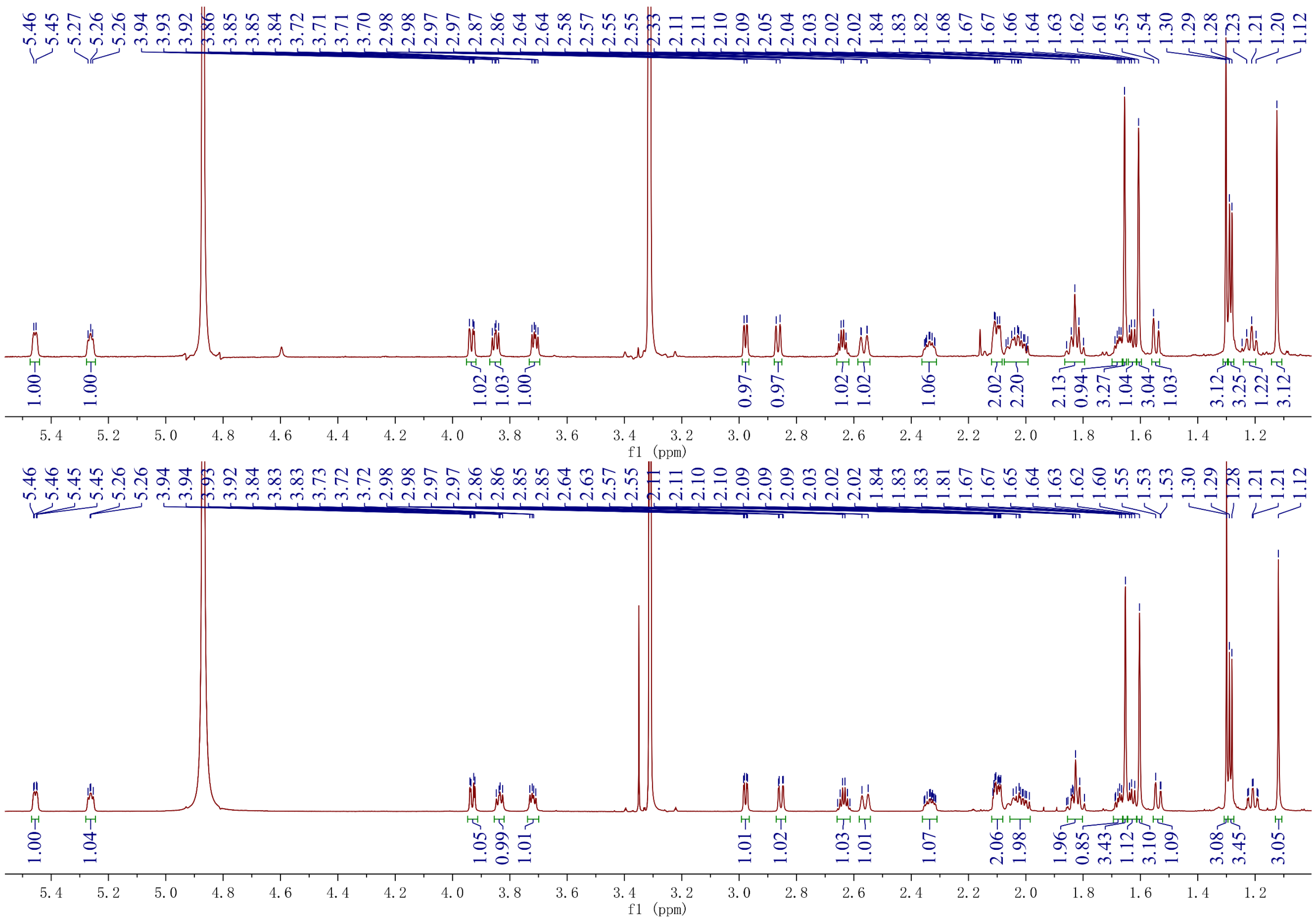

Figure S130. Comparison of ^{1}H NMR spectra of natural **1** (Methanol-d_4, upper) and synthetic **1** (Methanol-d_4, below).

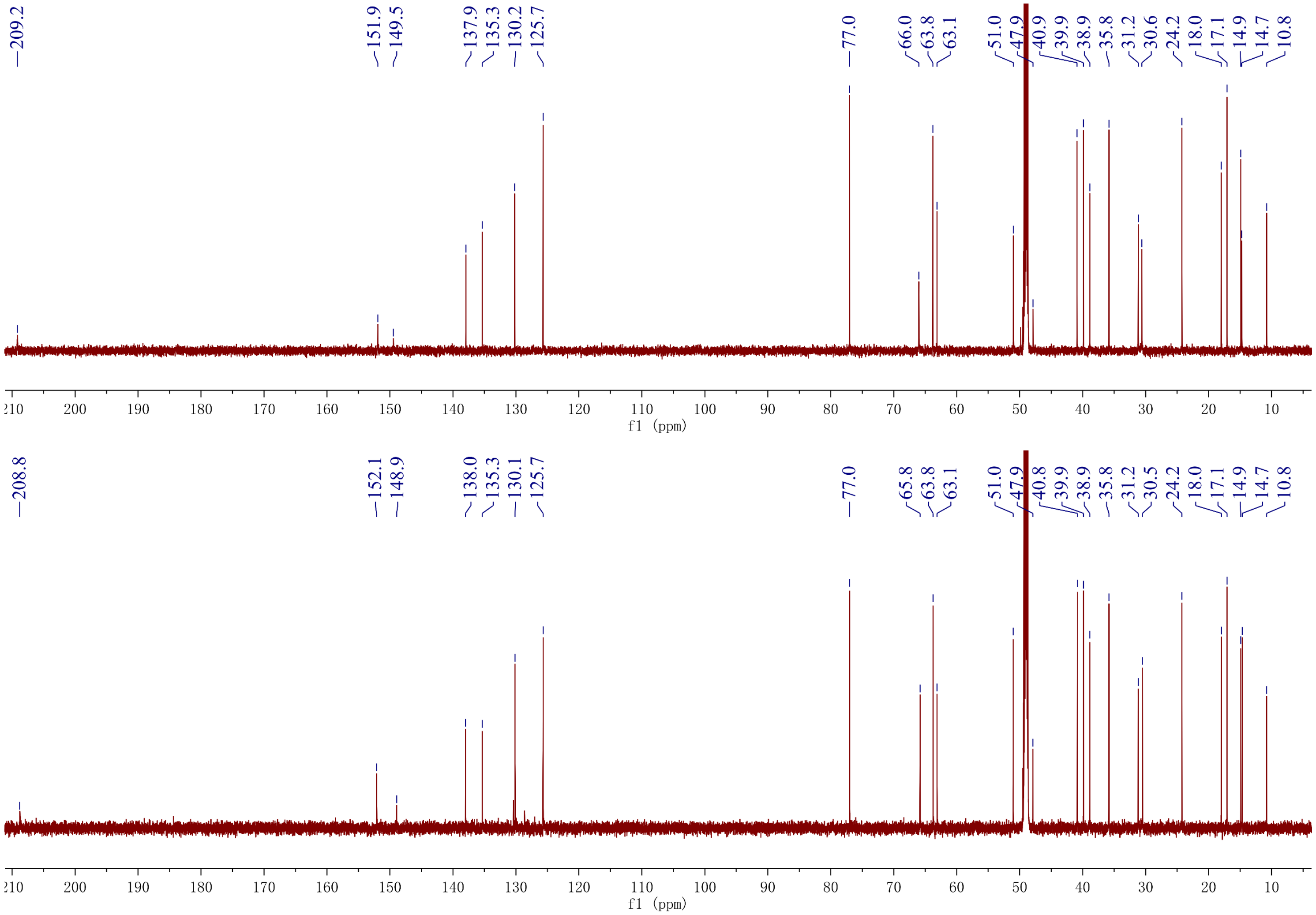

Figure S131. Comparison of ^{13}C NMR spectra of natural **1** (Methanol-d_4, upper) and synthetic **1** (Methanol-d_4, below).

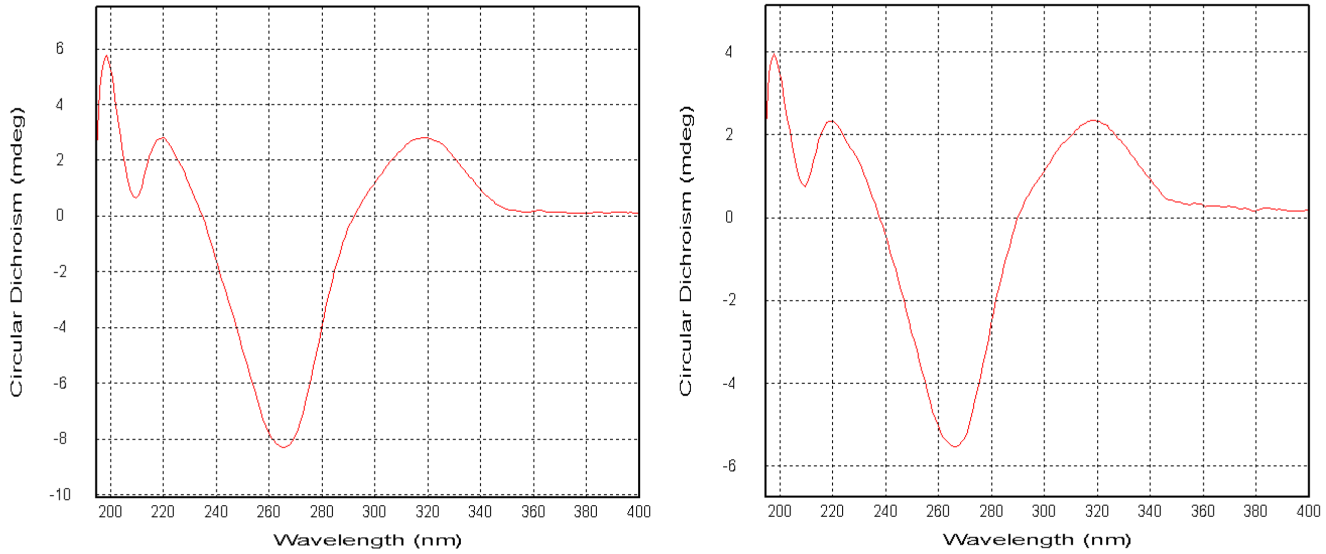

Figure S132. Comparison of ECD spectra of natural **1** (left) and synthetic **1** (right).

Rudolph Research Analytical

This sample was measured on an Autopol VI, Serial #91058
Manufactured by Rudolph Research Analytical, Hackettstown, NJ, USA.

Measurement Date : Friday, 28-MAY-2021

Set Temperature : OFF

Time Delay : Disabled

Delay between Measurement : Disabled

n	Average	Std.Dev.	% RSD	Maximum	Minimum
5	-19.89	0.28	-1.40	-19.64	-20.36

S.No	Sample ID	Time	Result	Scale	OR °Arc	WLG.nm	Lg.mm	Conc.g/100ml	Temp.
1	SHSJ29	10:49:14 AM	-19.82	SR	-0.0218	589	100.00	0.110	25.4
2	SHSJ29	10:49:23 AM	-19.64	SR	-0.0216	589	100.00	0.110	25.4
3	SHSJ29	10:49:31 AM	-19.73	SR	-0.0217	589	100.00	0.110	25.4
4	SHSJ29	10:49:39 AM	-19.91	SR	-0.0219	589	100.00	0.110	25.4
5	SHSJ29	10:49:47 AM	-20.36	SR	-0.0224	589	100.00	0.110	25.4

Rudolph Research Analytical

This sample was measured on an Autopol VI, Serial #91058
Manufactured by Rudolph Research Analytical, Hackettstown, NJ, USA.

Measurement Date : Tuesday, 02-JAN-2024

Set Temperature : 20.0

Time Delay : Disabled

Delay between Measurement : Disabled

n	Average	Std.Dev.	% RSD	Maximum	Minimum
5	-27.00	0.74	-2.74	-26.67	-28.33

S.No	Sample ID	Time	Result	Scale	OR °Arc	WLG.nm	Lg.mm	Conc.g/100ml	Temp.
1	SHSJR6	09:58:45 AM	-26.67	SR	-0.016	589	100.00	0.060	20.1
2	SHSJR6	09:58:51 AM	-26.67	SR	-0.016	589	100.00	0.060	20.1
3	SHSJR6	09:58:57 AM	-28.33	SR	-0.017	589	100.00	0.060	20.1
4	SHSJR6	09:59:04 AM	-26.67	SR	-0.016	589	100.00	0.060	20.0
5	SHSJR6	09:59:10 AM	-26.67	SR	-0.016	589	100.00	0.060	20.0

Figure S133. Comparison of ORD reports of natural **1** (upper) and synthetic **1** (below).

User Spectra

Fragmentor Voltage: 85 | Collision Energy: 0 | Ionization Mode: ESI

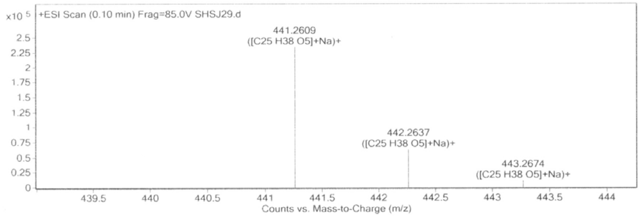

Peak List

m/z	z	Abund	Formula	Ion
64.0163	1	50962.85		
101.0036	1	42315.71		
203.147	1	22077.94		
401.2684	1	48142.01		
441.2609	1	234358.31	C25 H38 O5	(M+Na)+
442.2637	1	62559.24	C25 H38 O5	(M+Na)+
457.2363	1	29682.72		
477.3308	1	20745.66		
859.5311	1	74616.79		
860.5338	1	40277.12		

Formula Calculator Element Limits

Element	Min	Max
C	3	60
H	0	120
O	0	30

Formula Calculator Results

Formula	CalculatedMass	CalculatedMz	Mz	Diff. (mDa)	Diff. (ppm)	DBE
C25 H38 O5	418.2719	441.2611	441.2609	0.20	0.45	7.0000

--- End Of Report ---

User Spectra

Fragmentor Voltage: 135 | Collision Energy: 0 | Ionization Mode: ESI

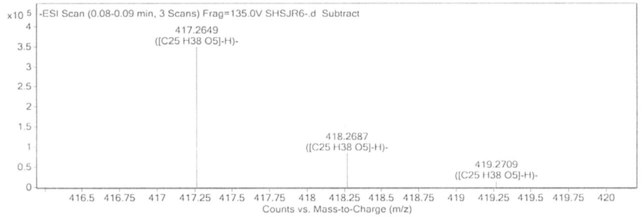

Peak List

m/z	z	Abund	Formula	Ion
112.9856	1	19135.64		
417.2649	1	349890.66	C25 H38 O5	(M-H)-
418.2687	1	83891.98	C25 H38 O5	(M-H)-
463.2702	1	57650.56		
480.2609	1	60373.5		
481.2649	1	17379.37		
485.2527	1	28478.36		
835.5367	1	106422.34		
836.5398	1	57021.38		
837.5423	1	17573.03		

Formula Calculator Element Limits

Element	Min	Max
C	3	60
H	0	150
O	0	20
N	0	5

Formula Calculator Results

Formula	CalculatedMass	CalculatedMz	Mz	Diff. (mDa)	Diff. (ppm)	DBE
C25 H38 O5	418.2719	417.2646	417.2649	-0.30	-0.72	7.0000

--- End Of Report ---

Figure S134. Comparison of HRESIMS spectra of natural **1** (left) and synthetic **1** (right).

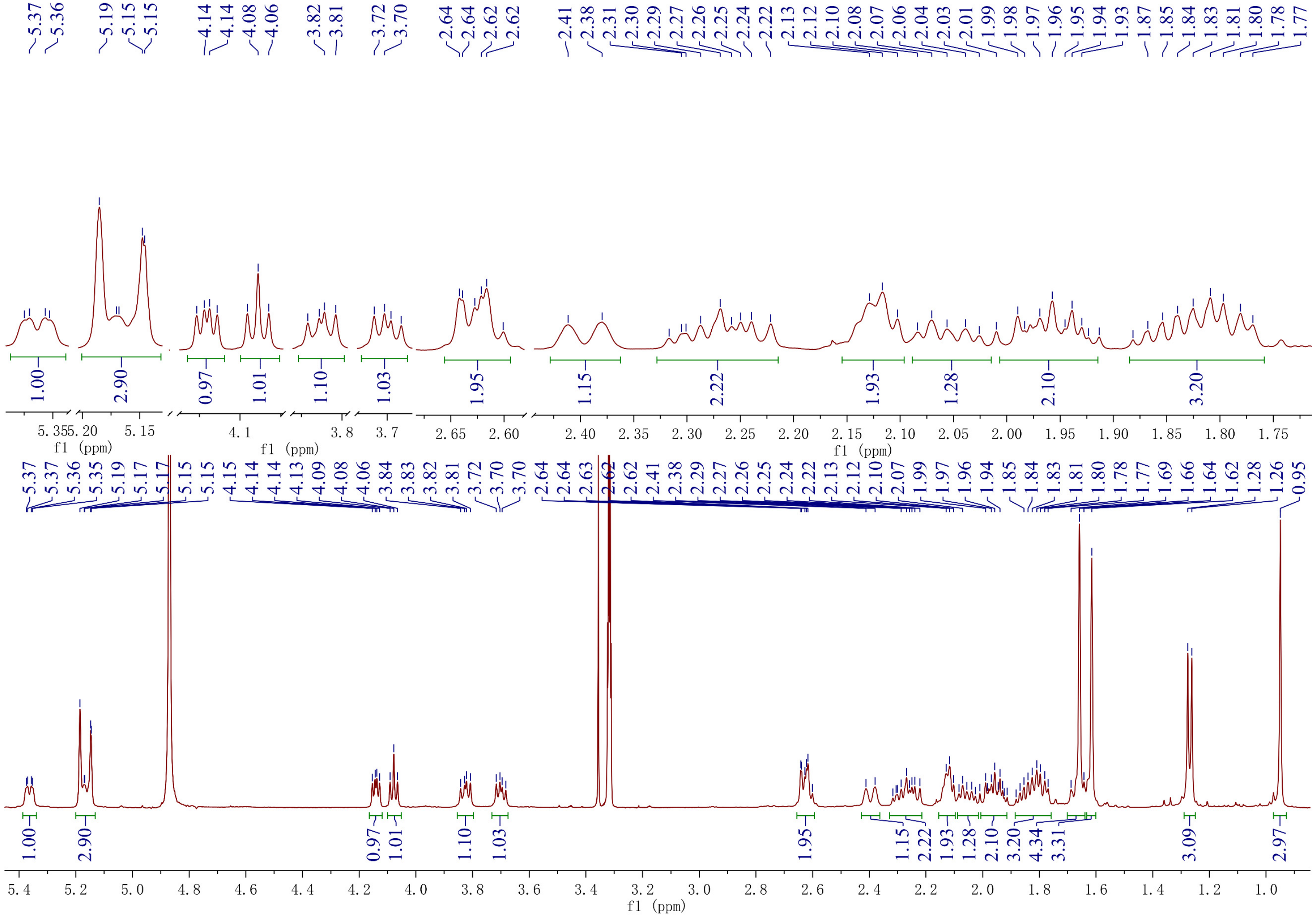

Figure S135. ^{1}H NMR spectrum of synthetic **2** (Methanol-d_4).

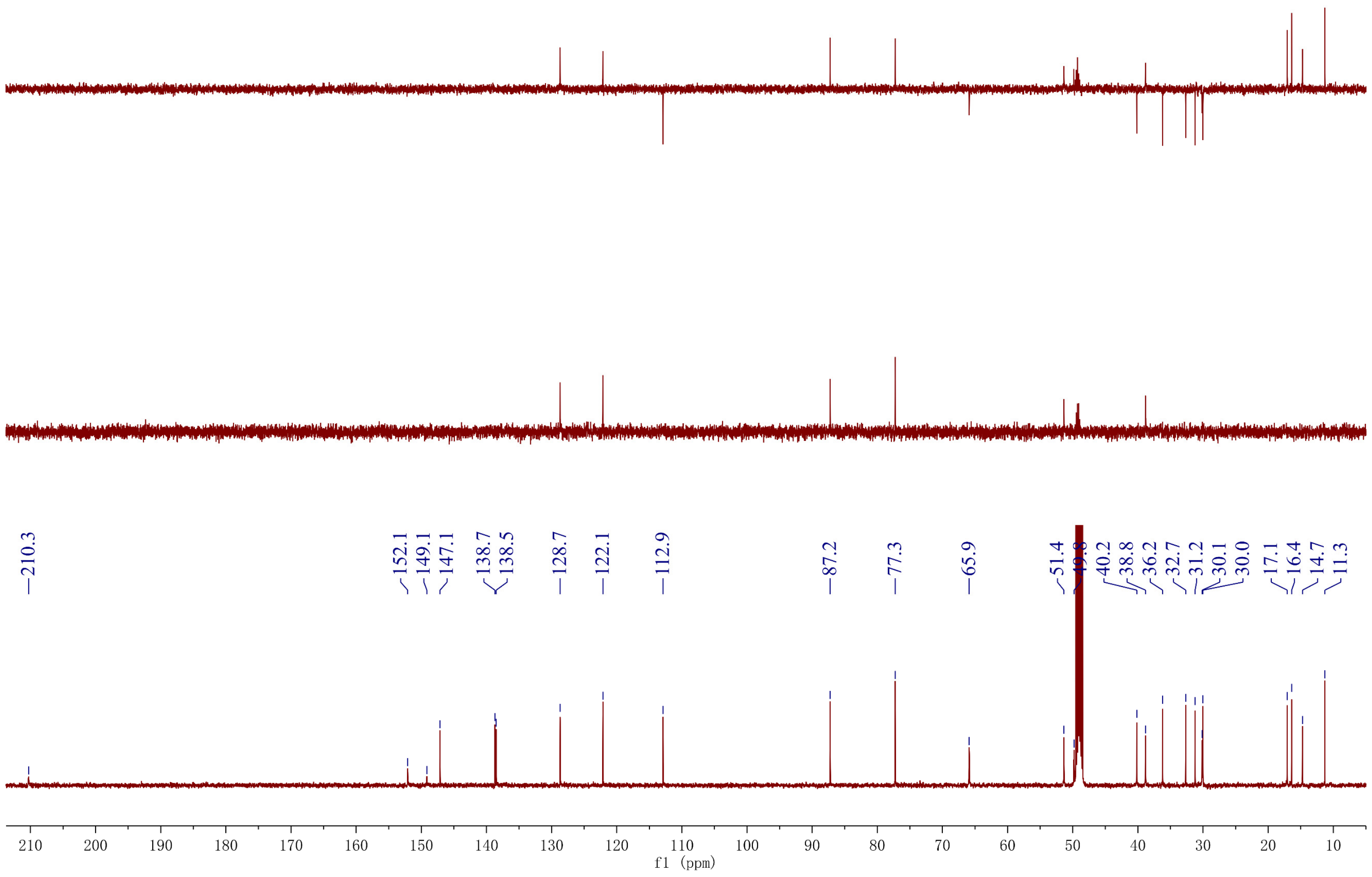

Figure S136. ^{13}C NMR and DEPT spectrum of synthetic **2** (Methanol-d_4).

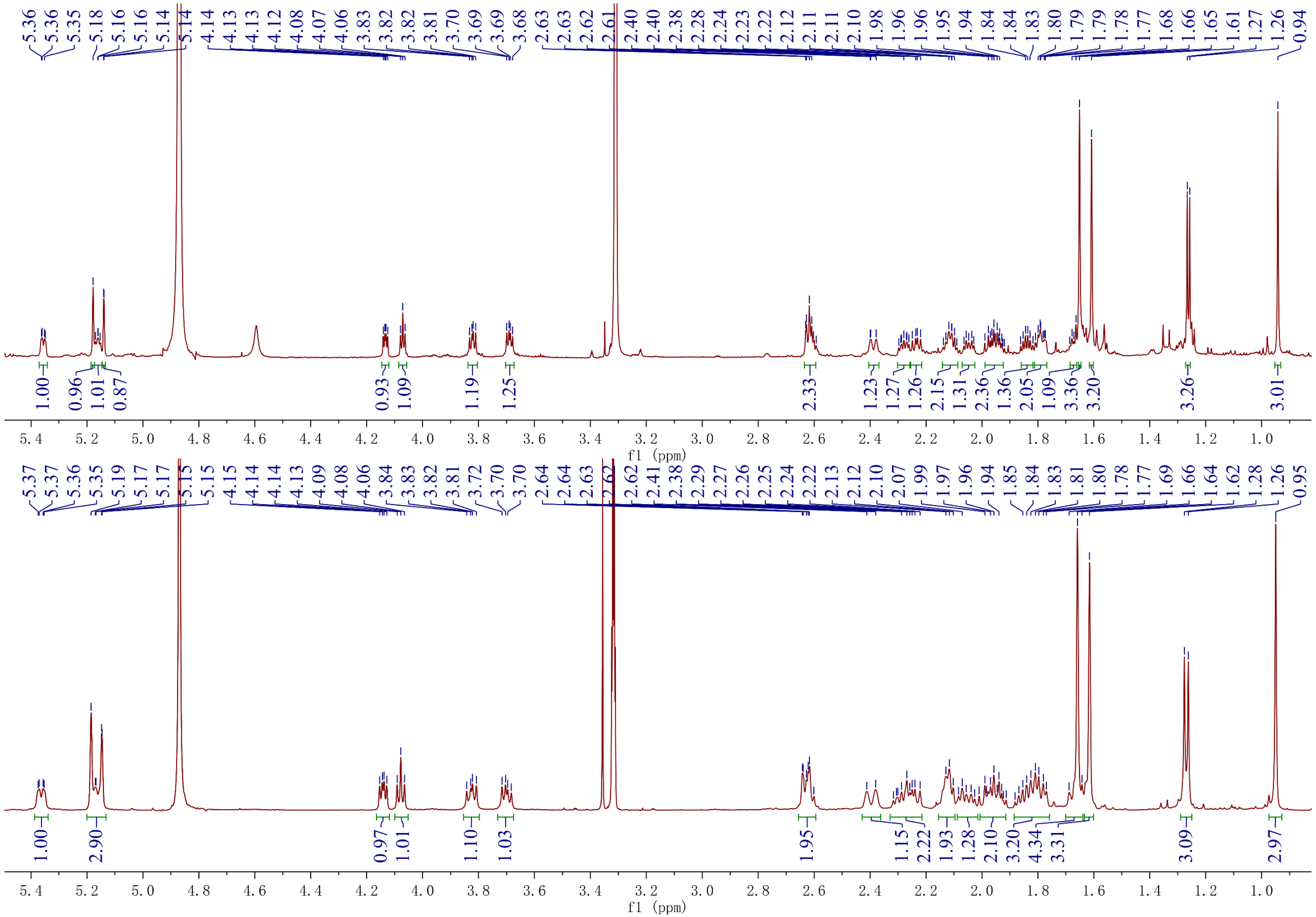

Figure S137. Comparison of ^{1}H NMR spectra of natural **2** (Methanol-d_4, upper) and synthetic **2** (Methanol-d_4, below).

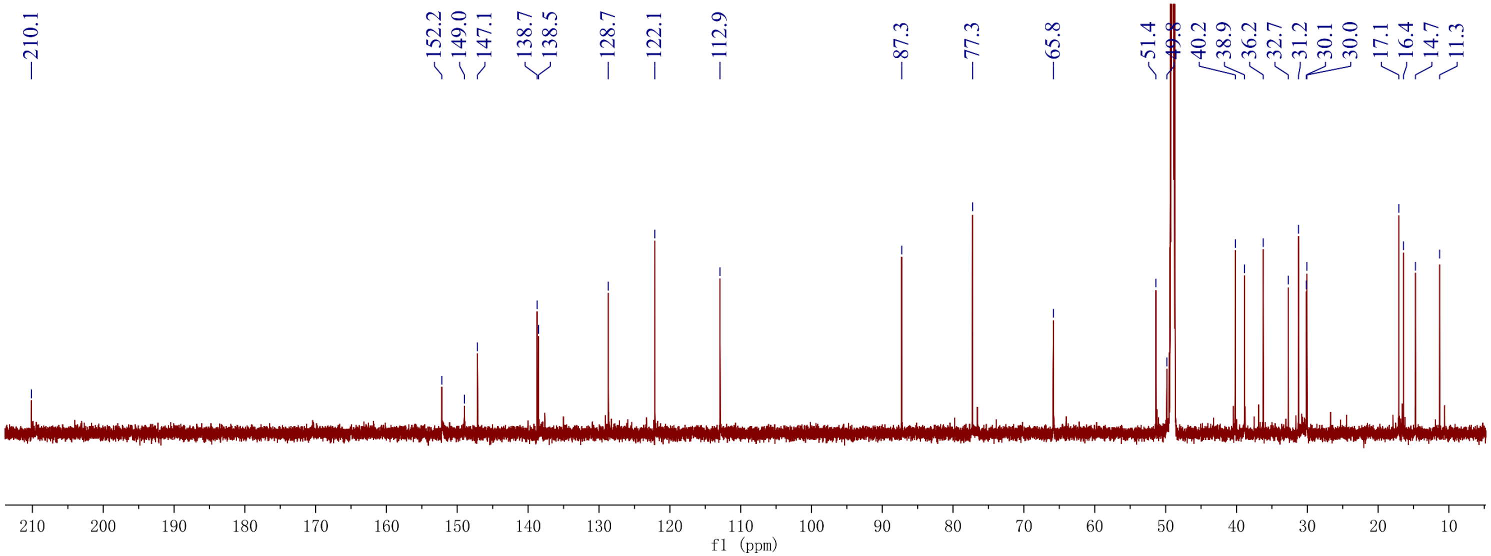

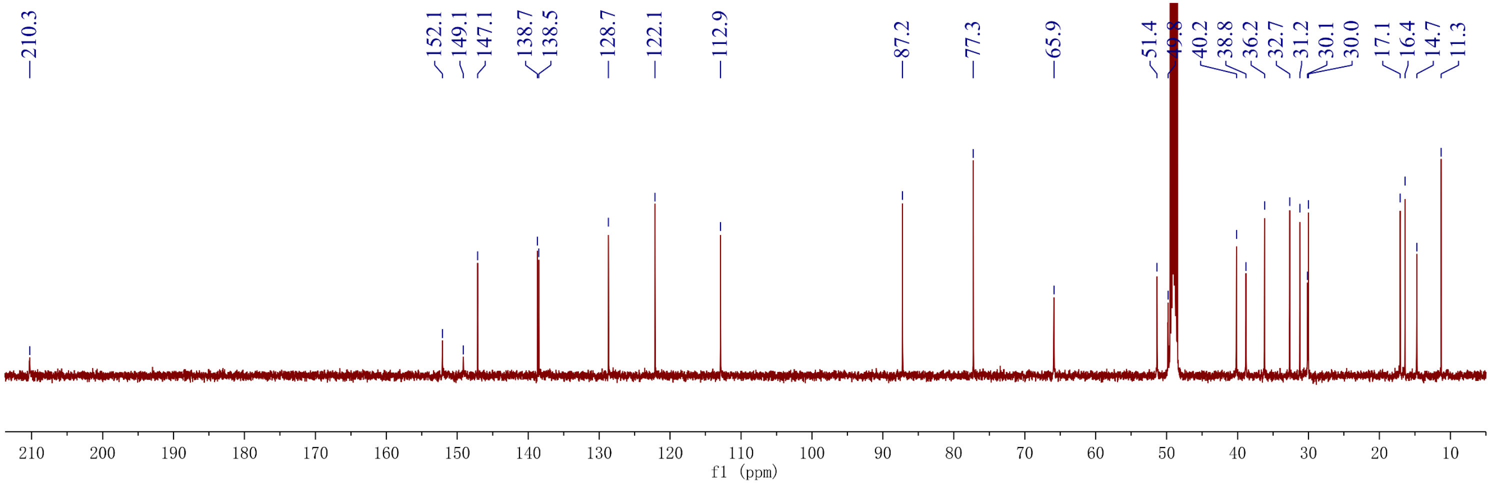

Figure S138. Comparison of ^{13}C NMR spectra of natural **2** (Methanol-d_4, upper) and synthetic **2** (Methanol-d_4, below).

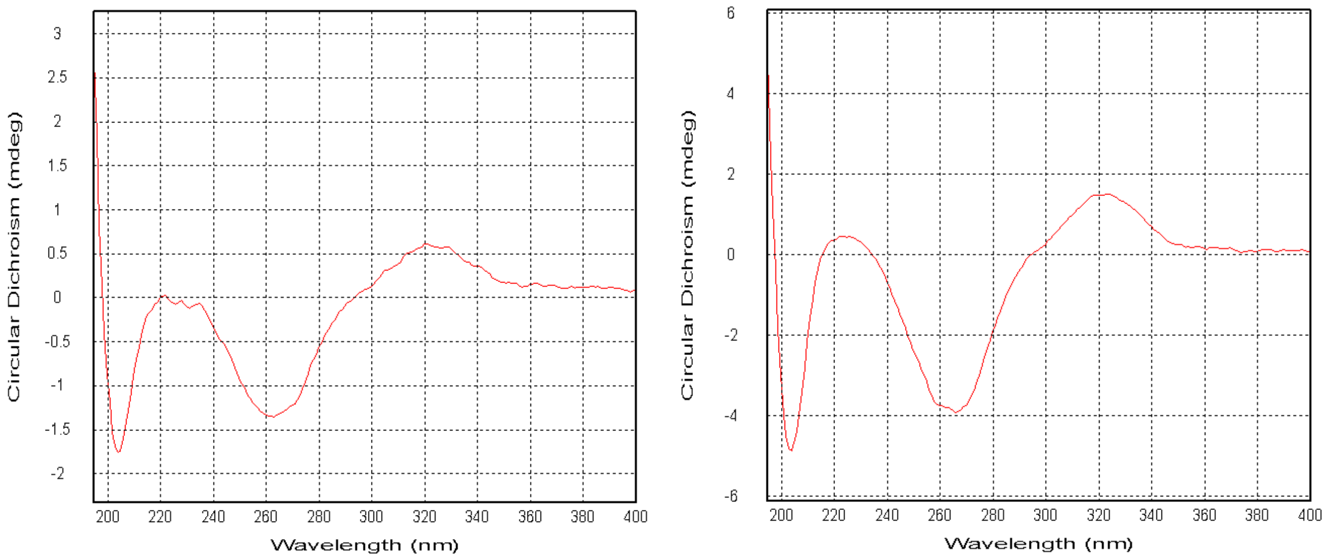

Figure S139. Comparison of ECD spectra of natural **2** (left) and synthetic **2** (right).

Rudolph Research Analytical

This sample was measured on an Autopol VI, Serial #91058
Manufactured by Rudolph Research Analytical, Hackettstown, NJ, USA.

Measurement Date : Friday, 28-MAY-2021

Set Temperature : OFF

Time Delay : Disabled

Delay between Measurement : Disabled

n	Average	Std.Dev.	% RSD	Maximum	Minimum
5	12.63	0.22	1.74	12.75	12.25

S.No	Sample ID	Time	Result	Scale	OR °Arc	WLG.nm	Lg.mm	Conc.g/100ml	Temp.
1	SHSJ26	10:56:19 AM	12.63	SR	0.0101	589	100.00	0.080	25.5
2	SHSJ26	10:56:27 AM	12.75	SR	0.0102	589	100.00	0.080	25.5
3	SHSJ26	10:56:35 AM	12.75	SR	0.0102	589	100.00	0.080	25.5
4	SHSJ26	10:56:44 AM	12.25	SR	0.0098	589	100.00	0.080	25.5
5	SHSJ26	10:56:52 AM	12.75	SR	0.0102	589	100.00	0.080	25.5

Rudolph Research Analytical

This sample was measured on an Autopol VI, Serial #91058
Manufactured by Rudolph Research Analytical, Hackettstown, NJ, USA.

Measurement Date : Tuesday, 02-JAN-2024

Set Temperature : 20.0

Time Delay : Disabled

Delay between Measurement : Disabled

n	Average	Std.Dev.	% RSD	Maximum	Minimum
5	24.29	0.00	0.00	24.29	24.29

S.No	Sample ID	Time	Result	Scale	OR °Arc	WLG.nm	Lg.mm	Conc.g/100ml	Temp.
1	SHSJR19	09:46:25 AM	24.29	SR	0.017	589	100.00	0.070	20.3
2	SHSJR19	09:46:31 AM	24.29	SR	0.017	589	100.00	0.070	20.2
3	SHSJR19	09:46:37 AM	24.29	SR	0.017	589	100.00	0.070	20.2
4	SHSJR19	09:46:44 AM	24.29	SR	0.017	589	100.00	0.070	20.2
5	SHSJR19	09:46:50 AM	24.29	SR	0.017	589	100.00	0.070	20.1

Figure S140. Comparison of ORD reports of natural **2** (upper) and synthetic **2** (below).

User Spectra

Fragmentor Voltage	Collision Energy	Ionization Mode
135	0	ESI

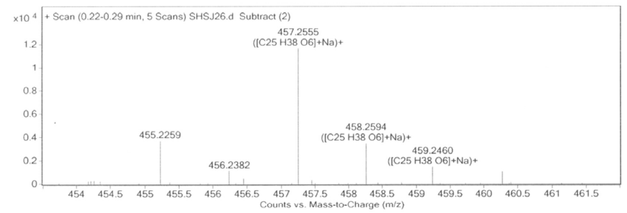

Peak List

m/z	z	Abund	Formula	Ion
274.2743	1	19733.72		
302.306	1	7544.31		
318.301	1	10747.7		
359.3175		6756.35		
381.298	1	7139.05		
399.2541	1	8276.84		
401.2686	1	9210.06		
417.2639	1	11656.33		
435.2749	1	12266.15		
457.2555	1	11678.55	C25 H38 O6	(M+Na)+

Formula Calculator Element Limits

Element	Min	Max
C	3	60
H	0	120
O	0	30

Formula Calculator Results

Formula	CalculatedMass	CalculatedMz	Mz	Diff. (mDa)	Diff. (ppm)	DBE
C25 H38 O6	434.2668	457.2561	457.2555	0.60	1.31	7.0000

--- End Of Report ---

User Spectra

Fragmentor Voltage	Collision Energy	Ionization Mode
135	0	ESI

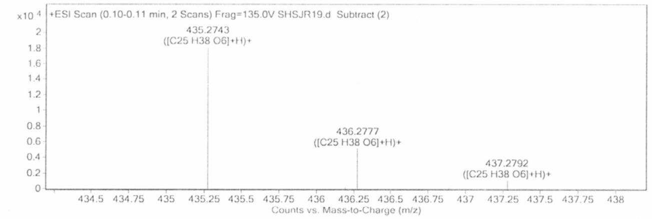

Peak List

m/z	z	Abund	Formula	Ion
399.2527	1	1842.71		
400.2513	1	1142.2		
401.2674	1	1634.17		
417.2634	1	6646.91		
418.2711	1	2191.41		
435.2743	1	18073.71	C25 H38 O6	(M+H)+
436.2777	1	5151.72	C25 H38 O6	(M+H)+
457.2563	1	10541.74		
458.26	1	2763.57		
519.2269	1	1151.73		

Formula Calculator Element Limits

Element	Min	Max
C	3	60
H	0	150
O	0	20

Formula Calculator Results

Formula	CalculatedMass	CalculatedMz	Mz	Diff. (mDa)	Diff. (ppm)	DBE
C25 H38 O6	434.2668	435.2741	435.2743	-0.20	-0.46	7.0000

--- End Of Report ---

Figure S141. Comparison of HRESIMS spectra of natural **2** (left) and synthetic **2** (right).

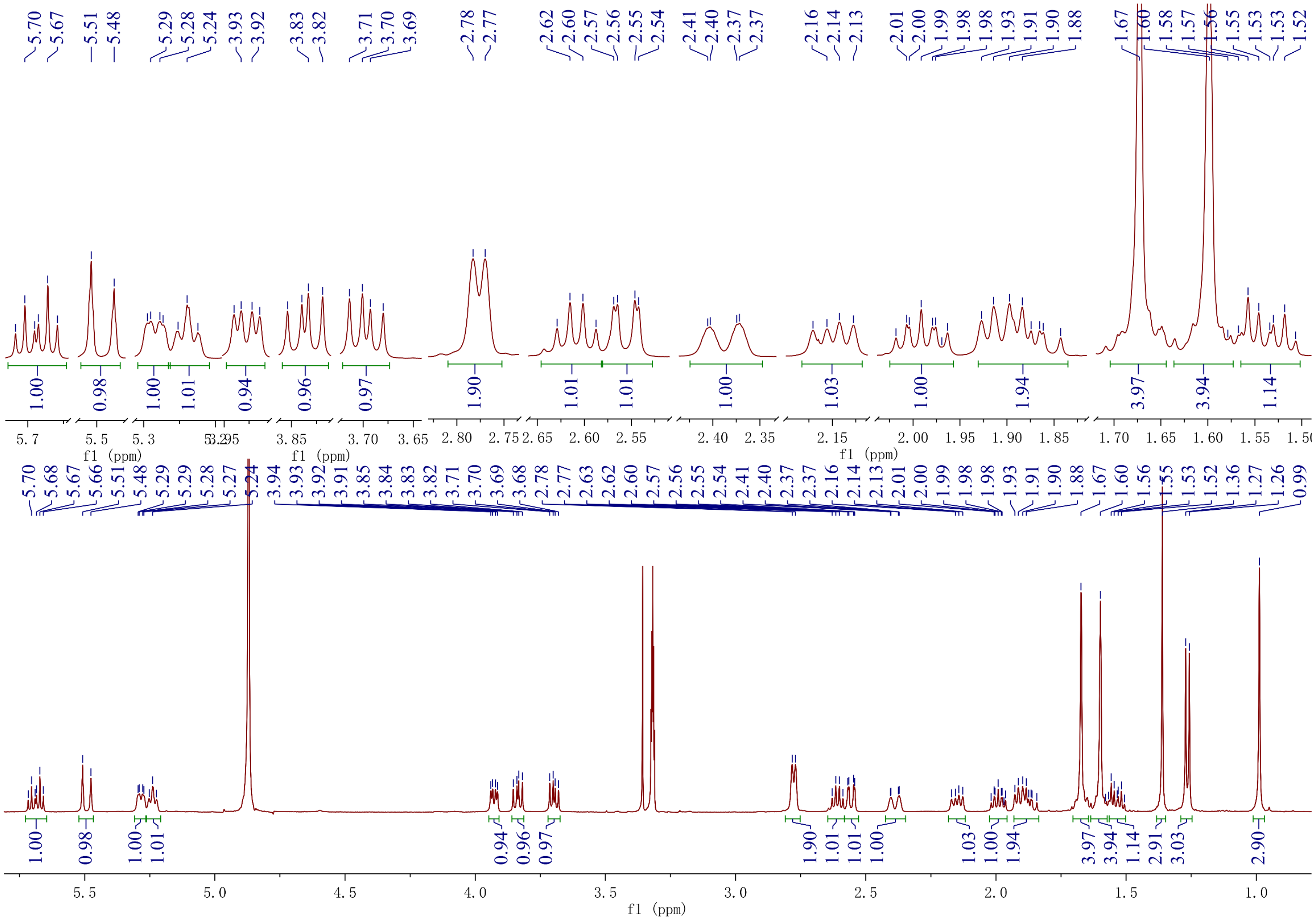

Figure S142. ^{1}H NMR spectrum of synthetic **3** (Methanol-d_4).

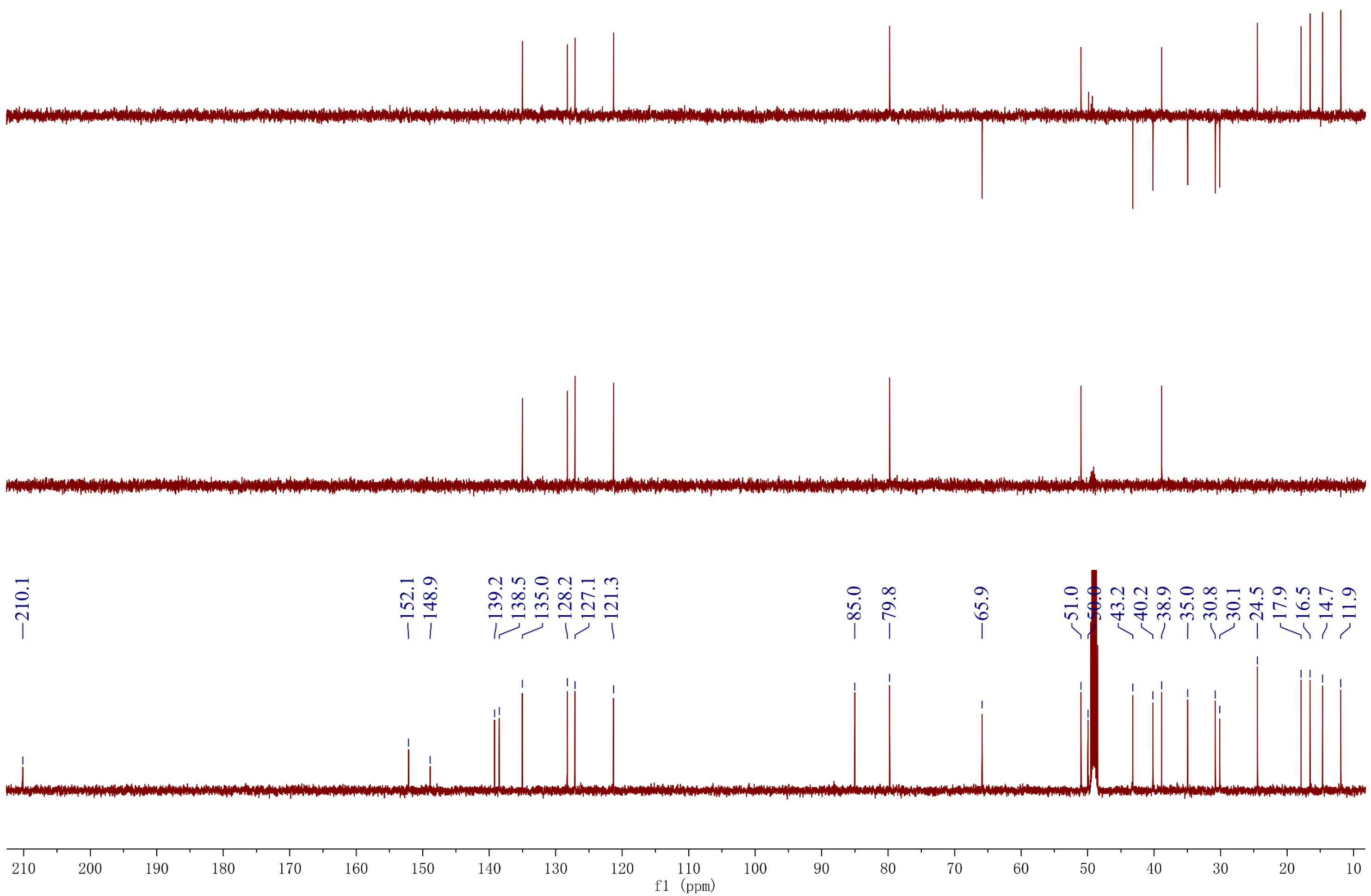

Figure S143. ^{13}C NMR and DEPT spectrum of synthetic **3** (Methanol-d_4).

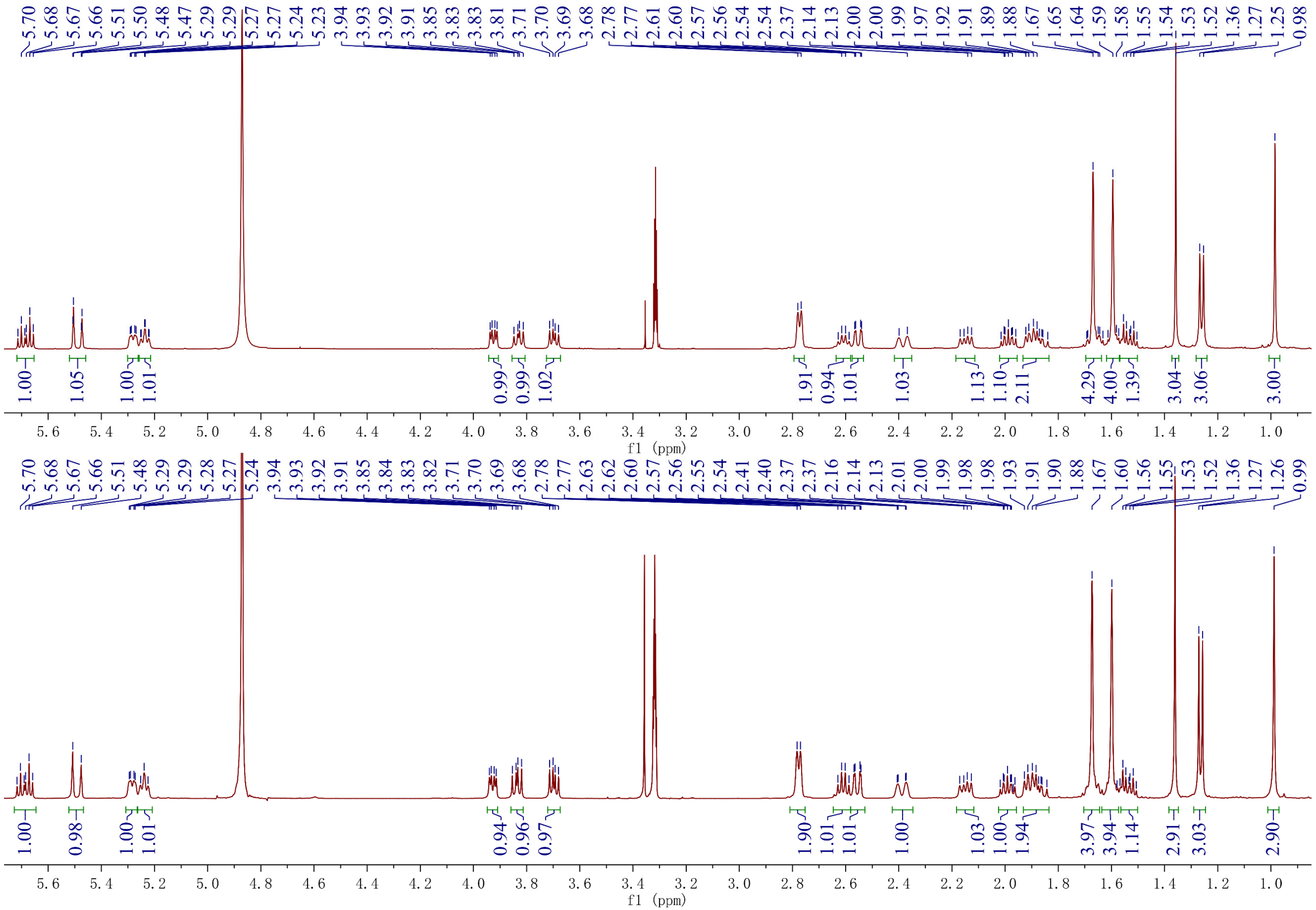

Figure S144. Comparison of ^{1}H NMR spectra of natural **3** (Methanol-d_4, upper) and synthetic **3** (Methanol-d_4, below).

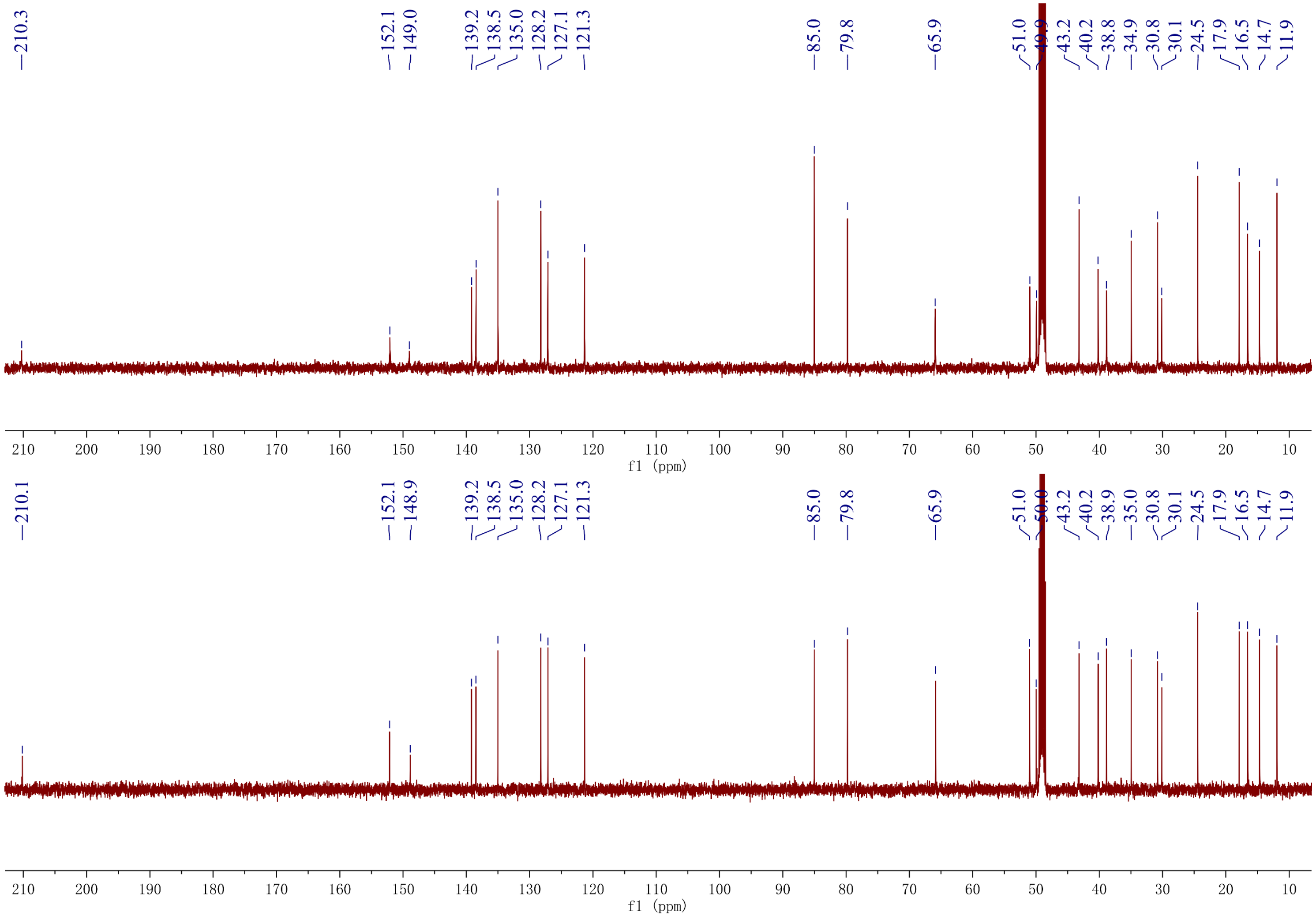

Figure S145. Comparison of ^{13}C NMR spectra of natural **3** (Methanol-d_4, upper) and synthetic **3** (Methanol-d_4, below).

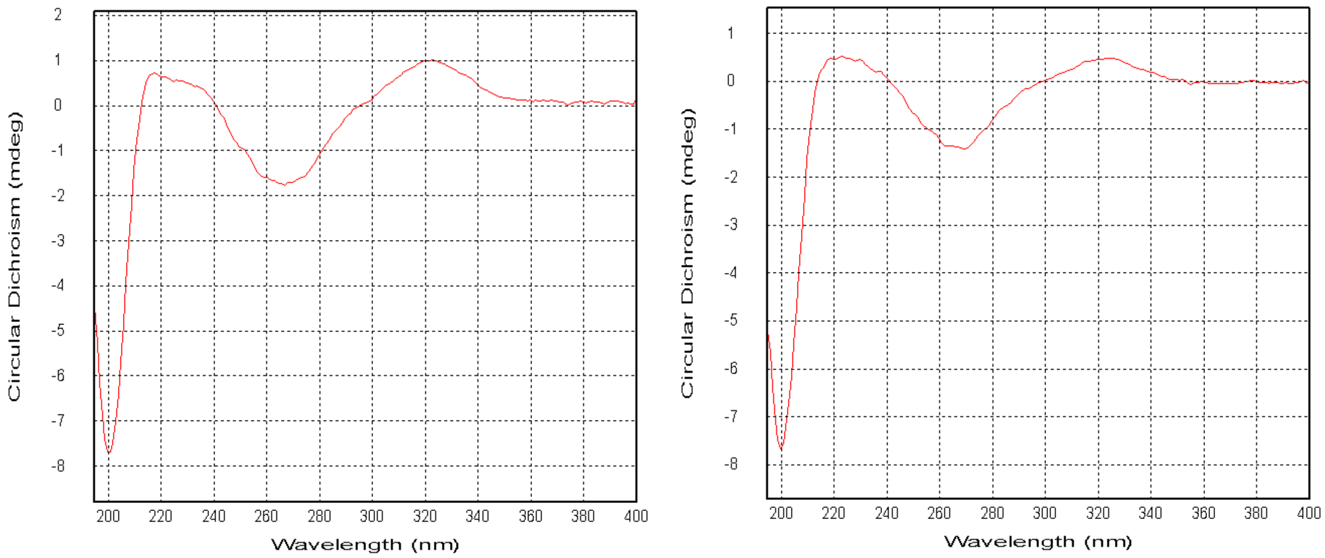

Figure S146. Comparison of ECD spectra of natural **3** (left) and synthetic **3** (right).

Rudolph Research Analytical

This sample was measured on an Autopol VI, Serial #91058
Manufactured by Rudolph Research Analytical, Hackettstown, NJ, USA.

Measurement Date : Tuesday, 02-JAN-2024

Set Temperature : 20.0

Time Delay : Disabled

Delay between Measurement : Disabled

n	Average	Std.Dev.	% RSD	Maximum	Minimum
5	2.25	0.84	37.33	3.75	1.88

S.No	Sample ID	Time	Result	Scale	OR °Arc	WLG.nm	Lg.mm	Conc.g/100ml	Temp.
1	SHSJR20	09:39:27 AM	3.75	SR	0.006	589	100.00	0.160	20.7
2	SHSJR20	09:39:33 AM	1.88	SR	0.003	589	100.00	0.160	20.7
3	SHSJR20	09:39:40 AM	1.88	SR	0.003	589	100.00	0.160	20.6
4	SHSJR20	09:39:46 AM	1.88	SR	0.003	589	100.00	0.160	20.5
5	SHSJR20	09:39:52 AM	1.88	SR	0.003	589	100.00	0.160	20.4

Rudolph Research Analytical

This sample was measured on an Autopol VI, Serial #91058
Manufactured by Rudolph Research Analytical, Hackettstown, NJ, USA.

Measurement Date : Tuesday, 30-JAN-2024

Set Temperature : 20.0

Time Delay : Disabled

Delay between Measurement : Disabled

n	Average	Std.Dev.	% RSD	Maximum	Minimum
5	1.40	0.89	63.57	3.00	1.00

S.No	Sample ID	Time	Result	Scale	OR °Arc	WLG.nm	Lg.mm	Conc.g/100ml	Temp.
1	SHSJR20A	12:05:15 PM	1.00	SR	0.001	589	100.00	0.100	19.9
2	SHSJR20A	12:05:21 PM	3.00	SR	0.003	589	100.00	0.100	19.9
3	SHSJR20A	12:05:27 PM	1.00	SR	0.001	589	100.00	0.100	20.0
4	SHSJR20A	12:05:34 PM	1.00	SR	0.001	589	100.00	0.100	20.0
5	SHSJR20A	12:05:40 PM	1.00	SR	0.001	589	100.00	0.100	20.0

Figure S147. Comparison of ORD reports of natural **3** (upper) and synthetic **3** (below).

User Spectra

Fragmentor Voltage	Collision Energy	Ionization Mode
135	0	ESI

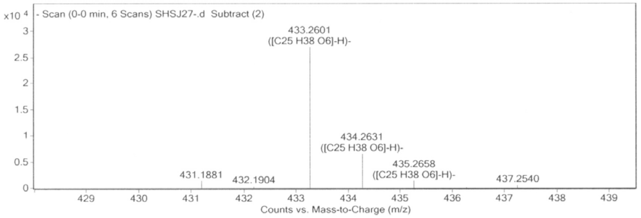

Peak List

m/z	z	Abund	Formula	Ion
89.0244	1	7591.31		
255.2326	1	3640.46		
283.2644	1	4161.78		
433.2601	1	26927.04	C25 H38 O6	(M-H)-
434.2631	1	6487.27	C25 H38 O6	(M-H)-
469.237	1	2407.77		
499.1738	1	1806.8		
501.2452	1	1461.75		
523.2908	1	2321.51		
547.2511		1343.94		

Formula Calculator Element Limits

Element	Min	Max
C	3	60
H	0	120
O	0	30
N	0	5

Formula Calculator Results

Formula	CalculatedMass	CalculatedMz	Mz	Diff. (mDa)	Diff. (ppm)	DBE
C25 H38 O6	434.2668	433.2596	433.2601	-0.50	-1.15	7.0000

--- End Of Report ---

User Spectra

Fragmentor Voltage	Collision Energy	Ionization Mode
135	0	ESI

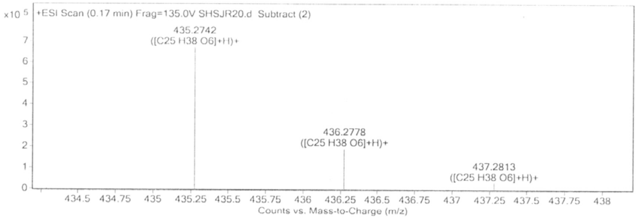

Peak List

m/z	z	Abund	Formula	Ion
401.2682	1	26365.45		
417.2634	1	89581.93		
435.2742	1	672156.69	C25 H38 O6	(M+H)+
436.2778	1	189705.41	C25 H38 O6	(M+H)+
437.2813	1	29702.89	C25 H38 O6	(M+H)+
441.2609	1	58528.23		
457.2559	1	148255.64		
458.2592	1	40816.92		
891.5236	1	124846.31		
892.527	1	69500.02		

Formula Calculator Element Limits

Element	Min	Max
C	3	60
H	0	150
O	0	20

Formula Calculator Results

Formula	CalculatedMass	CalculatedMz	Mz	Diff. (mDa)	Diff. (ppm)	DBE
C25 H38 O6	434.2668	435.2741	435.2742	-0.10	-0.23	7.0000

--- End Of Report ---

Figure S148. Comparison of HRESIMS spectra of natural **3** (left) and synthetic **3** (right).

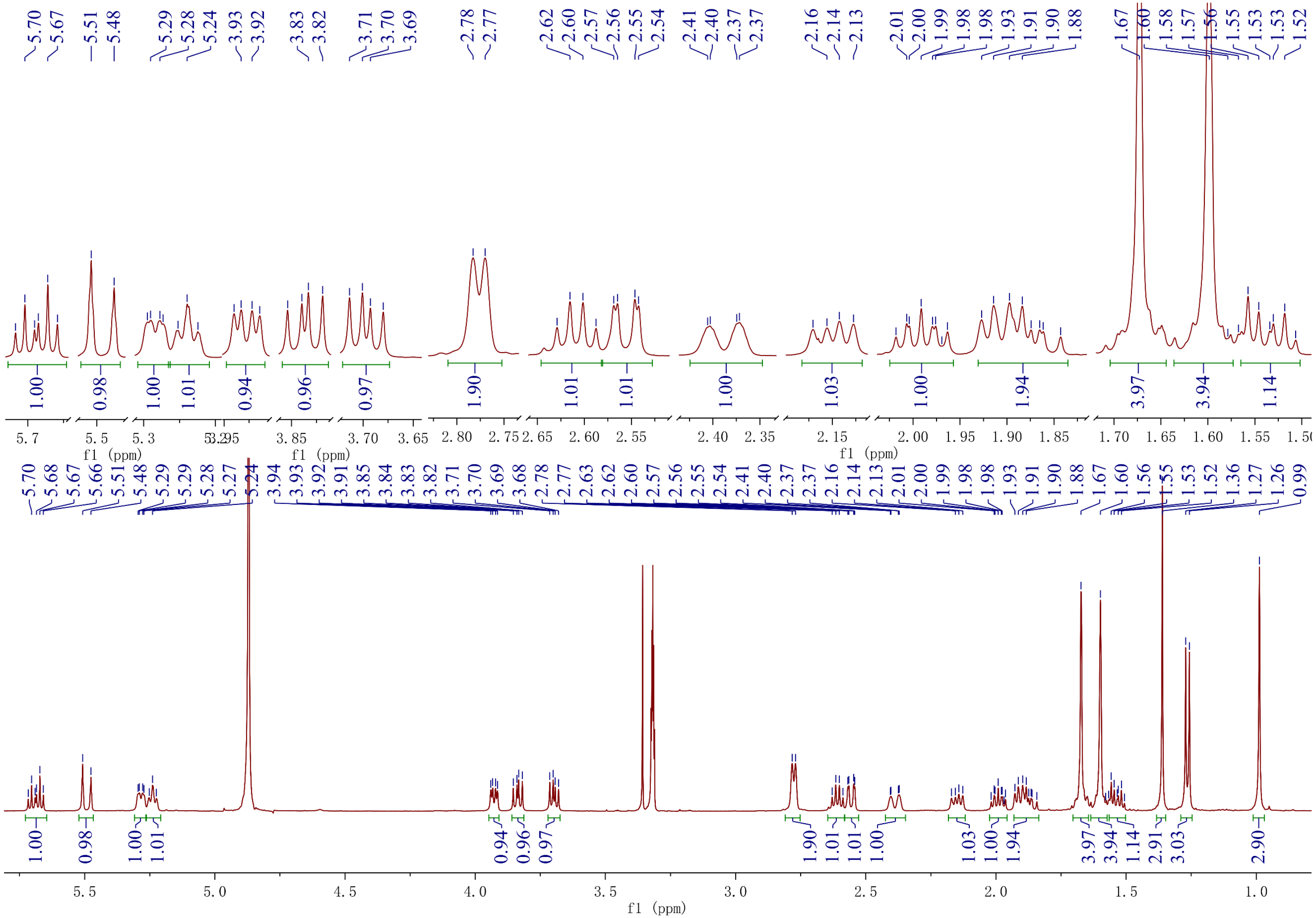

Figure S149. ^{1}H NMR spectrum of synthetic **4** (Methanol-d_4).

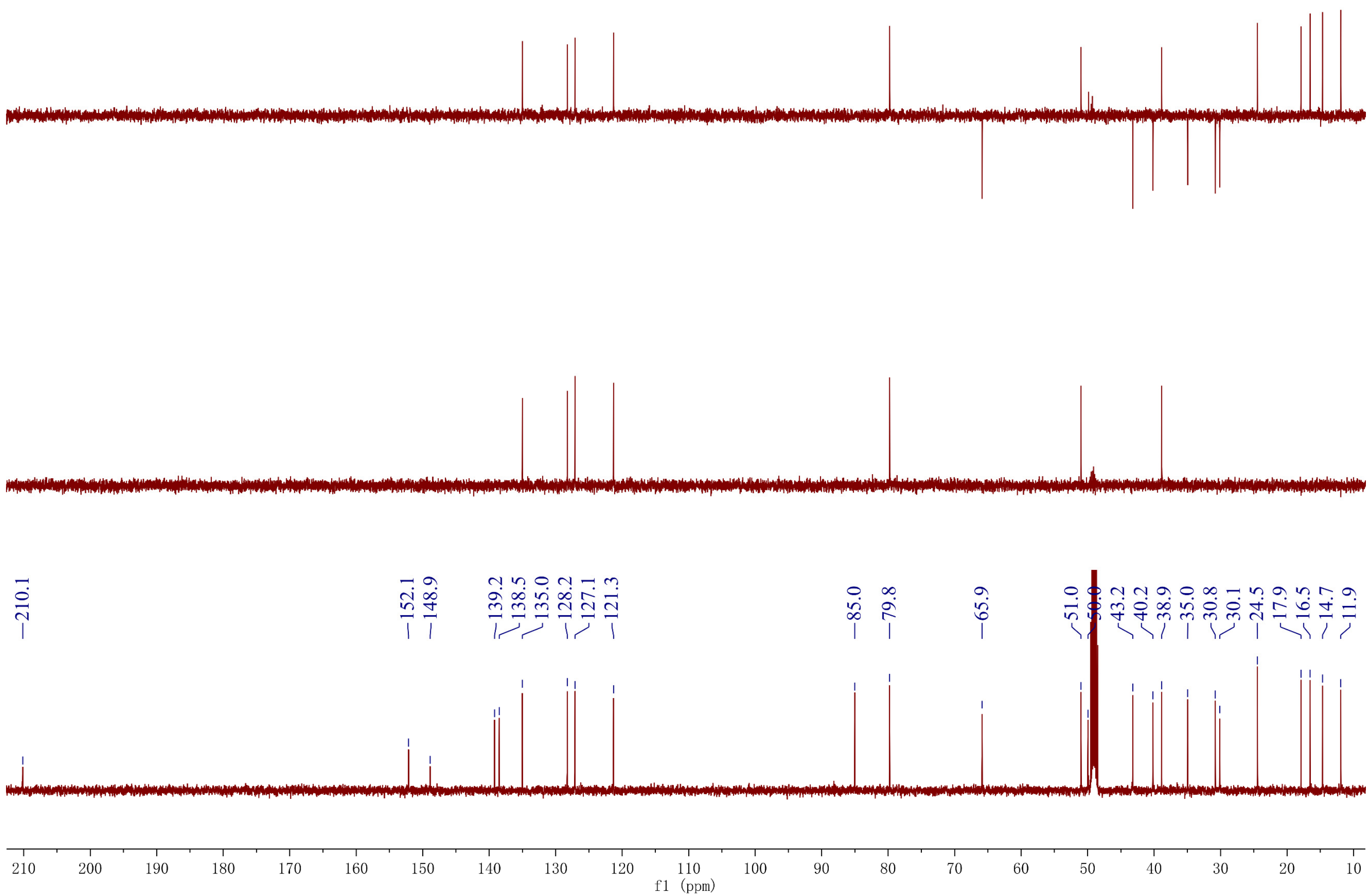

Figure S150. ^{13}C NMR and DEPT spectrum of synthetic **4** (Methanol-d_4).

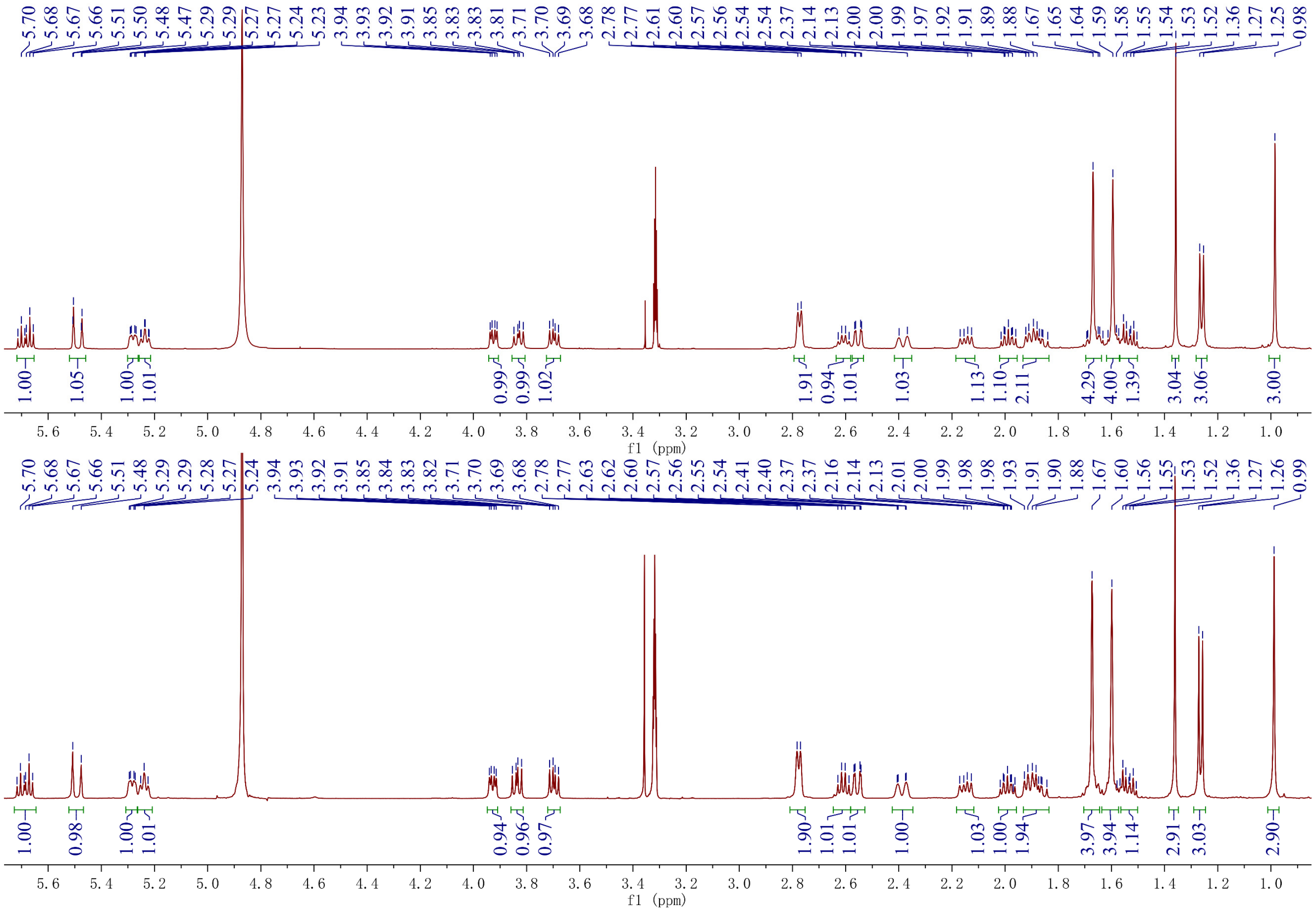

Figure S151. Comparison of ^{1}H NMR spectra of natural **4** (Methanol-d_4, upper) and synthetic **4** (Methanol-d_4, below).

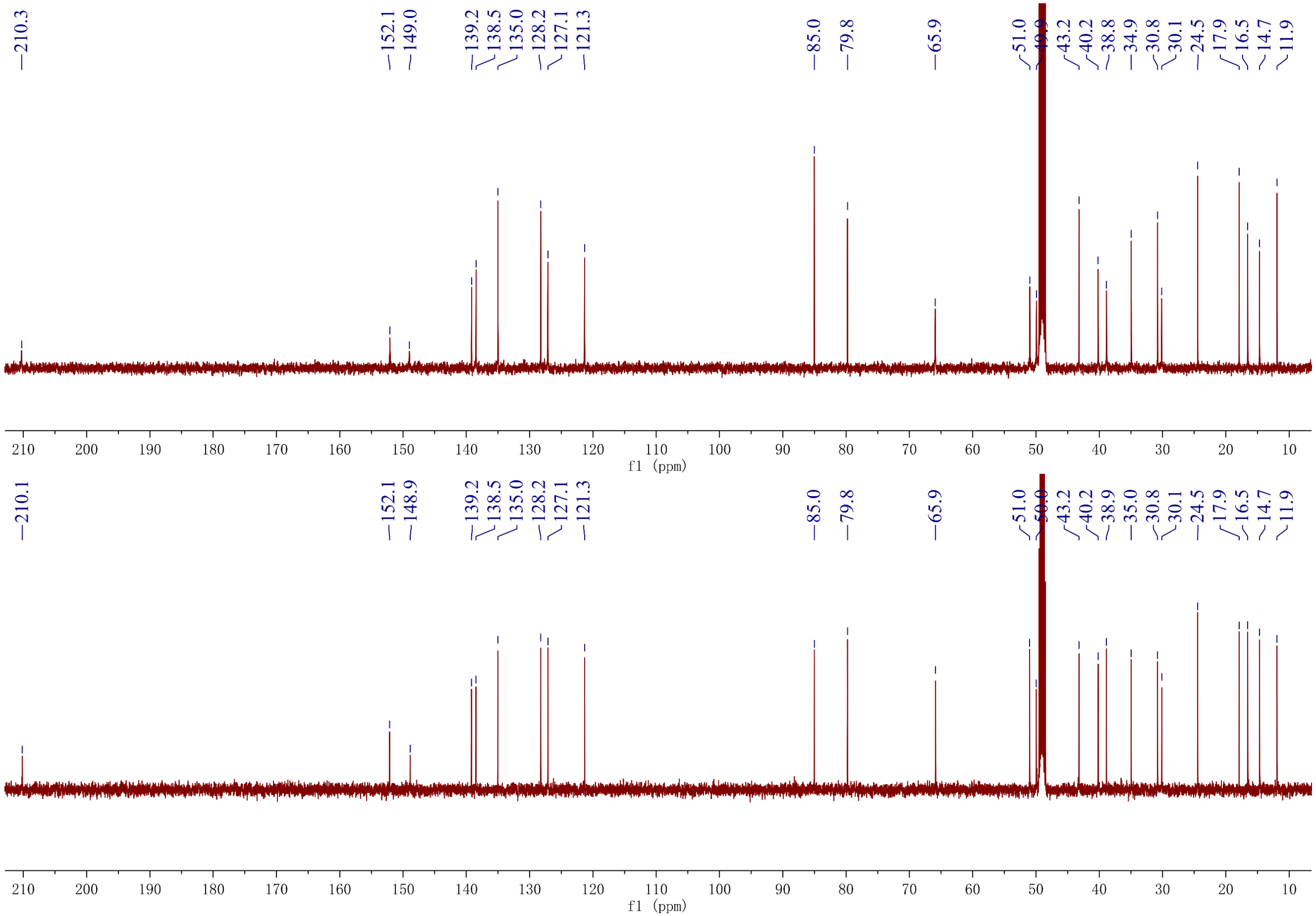

Figure S152. Comparison of ^{13}C NMR spectra of natural **4** (Methanol-d_4, upper) and synthetic **4** (Methanol-d_4, below).

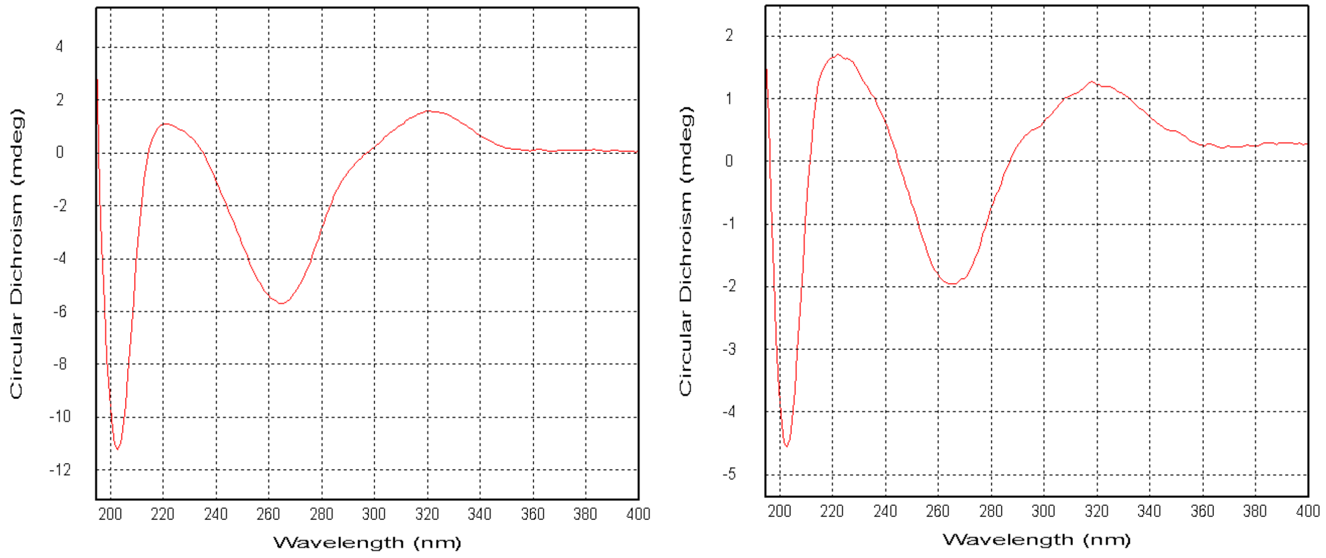

Figure S153. Comparison of ECD spectra of natural **4** (left) and synthetic **4** (right).

Rudolph Research Analytical

This sample was measured on an Autopol VI, Serial #91058
Manufactured by Rudolph Research Analytical, Hackettstown, NJ, USA.

Measurement Date : Friday, 28-MAY-2021

Set Temperature : OFF

Time Delay : Disabled

Delay between Measurement : Disabled

n	Average	Std.Dev.	% RSD	Maximum	Minimum
5	24.39	0.14	0.57	24.53	24.20

S.No	Sample ID	Time	Result	Scale	OR °Arc	WLG.nm	Lg.mm	Conc.g/100ml	Temp.
1	SHSJ44	10:01:08 AM	24.20	SR	0.0363	589	100.00	0.150	24.5
2	SHSJ44	10:01:16 AM	24.53	SR	0.0368	589	100.00	0.150	24.5
3	SHSJ44	10:01:24 AM	24.27	SR	0.0364	589	100.00	0.150	24.5
4	SHSJ44	10:01:33 AM	24.47	SR	0.0367	589	100.00	0.150	24.5
5	SHSJ44	10:01:41 AM	24.47	SR	0.0367	589	100.00	0.150	24.5

Rudolph Research Analytical

This sample was measured on an Autopol VI, Serial #91058
Manufactured by Rudolph Research Analytical, Hackettstown, NJ, USA.

Measurement Date : Tuesday, 02-JAN-2024

Set Temperature : 20.0

Time Delay : Disabled

Delay between Measurement : Disabled

n	Average	Std.Dev.	% RSD	Maximum	Minimum
5	6.50	0.56	8.61	7.50	6.25

S.No	Sample ID	Time	Result	Scale	OR °Arc	WLG.nm	Lg.mm	Conc.g/100ml	Temp.
1	SHSJR25	10:46:47 AM	6.25	SR	0.005	589	100.00	0.080	20.5
2	SHSJR25	10:46:53 AM	6.25	SR	0.005	589	100.00	0.080	20.4
3	SHSJR25	10:46:59 AM	6.25	SR	0.005	589	100.00	0.080	20.4
4	SHSJR25	10:47:06 AM	6.25	SR	0.005	589	100.00	0.080	20.3
5	SHSJR25	10:47:12 AM	7.50	SR	0.006	589	100.00	0.080	20.3

Figure S154. Comparison of ORD reports of natural **4** (upper) and synthetic **4** (below).

User Spectra

Fragmentor Voltage	Collision Energy	Ionization Mode
135	0	ESI

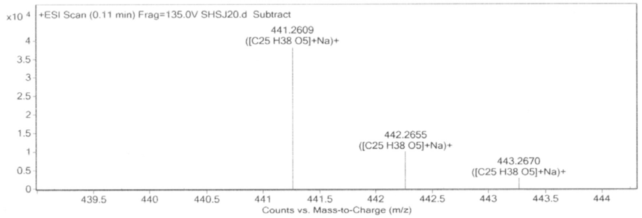

Peak List

m/z	z	Abund	Formula	Ion
102.1278	1	18761.85		
118.1229		4974.24		
251.1261	1	5738.05		
401.2684	1	21141.05		
402.2726	1	5871.94		
441.2609	1	37975.18	C25 H38 O5	(M+Na)+
442.2655	1	9870.73	C25 H38 O5	(M+Na)+
453.1681	1	5327.82		
654.3311	1	10755.1		
659.2876	1	7428.39		

Formula Calculator Element Limits

Element	Min	Max
C	3	60
H	0	120
O	0	30

Formula Calculator Results

Formula	CalculatedMass	CalculatedMz	Mz	Diff. (mDa)	Diff. (ppm)	DBE
C25 H38 O5	418.2719	441.2611	441.2609	0.20	0.45	7.0000

--- End Of Report ---

User Spectra

Fragmentor Voltage	Collision Energy	Ionization Mode
165	30	ESI

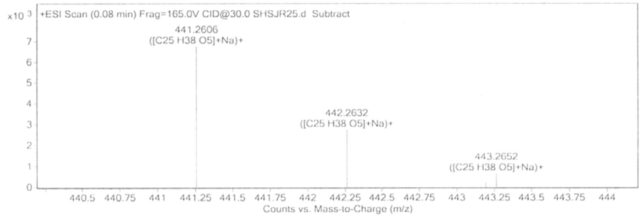

Peak List

m/z	z	Abund	Formula	Ion
85.0593	1	1346.26		
89.0598	1	1620.36		
95.0859	1	1804.82		
109.1012	1	1431.6		
121.0509	1	1611.01		
355.0689	1	1498.12		
429.0896	1	1607.21		
438.1964	1	1282.79		
441.2606	1	6749.26	C25 H38 O5	(M+Na)+
442.2632	1	2749.55	C25 H38 O5	(M+Na)+

Formula Calculator Element Limits

Element	Min	Max
C	3	60
H	0	150
O	0	20
N	0	5

Formula Calculator Results

Formula	CalculatedMass	CalculatedMz	Mz	Diff. (mDa)	Diff. (ppm)	DBE
C25 H38 O5	418.2719	441.2611	441.2606	0.50	1.13	7.0000

--- End Of Report ---

Figure S155. Comparison of HRESIMS spectra of natural **4** (left) and synthetic **4** (right).

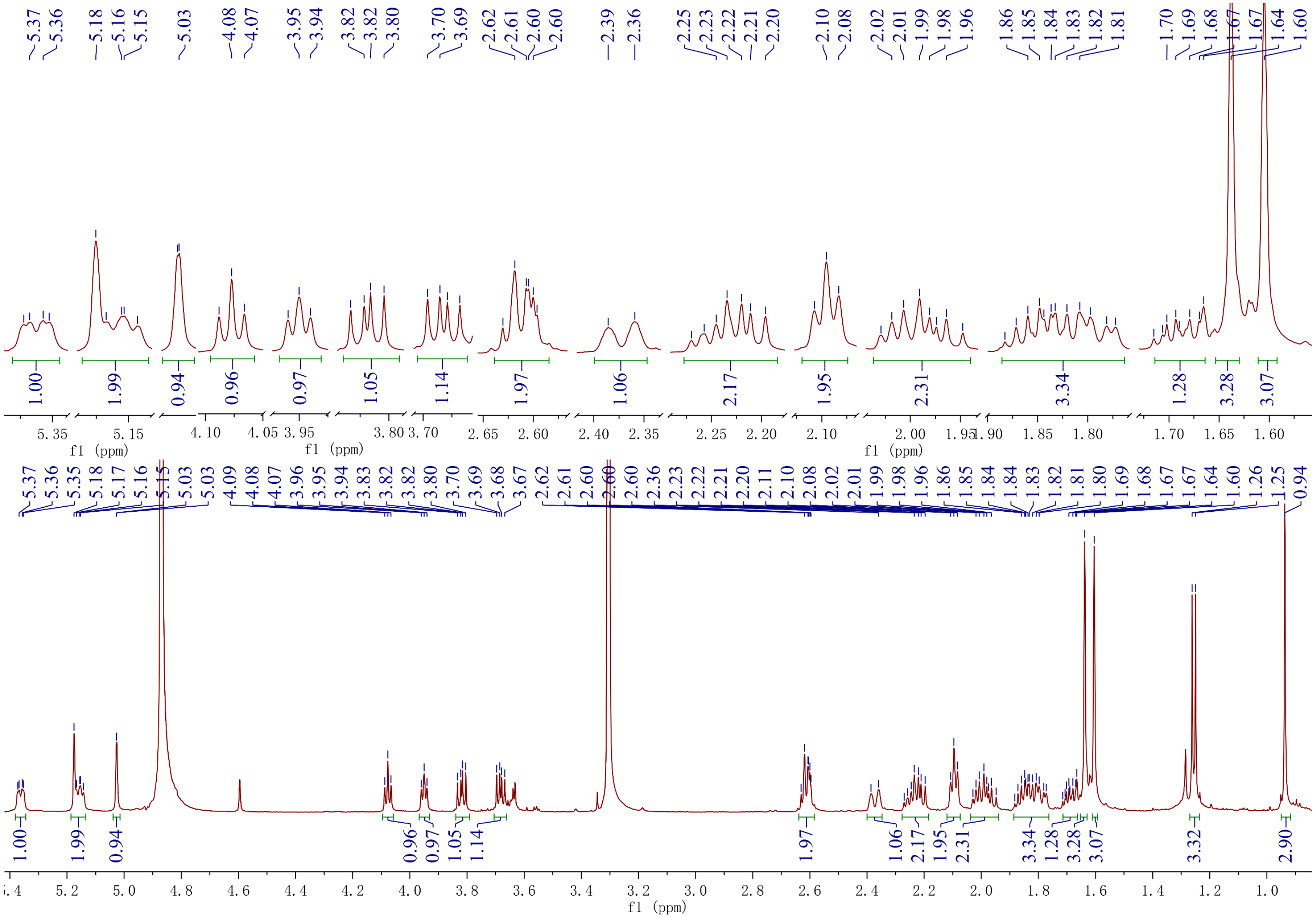

Figure S156. ^{1}H NMR spectrum of synthetic **5** (Methanol-d_4).

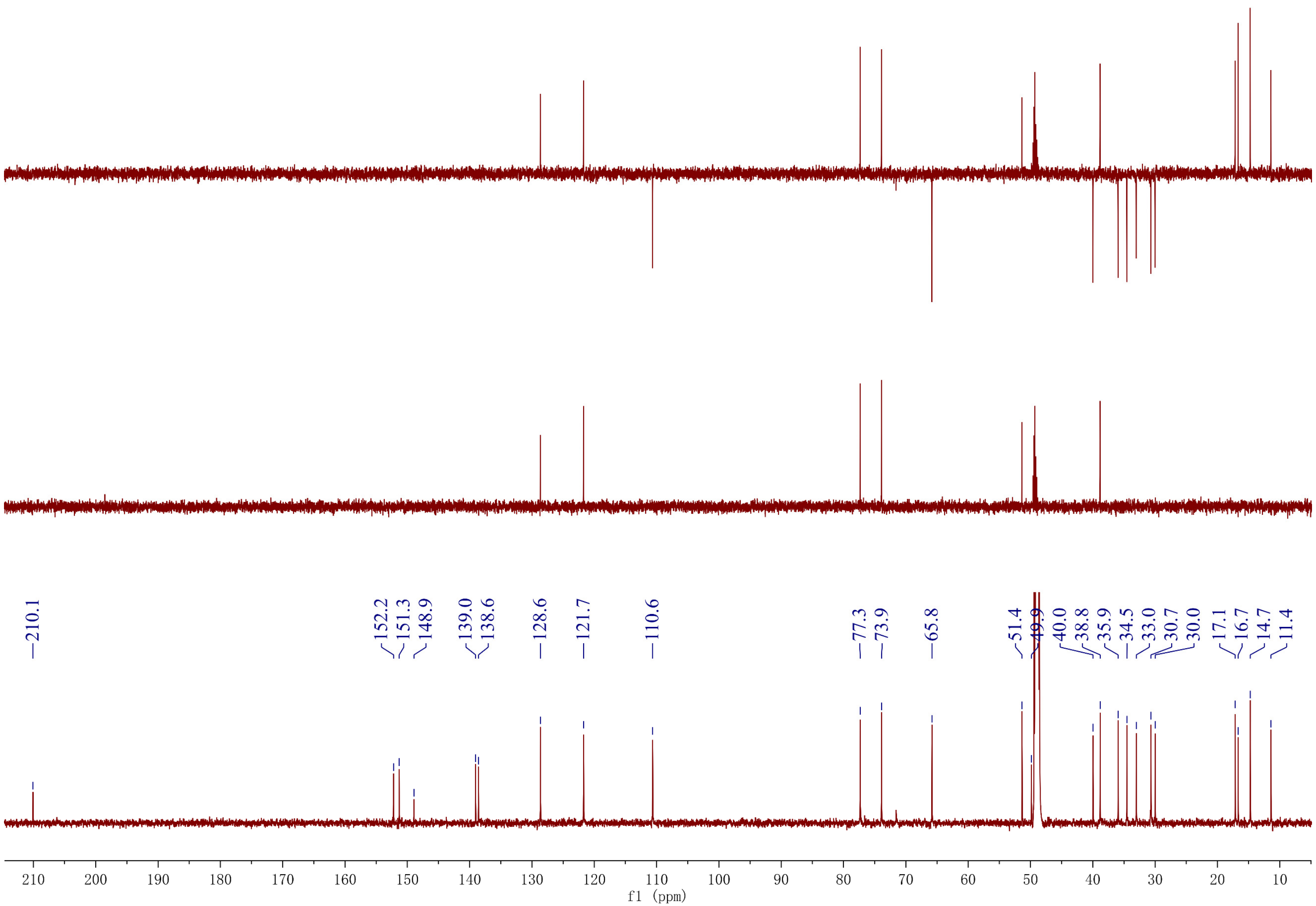

Figure S157. ^{13}C NMR and DEPT spectrum of synthetic **5** (Methanol-d_4).

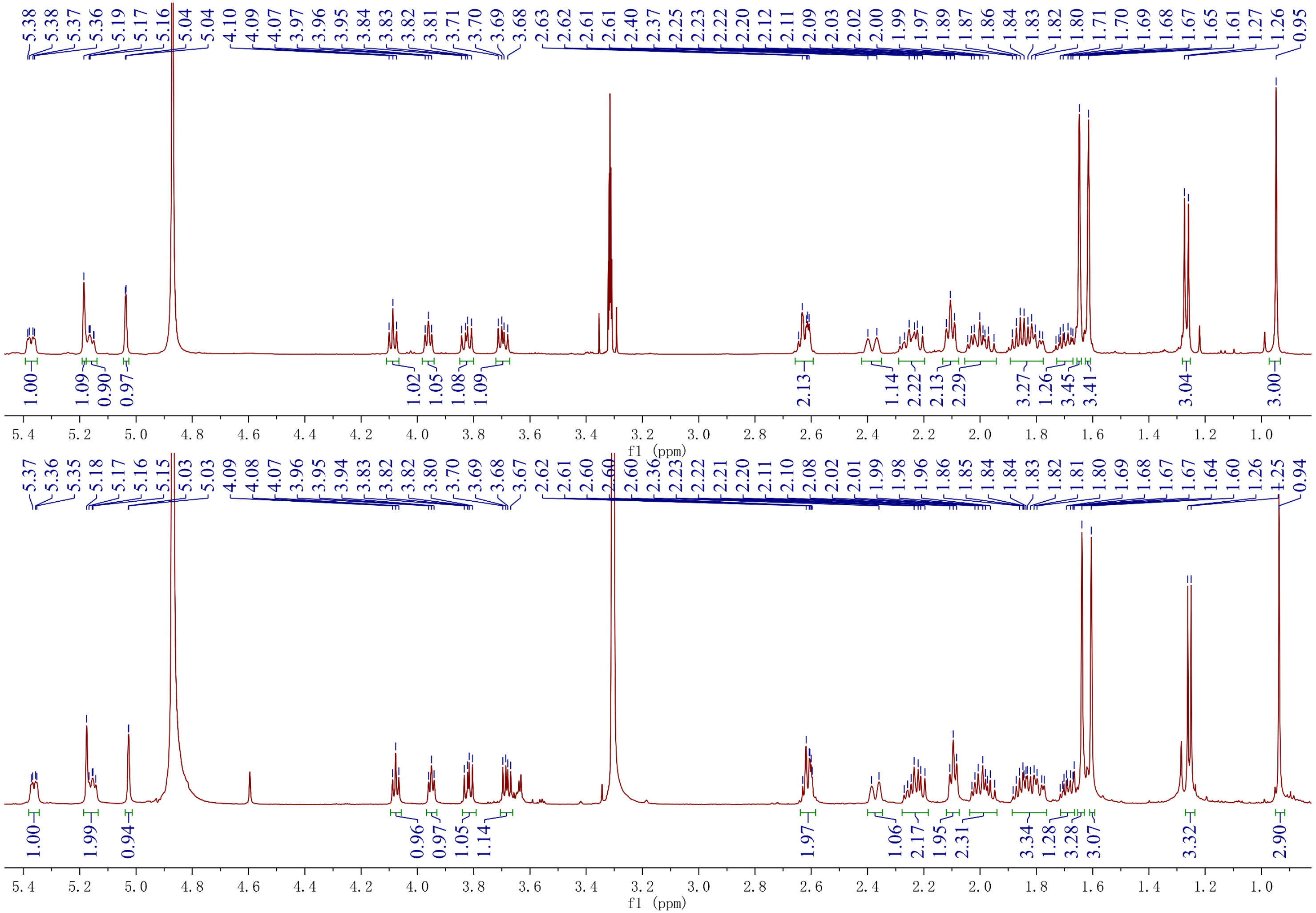

Figure S158. Comparison of ^{1}H NMR spectra of natural **5** (Methanol-d_4, upper) and synthetic **5** (Methanol-d_4, below).

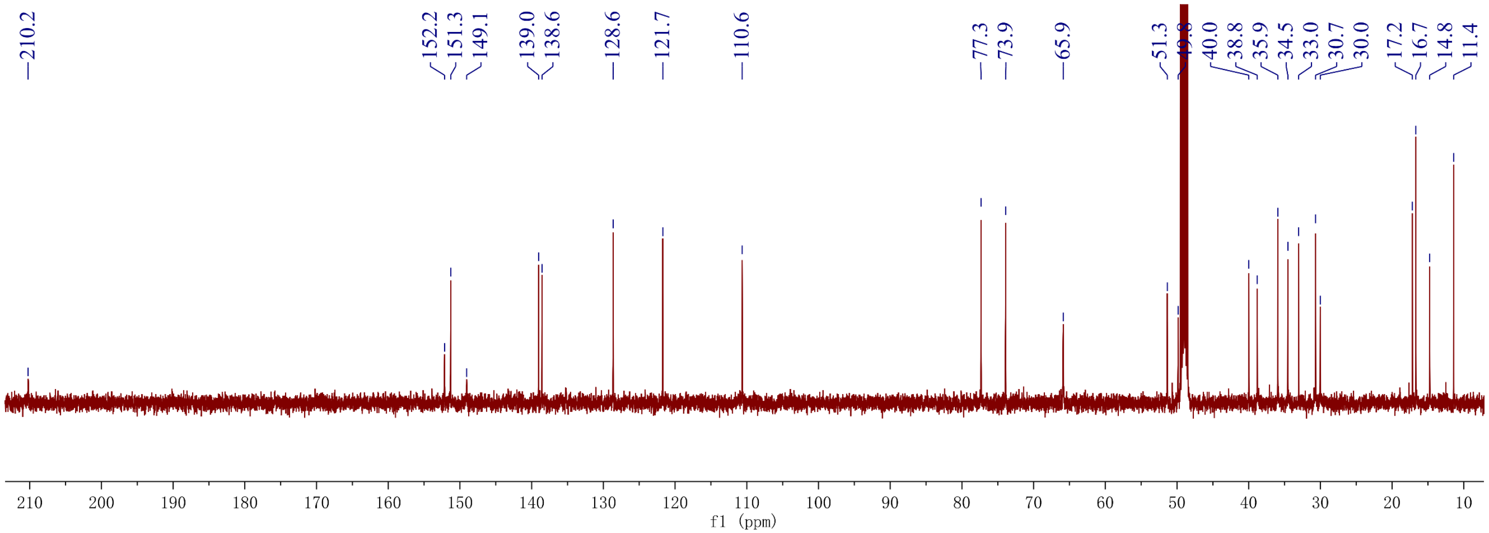

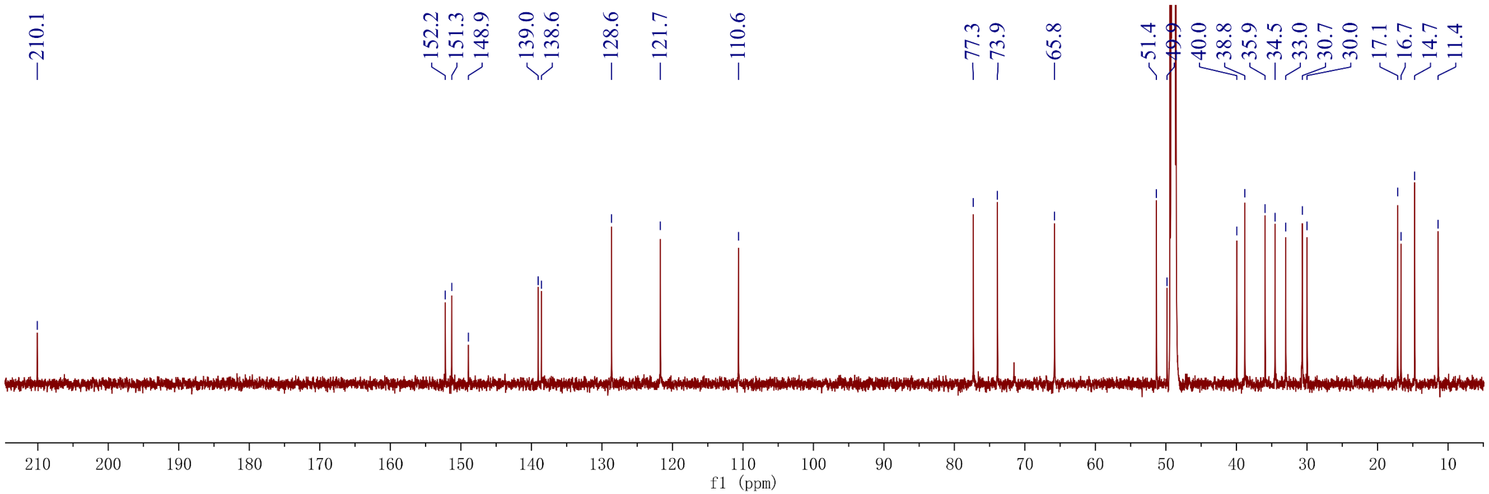

Figure S159. Comparison of ^{13}C NMR spectra of natural **5** (Methanol-d_4, upper) and synthetic **5** (Methanol-d_4, below).

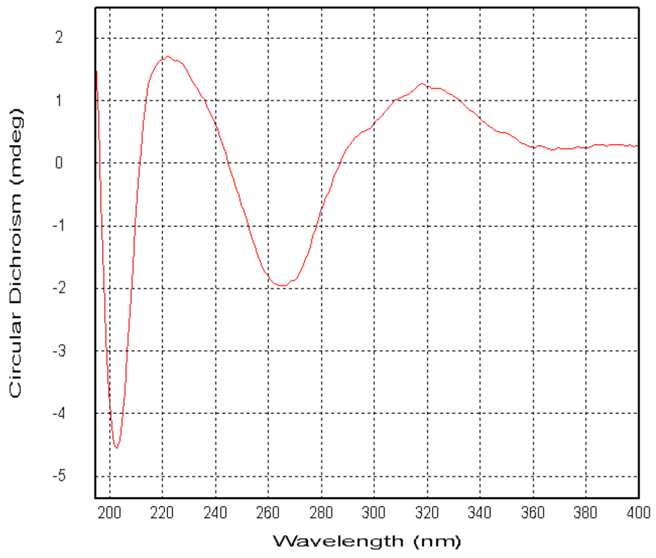

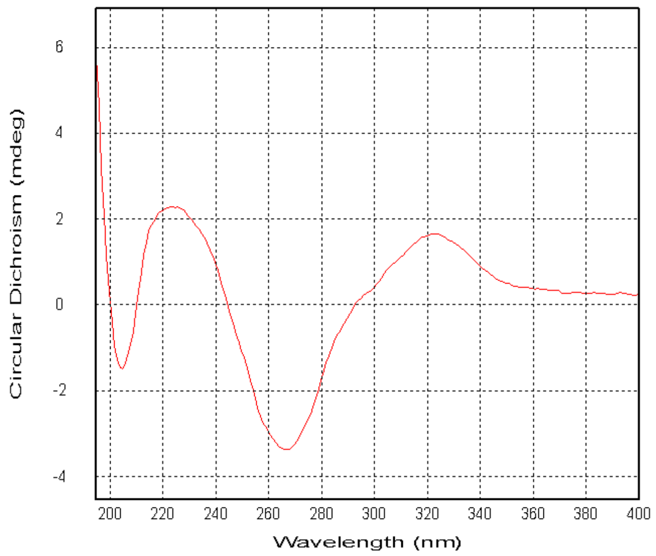

Figure S160. Comparison of ECD spectra of natural **5** (left) and synthetic **5** (right).

Rudolph Research Analytical

This sample was measured on an Autopol VI, Serial #91058
Manufactured by Rudolph Research Analytical, Hackettstown, NJ, USA.

Measurement Date : Friday, 28-MAY-2021

Set Temperature : OFF

Time Delay : Disabled

Delay between Measurement : Disabled

n	Average	Std.Dev.	% RSD	Maximum	Minimum
5	29.26	0.87	2.97	30.29	28.29

S.No	Sample ID	Time	Result	Scale	OR °Arc	WLG.nm	Lg.mm	Conc.g/100ml	Temp.
1	SHSJ46	10:27:07 AM	30.29	SR	0.0212	589	100.00	0.070	25.1
2	SHSJ46	10:27:16 AM	30.00	SR	0.0210	589	100.00	0.070	25.1
3	SHSJ46	10:27:24 AM	29.14	SR	0.0204	589	100.00	0.070	25.1
4	SHSJ46	10:27:32 AM	28.57	SR	0.0200	589	100.00	0.070	25.1
5	SHSJ46	10:27:40 AM	28.29	SR	0.0198	589	100.00	0.070	25.1

Rudolph Research Analytical

This sample was measured on an Autopol VI, Serial #91058
Manufactured by Rudolph Research Analytical, Hackettstown, NJ, USA.

Measurement Date : Tuesday, 02-JAN-2024

Set Temperature : 20.0

Time Delay : Disabled

Delay between Measurement : Disabled

n	Average	Std.Dev.	% RSD	Maximum	Minimum
5	18.18	0.00	0.00	18.18	18.18

S.No	Sample ID	Time	Result	Scale	OR °Arc	WLG.nm	Lg.mm	Conc.g/100ml	Temp.
1	SHSJR26	10:59:26 AM	18.18	SR	0.020	589	100.00	0.110	20.3
2	SHSJR26	10:59:32 AM	18.18	SR	0.020	589	100.00	0.110	20.2
3	SHSJR26	10:59:38 AM	18.18	SR	0.020	589	100.00	0.110	20.2
4	SHSJR26	10:59:45 AM	18.18	SR	0.020	589	100.00	0.110	20.1
5	SHSJR26	10:59:53 AM	18.18	SR	0.020	589	100.00	0.110	20.1

Figure S161. Comparison of ORD reports of natural **5** (upper) and synthetic **5** (below).

User Spectra

Fragmentor Voltage	Collision Energy	Ionization Mode
85	0	ESI

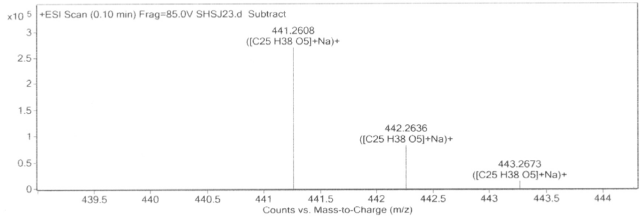

Peak List

m/z	z	Abund	Formula	Ion
62.0796		26226.71		
401.2682	1	80358.44		
402.2721	1	21978.86		
441.2608	1	270078.75	C25 H38 O5	(M+Na)+
442.2636	1	81982.8	C25 H38 O5	(M+Na)+
457.235	1	23741.88		
480.3497	1	38417.58		
859.5308	1	134767.95		
860.5338	1	70701.45		
861.5363	1	21257.1		

Formula Calculator Element Limits

Element	Min	Max
C	3	60
H	0	120
O	0	30

Formula Calculator Results

Formula	CalculatedMass	CalculatedMz	Mz	Diff. (mDa)	Diff. (ppm)	DBE
C25 H38 O5	418.2719	441.2611	441.2608	0.30	0.68	7.0000

--- End Of Report ---

User Spectra

Fragmentor Voltage	Collision Energy	Ionization Mode
165	30	ESI

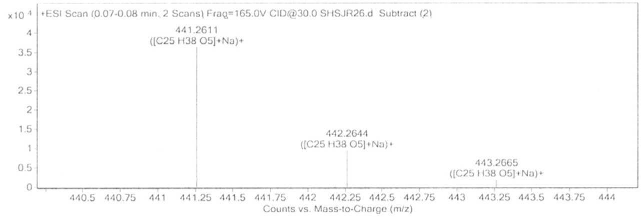

Peak List

m/z	z	Abund	Formula	Ion
81.0702	1	3212.22		
89.06	1	3799.69		
103.0395	1	5302.35		
119.0854	1	2771.7		
133.0994	1	2711.56		
147.1167	1	2810.18		
401.2686	1	6185.28		
441.2611	1	36368.43	C25 H38 O5	(M+Na)+
442.2644	1	9496.87	C25 H38 O5	(M+Na)+
859.5333	1	3357.79		

Formula Calculator Element Limits

Element	Min	Max
C	3	60
H	0	150
O	0	20
N	0	5

Formula Calculator Results

Formula	CalculatedMass	CalculatedMz	Mz	Diff. (mDa)	Diff. (ppm)	DBE
C25 H38 O5	418.2719	441.2611	441.2611	0.00	0.00	7.0000

--- End Of Report ---

Figure S162. Comparison of HRESIMS spectra of natural **5** (left) and synthetic **5** (right).

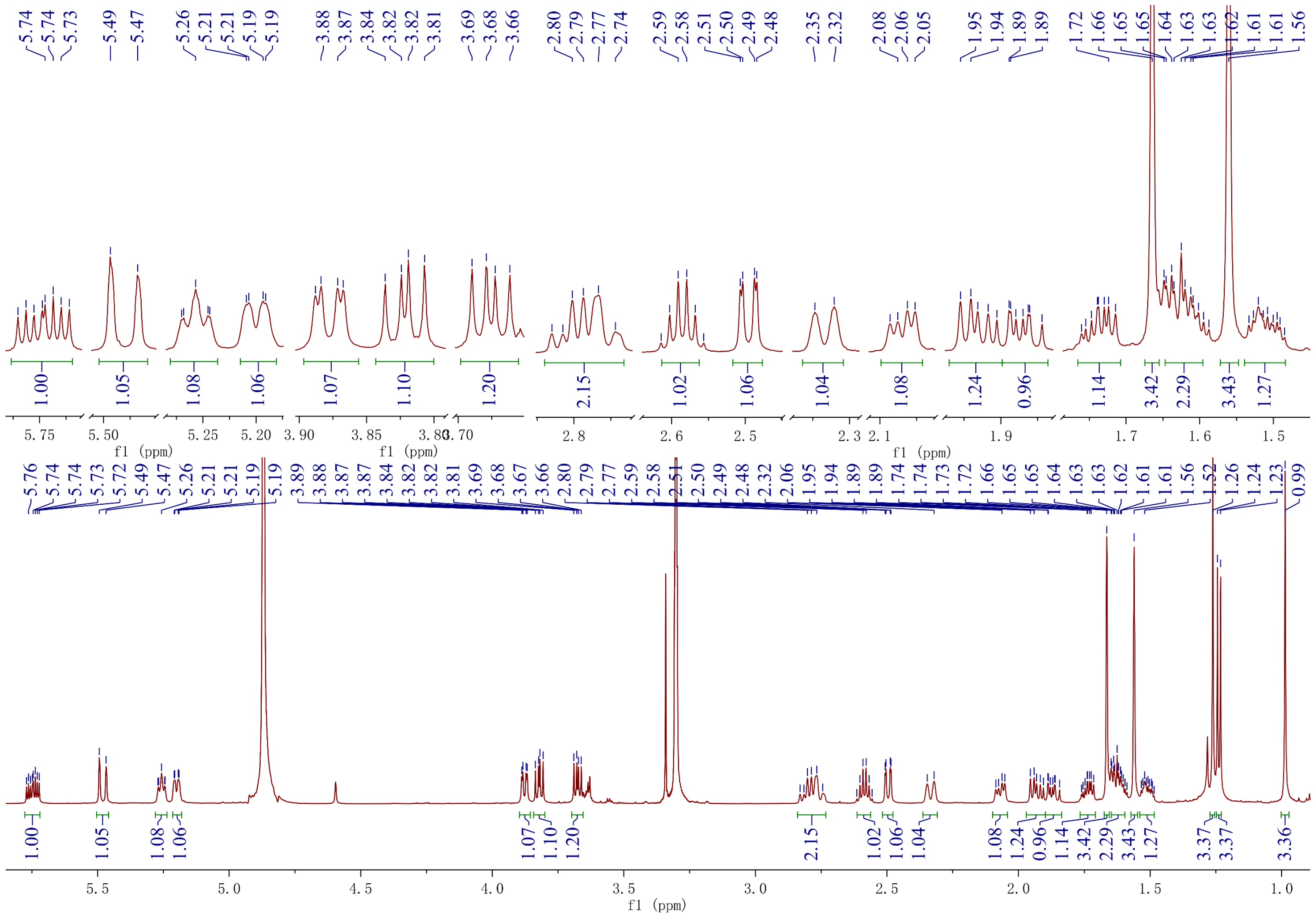

Figure S163. ^{1}H NMR spectrum of synthetic **6** (Methanol-d_4).

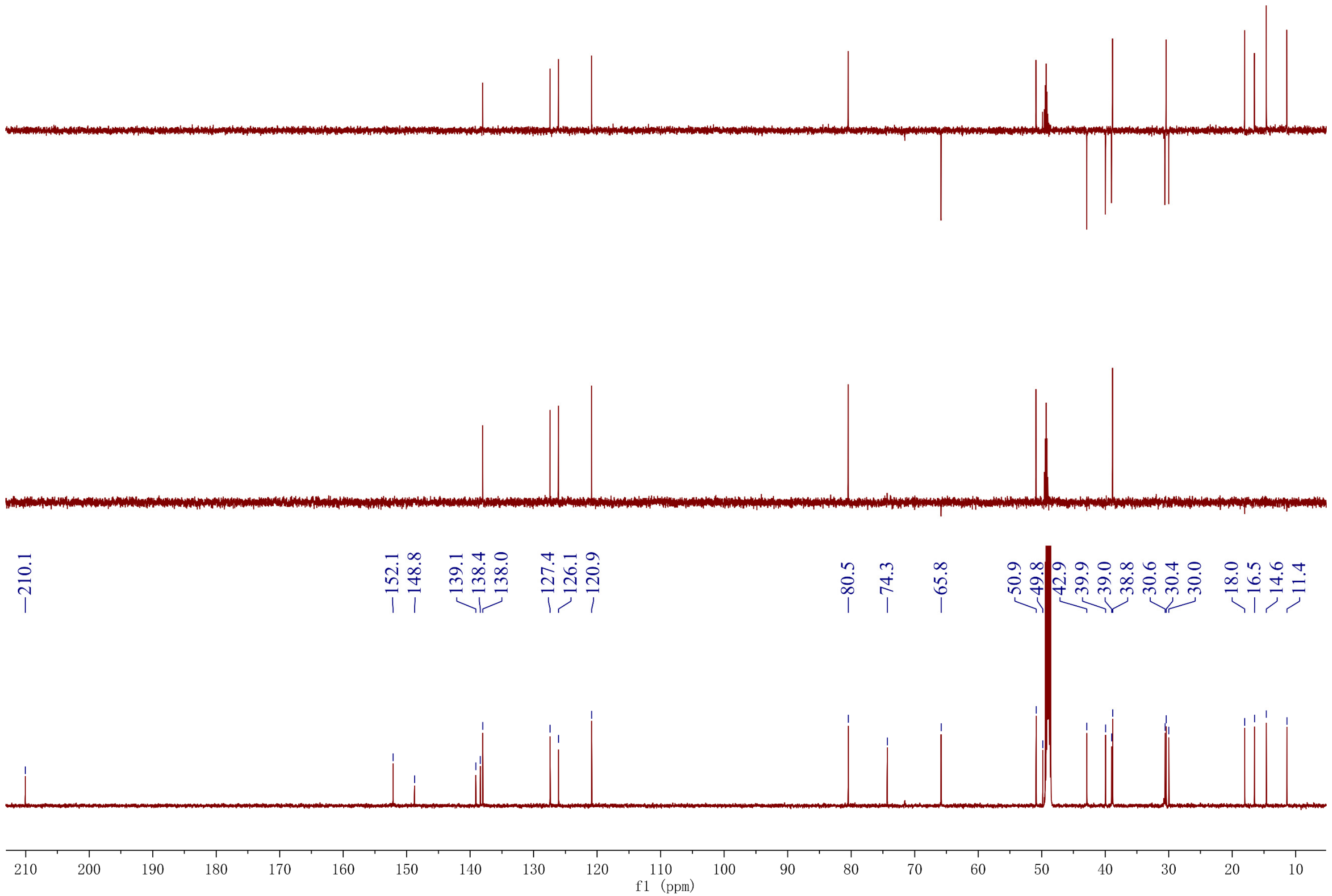

Figure S164. ^{13}C NMR and DEPT spectrum of synthetic **6** (Methanol-d_4).

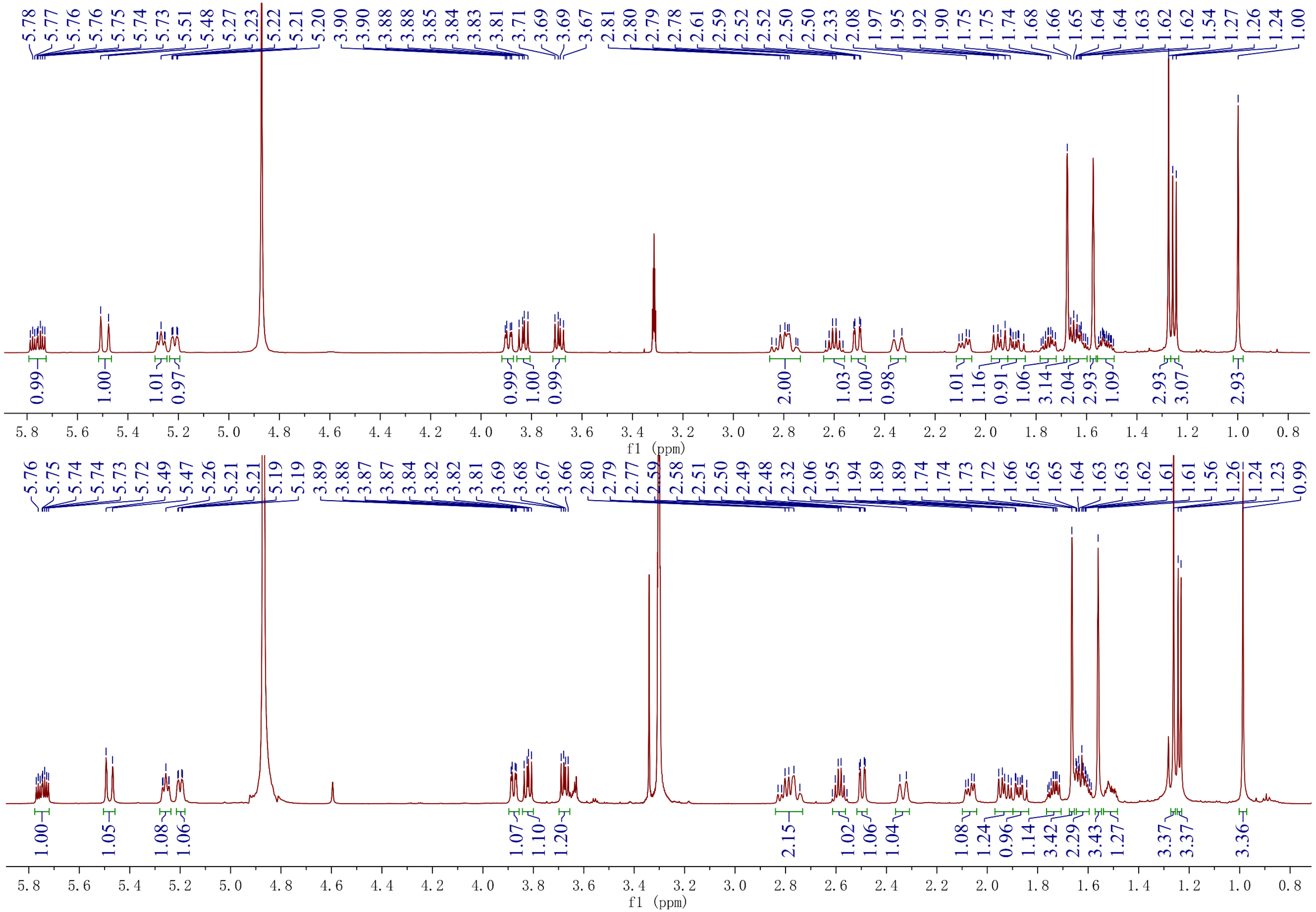

Figure S165. Comparison of ^{1}H NMR spectra of natural **6** (Methanol-d_4, upper) and synthetic **6** (Methanol-d_4, below).

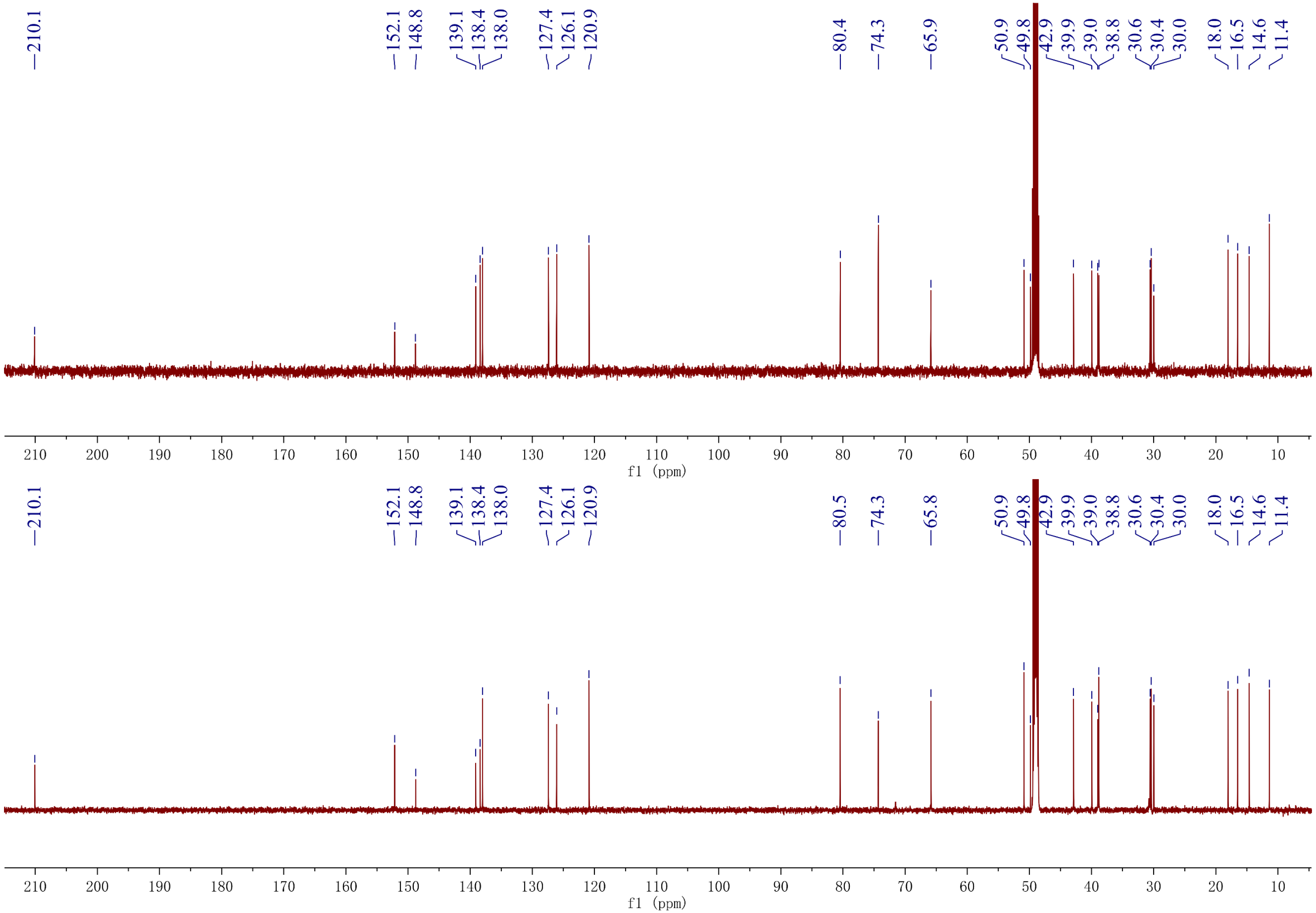

Figure S166. Comparison of ^{13}C NMR spectra of natural **6** (Methanol-d_4, upper) and synthetic **6** (Methanol-d_4, below).

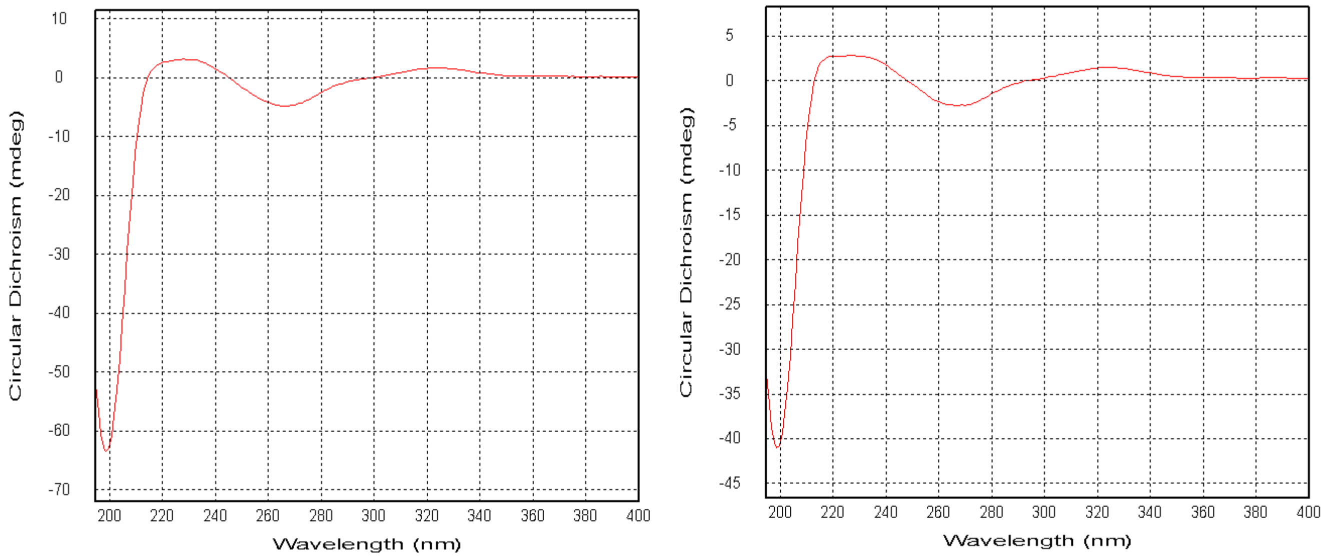

Figure S167. Comparison of ECD spectra of natural **6** (left) and synthetic **6** (right).

Rudolph Research Analytical

This sample was measured on an Autopol VI, Serial #91058
Manufactured by Rudolph Research Analytical, Hackettstown, NJ, USA.

Measurement Date : Friday, 28-MAY-2021

Set Temperature : OFF

Time Delay : Disabled

Delay between Measurement : Disabled

n	Average	Std.Dev.	% RSD	Maximum	Minimum
5	-31.07	0.34	-1.09	-30.87	-31.67

S.No	Sample ID	Time	Result	Scale	OR °Arc	WLG.nm	Lg.mm	Conc.g/100ml	Temp.
1	SHSJ22	11:10:50 AM	-30.87	SR	-0.0463	589	100.00	0.150	25.7
2	SHSJ22	11:10:58 AM	-30.87	SR	-0.0463	589	100.00	0.150	25.7
3	SHSJ22	11:11:06 AM	-31.00	SR	-0.0465	589	100.00	0.150	25.7
4	SHSJ22	11:11:14 AM	-30.93	SR	-0.0464	589	100.00	0.150	25.7
5	SHSJ22	11:11:23 AM	-31.67	SR	-0.0475	589	100.00	0.150	25.7

Rudolph Research Analytical

This sample was measured on an Autopol VI, Serial #91058
Manufactured by Rudolph Research Analytical, Hackettstown, NJ, USA.

Measurement Date : Tuesday, 02-JAN-2024

Set Temperature : 20.0

Time Delay : Disabled

Delay between Measurement : Disabled

n	Average	Std.Dev.	% RSD	Maximum	Minimum
5	-28.73	0.50	-1.74	-28.18	-29.09

S.No	Sample ID	Time	Result	Scale	OR °Arc	WLG.nm	Lg.mm	Conc.g/100ml	Temp.
1	SHSJR22	10:39:58 AM	-28.18	SR	-0.031	589	100.00	0.110	20.5
2	SHSJR22	10:40:05 AM	-29.09	SR	-0.032	589	100.00	0.110	20.4
3	SHSJR22	10:40:11 AM	-29.09	SR	-0.032	589	100.00	0.110	20.4
4	SHSJR22	10:40:17 AM	-28.18	SR	-0.031	589	100.00	0.110	20.3
5	SHSJR22	10:40:24 AM	-29.09	SR	-0.032	589	100.00	0.110	20.3

Figure S168. Comparison of ORD reports of natural **6** (upper) and synthetic **6** (below).

User Spectra

Fragmentor Voltage	Collision Energy	Ionization Mode
135	0	ESI

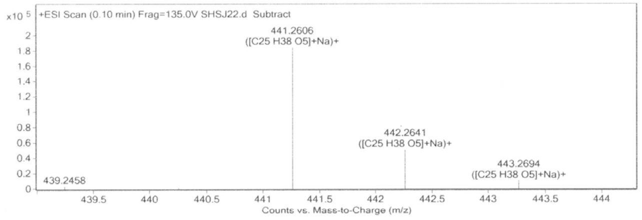

Peak List

m/z	z	Abund	Formula	Ion
383.2573	1	21521.98		
401.268	1	58534.31		
419.2788	1	149177.63		
420.2821	1	37957.27		
441.2606	1	184038.89	C25 H38 O5	(M+Na)+
442.2641	1	50619.09	C25 H38 O5	(M+Na)+
457.2354	1	21393.8		
859.5326	1	129515.99		
860.5353	1	70916.5		
861.5391	1	20052.4		

Formula Calculator Element Limits

Element	Min	Max
C	3	60
H	0	120
O	0	30

Formula Calculator Results

Formula	CalculatedMass	CalculatedMz	Mz	Diff. (mDa)	Diff. (ppm)	DBE
C25 H38 O5	418.2719	441.2611	441.2606	0.50	1.13	7.0000

--- End Of Report ---

User Spectra

Fragmentor Voltage	Collision Energy	Ionization Mode
135	0	ESI

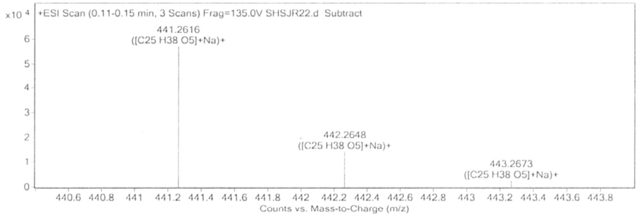

Peak List

m/z	z	Abund	Formula	Ion
383.2587	1	7489.95		
401.2687	1	27060.83		
402.2724	1	7447.99		
419.2795	1	42670.78		
420.283	1	12196.9		
441.2616	1	56995.06	C25 H38 O5	(M+Na)+
442.2648	1	14184.02	C25 H38 O5	(M+Na)+
457.2362	1	5534.85		
859.534	1	9737.29		
860.5373	1	5429.11		

Formula Calculator Element Limits

Element	Min	Max
C	3	60
H	0	150
O	0	20

Formula Calculator Results

Formula	CalculatedMass	CalculatedMz	Mz	Diff. (mDa)	Diff. (ppm)	DBE
C25 H38 O5	418.2719	441.2611	441.2616	-0.50	-1.13	7.0000

--- End Of Report ---

Figure S169. Comparison of HRESIMS spectra of natural **6** (left) and synthetic **6** (right).

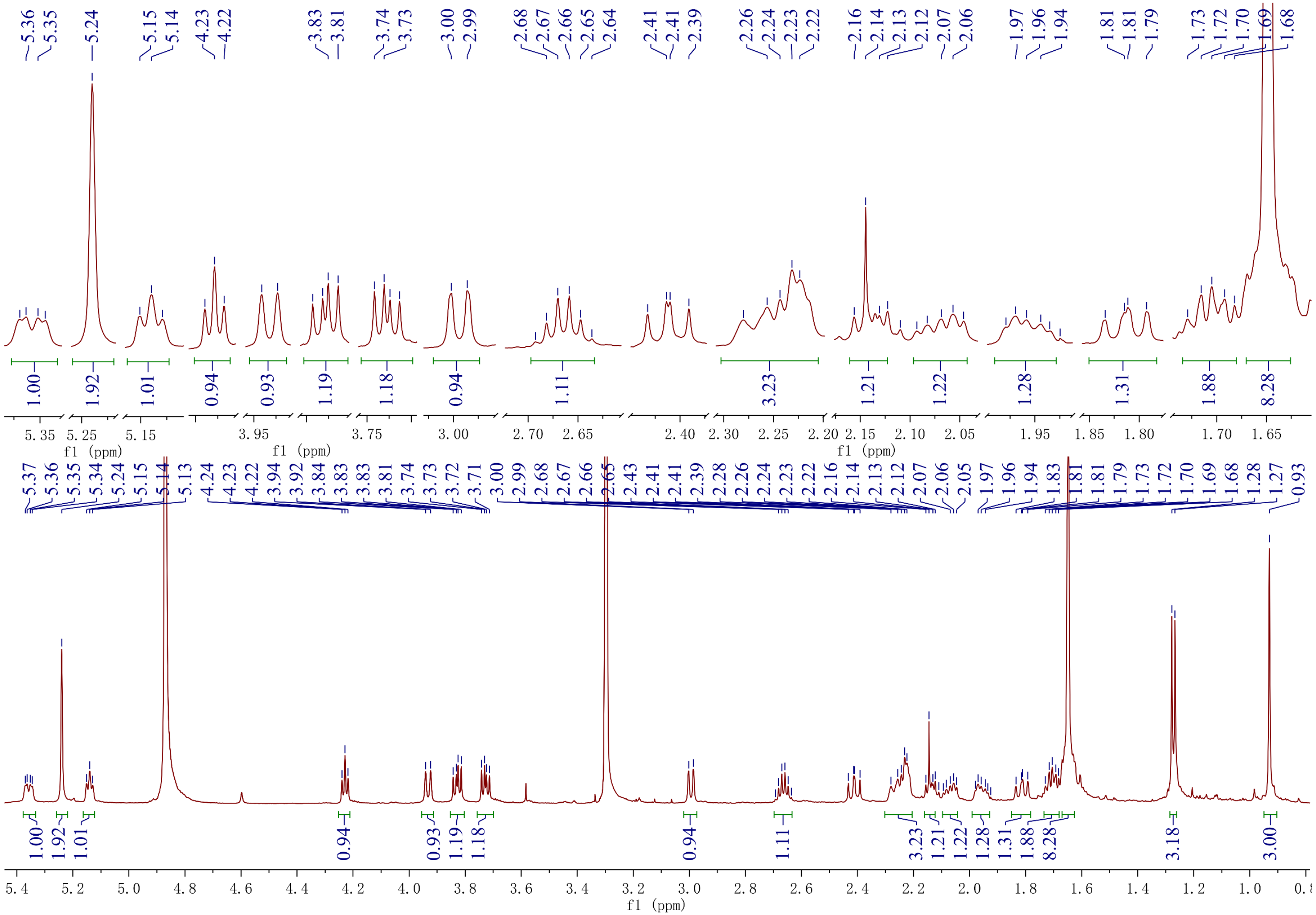

Figure S170. ^{1}H NMR spectrum of synthetic **9** (Methanol-d_4).

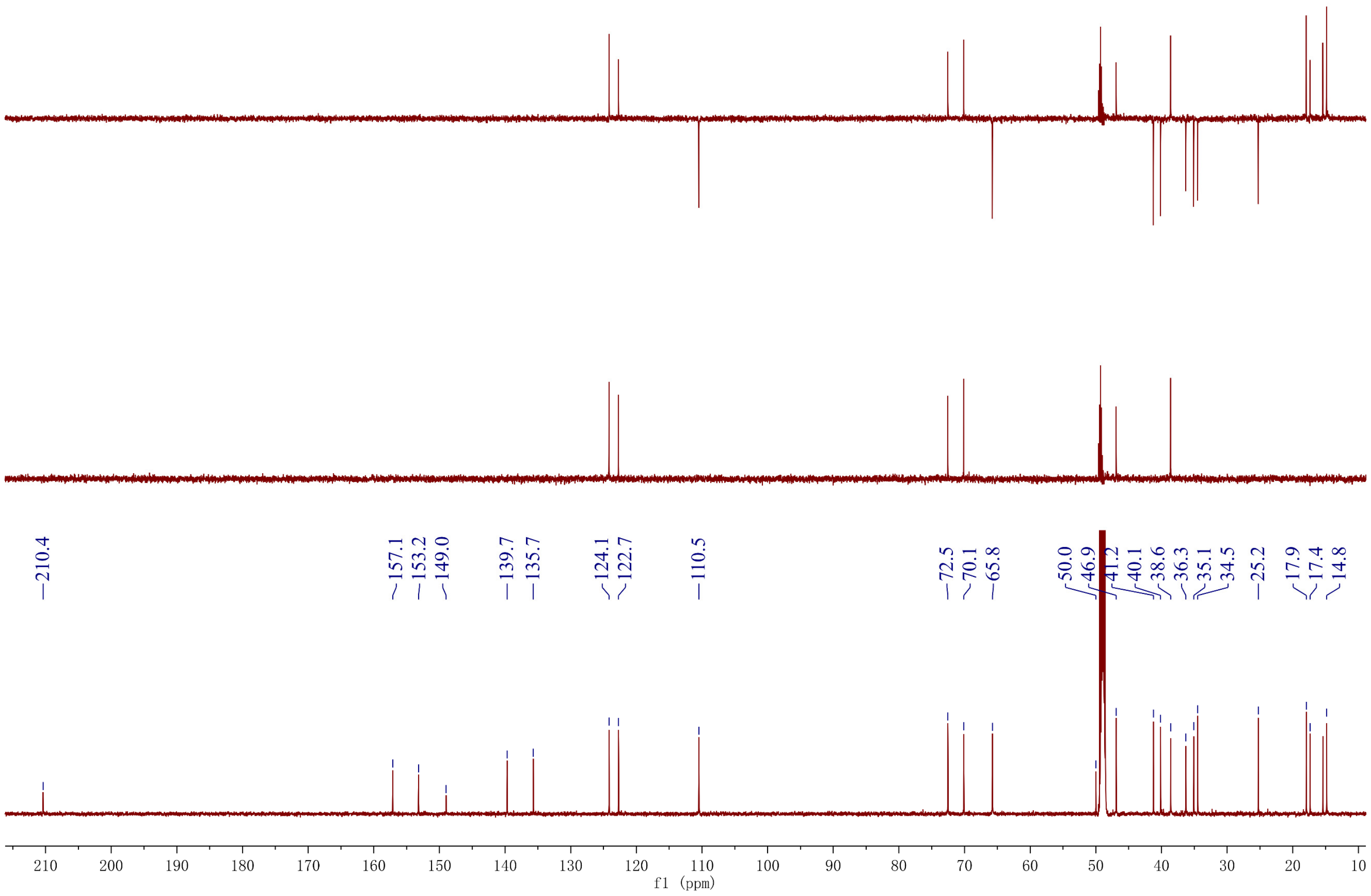

Figure S171. ^{13}C NMR and DEPT spectrum of synthetic **9** (Methanol-d_4).

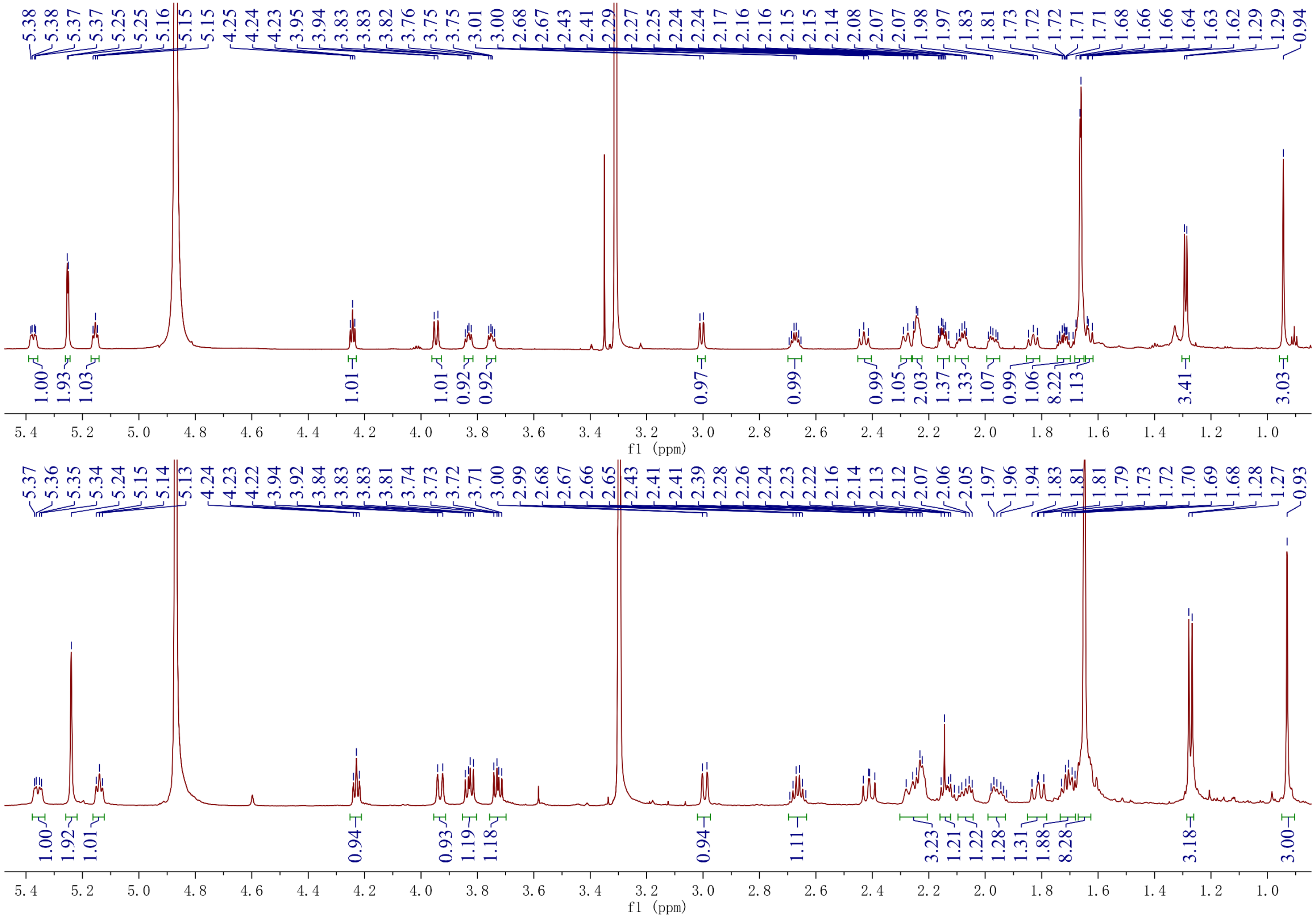

Figure S172. Comparison of ^{1}H NMR spectra of natural **9** (Methanol-d_4, upper) and synthetic **9** (Methanol-d_4, below).

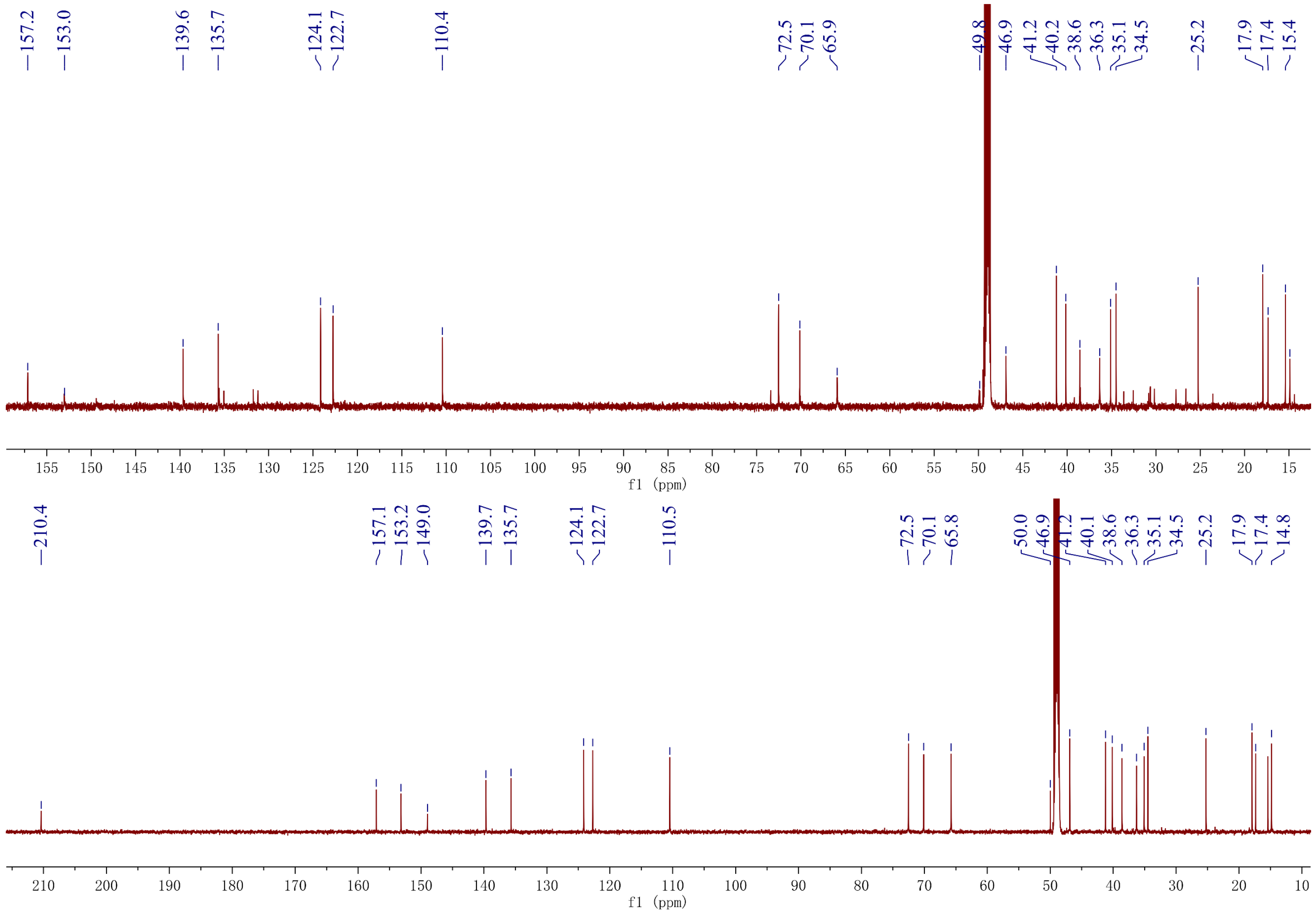

Figure S173. Comparison of ^{13}C NMR spectra of natural **9** (Methanol-d_4, upper) and synthetic **9** (Methanol-d_4, below).

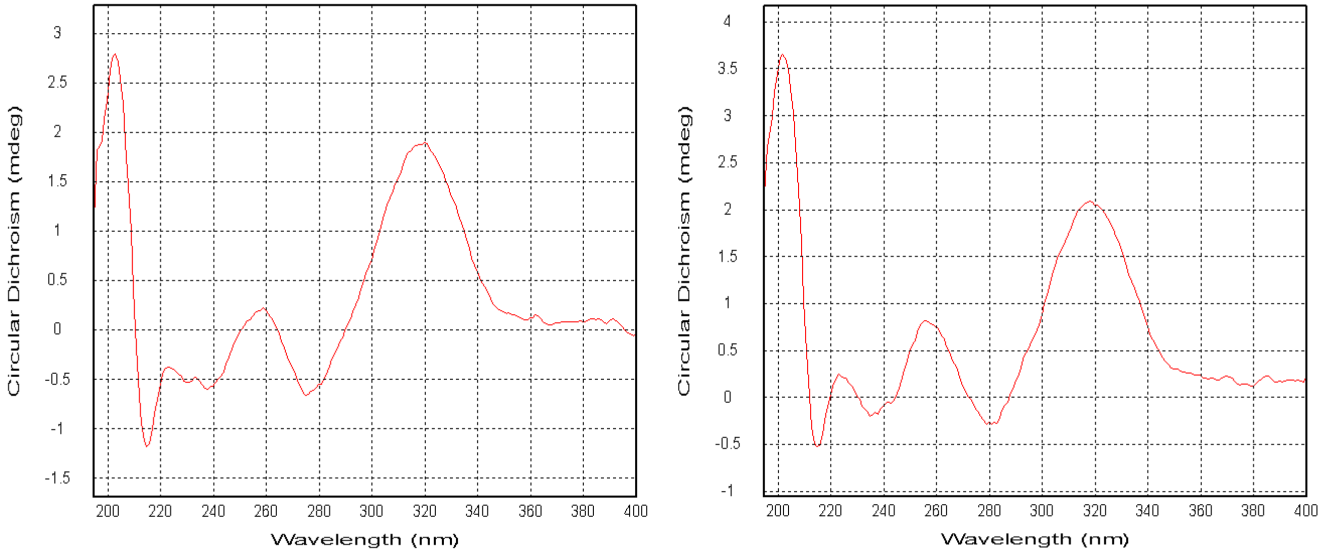

Figure S174. Comparison of ECD spectra of natural **9** (left) and synthetic **9** (right).

Rudolph Research Analytical

This sample was measured on an Autopol VI, Serial #91058
Manufactured by Rudolph Research Analytical, Hackettstown, NJ, USA.

Measurement Date : Friday, 28-MAY-2021

Set Temperature : OFF

Time Delay : Disabled

Delay between Measurement : Disabled

n	Average	Std.Dev.	% RSD	Maximum	Minimum
5	90.74	0.52	0.57	91.43	90.29

S.No	Sample ID	Time	Result	Scale	OR °Arc	WLG.nm	Lg.mm	Conc.g/100ml	Temp.
1	SHSJ32	11:03:24 AM	90.29	SR	0.0632	589	100.00	0.070	25.6
2	SHSJ32	11:03:33 AM	90.29	SR	0.0632	589	100.00	0.070	25.6
3	SHSJ32	11:03:41 AM	91.14	SR	0.0638	589	100.00	0.070	25.6
4	SHSJ32	11:03:49 AM	90.57	SR	0.0634	589	100.00	0.070	25.6
5	SHSJ32	11:03:57 AM	91.43	SR	0.0640	589	100.00	0.070	25.6

Rudolph Research Analytical

This sample was measured on an Autopol VI, Serial #91058
Manufactured by Rudolph Research Analytical, Hackettstown, NJ, USA.

Measurement Date : Tuesday, 02-JAN-2024

Set Temperature : 20.0

Time Delay : Disabled

Delay between Measurement : Disabled

n	Average	Std.Dev.	% RSD	Maximum	Minimum
5	47.60	0.89	1.86	48.00	46.00

S.No	Sample ID	Time	Result	Scale	OR °Arc	WLG.nm	Lg.mm	Conc.g/100ml	Temp.
1	SHSJR9	09:52:13 AM	48.00	SR	0.024	589	100.00	0.050	20.2
2	SHSJR9	09:52:19 AM	46.00	SR	0.023	589	100.00	0.050	20.2
3	SHSJR9	09:52:25 AM	48.00	SR	0.024	589	100.00	0.050	20.2
4	SHSJR9	09:52:32 AM	48.00	SR	0.024	589	100.00	0.050	20.2
5	SHSJR9	09:52:38 AM	48.00	SR	0.024	589	100.00	0.050	20.1

Figure S175. Comparison of ORD reports of natural **9** (upper) and synthetic **9** (below).

User Spectra

Fragmentor Voltage	Collision Energy	Ionization Mode
85	0	ESI

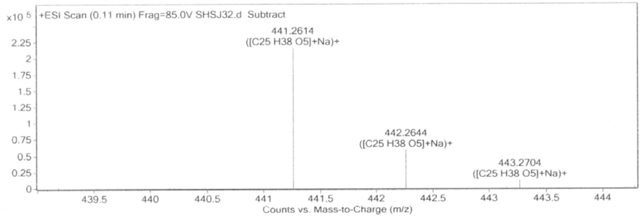

Peak List

m/z	z	Abund	Formula	Ion
102.1278	1	19902.18		
419.2791	1	66860.66		
420.2826	1	15081.68		
441.2614	1	216372.63	C25 H38 O5	(M+Na)+
442.2644	1	58581.28	C25 H38 O5	(M+Na)+
443.2704	1	12002.03	C25 H38 O5	(M+Na)+
457.237	1	21124.11		
859.5322	1	77402.36		
860.5356	1	41553.68		
861.5401	1	17929.89		

Formula Calculator Element Limits

Element	Min	Max
C	3	60
H	0	120
O	0	30

Formula Calculator Results

Formula	CalculatedMass	CalculatedMz	Mz	Diff. (mDa)	Diff. (ppm)	DBE
C25 H38 O5	418.2719	441.2611	441.2614	-0.30	-0.68	7.0000

--- End Of Report ---

User Spectra

Fragmentor Voltage	Collision Energy	Ionization Mode
165	30	ESI

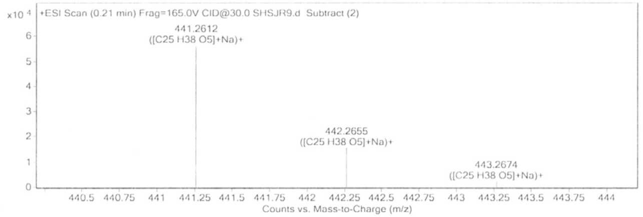

Peak List

m/z	z	Abund	Formula	Ion
81.0703	1	4629.02		
95.0856	1	4364.73		
107.0853	1	4457.47		
119.0856	1	5412.01		
121.1008	1	4981.26		
133.1014	1	5692.29		
147.1161	1	4914.29		
441.2612	1	55991.64	C25 H38 O5	(M+Na)+
442.2655	1	15512.71	C25 H38 O5	(M+Na)+
457.2544	1	6163.86		

Formula Calculator Element Limits

Element	Min	Max
C	3	60
H	0	150
O	0	20
N	0	5

Formula Calculator Results

Formula	CalculatedMass	CalculatedMz	Mz	Diff. (mDa)	Diff. (ppm)	DBE
C25 H38 O5	418.2719	441.2611	441.2612	-0.10	-0.23	7.0000

--- End Of Report ---

Figure S176. Comparison of HRESIMS spectra of natural **9** (left) and synthetic **9** (right).

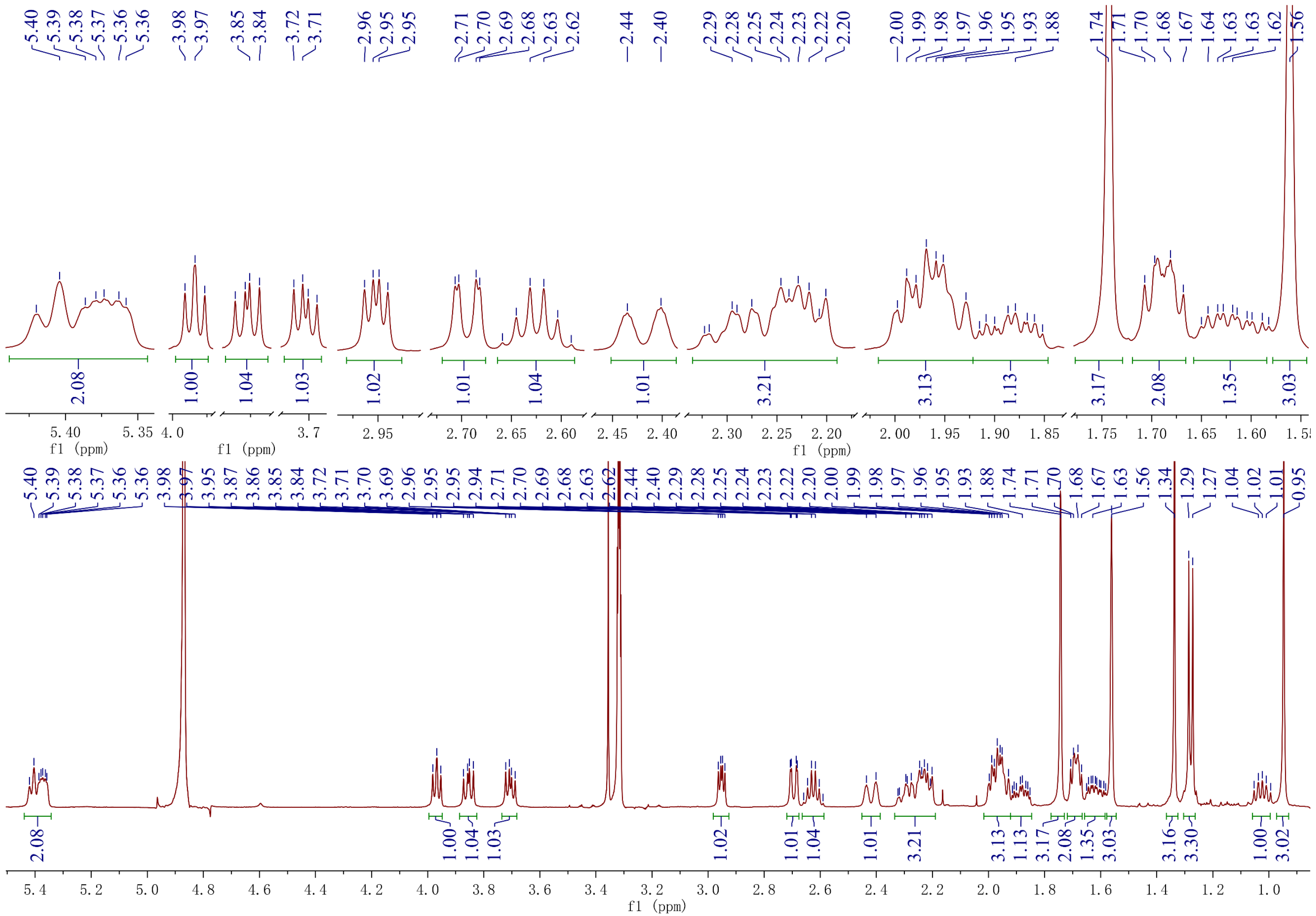

Figure S177. ^{1}H NMR spectrum of synthetic **11** (Methanol-d_4).

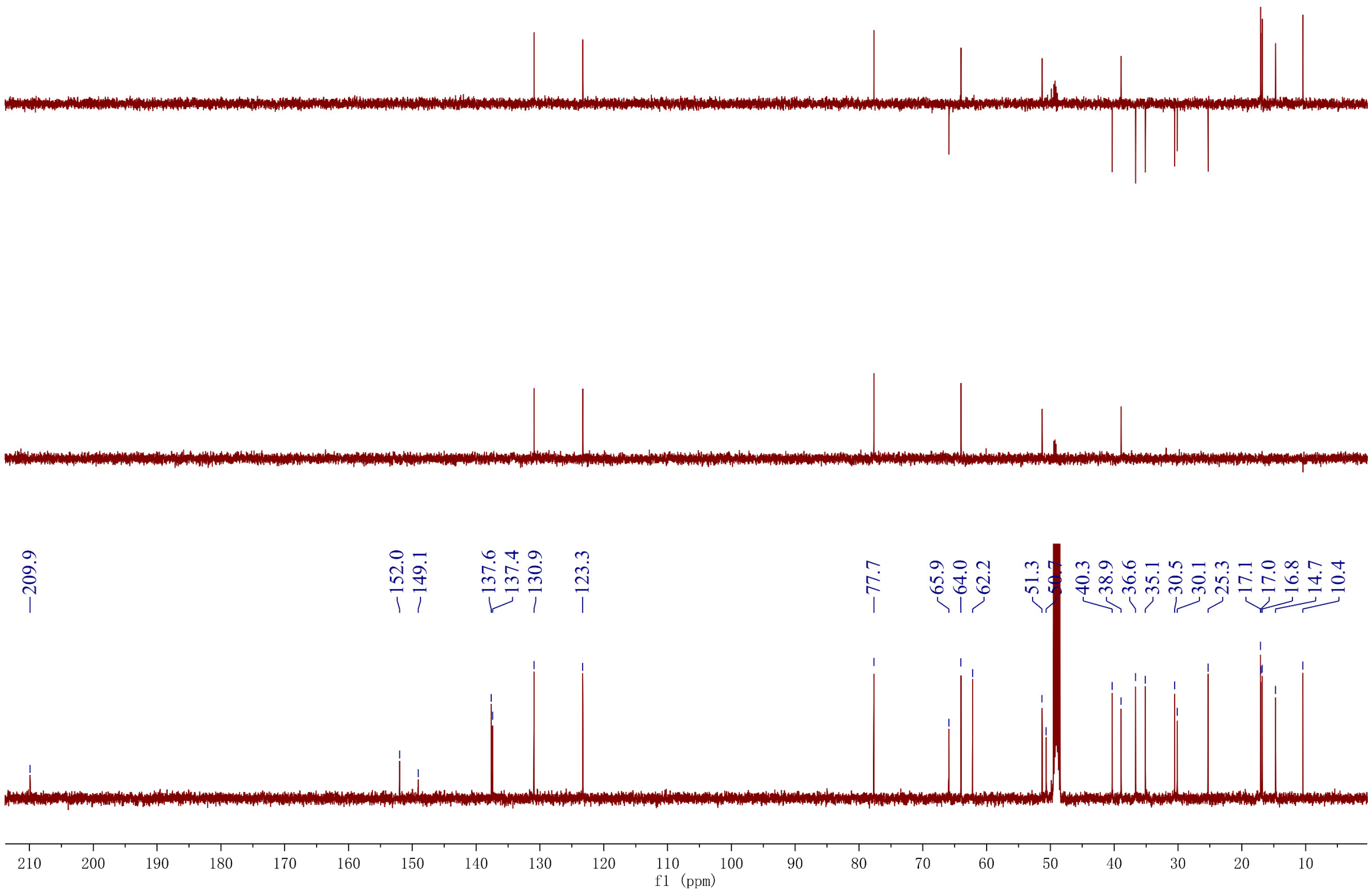

Figure S178. ^{13}C NMR and DEPT spectrum of synthetic **11** (Methanol-d_4).

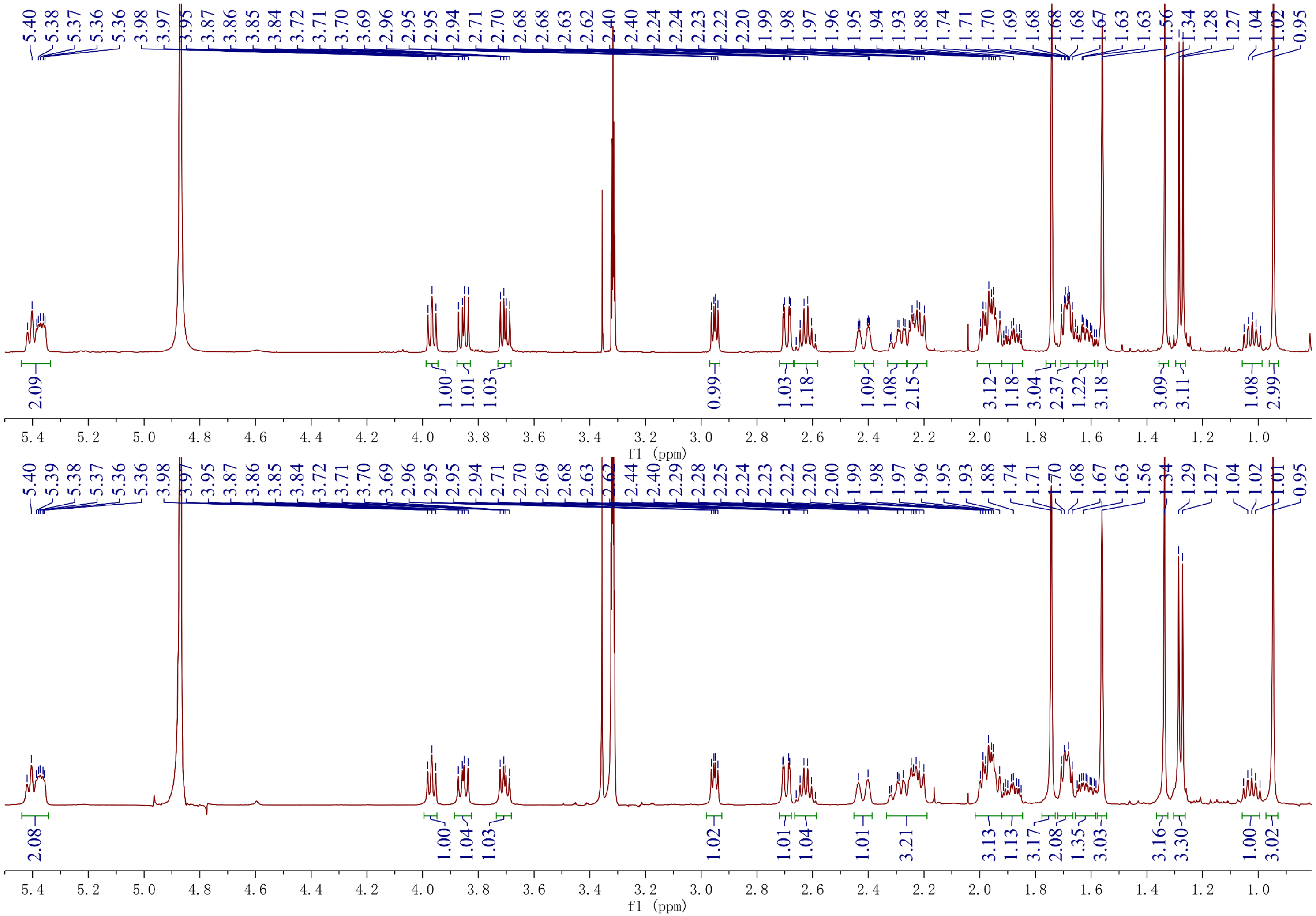

Figure S179. Comparison of ^{1}H NMR spectra of natural **11** (Methanol-d_4, upper) and synthetic **11** (Methanol-d_4, below).

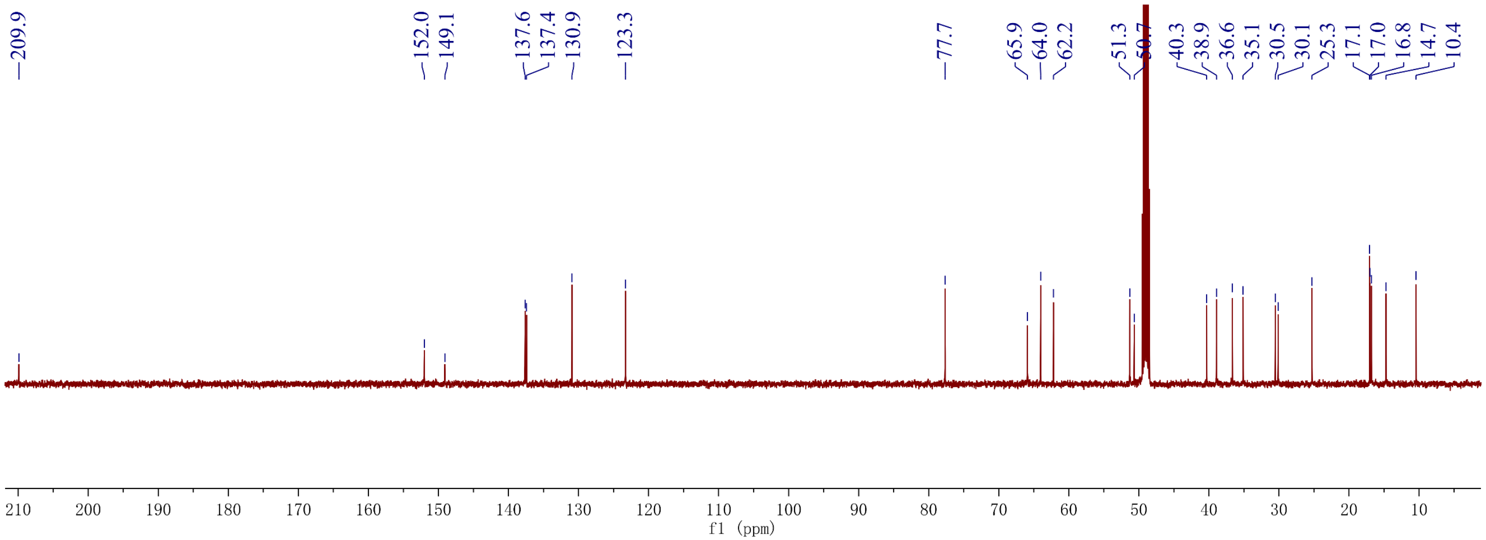

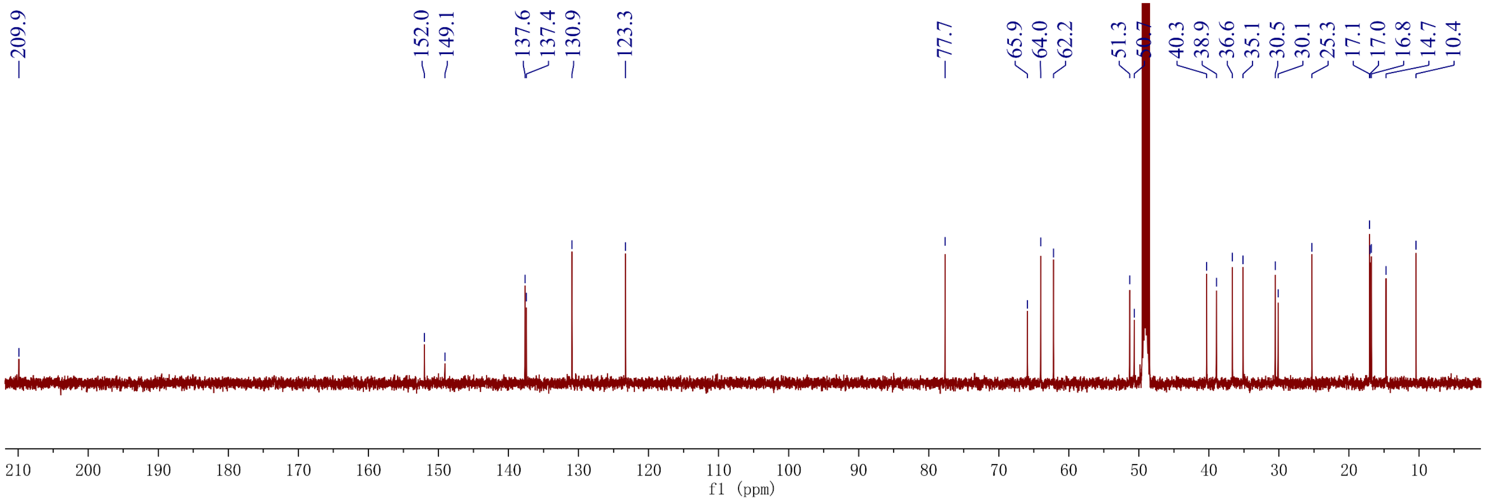

Figure S180. Comparison of ^{13}C NMR spectra of natural **11** (Methanol-d_4, upper) and synthetic **11** (Methanol-d_4, below).

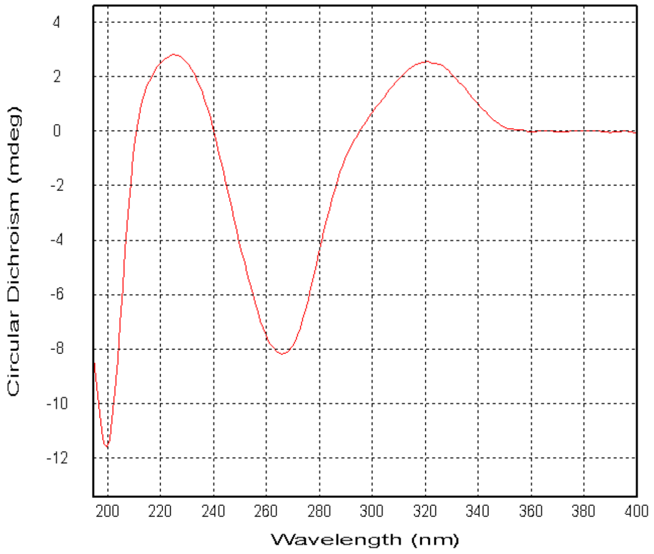

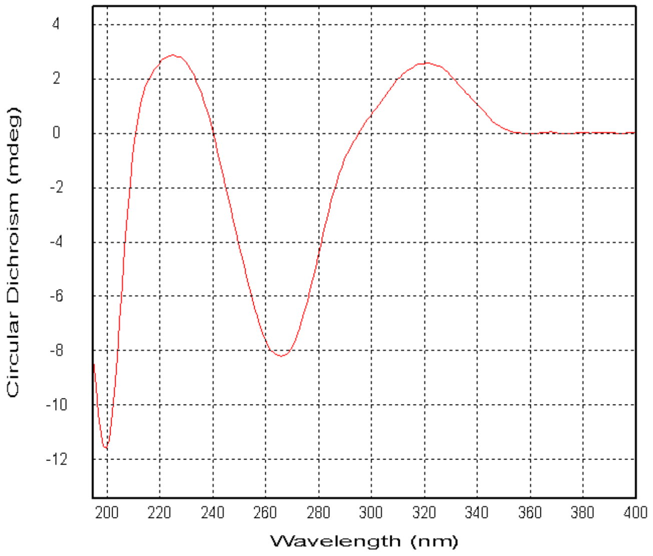

Figure S181. Comparison of ECD spectra of natural **11** (left) and synthetic **11** (right).

Rudolph Research Analytical

This sample was measured on an Autopol VI, Serial #91058
Manufactured by Rudolph Research Analytical, Hackettstown, NJ, USA.

Measurement Date : Monday, 21-APR-2025

Set Temperature : OFF

Time Delay : Disabled

Delay between Measurement : Disabled

n	Average	Std.Dev.	% RSD	Maximum	Minimum
5	-3.62	0.06	-1.65	-3.56	-3.70

S.No	Sample ID	Time	Result	Scale	OR °Arc	WLG.nm	Lg.mm	Conc.g/100ml	Temp.
1	SHSJ25	01:03:20 PM	-3.70	SR	-0.0054	589	100.00	0.146	27.1
2	SHSJ25	01:03:28 PM	-3.63	SR	-0.0053	589	100.00	0.146	27.1
3	SHSJ25	01:03:37 PM	-3.63	SR	-0.0053	589	100.00	0.146	27.1
4	SHSJ25	01:03:45 PM	-3.56	SR	-0.0052	589	100.00	0.146	27.1
5	SHSJ25	01:03:53 PM	-3.56	SR	-0.0052	589	100.00	0.146	27.1

Rudolph Research Analytical

This sample was measured on an Autopol VI, Serial #91058
Manufactured by Rudolph Research Analytical, Hackettstown, NJ, USA.

Measurement Date : Monday, 21-APR-2025

Set Temperature : OFF

Time Delay : Disabled

Delay between Measurement : Disabled

n	Average	Std.Dev.	% RSD	Maximum	Minimum
5	-3.19	0.39	-12.22	-2.62	-3.49

S.No	Sample ID	Time	Result	Scale	OR °Arc	WLG.nm	Lg.mm	Conc.g/100ml	Temp.
1	SHSJR4	01:12:18 PM	-3.49	SR	-0.0044	589	100.00	0.126	27.3
2	SHSJR4	01:12:27 PM	-2.62	SR	-0.0033	589	100.00	0.126	27.3
3	SHSJR4	01:12:35 PM	-3.49	SR	-0.0044	589	100.00	0.126	27.2
4	SHSJR4	01:12:43 PM	-2.94	SR	-0.0037	589	100.00	0.126	27.2
5	SHSJR4	01:12:52 PM	-3.41	SR	-0.0043	589	100.00	0.126	27.2

Figure S182. Comparison of ORD reports of natural **11** (upper) and synthetic **11** (below).

User Spectra

Fragmentor Voltage	Collision Energy	Ionization Mode
135	0	ESI

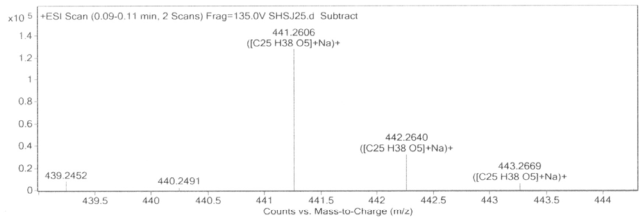

Peak List

m/z	z	Abund	Formula	Ion
401.268	1	39193.29		
419.2789	1	137111.34		
420.2817	1	36510.78		
441.2606	1	127678.91	C25 H38 O5	(M+Na)+
442.264	1	32169.09	C25 H38 O5	(M+Na)+
473.2499	1	19409.72		
857.5163	1	17778.36		
859.5325	1	125425.85		
860.5358	1	66198.67		
861.538	1	18883.01		

Formula Calculator Element Limits

Element	Min	Max
C	3	60
H	0	120
O	0	30

Formula Calculator Results

Formula	CalculatedMass	CalculatedMz	Mz	Diff. (mDa)	Diff. (ppm)	DBE
C25 H38 O5	418.2719	441.2611	441.2606	0.50	1.13	7.0000

--- End Of Report ---

User Spectra

Fragmentor Voltage	Collision Energy	Ionization Mode
135	0	ESI

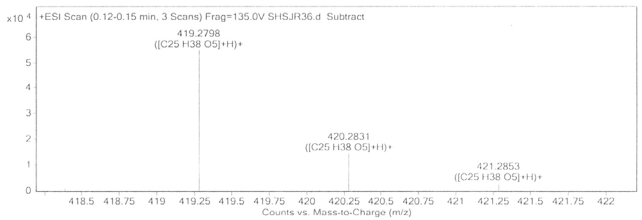

Peak List

m/z	z	Abund	Formula	Ion
205.6334	1	7357.01		
249.6311	1	7286.97		
353.2669	1	9215.78		
401.2674	1	18298.86		
415.2383	1	11287.16		
419.2798	1	54882.88	C25 H38 O5	(M+H)+
420.2831	1	14506.82	C25 H38 O5	(M+H)+
441.2614	1	20276.02		
443.2676	1	7592.56		
544.2586	1	7061.76		

Formula Calculator Element Limits

Element	Min	Max
C	3	60
H	0	150
O	0	20

Formula Calculator Results

Formula	CalculatedMass	CalculatedMz	Mz	Diff. (mDa)	Diff. (ppm)	DBE
C25 H38 O5	418.2719	419.2792	419.2798	-0.60	-1.43	7.0000

--- End Of Report ---

Figure S183. Comparison of HRESIMS spectra of natural **11** (left) and synthetic **11** (right).

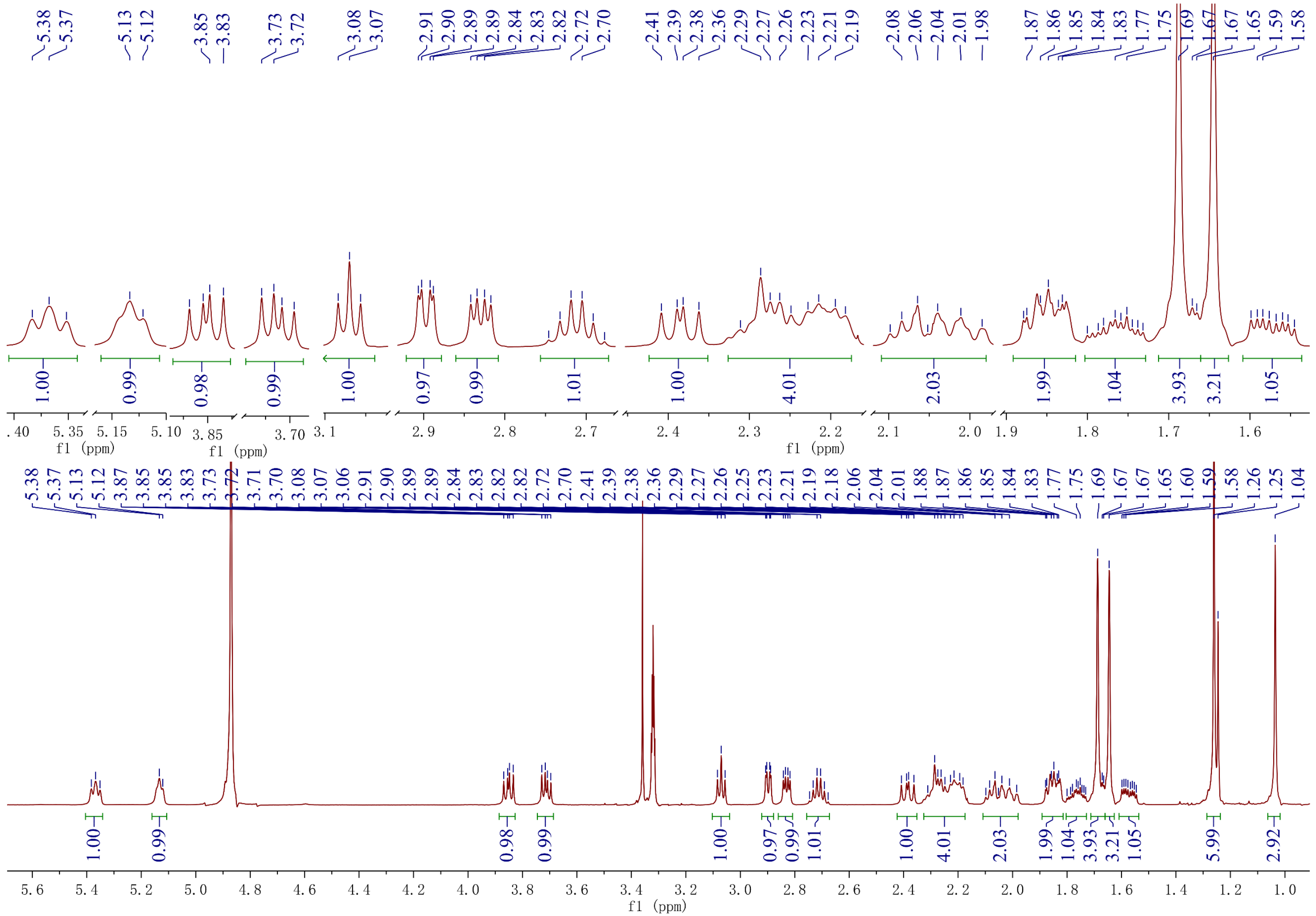

Figure S184. ^{1}H NMR spectrum of synthetic **12** (Methanol-d_4).

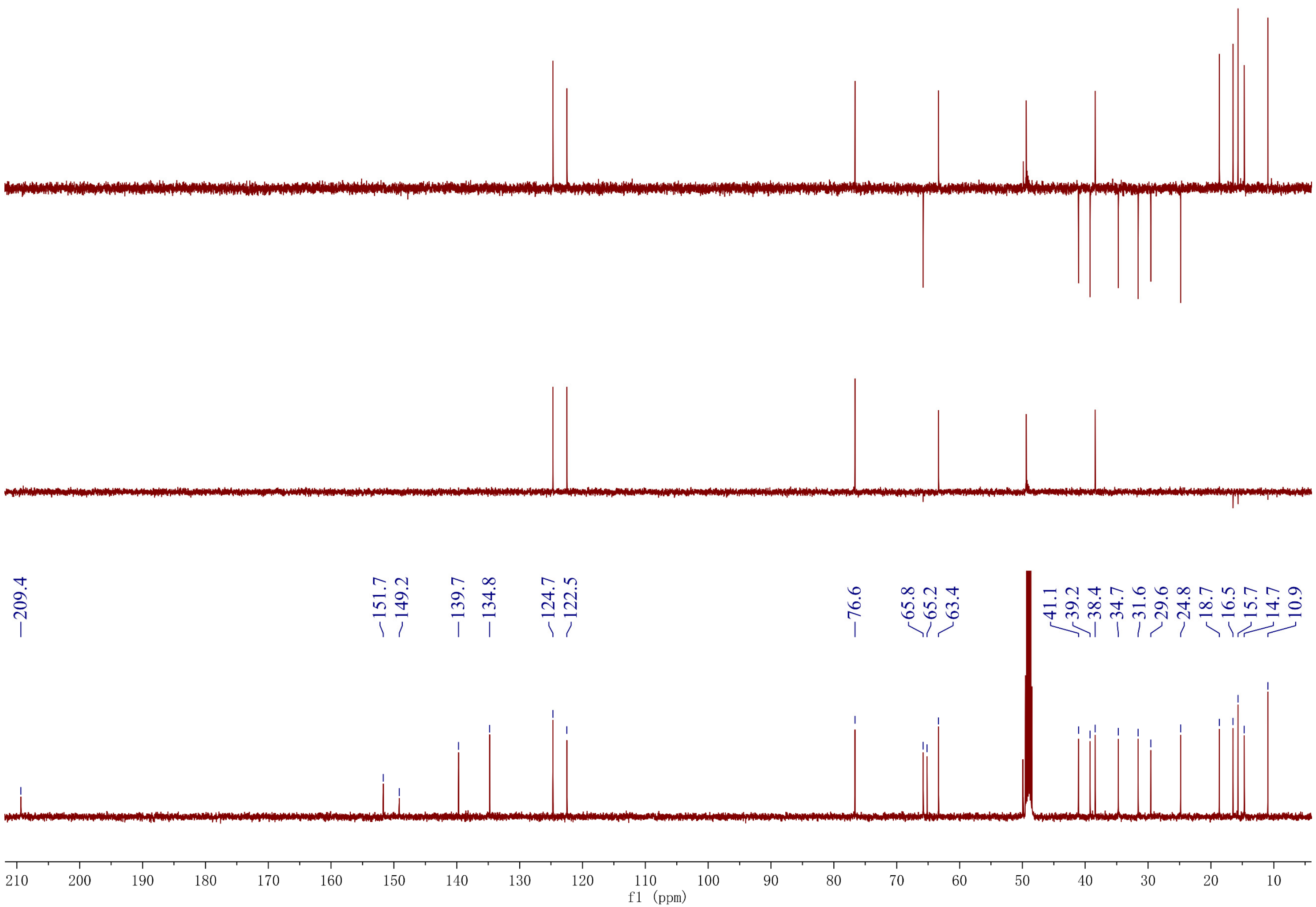

Figure S185. ^{13}C NMR and DEPT spectrum of synthetic **12** (Methanol-d_4).

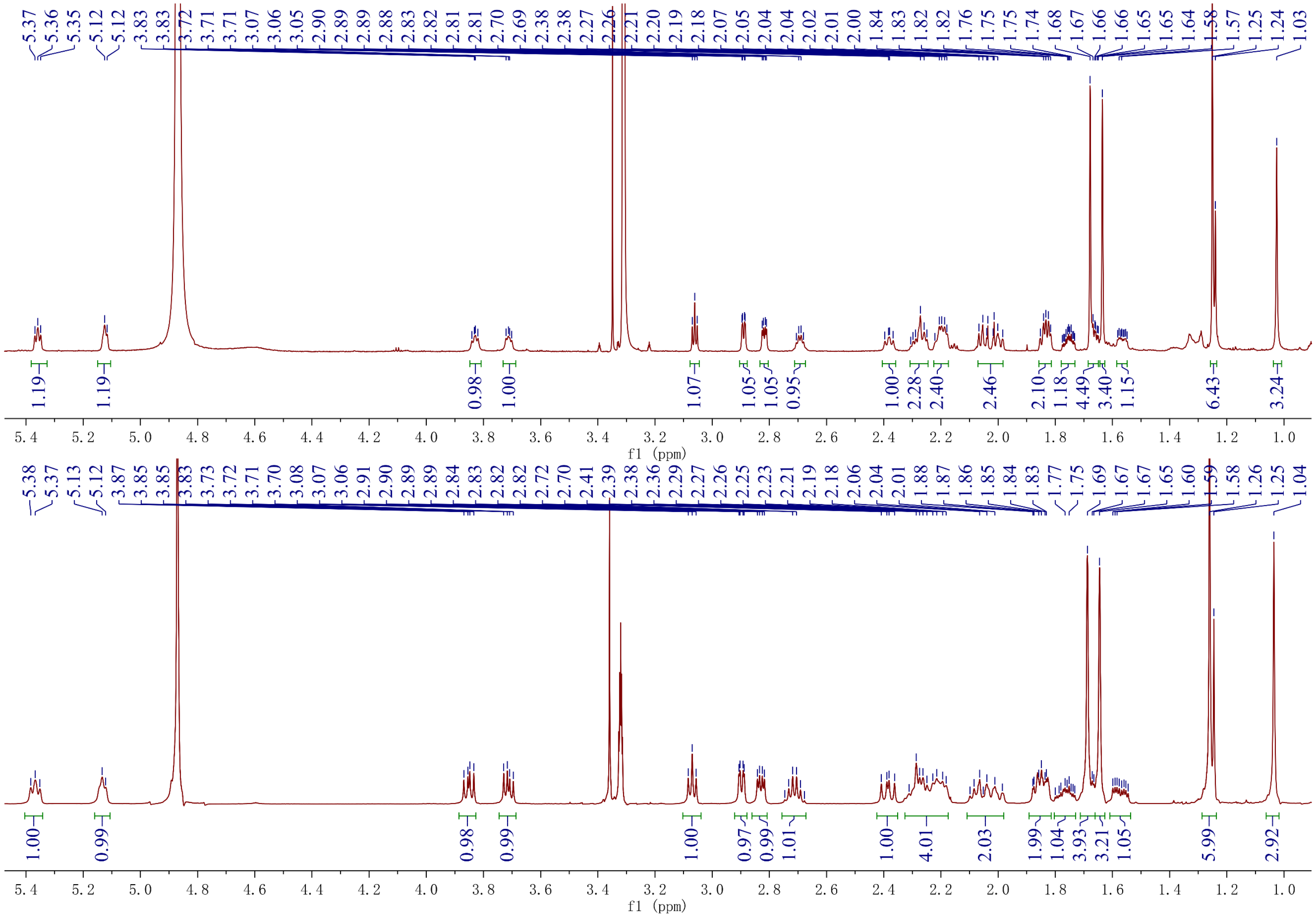

Figure S186. Comparison of ^{1}H NMR spectra of natural **12** (Methanol-d_4, upper) and synthetic **12** (Methanol-d_4, below).

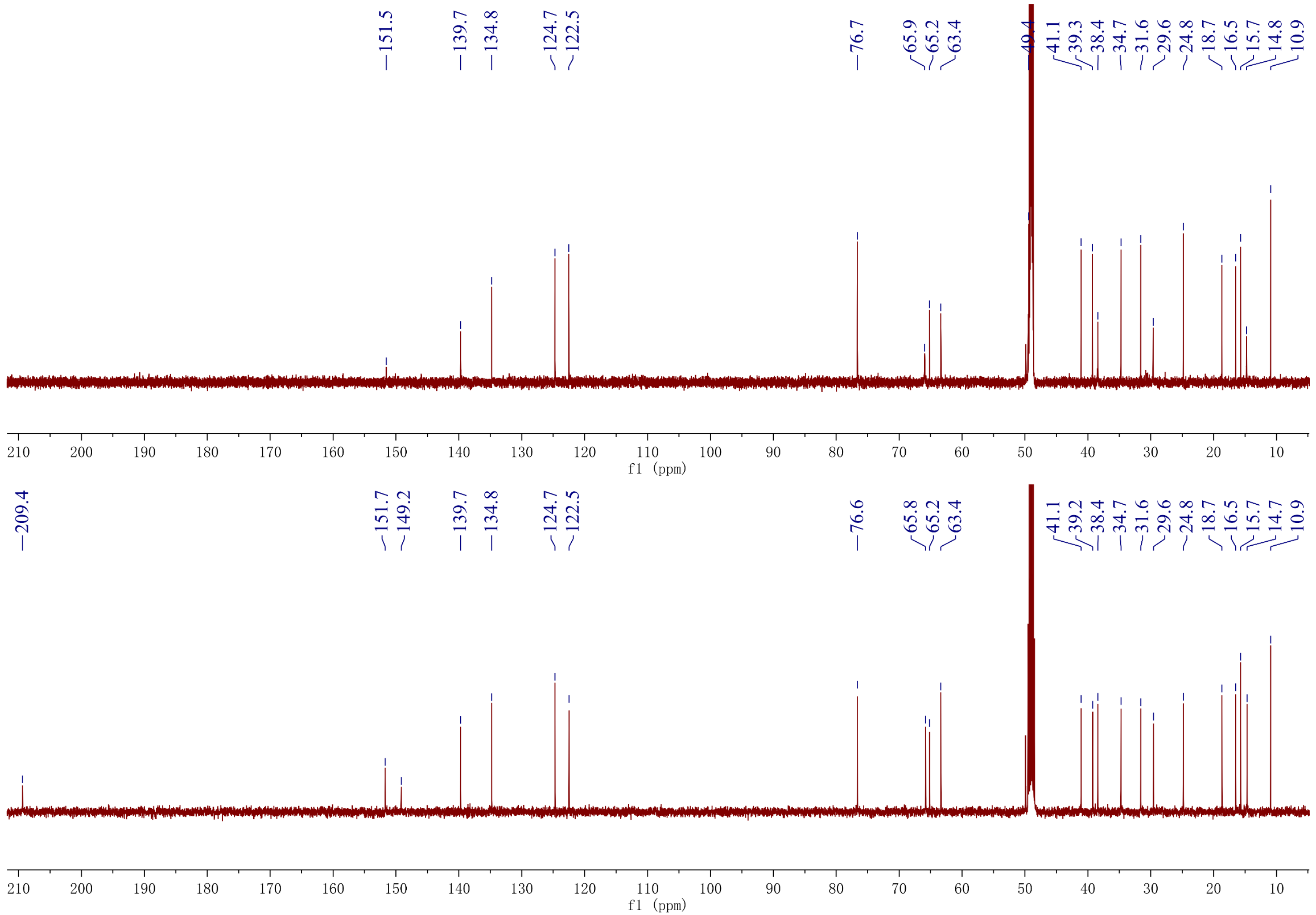

Figure S187. Comparison of ^{13}C NMR spectra of natural **12** (Methanol-d_4, upper) and synthetic **13** (Methanol-d_4, below).

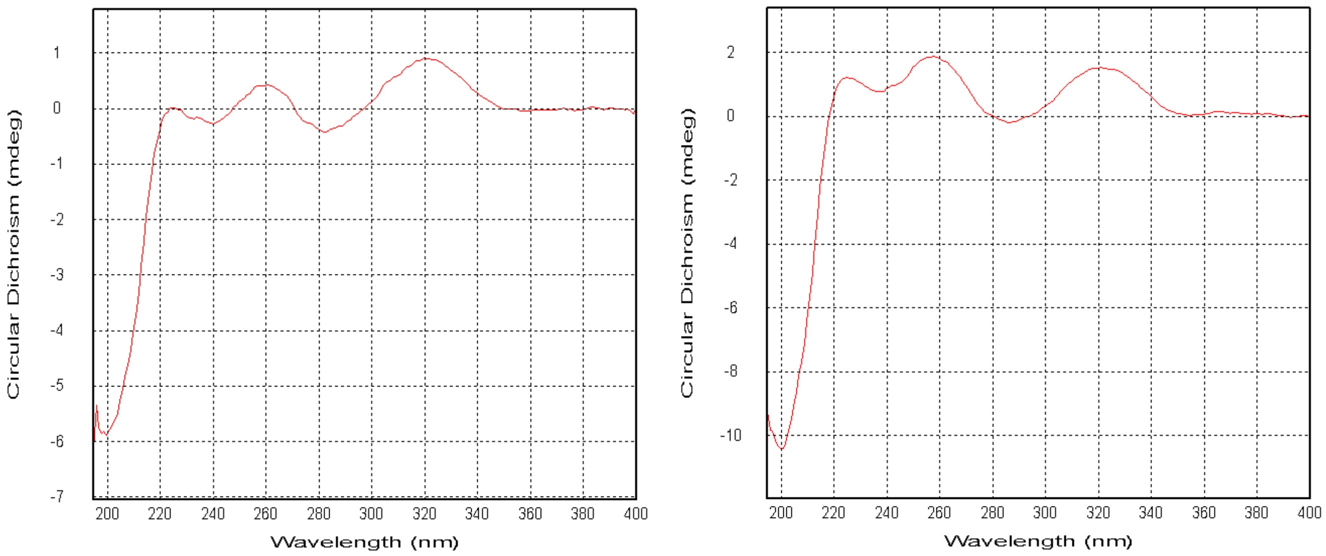

Figure S188. Comparison of ECD spectra of natural **12** (left) and synthetic **12** (right).

Rudolph Research Analytical

This sample was measured on an Autopol VI, Serial #91058
Manufactured by Rudolph Research Analytical, Hackettstown, NJ, USA.

Measurement Date : Monday, 21-APR-2025

Set Temperature : OFF

Time Delay : Disabled

Delay between Measurement : Disabled

n	Average	Std.Dev.	% RSD	Maximum	Minimum
5	7.80	0.41	5.25	8.25	7.25

S.No	Sample ID	Time	Result	Scale	OR °Arc	WLG.nm	Lg.mm	Conc.g/100ml	Temp.
1	SHSJ40	01:36:57 PM	8.00	SR	0.0032	589	100.00	0.040	27.2
2	SHSJ40	01:37:06 PM	8.25	SR	0.0033	589	100.00	0.040	27.2
3	SHSJ40	01:37:14 PM	8.00	SR	0.0032	589	100.00	0.040	27.2
4	SHSJ40	01:37:22 PM	7.50	SR	0.0030	589	100.00	0.040	27.1
5	SHSJ40	01:37:31 PM	7.25	SR	0.0029	589	100.00	0.040	27.1

Rudolph Research Analytical

This sample was measured on an Autopol VI, Serial #91058
Manufactured by Rudolph Research Analytical, Hackettstown, NJ, USA.

Measurement Date : Monday, 21-APR-2025

Set Temperature : OFF

Time Delay : Disabled

Delay between Measurement : Disabled

n	Average	Std.Dev.	% RSD	Maximum	Minimum
5	7.20	0.47	6.52	8.00	6.80

S.No	Sample ID	Time	Result	Scale	OR °Arc	WLG.nm	Lg.mm	Conc.g/100ml	Temp.
1	SHSJR8	01:42:42 PM	7.20	SR	0.0036	589	100.00	0.050	27.2
2	SHSJR8	01:42:50 PM	7.00	SR	0.0035	589	100.00	0.050	27.2
3	SHSJR8	01:42:58 PM	8.00	SR	0.0040	589	100.00	0.050	27.1
4	SHSJR8	01:43:06 PM	6.80	SR	0.0034	589	100.00	0.050	27.1
5	SHSJR8	01:43:15 PM	7.00	SR	0.0035	589	100.00	0.050	27.1

Figure S189. Comparison of ORD reports of natural **12** (upper) and synthetic **12** (below).

User Spectra

Fragmentor Voltage	Collision Energy	Ionization Mode
135	0	ESI

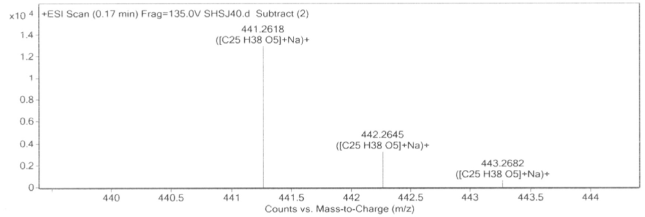

Peak List

m/z	z	Abund	Formula	Ion
274.2738	1	8199.57		
318.301	1	6034.8		
327.2012	1	7355.6		
344.2282	1	7035.6		
371.2279	1	5275.49		
388.2543	1	15162.05		
419.2791	1	10679.51		
432.281	1	17964.26		
441.2618	1	12961.15	C25 H38 O5	(M+Na)+
476.3068	1	17053.39		
520.3339	1	12448.07		
564.3602	1	8296.97		

Formula Calculator Element Limits

Element	Min	Max
C	3	60
H	0	120
O	0	30
N	0	5

Formula Calculator Results

Formula	CalculatedMass	CalculatedMz	Mz	Diff. (mDa)	Diff. (ppm)	DBE
C25 H38 O5	418.2719	441.2611	441.2618	-0.70	-1.59	7.0000

--- End Of Report ---

User Spectra

Fragmentor Voltage	Collision Energy	Ionization Mode
165	30	ESI

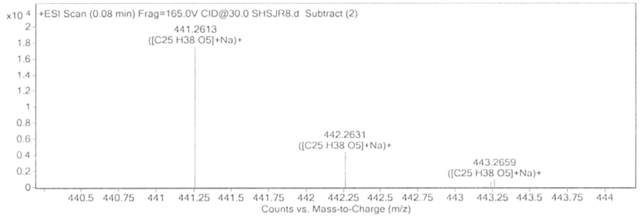

Peak List

m/z	z	Abund	Formula	Ion
81.0695	1	2585.86		
93.0697	1	3540.15		
119.0852	1	3055.99		
121.1002	1	4742.91		
133.1008	1	3439		
135.1159	1	3732.16		
441.2613	1	17524.14	C25 H38 O5	(M+Na)+
442.2631	1	4413.32	C25 H38 O5	(M+Na)+
457.2551	1	6040.37		
487.2655	1	6091.18		

Formula Calculator Element Limits

Element	Min	Max
C	3	60
H	0	150
O	0	20
N	0	5

Formula Calculator Results

Formula	CalculatedMass	CalculatedMz	Mz	Diff. (mDa)	Diff. (ppm)	DBE
C25 H38 O5	418.2719	441.2611	441.2613	-0.20	-0.45	7.0000

--- End Of Report ---

Figure S190. Comparison of HRESIMS spectra of natural **12** (left) and synthetic **12** (right).

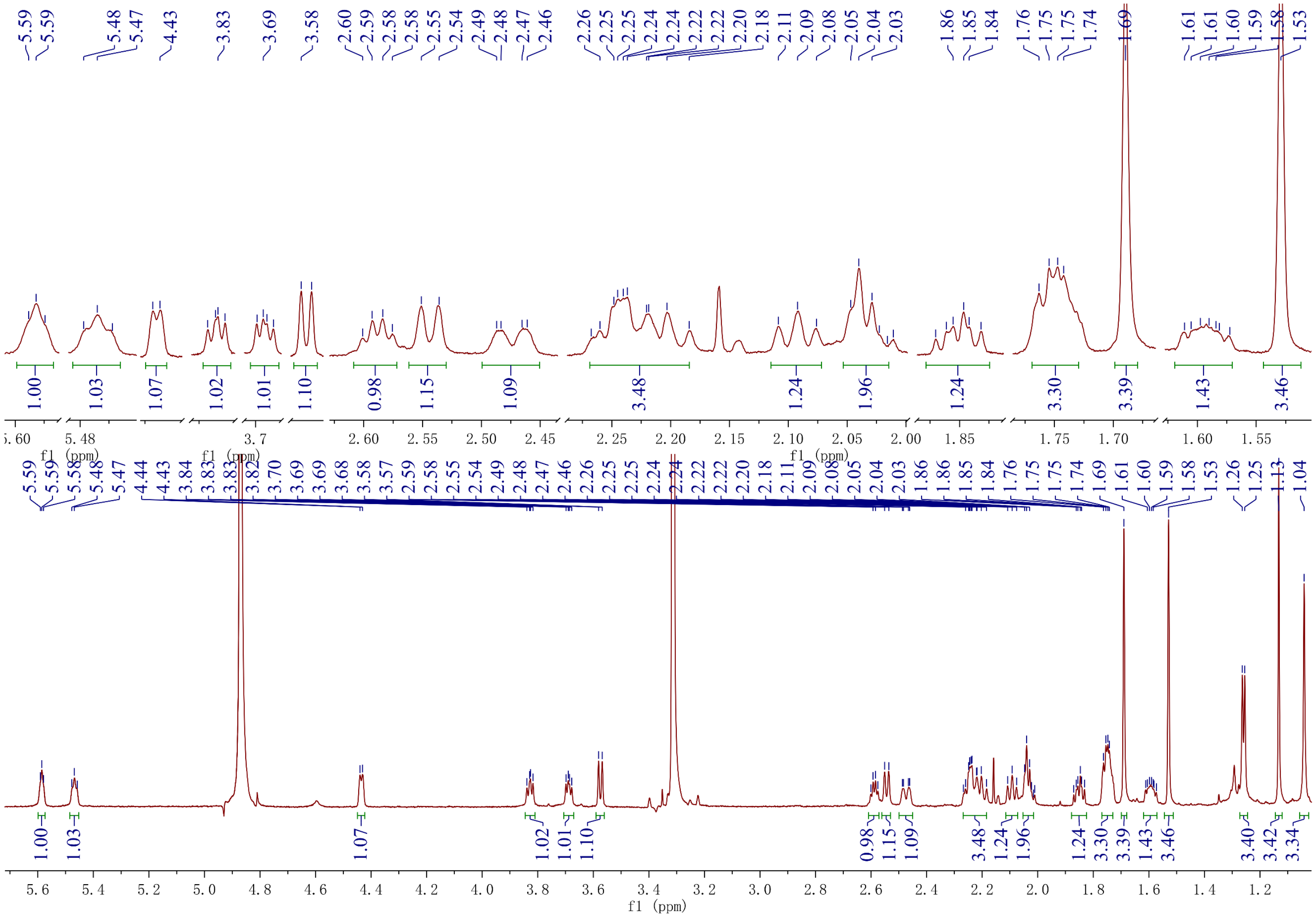

Figure S191. ^{1}H NMR spectrum of synthetic **13** (Methanol-d_4).

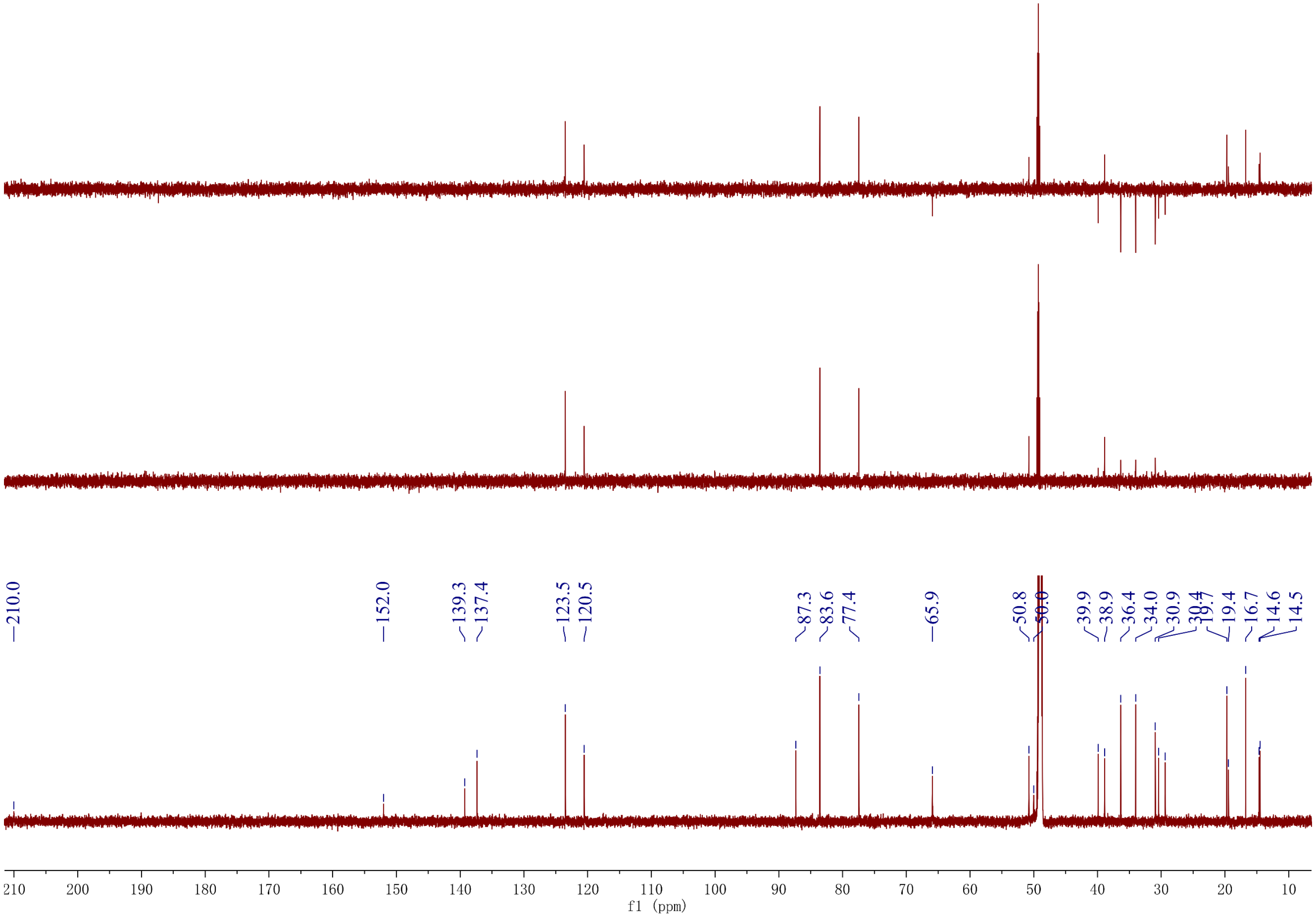

Figure S192. ^{13}C NMR and DEPT spectrum of synthetic **13** (Methanol-d_4).

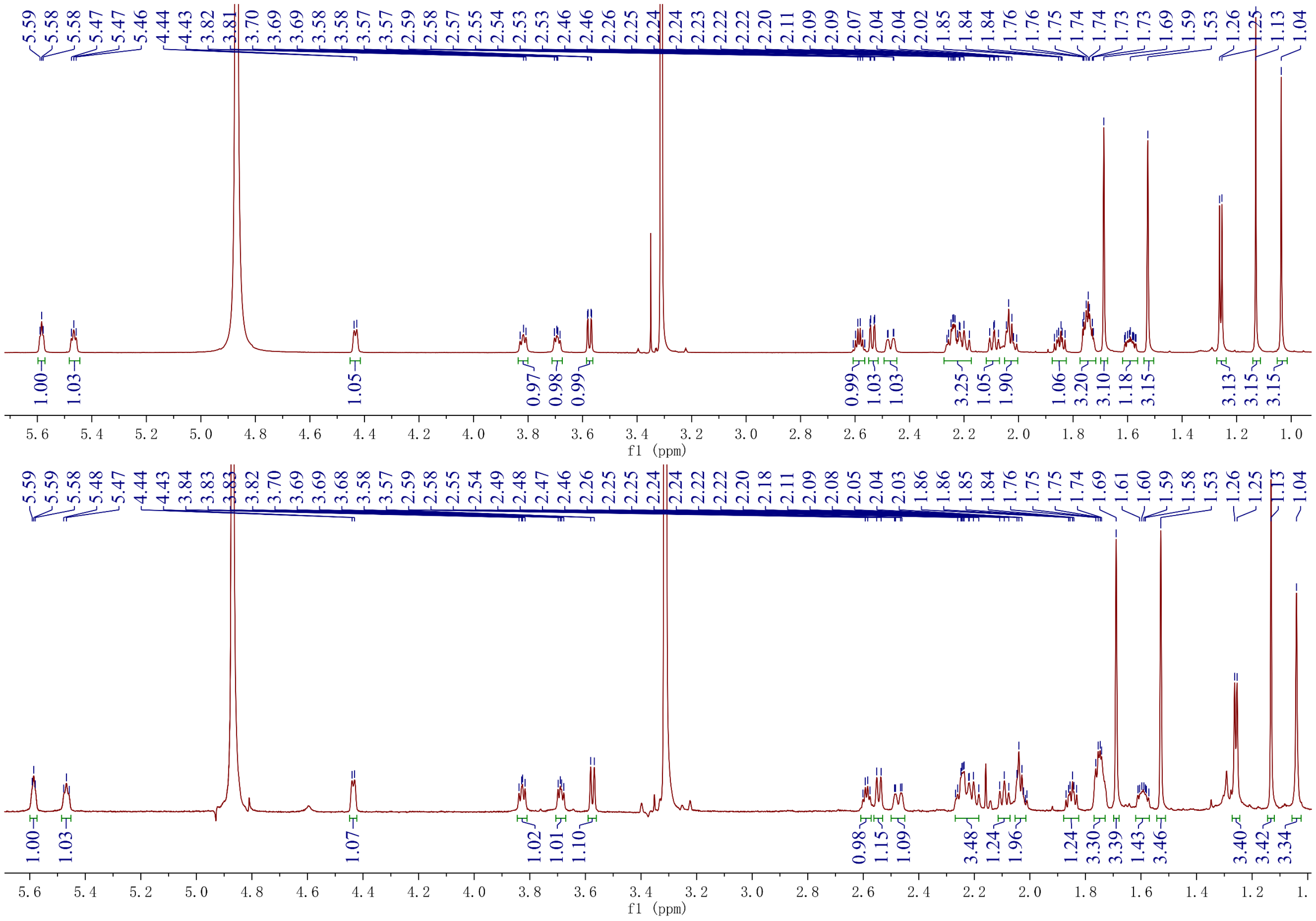

Figure S193. Comparison of ^{1}H NMR spectra of natural **13** (Methanol-d_4, upper) and synthetic **13** (Methanol-d_4, below).

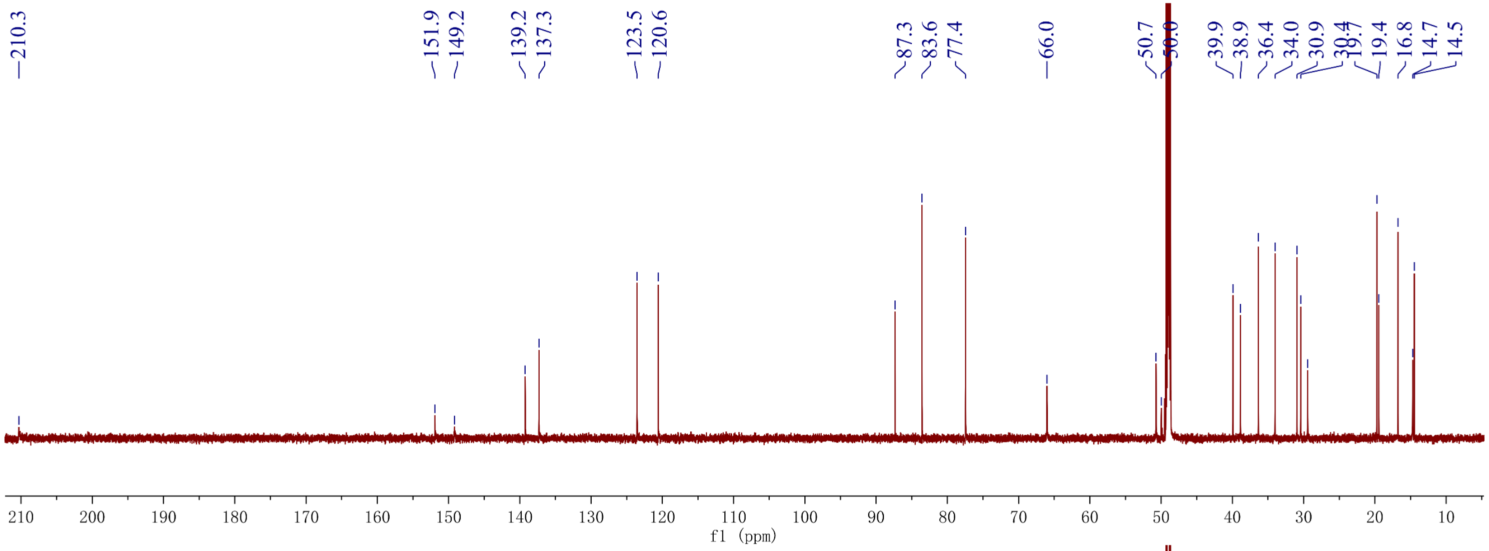

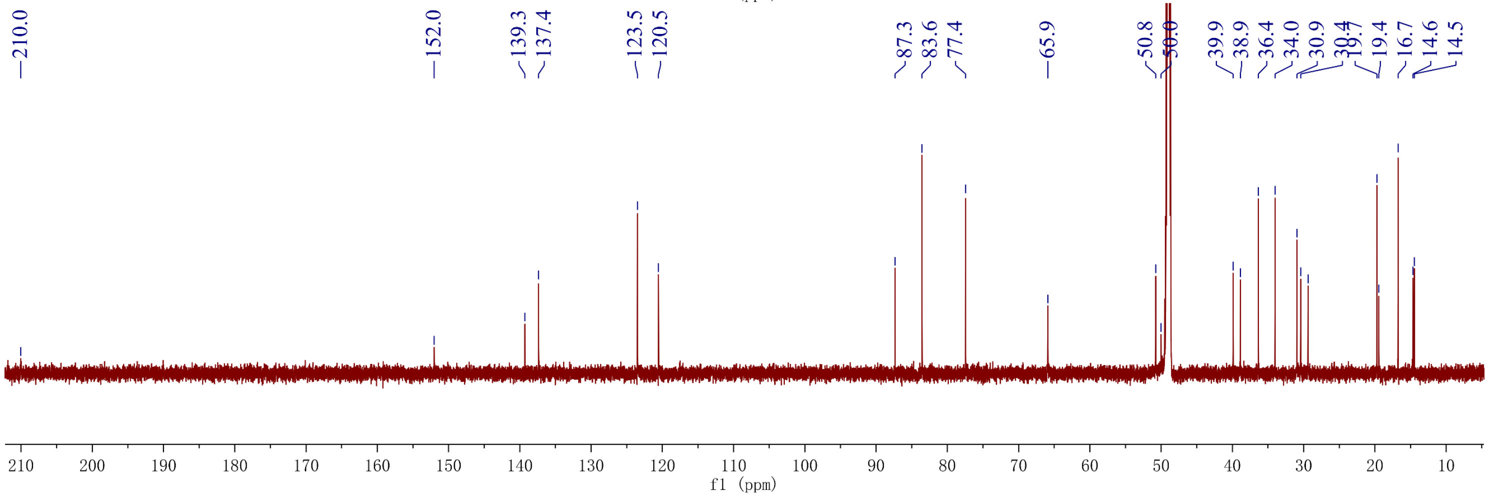

Figure S194. Comparison of ^{13}C NMR spectra of natural **13** (Methanol-d_4, upper) and synthetic **13** (Methanol-d_4, below).

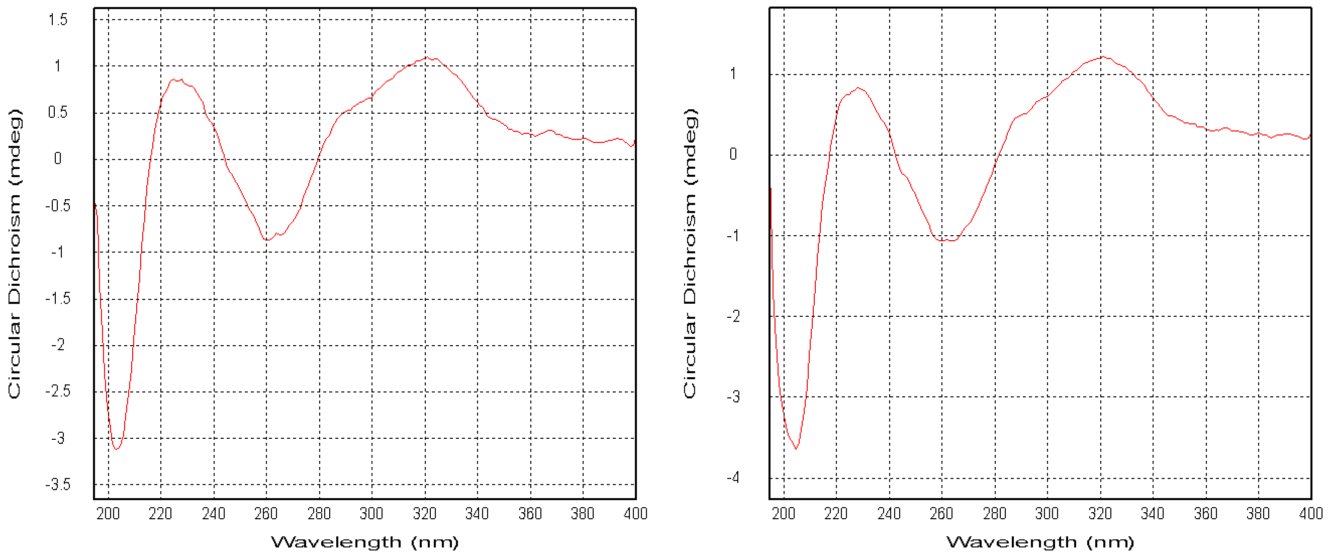

Figure S195. Comparison of ECD spectra of natural **13** (left) and synthetic **13** (right).

Rudolph Research Analytical

This sample was measured on an Autopol VI, Serial #91058
Manufactured by Rudolph Research Analytical, Hackettstown, NJ, USA.

Measurement Date : Wednesday, 02-JUL-2025

Set Temperature : 25.0

Time Delay : Disabled

Delay between Measurement : Disabled

n	Average	Std.Dev.	% RSD	Maximum	Minimum
5	-6.03	1.74	-28.85	-4.76	-7.94

S.No	Sample ID	Time	Result	Scale	OR °Arc	WLG.nm	Lg.mm	Conc.g/100ml	Temp.
1	SHSJ31	04:56:32 PM	-7.94	SR	-0.005	589	100.00	0.063	25.0
2	SHSJ31	04:56:38 PM	-7.94	SR	-0.005	589	100.00	0.063	25.0
3	SHSJ31	04:56:44 PM	-4.76	SR	-0.003	589	100.00	0.063	25.0
4	SHSJ31	04:56:51 PM	-4.76	SR	-0.003	589	100.00	0.063	25.0
5	SHSJ31	04:56:57 PM	-4.76	SR	-0.003	589	100.00	0.063	25.0

Rudolph Research Analytical

This sample was measured on an Autopol VI, Serial #91058
Manufactured by Rudolph Research Analytical, Hackettstown, NJ, USA.

Measurement Date : Wednesday, 02-JUL-2025

Set Temperature : 25.0

Time Delay : Disabled

Delay between Measurement : Disabled

n	Average	Std.Dev.	% RSD	Maximum	Minimum
5	-0.53	0.72	-135.84	0.00	-1.32

S.No	Sample ID	Time	Result	Scale	OR °Arc	WLG.nm	Lg.mm	Conc.g/100ml	Temp.
1	SHSJR7	05:11:42 PM	0.00	SR	0.000	589	100.00	0.076	25.0
2	SHSJR7	05:11:49 PM	0.00	SR	0.000	589	100.00	0.076	25.0
3	SHSJR7	05:11:55 PM	-1.32	SR	-0.001	589	100.00	0.076	25.0
4	SHSJR7	05:12:01 PM	0.00	SR	0.000	589	100.00	0.076	25.0
5	SHSJR7	05:12:08 PM	-1.32	SR	-0.001	589	100.00	0.076	25.0

Figure S196. Comparison of ORD reports of natural **13** (upper) and synthetic **13** (below).

User Spectra

Fragmentor Voltage	Collision Energy	Ionization Mode
250	0	ESI

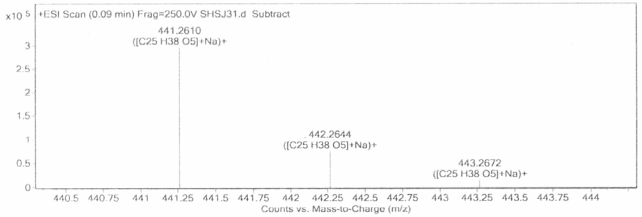

Peak List

m/z	z	Abund	Formula	Ion
441.261	1	295883.03	C25 H38 O5	(M+Na)+
442.2644	1	75607.93	C25 H38 O5	(M+Na)+
443.2672	1	14855.71	C25 H38 O5	(M+Na)+
463.242	1	30635.97		
464.2452	1	8597.86		
473.249	1	12152.49		
489.2447	1	10649.7		
859.5319	1	55033.84		
860.5357	1	29617.46		
861.538	1	9943.01		

Formula Calculator Element Limits

Element	Min	Max
C	3	60
H	0	200
O	0	20

Formula Calculator Results

Formula	CalculatedMass	CalculatedMz	Mz	Diff. (mDa)	Diff. (ppm)	DBE
C25 H38 O5	418.2719	441.2611	441.2610	0.10	0.23	7.0000

--- End Of Report ---

User Spectra

Fragmentor Voltage	Collision Energy	Ionization Mode
135	0	ESI

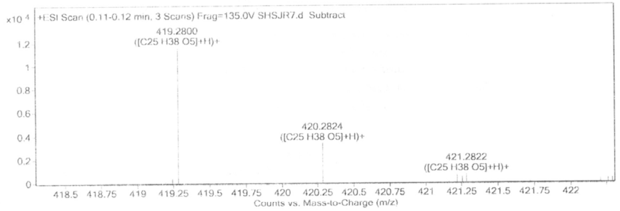

Peak List

m/z	z	Abund	Formula	Ion
240.6254	1	2894.31		
290.658	1	3169.34		
417.2644	1	2913.37		
419.28	1	11597.42	C25 H38 O5	(M+H)+
420.2824	1	3381.29	C25 H38 O5	(M+H)+
433.2584	1	2949.46		
435.2732	1	2417.13		
441.2615	1	4198.82		
449.2523	1	2889.22		
465.2484	1	2564.9		

Formula Calculator Element Limits

Element	Min	Max
C	3	50
H	0	100
O	0	20

Formula Calculator Results

Formula	CalculatedMass	CalculatedMz	Mz	Diff. (mDa)	Diff. (ppm)	DBE
C25 H38 O5	418.2719	419.2792	419.2800	-0.80	-1.91	7.0000

--- End Of Report ---

Figure S197. Comparison of HRESIMS spectra of natural **13** (left) and synthetic **13** (right).

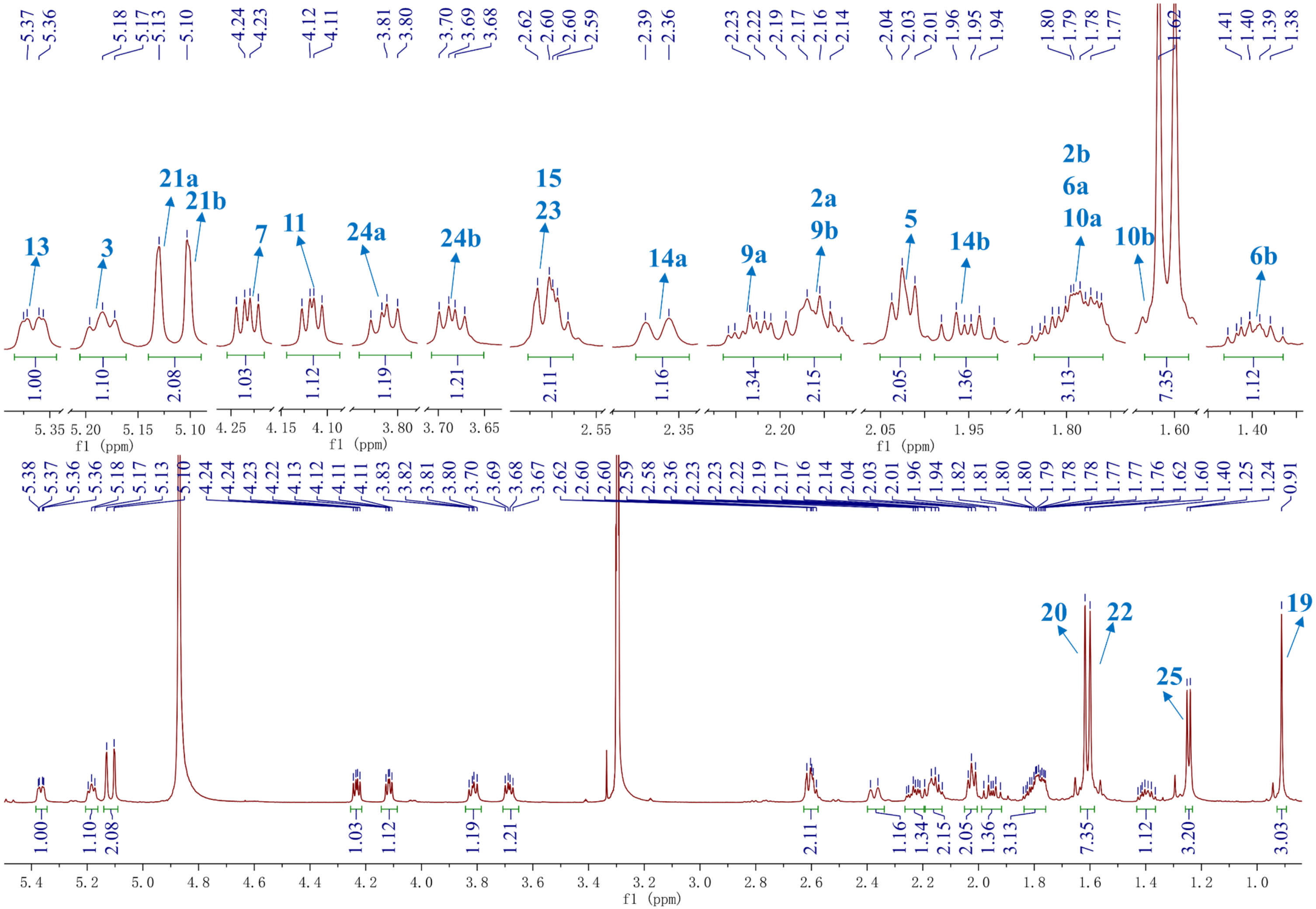

Figure S198. ^{1}H NMR spectrum of synthetic **16** (Methanol-d_4).

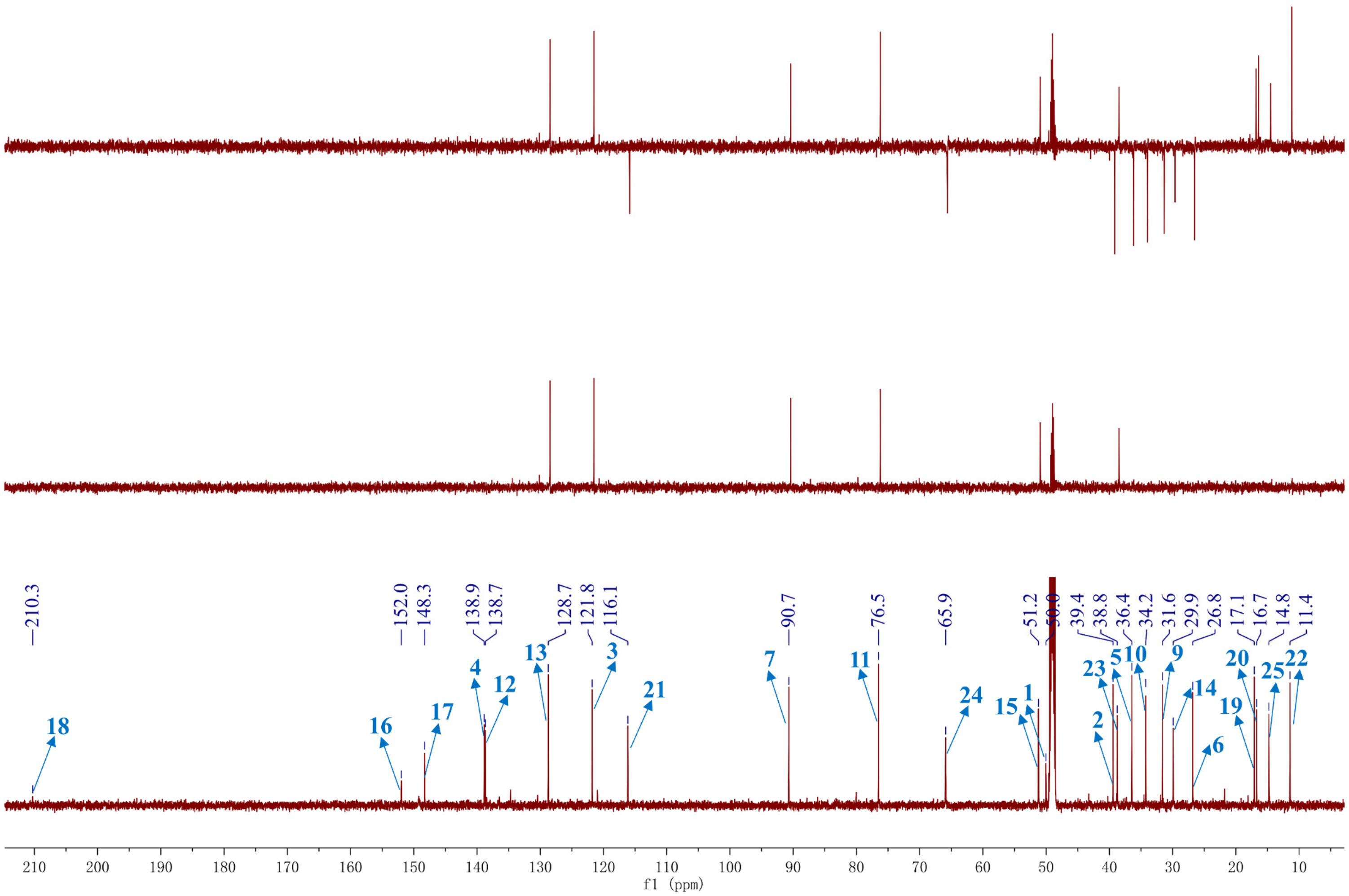

Figure S199. ^{13}C NMR and DEPT spectrum of synthetic **16** (Methanol-d_4).

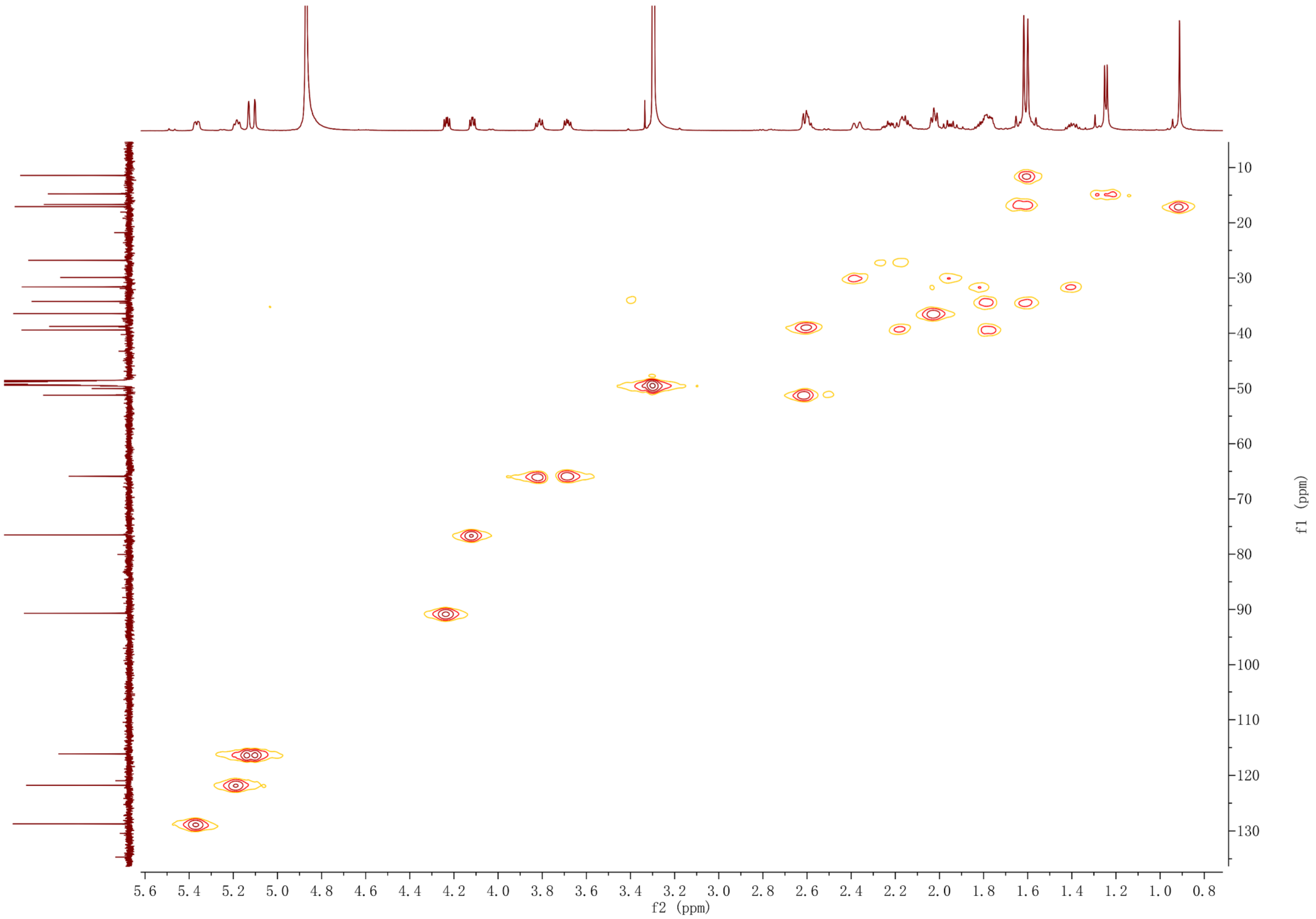

Figure S200. HSQC spectrum of synthetic **16** (Methanol-d_4).

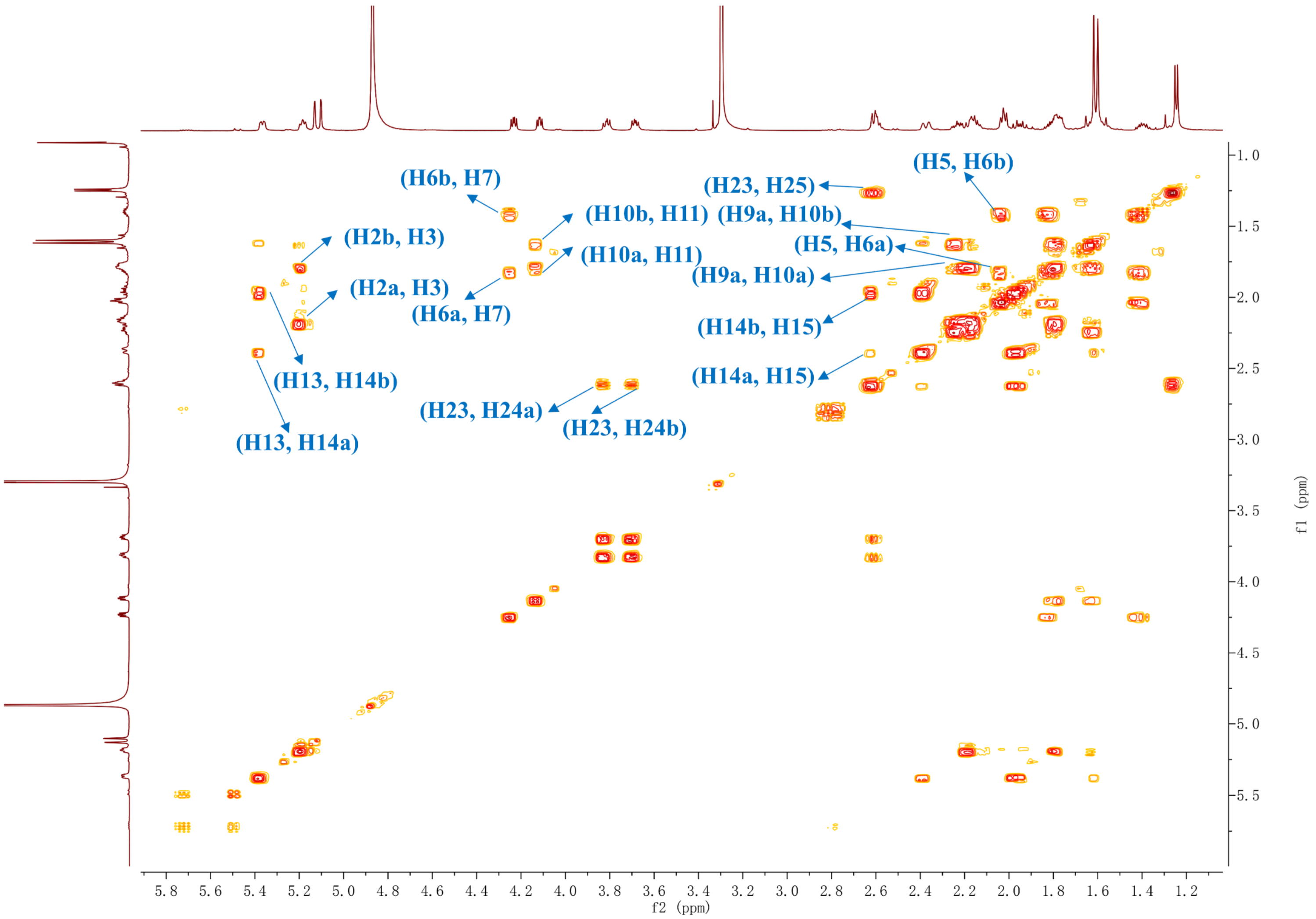

Figure S201. ^{1}H-^{1}H COSY spectrum of synthetic **16** (Methanol-d_4) and the selected cross-peaks (blue arrows and texts).

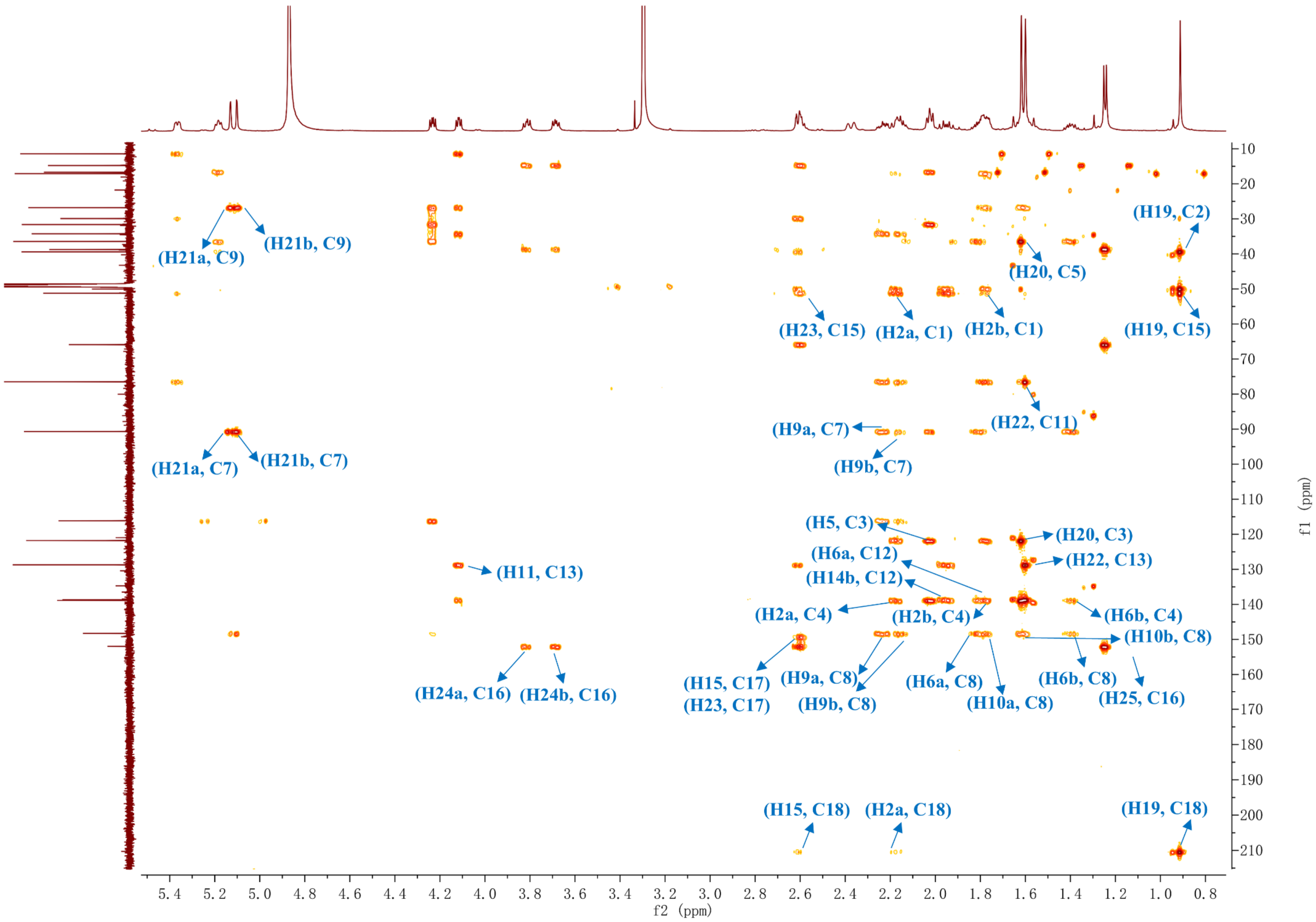

Figure S202. HMBC spectrum of synthetic **16** (Methanol-d_4) and the selected correlations (blue arrows and texts).

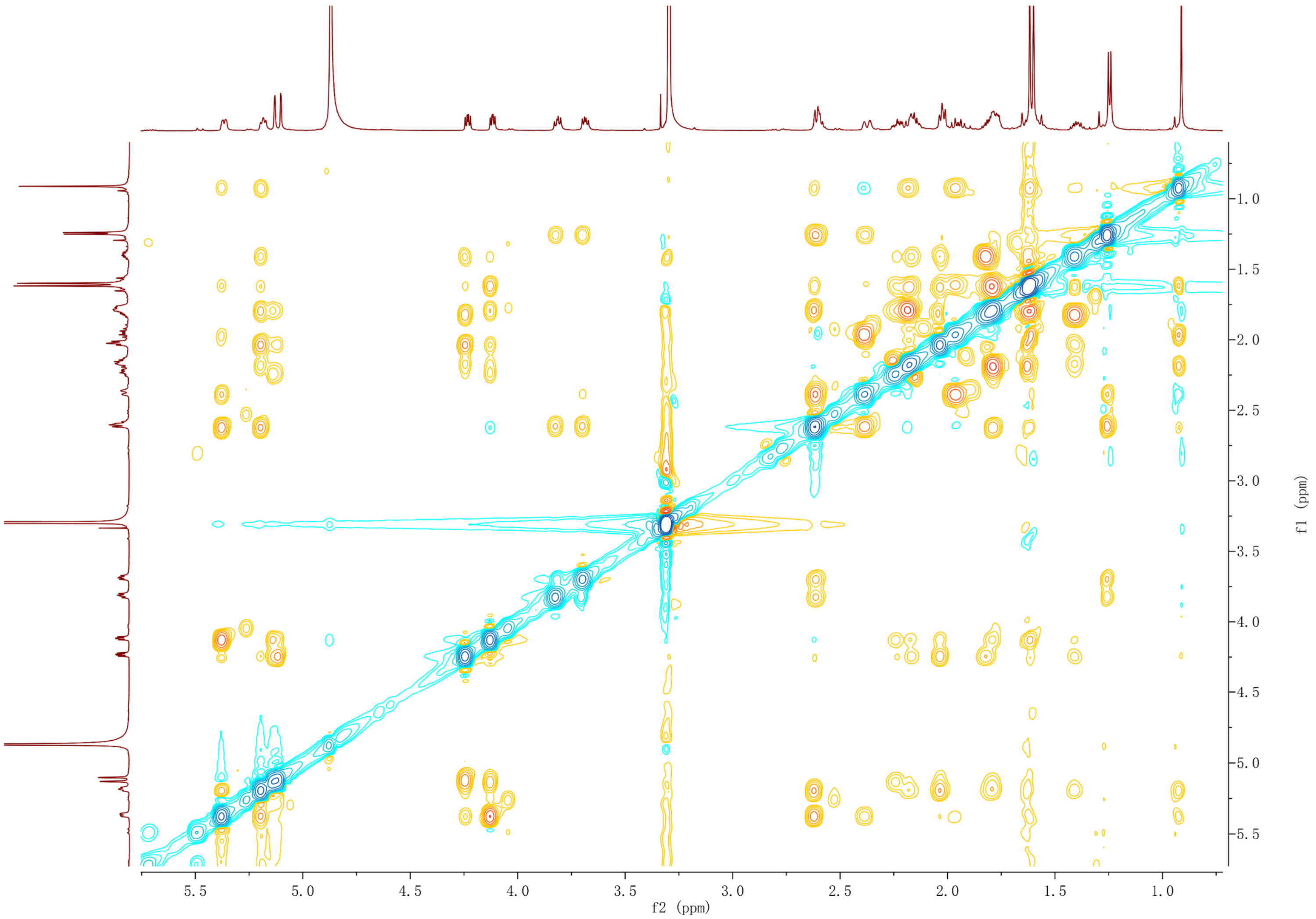

Figure S203. ROESY spectrum of synthetic **16** (Methanol-d_4) and the selected correlations (blue arrows and texts).

User Spectra

Fragmentor Voltage	Collision Energy	Ionization Mode
135	0	ESI

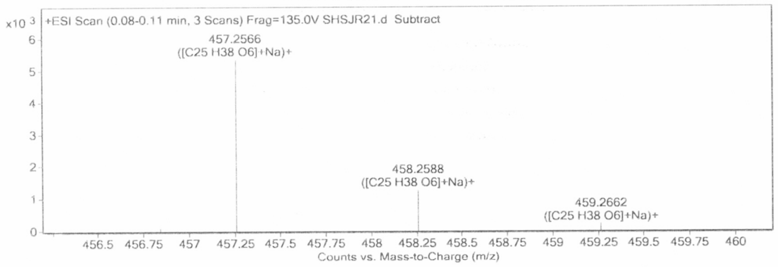

Peak List

m/z	z	Abund	Formula	Ion
142.9485	1	804.41		
399.2539	1	1977.72		
401.2683	1	1240.28		
417.2639	1	3900.6		
418.2672	1	1229.19		
435.2743	1	4460.06		
436.2772	1	972.66		
457.2566	1	5346.81	C25 H38 O6	(M+Na)+
458.2588	1	1264.6	C25 H38 O6	(M+Na)+
503.3133	1	1059.43		

Formula Calculator Element Limits

Element	Min	Max
C	3	60
H	0	200
O	0	20

Formula Calculator Results

Formula	CalculatedMass	CalculatedMz	Mz	Diff. (mDa)	Diff. (ppm)	DBE
C25 H38 O6	434.2668	457.2561	457.2566	-0.50	-1.09	7.0000

--- End Of Report ---

Figure S204. HRESIMS spectrum of synthetic **16**.
